# Dynamic stereomutation of vinylcyclopropanes with metalloradicals

**DOI:** 10.1038/s41586-024-07555-1

**Published:** 2024-06-19

**Authors:** Marvin Mendel, Teresa M. Karl, Jegor Hamm, Sherif J. Kaldas, Theresa Sperger, Bhaskar Mondal, Franziska Schoenebeck

**Affiliations:** Institute of Organic Chemistry, RWTH Aachen University, Aachen, Germany

**Keywords:** Synthetic chemistry methodology, Reaction mechanisms, Homogeneous catalysis

## Abstract

The ever increasing demands for greater sustainability and lower energy usage in chemical processes call for fundamentally new approaches and reactivity principles. In this context, the pronounced prevalence of odd-oxidation states in less precious metals bears untapped potential for fundamentally distinct reactivity modes via metalloradical catalysis^1–3^. Contrary to the well-established reactivity paradigm that organic free radicals, upon addition to a vinylcyclopropane, lead to rapid ring opening under strain release—a transformation that serves widely as a mechanistic probe (radical clock)^4^ for the intermediacy of radicals^5^—we herein show that a metal-based radical, that is, a Ni^(I)^ metalloradical, triggers reversible *cis*/*trans* isomerization instead of opening. The isomerization proceeds under chiral inversion and, depending on the substitution pattern, occurs at room temperature in less than 5 min, requiring solely the addition of the non-precious catalyst. Our combined computational and experimental mechanistic studies support metalloradical catalysis as origin of this profound reactivity, rationalize the observed stereoinversion and reveal key reactivity features of the process, including its reversibility. These insights enabled the iterative thermodynamic enrichment of enantiopure *cis*/*trans* mixtures towards a single diastereomer through multiple Ni^(I)^ catalysis rounds and also extensions to divinylcyclopropanes, which constitute strategic motifs in natural product- and total syntheses^6^. While the *trans*-isomer usually requires heating at approximately 200 °C to trigger thermal isomerization under racemization to *cis*-divinylcyclopropane, which then undergoes facile Cope-type rearrangement, the analogous contra-thermodynamic process is herein shown to proceed under Ni^(I)^ metalloradical catalysis under mild conditions without any loss of stereochemical integrity, enabling a mild and stereochemically pure access to seven-membered rings, fused ring systems and spirocycles.

## Abstract (web summary)

A Ni^(I)^ metalloradical catalyst enables reversible *cis*/*trans*-isomerization of vinylcyclopropanes under chiral inversion.

## Main

**Fig. 1:**
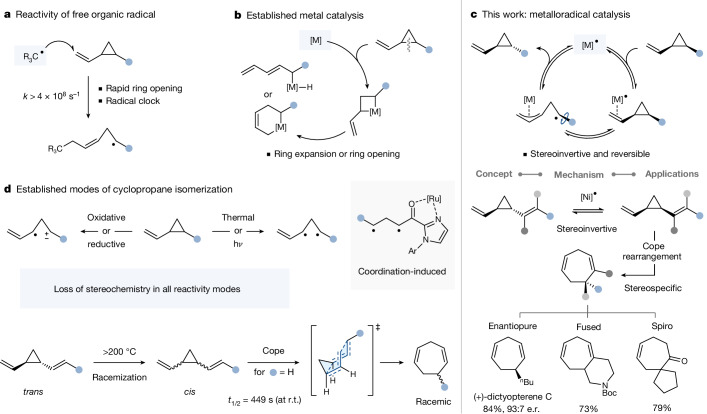
Reactivity modes of (di)vinylcyclopropanes. **a**–**c**, Reactivity of organic radicals (**a**), metals (**b**) and metalloradicals (**c**, this work) towards vinylcyclopropanes. **d**, Established modes of isomerization. e.r., enantiomeric ratio; r.t., room temperature.

Whereas the reactivity principles of organic free radicals are well established and have found widespread implementation in synthetic methodology developments^5^, there is comparably much less application of metalloradical-based reactivity, especially with regard to homogeneous catalysis and the discovery of potentially unique and enabling reactivity modes. Metalloradical catalysis operates via open-shell metal complexes and intermediates throughout the entire catalytic cycle^1–3^. Exemplary manifestations of this concept in synthesis include the radical relay catalysis of Cu^(I)^, Ti^(III)^ (refs. ^7,8^), porphyrin-type Co^(II)^ or Fe^(III)^ complexes^9^, which are based on the transposition of the radical character from the metal to the organic moiety through covalent bonding (Fig. 1). These catalytically generated radicals have been shown to give tamed reactivity^10^ compared with organic free radicals that do not have the metal bound—a feature also harnessed in several metalloenzymes. However, compared with omnipresent homogeneous metal catalysis based on closed-shell species, there is comparably much less exploitation and implementation of metalloradical catalysis in synthesis. To meet the ever increasing demands for greater sustainability, and to capitalize on the frequent occurrence of odd-oxidation states in non-precious metals, a greater understanding of the principles and potential of metalloradical catalysis would therefore be greatly enabling.

We herein disclose a metalloradical catalysis mode that is initiated by non-covalent interaction via π-coordination, which leads to reactivity that is fundamentally in contrast to organic free radical reactivity and enables a reversible *cis*/*trans*-isomerization of vinylcyclopropanes under chiral inversion.

Vinylcyclopropanes serve as valuable synthetic precursors for cycloadditions or rearrangements and are also key structural units in natural products and bioactive compounds, such as the antivirals Simeprevir (against hepatitis C) or Danoprevir (against COVID-19)^11,12^. The geometry of the vinylcyclopropane, that is, *cis* versus *trans*, impacts its overall function, and stereoselective synthetic approaches to these motifs have been developed accordingly^13,14^. Although remarkable developments have been achieved, the selectivity of cyclopropane construction can be substrate-specific or involve multistep processes, especially in efforts to reach high enantioselectivity along with *cis*/*trans* selectivity, which is well addressed for higher substituted cyclopropanes, but still challenging for 1,2-disubstituted analogues^13,15–17^. A complementary strategy would be the unselective (and potentially enantiopure) synthesis of *cis*/*trans* mixtures of vinylcyclopropane derivatives, followed by their isomerization towards a single isomer. Ideally, this process is steerable towards either isomer without compromises on enantiopurity. However, reversible *cis*/*trans* isomerization without loss of enantiopurity is fundamentally unknown. If realizable, it could find numerous applications, for example, in strategic uses of divinylcyclopropanes. Such species are more (and sometimes solely) stable in their *trans*-geometry and feature in several natural products, while the *cis*-isomer is prone to undergo rearrangement^6,18^. This orbital-symmetry-controlled stereo-retentive electrocyclic reaction has served as a key strategic transformation in total syntheses, but frequently requires the *cis*-isomer to be made in situ (owing to its high reactivity), which was achieved predominantly through direct construction of the cyclopropane ring. Construction of the more stable *trans*-divinylcyclopropane, followed by isomerization to *cis* requires high temperature (approximately 200 °C) and occurs under racemization^19–22^. Such thermally or photochemically induced homolytic scissions towards diradical intermediates^23^ can also trigger the *cis*-to-*trans* isomerization of alternatively substituted cyclopropanes and, with appropriate metal complexation for specific substrates, also at lower temperature (80 °C)^24^, albeit under loss of enantiopurity towards the racemic, thermodynamic *trans*-isomer. Isomerization may also occur in a polar mechanism via zwitterionic intermediates, oxidatively^25^, reductively or under Lewis acid catalysis for specific substrates with the analogous challenges on enantiopurity (that is, its loss)^18^, although remarkable strides towards photochemically assisted chiral resolution with a chiral catalyst have been made recently for specifically substituted cyclopropyl ketones^26,27^. There are isolated reports of *cis*/*trans* isomerization with a precious metal (Au, Rh)^28–31^ at elevated temperature for which the wider potential (that is, scope) and stereospecificity have not (yet) been established. On the other hand, transition metal catalysis based on Ni, Pd, Rh, Ir or Fe has been explored for their addition to vinylcyclopropanes and subsequent follow up chemistry (for example, rearrangement, cycloaddition) towards products of decreased ring-strain^32–35^.

We reported previously that the *N*-heterocyclic carbene (1,3-bis(2,6-diisopropylphenyl)-1,3-dihydro-2*H*-imidazol-2-ylidene; IPr)-derived Ni^(I)^ dimer **1** interacts with a double bond to trigger homolytic Ni–Ni scission and formation of an olefin-bound Ni^(I)^ metalloradical complex^36^. The radical character in this π-coordinated metalloradical is located primarily at the Ni-centre, and this metalloradical consequently differs from covalently attached metalloradicals as introduced above, where the radical character is transposed primarily to the organic moiety via π-bond cleavage and formation of a C_*sp*2_-hybridized carbon-centred radical. The Ni^(I)^ metalloradical–olefin complex was found previously to have an inherent driving force for reorganization, which was leveraged in selective olefin migration^36^. Our preliminary tests with a specific diphenyl substituted vinylcyclopropane indicated a partial isomerization instead of opening, which fundamentally questions the validity of vinylcyclopropanes as mechanistic probes to test for metal-based radical intermediates.

To date, neither the factors that dictate isomerization versus opening have been elucidated, nor the wider scope and potential of this reactivity in synthesis. We therefore set out to resolve these fundamental questions with a view to devise a selective and general isomerization for vinylcyclopropanes that proceeds without any loss of enantiopurity.

We initially set out to study the reactivity of Ni^(I)^ dimer **1** with the 4-methoxyphenyl-substituted vinylcyclopropane **2** and evaluated various reaction parameters, which ultimately revealed that, with only 1 mol% of catalyst at room temperature, the *cis*-cyclopropane **2** was fully transformed to the corresponding *trans*-isomer within 5 min (in 90:10 *trans:cis* selectivity and 98% yield) in dioxane (or tetrahydrofuran). Such mildness and extreme speed under such low catalyst loading was remarkable, considering that the Ni-free isomerization requires heating at over 200 °C (ref. ^20^).

**Fig. 2:**
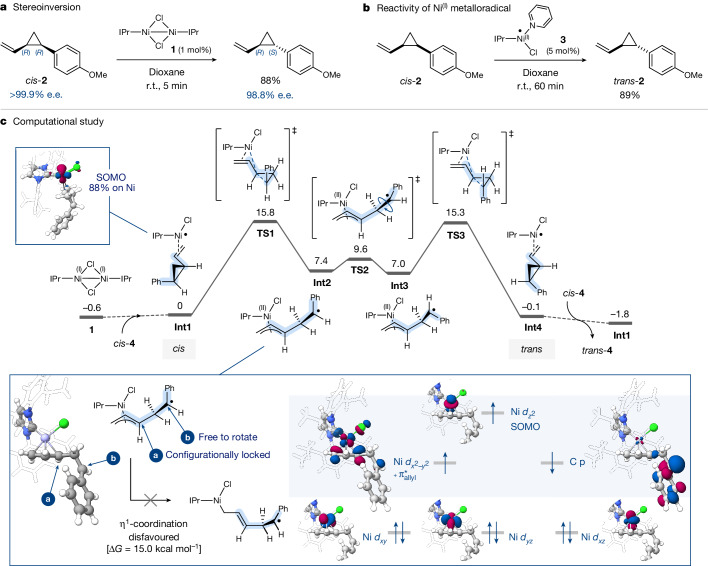
Mechanistic insight. **a**, Selective stereoinversion of distal stereocentre. **b**, Ni^(I)^ metalloradical reactivity. **c**, Computational study of reaction pathway and orbital analysis of intermediate (**Int2**).

As outlined above, the thermal isomerization is postulated to proceed via homolytic scission and diradical intermediates that is accompanied with loss of stereochemical information if a chiral starting material is used. We therefore next examined the isomerization for its potential to retain enantiopurity. To this end, we prepared (*R,R*)-*cis*-**2** in >99.9% enantiomeric excess (e.e.) and subjected it to the same isomerization conditions (Fig. 2a). The corresponding *trans*-product **2** was generated under retention of enantiopurity in 98.8% e.e. (in 88% yield) within 5 min at room temperature using 1 mol% of Ni^(I)^ dimer **1**. To the best of our knowledge, such a mild and rapid *cis*/*trans* isomerization of vinylcyclopropanes without any loss of enantiopurity is unprecedented to date.

Our closer inspection of the absolute stereochemistry of the product revealed that the carbon distal from the vinyl substituent was inverted selectively during isomerization, having generated (*R,S*)-*trans-***2** (Fig. 2a). Ni^(I)^ therefore must have a profound effect on the overall reaction path, in locking the stereocentre adjacent to the vinyl substituent, while also influencing the overall driving force to favour the strained, ring-closed product over ring-opened/rearranged products.

To gain greater insight, we undertook computational studies at CPCM (1,4-dioxane) M06L/def2-TZVP//MN15/6-31G(d)(SDD) level of theory, following a comprehensive methods assessment (see Supplementary Information). The calculations suggest that, owing to the relatively weak Ni–Ni bond in dimer **1**, homolytic scission towards the Ni^(I)^ monomer and π-coordination to the olefin occurs readily. The vinylcyclopropane serves solely as π-ligand to the Ni^(I)^ and no radical character is induced to the organic moiety, residing solely on the [Ni^(I)^Cl(IPr)] with 88% of the radical being located in the *d*_*xy*_ orbital of Ni^(I)^ (Fig. 2c). However, a geometric change from tetrahedral to square-planar arrangement of this metalloradical–olefin complex, which is 4.3 kcal mol^−1^ higher in free energy, leads to partial induction of radical character onto the vinylcyclopropane (see Supplementary Information for details). Subsequent cyclopropane C–C bond cleavage proceeds with an overall activation free energy barrier of 15.8 kcal mol^−1^ and is endergonic by 7.4 kcal mol^−1^. The ring-opened intermediate **Int2** is square-planar in geometry and the energetically favoured state is of broken-symmetry with two electrons in parallel spin located on the Ni^(II)^-centre and a radical located on the remote carbon, which resulted from homolytic scission of the C–C bond in the cyclopropane. Our examination of a potential alternative Ni^(I)^/Ni^(III)^ oxidative addition directly into the cyclopropane ring suggests that, although a higher energy transition state can be located for the direct addition by Ni^(I)^, no Ni^(III)^ intermediate is subsequently formed. Instead, the intrinsic reaction coordinate leads also to **Int2** (see Supplementary Information for additional details). Rotation of intermediate **Int2** is facile, proceeding with an activation free energy barrier of Δ*G*^‡^ = 2.2 kcal mol^−1^, and subsequent ring closure is thermodynamically downhill relative to the opened intermediate (Fig. 2c). Only carbon (b) is rotatable; the configuration of carbon (a) is locked through the formation of a Ni^(II)^–π–allyl complex. Our calculations suggest that any alternative isomer (or spin state) of **Int2** that could allow for carbon (a) inversion lies 7–25 kcal mol^−1^ higher in energy than the transition state for ring closure **TS3** (see Supplementary Information for additional details). Consequently, the lack of racemization observed under Ni^(I)^-catalysed isomerization (as opposed to thermal isomerization) means that, although formally in both processes a homolytic scission of the central cyclopropane bond takes place, the Ni-coordination locks one stereocentre through a π–allyl complex, leaving only one centre to rotate (and invert).

Another notable difference to organic free radical reactivity is that addition of an organic free radical, upon ring opening, delivers a more stabilized radical, both in terms of strain release and also the resonance delocalization gained through the aromatic substituent. In contrast, for the Ni^(I)^ metalloradical, the radical is clearly more stabilized on the Ni-centre in the closed cyclopropane form, in which the vinylcyclopropane serves predominantly as a π-ligand. So called ‘radical clocks’ should hence be used with caution to test for the intermediacy of metal-based radicals.

Finally, the computed profile also suggests that the Ni^(I)^-bound *cis*- and *trans*-vinylcyclopropanes have only minute differences in energies, which suggests full reversibility of the isomerization process. The equilibrium is shifted ultimately through coordination/decoordination of the Ni^(I)^ so as to reach the overall *cis*:*trans* ratio that reflects the inherent *cis*/*trans* preference of the substrate itself. For compound **4**, the observed ratio indeed matches the substrate’s free energy difference of the *cis*- and *trans*-isomer, which is 1.8 kcal mol^−1^. This suggests that the overall selectivity of the isomerization is readily predictable upon assessment of the inherent energy difference of the *cis-* and *trans-*isomer.

To further confirm the metalloradical nature of the process, we examined whether a separately synthesized IPr-ligand-derived Ni^(I)^ monomer would trigger the analogous reactivity as observed when starting from dimer **1**. To this end, we synthesized the known pyridine-coordinated (IPr)Ni^(I)^ monomer **3** (ref. ^37^) and subjected 5 mol% thereof to *cis*-**2** in dioxane at room temperature for 60 min, which resulted in the analogous isomerization to 89% of *trans*-**2** (Fig. 2b). Our electron paramagnetic resonance (EPR) measurements of **3** itself in dioxane or with added *cis*-**2** substrate gave distinct signals characteristic of the intermediacy of a metal-based radical. While the Ni^(I)^ dimer **1** in dioxane is EPR silent, consistent with its dimeric low-spin configuration, upon addition of the substrate *cis*-**2**, the EPR spectrum showed a distinct signal characteristic of a metal-based radical, which would be in line with our above calculations and proposed pathway.

Ni(cod)_2_/IPr has been used previously for the rearrangement of vinylcyclopropanes^32,38^. Calculations support the ring-opened/rearranged products to be favoured for **2** with Ni^(0)^, but the process is calculated to be at least 10 kcal mol^−1^ higher in overall activation barrier than the Ni^(I)^-based *cis*/*trans* isomerization (see Supplementary Information). Our tests with Ni(cod)_2_/IPr showed diminished reactivity with strong dependence on solvent, substrate and temperature, overall suggesting divergent catalyst speciations. Indeed, we were able to detect an EPR signal characteristic of in situ formed Ni^(I)^. In other words, Ni(cod)_2_/IPr can engage in rearrangement and/or in situ formation of Ni^(I)^ (ref. ^39^), overall leading to product mixtures (see Supplementary Information).

To test the wider synthetic potential of the isomerization, we set out to examine alternative vinylcyclopropane motifs. Variation of the aromatic-substituents had no marked impact; isomerization was equally rapid (5 min, room temperature) and efficient, yielding approximately 90:10 *trans*:*cis* selectivity with 82–98% yield with methoxy (**2**), alkyl (**5**–**6**) or trifluoromethyl-substitution (**7**) as well as heterocyclic motifs, such as a benzofuran-substituted vinylcyclopropane (**9**). The same final *cis*:*trans* ratio resulted irrespective of whether the exclusive *cis*-isomer or a *cis*/*trans* mixture was subjected to isomerization. Beyond aromatic substitution, vinylcyclopropyl ketones (aromatic and aliphatic, **10**–**12**), esters (**13**–**15**), amides (**21**–**22**) as well as Weinreb amides (**23**) also isomerized efficiently to the corresponding *trans*-products in high selectivity and yield, albeit under slightly longer reaction times of 15 min to 2 h at room temperature. Even the free carboxylic acid was well tolerated and delivered **17** within 15 min at room temperature using 5 mol% of catalyst **1**. As there was no by-product formation, purification was achieved by straightforward filtration.

We next studied whether isomerization of *N*-methyliminodiacetyl boronate (BMIDA)-, pinacol boronic ester (BPin)- and germyl-substituted vinylcyclopropanes would also be feasible. Given the enabling value of these modular platforms to access a wide range of compounds upon (stereospecific) derivatization^40^, we envisioned that the straightforward construction of these building blocks as *cis*/*trans* mixtures, followed by Ni^(I)^-catalysed enrichment to the *trans*-isomer would be of value. Indeed, we observed facile isomerization to **24**–**26**, albeit under slightly more forced conditions (60 °C and/or extended reaction time). Capitalizing on the rich stereospecific diversification chemistry of the boronic ester unit can thus allow formation of substituted *trans*-vinylcyclopropanes with rests that might either be incompatible with Ni^(I)^ catalysis or for which the inherent *cis*/*trans* stability difference of the substrate itself may not allow discrimination or induced selectivity in the reversible Ni^(I)^-catalysed isomerization process. For example, this is the case for alkyl substituted versions, for which only bulky substituents (**20**) deliver high *trans*-thermodynamic preference and selectivity.

We subsequently examined whether additional substitution on the vinyl motif could also be tolerated and synthesized alkenyl cyclopropanes (**19**, **28**–**36**) that contain internal alkenes in *E* or *Z* geometry. Although longer reaction times and elevated temperature were found to be necessary for these cases, high *trans*-yields were seen in all cases under full retention of the olefin (*Z* or *E*) geometry. There was no isomerization of the olefin; solely the cyclopropane isomerized, in line with our mechanism in Fig. 2c and the locked π–allyl configuration in **Int2**. Similarly, 1,1-disubstituted olefins (**19**, **35**–**36**) and higher substitution at the cyclopropane ring (**18**, **19**) were also tolerated and, in the absence of the vinyl group, the starting material was fully recovered (**27**).

The scalability of the process was next examined. To this end, we employed 1 g of cyclopropane **16**; since it is a liquid, we omitted any solvent and attempted the isomerization of the neat compound, adding solely 0.5 mol% of Ni^(I)^ catalyst **1**. After 3.5 h at room temperature, a 92:8 *trans*:*cis* mixture of **16** was generated and isolated in 93% yield. Neither additives nor solvent nor heating were necessary, only minute amounts of the non-precious Ni^(I)^ metalloradical was required and full atom-economy was retained in the process.

**Fig. 3:**
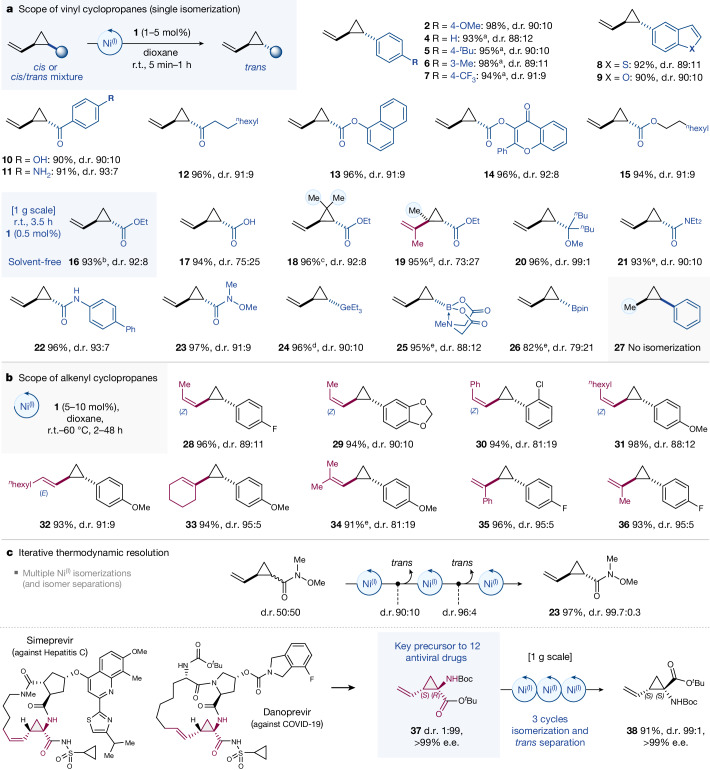
Reactivity and selectivity investigations. **a**, Scope of vinylcyclopropane isomerization. **b**, Scope of isomerization of internal alkenyl cyclopropanes. **c**, Iterative thermodynamic resolution. ^a^Yield determined by quantitative ^1^H NMR. ^b^**1** (0.5 mol%), reaction time 3.5 h. ^c^Reaction time 18–24 h at 60 °C. ^d^Reaction time 24 h. ^e^Reaction time 72 h at 60 °C.

As discussed above, the ultimate *cis*:*trans* ratio upon stereomutation depends on the inherent energetic prerequisite of the substrate itself. In light of the reversibility of the isomerization, any removal of the *trans*-isomer should then lead to further enrichment towards more *trans*-isomer of the remaining mixture, if multiple rounds of Ni^(I)^ based isomerization are conducted. To test for the possibility of such an ‘iterative thermodynamic resolution’, we studied a 50:50 *cis*/*trans* mixture of the Weinreb amide **23**, which gave a 91:9 *trans*/*cis* mixture after 1 h Ni^(I)^ catalysis, as shown in Fig. 3a. We found that 81% of the *trans*-product was separated readily by column chromatography and the remaining mixture re-subjected to another round of Ni^(I)^ isomerization. This sequential separation/isomerization was then repeated. After three rounds of this thermodynamic resolution, *trans*-**23** was obtained in 97% yield and more than 99:1 diastereoselectivity (Fig. 3). Given the existing methodological challenges in accessing 1,2-disubstituted cyclopropanes in high e.e. and diastereometric ratio (d.r.), the metalloradical-based iterative thermodynamic enrichment is a powerful downstream manipulation. Beyond 1,2-disubstitution, we further considered the enantiopure trisubstituted **37**, which has the status of an essential pharmacophore as it is not only the key building block of the anti-viral drugs Simeprevir and Danoprevir (against hepatitis C and COVID-19, respectively) but overall featured in a dozen drugs that are either in clinical trial or already approved^41^. Large-scale industrial processes to **37** have been developed^41^. We envisioned that the Ni-based iterative thermodynamic enrichment to the opposite enantiomer could greatly advance the preparation, discovery and large-scale synthesis (based on otherwise existing processes) of new anti-viral drugs. Indeed, three rounds of Ni^(I)^-based enrichment converted 1 g of enantiopure (1*R*,2*S*)-**37** (1:99 d.r., more than 99% e.e.) to the single enantiomer (1*S*,2*S*)-**38** in 99:1 d.r., more than 99% e.e. (in 91% yield). Both separation techniques, that is, crystallization and column chromatography, proved effective in this context.

To further harness the reversibility of the isomerization, its unique capability to retain stereochemical information and its exceptional mildness, we next considered to extend our studies to divinylcyclopropanes. We considered the identified mechanistic features an ideal match to facilitate this strategic transformation, as the reversible nature of the Ni^(I)^-based isomerization should cause any formed *cis*-1,2-divinylcyclopropane to undergo stereospecific Cope-type rearrangement and hence be removed from the equilibrium. Overall, the first counter-thermodynamic *trans*-to-*cis* isomerization of a divinylcyclopropane under retention of enantiopurity should result, followed by Cope-type rearrangement to generate the enantiomerically pure product.

**Fig. 4:**
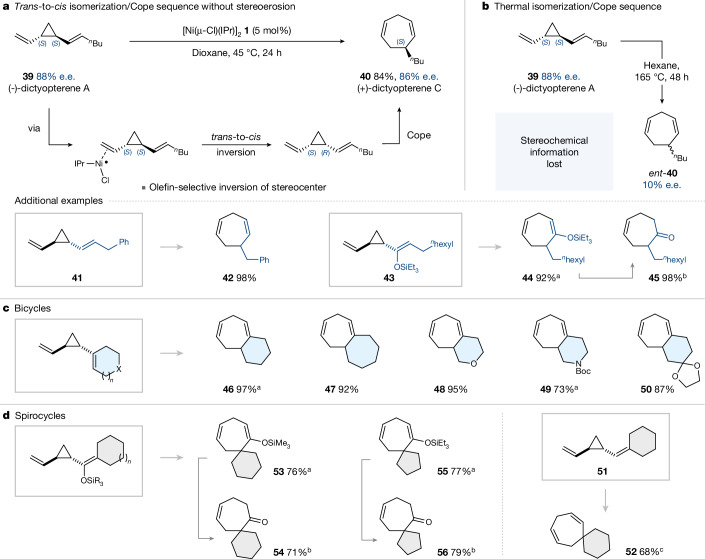
Tandem *trans*-to-*cis* isomerization/Cope rearrangement. **a**, Enantiopure synthesis of (+)-dictyopterene C′. **b**, Loss of stereochemical information in thermal isomerization/Cope sequence. **c**,**d**, Application to fused bicyclic scaffolds (**c**) and spirocycles (**d**). ^a^Yield determined by quantitative ^1^H NMR. ^b^Deprotection conditions: tetra-*n*-butylammonium fluoride (five equivalents), acetic acid (five equivalents), 0 °C, 1 h, then r.t., 2 h. Yield over two steps. ^c^Reaction performed at 80 °C.

As proof-of-principle, we undertook the enantioselective synthesis of (−)-dictyopterene A (**39**, 88% e.e.; Fig. 4a), which is a class of natural products featured in marine algae. We isolated the corresponding seven-membered ring, that is, (+)-dictyopterene C′ (**40**) in 84% yield as the exclusive product in 86% e.e., following the Ni^(I)^ catalysed isomerization-Cope sequence at 45 °C over 24 h. The isomerization proceeded under exclusive inversion of the stereocentre distal from the unsubstituted vinyl substituent, in line with our identified mechanism in Fig. 2 and the higher reactivity that we observed for non-substituted vinylcyclopropanes to associate the Ni^(I)^ (cf. Figs. 3a and 3b). Such a mild and enantiopure isomerization-Cope sequence is, to our knowledge, unprecedented. The established corresponding thermal process that starts with enantiopure *trans*-**39** is known to require heating at 165 °C over 48 h and proceeds under loss of stereochemistry^18^. Indeed, we also obtained the seven-membered ring in modest 10% e.e. (enantiomer-**40**, Fig. 4b).

The phenyl substituted analogue **41** proceeded equally efficiently to **42** under Ni^(I)^ catalysis. Similarly, the silyl enol ether **43** cyclized smoothly to **44**, which eventually yields the ketone **45** upon desilylation. The analogous Ni-free, thermal process has been reported to occur under heating at 230 °C (ref. ^42^) and has found applications in natural product syntheses^6^.

We next considered variants that contain one of the vinyl substituents in a cyclic geometry that would, upon contra-thermodynamic *trans*-to-*cis* isomerization followed by Cope-type rearrangement, result in a fused bicyclic product. Such processes, but thermally induced (more than 130 °C), have been used strategically in synthesis, including of natural products (for example, (+/−)-*beta*-himachalene^43^ or karahanaenone^44^). We observed that, under metalloradical catalysis, the analogous process happened cleanly at 60 °C starting from the *trans*-1-(2-vinylcyclopropyl)cyclohex-1-ene and delivered fused bicycle **46** in 97% yield (Fig. 4c). The same process was equally effective with a larger ring (**47**) or one containing heteroatoms (**48**–**50**).

We next considered the synthesis of spirocycles starting from *trans*-divinylcyclopropanes (Fig. 4d). We obtained spirocycle **52** in 68% under Ni^(I)^-metalloradical catalysis at 80 °C over 24 h. Silyl enol ethers also proceeded smoothly (at 60 °C), and we generated the corresponding spirocyclic ketones (**54**, **56**) after deprotection of the corresponding silyl enol ethers (**53**, **55**). Rearrangements of such silyl enol ethers were previously thermally conducted at 230 °C (refs. ^18,42^).

In summary, this report disclosed the distinct reactivity of a Ni^(I)^-metalloradical, which, in contrast to organic free radicals or many closed-shell metal catalysts, does not lead to ring opening of a (di)vinylcyclopropane under strain release but instead triggers its reversible isomerization under selective inversion of a single (stereo)centre under mild conditions. In light of the pronounced prevalence of odd-oxidation states in non-precious metal species, this study further manifests their potential to enhance the synthetic toolbox and to meet global demands for greater sustainability and lower energy usage in chemical processes.

## Online content

Any methods, additional references, Nature Portfolio reporting summaries, source data, extended data, supplementary information, acknowledgements, peer review information; details of author contributions and competing interests; and statements of data and code availability are available at 10.1038/s41586-024-07555-1.

### Supplementary information

Supplementary Information This file contains Supplementary Sections 1–15 – see Contents page for details.

## Data Availability

The authors declare that the data supporting the findings of this study are available within the paper and its Supplementary Information.
