# Supplementary material for: Dynamic stereomutation of vinylcyclopropanes with metalloradicals
Source: Nature. 2024 Jun 19;631(8019):80–6. doi: 10.1038/s41586-024-07555-1 (PMC11222138; doi:10.1038/s41586-024-07555-1)
Supplement: Supplementary file 1 — This file contains Supplementary Sections 1–15 – see Contents page for details. [file 41586_2024_7555_MOESM1_ESM.pdf]

nature portfolio

https://doi.org/10.1038/s41586-024-07555-1

Supplementary information

Dynamic stereomutation of vinylcyclopropanes with metalloradicals

In the format provided by the authors and unedited

Supporting Information

Dynamic Stereomutation of Vinylcyclopropanes with Metalloradicals

Marvin Mendel, Teresa M. Karl, Jegor Hamm, Sherif J. Kaldas, Theresa Sperger, Bhaskar Mondal and Franziska Schoenebeck*

RWTH Aachen University, Institute of Organic Chemistry, Landoltweg 1, 52074 Aachen, Germany.

Table of Contents

1. General experimental details S5

2. Synthesis of Nickel(I) complexes S7

2.1. Synthesis of IPr ligand S7

2.2. Synthesis of [Ni(μ-Cl)(IPr)]$_2$ dimer (1) S7

2.3. [Ni(Cl)(IPr)(pyridine)] (3) S7

3. Reaction Optimization S8

4. Synthesis of vinylcyclopropane starting materials S12

4.1. General procedures S12

4.1.1. General overview S12

4.1.2. General procedure A: Cyclopropanation with ethyl diazoacetate S13

4.1.3. General Procedure B: Synthesis of vinylcyclopropyl esters S14

4.1.4. General Procedure C: Synthesis of vinylcyclopropyl ketones S14

4.1.5. General procedure D: Synthesis of vinylcyclopropyl amides S15

4.1.6. General Procedure E: Zweifel olefination S15

4.1.7. General Procedure F: Pd(I) catalyzed synthesis of vinyl cyclopropanes[6] S16

4.2. Characterization data S17

5. Isomerization and characterization data of *trans*-vinylcyclopropanes S34

5.1. General Procedure for vinylcyclopropane isomerization S34

5.2. Characterization data of the products S34

6. Scale up of isomerization S46

7. Mechanistic studies S47

7.1. Study on driving force S47

7.2. Isomerization reaction using mononuclear Nickel(I) complex S48

7.3. Reactivity comparison Ni(I) vs. Ni(0) S49

7.3.1. Solvent effect on isomerization S49

7.3.2. Reactivity with internal vinylcyclopropanes S51

7.3.3. Computational study on Ni(0) reactivity S52

7.4. EPR spectroscopic studies S53

7.5. Isomerization without vinyl handle S58

7.5.1. Synthesis and characterization of (*cis*-2-methylcyclopropyl)benzene (27) S58

7.5.2. Isomerization of (*cis*-2-methylcyclopropyl)benzene (27) S58

8. Enantioinvertive vinylcyclopropane isomerization S60

8.1. Synthesis and characterization of enantiopure vinylcyclopropanes S60

8.1.1. General procedure for reduction S61
8.1.2. General procedure for DMP oxidation S63
8.1.3. General procedure for Wittig olefination S63
8.2. Stereoinversive isomerization and downstream derivatization S64
9. Dynamic thermodynamic resolution S65
9.1. Thermodynamic resolution of vinyl cyclopropyl-Weinreb amide (23) S65
9.2. Thermodynamic resolution of vinyl-ACCA ester (37) S66
9.2.1. *trans*-enrichment by flash silica column chromatography S66
9.2.2. *trans*-enrichment by crystallization S66
10. Synthesis of *trans*-divinylcyclopropane starting materials S68
10.1. Synthesis of (1*S*,2*S*)-1-((*E*)-hex-1-en-1-yl)-2-vinylcyclopropane [(-)-dictyopterene A] (39) S68
10.2. General Procedure G: Pd(I) catalyzed synthesis of *trans*-divinylcyclopropanes S69
10.2.1. Characterization data of *trans*-divinylcyclopropanes S70
10.3. Synthesis of ((*trans*-2-vinylcyclopropyl)methylene)cyclohexane (51) S72
10.4. Synthesis of *trans*-vinylcyclopropyl silyl enol ethers S73
10.4.1. Synthesis and characterization data of *trans*-vinylcyclopropylketones S73
10.4.2. General procedure H: synthesis of *trans*-vinylcyclopropyl silyl enol ethers S74
10.4.3. Characterization data of *trans*-vinylcyclopropyl silyl enol ethers S74
11. *Trans* to *cis* isomerization/Cope sequence S76
11.1. General procedure for *trans* to *cis* isomerization/Cope sequence S76
11.2. Characterization data of cyclized products S76
12. Computational Details S82
12.1. Method survey S82
12.1.1. Geometry optimization S82
12.1.2. Energies S83
12.2. Detailed pathway S86
12.2.1. Orbital analyses S86
12.3. Mechanistic alternatives S88
12.3.1. Ni(I)/Ni(III) pathway S88
12.3.2. Other isomers of intermediate Int2 .... S89
12.4. XYZ Coordinates and Energies of Optimized Structures S90
13. Chiral HPLC and GC Analyses S112
13.1. Chiral analysis of enantiopure cyclopropanes S112
13.2. Chiral analysis of *tert*-butyl-1-((*tert*-butoxycarbonyl)amino)-2-vinylcyclopropane-1-carboxylate S117

13.3. Chiral analysis of dictyopterenes S119

14. NMR Spectra S122

14.1. NMR spectra of vinylcyclopropanes and isomerized *trans*-products S122

14.2. NMR spectra of chiral vinylcyclopropane probe and intermediates S200

14.3. NMR spectra of divinylcyclopropanes and cyclized products S202

15. References S229

1. General experimental details

Techniques

All reactions were performed utilizing standard Schlenk techniques unless otherwise stated. The synthesis of Ni-complexes and isomerization reactions have been performed under an Argon atmosphere inside a glovebox. Glassware and magnetic stir bars were dried in an oven (130 °C) for at least 24 hours prior to use and allowed to cool under vacuum at 0.2 mmHg (oil pump). Liquid reagents, solutions or solvents were added via syringe or cannula through rubber septa. Unless otherwise stated, experiments were carried out at room temperature (25 ± 2 °C). The removal of solvents in vacuo was achieved using a rotary evaporator (bath temperatures up to 40 °C) at a pressure of 20 mmHg (diaphragm pump), or at 0.1 mmHg (oil pump) on a vacuum line at room temperature.

Solvents, reagents and starting materials

Unless otherwise stated, all anhydrous solvents were purchased from Sigma Aldrich. Hexane, THF, Et_2O, DCM and toluene were dried using a PS-MD-5 solvent purification system from Innovative Technology. Technical grade solvents were distilled prior to use for chromatography and extraction.

Unless otherwise stated, all reagents and starting materials were commercially available and used as received. *The following reagents were purchased and used as received*: $Ni(COD)_2$ (Sigma Aldrich), (*Z*)-1-bromoprop-1-ene (Sigma Aldrich), 2-Bromopropene (Alfa Aesar), 1-bromo-2-methylprop-1-ene (Sigma Aldrich), α-Bromostyrene (Sigma Aldrich), (*E*)-1-iodooct-1-ene (Sigma Aldrich), 1-bromocyclohex-1-ene (BLDpharm), 5-bromobenzo[*b*]thiophene (BLDpharm), 5-bromobenzofuran (BLDpharm), (4-(bis(trimethylsilyl)amino)phenyl)magnesium chloride (Sigma Aldrich), 4-iodo-2,3-dihydro-1*H*-inden-1-one (Sigma Aldrich), 5-iodo-2-methoxybenzonitrile (Sigma Aldrich), 1-Bromo-1-cycloheptene (ApolloScientific), bromo-3,6-dihydro-2*H*-pyran (Sigma Aldrich), methyl 5-bromofuran-2-carboxylate (BLDpharm), *tert*-butyl 4-(((trifluoromethyl)sulfonyl)oxy)-3,6-dihydropyridine-1(2*H*)-carboxylate (Fluorochem), Ethyl 2,2-dimethyl-3-(2-methylprop-1-en-1-yl)cyclopropane-1-carboxylate (Acros organics), (1*R*,2*S*)-1-((*tert*-butoxycarbonyl)amino)-2-vinylcyclopropane-1-carboxylic acid (BLDpharm).

The following compounds were synthesized according to literature procedures: 1-iodocyclohex-1-ene,[1] (*Z*)-1-iodooct-1-ene,[2] (*E*)-1-bromohex-1-ene,[3] (bromoethynyl)benzene,[4] (*E*)-octa-1,3-diene,[5] 1,3-dioxoisoindolin-2-yl 2-diazoacetate,[5] (*S*)-4,5-dihydro-2,4-diphenyloxazole,[5] [(*S*)-4,5-dihydro-2,4-diphenyloxazole $Ru(CH_3CN)_4$] PF_6 [(*S*)-Ru(L)],[5] (*E*)-(3-bromoallyl)benzene,[6] 1,4-dioxaspiro[4.5]dec-7-en-8-yl trifluoromethanesulfonate,[7] *cis*-1-Iodo-2-vinylcyclopropane,[6] *trans*-1-Iodo-2-vinylcyclopropane.[6]

Purification

Thin layer chromatography (TLC) was performed on Macherey Nagel ALUGRAM Xtra SIL G UV254 aluminium plates with unmodified silica and visualized either under UV light or stained with iodine, $KMnO_4$ or PMA. Flash silica gel column chromatography was performed with silica gel (0.04 – 0.063 mm particle size) purchased from Macherey Nagel. Preparative HPLC was performed on a Knauer Azura HPLC (employing UV detector 2600, at 254 and 230 nm) using a LiChrosorb Si60 column (Merck, 250 x 25 mm, 7 µm silica poriosity). For chiral separation a Chiralpak IA column (Daicel, 250 x 20 mm, 5 µm silica poriosity) was employed.

Characterization

All ^{1}H, ^{13}C and ^{19}F NMR spectra were recorded on Bruker Avance Neo 600, Varian VNMRS 600 or Varian VNMRS 400 or Bruker Avance Neo 400 spectrometers at ambient temperature (unless otherwise specified). Chemical shifts (δ) are reported in parts per million (ppm) and were referenced either to residual solvent peak ($CDCl_3$, CD_2Cl_2, C_6D_6, CD_3CN for ^{1}H and ^{13}C spectra) or internally by the instrument after locking and shimming to the deuterated solvent (for ^{11}B and ^{19}F). Coupling constants (*J*) are given in Hertz (Hz). Multiplicities of signals in ^{1}H, ^{19}F, and ^{13}C NMR were designated as s (singlet), d (doublet), dd (doublet of doublets), dt (doublet of triplets), ddd (doublet of doublets of doublets), t (triplet), td (triplet of doublets), q (quartet), p (quintet), h (sextet), hept (septet), and m (multiplet). For general and accurate prediction of the diastereomeric ratio of the corresponding *cis* and *trans* vinyl cyclopropanes, a relaxation delay of 10 seconds was applied.

Gas chromatography coupled with mass spectrometry (GC-MS) was performed on an Agilent Technologies 5975 series MSD mass spectrometer under electrospray ionization (EI) mode coupled with an Agilent Technologies 7820A gas chromatograph employing an Agilent 19091s-433 HP-5MS column (30 m x 0.250 µm x 0.250 µm). High-resolution mass spectrometry (HRMS) was performed using a Thermo Scientific LTQ Orbitrap XL spectrometer (ESI), Finnigan MAT 95 (EI) or Bruker Maxis II LC-MS-System (APCI). Low-resolution masses of known compounds were extracted from their GC-MS chromatograms.

CW-EPR spectra were recorded on a Freiberg Instruments MS5000 spectrometer with a FC 400 frequency generator and a low temperature Dewar filled with liquid nitrogen for sample cooling. All spectra were recorded with the same microwave power of 50 mW, modulation amplitude of 0.2 mT and sweep time constant of 60 s. EPR simulations were done using EasySpin-5.2.35[8] in MATLAB (version R2021a).

Melting points were measured with a Melting Point Meter MPM-H2 with visual detection and temperature increase of 1 °C/min.

IR spectra were recorded on a Spectrum 100 spectrometer with a UATR Diamond/KRS-5 crystal with attenuated total reflectance (ATR).

Analytical HPLC of chiral compounds was performed on a Hewlett-Packard 1100 Series instrument using chiral stationary phases (details on page S112 ff.). Analytical GC of chiral compounds was performed on an Agilent GC 8890 instrument using chiral stationary phases (details on page S117 ff.).

Optical rotations were measured on a Perkin Elmer 241 Polarimeter at the Sodium D line (589 nm) at 25 °C. Specific rotation $[\alpha]_D^{25}$ was calculated as $[\alpha]_D^{25} = (100 \cdot \alpha)/(l \cdot c)$ with l = 10 cm. Concentration and solvent are provided in g/100 mL.

2. Synthesis of Nickel$^{(I)}$ complexes

2.1. Synthesis of IPr ligand

1,3-Bis-(2,6-di-isopropylphenyl)imidazolium tetrafluoroborate (IPrH·BF_4) was synthesized following the reported procedure by Briggs.[9] The free carbene 1,3-bis(2,6-di-isopropylphenyl)-1,3-dihydro-2*H*-imidazol-2-ylidene (IPr) was prepared according to the protocol developed by Nolan and co-workers.[10]

2.2. Synthesis of [Ni(μ-Cl)(IPr)]$_2$ dimer (1)

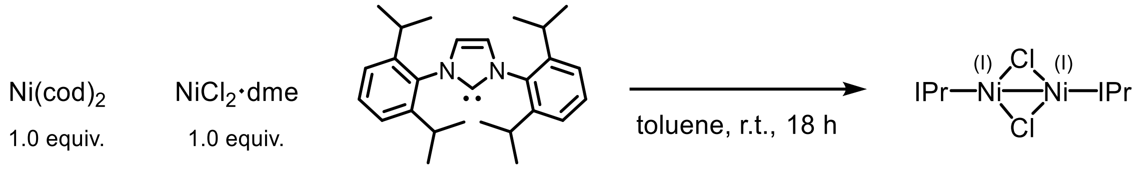

IPr (2.0 equiv.) **1**

The following experimental procedure was carried out inside an Argon-filled glovebox. Air and moisture exclusion must be ensured at all times. Ni(dme)Cl_2 (279 mg, 1.27 mmol, 1.0 equiv.), Ni(cod)$_2$ (349 mg, 1.27 mmol, 1.0 equiv.) and IPr (987 mg, 2.54 mmol, 2.0 equiv.) were suspended in toluene (20 mL). The resulting mixture was stirred at room temperature for exactly 18 h. The obtained red mixture was filtered through a small pad of dry Celite in a fritted funnel and the filtrate concentrated to 8 mL under reduced pressure. Pentane (32 mL) was added and the mixture was placed to the freezer at -30 °C overnight. The formed crystals were collected by filtration and washed with cold pentane (3 x 2 mL) to afford [Ni(μ-Cl)(IPr)]$_2$ as greenish yellow crystals in 66% yield (807mg, 0.84 mmol). The crystals were stored in the freezer (-30 °C). **^{1}H NMR** (400 MHz, C_6D_6): δ 7.08-7.14 (m, 12H), 6.67 (s, 4H), 3.07-3.13 (sept, J = 6.6 Hz, 8H), 2.51 (d, J = 6.6 Hz, 24H), 1.16 (d, J = 6.6 Hz, 24H). Spectroscopic data match with those reported previously in the literature.[11]

Note: If the crystals contained red impurities a recrystallization was performed: The solid was dissolved in a minimum amount of dry toluene and four times the volume of pentane was added. The mixture was then cooled at -30 °C overnight and crystals were collected by filtration and washed with cold pentane (3 x 2 mL).

2.3. [Ni(Cl)(IPr)(pyridine)] (3)

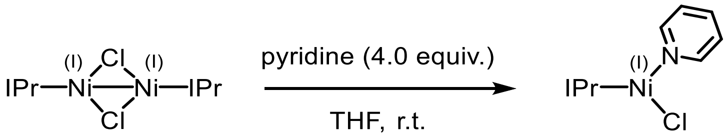

3

Synthesized following the procedure of Matsubara and co-workers.[12] Inside an argon filled glovebox, [Ni(μ-Cl)(IPr)]$_2$ (30 mg, 0.03 mmol, 1.0 equiv.) and THF (0.5 mL) were added to a 4 mL screw-capped vial. Then, pyridine (9.6 μL, 0.12 mmol, 4.0 equiv.) was added and the vial was shaken until the compound had dissolved and a clear red solution occurred. Next, pentane (2.5 mL) was slowly added and the solution was placed to the freezer at -30°C for crystallization overnight. Red crystals of **3** were obtained after removal of the liquid and washing with a small amount of pentane and dried under vacuum (11 mg, 0.02 mmol, 65%). **^{1}HNMR** (400 MHz, C_6D_6) δ 10.7 (bs), 6.95 (bs), 6.37 (bs), 3.08 (bs), 2.48 (bs), 1.75 (bs), 1.18 (bs). The data are in agreement with those previously reported in the literature.[12]

3. Reaction Optimization

All reactions were carried out inside an argon-filled glovebox. A 4 mL screw-cap vial equipped with a Teflon-coated magnetic stir bar was sequentially charged with the corresponding cyclopropane substrate (1.0 equiv.), anhydrous 1,4-dioxane (0.4 M) and [Ni(μ-Cl)(IPr)]$_2$, in that order. The reaction vial was then sealed and allowed to stir inside the glovebox at the indicated temperature. After the indicated time the reaction mixture was removed from the glovebox and was quenched by the addition of wet pentane. A spatula tip of ammonium pyrrolidine-1-dithiocarboxylic acid was added and the mixture stirred for additional 15 min to precipitate nickel.[13] The resulting mixture was filtered through a plug of silica, washing with Et_2O or EtOAc or MeCN and the filtrate was concentrated under reduced pressure. The crude material was analyzed by quantitative ^{1}H NMR (10 s relaxation delay).

Table S1. Reaction optimization of vinylcyclopropyl arenes.

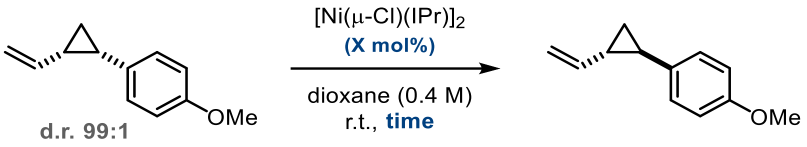

entry	catalyst loading	time	d.r. (*cis*:*trans*)
**1**	5 mol%	15 min	10:90
**2**	2.5 mol%	15 min	10:90
**3**	1 mol%	15 min	10:90
**4**	5 mol%	5 min	10:90
**5**	**1 mol%**	**5 min**	**10:90 (98%)[c]**
**6[a]**	1 mol%	5 min	16:84
**7[a]**	1 mol%	2 min	22:78
**8**	1 mol%	2 min	16:84
**9**	5 mol%	24 h	10:90
**10[b]**	5 mol%	24 h	12:88

a) THF (0.4 M) was used as solvent; b) 60 °C reaction temperature; c) isolated yield.

Table S2. Reaction optimization of vinylcyclopropyl esters.

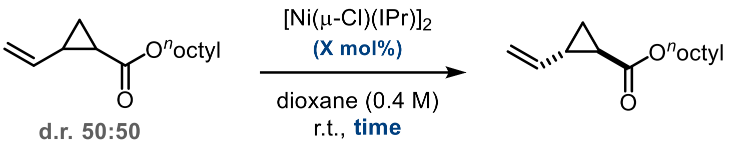

entry	catalyst loading	time	d.r. (*cis*:*trans*)
**1**	5 mol%	15 min	80:20
**2**	5 mol%	30 min	16:84
**3**	**5 mol%**	**60 min**	**9:91 (94%)[a]**
**4**	5 mol%	24 h	9:91
**5[b]**	**5 mol%**	**15 min**	**9:91 (96%)[a]**

a) isolated yield; b) vinylcyclopropyl ketone was applied as starting material.

Table S3. Reaction optimization of vinylcyclopropyl amides.

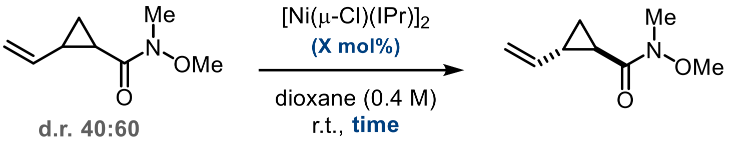

entry	catalyst loading	time	d.r. (*cis*:*trans*)
1	5 mol%	15 min	17:83
2	5 mol%	30 min	16:84
**3**	**5 mol%**	**60 min**	**9:91 (97%)[a]**
4	5 mol%	24 h	9:91

a) isolated yield.

Table S4. Reaction optimization of vinylcyclopropyl carboxylic acid.

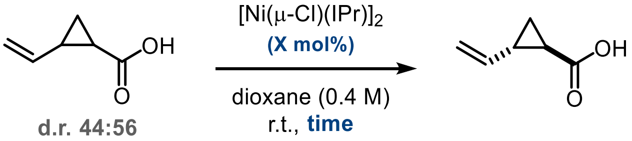

entry	catalyst loading	time	d.r. (*cis*:*trans*)
1	1 mol%	5 min	35:65
2	5 mol%	5 min	31:69
3	1 mol%	15 min	35:65
**4**	**5 mol%**	**15 min**	**25:75 (94%)[a]**
5	5 mol%	36 h	25:75 (80%)[a]

a) isolated yield.

Table S5. Reaction optimization of vinylcyclopropyl alkyl.

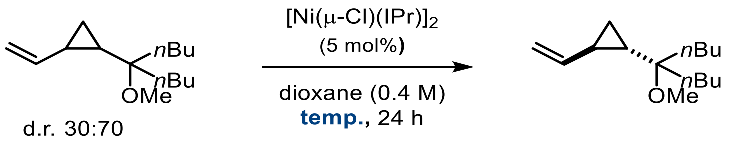

entry	catalyst loading	temperature	d.r. (*cis*:*trans*)
1	5 mol%	45 °C	15:85
**2**	**5 mol%**	**60 °C**	**1:99 (96%)[a]**

a) isolated yield.

Table S6. Reaction optimization of vinylcyclopropyl alkylgermane and -boronates.

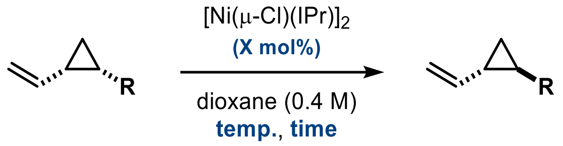

entry	R (d.r. *cis*:*trans*)	catalyst loading	temperature	time	d.r. (*cis*:*trans*)
1	$GeEt_3$ (100:0)	5 mol%	r.t.	15 min	100:0
2	$GeEt_3$ (100:0)	5 mol%	r.t.	2 h	90:10
3	$GeEt_3$ (100:0)	5 mol%	r.t.	14 h	54:46
**4**	**$GeEt_3$ (100:0)**	**5 mol%**	**r.t.**	**24 h**	**10:90 (96%)[a]**
5	BMIDA (35:65)	5 mol%	r.t.	24 h	35:65
6	BMIDA (35:65)	5 mol%	60 °C	24 h	22:78
**7**	**BMIDA (35:65)**	**5 mol%**	**60 °C**	**72 h**	**12:88 (95%)[a]**
8	BPin (41:59)	5 mol%	60 °C	72 h	21:79 (82%)[a]
**9[b]**	**BPin (41:59)**	**5 mol%**	**60 °C**	**72 h**	**22:78 (88%)[a]**

a) isolated yield; b) addition of 10 mol% pyridine.

Table S7. Reaction optimization of *Z*-alkenylcyclopropyl benzene.

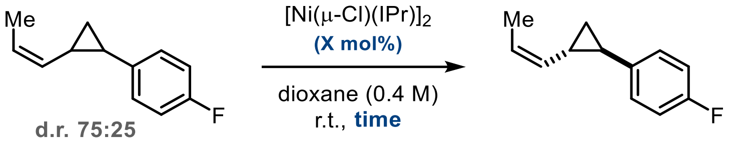

entry	catalyst loading	time	d.r. (*cis*:*trans*)
1	1 mol%	5 min	70:30
2	1 mol%	2 h	40:60
3	5 mol%	1 h	22:78
**4**	**5 mol%**	**2 h**	**11:89 (96%)[a]**
5	5 mol%	24 h	11:89 (88%)[a]

a) isolated yield.

Table S8. Reaction optimization of higher substituted alkenylcyclopropyl benzene.

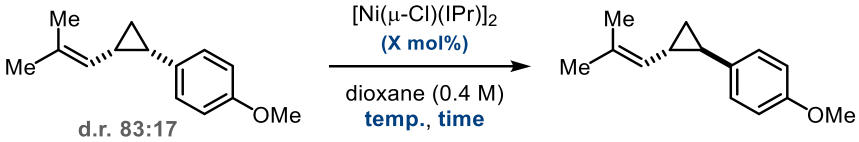

entry	catalyst loading	temperature	time	d.r. (*cis*:*trans*)
1	5 mol%	r.t.	2 h	83:17
2	5 mol%	r.t.	24 h	79:21
3	5 mol%	r.t.	48 h	76:24
4	5 mol%	60 °C	72 h	28:72
**5**	**10 mol%**	**60 °C**	**72 h**	**18:82 (91%)[a]**

a) isolated yield.

Table S9. Reaction optimization of vinyl-ACCA ester isomerization.

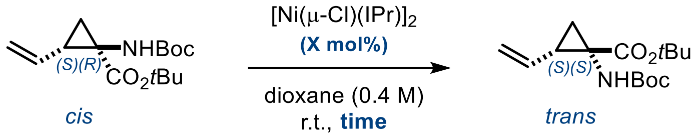

entry	catalyst loading	time	d.r. (*cis*:*trans*)	Yield[b]
**1**[a]	5 mol%	24 h	25:75	n.d.
**2**	5 mol%	24 h	20:80	n.d.
**3**	2 mol%	2 h	16:84	>99%
**4**	2 mol%	1 h	16:84	>99%
**5**	2 mol%	30 min	14:86	>99%
**6**	2 mol%	15 min	15:85	>99%
**7**	1 mol%	15 min	14:86	n.d.
**8**	**1 mol%**	**10 min**	**14:86**	**>99% (96%)**[c]
**9**	1 mol%	5 min	20:80	n.d.

a) 60 °C reaction temperature; b) qNMR yield using ethylenecarbonate as internal standard; c) isolated yield.

4. Synthesis of vinylcyclopropane starting materials

4.1. General procedures

4.1.1. General overview

General Procedure A: Cyclopropanation with ethyl diazoacetate

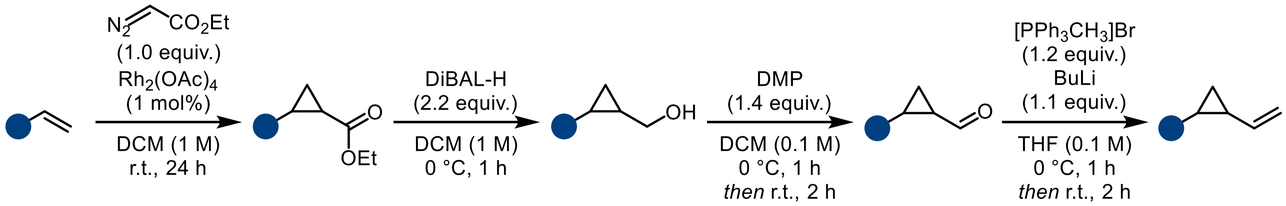

General Procedure B: Synthesis of vinylcyclopropyl ester

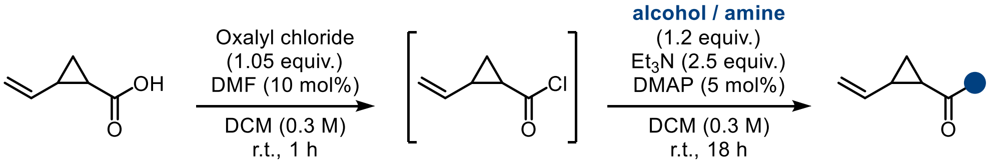

General Procedure C: Synthesis of vinylcyclopropyl ketones

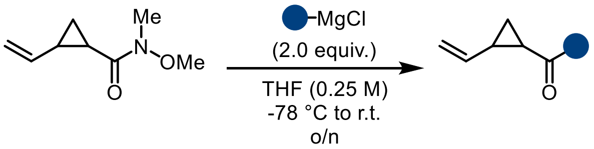

General Procedure D: Synthesis of vinylcyclopropyl amides

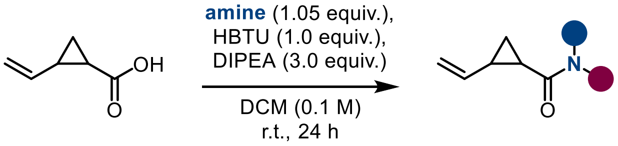

General Procedure E: Synthesis of vinylcyclopropyl boronates and Zweifel olefination

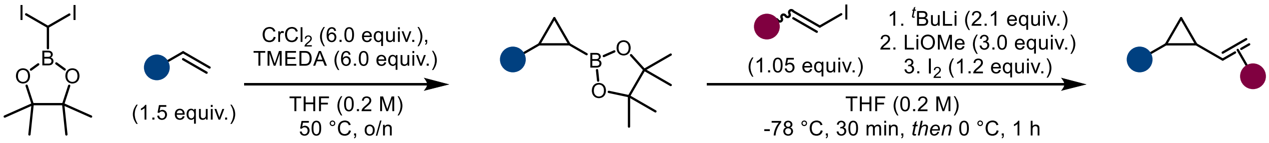

General Procedure F: Pd(I) mediated cross coupling of cyclopropyl zincates

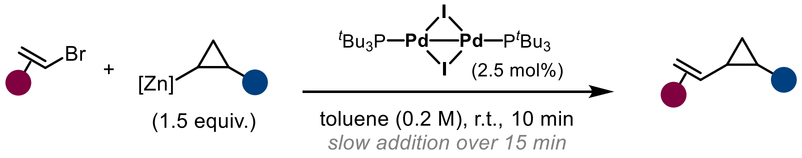

4.1.2. General procedure A: Cyclopropanation with ethyl diazoacetate

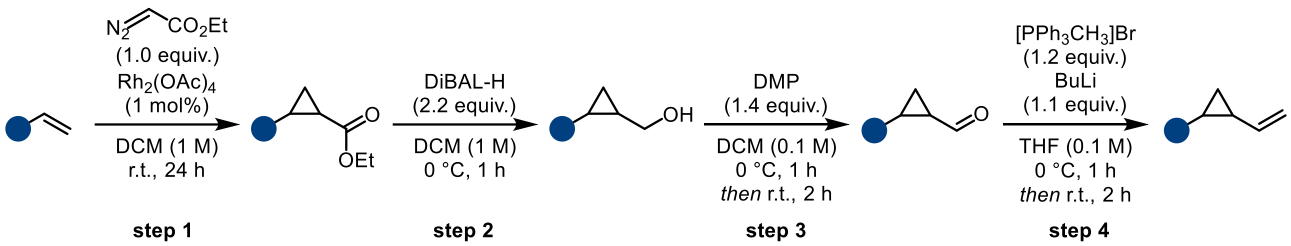

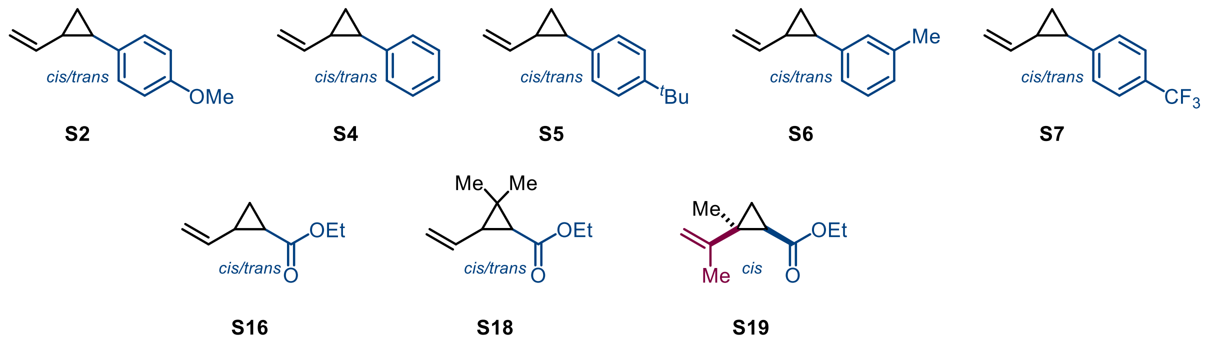

Step 1: An oven dried flask equipped with a magnetic stir bar was charged with the corresponding styrene (1.0 equiv.), $Rh_2(OAc)_4$ (1 mol%) and dry DCM (1 M). Next, a solution of diazo ethyl diazoacetate (1.0 equiv.) in DCM (2 M) was added via syringe pump and over the course of 1 hour. After complete addition the resulting green mixture was stirred for further 24 hours before it was filtered through a short pad of silica gel. The mixture was concentrated under reduced pressure and flash silica gel column chromatography afforded corresponding product as a diastereomeric mixture.

Step 2: Diisobutylaluminium hydride (1.0 M in toluene, 2.2 equiv.) was added dropwise over the course of 15 min to a stirred solution of the respective cyclopropyl ester (1.0 equiv.) in DCM (1 M) at −78 °C. The mixture was stirred for 1 h at 0 °C and HCl (1 M, 30 mL) was then slowly added. The aqueous layer was extracted with DCM (2 x 20 mL) and the combined organic extracts were dried over Na_2SO_4, filtered, and concentrated under reduced pressure. Flash silica gel column chromatography afforded the corresponding cyclopropyl alcohol as a diastereomeric mixture.

Step 3: The corresponding alcohol (1 mmol, 1.0 equiv.) was weighed into an oven dried flask and dissolved in dry DCM (10 mL, 0.1 M). The solution was cooled to 0 °C before DMP (600 mg, 1.4 mmol, 1.4 equiv.) was added and the reaction mixture was stirred for 1 h at 0 °C and then for 2 h at room temperature. The mixture was quenched by adding 10 mL of an aqueous 1:1 mixture of sat. $NaHCO_3$ and sat. $Na_2S_2O_3$ and it was stirred until both phases were clear. Next, the phases were separated, and the aqueous phase was extracted with DCM (3 x 10 mL). The combined organic layers were dried over Na_2SO_4, filtered, and concentrated under reduced pressure. The crude aldehydes were obtained quantitative in high purity and were used directly for the next step without further purification.

Step 4: An oven dried flask was charged with methyltriphenylphosphonium bromide (1.2 mmol, 1.2 equiv.) before it was evacuated and backfilled with argon for three times. It was suspended in dry THF (10 mL, 0.1 M), cooled to 0 °C and ^{n}BuLi (1.1 mmol, 1.1 equiv., 2.5 M in hexane) was added dropwise

and the reaction mixture stirred for 30 min at 0 °C. Then, a solution of the corresponding aldehyde (1.0 mmol, 1.0 equiv., 0.5 M in THF) was added and the resulting mixture was stirred for 1 h at 0 °C followed by 2 h at room temperature. The mixture was quenched by adding 5 mL of sat. aqueous NH_4Cl and phases were separated. The aqueous phase was extracted with DCM (2 x 10 mL). The combined organic layers were dried over Na_2SO_4, filtered, and concentrated under reduced pressure. The crude vinylcyclopropane was purified by silica silica gel column chromatography.

4.1.3. General Procedure B: Synthesis of vinylcyclopropyl esters

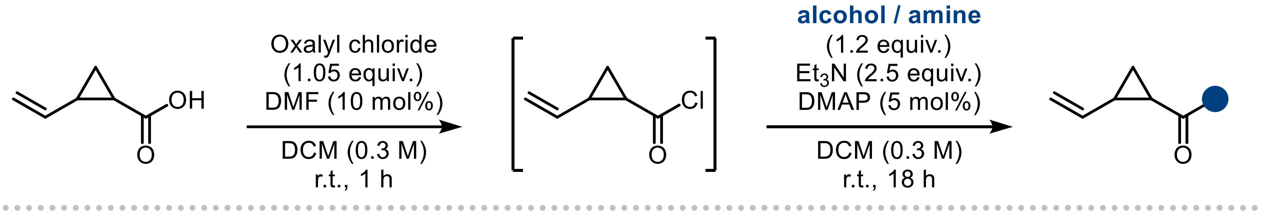

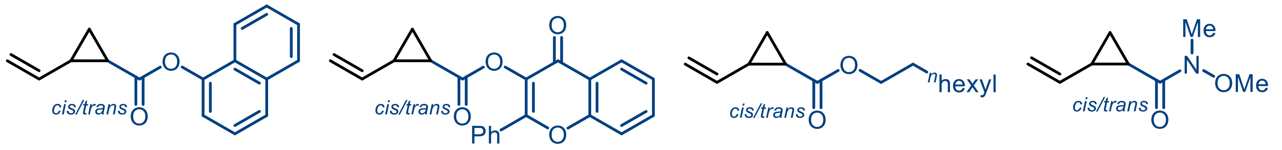

S13 **S14** **S15** **S23**

2-Vinylcyclopropane-1-carboxylic acid (1.0 equiv.) was dissolved in anhydrous DCM (0.3 M) and DMF (10 mol%) was added. The mixture was cooled to 0 °C and stirred for 5 minutes before a solution of oxalyl chloride (1.05 equiv.) in anhydrous DCM (2 M) was added dropwise to the reaction mixture. The reaction was then stirred at room temperature for 1 hour (until no gas evolution was observed). The freshly prepared acid chloride was then added to a stirred solution of the corresponding alcohol or amine (1.2 equiv.), Et_3N (2.5 equiv.) and DMAP (5 mol%) in anhydrous DCM (0.3 M) at 0 °C. After stirring for 5 minutes the reaction mixture was allowed to warm up to room temperature and stirred overnight. The mixture was quenched by adding 10 mL of sat. aqueous NH_4Cl and the phases were separated. The aqueous phase was extracted with DCM (2 x 10 mL). The combined organic layers were dried over Na_2SO_4, filtered, and concentrated under reduced pressure. The crude vinylcyclopropane was purified by silica gel column chromatography.

4.1.4. General Procedure C: Synthesis of vinylcyclopropyl ketones

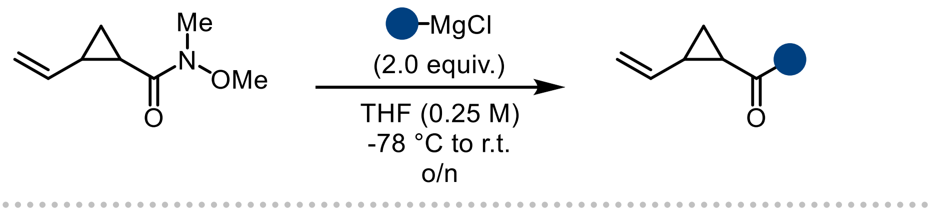

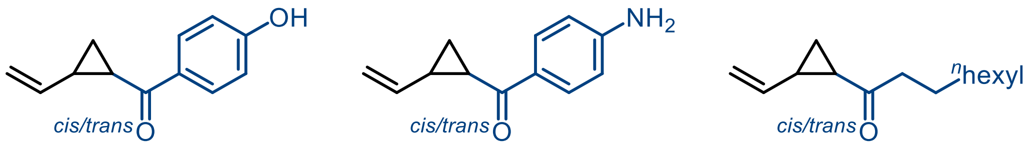

S10 **S11** **S12**

N-methoxy-*N*-methyl-2-vinylcyclopropane-1-carboxamide (1.0 mmol, 1.0 equiv.) was dissolved in anhydrous THF (0.25 M). The mixture was cooled to -78 °C before a solution of the corresponding organomagnesium compound (2.0 equiv.) in anhydrous THF or Et_2O (0.5 - 2 M) was added dropwise to the reaction mixture. After stirring for 5 minutes the reaction mixture was allowed to warm up to room temperature and stirred over night. The mixture was quenched by adding 10 mL of sat. aqueous NH_4Cl and phases were separated. The aqueous phase was extracted with DCM (2 x 10 mL). The combined organic layers were dried over Na_2SO_4, filtered, and concentrated under reduced pressure. The crude vinyl cyclopropane was purified by silica gel column chromatography.

4.1.5. General procedure D: Synthesis of vinylcyclopropyl amides

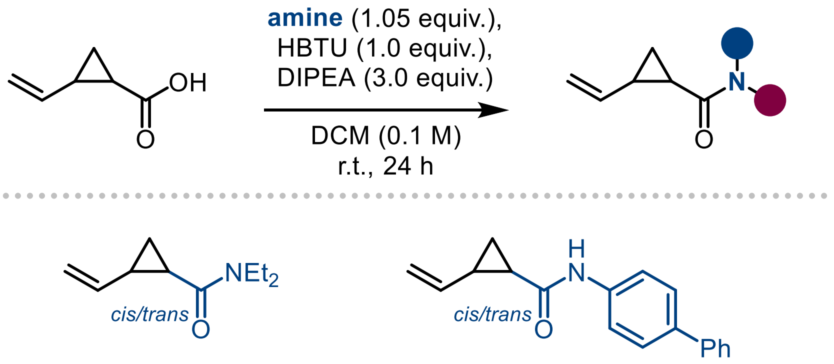

S21 **S22**

2-Vinylcyclopropane-1-carboxylic acid (1.0 equiv.), HBTU (1.05 equiv.) and DIPEA (3.0 equiv.) were dissolved in anhydrous DCM (0.1 M) and stirred for 5 minutes before the amine (1.05 equiv.) was added. The reaction was then stirred at room temperature overnight. The reaction mixture was then diluted with DCM (10 mL) and washed with saturated $NaHCO_3$ solution (10 mL), 2 M HCl solution (10 mL) and brine (10 mL). The organic phase was dried over $MgSO_4$ and concentrated *in vacuo* before purification by silica gel silica gel column chromatography.

4.1.6. General Procedure E: Zweifel olefination

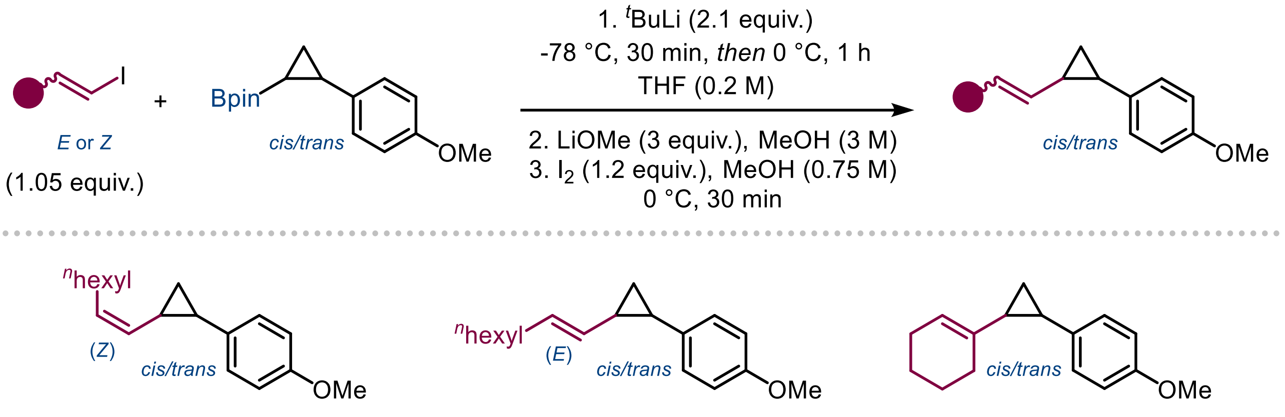

S31 **S32** **S33**

The corresponding vinyl iodide (0.525 mmol, 1.05 equiv.) was added to an oven dried Schlenk flask and dissolved in dry THF (2.5 mL, 0.2 M). The solution was then cooled to -78 °C and ^{t}BuLi (0.62 mL, 1.05 mmol, 2.1 equiv, 1.7 M in pentane) was added dropwise over a period of 10 min. After complete addition

the mixture was stirred for 30 min at this temperature before a solution of 2-(2-(4-methoxyphenyl)cyclopropyl)-4,4,5,5-tetramethyl-1,3,2-dioxaborolane (137 mg, 0.5 mmol, 1.0 equiv.) in THF (2 mL, 0.25 M) was added dropwise. The reaction mixture was stirred for additional 15 min at -78 °C then warmed up to 0 °C and stirred for 1 h. Next, a suspension of LiOMe (57 mg, 1.5 mmol, 3.0 equiv.) in MeOH (0.5 mL, 3 M) was added and the mixture was cooled to 0 °C before a solution of iodine (153 mg, 0.6 mmol, 1.2 equiv.) in MeOH (0.8 mL, 0.75 M) was added dropwise at this temperature. The reaction was stirred for 30 min before it was quenched with sat. aqueous Na_2SO_3 solution (10 mL). Phases were separated and the aqueous phase was extracted with DCM (3x 10 mL), dried over Na_2SO_4, filtered, and concentrated under reduced pressure. The crude material was then purified by silica gel column chromatography.

4.1.7. General Procedure F: Pd(I) catalyzed synthesis of vinyl cyclopropanes[6]

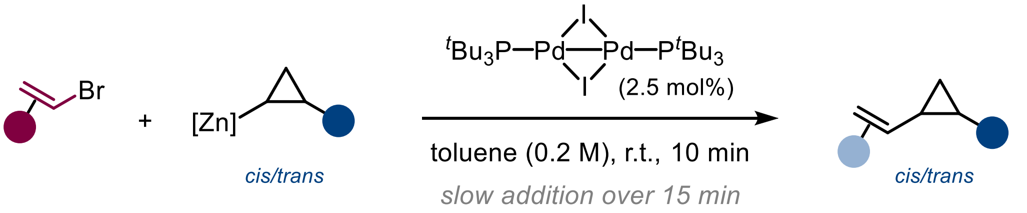

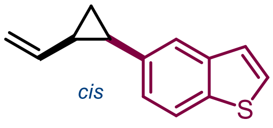

S8

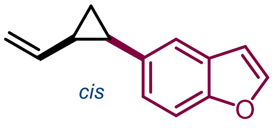

S9

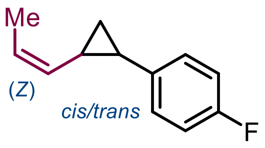

S28

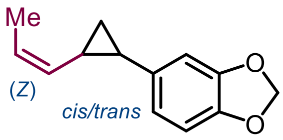

S29

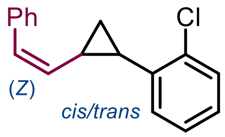

S30

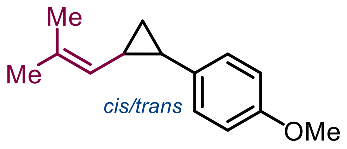

S34

Synthesized according to literature procedure.[6] In an argon filled glovebox, vinyl/alkynyl halide (1.0 equiv.) was placed into an oven dried 20 mL screw cap vial (or an oven dried round bottom flask in case of scale up) equipped with a magnetic stir bar. It was dissolved in dry toluene (0.2 M) and [Pd(μ-I)(P(t-Bu)$_3$)]$_2$-dimer (2.5 mol%) was added. The vial was sealed with a rubber septum, brought outside and connected to a Schlenk line. Next, a solution of cyclopropyl organozincate (1.2–2.0 equiv.) was either added slowly to the reaction mixture via syringe pump (over 15 min) or the obtained toluene solution was added slowly to the cyclopropyl organozincate solution via syringe pump (over 15 min). The reaction mixture was stirred for additional 10 min, before it was quenched by the addition of wet pentane or hexane. A spatula tip of ammonium pyrrolidine-1-dithiocarboxylic acid was added and the mixture was stirred for additional 15 min to precipitate palladium.[13] The mixture was filtered through a plug of silica, washing with Et_2O and the filtrate was concentrated under reduced pressure. The crude material was further purified by silica gel column chromatography.

4.2. Characterization data

1-methoxy-4-(*cis*-2-vinylcyclopropyl)benzene (S2)

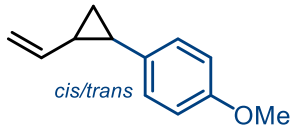

Prepared following General Procedure A from *cis*-2-(4-methoxyphenyl)cyclopropane-1-carbaldehyde (159 mg, 0.9 mmol). The title product was obtained after purification by silica gel column chromatography (50:1 pentane:Et_2O) as a colorless oil (110 mg, 0.63 mmol, 70%). **R_f** = 0.38 (50:1 hexane:Et_2O). **^{1}H NMR** (400 MHz, $CDCl_3$) δ 7.17 – 7.10 (m, 2H), 6.86 – 6.79 (m, 2H), 5.14 – 5.06 (m, 2H), 4.89 – 4.81 (m, 1H), 3.79 (s, 3H), 2.29 (td, *J* = 8.5, 6.4 Hz, 1H), 1.88 – 1.75 (m, 1H), 1.23 (td, *J* = 8.4, 5.1 Hz, 1H), 0.96 (q, *J* = 5.5 Hz, 1H). **^{13}C NMR** (101 MHz, $CDCl_3$) δ 158.1, 138.6, 130.9, 130.3, 113.9, 113.6, 55.4, 22.8, 22.6, 12.0. **IR** (neat, cm^{-1}): 3074, 3001, 1633, 1611, 1512, 1458, 1295, 1244, 1177, 1034, 985, 894, 830, 798. **HRMS** (ESI): *m/z* $[M+H]^+$ calculated for $C_{12}H_{15}O$: 175.1117, found 175.1114.

(2-vinylcyclopropyl)benzene (S4)

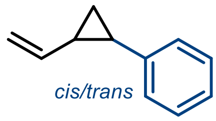

Prepared following General Procedure A from 2-phenylcyclopropane-1-carbaldehyde (142 mg, 1 mmol). The title product was obtained after purification by silica gel column chromatography (pentane) as a colorless oil (105 mg, 0.728 mmol, 73%, 40:60 *cis/trans* (^{1}H NMR)). **R_f** = 0.4 (hexane). **^{1}H NMR** (600 MHz, $CDCl_3$) δ 7.31 – 7.25 (m, 2H *cis* + 2H *trans*), 7.24 – 7.15 (m, 3H *cis* + 1H *trans*), 7.09 (d, *J* = 7.6 Hz, 2H *trans*), 5.56 (ddd, *J* = 17.3, 10.2, 8.5 Hz, 1H, *trans*), 5.17 – 5.08 (m, 2H *cis* + 1H *trans*), 4.95 (dd, *J* = 10.3, 1.4 Hz, 1H, *trans*), 4.89 – 4.85 (m, 1H, *cis*), 2.37 (td, *J* = 8.6, 6.4 Hz, 1H, *cis*), 1.94 (dt, *J* = 9.4, 5.1 Hz, 1H, *trans*), 1.92 – 1.85 (m, 1H, *cis*), 1.72 (tt, *J* = 8.8, 5.0 Hz, 1H, *trans*), 1.28 (td, *J* = 8.4, 5.2 Hz, 1H, *cis*), 1.22 (dt, *J* = 8.5, 5.4 Hz, 1H, *trans*), 1.12 (dt, *J* = 8.9, 5.3 Hz, 1H, *trans*), 1.06 (q, *J* = 5.7 Hz, 1H, *cis*). **^{13}C NMR** (151 MHz, $CDCl_3$) δ 142.5, 140.8, 138.9, 138.2, 129.3, 128.5, 128.2, 126.1, 125.8, 125.8, 114.2, 112.7, 27.5, 25.4, 23.4, 23.1, 16.9, 11.8. **IR** (neat, cm^{-1}): 3075, 3025, 1635, 1603, 1496, 1453, 1077, 1030, 985, 895, 838, 747, 696. **HRMS** (EI): *m/z* $[M]^+$ calculated for $C_{11}H_{12}$: 144.0934, found 144.0937.

1-(*tert*-butyl)-4-(2-vinylcyclopropyl)benzene (S5)

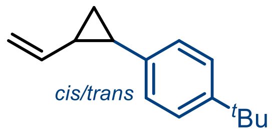

Prepared following General Procedure A from 2-(4-(*tert*-butyl)phenyl)cyclopropane-1-carbaldehyde (101 mg, 0.5 mmol). The title product was obtained after purification by silica gel column chromatography (pentane) as a colorless oil (99 mg, 0.49 mmol, 98%, 27:73 *cis/trans* (^{1}H NMR)). **R_f** = 0.34 (hexane). **^{1}H NMR** (600 MHz, $CDCl_3$) δ 7.31 (dd, *J* = 8.4, 2.6 Hz, 2H *cis* + 2H *trans*), 7.15 (d, *J* = 7.8 Hz, 2H, *cis*), 7.05 – 7.01 (m, 2H, *trans*), 5.54 (ddd, *J* = 17.5, 10.2, 8.4 Hz, 1H, *trans*), 5.21 – 5.12 (m, 2H, *cis*), 5.10 (dd, *J* = 17.1, 1.6 Hz, 1H, *trans*), 4.93 (dd, *J* = 10.3, 1.6 Hz, 1H, *trans*), 4.87 (dd, *J* = 8.6, 3.7 Hz, 1H, *cis*), 2.32 (q, *J* = 8.4 Hz, 1H, *cis*), 1.91 (dt, *J* = 9.3, 5.0 Hz, 1H, *trans*), 1.86 (qd, *J* = 8.1, 5.3 Hz, 1H, *cis*), 1.70 (tt, *J* = 8.9, 5.0 Hz, 1H, *trans*), 1.34 – 1.30 (m, 9H *cis* + 9H *trans*), 1.28 – 1.23 (m, 1H, *cis*), 1.19 (dt, *J* = 8.7, 5.5 Hz, 1H, *trans*), 1.10 (dt, *J* = 9.3, 5.4 Hz, 1H, *trans*), 1.01 (q, *J* = 5.7 Hz, 1H, *cis*). **^{13}C NMR** (151 MHz, $CDCl_3$) δ 148.8, 148.7, 140.9, 139.4, 138.6, 135.8, 128.9, 125.5, 125.4, 125.1, 114.0, 112.5, 34.5, 34.5, 31.5, 31.5, 27.4, 25.0, 23.1, 23.0, 16.8, 12.0. **IR** (neat, cm^{-1}): 3077, 2960, 1635, 1516, 1463, 1363, 1268, 1197, 1118, 1024, 984, 894, 830, 688. **HRMS** (EI): *m/z* $[M]^+$ calculated for $C_{15}H_{20}$: 200.1560, found 200.1554.

1-methyl-3-(2-vinylcyclopropyl)benzene (S6)

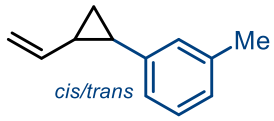

Prepared following General Procedure A from 2-(m-tolyl)cyclopropane-1-carbaldehyde (161 mg, 1.0 mmol). The title product was obtained after purification by silica gel column chromatography (pentane) as a colorless oil (126 mg, 0.796 mmol, 80%, 34:66 *cis/trans* (^{1}H NMR)). **R$_f$** = 0.43 (hexane). **^{1}H NMR** (600 MHz, $CDCl_3$) δ 7.19 – 7.14 (m, 1H *cis*, 1H *trans*), 7.06 – 6.99 (m, 3H, *cis*), 6.98 (ddt, *J* = 7.6, 1.9, 0.8 Hz, 1H, *trans*), 6.89 (dt, *J* = 9.5, 1.8 Hz, 2H, *trans*), 5.54 (ddd, *J* = 17.0, 10.3, 8.5 Hz, 1H, *trans*), 5.15 – 5.09 (m, 2H *cis*, 1H *trans*), 4.94 (dd, *J* = 10.3, 1.6 Hz, 1H, *trans*), 4.86 (dd, *J* = 7.3, 5.0 Hz, 1H, *cis*), 2.34 (s, 3H, *cis*), 2.34 – 2.32 (m, 3H *trans*, 1H *cis*), 1.90 (ddd, *J* = 8.7, 5.8, 4.3 Hz, 1H, *trans*), 1.88 – 1.84 (m, 1H, *cis*), 1.70 (tdd, *J* = 8.6, 5.6, 4.3 Hz, 1H, *trans*), 1.25 (td, *J* = 8.4, 5.2 Hz, 1H, *cis*), 1.20 (ddd, *J* = 8.5, 5.8, 4.9 Hz, 1H, *trans*), 1.10 (ddd, *J* = 8.7, 5.7, 5.0 Hz, 1H, *trans*), 1.04 (dt, *J* = 6.4, 5.4 Hz, 1H, *cis*). **^{13}C NMR** (151 MHz, $CDCl_3$) δ 142.4, 140.9, 138.7, 138.4, 138.1, 137.7, 130.1, 128.4, 128.0, 126.9, 126.7, 126.6, 126.2, 122.8, 114.1, 112.6, 27.5, 25.3, 23.4, 23.1, 21.6, 21.6, 16.8, 11.8. **HRMS** (EI): *m/z* $[M]^+$ calculated for $C_{12}H_{14}$: 158.1090, found 158.1090.

1-(trifluoromethyl)-4-(2-vinylcyclopropyl)benzene (S7)

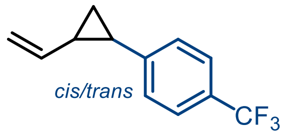

Prepared following General Procedure A from 2-(4-(trifluoromethyl)phenyl)cyclopropane-1-carbaldehyde (215 mg, 1.0 mmol). The title product was obtained after purification by silica gel column chromatography (pentane) as a colorless oil (150 mg, 0.706 mmol, 71%, 36:64 *cis/trans* (^{1}H NMR)). **R$_f$** = 0.52 (hexane). **^{1}H NMR** (600 MHz, $CDCl_3$) δ 7.55 – 7.49 (m, 2H *cis* + 2H *trans*), 7.31 (d, *J* = 8.0 Hz, 2H, *cis*), 7.16 (d, *J* = 8.0 Hz, 2H, *trans*), 5.55 (dddd, *J* = 17.0, 9.8, 8.3, 1.1 Hz, 1H, *trans*), 5.18 – 5.11 (m, 1H *cis* + 1H *trans*), 5.11 – 5.04 (m, 1H, *cis*), 5.00 – 4.96 (m, 1H, *trans*), 4.90 (dt, *J* = 10.1, 1.6 Hz, 1H, *cis*), 2.38 (q, *J* = 8.1 Hz, 1H, *cis*), 2.00 – 1.91 (m, 1H *cis* + 1H *trans*), 1.74 (tt, *J* = 8.9, 5.0 Hz, 1H, *trans*), 1.36 – 1.30 (m, 1H, *cis*), 1.27 – 1.22 (m, 1H, *trans*), 1.22 – 1.17 (m, 1H, *trans*), 1.09 (q, *J* = 5.9 Hz, 1H, *cis*). **^{19}F NMR** (565 MHz, $CDCl_3$) δ -62.29 (s, 3F, *trans*), -62.32 (s, 3F, *cis*). **^{13}C NMR** (151 MHz, $CDCl_3$) δ 146.8, 143.2, 140.0, 137.3, 129.5, 128.3 (q, *J* = 32.3 Hz), 128.0 (q, *J* = 32.3 Hz), 126.0, 125.4 (q, *J* = 3.7 Hz), 125.1 (q, *J* = 3.7 Hz), 123.6, 123.6, 115.2, 113.4, 28.2, 25.2, 23.4, 23.3, 17.4, 12.1. **HRMS** (EI): *m/z* $[M]^+$ calculated for $C_{12}H_{11}F_3$: 212.0807, found 212.0800.

5-(*cis*-2-vinylcyclopropyl)benzo[*b*]thiophene (S8)

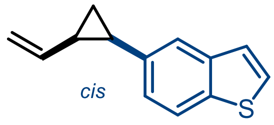

Synthesized following the General Procedure F using 5-bromobenzo[*b*]thiophene (213 mg, 1 mmol, 1.0 equiv.) and a suspension of freshly prepared (*cis*-2-vinylcyclopropyl)zinc(II) chloride[6] (4.9 mL, 2.0 equiv.). Flash column chromatography (100:1 pentane:Et_2O, PMA) afforded the title product as a colorless oil (77 mg, 0.38 mmol, 38%). **R$_f$** = 0.42 (100:1 pentane:Et_2O). **^{1}H NMR** (600 MHz, $CDCl_3$) δ 7.78 (d, *J* = 8.3 Hz, 1H), 7.66 (s, 1H), 7.42 (d, *J* = 5.4 Hz, 1H), 7.29 (dd, *J* = 5.4, 0.8 Hz, 1H), 7.24 (dd, *J* = 8.3, 1.7 Hz, 1H), 5.18 – 5.06 (m, 2H), 4.85 – 4.83 (m, 1H), 2.48 (td, *J* = 8.6, 6.4 Hz, 1H), 1.92 (qd, *J* = 8.6, 5.4 Hz, 1H), 1.32 (td, *J* = 8.4, 5.2 Hz, 1H), 1.12 (dt, *J* = 6.4, 5.4 Hz, 1H). **^{13}C NMR** (151 MHz, $CDCl_3$) δ 139.9, 138.4, 137.7, 135.1, 126.6, 126.4, 123.8 (2C), 122.1, 114.1, 23.4, 23.1, 12.1. **HRMS** (ESI): *m/z* $[M]^+$ calculated for $C_{13}H_{12}S$: 200.0654, found 200.0656.

5-(*cis*-2-vinylcyclopropyl)benzofuran (S9)

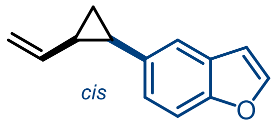

Synthesized following the General Procedure F using 5-bromobenzofuran (197 mg, 1 mmol, 1.0 equiv.) and a suspension of freshly prepared (*cis*-2-vinylcyclopropyl)zinc(II) chloride[6] (4.9 mL, 2.0 equiv.). Flash column chromatography (pentane/Et_2O 100:1) afforded the title product as a colorless oil (180 mg, 0.98 mmol, 98%). **R_f** = 0.2 (100:1 pentane:Et_2O, PMA). **^{1}H NMR** (600 MHz, $CDCl_3$) δ 7.59 (d, *J* = 2.2 Hz, 1H), 7.45 – 7.42 (m, 1H), 7.41 (d, *J* = 8.5 Hz, 1H), 7.18 (dd, *J* = 8.5, 1.8 Hz, 1H), 6.72 (dd, *J* = 2.2, 1.0 Hz, 1H), 5.17 – 5.03 (m, 2H), 4.83 (dd, *J* = 10.0, 2.3 Hz, 1H), 2.45 (td, *J* = 8.6, 6.3 Hz, 1H), 1.88 (qd, *J* = 8.7, 5.4 Hz, 1H), 1.30 (td, *J* = 8.4, 5.2 Hz, 1H), 1.06 (dt, *J* = 6.3, 5.3 Hz, 1H). **^{13}C NMR** (151 MHz, $CDCl_3$) δ 153.8, 145.2, 138.6, 133.3, 127.4, 126.2, 121.4, 113.9, 110.9, 106.6, 23.3, 22.9, 12.2. **HRMS** (ESI): *m/z* $[M]^+$ calculated for $C_{13}H_{12}O$: 184.0882, found 184.0883.

(4-hydroxyphenyl)(2-vinylcyclopropyl)methanone (S10)

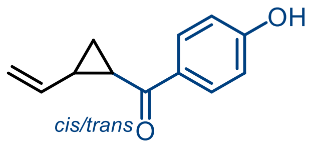

Prepared from *N*-methoxy-*N*-methyl-2-vinylcyclopropane-1-carboxamide **S23** (233 mg, 1.5 mmol) and (4(4-((trimethylsilyl)oxy)phenyl)magnesium bromide (9.1 mL, 2.0 equiv., 0.33 M in THF, see below) following General Procedure C with a reaction time of 20 h. The title product was obtained after purification by silica gel column chromatography (4:1 hexane:EtOAc) as a beige solid (268 mg, 1.43 mmol, 95%, 41:59 *cis*/*trans* (^{1}H NMR)). **R_f** = 0.22 (4:1 hexane:EtOAc). **M.p.** = 80.2 – 85.1 °C. **^{1}H NMR** (600 MHz, $CDCl_3$) δ 7.96 – 7.91 (m, 2H *cis* + 2H *trans*), 6.95 – 6.88 (m, 2H *cis* + 2H *trans*), 6.73 (s, 1H, *trans*), 6.65 (s, 1H, *cis*), 5.63 (ddd, *J* = 17.1, 10.3, 9.2 Hz, 1H, *cis*), 5.54 (ddd, *J* = 17.0, 10.3, 8.5 Hz, 1H, *trans*), 5.24 – 5.17 (m, 1H *cis* + 1H *trans*), 5.03 (dd, *J* = 10.3, 1.3 Hz, 1H, *trans*), 4.97 (dd, *J* = 10.3, 1.7 Hz, 1H, *cis*), 2.92 (ddd, *J* = 8.9, 7.5, 6.0 Hz, 1H, *cis*), 2.66 (ddd, *J* = 8.0, 5.2, 3.8 Hz, 1H, *trans*), 2.27 – 2.15 (m, 1H *cis* + 1H *trans*), 1.73 – 1.64 (m, 1H *cis* + 1H *trans*), 1.32 (ddd, *J* = 8.4, 7.5, 4.6 Hz, 1H, *cis*), 1.18 (ddd, *J* = 8.0, 6.4, 4.0 Hz, 1H, *trans*). **^{13}C NMR** (151 MHz, $CDCl_3$) δ 198.4, 197.1, 160.7, 160.5, 138.6, 135.1, 131.5, 130.9, 130.9, 130.7, 116.4, 115.6, 115.5, 115.2, 29.5, 28.2, 26.5, 25.7, 18.2, 14.3. **IR** (neat, cm^{-1}): 3155, 2814, 2469, 1633, 1602, 1566, 1513, 1441, 1386, 1291, 1224, 1163, 1053, 1026, 990, 911, 849, 810, 740, 663. **HRMS** (ESI): *m/z* $[M+Na]^+$ calculated for $C_{12}H_{12}O_2Na$: 211.0730, found 211.0722.

(4-((trimethylsilyl)oxy)phenyl)magnesium bromide

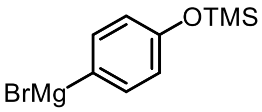

Inside an argon filled glovebox (4-bromophenoxy)trimethylsilane (1.23 g, 5 mmol, 1.0 equiv.) was placed to an oven-dried screw top vial. It was dissolved in dry THF (10 mL, 0.5 M) and fine magnesium shavings (243 mg, 10 mmol, 2.0 equiv.) were added. The mixture was stirred overnight at room temperature. The starting bromide was fully consumed and titration with iodine afforded a concentration of 0.33 M. The compound was used in the next step without further analysis.

(4-aminophenyl)(2-vinylcyclopropyl)methanone (S11)

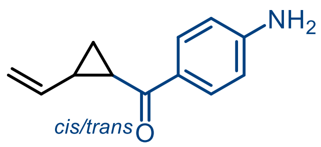

Prepared from *N*-methoxy-*N*-methyl-2-vinylcyclopropane-1-carboxamide **S23** (155 mg, 1.0 mmol) and (4-(bis(trimethylsilyl)amino)phenyl) magnesium chloride (4.0 mL, 2.0 equiv., 0.5 M in THF) following General Procedure C with a reaction time of 16 h. The crude was then dissolved in

10 mL MeOH, aqueous HCl (2.0 mL, 1M) was added and it was stirred over night at room temperature. The mixture was diluted with DCM (10 mL) and H_2O (10 mL) and phases were separated. The aqueous layer was further extracted with DCM (3x10 mL) and the combined organic layers were dried over Na_2SO_4 and concentrated under reduced pressure. The title product was obtained after purification by silica gel column chromatography (2:1 hexane:EtOAc, 1% Et_3N) as a beige solid (72 mg, 0.38 mmol, 38%, 37:63 *cis/trans* (^{1}H NMR)). **R_f** = 0.2 (2:1 hexane:EtOAc). **M.p.** = 96.8 – 99.1 °C. **^{1}H NMR** (600 MHz, $CDCl_3$) δ 7.88 – 7.84 (m, 2H *cis* + 2H *trans*), 6.68 – 6.63 (m, 2H *cis* + 2H *trans*), 5.65 (ddd, *J* = 17.1, 10.3, 9.3 Hz, 1H, *cis*), 5.53 (ddd, *J* = 17.0, 10.3, 8.5 Hz, 1H, *trans*), 5.18 (dt, *J* = 17.1, 1.6 Hz, 1H *cis* + 1H *trans*), 5.00 (dd, *J* = 10.3, 1.4 Hz, 1H, *trans*), 4.94 (dd, *J* = 10.3, 1.9 Hz, 1H, *cis*), 4.16 – 4.08 (m, 2H *cis* + 2H *trans*), 2.87 (ddd, *J* = 8.9, 7.5, 6.0 Hz, 1H, *cis*), 2.60 (ddd, *J* = 8.0, 5.2, 3.8 Hz, 1H, *trans*), 2.18 – 2.10 (m, 1H *cis* + 1H *trans*), 1.66 – 1.59 (m, 1H *cis* + 1H *trans*), 1.28 – 1.22 (m, 1H, *cis*), 1.10 (ddd, *J* = 8.0, 6.2, 3.9 Hz, 1H, *trans*). **^{13}C NMR** (151 MHz, $CDCl_3$) δ 196.6, 195.4, 151.1, 151.0, 139.1, 135.7, 130.7, 130.6, 129.2, 128.5, 115.7, 114.6, 113.9, 113.9, 28.6, 27.5, 26.0, 25.2, 17.6, 13.8. **IR** (neat, cm^{-1}): 3412, 3334, 3223, 3006, 2976, 2681, 2324, 2163, 1829, 1744, 1631, 1586, 1515, 1440, 1386, 1306, 1236, 1170, 1133, 1048, 1002, 910, 842, 790, 750, 696, 667. **HRMS** (ESI): *m/z* $[M+Na]^+$ calculated for $C_{12}H_{13}ONNa$: 210.0889, found 210.0886.

1-(2-vinylcyclopropyl)nonan-1-one (S12)

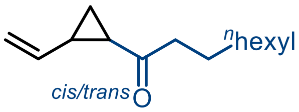

Prepared from *N*-methoxy-*N*-methyl-2-vinylcyclopropane-1-carboxamide **S23** (155 mg, 1.0 mmol) and octylmagnesium chloride (1.0 mL, 2.0 equiv., 2M in THF) following General Procedure C with a reaction time of 16 h. The title product was obtained after purification by silica gel column chromatography (50:1 pentane:Et_2O) as a colorless oil (199 mg, 0.95 mmol, 95%, 40:60 *cis/trans* (^{1}H NMR)). **R_f** = 0.32 (*trans*) and 0.29 (*cis*) (50:1 pentane:Et_2O, $KMnO_4$). **^{1}H NMR** (600 MHz, $CDCl_3$) δ 5.65 (dt, *J* = 17.1, 9.8 Hz, 1H, *cis*), 5.40 (ddd, *J* = 17.0, 10.3, 8.5 Hz, 1H, *trans*), 5.20 – 5.11 (m, 1H *cis* + 1H *trans*), 5.00 – 4.94 (m, 1H *cis* + 1H *trans*), 2.53 (td, *J* = 7.3, 1.9 Hz, 2H, *trans*), 2.50 (td, *J* = 7.3, 2.0 Hz, 2H, *cis*), 2.26 (ddd, *J* = 8.8, 7.6, 6.0 Hz, 1H, *cis*), 2.04 – 1.92 (m, 1H *cis* + 2H *trans*), 1.63 – 1.54 (m, 2H *cis* + 2H *trans*), 1.44 – 1.37 (m, 1H *cis* + 1H *trans*), 1.31 – 1.22 (m, 10H *cis* + 10H *trans*), 1.15 (td, *J* = 7.9, 4.5 Hz, 1H, *cis*), 0.98 (ddd, *J* = 8.1, 6.3, 3.9 Hz, 1H, *trans*), 0.89 – 0.85 (m, 3H *cis* + 3H *trans*). **^{13}C NMR** (151 MHz, $CDCl_3$) δ 209.4, 208.3, 138.7, 135.4, 115.8, 114.7, 44.9, 44.1, 32.0, 29.6, 29.5, 29.4, 29.4, 29.3, 28.4, 27.6, 24.1, 24.1, 22.8, 17.6, 14.8, 14.2. **IR** (neat, cm^{-1}): 3083, 3005, 2925, 2856, 2324, 2616, 1697, 1637, 1458, 1385, 1288, 1199, 1130, 1080, 990, 902, 839, 786, 723. **HRMS** (ESI): *m/z* $[M+Na]^+$ calculated for $C_{14}H_{24}ONa$: 231.1719, found 231.1716.

Naphthalen-1-yl 2-vinylcyclopropane-1-carboxylate (S13)

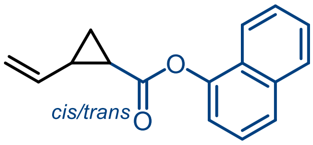

Prepared from 2-vinylcyclopropane-1-carboxylic acid **S17** (224 mg, 2.0 mmol) and naphthalen-1-ol (346 mg, 2.4 mmol) following General Procedure B with a reaction time of 16 h. The title product was obtained after purification by silica gel column chromatography (20:1 pentane:Et_2O) as a colorless oil (295 mg, 1.24 mmol, 62%, 37:63 *cis/trans* (^{1}H NMR)). **R_f** = 0.33 (20:1 pentane:Et_2O). **^{1}H NMR** (400 MHz, $CDCl_3$) δ 7.92 – 7.85 (m, 2H *cis* + 2H *trans*), 7.78 – 7.71 (m, 1H *cis* + 1H *trans*), 7.56 – 7.43 (m, 3H *cis* + 3H *trans*), 7.28 – 7.22 (m, 1H *cis* + 1H *trans*), 5.87 (ddd, *J* = 17.1, 10.2, 9.2 Hz, 1H, *cis*), 5.55 (ddd, *J* = 17.1, 10.2, 8.3 Hz, 1H, *trans*), 5.39 (dd, *J* = 17.2, 1.7 Hz, 1H, *cis*), 5.30 (d, *J* = 17.1, 1H, *trans*), 5.16 (dd, *J* = 10.3, 1.7 Hz, 1H, *cis*), 5.12 (dd, *J* = 10.3, 1.3 Hz, 1H, *trans*), 2.39 (ddd, *J* = 8.8, 7.8, 5.9 Hz, 1H, *cis*), 2.31 (qd, *J* = 8.8, 3.9 Hz, 1H, *trans*), 2.26 – 2.16 (m, 1H, *cis*), 2.08 (ddd, *J* = 8.7, 5.1, 4.0 Hz, 1H, *trans*),

1.64 (dt, *J* = 9.3, 4.8 Hz, 1H, *trans*), 1.52 – 1.43 (m, 2H, *cis*), 1.24 (ddd, *J* = 8.3, 6.4, 4.4 Hz, 1H, *trans*). **^{13}C NMR** (101 MHz, $CDCl_3$) δ 172.2, 170.8, 146.9, 146.7, 137.8, 135.1, 134.8, 128.2, 128.1, 127.0, 127.0, 126.6, 126.5, 126.5, 126.1, 125.5, 121.5, 121.3, 118.3, 118.2, 117.1, 115.6, 26.7, 25.9, 21.9, 21.1, 16.5, 15.0. **IR** (neat, cm^{-1}): 3061, 3011, 2326, 1745, 1636, 1598, 1508, 1443, 1378, 1318, 1222, 1130, 1047, 1012, 988, 909, 872, 846, 823, 769, 726. **HRMS** (ESI): *m/z* [M+Na]$^+$ calculated for $C_{16}H_{14}O_2Na$: 261.0886, found 261.0884.

4-oxo-2-phenyl-*4H*-chromen-3-yl 2-vinylcyclopropane-1-carboxylate (S14)

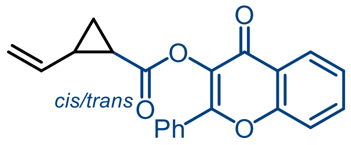

Prepared from 2-vinylcyclopropane-1-carboxylic acid **S17** (224 mg, 2.0 mmol) and 3-hydroxy-2-phenyl-4*H*-chromen-4-one (572 mg, 2.4 mmol) following General Procedure B with a reaction time of 18 h. The title product was obtained after purification by silica gel column chromatography (5:1 hexane:EtOAc) as a beige solid (329 mg, 1.0 mmol, 50%, 41:59 *cis/trans* (^{1}H NMR)). **R_f** = 0.18 (5:1 hexane:EtOAc). **M.p.** = 89.8 – 101.4 °C. **^{1}H NMR** (600 MHz, $CDCl_3$) δ 8.29 – 8.23 (m, 1H *cis* + 1H *trans*), 7.87 (d, *J* = 6.7 Hz, 2H *trans*), 7.83 (d, *J* = 7.6 Hz, 2H *cis*), 7.71 – 7.68 (m, 1H *cis* + 1H *trans*), 7.57 – 7.46 (m, 4H *cis* + 4H *trans*), 7.44 – 7.38 (m, 1H *cis* + 1H *trans*), 5.73 (dt, *J* = 18.8, 10.0 Hz, 1H, *cis*), 5.46 (dt, *J* = 18.1, 9.4 Hz, 1H, *trans*), 5.29 (d, *J* = 14.8 Hz, 1H, *cis*), 5.22 (d, *J* = 17.0 Hz, 1H, *trans*), 5.09 – 5.03 (m, 1H *cis* + 1H *trans*), 2.29 (q, *J* = 7.9 Hz, 1H, *cis*), 2.22 – 2.16 (m, 1H, *trans*), 2.12 (t, *J* = 8.6 Hz, 1H, *cis*), 1.96 (q, *J* = 4.2 Hz, 1H, *trans*), 1.56 (q, *J* = 4.2 Hz, 1H, *trans*), 1.45 – 1.37 (m, 2H, *cis*), 1.19 – 1.14 (m, 1H, *trans*). **^{13}C NMR** (151 MHz, $CDCl_3$) δ 172.4, 172.3, 170.6, 169.4, 156.4, 156.3, 155.7, 137.6, 134.7, 134.0, 134.0, 133.8, 133.7, 131.3, 131.3, 130.1, 130.1, 128.7, 128.7, 128.4, 128.4, 126.3, 126.2, 125.3, 125.2, 123.8, 118.2, 118.2, 116.8, 115.6, 26.7, 26.1, 21.8, 20.7, 16.3, 15.2. **IR** (neat, cm^{-1}): 3074, 2324, 2168, 1752, 1642, 1570, 1468, 1385, 1287, 1239, 1188, 1117, 987, 956, 899, 848, 757, 692. **HRMS** (ESI): *m/z* [M+Na]$^+$ calculated for $C_{21}H_{16}O_4Na$: 355.0941, found 355.0930.

Octyl 2-vinylcyclopropane-1-carboxylate (S15)

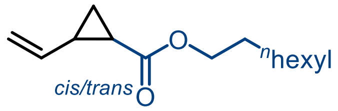

Prepared from 2-vinylcyclopropane-1-carboxylic acid **S17** (224 mg, 2.0 mmol) and octan-1-ol (313 mg, 2.4 mmol) following General Procedure B with a reaction time of 16 h. The title product was obtained after purification by silica gel column chromatography (30:1 pentane:Et_2O) as a colorless oil (214 mg, 0.95 mmol, 48%, 50:50 *cis/trans* (^{1}H NMR)). **R_f** = 0.4 (25:1 pentane:Et_2O, $KMnO_4$). **^{1}H NMR** (600 MHz, $CDCl_3$) δ 5.77 (dddd, *J* = 17.4, 9.5, 7.4, 1.7 Hz, 1H, *cis*), 5.39 (ddd, *J* = 17.0, 10.3, 8.4 Hz, 1H, *trans*), 5.22 (dd, *J* = 17.1, 1.8 Hz, 1H, *cis*), 5.15 (dd, *J* = 17.1, 1.3 Hz, 1H, *trans*), 5.03 (dd, *J* = 10.3, 1.8 Hz, 1H, *cis*), 4.98 (dd, *J* = 10.3, 1.4 Hz, 1H *trans*), 4.09 – 4.02 (m, 2H *cis* + 2H *trans*), 2.05 – 1.97 (m, 1H, *trans*), 1.97 – 1.87 (m, 2H, *cis*), 1.66 – 1.57 (m, 2H *cis* + 3H *trans*), 1.39 – 1.23 (m, 11H *cis* + 11H *trans*), 1.21 (td, *J* = 8.2, 4.8 Hz, 1H, *cis*), 0.96 (ddd, *J* = 8.3, 6.2, 4.3 Hz, 1H, *trans*), 0.90 – 0.84 (m, 3H *cis* + 3H *trans*). **^{13}C NMR** (151 MHz, $CDCl_3$) δ 173.6 (*trans*), 172.1 (*cis*), 138.3 (*trans*), 135.6 (*cis*), 116.2 (*cis*), 114.8 (*trans*), 65.0 (*trans*), 64.8 (*cis*), 31.9 (*cis* + *trans*), 29.3 (*cis* + *trans*), 29.3 (*cis* + *trans*), 28.9 (*cis*), 28.8 (*trans*), 26.0 (*cis* + *trans*), 25.6 (*trans*), 24.9 (*cis*), 22.8 (*cis* + *trans*), 22.0 (*trans*), 21.1 (*cis*), 15.7 (*trans*), 14.2 (*cis*), 14.2 (*cis* + *trans*). **IR** (neat, cm^{-1}): 2926, 2857, 2326, 2090, 1726, 1638, 1460, 1401, 1372, 1266, 1167, 1084, 1049, 991, 904, 854, 815, 790, 726. **HRMS** (ESI): *m/z* [M+Na]$^+$ calculated for $C_{14}H_{24}O_2Na$: 247.1669, found 247.1665.

Ethyl 2-vinylcyclopropane-1-carboxylate (S16)

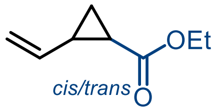

Prepared from butadiene (55 mL, 10 equiv., 15% in hexane) and ethyl diazoacetate (1.14 g, 10 mmol, 1.0 equiv.) following General Procedure A - step 1. The title product was obtained after purification by silica gel column chromatography (50:1 → 20:1 → 10:1 pentane:Et_2O) as a colorless oil (627 mg, 4.47 mmol, 45%, 44:56 *cis/trans* (^{1}H NMR)). **R$_f$** = 0.18 (50:1 pentane:Et_2O, PMA). **^{1}H NMR** (600 MHz, $CDCl_3$) δ 5.82 – 5.73 (m, 1H, *cis*), 5.39 (ddd, *J* = 17.1, 10.3, 8.4 Hz, 1H, *trans*), 5.23 (dd, *J* = 17.1, 1.9 Hz, 1H, *cis*), 5.16 (dd, *J* = 17.1, 1.6 Hz, 1H, *trans*), 5.04 (dd, *J* = 10.3, 1.9 Hz, 1H, *cis*), 4.98 (dd, *J* = 10.2, 1.5 Hz, 1H, *trans*), 4.16 – 4.11 (m, 2H *cis*, 2H *trans*), 2.01 (tdd, *J* = 8.8, 6.1, 3.9 Hz, 1H, *trans*), 1.95 – 1.89 (m, 2H, *cis*), 1.63 (ddd, *J* = 8.7, 5.2, 3.9 Hz, 1H, *trans*), 1.38 – 1.34 (m, 1H, *trans*), 1.29 – 1.23 (m, 3H *trans,* 4H *cis*), 1.21 (dt, *J* = 8.3, 4.2 Hz, 1H, *cis*), 0.97 (ddd, *J* = 8.4, 6.2, 4.3 Hz, 1H, *trans*). **^{13}C NMR** (151 MHz, $CDCl_3$) δ 173.5, 172.0, 138.3, 135.6, 116.2, 114.9, 60.7, 60.6, 25.6, 24.8, 22.0, 21.1, 15.6, 14.5, 14.4, 14.2. **IR** (neat, cm^{-1}): 2983, 1723, 1638, 1448, 1384, 1269, 1168, 1093, 1036, 987, 906. **HRMS** (ESI): *m/z* [M]$^+$ calculated for $C_8H_{12}O_2$: 140.0832, found 140.0833.

2-vinylcyclopropane-1-carboxylic acid (S17)

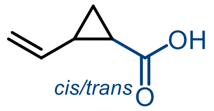

Synthesized according to a modified literature procedure:[14,15] Ethyl 2-vinylcyclopropane-1-carboxylate **S16** (627 mg, 4.47 mmol, 1.0 equiv.) was placed into a 25 mL round bottom flask and aqueous KOH (2.5 mL, 1.5 equiv. 3 M) was added before it was heated at reflux for 3 h. The mixture was cooled down to room temperature and it was diluted with DCM (10 mL) and 10 mL of an aqueous HCl solution (1 M) was added. The phases were separated, and the aqueous phase was extracted with DCM (2 x 10 mL). The combined organic extracts were dried over Na_2SO_4, filtered, and concentrated under reduced pressure (600 mbar, 40 °C). The crude acid was obtained as a colourless liquid in high purity and was used directly for the next step without further purification (464 mg, 4.14 mmol. 88%, 44:56 *cis/trans* (^{1}H NMR)). **^{1}H NMR** (600 MHz, $CDCl_3$) δ 5.79 (ddd, *J* = 17.1, 10.3, 9.2 Hz, 1H, *cis*), 5.40 (ddd, *J* = 17.0, 10.2, 8.3 Hz, 1H, *trans*), 5.26 (dd, *J* = 17.1, 1.7 Hz, 1H, *cis*), 5.18 (dt, *J* = 17.0, 1.0 Hz, 1H, *trans*), 5.08 (dd, *J* = 10.3, 1.8 Hz, 1H, *cis*), 5.01 (dd, *J* = 10.3, 1.3 Hz, 1H, *trans*), 2.09 (tdd, *J* = 8.6, 6.3, 3.8 Hz, 1H, *trans*), 2.07 – 1.98 (m, 1H, *cis*), 1.92 (ddd, *J* = 8.7, 7.5, 6.2 Hz, 1H, *cis*), 1.64 (ddd, *J* = 8.6, 5.1, 3.8 Hz, 1H, *trans*), 1.43 (dt, *J* = 9.2, 4.8 Hz, 1H, *trans*), 1.32 – 1.28 (m, 2H, *cis*), 1.06 (ddd, *J* = 8.3, 6.4, 4.4 Hz, 1H, *trans*). *Note*: The acid proton was not observed in ^{1}H NMR due to H-D exchange. **^{13}C NMR** (151 MHz, $CDCl_3$) δ 180.2, 178.9, 137.7, 135.0, 116.9, 115.5, 26.6, 26.0, 21.9, 21.0, 16.3, 15.1. **IR** (neat, cm^{-1}): 2916, 2648, 2567, 1690, 1640, 1434, 1357, 1292, 1228, 1084, 1052, 980, 905, 854, 677. **HRMS** (ESI): *m/z* [M-CO_2H]$^+$ calculated for C_5H_7: 67.0542, found 67.0542.

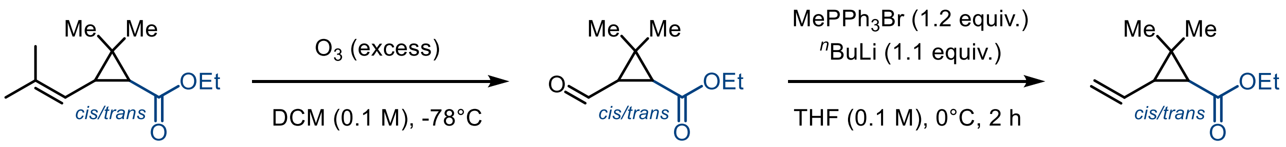

Ethyl-3-formyl-2,2-dimethylcyclopropane-1-carboxylate

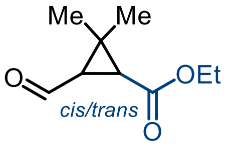

Ethyl 2,2-dimethyl-3-(2-methylprop-1-en-1-yl)cyclopropane-1-carboxylate (1.70 g, 10.00 mmol, 1.0 equiv.) was dissolved in dry DCM (0.1 M, 100 mL) and cooled to -78°C. Ozone was bubbled through the cooled solution until a blue color persisted in the flask (ca. 15 min). To remove the excess ozone, oxygen was bubbled through the solution until the blue color vanished. Triphenylphosphine (2.89 g, 11.0 mmol, 1.1 equiv.)

was added at -78°C and the solution was allowed to warm up to room temperature. The solvent was removed under reduced pressure and pentane/Et_20 (3:1) was added to precipitate triphenylphosphine oxide. The mixture was stirred for an additional 1 h and then was filtered through Celite. The mixture was concentrated under reduced pressure and the crude aldehyde was obtained as colorless oil. It was directly used in the next step without further purification.

Ethyl 2,2-dimethyl-3-vinylcyclopropane-1-carboxylate

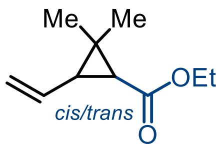

Synthesized from ethyl-3-formyl-2,2-dimethylcyclopropane-1-carboxylate (10.00 mmol, 1.0 equiv.) following General Procedure A - step 4. Flash column chromatography (200:1 pentane:Et_2O) afforded the title product as a colorless oil (846,0 mg, 5.03 mmol, 50% over 2 steps, 27:73 *cis/trans* (^{1}H NMR)). **R_f** = 0.17 (200:1 pentane:Et_2O). **^{1}H NMR** (600 MHz, $CDCl_3$) δ 6.18 – 6.08 (m, 1H, *cis*), 5.62 – 5.53 (m, 1H, *trans*), 5.23 – 5.17 (m, 1H, *cis* + 1H, *trans*), 5.10 – 5.05 (m, 1H, *cis* + 1H, *trans*), 4.19 – 4.05 (m, 2H, *cis* + 2H, *trans*), 2.05 (dd, *J* = 8.4, 5.4 Hz, 1H, *trans*), 1.82 (t, *J* = 9.2 Hz, 1H, *cis*), 1.70 (d, *J* = 8.6 Hz, 1H, *cis*), 1.56 (d, *J* = 5.4 Hz, 1H, *trans*), 1.30 – 1.23 (m, 6H, *cis* + 6H, *trans*), 1.19 (s, 3H, *cis*), 1.16 (s, 3H, *trans*). **^{13}C NMR** (151 MHz, $CDCl_3$) δ 172.1 (*trans*), 171.2 (*cis*), 135.5 (*trans*), 133.4 (*cis*), 116.5 (*trans*), 116.4 (*cis*), 60.5 (*trans*), 60.1 (*cis*), 37.0 (*cis*), 36.8 (*trans*), 34.0 (*trans*), 32.2 (*cis*), 28.8 (*cis* + *trans*), 27.1 (*cis*), 22.0 (*trans*), 20.5 (*trans*), 14.8 (*cis*), 14.5 (*trans*), 14.5 (*cis*). **HRMS** (EI): *m/z* $[M+Na]^+$ calculated for $C_{10}H_{16}O_2Na$: 191.1048, found 191.1041.

Ethyl *cis*-2,2-dimethyl-3-vinylcyclopropane-1-carboxylate (S18)

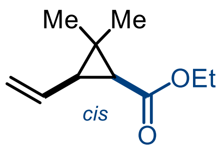

Separated from the *cis/trans* mixture of ethyl 2,2-dimethyl-3-vinylcyclopropane-1-carboxylate via preparative HPLC (column: LiChrosorb Si (250x25)mm, 7 µm; mobile phase: 9:1 hexane:EtOAc; flow: 15 mL/min; pressure: 32 bar A, 30 bar B) (8:92 *trans/cis* (^{1}H NMR)). **^{1}H NMR** (600 MHz, $CDCl_3$) δ 6.13 (ddd, *J* = 17.1, 10.4, 9.6 Hz, 1H), 5.20 (dd, *J* = 17.2, 2.1 Hz, 1H), 5.08 (dd, *J* = 10.4, 2.1 Hz, 1H), 4.12 – 4.08 (m, 2H), 1.82 (t, *J* = 9.2 Hz, 1H), 1.69 (d, *J* = 8.6 Hz, 1H), 1.29 (s, 3H), 1.25 (t, *J* = 7.1 Hz, 3H), 1.19 (s, 3H). **^{13}C NMR** (151 MHz, $CDCl_3$) δ 171.2, 133.4, 116.4, 60.1, 37.0, 32.2, 28.8, 27.1, 14.8, 14.5. **IR** (neat, cm^{-1}): 2924 (s), 2321 (w), 1726 (s), 1633 (m), 1383 (s), 1336 (m), 1185 (s), 1093 (s), 904 (m), 841 (w), 575 (w). **HRMS** (EI): *m/z* $[M+Na]^+$ calculated for $C_{10}H_{16}O_2Na$: 191.1048, found 191.1043.

Ethyl *cis*-2-methyl-2-(prop-1-en-2-yl)cyclopropane-1-carboxylate (S19)

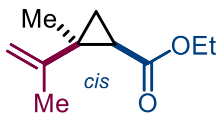

Prepared following General Procedure A from ethyl 2-diazoacetate (2.6 mL, 20.00 mmol, 1.0 equiv.), 2,3-dimethylbuta-1,3-diene (8.2 g, 100 mmol, 5.0 equiv.) and $Rh_2(OAc)_4$ (1 mol%, 17.7 mg, 0.2 mmol, 0.01 equiv.). Flash column chromatography afforded the title product as a colorless oil (910.1 mg, 5.41 mmol, 27%, 92:8 *cis/trans* (^{1}H NMR)). **R_f** = 0.15 (200:1 pentane:Et_2O). **^{1}H NMR** (600 MHz, $CDCl_3$) δ 4.90 – 4.89 (m, 1H), 4.90 – 4.87 (m, 1H), 4.08 (q, *J* = 7.1 Hz, 2H), 1.72 – 1.69 (m, 3H), 1.64 (dd, *J* = 7.8, 5.5 Hz, 1H), 1.51 (t, *J* = 5.0 Hz, 1H), 1.26 (s, 3H), 1.23 (t, *J* = 7.1 Hz, 3H), 0.95 (dd, *J* = 7.8, 4.5 Hz, 1H). **^{13}C NMR** (151 MHz, $CDCl_3$) δ 171.8, 144.5, 113.6, 60.4, 33.6, 27.9, 25.4, 20.8, 20.5, 14.5. **IR** (neat, cm^{-1}): 2970 (m), 2331 (w), 1727 (s), 1648 (w), 1380 (m), 1162 (s), 1064 (m), 895 (m), 841 (m), 680 (w). **HRMS** (EI): *m/z* $[M+Na]^+$ calculated for $C_{10}H_{16}O_2Na$: 191.1048, found 191.1043.

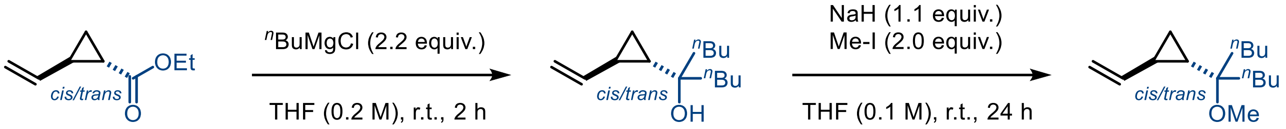

5-(2-vinylcyclopropyl)nonan-5-ol

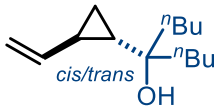

Ethyl 2-vinylcyclopropane-1-carboxylate **S16** (1.40 g, 10.00 mmol, 1.0 equiv.) was dissolved in dry THF (50 mL, 0.2 M) and cooled down to 0 °C. Butyl magnesium chloride solution (25.00 mmol, 2.5 equiv., 1.0 M in THF) was added dropwise over the course of 15 min to the stirring solution at 0 °C. The reaction mixture was allowed to warm to room temperature and was stirred overnight. The mixture was quenched by slowly adding 50 mL of sat. NH_4Cl. Next, the phases were separated, and the aqueous layer was extracted with Et_2O (2 x 50 mL). The combined organic layers were dried over Na_2SO_4, filtered, and concentrated under reduced pressure. The crude was used directly for the next step without further purification and analysis.

1-(5-methoxynonan-5-yl)-2-vinylcyclopropane (S20)

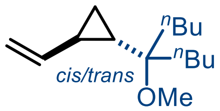

To a suspension of NaH (293.3 mg, 11.00 mmol, 1.1 equiv.) in THF (100 mL, 0.1 M) was added dropwise a solution of 5-(2-vinylcyclopropyl)nonan-5-ol (2.10 g, 10.00 mmol, 1.0 equiv.) in THF (20 mL) at 0 °C. After stirring for 2 h at room temperature, a solution of MeI (1.87 mL, 30.00 mmol, 3.0 equiv.) in THF (20 mL) was added at 0 °C. The solution was stirred at room temperature for 24 h. The mixture was quenched by adding H_2O (100 mL). Next, the phases were separated, and the aqueous layer was extracted with Et_2O (3 x 100 mL). The combined organic layers were dried over Na_2SO_4, filtered, and concentrated under reduced pressure. Purification by silica gel column chromatography (100:1 hexane:EtOAc) afforded the title compound as a colorless oil in two separate fractions (*fraction 1*: 110 mg, 0.490 mmol, 5%, 30:70 cis/trans (^{1}H NMR); *fraction 2*: 210 mg, 0.935 mmol, 9%, 12:88 cis/trans (^{1}H NMR)). *Note: Fraction 1 was obtained in 90% purity and was used without further purification for all further reactions.*
R_f = 0.2 (100:1 hexane:EtOAc, PMA). **^{1}H NMR** (400 MHz, $CDCl_3$) δ 5.85 (dt, *J* = 17.5, 9.9 Hz, 1H *cis*), 5.37 (ddd, *J* = 17.3, 10.2, 8.7 Hz, 1H *trans*), 5.11 – 5.00 (m, 1H *cis*, 1H *trans*), 4.90 – 4.80 (m, 1H *cis*, 1H *trans*), 3.14 (s, 3H *trans*), 3.12 (s, 3H *cis*), 1.98 (q, *J* = 7.5, 6.9 Hz, 2H *cis*), 1.58 – 1.21 (m, 13H *trans,* 14H *cis*), 0.98 – 0.82 (m, 6H *trans*, 6H *cis*), 0.85 – 0.72 (m, 2H *trans*), 0.52 (ddd, *J* = 7.9, 5.1, 3.4 Hz, 1H *trans*). **^{13}C NMR** (101 MHz, $CDCl_3$): δ 142.0, 140.6, 112.4, 112.0, 77.0, 76.5, 49.2, 48.6, 36.8, 36.6, 35.6, 34.8, 27.7, 26.5, 26.3, 25.8, 25.6, 23.6, 23.6, 20.4, 18.1, 14.3, 10.0, 9.9. **IR** (neat, cm^{-1}): 3080, 2936, 2868, 2827, 2325, 2194, 2164, 2069, 2029, 1924, 1791, 1701, 1635, 1461, 1379, 1335, 1259, 1199, 1170, 1081, 1040, 985, 889, 826, 779, 730, 659. **HRMS** (ESI): *m/z* $[M+Na]^+$ calculated for $C_{15}H_{28}ONa$: 247.2032, found 247.2031.

***N,N*-diethyl-2-vinylcyclopropane-1-carboxamide (S21)**

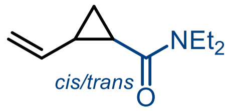

Prepared from 2-vinylcyclopropane-1-carboxylic acid **S17** (56 mg, 0.5 mmol) and diethylamine (55 µL, 0.525 mmol) following General Procedure D. The title product was obtained after purification by silica gel column chromatography (3:2 hexane:EtOAc) as a colorless oil (46 mg, 0.275 mmol, 55%, 44:56 *cis/trans* (^{1}H NMR)). **R_f** = 0.32 (3:2 hexane:EtOAc, PMA). **^{1}H NMR** (600 MHz, C_6D_6) δ 5.94 (dt, *J* = 17.3, 9.6 Hz, 1H, *cis*), 5.28 – 5.20 (m, 1H, *trans*), 5.15 (dd, *J* = 17.2, 2.0 Hz, 1H, *cis*), 5.03 (dd, *J* = 17.0, 1.4 Hz, 1H, *trans*), 4.96 (dd, *J* = 10.3, 2.0 Hz, 1H, *cis*), 4.87 (dd, *J* = 10.2, 1.4 Hz, 1H, *trans*), 3.36 – 3.23 (m, 1H *cis* + 1H *trans*), 3.23 – 3.09 (m, 1H *cis* +

1H *trans*), 2.99 (dq, *J* = 14.2, 7.1 Hz, 1H, *cis*), 2.86 (q, *J* = 7.1 Hz, 2H, *trans*), 2.78 (dq, *J* = 14.3, 7.1 Hz, 1H, *cis*), 2.20 (tdd, *J* = 8.8, 5.7, 4.3 Hz, 1H, *trans*), 1.72 (q, *J* = 5.9 Hz, 1H, *cis*), 1.67 (dt, *J* = 8.7, 4.4 Hz, 1H, *trans*), 1.55 – 1.48 (m, 2H, *cis*), 1.48 – 1.44 (m, 1H, *trans*), 0.97 – 0.92 (m, 3H *cis* + 3H *trans*), 0.81 – 0.75 (m, 4H *cis* + 3H *trans*), 0.65 (ddd, *J* = 8.2, 5.9, 3.7 Hz, 1H, *trans*). **^{13}C NMR** (151 MHz, C_6D_6) δ 170.2, 168.4, 139.7, 137.5, 114.7, 113.9, 41.9, 41.7, 41.1, 40.7, 25.2, 24.1, 20.9, 20.6, 15.0, 14.7, 13.6, 12.5. **IR** (neat, cm^{-1}): 2975, 2932, 1631, 1456, 1430, 1375, 1255, 1221, 1138, 1082, 899, 786. **HRMS** (ESI): *m/z* [M+Na]$^+$ calculated for $C_{10}H_{17}ONNa$: 190.1202, found 190.1201.

N-([1,1'-biphenyl]-4-yl)-2-vinylcyclopropane-1-carboxamide (S22)

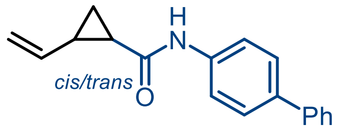

Prepared from 2-vinylcyclopropane-1-carboxylic acid **S17** (112 mg, 0.5 mmol) and 4-aminobiphenyl (180 mg, 1.05 mmol) following General Procedure D. The title product was obtained after purification by silica gel column chromatography (3:2 pentane:EtOAc) as a beige solid (88 mg, 0.33 mmol, 67%, 39:61 *cis/trans* (^{1}H NMR)). **M.p.** = 138.6 – 145.6 °C. **R$_f$** = 0.32 (*trans*) and 0.15 (*cis*) (4:1 hexane:EtOAc). **^{1}H NMR** (600 MHz, $CDCl_3$) δ 7.64 – 7.51 (m, 7H *cis* + 7H *trans*), 7.42 (dd, *J* = 7.7 Hz, 2H *cis* + 2H *trans*), 7.33 (dd, *J* = 7.4 Hz, 1H *cis* + 1H *trans*), 5.87 (ddd, *J* = 17.2, 10.4, 9.2 Hz, 1H *cis*), 5.45 (ddd, *J* = 17.1, 10.3, 8.5 Hz, 1H *trans*), 5.25 (dd, *J* = 17.1, 1.8 Hz, 1H *cis*), 5.20 (dd, *J* = 17.0, 1.3 Hz, 1H *trans*), 5.05 (dd, *J* = 10.3, 1.8 Hz, 1H *cis*), 5.02 (dd, *J* = 10.3, 1.4 Hz, 1H *trans*), 2.13 (tdd, *J* = 8.7, 6.2, 3.9 Hz, 1H *trans*), 1.96 (qd, *J* = 8.7, 6.7 Hz, 1H *cis*), 1.89 (td, *J* = 8.3, 5.8 Hz, 1H *cis*), 1.57 (dt, *J* = 8.5, 4.5 Hz, 1H *trans*), 1.52 (dt, *J* = 9.2, 4.7 Hz, 1H *trans*), 1.45 (q, *J* = 5.8 Hz, 1H *cis*), 1.24 (tt, *J* = 8.2, 4.2 Hz, 1H *cis*), 1.01 (ddd, *J* = 8.0, 6.2, 4.3 Hz, 1H *trans*). **^{13}C NMR** (151 MHz, $CDCl_3$) δ 170.5, 168.9, 140.6, 140.6, 138.5, 137.5, 137.4, 137.1, 137.1, 135.7, 128.9, 127.7, 127.2, 126.9, 120.1, 116.1, 114.9, 25.5, 25.1, 25.1, 24.0, 15.4, 13.3. **IR** (neat, cm^{-1}): 3292, 3033, 1650, 1596, 1532, 1487, 1448, 1401, 1316, 1203, 1077, 1004, 899, 833, 758, 685. **HRMS** (ESI): *m/z* [M+Na]$^+$ calculated for $C_{18}H_{17}ONNa$: 286.1202, found 286.1206.

N-methoxy-N-methyl-2-vinylcyclopropane-1-carboxamide (S23)

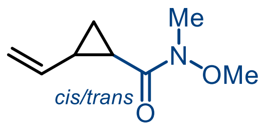

Prepared from 2-vinylcyclopropane-1-carboxylic acid **S17** (560 mg, 5.0 mmol), *N,O*-dimethylhydroxylamine hydrochloride (585 mg, 6.0 mmol) and Et_3N (2.1 mL, 3.0 equiv.) following General Procedure B with a reaction time of 18 h. The crude was then dissolved in a minimum amount of Et_2O and filtered over a plug of silica washed with Et_2O and concentrated. The title product was obtained in high purity as a clear oil and was used without further purification (732 mg, 4.7 mmol, 94%, 42:58 *cis/trans* (^{1}H NMR)). **^{1}H NMR** (400 MHz, $CDCl_3$) δ 5.67 (dt, *J* = 17.0, 9.9 Hz, 1H *cis*), 5.44 (ddd, *J* = 17.2, 10.2, 8.5 Hz, 1H *trans*), 5.24 – 5.10 (m, 1H *cis* + 1H *trans*), 5.03 – 4.92 (m, 1H *cis* + 1H *trans*), 3.72 (s, 3H *trans*), 3.70 (s, 3H *cis*), 3.20 (s, 3H *cis* + 3H *trans*), 2.44 (br, 1H *cis*), 2.16 (br, 1H *trans*), 2.03 – 1.87 (m, 1H *cis* + 1H *trans*), 1.46 – 1.31 (m, 1H *cis* + 1H *trans*), 1.15 (td, *J* = 8.1, 4.6 Hz, 1H *cis*), 0.94 (ddd, *J* = 8.3, 6.0, 4.0 Hz, 1H *trans*). **^{13}C NMR** (151 MHz, $CDCl_3$) δ 138.9, 136.3, 115.6, 114.5, 61.8, 61.7, 32.7, 25.5, 24.7, 19.3, 18.9, 15.1, 12.6. *Note:* The carbonyl carbon was not observed in ^{13}C NMR. **IR** (neat, cm^{-1}): 3082, 3004, 2967, 2938, 2821, 2325, 2172, 2090, 1651, 1421, 1390, 1333, 1175, 1101, 1048, 1000, 966, 903, 845, 823, 791, 765, 720, 665. **HRMS** (ESI): *m/z* [M+Na]$^+$ calculated for $C_8H_{13}O_2NNa$: 178.0839, found 178.0837.

(*cis*-2-vinylcyclopropyl)triethylgermane (S24)

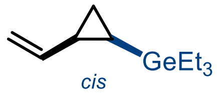

Inside the Glovebox an oven dried flask was charged with *cis*-1-Iodo-2-vinylcyclopropane[6] (194 mg, 1mmol, 1.0 equiv.) and anhydrous THF (2.5 mL, 0.4 M). Outside the Glovebox the mixture was connected to the Schlenk line and cooled to -78 °C. Next, ^{t}BuLi (1.18 mL, 2.05 equiv., 1.7 M in hexane) was added dropwise over a period of 15 min using a syringe pump. After complete addition the mixture was stirred for further 30 min at –78 °C before neat Et_3GeCl (0.2 mL, 1.2 mmol, 1.2 equiv.) was added. The mixture was allowed to warm up to room temperature and stirred overnight. At the next day water (10 mL) and pentane (10 mL) were added, phases were separated and the aqueous phase was extracted with pentane (2x 10 mL). The combined organic phases were dried over Na_2SO_4, filtered and concentrated under reduced pressure. Flash column chromatography (pentane) afforded the title product as a colorless liquid (51 mg, 0.22 mmol, 22%). **R$_f$** = 0.8 (pentane, PMA). **^{1}H NMR** (600 MHz, $CDCl_3$) δ 5.36 (ddd, *J* = 16.9, 10.1, 9.2 Hz, 1H), 5.12 (dd, *J* = 16.9, 1.9 Hz, 1H), 4.87 (dd, *J* = 10.2, 1.9 Hz, 1H), 1.68 (tdd, *J* = 9.1, 7.8, 4.7 Hz, 1H), 1.04 (t, *J* = 7.9 Hz, 10H), 0.74 (p, *J* = 7.9 Hz, 6H), 0.41 (dt, *J* = 7.8, 4.4 Hz, 1H), 0.18 (td, *J* = 9.6, 7.8 Hz, 1H). **^{13}C NMR** (151 MHz, $CDCl_3$) δ 142.4, 112.7, 19.2, 10.3, 9.1, 5.1, 4.9. **HRMS** (ESI): *m/z* [M-Et]$^+$ calculated for $C_9H_{17}Ge$: 199.0536, found 199.0532. *Note: Attention, compound is volatile.*

6-methyl-2-(2-vinylcyclopropyl)-1,3,6,2-dioxazaborocane-4,8-dione (S25)

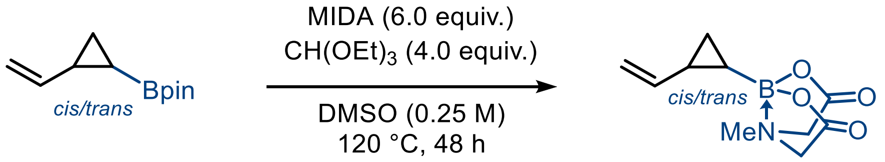

A 20 mL screw cap vial was charged with 4,4,5,5-tetramethyl-2-(2-vinylcyclopropyl)-1,3,2-dioxaborolane **S26** (100 mg, 0.52 mmol, 1.0 equiv.), *N*-methyliminodiacetic acid (455 mg, 3.1 mmol, 6.0 equiv.), triethyl orthoformate (0.342 mL, 2.1 mmol, 4.0 equiv.) and anhydrous DMSO (2 mL, 0.25 M). The vial was closed properly using a standard screw cap and the mixture was stirred vigorously at 120 °C for 48 hours. The reaction was then allowed to cool to room temperature, diluted with ethyl acetate (20 mL) and washed with sat. $NaHCO_3$ (2x10 mL) and brine (10 mL), dried over Na_2SO_4 and concentrated *in vacuo*. Purification by silica gel column chromatography (20:1 DCM:MeOH) afforded the title compound as a diastereomeric mixture as a white solid (60 mg, 0.27 mmol, 52%, 35:65 *cis/trans* (^{1}H NMR)). **R$_f$** = 0.17 (20:1 DCM:MeOH, PMA). **M.p.** = 44.8–48.6 °C. **^{1}H NMR** (600 MHz, CD_3CN) δ 5.58 (dt, *J* = 17.3, 10.1 Hz, 1H, *cis*), 5.37 (dt, *J* = 17.5, 9.6 Hz, 1H, *trans*), 5.15 (dd, *J* = 17.1, 2.1 Hz, 1H, *cis*), 5.10 (dd, *J* = 17.2, 1.8 Hz, 1H, *trans*), 4.90 (dd, *J* = 10.3, 2.1 Hz, 1H, *cis*), 4.83 (dd, *J* = 10.2, 1.8 Hz, 1H, *trans*), 3.97 – 3.89 (m, 2H *cis* + 2H *trans*), 3.85 – 3.78 (m, 2H *cis* + 2H *trans*), 2.96 (s, 3H, *cis*), 2.94 (s, 3H, *trans*), 1.66 (dt, *J* = 14.7, 9.1 Hz, 1H, *cis*), 1.31 (tt, *J* = 8.7, 5.2 Hz, 1H, *trans*), 1.01 – 0.95 (m, 1H, *cis*), 0.66 – 0.58 (m, 2H, *trans*), 0.42 (dt, *J* = 8.3, 4.3 Hz, 1H, *cis*), 0.08 (q, *J* = 9.1 Hz, 1H, *cis*), -0.16 (dt, *J* = 9.8, 6.3 Hz, 1H, *trans*). **^{11}B NMR** (193 MHz, CD_3CN) δ 17.9, 17.6. **^{13}C NMR** (151 MHz, CD_3CN) δ 169.2, 169.2, 169.1, 169.0, 144.0, 141.9, 113.9, 111.9, 63.1, 63.1, 63.0, 63.0, 47.4, 47.4, 20.6, 19.6, 10.8, 10.7, 7.5, 6.6. **MS** (70eV, EI): *trans isomer* (GC retention time 11.11 min), *m/z* (%): 223 (1) [M$^+$], 156 (100) [MIDA$^+$], 128 (15), 100 (42), 70 (10), 66 (3) [vinyl cyclopropyl$^+$]; *cis isomer* (GC retention time 11.24 min), *m/z* (%): *m/z* (%): 223 (1) [M$^+$], 207 (1), 156 (100) [MIDA$^+$], 128 (14), 100 (41), 70 (10), 66 (3) [vinyl cyclopropyl$^+$]. **IR**

(neat, cm^{-1}): 3001, 2960, 2923, 1744, 1634, 1456, 1337, 1289, 1248, 1124, 1075, 993, 961, 890, 861, 709, 664. **HRMS** (ESI): *m/z* [M+Na]$^+$ calculated for $C_{10}H_{14}O_4N^{11}BNa$: 246.0908, found 246.0907.

4,4,5,5-tetramethyl-2-(2-vinylcyclopropyl)-1,3,2-dioxaborolane (S26)

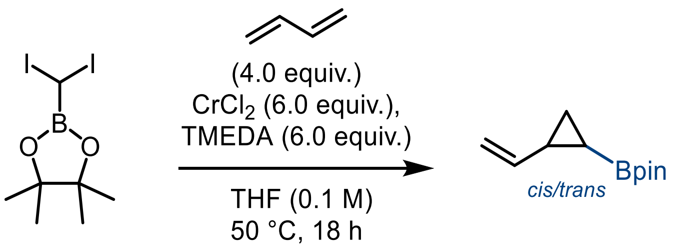

Synthesized according to a modified literature procedure:[16] In an argon-filled glovebox, a 50 mL round-bottom flask equipped with a magnetic stir bar and a rubber septum was charged sequentially with $CrCl_2$ (740 mg, 6.0 mmol, 6.0 equiv.), dry THF (10 mL, 0.1 M) and TMEDA (900 μL, 6.0 mmol, 6.0 equiv.). The reaction was stirred for 20 minutes inside the glovebox before 2-(diiodomethyl)-4,4,5,5-tetramethyl-1,3,2-dioxaborolane[17] (394 mg, 1.0 mmol, 1.0 equiv.) was added in one portion. The reaction mixture was stirred for 30 minutes and then removed from the glovebox. Next, a solution of 1,3-butadiene (2.0 mL, 4.0 mmol, 4.0 equiv., 2.0 M in THF) was added. After further stirring for 18 hours at 50 °C the resulting reaction mixture was filtered through a plug of silica gel eluting with diethyl ether. The organic phase was then washed with water and brine, dried over Na_2SO_4, filtered and concentrated *in vacuo* to afford the product as a clear and colorless oil (157.2 mg, 0.81 mmol, 81%, 41:59 *cis/trans* (^{1}H NMR)). The compound showed sufficient purity at this stage and subsequent silica gel column chromatography was not required. **^{1}H NMR** (600 MHz, $CDCl_3$) δ 5.64 (dt, *J* = 16.7, 10.0 Hz, 1H, *cis*), 5.30 (dt, *J* = 16.5, 9.4 Hz, 1H, *trans*), 5.16 – 5.07 (m, 1H *cis* + 1H *trans*), 4.90 – 4.83 (m, 1H *cis* + 1H *trans*), 1.78 – 1.71 (m, 1H, *cis*), 1.63 (tt, *J* = 8.9, 5.3 Hz, 1H, *trans*), 1.24 – 1.21 (m, 12H *cis* + 12H *trans*), 1.04 – 0.99 (m, 1H, *cis*), 0.91 (td, *J* = 7.2, 3.4 Hz, 1H, *trans*), 0.76 – 0.71 (m, 1H, *cis*), 0.69 (dt, *J* = 9.2, 4.2 Hz, 1H, *trans*), 0.23 (td, *J* = 9.2, 7.3 Hz, 1H, *cis*), -0.04 (dt, *J* = 9.3, 6.0 Hz, 1H, *trans*). **^{13}C NMR** (151 MHz, $CDCl_3$) δ 142.4, 140.7, 112.9, 112.2, 83.3, 83.2, 25.2, 24.9 (2C), 24.8, 21.9, 21.6, 12.6, 12.3. **^{11}B NMR** (193 MHz, $CDCl_3$) δ 33.0. **IR** (neat, cm^{-1}): 3078, 2979, 2930, 1635, 1437, 1405, 1372, 1319, 1216, 1144, 979, 944, 895, 847, 803, 671. **HRMS** (EI): *m/z* [M]$^+$ calculated for $C_{11}H_{19}O_2{}^{11}B$: 194.1473, found 194.1477. *Note: Caution compound is volatile.*

(*Z*)-1-fluoro-4-(2-(prop-1-en-1-yl)cyclopropyl)benzene (S28)

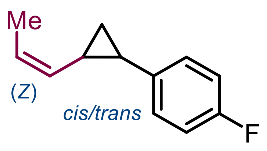

Synthesized following the General Procedure F using (*Z*)-1-bromoprop-1-ene (363 mg, 3.0 mmol) and a suspension of freshly prepared (2-(4-fluorophenyl)cyclopropyl)zinc(II) chloride[6] (3.6 mmol). Flash silica gel column chromatography (pentane) afforded the title product as a diastereomeric mixture as colorless oil (514 mg, 2.92 mmol, 97%, 65:35 *cis/trans* (^{1}H NMR)). **R_f** = 0.88 (pentane). **^{1}H NMR** (600 MHz, $CDCl_3$) δ 7.18 – 7.12 (m, 2H, *cis*), 7.07 – 7.02 (m, 2H, *trans*), 6.99 – 6.93 (m, 2H *cis* + 2H *trans*), 5.51 – 5.42 (m, 1H, *trans*), 5.44 – 5.35 (m, 1H, *cis*), 4.96 (ddt, *J* = 10.9, 9.2, 1.7 Hz, 1H, *trans*), 4.64 (ddt, *J* = 11.1, 9.4, 1.8 Hz, 1H, *cis*), 2.30 (q, *J* = 8.1 Hz, 1H, *cis*), 2.03 – 1.93 (m, 1H, *cis*), 1.87 (dt, *J* = 9.2, 4.8 Hz, 1H, *trans*), 1.79 (tt, *J* = 9.4, 5.1 Hz, 1H, *trans*), 1.73 (d, *J* = 6.8 Hz, 3H *cis* + 3H *trans*), 1.30 (tdd, *J* = 8.5, 5.1, 1.2 Hz, 1H, *cis*), 1.24 – 1.14 (m, 1H, *trans*), 1.07 – 0.97 (m, 1H, *trans*), 0.89 (q, *J* = 5.6, 5.1 Hz, 1H, *cis*). **^{13}C NMR** (151 MHz, $CDCl_3$) δ 161.4 (d, *J* = 243.6 Hz, *cis*), 161.3 (d, *J* = 243.0 Hz, *trans*), 138.4 (d, *J* = 3.1

Hz, *trans*), 134.8 (d, *J* = 3.1 Hz, *cis*), 133.0 (*trans*), 130.7 (d, *J* = 7.9 Hz, *cis*), 129.5 (*cis*), 127.3 (d, *J* = 7.8 Hz, *trans*), 124.7 (*cis*), 123.5 (*trans*), 115.2 (d, *J* = 21.2 Hz, *trans*), 114.9 (d, *J* = 21.2 Hz, *cis*), 24.5 (*trans*), 22.3 (*cis* + *trans*), 17.1 (*trans*), 17.1 (*cis*), 13.4 (*trans*), 13.3 (*cis*), 12.7 (*cis*). **^{19}F NMR** (565 MHz, $CDCl_3$) δ -117.52 (m, 1F, *cis*), -117.99 (m, 1F, *trans*). **MS** (70eV, EI): *cis and trans isomer* (GC retention time 5.972 min), *m/z* (%): 176 (38) [M$^+$], 161 (77), 147 (100), 133 (39), 122 (12), 109 (39). **HRMS** (EI): *m/z* [M]$^+$ calculated for $C_{12}H_{13}F$: 176.0996, found 176.0987. The data are in agreement with those previously reported in the literature.[6]

(*Z*)-5-(2-(prop-1-en-1-yl)cyclopropyl)benzo[*d*][1,3]dioxole (S29)

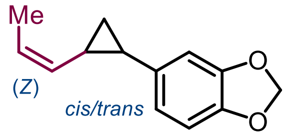

Synthesized following the General Procedure F using (*Z*)-1-bromoprop-1-ene (605 mg, 5.0 mmol, 1.0 equiv.) and a suspension of freshly prepared (2-(benzo[*d*][1,3]dioxol-5-yl)cyclopropyl)zinc(II) chloride[6] (6.0 mmol, 1.2 equiv.). Flash silica gel column chromatography (heptane) afforded the title product as a diastereomeric mixture as a colourless oil (950 mg, 4.70 mmol, 94%, 52:48 *cis*/*trans* (^{1}H NMR)). **R$_f$** = 0.19 (heptane). **^{1}H NMR** (600 MHz, $CDCl_3$) δ 6.75 – 6.70 (m, 2H, *cis* + *trans*), 6.71 – 6.65 (m, 2H, *cis*), 6.60 (dd, *J* = 8.0, 1.7 Hz, 1H, *trans*), 6.57 (d, *J* = 1.8 Hz, 1H, *trans*), 5.92 (s, 2H, *cis*), 5.91 (s, 2H, *trans*), 5.45 (dq, *J* = 10.7, 7.0 Hz, 1H, *trans*), 5.43 – 5.35 (m, 1H, *cis*), 4.94 (ddd, *J* = 11.0, 9.2, 1.9 Hz, 1H, *trans*), 4.68 (ddd, *J* = 11.2, 9.4, 1.8 Hz, 1H, *cis*), 2.27 (td, *J* = 8.5, 6.2 Hz, 1H, *cis*), 1.94 (qd, *J* = 8.9, 5.5 Hz, 1H, *cis*), 1.83 (dt, *J* = 9.3, 5.0 Hz, 1H, *trans*), 1.76 (dt, *J* = 9.4, 4.9 Hz, 1H, *trans*), 1.73 (d, *J* = 6.8 Hz, 6H, *cis* + *trans*), 1.25 (td, *J* = 8.4, 4.9 Hz, 1H, *cis*), 1.14 (dt, *J* = 8.5, 5.2 Hz, 1H, *trans*), 0.95 (dt, *J* = 8.6, 5.2 Hz, 1H, *trans*), 0.85 (q, *J* = 5.6 Hz, 1H, *cis*). **^{13}C NMR** (151 MHz, $CDCl_3$) δ 147.8, 147.4, 145.8, 145.7, 136.7, 133.1, 133.1, 129.8, 124.3, 123.3, 122.2, 119.2, 109.9, 108.2, 108.0, 106.4, 100.9, 100.9, 25.1, 22.9, 22.1, 17.1, 16.9, 13.4, 13.3, 12.7. **HRMS** (ESI): *m/z* [M]$^+$ calculated for $C_{13}H_{14}O_2$: 202.0988, found 202.0979. The data are in agreement with those previously reported in the literature.[6]

1-chloro-2-(2-(phenylethynyl)cyclopropyl)benzene

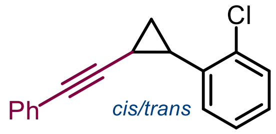

Synthesized following the General Procedure F using (bromoethynyl)benzene (218 mg, 1.2 mmol, 1.2 equiv.) and a suspension of freshly prepared (2-(2-chlorophenyl)cyclopropyl)zinc(II) chloride[6] (1.0 mmol). Flash silica gel column chromatography (hexane) afforded the title product as a diastereomeric mixture as a colorless oil (117 mg, 0.46 mmol, 46%, 62:38 *cis*/*trans* (^{1}H NMR)). **R$_f$** = 0.28 (hexane). **^{1}H NMR** (600 MHz, $CDCl_3$) δ 7.44 – 7.37 (m, 4H), 7.30 – 7.27 (m, 2H), 7.25 – 7.13 (m, 8H), 7.05 – 6.99 (m, 4H), 2.66 – 2.58 (m, 2H), 2.11 (ddd, *J* = 8.5, 5.6 Hz, 1H, *cis*), 1.71 (ddd, *J* = 8.8, 5.2 Hz, 1H, *trans*), 1.51 – 1.44 (m, 2H), 1.38 – 1.30 (m, 2H). The product was directly used in synthesis without further analysis.

(*Z*)-1-chloro-2-(2-styrylcyclopropyl)benzene (S30)

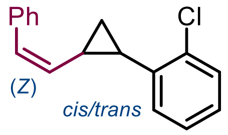

1-chloro-2-(2-(phenylethynyl)cyclopropyl)benzene (50 mg, 0.2 mmol, 1.0 equiv.) was dissolved in a 1:1 mixture of hexane/EtOAc (1 mL, 0.25 M). Next, Lindlar catalyst (10 mg, 20 mol%) and pyridine (0.016 mL, 0.2 mmol, 1.0 equiv.) were added and the flask was sealed with a septum. The flask was connected to a hydrogen balloon and the atmosphere was exchanged by bubbling hydrogen through the solution for 5 min before the reaction was stirred for 3 hours at room temperature under hydrogen. The reaction mixture was filtered over a plug of silica washing with Et_2O. The solution was then concentrated under

reduced pressure. Flash silica gel column chromatography (pentane) afforded the title product as a diastereomeric mixture as a colorless oil (36 mg, 0.14 mmol, 71%, 69:31 *cis/trans*, 93:7 *Z/E* (^{1}H NMR)). **R$_f$** = 0.26 (hexane). **^{1}H NMR** (600 MHz, $CDCl_3$) δ 7.48 (d, *J* = 7.5 Hz, 2H, *cis*), 7.41 – 7.33 (m, 3H *cis* + 2H *trans*), 7.30 (t, *J* = 7.6 Hz, 2H, *trans*), 7.25 – 7.09 (m, 4H *cis* + 4H *trans*), 6.94 (dd, *J* = 7.8, 1.0 Hz, 1H, *trans*), 6.47 (d, *J* = 11.4 Hz, 1H, *trans*), 6.31 (d, *J* = 11.6 Hz, 1H, *cis*), 5.34 (dd, *J* = 11.2, 9.9 Hz, 1H, *trans*), 4.80 (dd, *J* = 11.3, 10.1 Hz, 1H, *cis*), 2.54 (q, *J* = 8.3 Hz, 1H, *cis*), 2.47 (qd, *J* = 9.0, 6.0 Hz, 1H, *cis*), 2.37 (dt, *J* = 8.9, 5.5 Hz, 1H, *trans*), 2.10 (tt, *J* = 9.5, 5.0 Hz, 1H, *trans*), 1.42 (td, *J* = 8.3, 5.3 Hz, 1H, *cis*), 1.33 (dt, *J* = 8.6, 5.6 Hz, 1H, *trans*), 1.19 (dt, *J* = 9.0, 5.2 Hz, 1H, *trans*), 1.14 (q, *J* = 5.5 Hz, 1H, *cis*). **^{13}C NMR** (151 MHz, $CDCl_3$) δ 139.2, 137.9, 137.7, 136.8, 136.7, 135.4, 134.8, 131.3, 129.8, 129.6, 129.4, 129.4, 129.0, 128.9, 128.6, 128.3, 128.3, 127.6, 127.1, 126.9, 126.7, 126.6, 126.5, 126.4, 24.1, 23.3, 22.7, 19.2, 16.8, 13.6. **MS** (70eV, EI): *trans isomer* (GC retention time 10.358 min), *m/z* (%): 254 (13) [M$^+$], 215 (6), 202 (11), 189 (4), 176 (3), 163 (5), 130 (11), 129 (100), 91 (23); *cis isomer* (GC retention time 10.482 min), *m/z* (%): 254 (15) [M$^+$], 217 (32), 202 (32), 189 (7), 178 (4), 163 (6), 151 (2), 141 (11), 129 (100), 91 (23). **HRMS** (ESI): *m/z* [M]$^+$ calculated for $C_{17}H_{15}Cl$: 254.0857, found 254.0862.

2-(2-(4-methoxyphenyl)cyclopropyl)-4,4,5,5-tetramethyl-1,3,2-dioxaborolane

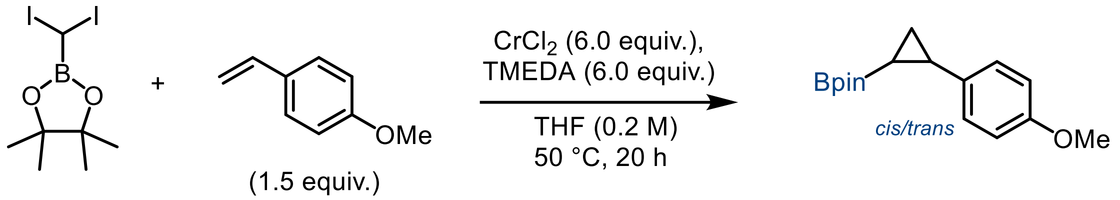

This compound was synthesized according to a modified literature procedure.[16] In an argon-filled glovebox, a 50 mL round-bottom flask equipped with a magnetic stir bar and a rubber septum was charged sequentially with $CrCl_2$ (2.62 g, 30 mmol, 6.0 equiv.), dry THF (25 mL, 0.2 M) and TMEDA (4.53 mL, 30 mmol, 6.0 equiv.). The mixture was stirred for 20 minutes inside the glovebox before 2-(diiodomethyl)-4,4,5,5-tetramethyl-1,3,2-dioxaborolane[4] (1.97 g, 5.0 mmol, 1.0 equiv.) was added in one portion. The reaction mixture was stirred for 30 minutes and then removed from the glovebox. Next, 1-methoxy-4-vinylbenzene (1.0 mL, 7.5 mmol, 1.5 equiv.) was added. The green/brown reaction mixture was then stirred for 20 hours at 50 °C. The mixture was allowed to cool to room temperature and water (50 mL) was added. Phases were separated and the aqueous phase was extracted with EtOAc (3x 50 mL). The combined organic layers were dried over Na_2SO_4, filtered and concentrated under reduced pressure. Flash silica gel column chromatography (50:1 hexane:EtOAc) afforded the title product as a diastereomeric mixture as a pale yellow oil (412 mg, 1.5 mmol, 30%, 23:77 *cis/trans* (^{1}H NMR)). **R$_f$** = 0.3 (20:1 hexane:EtOAc). **^{1}H NMR** (600 MHz, $CDCl_3$) δ 7.19 (dd, *J* = 8.6, 2.1 Hz, 2H, *cis*), 7.01 (dd, *J* = 8.5, 2.2 Hz, 2H, *trans*), 6.82 – 6.74 (m, 2H *cis* + 2H *trans*), 3.79 – 3.75 (m, 3H *cis* + 3H *trans*), 2.33 – 2.24 (m, 1H, *cis*), 2.11 – 2.02 (m, 1H, *trans*), 1.27 – 1.22 (m, 12H, *trans*), 1.22 – 1.18 (m, 1H, *cis*), 1.14 – 1.08 (m, 1H, *trans*), 1.08 – 1.05 (m, 1H, *cis*), 1.03 (s, 3H, *cis*), 0.97 – 0.91 (m, 1H, *trans*), 0.90 (s, 3H, *cis*), 0.44 – 0.34 (m, 1H, *cis*), 0.26 – 0.17 (m, 1H, *trans*). **^{13}C NMR** (151 MHz, $CDCl_3$) δ 158.0 (*cis*), 157.9 (*trans*), 135.5 (*trans*), 133.2 (*cis*), 130.0 (*cis*), 127.0 (*trans*), 113.9 (*trans*), 113.3 (*cis*), 83.3 (*trans*), 83.0 (*cis*), 55.5 (*cis*), 55.5 (*trans*), 25.0 (*cis*), 24.9 (*trans*), 24.9 (*trans*), 24.6 (*cis*), 21.4 (*trans*), 21.2 (*cis*), 14.6 (*trans*), 9.1 (*cis*). **MS** (70eV, EI): *cis isomer* (GC retention time 9.15 min), *m/z* (%): 274 (100) [M$^+$], 215 (12), 175 (44), 156 (37), 147 (39), 115 (49), 83 (34); *trans isomer* (GC retention time 9.80 min), *m/z* (%): 274

(100) [M+], 215 (11), 175 (42), 156 (23), 147 (40), 115 (44), 83 (30). The data are in agreement with those previously reported in the literature.[16]

(*Z*)-1-methoxy-4-(2-(oct-1-en-1-yl)cyclopropyl)benzene (S31)

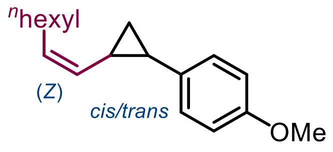

Prepared following General Procedure E using (*E*)-1-iodooct-1-ene (125 mg, 0.525 mmol). Flash silica gel column chromatography (50:1 hexane:EtOAc) afforded the title compound as a diastereomeric mixture as a colorless oil (60 mg, 0.23 mmol, 46%, 53:47 *cis/trans*, 92:8 *Z/E* (^{1}H NMR)). **R$_f$** = 0.5 (50:1 hexane:EtOAc). **^{1}H NMR** (600 MHz, $CDCl_3$) δ 7.13 – 7.09 (m, 2H, *cis*), 7.04 – 7.00 (m, 2H, *trans*), 6.84 – 6.79 (m, 2H *cis* + 2H *trans*), 5.37 (dt, *J* = 10.7, 7.4 Hz, 1H, *trans*), 5.29 (dt, *J* = 10.8, 7.4 Hz, 1H, *cis*), 4.92 (t, *J* = 10.1 Hz, 1H, *trans*), 4.62 (t, *J* = 10.3 Hz, 1H, *cis*), 3.78 (s, 3H *cis* + 3H *trans*), 2.30 – 2.23 (m, 1H, *cis*), 2.18 – 2.10 (m, 2H *cis* + 2H *trans*), 1.97 – 1.88 (m, 1H, *cis*), 1.86 – 1.80 (m, 1H, *trans*), 1.77 – 1.70 (m, 1H, *trans*), 1.41 – 1.21 (m, 9H *cis* + 8H *trans*), 1.17 – 1.11 (m, 1H, *trans*), 0.98 – 0.91 (m, 1H, *trans*), 0.92 – 0.84 (m, 4H *cis* + 3H *trans*). **^{13}C NMR** (151 MHz, $CDCl_3$) δ 157.9 (*cis*), 157.9 (*trans*), 134.8 (*trans*), 132.5 (*trans*), 131.2 (*cis*), 130.4 (*cis*), 130.3 (*cis*), 129.3 (*trans*), 129.1 (*cis*), 127.1 (*trans*), 113.9 (*trans*), 113.6 (*cis*), 55.5 (*trans*), 55.4 (*cis*), 32.0 (*cis*), 31.9 (*trans*), 29.9 (*trans*), 29.9 (*cis*), 29.1 (*cis*), 29.1 (*trans*), 27.9 (*trans*), 27.8 (*cis*), 24.6, 22.8 (*cis*), 22.8 (*trans*), 22.4 (*cis*), 22.2 (*trans*), 17.3 (*cis*), 16.9 (*trans*), 14.3 (*cis*), 14.3 (*trans*), 12.6 (*cis*). *Note*: Only *Z*-isomers were assigned. **MS** (70eV, EI): *cis isomer* (GC retention time 10.22 min), *m/z* (%): 258 (20) [M+], 173 (84), 159 (27), 147 (11), 134 (92), 121 (100); *trans isomer* (GC retention time 10.09 min), *m/z* (%): 258 (23) [M+], 173 (87), 159 (28), 147 (12), 134 (92), 121(100). The data are in agreement with those previously reported in the literature.[6]

(E)-1-methoxy-4-(2-(oct-1-en-1-yl)cyclopropyl)benzene (S32)

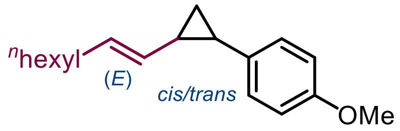

Prepared following General Procedure E using (*Z*)-1-iodooct-1-ene (125 mg, 0.525 mmol). Flash silica gel column chromatography (50:1 hexane:EtOAc) afforded the title compound as a diastereomeric mixture as a colorless oil (53 mg, 0.20 mmol, 41%, 59:41 *cis/trans*, 14:86 *Z/E* (^{1}H NMR)). **R$_f$** = 0.52 (50:1 hexane:EtOAc). **^{1}H NMR** (600 MHz, $CDCl_3$) δ 7.13 – 7.09 (m, 2H, *cis*), 7.00 – 6.98 (m, 2H, *trans*), 6.82 – 6.79 (m, 2H *cis* + 2H *trans*), 5.51 (dq, *J* = 15.2, 6.6 Hz, 1H *cis* + 1H *trans*), 5.13 (dd, *J* = 15.3, 8.4 Hz, 1H, *trans*), 4.73 (dd, *J* = 15.2, 8.9 Hz, 1H, *cis*), 3.79 (s, 3H, *cis*), 3.78 (s, 3H, *trans*), 2.20 (td, *J* = 8.6, 6.2 Hz, 1H, *cis*), 2.01 – 1.97 (m, 2H, *trans*), 1.86 (q, *J* = 7.4 Hz, 2H, *cis*), 1.80 (dt, *J* = 9.3, 5.0 Hz, 1H, *trans*), 1.74 (qd, *J* = 8.7, 5.6 Hz, 1H, *cis*), 1.57 – 1.52 (m, 1H, *trans*), 1.37 – 1.13 (m, 10H *cis* + 8H *trans*), 1.06 (dt, *J* = 8.5, 5.2 Hz, 1H, *trans*), 0.98 (dt, *J* = 8.4, 5.1 Hz, 1H, *trans*), 0.89 (t, *J* = 7.0 Hz, 3H, *trans*), 0.86 (t, *J* = 7.2 Hz, 3H, *cis*). **^{13}C NMR** (151 MHz, $CDCl_3$) δ 157.9 (*cis*), 157.8 (*trans*), 134.9 (*trans*), 132.2 (*trans*), 131.3 (*cis*), 130.8 (*cis*), 130.3 (*cis*), 129.4 (*cis*), 129.3 (*trans*), 126.8 (*trans*), 113.9 (*trans*), 113.5 (*cis*), 55.5 (*trans*), 55.4 (*cis*), 32.7 (*cis*), 32.7 (*trans*), 31.9 (*trans*), 31.9 (*cis*), 29.7 (*trans*), 29.7 (*cis*), 29.0 (*trans*), 28.8 (*cis*), 26.0 (*trans*), 24.3 (*trans*), 22.8 (*cis* + *trans*), 22.2 (*cis*), 21.6 (*cis*), 16.4 (*trans*), 14.4 (*cis*), 14.3 (*trans*), 11.8 (*cis*). *Note*: Only *E*-isomers were assigned. **MS** (70eV, EI): *cis isomer* (GC retention time 9.93 min), *m/z* (%): 258 (24) [M+], 173 (96), 159 (30), 147 (12), 134 (88), 121 (100); *trans isomer* (GC retention time 10.44 min), *m/z* (%): 258 (26) [M+], 173 (97), 159 (30), 147 (12), 134 (89), 121(100).

1-(2-(cyclohex-1-en-1-yl)cyclopropyl)-4-methoxybenzene (S33)

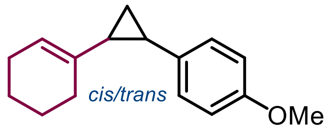

Prepared following General Procedure E using 1-iodocyclohex-1-ene (109 mg, 0.525 mmol). Flash silica gel column chromatography (30:1 hexane:EtOAc) afforded the title compound as a diastereomeric mixture as a colorless oil (60 mg, 0.23 mmol, 46%, 25:75 *cis/trans* (^{1}H NMR)). $\mathbf{R_f}$ = 0.67 (10:1 hexane:EtOAc). **^{1}H NMR** (600 MHz, $CDCl_3$) δ 7.04 – 6.97 (m, 2H *cis* + 2H *trans*), 6.81 (dd, *J* = 8.7, 2.1 Hz, 2H, *trans*), 6.77 – 6.72 (m, 2H, *cis*), 5.48 (s, 1H, *trans*), 5.46 (s, 1H, *cis*), 3.80 – 3.76 (m, 3H *cis* + 3H *trans*), 2.16 – 2.08 (m, 1H, *cis*), 2.02 – 1.98 (m, 2H, *trans*), 1.95 – 1.90 (m, 2H, *trans*), 1.90 – 1.83 (m, 1H, *trans*), 1.75 – 1.66 (m, 2H, *cis*), 1.67 – 1.60 (m, 1H *cis* + 2H *trans*), 1.61 – 1.52 (m, 1H *cis* + 2H *trans*), 1.53 – 1.46 (m, 1H, *trans*), 1.45 – 1.34 (m, 3H, *cis*), 1.28 – 1.18 (m, 2H, *cis*), 1.16 – 1.07 (m, 1H *cis* + 1H *trans*), 1.08 – 1.02 (m, 1H, *cis*), 0.93 (dt, *J* = 8.7, 5.3 Hz, 1H, *trans*). **^{13}C NMR** (151 MHz, $CDCl_3$) δ 157.8 (*cis*+*trans*), 137.5 (*trans*), 135.6 (*trans*), 134.3 (*cis*), 131.5 (*cis*), 129.0 (*cis*), 127.0 (*trans*), 123.8 (*cis*), 120.2 (*trans*), 113.9 (*trans*), 113.0 (*cis*), 55.5 (*trans*), 55.4 (*cis*), 29.7 (*trans*), 29.5 (*cis*), 27.2 (*trans*), 26.8 (*cis*), 25.4 (*cis*+*trans*), 23.1 (*trans*), 22.9 (*cis*), 22.8 (*trans*), 22.6 (*cis*), 22.6 (*trans*), 21.4 (*cis*), 13.9 (*trans*), 8.9 (*cis*). **MS** (70eV, EI): *cis isomer* (GC retention time 9.34 min), *m/z* (%): 228 (100) [M$^+$], 213 (24), 199 (24), 185 (40), 171 (51), 159 (30), 121 (94), 91 (58); *trans isomer* (GC retention time 9.95 min), *m/z* (%): 228 (100) [M$^+$], 213 (22), 199 (22), 185 (36), 171 (46), 159 (27), 121 (77), 91 (48).

1-methoxy-4-(2-(2-methylprop-1-en-1-yl)cyclopropyl)benzene (S34)

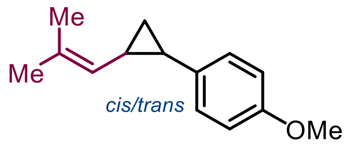

Synthesized following the General Procedure F using 1-bromo-2-methylprop-1-ene (67.5 mg, 0.5 mmol, 1.0 equiv.) and a suspension of freshly prepared (2-(4-methoxyphenyl)cyclopropyl)zinc(II) chloride[6] (0.6 mmol, 1.2 equiv.). Flash silica gel column chromatography (50:1 hexane:EtOAc) afforded the title product as a diastereomeric mixture as a colorless liquid (94 mg, 0.46 mmol, 93%, 55:45 *cis/trans* (^{1}H NMR)). $\mathbf{R_f}$ = 0.42 (50:1 hexane:EtOAc). **^{1}H NMR** (600 MHz, $CDCl_3$) δ 7.09 (d, *J* = 8.4 Hz, 2H, *cis*), 7.02 (d, *J* = 8.2 Hz, 2H, *trans*), 6.84 – 6.80 (m, 2H *cis* + 2H *trans*), 4.73 (d, *J* = 8.9 Hz, 1H, *trans*), 4.46 (d, *J* = 8.7 Hz, 1H, *cis*), 3.80 (s, 3H, *trans*), 3.78 (s, 3H, *cis*), 2.21 (td, *J* = 8.5, 6.2 Hz, 1H, *cis*), 1.88 – 1.77 (m, 1H *cis* + 1H *trans*), 1.74 – 1.70 (m, 3H *cis* + 6H *trans*), 1.64 (tt, *J* = 9.3, 5.1 Hz, 1H, *trans*), 1.57 (s, 3H, *cis*), 1.21 (td, *J* = 8.5, 4.8 Hz, 1H, *cis*), 1.10 (dt, *J* = 8.5, 5.2 Hz, 1H, *trans*), 0.77 (q, *J* = 5.6 Hz, 1H, *trans*), 0.64 – 0.59 (m, 1H, *cis*). **^{13}C NMR** (151 MHz, $CDCl_3$) δ 157.8, 157.8, 135.1, 132.5, 131.6, 131.5, 130.1, 127.5, 126.9, 123.5, 113.9, 113.5, 55.5, 55.4, 25.8, 25.7, 24.3, 22.9, 22.1, 18.5, 18.4, 18.0, 16.7, 12.5. **HRMS** (ESI): *m/z* [M]$^+$ calculated for $C_{14}H_{19}O$: 203.1430, found 203.1428. The data are in agreement with those previously reported in the literature.[6]

1-Fluoro-4-(2-(1-phenylvinyl)cyclopropyl)benzene (S35)

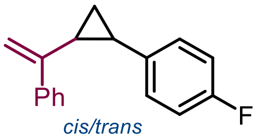

Synthesized following the General Procedure F using (1-bromovinyl)benzene (366.1 mg, 2.00 mmol, 1.0 equiv.) and a suspension of freshly prepared (2-(4-fluorophenyl)cyclopropyl)zinc(II) chloride (0.3 M, 8.6 mL, 1.3 equiv.). Flash column chromatography (pentane) afforded the title product as a colorless oil (364,6 mg, 1.53 mmol, 77%, 86:14 *cis/trans* (^{1}H NMR)). $\mathbf{R_f}$ = 0.45 (pentane). **^{1}H NMR** (600 MHz, $CDCl_3$) δ 7.44 – 7.41 (m, 2H, *trans*), 7.28 – 7.25 (m, 2H, *cis*), 7.24 – 7.09 (m, 3H, *trans* + 3H, *cis*), 7.06 – 7.01 (m, 2H, *trans*), 6.95 – 6.90 (m, 2H, *trans* + 2H, *cis*), 6.73 (dd, *J* = 8.7, 8.7 Hz, 2H, *cis*), 5.29 (m, 1H, *trans* + 1H, *cis*), 4.96 (s, 1H, *trans*), 4.84 (s, 1H, *cis*), 2.44 – 2.37 (m, 1H, *cis*), 2.18 – 2.12 (m, 1H, *cis*), 1.93 – 1.88 (m,

1H, *trans*), 1.85 – 1.80 (m, 1H, *trans*), 1.34 – 1.29 (m, 1H, *trans*), 1.29 – 1.21 (m, 2H, *cis*), 1.17 – 1.13 (m, 1H, *trans*). **^{13}C NMR** (151 MHz, $CDCl_3$) δ 161.4 (d, *J* = 243.7 Hz, *trans*), 161.3 (d, *J* = 243.3 Hz, *cis*), 148.3 (*trans*), 143.5 (*cis*), 141.5 (*cis*), 141.1 (*trans*), 138.2 (d, *J* = 2.8 Hz, *trans*), 133.8 (d, *J* = 3.0 Hz, *cis*), 129.8 (d, *J* = 7.9 Hz, *cis*), 128.4 (*trans*), 128.1 (*cis*), 127.8 (*trans*), 127.4 (*cis*), 127.3 (d, *J* = 7.9 Hz, *trans*), 126.2 (*trans*), 126.1 (*cis*), 115.4 (d, *J* = 21.4 Hz, *trans*), 114.4 (d, *J* = 21.3 Hz, *cis*), 113.2 (*cis*), 109.6 (*trans*), 27.9 (*trans*), 25.8 (*trans*), 25.5 (*cis*), 23.6 (*cis*), 15.9 (*trans*), 10.0 (*cis*). **^{19}F NMR** (565 MHz, $CDCl_3$ δ -117.65 (*trans*), -117.87 (*cis*). **HRMS** (EI): *m/z* $[M]^+$ calculated for $C_{17}H_{15}F$: 238.1158, found 238.1154.

1-Fluoro-4-(2-(prop-1-en-2-yl)cyclopropyl)benzene (S36)

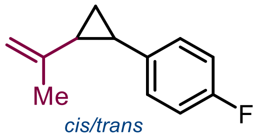

Synthesized following the General Procedure F using 2-bromoprop-1-ene (242.0 mg, 2.00 mmol, 1.0 equiv.) and a suspension of freshly prepared (2-(4-fluorophenyl)cyclopropyl)zinc(II) chloride (0.3 M, 8.6 mL, 1.3 equiv.). Flash column chromatography (pentane) afforded the title product as a colorless oil (256.3 mg, 1.45 mmol, 73%, 62:38 *cis/trans* (^{1}H NMR)). **R_f** = 0.9 (pentane). **^{1}H NMR** (600 MHz, $CDCl_3$) δ 7.09 – 7.03 (m, 2H, *cis* + 2H, *trans*), 6.95 (dd, *J* = 8.7, 8.7 Hz, 2H, *trans*), 6.90 (dd, *J* = 8.6, 8.6 Hz, 2H, *cis*), 4.76 – 4.73 (m, 1H, *cis* + 2H, *trans*), 4.66 (s, 1H, cis), 2.25 (q, *J* = 8.0 Hz, 1H, *cis*), 1.95 (dt, *J* = 9.8, 5.3 Hz, 1H, *trans*), 1.79 (q, *J* = 8.0 Hz, 1H, *cis*), 1.73 (s, 3H, *trans*), 1.64 – 1.59 (m, 1H, *trans*), 1.41 (s, 3H, *cis*), 1.20 (dt, *J* = 8.8, 5.5 Hz, 1H, *trans*), 1.17 – 1.12 (m, 2H, *cis*), 1.04 (dt, *J* = 8.7, 5.4 Hz, 1H, *trans*). **^{13}C NMR** δ 161.4 (d, *J* = 243.4 Hz, *cis*), 161.3 (d, *J* = 243.4 Hz, *trans*), 145.3 (*trans*), 142.0 (*cis*), 138.6 (d, *J* = 3.0 Hz, *trans*), 134.4 (d, *J* = 3.0 Hz, *cis*), 129.7 (d, *J* = 7.9 Hz, *cis*), 127.4 (d, *J* = 7.9 Hz, *trans*), 115.2 (d, *J* = 21.2 Hz, *cis*), 114.5 (d, *J* = 21.2 Hz, *cis*), 112.1 (*cis*), 108.8 (*trans*), 29.9 (*trans*), 26.9 (*cis*), 23.4 (*trans*), 23.2 (*cis*), 22.0 (*cis*), 20.9 (*trans*), 15.1 (*trans*), 9.6 (*cis*). **^{19}F NMR** (565 MHz, $CDCl_3$) δ -117.93 (*trans*), -117.99 (*cis*). **HRMS** (EI): *m/z* $[M]^+$ calculated for $C_{12}H_{13}F$: 176.1001, found 176.0997.

***tert*-butyl (1*R*,2*S*)-1-((*tert*-butoxycarbonyl)amino)-2-vinylcyclopropane-1-carboxylate (37)**

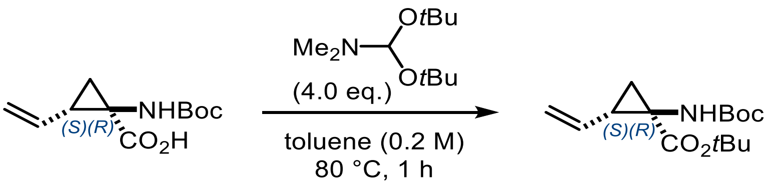

Under argon atmosphere, a solution of (1*R*,2*S*)-1-((*tert*-butoxycarbonyl)amino)-2-vinylcyclopropane-1-carboxylic acid (562 mg, 2.47 mmol, 1.0 equiv.) in dry toluene (12 mL, 0.2 M) was heated to 80 °C. Then, 1,1-di-*tert*-butoxy-*N,N*-dimethylmethanamine (2.01 g, 9.89 mmol, 4.0 equiv.) was added dropwise over the course of 15 minutes and the mixture was stirred for 1 hour at 80 °C. The mixture was cooled down to 0 °C and it was diluted with EtOAc (12 mL) before sat. $NaHCO_3$ (15 mL) was added. The phases were separated and the organic layer was washed with water (3x10 mL), dried over $MgSO_4$, filtered and concentrated under reduced pressure. Flash column chromatography (4:1 pentane:Et_2O) afforded the title product as a white solid (420 mg, 1.48 mmol, 60%, >99% ee). **R_f** = 0.37 (4:1 pentane:Et_2O, PMA). **$[\alpha]_D^{25}$** = +38.5 (*c* 1.0 $CHCl_3$, >99% ee). **M.p.** = 84.6 – 86.9 °C. **^{1}H NMR** (600 MHz, $CDCl_3$) δ 5.75 (dt, *J* = 18.0, 9.5 Hz, 1H), 5.25 (d, *J* = 17.2 Hz, 1H), 5.14 (brs, 1H), 5.09 (d, *J* = 10.3 Hz, 1H), 2.05 (q, *J* = 8.8 Hz, 1H), 1.72 (brs, 1H), 1.46 – 1.43 (m, 19H). **^{13}C NMR** (151 MHz, $CDCl_3$) δ 169.9, 155.9, 134.1, 117.4, 81.7, 80.0, 41.5, 33.6, 28.5, 28.3, 22.7. **IR** (neat, cm^{-1}): 3325, 3086, 2978, 2933, 2287, 2111, 1703, 1638, 1501, 1390,

1359, 1328, 1250, 1154, 1094, 1045, 1027, 1000, 975, 941, 906, 848, 784, 758, 694, 661. **HRMS** (ESI): *m/z* $[M+Na]^+$ calculated for $C_{15}H_{25}O_4NNa$: 306.1676, found 306.1680.

Note: The corresponding racemate (*rac*-**37**) was synthesized following the literature procedure of Beaulieu and co-workers starting from *tert*-butyl (*E*)-2-(benzylideneamino)acetate (2.1 g, 9.58 mmol).[18]

5. Isomerization and characterization data of *trans*-vinylcyclopropanes

5.1. General Procedure for vinylcyclopropane isomerization

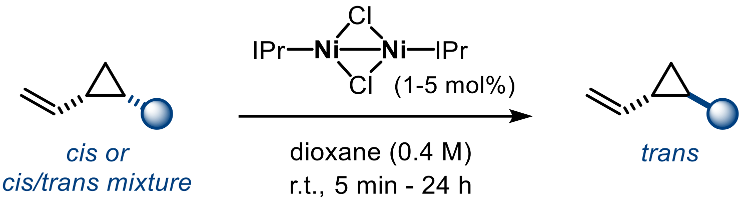

Inside an argon-filled glovebox, an oven dried 4 mL screw-cap vial equipped with a magnetic stir bar was charged with the corresponding cyclopropane substrate (1.0 equiv.), anhydrous 1,4-dioxane (0.4 M) and $[Ni(\mu\text{-}Cl)(IPr)]_2$ **1** (1-5 mol%), in that order. The reaction vial was then sealed and allowed to stir inside the glovebox at the indicated temperature for the indicated time. Upon completion the reaction mixture was removed from the glovebox and quenched by the addition of wet pentane (*i.e.* technical grade pentane that had been distilled and stored on the bench). A spatula tip of ammonium pyrrolidine-1-dithiocarboxylic acid was added and the mixture stirred for additional 15 min to precipitate nickel.[13] The mixture was filtered through a plug of silica, rinsing with Et_2O or EtOAc or MeCN and the filtrate was concentrated under reduced pressure. The *trans*-vinylcyclopropane was then obtained in high purity ranging from 90 to 99%. In case of Nickel impurities, the crude was dissolved in Et_2O and filtered another time over a plug of silica again rinsing the plug with Et_2O. The filtrate was concentrated under reduced pressure. Some compounds were further purified by silica gel column chromatography.

Note: In most cases, the colour of the reaction mixture after completion is dark red. After addition of ammonium pyrrolidine-1-dithiocarboxylic acid and subsequent mixing, the colour changes rapidly to yellow. After further mixing (15 min) the solution becomes clear and precipitation occurs.

5.2. Characterization data of the products

trans-1-methoxy-4-(2-vinylcyclopropyl)benzene (2)

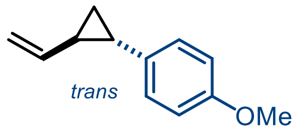

General procedure was followed using a diastereomeric mixture of vinyl cyclopropane (17.4 mg, 0.100 mmol, 99:1 *cis/trans* (^{1}H NMR)) and Nickel dimer (1.0 mg, 1 mol%). The reaction mixture was stirred for 5 min at room temperature. Purification by filtration over a short silica plug washing with Et_2O afforded the title product as a low-melting solid (17.1 mg, 0.098 mmol, 98%, 10:90 *cis/trans* (^{1}H NMR)). **R_f** = 0.41 (50:1 pentane:Et_2O). **^{1}H NMR** (600 MHz, $CDCl_3$) δ 7.01 (d, *J* = 8.7 Hz, 2H), 6.82 (d, *J* = 8.7 Hz, 2H), 5.53 (ddd, *J* = 16.8, 10.3, 8.5 Hz, 1H), 5.09 (d, *J* = 17.0 Hz, 1H), 4.92 (dd, *J* = 10.3, 1.6 Hz, 1H), 3.78 (s, 3H), 1.89 (dt, *J* = 9.6, 5.0 Hz, 1H), 1.62 (tt, *J* = 8.8, 4.9 Hz, 1H), 1.13 (dt, *J* = 8.4, 5.4 Hz, 1H), 1.05 (dt, *J* = 8.6, 5.3 Hz, 1H). **^{13}C NMR** (151 MHz, $CDCl_3$) δ 157.9, 141.0, 134.4, 127.0, 114.0, 112.4, 55.5, 27.0, 24.7, 16.4. **IR** (neat, cm^{-1}): 3074, 3001, 2927, 2837, 2325, 2067, 1997, 1878, 1797, 1680, 1633, 1611, 1513, 1459, 1376, 1295, 1245, 1177, 1112, 1076, 1034, 986, 938, 896, 825, 716. **HRMS** (EI): *m/z*: calculated for $[M]^+$ $C_{12}H_{14}O$: 174.1039, found: 174.1040. The data are in agreement with those previously reported in the literature.[19]

trans-(2-vinylcyclopropyl)benzene (4)

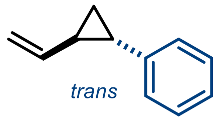

General Procedure was followed using a diastereomeric mixture of vinyl cyclopropane (14.4 mg, 0.100 mmol, 40:60 *cis/trans* (^{1}H NMR)) and Nickel dimer (1.0 mg, 1 mol%). The reaction mixture was stirred for 5 min at room temperature. Purification by filtration over a short silica plug rinsing with Et_2O afforded the title product as a colorless liquid (0.093 mmol, 93% (determined by quantitative ^{1}H NMR), 12:88 *cis/trans* (^{1}H NMR)). **R_f** = 0.43 (pentane). **^{1}H NMR** (400 MHz, $CDCl_3$) δ 7.31 – 7.25 (m, 2H), 7.21 – 7.15 (m, 1H), 7.12 – 7.07 (m, 2H), 5.56 (ddd, *J* = 16.9, 10.3, 8.5 Hz, 1H), 5.13 (dd, *J* = 17.0, 1.5 Hz, 1H), 4.96 (dd, *J* = 10.3, 1.6 Hz, 1H), 1.95 (ddd, *J* = 8.7, 5.7, 4.3 Hz, 1H), 1.77 – 1.68 (m, 1H), 1.22 (dt, *J* = 8.4, 5.4 Hz, 1H), 1.13 (dt, *J* = 8.6, 5.3 Hz, 1H). **^{13}C NMR** (151 MHz, $CDCl_3$) δ 142.5, 140.8, 128.5, 125.8, 125.8, 112.7, 27.5, 25.4, 16.9. **IR** (neat, cm^{-1}): 3068, 3026, 2926, 2737, 2340, 2062, 1946, 1727, 1681, 1633, 1604, 1495, 1452, 1372, 1331, 1295, 1217, 1179, 1121, 1074, 1030, 982, 932, 896, 841, 747, 695, 661. **HRMS** (EI): *m/z* $[M]^+$ calculated for $C_{11}H_{12}$: 144.0934, found: 144.0932. The data are in agreement with those previously reported in the literature.[20] *Note: Caution, compound is volatile.*

trans-1-(tert-butyl)-4-(2-vinylcyclopropyl)benzene (5)

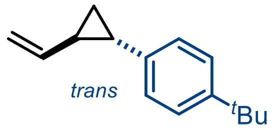

General procedure was followed using a diastereomeric mixture of vinyl cyclopropane (19.0 mg, 0.095 mmol, 27:73 *cis/trans* (^{1}H NMR)) and Nickel dimer (0.9 mg, 1 mol%). The reaction mixture was stirred for 5 min at room temperature. Purification by filtration over a short silica plug washing with Et_2O afforded the title product as a colorless liquid (0.090 mmol, 95% (determined by quantitative ^{1}H NMR), 10:90 *cis/trans* (^{1}H NMR)). **R_f** = 0.4 (pentane). **^{1}H NMR** (300 MHz, $CDCl_3$) δ 7.35 – 7.30 (m, 2H), 7.08 – 7.01 (m, 2H), 5.63 – 5.48 (m, 1H), 5.15 – 5.07 (m, 1H), 4.98 – 4.91 (m, 1H), 1.97 – 1.89 (m, 1H), 1.77 – 1.66 (m, 1H), 1.33 (s, 9H), 1.21 (dddd, *J* = 8.4, 5.7, 4.8, 0.7 Hz, 1H), 1.11 (dddd, *J* = 8.7, 5.6, 4.9, 0.6 Hz, 1H). **^{13}C NMR** (151 MHz, $CDCl_3$) δ 148.7, 140.9, 139.4, 125.5, 125.4, 112.5, 34.5, 31.5, 27.4, 25.0, 16.8. **IR** (neat, cm^{-1}): 3077, 3003, 2960, 2906, 2869, 2714, 2323, 2199, 2078, 1991, 1900, 1791, 1736, 1688, 1635, 1516, 1462, 1393, 1363, 1268, 1223, 1199, 1118, 1076, 1023, 984, 938, 895, 826, 735, 682. **HRMS** (EI): *m/z* $[M]^+$ calculated for $C_{15}H_{20}$: 200.1560, found: 200.1559. *Note: Caution, compound is volatile.*

trans-1-methyl-3-(2-vinylcyclopropyl)benzene (6)

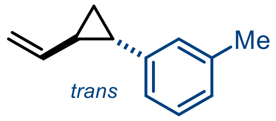

General procedure was followed using a diastereomeric mixture of vinyl cyclopropane (15.8 mg, 0.100 mmol, 33:67 *cis/trans* (^{1}H NMR)) and Nickel dimer (1.0 mg, 1 mol%). The reaction mixture was stirred for 5 min at room temperature. Purification by filtration over a short silica plug washing with Et_2O afforded the title product as a colorless liquid (0.098 mmol, 98% (determined by quantitative ^{1}H NMR), 11:89 *cis/trans* (^{1}H NMR)). **R_f** = 0.43 (pentane). **^{1}H NMR** (600 MHz, $CDCl_3$) δ 7.15 (dd, *J* = 7.5 Hz, 1H), 6.97 (d, *J* = 7.5 Hz, 1H), 6.91 – 6.85 (m, 2H), 5.57 – 5.49 (m, 1H), 5.10 (d, *J* = 17.2 Hz, 1H), 4.93 (dt, *J* = 10.3, 1.3 Hz, 1H), 2.32 (s, 3H), 1.89 (dt, *J* = 9.4, 5.0 Hz, 1H), 1.69 (tt, *J* = 8.8, 5.0 Hz, 1H), 1.19 (dt, *J* = 8.0, 5.2 Hz, 1H), 1.09 (dt, *J* = 8.2, 5.2 Hz, 1H). **^{13}C NMR** (151 MHz, $CDCl_3$) δ 142.4, 140.9, 138.0, 128.4, 126.7, 126.5, 122.8, 112.6, 27.4, 25.3, 21.6, 16.8. **IR** (neat, cm^{-1}): 3454, 3010, 2920, 2731, 2324, 2193, 2079, 1725, 1686, 1634, 1607, 1489, 1453, 1375, 1279, 1241, 1197, 1167, 1076, 1037, 985, 961, 895, 837, 776, 696, 660. **HRMS** (EI): *m/z* $[M]^+$ calculated for $C_{12}H_{14}$: 158.1090, found: 158.1088. *Note: Caution, compound is volatile.*

trans-1-(trifluoromethyl)-4-(2-vinylcyclopropyl)benzene (7)

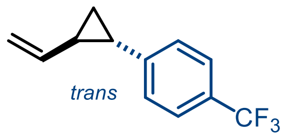

General procedure was followed using a diastereomeric mixture of vinyl cyclopropane (21.2 mg, 0.100 mmol, 35:65 *cis*/*trans* (^{1}H NMR)) and Nickel dimer (1.0 mg, 1 mol%). The reaction mixture was stirred for 5 min at room temperature. Purification by flash silica gel column chromatography (pentane) afforded the title product as a colorless liquid (0.094 mmol, 94% (determined by quantitative ^{1}H NMR), 9:91 *cis*/*trans* (^{1}H NMR)). **R$_f$** = 0.58 (hexane). **^{1}H NMR** (600 MHz, $CDCl_3$) δ 7.50 (d, *J* = 8.1 Hz, 2H), 7.15 (d, *J* = 8.1 Hz, 2H), 5.54 (ddd, *J* = 17.0, 10.3, 8.3 Hz, 1H), 5.13 (dt, *J* = 17.1, 1.4 Hz, 1H), 4.97 (dd, *J* = 10.3, 1.4 Hz, 1H), 1.97 (dt, *J* = 9.4, 4.9 Hz, 1H), 1.77 – 1.71 (m, 1H), 1.24 (dt, *J* = 8.6, 5.4 Hz, 1H), 1.19 (dt, *J* = 8.7, 5.5 Hz, 1H). **^{19}F NMR** (282 MHz, $CDCl_3$) δ -62.30 (s, 3F). **^{13}C NMR** (151 MHz, $CDCl_3$) δ 146.8, 140.0, 128.0 (q, *J* = 32.3 Hz), 126.0, 125.4 (q, *J* = 3.9 Hz), 123.6, 113.4, 28.2, 25.2, 17.4. **IR** (neat, cm^{-1}): 3081, 3010, 2684, 2327, 1697, 1618, 1521, 1454, 1418, 1323, 1226, 1162, 1115, 1067, 1015, 986, 902, 832, 735, 665. **HRMS** (EI): *m/z* [M]$^+$ calculated for $C_{12}H_{11}F_3$: 212.0807, found: 212.0808. *Note: Caution, compound is volatile.*

5-(_trans_-2-vinylcyclopropyl)benzo[_b_]thiophene (8)

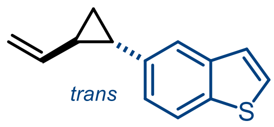

General procedure was followed using 5-(*cis*-2-vinylcyclopropyl)benzo[*b*]thiophene (40.1 mg, 0.200 mmol) and Nickel dimer (9.7 mg, 5 mol%). The reaction mixture was stirred for 60 min at room temperature. Purification by filtration over a short silica plug washing with Et_2O afforded the title product as a colorless oil (36.9 mg, 0.184 mmol, 92%, 11:89 *cis*/*trans* (^{1}H NMR)). **R$_f$** = 0.31 (100:1 pentane:Et_2O). **^{1}H NMR** (600 MHz, $CDCl_3$) δ 7.76 (d, *J* = 8.4 Hz, 1H), 7.54 (s, 1H), 7.44 – 7.39 (m, 1H), 7.28 – 7.24 (m, 1H), 7.09 (d, *J* = 8.2 Hz, 1H), 5.57 (dt, *J* = 18.3, 9.5 Hz, 1H), 5.13 (d, *J* = 17.0 Hz, 1H), 4.96 (d, *J* = 10.3 Hz, 1H), 2.09 – 2.03 (m, 1H), 1.79 – 1.71 (m, 1H), 1.30 – 1.24 (m, 1H), 1.19 – 1.12 (m, 1H). **^{13}C NMR** (151 MHz, $CDCl_3$) δ 140.8, 140.0, 138.6, 137.3, 126.9, 123.7, 123.1, 122.4, 120.6, 112.7, 27.6, 25.4, 16.8. **IR** (neat, cm^{-1}): 3075, 3003, 2326, 2167, 1883, 1728, 1685, 1633, 1604, 1505, 1432, 1374, 1324, 1262, 1227, 1196, 1157, 1084, 1045, 984, 894, 803, 751, 731, 695. **HRMS** (EI): *m/z* [M]$^+$ calculated for $C_{13}H_{12}S$: 200.0654, found 200.0654.

5-(_trans_-2-vinylcyclopropyl)benzofuran (9)

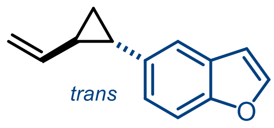

General procedure was followed using 5-(*cis*-2-vinylcyclopropyl)benzofuran (18.4 mg, 0.100 mmol) and Nickel dimer (4.8 mg, 5 mol%). The reaction mixture was stirred for 5 min at room temperature. Purification by filtration over a short silica plug washing with Et_2O afforded the title product as a yellowish oil (16.6 mg, 0.090 mmol, 90%, 10:90 *cis*/*trans* (^{1}H NMR)). **R$_f$** = 0.28 (100:1 pentane:Et_2O). **^{1}H NMR** (600 MHz, $CDCl_3$) δ 7.59 (s, 1H), 7.40 (d, *J* = 8.6 Hz, 1H), 7.31 (s, 1H), 7.04 (dd, *J* = 8.7, 2.3 Hz, 1H), 6.70 (s, 1H), 5.62 – 5.52 (m, 1H), 5.13 (dd, *J* = 17.1, 2.4 Hz, 1H), 4.95 (dd, *J* = 10.3, 2.4 Hz, 1H), 2.08 – 2.01 (m, 1H), 1.75 – 1.68 (m, 1H), 1.27 – 1.19 (m, 1H), 1.16 – 1.09 (m, 1H). **^{13}C NMR** (151 MHz, $CDCl_3$) δ 153.7, 145.4, 141.0, 136.8, 127.7, 122.8, 118.0, 112.5, 111.2, 106.5, 27.4, 25.4, 16.7. **IR** (neat, cm^{-1}): 3117, 3075, 3002, 2666, 2325, 2086, 1994, 1871, 1805, 1728, 1687, 1633, 1536, 1469, 1371, 1329, 1261, 1207, 1186, 1128, 1077, 1030, 986, 964, 896, 845, 804, 766, 735, 661. **HRMS** (EI): *m/z* [M]$^+$ calculated for $C_{13}H_{12}O$: 184.0883, found 184.0883.

(4-hydroxyphenyl)(*trans*-2-vinylcyclopropyl)methanone (10)

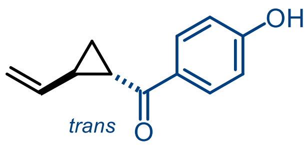

General procedure was followed using a diastereomeric mixture of vinyl cyclopropane (18.8 mg, 0.100 mmol, 41:59 *cis/trans* (^{1}H NMR)) and Nickel dimer (4.8 mg, 5 mol%). The reaction mixture was stirred for 15 min at room temperature. Purification by filtration over a short silica plug washing with EtOAc afforded the title product as a white solid (17.0 mg, 0.090 mmol, 90%, 10:90 *cis/trans* (^{1}H NMR)). **R$_f$** = 0.25 (4:1 hexane:EtOAc). **M.p.** = 78.2 – 82.3 °C. **^{1}H NMR** (600 MHz, $CDCl_3$) δ 7.94 (d, *J* = 8.4 Hz, 2H), 6.92 (d, *J* = 8.4 Hz, 2H), 5.54 (ddd, *J* = 17.0, 10.3, 8.5 Hz, 1H), 5.20 (d, *J* = 17.0 Hz, 1H), 5.03 (d, *J* = 10.3 Hz, 1H), 2.66 (dt, *J* = 8.5, 4.4 Hz, 1H), 2.22 – 2.16 (m, 1H), 1.70 (dt, *J* = 9.0, 4.6 Hz, 1H), 1.18 (ddd, *J* = 7.9, 6.4, 4.0 Hz, 1H). **^{13}C NMR** (151 MHz, $CDCl_3$) δ 198.4, 160.8, 138.6, 130.9, 130.7, 115.6, 115.2, 29.5, 26.5, 18.2. **IR** (neat, cm^{-1}): 3338, 3014, 2466, 1834, 1638, 1603, 1573, 1515, 1442, 1388, 1316, 1283, 1224, 1165, 1115, 1075, 1051, 1024, 990, 911, 848, 812, 748, 688, 665. **HRMS** (ESI): *m/z* [M+Na]$^+$ calculated for $C_{12}H_{12}O_2Na$: 211.0730, found: 211.0727.

(4-aminophenyl)(*trans*-2-vinylcyclopropyl)methanone (11)

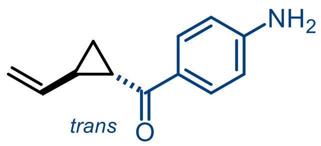

General procedure was followed using a diastereomeric mixture of vinyl cyclopropane (18.7 mg, 0.100 mmol, 37:63 *cis/trans* (^{1}H NMR)) and Nickel dimer (4.8 mg, 5 mol%). The reaction mixture was stirred for 15 min at room temperature. Purification by filtration over a short silica plug washing with EtOAc afforded the title product as a beige solid (17.0 mg, 0.098 mmol, 91%, 7:93 *cis/trans* (^{1}H NMR)). **R$_f$** = 0.2 (2:1 hexane:EtOAc). **M.p.** = 106.4 – 109.8 °C. **^{1}H NMR** (400 MHz, $CDCl_3$) δ 7.86 (d, *J* = 8.6 Hz, 2H), 6.66 (d, *J* = 8.5 Hz, 2H), 5.52 (ddd, *J* = 17.2, 10.2, 8.6 Hz, 1H), 5.18 (dd, *J* = 17.1, 1.4 Hz, 1H), 5.00 (dd, *J* = 10.3, 1.4 Hz, 1H), 4.17 – 4.12 (m, 2H), 2.60 (ddd, *J* = 8.5, 5.1, 3.9 Hz, 1H), 2.13 (tdd, *J* = 8.8, 6.0, 3.8 Hz, 1H), 1.63 (ddd, *J* = 8.8, 5.1, 3.9 Hz, 1H), 1.10 (ddd, *J* = 8.1, 6.3, 3.8 Hz, 1H). **^{13}C NMR** (101 MHz, $CDCl_3$) δ 196.6, 151.1, 139.1, 130.7, 130.6, 128.4, 114.7, 113.9, 113.9, 28.7, 25.9, 17.7. **IR** (neat, cm^{-1}): 3405, 3330, 3221, 2975, 2686, 2475, 2323, 1743, 1633, 1587, 1515, 1441, 1384, 1305, 1235, 1170, 1081, 1049, 1021, 912, 843, 749, 695, 668. **HRMS** (ESI): *m/z* [M+Na]$^+$ calculated for $C_{12}H_{13}ONNa$: 210.0889, found: 210.0888.

1-(*trans*-2-vinylcyclopropyl)nonan-1-one (12)

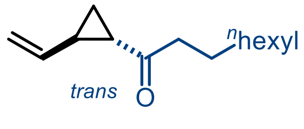

General procedure was followed using a diastereomeric mixture of 1-(2-vinylcyclopropyl)nonan-1-one (20.8 mg, 0.100 mmol, 40:60 *cis/trans* (^{1}H NMR)) and Nickel dimer (4.8 mg, 5 mol%). The reaction mixture was stirred for 15 min at room temperature. Purification by filtration over a short silica plug washing with Et_2O afforded the title product as a colorless oil (20.0 mg, 0.096 mmol, 96%, 9:91 *cis/trans* (^{1}H NMR)). **R$_f$** = 0.25 (50:1 pentane:Et_2O). **^{1}H NMR** (600 MHz, $CDCl_3$) δ 5.40 (ddd, *J* = 17.0, 10.3, 8.5 Hz, 1H), 5.14 (dd, *J* = 17.1, 1.4 Hz, 1H), 4.97 (dd, *J* = 10.3, 1.4 Hz, 1H), 2.54 (td, *J* = 7.3, 1.9 Hz, 2H), 2.03 – 1.92 (m, 2H), 1.63 – 1.56 (m, 2H), 1.43 (ddd, *J* = 8.9, 5.2, 3.9 Hz, 1H), 1.31 – 1.23 (m, 10H), 0.98 (ddd, *J* = 8.1, 6.3, 4.0 Hz, 1H), 0.87 (t, *J* = 7.0 Hz, 3H). **^{13}C NMR** (151 MHz, $CDCl_3$) δ 209.5, 138.7, 114.7, 44.1, 32.0, 29.6, 29.5, 29.4, 29.3, 28.4, 24.2, 22.8, 17.6, 14.2. **IR** (neat, cm^{-1}): 3004, 2925, 2856, 2324, 2159, 1697, 1638, 1458, 1385, 1305, 1201, 1129, 1083, 987, 902, 838, 722. **HRMS** (ESI): *m/z* [M+Na]$^+$ calculated for $C_{14}H_{24}ONa$: 231.1719, found: 231.1716.

Naphthalen-1-yl *trans*-2-vinylcyclopropane-1-carboxylate (13)

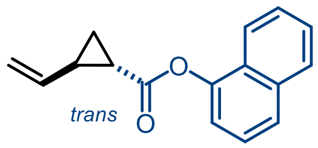

General procedure was followed using a diastereomeric mixture of vinyl cyclopropane (12.5 mg, 0.052 mmol, 37:63 *cis/trans* (^{1}H NMR)) and Nickel dimer (2.5 mg, 5 mol%). The reaction mixture was stirred for 1 h at room temperature. Purification by filtration over a short silica plug washing with Et_2O afforded the title product as a colorless oil (12.0 mg, 0.052 mmol, 96%, 9:91 *cis/trans* (^{1}H NMR)). **R$_f$** = 0.33 (20:1 pentane:Et_2O). **^{1}H NMR** (400 MHz, $CDCl_3$) δ 7.94 – 7.84 (m, 2H), 7.75 (d, *J* = 8.2 Hz, 1H), 7.55 – 7.51 (m, 1H), 7.47 (dd, *J* = 7.9 Hz, 1H), 7.27 (d, *J* = 7.6 Hz, 1H), 5.55 (ddd, *J* = 17.0, 10.2, 8.3 Hz, 1H), 5.31 (dd, *J* = 16.9, 1.3 Hz, 1H), 5.12 (dd, *J* = 10.3, 1.3 Hz, 1H), 2.31 (qd, *J* = 8.8, 3.9 Hz, 1H), 2.08 (ddd, *J* = 8.7, 5.1, 4.0 Hz, 1H), 1.65 (dt, *J* = 9.2, 4.8 Hz, 1H), 1.24 (ddd, *J* = 8.3, 6.4, 4.5 Hz, 1H). **^{13}C NMR** (101 MHz, $CDCl_3$) δ 172.2, 146.7, 137.8, 134.7, 128.2, 126.9, 126.6, 126.5, 126.1, 125.5, 121.3, 118.2, 115.6, 26.7, 21.9, 16.5. **IR** (neat, cm^{-1}): 3062, 3011, 2325, 2092, 1745, 1637, 1598, 1508, 1445, 1379, 1319, 1222, 1132, 1087, 1046, 987, 909, 872, 845, 792, 770, 729. **HRMS** (ESI): *m/z* $[M+Na]^+$ calculated for $C_{16}H_{14}O_2Na$: 261.0886, found: 261.0884.

4-oxo-2-phenyl-4H-chromen-3-yl *trans*-2-vinylcyclopropane-1-carboxylate (14)

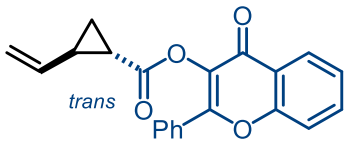

General procedure was followed using a diastereomeric mixture of vinyl cyclopropane (33.2 mg, 0.100 mmol, 41:59 *cis/trans* (^{1}H NMR)) and Nickel dimer (4.8 mg, 5 mol%). The reaction mixture was stirred for 1 h at room temperature. Purification by filtration (2 times) over a short silica plug washing with EtOAc afforded the title product as a white solid (32.0 mg, 0.096 mmol, 96%, 8:92 *cis/trans* (^{1}H NMR)). **R$_f$** = 0.28 (5:1 hexane:EtOAc). **M.p.** = 103.2 – 107.8 °C. **^{1}H NMR** (600 MHz, $CDCl_3$) δ 8.27 (dd, *J* = 8.0, 1.7 Hz, 1H), 7.87 (dd, *J* = 7.6, 2.0 Hz, 2H), 7.74 – 7.68 (m, 1H), 7.58 – 7.51 (m, 4H), 7.44 (dd, *J* = 7.5 Hz, 1H), 5.47 (ddd, *J* = 17.0, 10.2, 8.3 Hz, 1H), 5.22 (d, *J* = 17.0 Hz, 1H), 5.06 (d, *J* = 10.2 Hz, 1H), 2.22 – 2.16 (m, 1H), 1.96 (dt, *J* = 8.7, 4.5 Hz, 1H), 1.56 (dt, *J* = 9.4, 4.9 Hz, 1H), 1.16 (ddd, *J* = 8.3, 6.4, 4.6 Hz, 1H). **^{13}C NMR** (151 MHz, $CDCl_3$) δ 172.4, 170.7, 156.4, 155.8, 137.6, 134.1, 133.7, 131.4, 130.2, 128.8, 128.5, 126.3, 125.3, 123.8, 118.2, 115.6, 26.7, 21.8, 16.4. **IR** (neat, cm^{-1}): 3286, 3074, 2924, 2854, 2186, 1752, 1642, 1612, 1568, 1468, 1386, 1286, 1239, 1188, 1118, 1031, 990, 899, 847, 756, 693. **HRMS** (CI): *m/z* $[M+H]^+$ calculated for $C_{21}H_{17}O_4$: 333.1121, found: 333.1120.

Octyl *trans*-2-vinylcyclopropane-1-carboxylate (15)

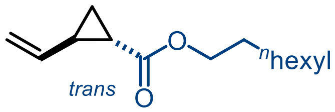

General procedure was followed using a diastereomeric mixture of vinyl cyclopropane (9.0 mg, 0.041 mmol, 50:50 *cis/trans* (^{1}H NMR)) and Nickel dimer (1.9 mg, 5 mol%). The reaction mixture was stirred for 1 h at room temperature. Purification by filtration over a short silica plug washing with Et_2O afforded the title product as a colorless oil (8.5 mg, 0.040 mmol, 94%, 9:91 *cis/trans* (^{1}H NMR)). **R$_f$** = 0.31 (30:1, pentane:Et_2O, $KMnO_4$). **^{1}H NMR** (600 MHz, $CDCl_3$) δ 5.40 (ddd, *J* = 17.0, 10.3, 8.4 Hz, 1H), 5.16 (dd, *J* = 17.0, 1.4 Hz, 1H), 4.99 (dd, *J* = 10.3, 1.4 Hz, 1H), 4.06 (td, *J* = 6.8, 2.3 Hz, 2H), 2.05 – 1.98 (m, 1H), 1.66 – 1.59 (m, 3H), 1.39 – 1.23 (m, 11H), 0.97 (ddd, *J* = 8.4, 6.3, 4.3 Hz, 1H), 0.88 (t, *J* = 6.9 Hz, 3H). **^{13}C NMR** (151 MHz, $CDCl_3$) δ 173.6, 138.3, 114.9, 65.0, 31.9, 29.4, 29.3, 28.8, 26.1, 25.7, 22.8, 22.0, 15.7, 14.2. **IR** (neat, cm^{-1}): 2926, 2857, 2327, 2111, 1726, 1639, 1459, 1400, 1373, 1266, 1169, 1086, 1048, 987, 905, 853, 818, 731. **HRMS** (ESI): *m/z* $[M+Na]^+$ calculated for $C_{14}H_{24}O_2Na$: 247.1669, found: 247.1664.

trans-2-vinylcyclopropane-1-carboxylic acid (17)

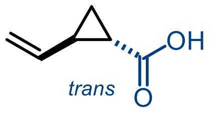

General procedure was followed using a diastereomeric mixture of vinyl cyclopropane (22.5 mg, 0.200 mmol, 44:56 *cis/trans* (^{1}H NMR)) and Nickel dimer (9.7 mg, 5 mol%). The reaction mixture was stirred for 15 min at room temperature. Purification by filtration over a short silica plug washing with Et_2O afforded the title product as a colorless oil (21.0 mg, 0.187 mmol, 94%, 25:75 *cis/trans* (^{1}H NMR)). **R_f** = 0.37 (10:1 pentane:Et_2O, 1% AcOH, $KMnO_4$). **^{1}H NMR** (600 MHz, $CDCl_3$) δ 5.40 (ddd, *J* = 17.0, 10.2, 8.3 Hz, 1H), 5.18 (d, *J* = 17.0 Hz, 1H), 5.02 (dd, *J* = 10.3, 1.3 Hz, 1H), 2.12 – 2.06 (m, 1H), 1.68 – 1.61 (m, 1H), 1.43 (dt, *J* = 9.2, 4.7 Hz, 1H), 1.06 (ddd, *J* = 8.3, 6.4, 4.4 Hz, 1H). *Note*: The acid proton was not observed in ^{1}H NMR due to H-D exchange. **^{13}C NMR** (151 MHz, $CDCl_3$) δ 179.7, 137.7, 115.4, 26.6, 21.8, 16.3. **IR** (neat, cm^{-1}): 2923, 2643, 2569, 1690, 1641, 1433, 1360, 1292, 1227, 1084, 1052, 980, 905, 851, 678. **HRMS** (EI): *m/z* [M]$^+$ calculated for $C_6H_8O_2$: 112.0519, found: 112.0504. The data are in agreement with those previously reported in the literature.[21] *Note: Caution, compound is volatile.*

Ethyl _trans_-2,2-dimethyl-3-vinylcyclopropane-1-carboxylate (18)

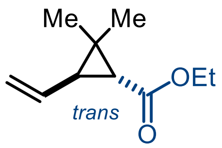

General procedure was followed using ethyl *cis*-2,2-dimethyl-3-vinylcyclopropane-1-carboxylate (16.8 mg, 0.100 mmol, 1.0 equiv., 8:92 *trans/cis* (^{1}H NMR)) and Nickel dimer (5 mol%). The reaction mixture was stirred for 24 h at at 60 °C. Purification by filtration over a short silica plug washing with Et_2O afforded the title product as a colorless oil (16.2 mg, 0.096 mmol, 96%, 92:8 *trans/cis* (^{1}H NMR)). **^{1}H NMR** (600 MHz, $CDCl_3$) δ 5.57 (ddd, *J* = 17.0, 10.3, 8.4 Hz, 1H), 5.18 (dd, *J* = 17.1, 1.8 Hz, 1H), 5.09 – 5.04 (m, 1H), 4.16 – 4.08 (m, 2H), 2.05 (dd, *J* = 8.4, 5.4 Hz, 1H), 1.56 (d, *J* = 5.4 Hz, 1H), 1.30 – 1.24 (m, 6H), 1.15 (s, 3H). **^{13}C NMR** (151 MHz, $CDCl_3$) δ 172.1, 135.5, 116.5, 60.5, 36.8, 34.0, 28.8, 22.0, 20.4, 14.5. **IR** (neat, cm^{-1}): 2952 (s), 2326 (w), 1723 (s), 1635 (m), 1384 (m), 1330 (m), 1179 (s), 1113 (s), 904 (m), 836 (m), 674 (w). **HRMS** (EI): *m/z* [M+Na]$^+$ calculated for $C_{10}H_{16}O_2Na$: 191.1048, found 191.1040.

Ethyl _trans_-2-methyl-2-(prop-1-en-2-yl)cyclopropane-1-carboxylate (19)

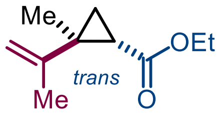

General procedure was followed using ethyl *cis*-2-methyl-2-(prop-1-en-2-yl)cyclopropane-1-carboxylate (16.8 mg, 0.100 mmol, 1.0 equiv., 8:92 *trans/cis* (^{1}H NMR)) in diethyl ether. The reaction mixture was stirred for 18 h at room temperature. Purification by filtration over a short silica plug washing with Et_2O afforded the title product as a colorless oil (16.0 mg, 0.095 mmol, 95%, 73:27 *trans/cis* (^{1}H NMR)). **^{1}H NMR** (600 MHz, $CDCl_3$) δ 4.91 – 4.87 (m, 1H *cis*), 4.90 – 4.86 (m, 1H *cis*), 4.83 – 4.80 (m, 1H *trans*), 4.78 – 4.74 (m, 1H *trans*), 4.19 – 4.09 (m, 2H *trans*), 4.08 (qd, *J* = 7.1, 1.0 Hz, 1H *cis*), 1.74 (s, 3H *trans*), 1.71 (dd, *J* = 7.5, 5.2 Hz, 1H *trans*), 1.70 (s, 3H *cis*), 1.63 (dd, *J* = 7.8, 5.4 Hz, 1H *cis*), 1.51 (t, *J* = 5.0 Hz, 1H *cis*), 1.30 (s, 3H *trans*), 1.29 – 1.24 (m, 3H *trans* + 3H *cis*), 1.22 (t, *J* = 7.1 Hz, 3H *cis*), 1.22 – 1.15 (m, 2H *trans*), 0.94 (dd, *J* = 7.8, 4.4 Hz, 1H *cis*). **^{13}C NMR** (151 MHz, $CDCl_3$) δ ^{13}C NMR (151 MHz, $CDCl_3$) δ 172.4, 171.8, 148.5, 144.4, 113.6, 110.9, 60.5, 60.3, 33.6, 31.9, 27.9, 26.4, 25.4, 20.8, 20.5, 20.2, 20.0, 17.0, 14.5, 14.4. **IR** (neat, cm^{-1}): 2920 (s), 2255 (w), 1728 (s), 1645 (m), 1382 (s), 1330 (m), 1161 (s), 1028 (s), 910 (m), 732 (s), 648 (w). **HRMS** (EI): *m/z* [M]$^+$ calculated for $C_{10}H_{16}O_2$: 168.1150, found 168.1150.

trans-1-(5-methoxynonan-5-yl)-2-vinylcyclopropane (20)

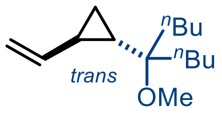

General procedure was followed using a diastereomeric mixture of vinyl cyclopropane (49.8 mg, 90w%, 0.200 mmol, 30:70 cis/trans (^{1}H NMR)) and Nickel dimer (9.65 mg, 5 mol%). The reaction mixture was stirred for 24 h at 60 °C. Flash silica gel column chromatography (100:1 hexane:EtOAc) afforded the title compound as a colorless oil (48 mg, 90w%, 0.192 mmol, 96%, 1:99 *cis/trans* (^{1}H NMR)). **R$_f$** = 0.18 (100:1 hexane:EtOAc, PMA). **^{1}H NMR** (600 MHz, $CDCl_3$) δ 5.37 (ddd, *J* = 17.1, 10.2, 8.7 Hz, 1H), 5.05 (dd, *J* = 17.0, 1.7 Hz, 1H), 4.86 (dd, *J* = 10.2, 1.8 Hz, 1H), 3.14 (s, 3H), 1.48 – 1.37 (m, 4H), 1.34 – 1.25 (m, 9H), 0.93 – 0.89 (m, 6H), 0.84 – 0.74 (m, 2H), 0.52 (ddd, *J* = 8.8, 5.1, 4.1 Hz, 1H). **^{13}C NMR** (151 MHz, $CDCl_3$): δ 142.0, 112.0, 76.5, 49.1, 35.6, 34.8, 27.7, 25.8, 25.6, 23.6, 23.6, 18.1, 14.3, 9.9. **IR** (neat, cm^{-1}): 3183, 3148, 3074, 2957, 2868, 2292, 2190, 2066, 2033, 1934, 1796, 1702, 1635, 1590, 1535, 1461, 1399, 1380, 1329, 1268, 1201, 1176, 1081, 986, 936, 890, 802, 738, 708, 659. **HRMS** (ESI): *m/z* $[M+Na]^+$ calculated for $C_{15}H_{28}ONa$: 247.2032, found 247.2021.

trans-N,N-diethyl-2-vinylcyclopropane-1-carboxamide (21)

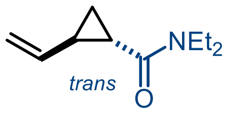

General procedure was followed using a diastereomeric mixture of vinyl cyclopropane (33.5 mg, 0.200 mmol, 44:56 *cis/trans* (^{1}H NMR)) and Nickel dimer (9.7 mg, 5 mol%). The reaction mixture was stirred for 2 h at room temperature. Purification by filtration over a short silica plug washing with Et_2O afforded the title product as a colorless oil (31.1 mg, 0.186 mmol, 93%, 10:90 *cis/trans* (^{1}H NMR)). **R$_f$** = 0.38 (3:2 hexane:EtOAc, PMA). **^{1}H NMR** (600 MHz, C_6D_6) δ 5.23 (ddt, *J* = 17.0, 10.3, 8.7 Hz, 1H), 5.03 (dd, *J* = 17.1, 1.7 Hz, 1H), 4.87 (dd, *J* = 10.2, 1.6 Hz, 1H), 3.26 (dq, *J* = 14.1, 7.1 Hz, 1H), 3.18 (dq, *J* = 14.0, 7.1 Hz, 1H), 2.85 (q, *J* = 7.1 Hz, 2H), 2.20 (tdd, *J* = 8.8, 5.9, 3.9 Hz, 1H), 1.67 (ddd, *J* = 8.7, 5.2, 3.7 Hz, 1H), 1.45 (ddd, *J* = 8.1, 5.2, 4.0 Hz, 1H), 0.94 (t, *J* = 7.1 Hz, 3H), 0.77 (t, *J* = 7.2 Hz, 3H), 0.65 (ddd, *J* = 8.1, 5.9, 3.7 Hz, 1H). **^{13}C NMR** (151 MHz, C_6D_6) δ 170.2, 139.7, 113.9, 41.9, 41.1, 25.2, 20.9, 15.0, 13.6. **IR** (neat, cm^{-1}): 3479, 3255, 3081, 2975, 2933, 2320, 2158, 1908, 1737, 1629, 1456, 1430, 1374, 1310, 1254, 1221, 1139, 1082, 969, 898, 851, 789, 729, 664. **HRMS** (ESI): *m/z* $[M+Na]^+$ calculated for $C_{10}H_{17}NONa$: 190.1202, found: 190.1209.

trans-N-([1,1'-biphenyl]-4-yl)-trans-2-vinylcyclopropane-1-carboxamide (22)

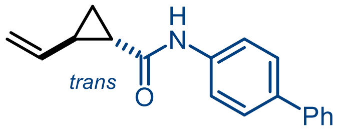

General procedure was followed using a diastereomeric mixture of vinyl cyclopropane (11.0 mg, 0.042 mmol, 39:61 *cis/trans* (^{1}H NMR)) and Nickel dimer (2.0 mg, 5 mol%). The reaction mixture was stirred for 1 h at room temperature. Purification by filtration over a short silica plug washing with Et_2O afforded the title product as a white solid (10.6 mg, 0.040 mmol, 96%, 7:93 *cis/trans* (^{1}H NMR)). **R$_f$** = 0.32 (4:1 hexane:EtOAc). **M.p.** = 171.7 – 174.6 °C. **^{1}H NMR** (600 MHz, $CDCl_3$) δ 7.61 – 7.52 (m, 6H), 7.51 (s, 1H), 7.42 (dd, *J* = 7.7 Hz, 2H), 7.33 (dd, *J* = 7.6 Hz, 1H), 5.46 (dt, *J* = 18.1, 9.6 Hz, 1H), 5.20 (d, *J* = 17.0 Hz, 1H), 5.02 (d, *J* = 10.0 Hz, 1H), 2.13 (dq, *J* = 10.5, 5.3 Hz, 1H), 1.58 – 1.50 (m, 2H), 1.01 (td, *J* = 7.1, 3.8 Hz, 1H). **^{13}C NMR** (151 MHz, $CDCl_3$) δ 170.4, 140.6, 138.5, 137.4, 137.1, 128.9, 127.7, 127.2, 127.0, 120.1, 114.9, 25.5, 25.1, 15.4. **IR** (neat, cm^{-1}): 3292, 3035, 2329, 1903, 1812, 1650, 1596, 1532, 1486, 1448, 1401, 1316, 1253, 1203, 1184, 1112, 1077, 1039, 1004, 983, 899, 833, 758, 685. **HRMS** (ESI): *m/z* $[M+Na]^+$ calculated for $C_{18}H_{17}NONa$: 286.1202, found: 286.1204.

trans-_N_-methoxy-_N_-methyl-2-vinylcyclopropane-1-carboxamide (23)

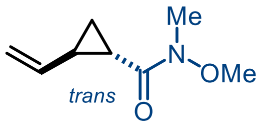

General procedure was followed using a diastereomeric mixture of vinyl cyclopropane (15.5 mg, 0.100 mmol, 42:58 *cis/trans* (^{1}H NMR)) and Nickel dimer (4.8 mg, 5 mol%). The reaction mixture was stirred for 1 h at room temperature. Purification by filtration over a short silica plug washing with Et_2O afforded the title product as a colorless oil (15.0 mg, 0.096 mmol, 97%, 9:91 *cis/trans* (^{1}H NMR)). **R$_f$** = 0.47 (3:2 pentane:Et_2O, $KMnO_4$). **^{1}H NMR** (400 MHz, $CDCl_3$) δ 5.45 (ddd, *J* = 17.0, 10.2, 8.5 Hz, 1H), 5.16 (ddd, *J* = 17.0, 1.6, 0.7 Hz, 1H), 4.97 (dd, *J* = 10.2, 1.2 Hz, 1H), 3.73 (s, 3H), 3.21 (s, 3H), 2.20 – 2.12 (m, 1H), 1.98 (tdd, *J* = 8.6, 6.1, 4.0 Hz, 1H), 1.41 (ddd, *J* = 8.9, 5.3, 4.1 Hz, 1H), 0.96 (ddd, *J* = 8.3, 6.1, 4.1 Hz, 1H). **^{13}C NMR** (151 MHz, $CDCl_3$) δ 138.9, 114.5, 61.8, 32.7, 25.5, 19.3, 15.2. *Note:* The carbonyl carbon was not observed in ^{13}C NMR. **IR** (neat, cm^{-1}): 3083, 3003, 2967, 2937, 2326, 2094, 1651, 1421, 1390, 1335, 1175, 1104, 1048, 1004, 964, 902, 845, 764, 720, 664. **HRMS** (ESI): *m/z* [M+Na]$^+$ calculated for $C_8H_{13}O_2NNa$: 188.0839, found: 188.0843.

(_trans_-2-vinylcyclopropyl)triethylgermane (24)

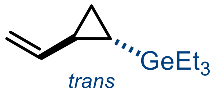

General procedure was followed using (*cis*-2-vinylcyclopropyl)triethylgermane (22.7 mg, 0.100 mmol) and Nickel dimer (4.8 mg, 5 mol%).The reaction mixture was stirred for 24 h at room temperature. Purification by flash silica gel column chromatography (pentane) afforded the title product as a colorless oil (21.8 mg, 0.096 mmol, 96%, 10:90 *cis/trans* (^{1}H NMR)). **R$_f$** = 0.73 (hexane, $KMnO_4$). **^{1}H NMR** (600 MHz, $CDCl_3$) δ 5.32 (ddd, *J* = 17.0, 10.2, 8.8 Hz, 1H), 5.05 (dd, *J* = 17.0, 1.7 Hz, 1H), 4.81 (dd, *J* = 10.2, 1.8 Hz, 1H), 1.35 – 1.29 (m, 1H), 1.05 – 1.00 (m, 9H+1H), 0.71 – 0.64 (m, 6H+1H), -0.07 (ddd, *J* = 9.7, 7.6, 5.8 Hz, 1H). **^{13}C NMR** (151 MHz, $CDCl_3$) δ 143.9, 110.7, 18.6, 10.5, 8.9, 4.9, 3.5. **IR** (neat, cm^{-1}): 2947, 2870, 2826, 2730, 2326, 2094, 1990, 1744, 1457, 1426, 1375, 1215, 1011, 961, 692. **HRMS** (CI): *m/z* [M-C_2H_5]$^+$ calculated for $C_9H_{17}Ge$: 199.0537, found 199.0536. *Note: Attention, compound is volatile.*

trans-6-methyl-2-(_trans_-2-vinylcyclopropyl)-1,3,6,2-dioxazaborocane-4,8-dione (25)

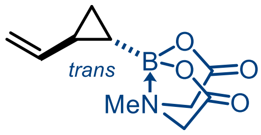

General procedure was followed using a diastereomeric mixture of vinyl cyclopropane (22.3 mg, 0.100 mmol, 35:65 *cis/trans* (^{1}H NMR)) and Nickel dimer (4.8 mg, 5 mol%). The reaction mixture was stirred for 72 h at 60 °C. Purification by flash silica gel column chromatography (20:1 DCM:MeOH) afforded the title product as a white solid (21.2 mg, 0.095 mmol, 95%, 12:88 *cis/trans* (^{1}H NMR)). **R$_f$** = 0.17 (20:1 DCM:MeOH, PMA). **M.p.** = 90.2 – 96.7 °C. **^{1}H NMR** (600 MHz, CD_3CN) δ 5.37 (dt, *J* = 18.2, 9.6 Hz, 1H), 5.10 (d, *J* = 17.0 Hz, 1H), 4.82 (d, *J* = 10.2 Hz, 1H), 3.94 (d, *J* = 17.1 Hz, 2H), 3.81 (d, *J* = 17.1 Hz, 2H), 2.94 (s, 3H), 1.33 – 1.26 (m, 1H), 0.65 – 0.58 (m, 2H), -0.12 – -0.19 (m, 1H). **^{11}B NMR** (193 MHz, CD_3CN) δ 12.4. **^{13}C NMR** (151 MHz, CD_3CN) δ 169.2, 169.1, 144.0, 111.9, 63.0, 62.9, 47.4, 19.5, 10.7. *Note:* The carbon attached to boron was not observed in ^{13}C NMR. **IR** (neat, cm^{-1}): 3502, 3073, 3002, 2960, 2920, 2851, 2164, 1744, 1634, 1540, 1456, 1337, 1290, 1248, 1194, 1123, 1075, 991, 961, 891, 861, 709, 663. **HRMS** (ESI): *m/z* [M+Na]$^+$ calculated for $C_{10}H_{14}O_4NBNa$: 246.0908, found: 246.0906.

trans-4,4,5,5-tetramethyl-2-(_trans_-2-vinylcyclopropyl)-1,3,2-dioxaborolane (26)

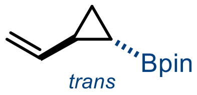

General procedure was followed using a diastereomeric mixture of vinyl cyclopropane (38.8 mg, 0.200 mmol, 41:59 *cis*/*trans* (^{1}H NMR)) and Nickel dimer (9.7 mg, 5 mol%). The reaction mixture was stirred for 72 h at 60 °C. Purification by flash silica gel column chromatography (50:1 pentane:Et_2O) afforded the title product as a colorless oil (31.8 mg, 0.164 mmol, 82%, 21:79 *cis*/*trans* (^{1}H NMR)). **R$_f$** = 0.14 (50:1 pentane:Et_2O, PMA). **^{1}H NMR** (600 MHz, $CDCl_3$) δ 5.31 (ddd, *J* = 17.0, 10.2, 8.8 Hz, 1H), 5.10 (dd, *J* = 17.0, 1.7 Hz, 1H), 4.86 (dd, *J* = 10.2, 1.7 Hz, 1H), 1.64 (tt, *J* = 8.3, 5.1 Hz, 1H), 1.24 – 1.20 (m, 12H), 0.92 (ddd, *J* = 7.9, 6.7, 3.6 Hz, 1H), 0.69 (ddd, *J* = 9.7, 5.0, 3.5 Hz, 1H), -0.04 (ddd, *J* = 9.7, 6.7, 5.2 Hz, 1H). **^{11}B NMR** (193 MHz, $CDCl_3$) δ 32.9. **^{13}C NMR** (151 MHz, $CDCl_3$) δ 142.4, 112.2, 83.2, 25.2, 24.9, 24.8, 21.6, 12.6. **IR** (neat, cm^{-1}): 3078, 2980, 2931, 1730, 1636, 1513, 1436, 1404, 1372, 1318, 1218, 1144, 1069, 1043, 980, 945, 895, 844, 719, 672. **HRMS** (ESI): *m/z* [M]$^+$ calculated for $C_{11}H_{19}O_2B$: 194.1472, found 194.1469. *Note: Attention, compound is volatile.*

trans-(Z)-1-fluoro-4-(2-(prop-1-en-1-yl)cyclopropyl)benzene (28)

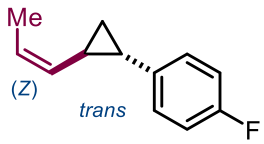

General procedure was followed using a diastereomeric mixture of vinyl cyclopropane (17.6 mg, 0.100 mmol, 65:35 *cis*/*trans* (^{1}H NMR)) and Nickel dimer (4.8 mg, 5 mol%). The reaction mixture was stirred for 2 h at room temperature. Purification by filtration over a short silica plug washing with Et_2O afforded the title product as a colorless oil (17.0 mg, 0.096 mmol, 96%, 11:89 *cis*/*trans* (^{1}H NMR)). **R$_f$** (pentane) = 0.88. **^{1}H NMR** (600 MHz, $CDCl_3$) δ 7.07 – 7.02 (m, 2H), 6.98 – 6.92 (m, 2H), 5.46 (dq, *J* = 10.5, 6.8 Hz, 1H), 4.96 (ddq, *J* = 10.9, 9.2, 1.8 Hz, 1H), 1.86 (ddd, *J* = 9.3, 5.0 Hz, 1H), 1.81 – 1.75 (m, 1H), 1.72 (dd, *J* = 6.9, 1.7 Hz, 3H), 1.17 (ddd, *J* = 8.6, 5.3 Hz, 1H), 1.00 (ddd, *J* = 8.6, 5.2 Hz, 1H). **^{13}C NMR** (151 MHz, $CDCl_3$) δ 161.3 (d, *J* = 243.2 Hz), 138.4 (d, *J* = 3.1 Hz), 133.0, 127.3 (d, *J* = 7.8 Hz), 123.5, 115.2 (d, *J* = 21.2 Hz), 24.5, 22.3, 17.1, 13.4. **^{19}F NMR** (565 MHz, $CDCl_3$) δ -118.0 (m). **HRMS** (EI): *m/z* [M]$^+$ calculated for $C_{12}H_{13}F$: 176.0996, found 176.0987. *Note: The product is volatile.*

trans-5-(2-((_Z_)-prop-1-en-1-yl)cyclopropyl)benzo[_d_][1,3]dioxole (29)

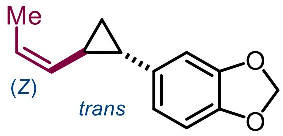

General procedure was followed using a diastereomeric mixture of vinyl cyclopropane (20.2 mg, 0.100 mmol, 65:35 *cis*/*trans* (^{1}H NMR)) and Nickel dimer (4.8 mg, 5 mol%). The reaction mixture was stirred for 2 h at room temperature. Purification by filtration over a short silica plug washing with Et_2O afforded the title product as a yellowish oil (19.0 mg, 0.093 mmol, 94%, 10:90 *cis*/*trans* (^{1}H NMR)). **R$_f$ =** 0.3 (100:1 pentane:Et_2O). **^{1}H NMR** (600 MHz, $CDCl_3$) δ 6.72 (d, *J* = 8.0 Hz, 1H), 6.60 (dd, *J* = 8.0, 1.8 Hz, 1H), 6.57 (d, *J* = 1.8 Hz, 1H), 5.91 (s, 2H), 5.44 (dqd, *J* = 10.7, 6.8, 1.0 Hz, 1H), 4.94 (ddq, *J* = 11.0, 9.3, 1.8 Hz, 1H), 1.83 (ddd, *J* = 8.7, 5.6, 4.3 Hz, 1H), 1.78 – 1.73 (m, 1H), 1.72 (dd, *J* = 6.9, 1.8 Hz, 3H), 1.14 (ddd, *J* = 8.6, 5.6, 4.8 Hz, 1H), 0.95 (dt, *J* = 8.6, 5.2 Hz, 1H). **^{13}C NMR** (151 MHz, $CDCl_3$) δ 147.8, 145.6, 136.7, 133.1, 123.3, 119.2, 108.2, 106.4, 100.9, 25.1, 22.1, 16.9, 13.4. **IR** (neat, cm^{-1}): 3781, 3674, 3566, 3070, 3011, 2886, 2776, 2322, 2079, 1857, 1653, 1608, 1493, 1441, 1236, 1207, 1103, 1074, 1037, 965, 933, 880, 804, 743, 700. **HRMS** (EI): *m/z* [M]$^+$ calculated for $C_{13}H_{14}O_2$: 202.0988, found 202.0988.

trans-(Z)-1-chloro-2-(2-styrylcyclopropyl)benzene (30)

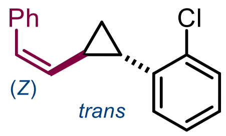

General procedure was followed using a diastereomeric mixture of vinyl cyclopropane (12.7 mg, 0.050 mmol, 69:31 *cis/trans* (^{1}H NMR)) and Nickel dimer (2.4 mg, 5 mol%). The reaction mixture was stirred for 2 h at room temperature. Purification by filtration over a short silica plug washing with Et_2O afforded the title product as a colorless oil (12.0 mg, 0.047 mmol, 94%, 19:81 *cis/trans* (^{1}H NMR)). **R_f** = 0.4 (50:1 hexane:EtOAc). **^{1}H NMR** (600 MHz, $CDCl_3$) δ 7.41 – 7.37 (m, 2H), 7.33 – 7.27 (m, 2H), 7.26 – 7.18 (m, 2H), 7.17 (ddd, *J* = 7.6, 7.6, 1.2 Hz, 1H), 7.12 (ddd, *J* = 7.6, 7.6, 1.6 Hz, 1H), 6.94 (dd, *J* = 7.6, 1.3 Hz, 1H), 6.47 (d, *J* = 11.5 Hz, 1H), 5.34 (dd, *J* = 11.3, 9.8 Hz, 1H), 2.40 – 2.34 (m, 1H), 2.14 – 2.06 (m, 1H), 1.33 (dt, *J* = 8.6, 5.5 Hz, 1H), 1.19 (dt, *J* = 8.7, 5.2 Hz, 1H). **^{13}C NMR** (151 MHz, $CDCl_3$) δ 139.2, 137.7, 135.4, 134.8, 129.4, 128.9, 128.6, 128.3, 127.1, 126.9, 126.7, 126.3, 24.1, 22.7, 16.8. **MS** (70eV, EI): *trans isomer* (GC retention time 10.350 min), *m/z* (%): 254 (13) [M$^+$], 215 (5), 202 (10), 189 (4), 178 (4), 163 (5), 149 (2), 141 (11), 129 (100), 91 (23). **HRMS** (APCI): *m/z* [M+H]$^+$ calculated for $C_{17}H_{16}Cl$: 255.0935, found 255.0933.

trans-(Z)-1-methoxy-4-(2-(oct-1-en-1-yl)cyclopropyl)benzene (31)

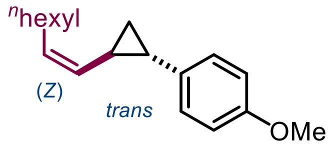

General procedure was followed using a diastereomeric mixture of vinyl cyclopropane (25.8 mg, 0.100 mmol, 54:46 *cis/trans*, 91:9 *Z/E* (^{1}H NMR)) and Nickel dimer (4.8 mg, 5 mol%). The reaction mixture was stirred for 2 h at room temperature. Purification by filtration over a short silica plug washing with Et_2O afforded the title product as a colorless oil (25.3 mg, 0.098 mmol, 98%, 12:88 *cis/trans*, 91:9 *Z/E* (^{1}H NMR)). **R_f** = 0.5 (50:1 hexane:EtOAc). **^{1}H NMR** (600 MHz, $CDCl_3$) δ 7.05 – 7.00 (m, 2H), 6.84 – 6.80 (m, 2H), 5.37 (dt, *J* = 10.7, 7.4 Hz, 1H), 4.96 – 4.89 (m, 1H), 3.79 (s, 3H), 2.19 – 2.09 (m, 2H), 1.83 (ddd, *J* = 9.1, 5.1 Hz, 1H), 1.78 – 1.70 (m, 1H), 1.40 – 1.33 (m, 2H), 1.34 – 1.22 (m, 6H), 1.14 (ddd, *J* = 8.5, 5.2 Hz, 1H), 0.95 (ddd, *J* = 8.6, 5.2 Hz, 1H), 0.87 (t, *J* = 6.9 Hz, 3H). **^{13}C NMR** (151 MHz, $CDCl_3$) δ 157.9, 134.8, 132.5, 129.3, 127.0, 113.9, 55.5, 31.9, 29.9, 29.1, 27.9, 24.6, 22.8, 22.2, 16.9, 14.3. **HRMS** (APCI): *m/z* [M+H]$^+$ calculated for $C_{18}H_{27}O$: 259.2056, found 259.2062.

trans-(E)-1-methoxy-4-(2-(oct-1-en-1-yl)cyclopropyl)benzene (32)

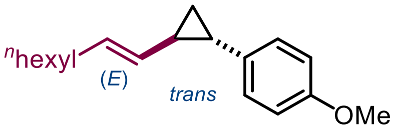

General procedure was followed using a diastereomeric mixture of vinyl cyclopropane (15.0 mg, 0.058 mmol, 59:41 *cis/trans*, 14:86 *Z/E* (^{1}H NMR)) and Nickel dimer (2.8 mg, 5 mol%). The reaction mixture was stirred for 48 h at 60 °C. Flash silica gel column chromatography (100:1 pentane:Et_2O) afforded the title compound as a colorless oil (13.9 mg, 0.054 mmol, 93%, 9:91 *cis/trans*, 15:85 *Z/E* (^{1}H NMR)). **R_f** = 0.24 (100:1 pentane:Et_2O). **^{1}H NMR** (400 MHz, $CDCl_3$) δ 6.99 (d, *J* = 8.6 Hz, 2H), 6.81 (d, *J* = 8.5 Hz, 2H), 5.52 (dt, *J* = 14.3, 6.8 Hz, 1H), 5.13 (dd, *J* = 15.2, 8.2 Hz, 1H), 3.78 (s, 3H), 1.99 (q, *J* = 6.9 Hz, 2H), 1.80 (dt, *J* = 9.4, 5.1 Hz, 1H), 1.56 – 1.51 (m, 1H), 1.38 – 1.23 (m, 8H), 1.06 (dt, *J* = 8.5, 5.3 Hz, 1H), 0.98 (dt, *J* = 8.5, 5.3 Hz, 1H), 0.88 (t, *J* = 6.8 Hz, 3H). **^{13}C NMR** (151 MHz, $CDCl_3$) δ 157.8, 134.9, 132.2, 129.3, 126.9, 113.9, 55.5, 32.7, 31.9, 29.8, 29.0, 26.1, 24.3, 22.8, 16.4, 14.3. **HRMS** (APCI): *m/z* [M+H]$^+$ calculated for $C_{18}H_{27}O$: 259.2056, found 259.2061.

trans-1-(2-(cyclohex-1-en-1-yl)cyclopropyl)-4-methoxybenzene (33)

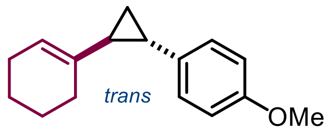

General procedure was followed using a diastereomeric mixture of vinyl cyclopropane (45.7 mg, 0.200 mmol, 25:75 *cis/trans* (^{1}H NMR)) and Nickel dimer (9.7 mg, 5 mol%). The reaction mixture was stirred for 24 h at 60 °C. Flash silica gel column chromatography (30:1 hexane:EtOAc) afforded the title compound as a colorless oil (42.9 mg, 0.188 mmol, 94%, 5:95 *cis/trans* (^{1}H NMR)). **R_f** = 0.67 (10:1 hexane:EtOAc). **^{1}H NMR** (600 MHz, $CDCl_3$) δ 7.04 – 7.00 (m, 2H), 6.83 – 6.78 (m, 2H), 5.51 – 5.46 (m, 1H), 3.78 (s, 3H), 2.04 – 1.98 (m, 2H), 1.96 – 1.90 (m, 2H), 1.86 (ddd, *J* = 9.0, 5.2 Hz, 1H), 1.67 – 1.60 (m, 2H), 1.61 – 1.54 (m, 2H), 1.53 – 1.46 (m, 1H), 1.16 – 1.09 (m, 1H), 0.93 (ddd, *J* = 8.8, 5.2 Hz, 1H). **^{13}C NMR** (151 MHz, $CDCl_3$) δ 157.8, 137.5, 135.5, 127.0, 120.3, 113.9, 55.5, 29.8, 27.1, 25.4, 23.1, 22.8, 22.5, 13.9. **HRMS** (APCI): *m/z* $[M+H]^+$ calculated for $C_{16}H_{21}O$: 229.1587, found 229.1591.

trans-1-methoxy-4-(2-(2-methylprop-1-en-1-yl)cyclopropyl)benzene (34)

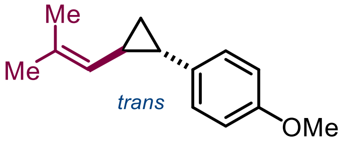

General procedure was followed using a diastereomeric mixture of vinyl cyclopropane (40.5 mg, 0.200 mmol, 57:43 *cis/trans* (^{1}H NMR)) and Nickel dimer (19.3 mg, 10 mol%). The reaction mixture was stirred for 72 h at 60 °C. Flash silica gel column chromatography (50:1 pentane:Et_2O) afforded the title compound as a colorless oil (36.8 mg, 0.182 mmol, 91%, 19:81 *cis/trans* (1H NMR)). **R_f** = 0.25 (50:1 pentane:Et_2O). **^{1}H NMR** (400 MHz, $CDCl_3$) δ 7.01 (d, *J* = 8.7 Hz, 2H), 6.81 (d, *J* = 8.6 Hz, 2H), 4.73 (dt, *J* = 9.1, 1.6 Hz, 1H), 3.78 (s, 3H), 1.78 (dt, *J* = 9.1, 4.9 Hz, 1H), 1.72 – 1.70 (m, 6H), 1.63 (tt, *J* = 9.0, 4.9 Hz, 1H), 1.10 (dt, *J* = 8.6, 5.2 Hz, 1H), 0.90 (dt, *J* = 8.6, 5.1 Hz, 1H). **^{13}C NMR** (101 MHz, $CDCl_3$) δ 157.8, 135.1, 131.5, 127.5, 126.9, 113.9, 55.5, 25.7, 24.3, 22.8, 18.5, 16.7. **HRMS** (ESI): *m/z* $[M]^+$ calculated for $C_{14}H_{19}O$: 203.1430, found 203.1428.

trans-1-fluoro-4-(2-(1-phenylvinyl)cyclopropyl)benzene (35)

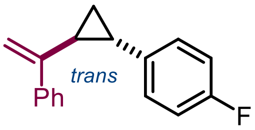

General procedure was followed using a diastereomeric mixture of 1-fluoro-4-(2-(1-phenylvinyl)cyclopropyl)benzene (23.8 mg, 0.100 mmol, 1.0 equiv., 86:14 *cis/trans* (^{1}H NMR)) and Nickel dimer (5 mol%). The reaction mixture was stirred for 24 h at at 60 °C. Purification by filtration over a short silica plug washing with Et_2O afforded the title product as a colorless oil (22.9 mg, 0.096 mmol, 96%, 95:5 *trans/cis* (^{1}H NMR)). **^{1}H NMR** (600 MHz, $CDCl_3$) δ 7.45 – 7.40 (m, 2H), 7.27 – 7.16 (m, 3H), 7.05 – 7.00 (m, 2H), 6.94 – 6.89 (m, 2H), 5.29 (s, 1H), 4.96 (s, 1H), 1.93 – 1.89 (m, 1H), 1.85 – 1.80 (m, 1H), 1.34 – 1.29 (m, 1H), 1.18 – 1.12 (m, 1H).**^{13}C NMR** (151 MHz, $CDCl_3$) δ 161.4 (d, *J* = 243.7 Hz), 148.3, 141.1, 138.3 (d, *J* = 3.2 Hz), 128.4, 127.8, 127.3 (d, *J* = 7.8 Hz), 126.2, 115.4 (d, *J* = 21.3 Hz), 109.6, 27.9, 25.8, 15.9. **^{19}F NMR** (565 MHz, $CDCl_3$) δ -117.62. **HRMS** (EI): *m/z* $[M]^+$ calculated for $C_{17}H_{15}F$: 238.1158, found 238.1156.

trans-1-fluoro-4-(2-(prop-1-en-2-yl)cyclopropyl)benzene (36)

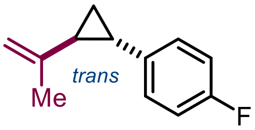

General procedure was followed using a diastereomeric mixture of 1-fluoro-4-(2-(prop-1-en-2-yl)cyclopropyl)benzene (17.6 mg, 0.100 mmol, 1.0 equiv., 62:38 *cis/trans* (^{1}H NMR)) and Nickel dimer (5 mol%). The reaction mixture was stirred for 24 h at at 60 °C. Purification by filtration over a short silica plug washing with Et_2O afforded the title product as a colorless oil (16.3 mg, 0.093 mmol, 93%, 95:5 *trans/cis* (^{1}H NMR)).

^{1}H NMR (600 MHz, $CDCl_3$) δ 7.07 – 7.02 (m, 2H), 6.95 (m, 2H), 4.76 – 4.75 (m, 1H), 4.74 (m, 1H), 1.96 (dt, *J* = 8.8, 5.2 Hz, 1H), 1.73 (s, 3H), 1.62 (dt, *J* = 8.7, 5.3 Hz, 1H), 1.20 (m, 1H), 1.06 – 1.02 (m, 1H). **^{13}C NMR** (151 MHz, $CDCl_3$) δ 161.3 (d, *J* = 243.4 Hz), 145.3, 138.6 (d, *J* = 3.0 Hz), 127.4 (d, *J* = 7.9 Hz), 115.2 (d, *J* = 21.2 Hz), 108.8, 29.9, 23.4, 20.9, 15.1. **^{19}F NMR** (565 MHz, $CDCl_3$) δ -117.93. **HRMS** (EI): *m/z* $[M]^+$ calculated for $C_{12}H_{13}F$: 176.1001, found 176.0993.

tert-butyl (1*S*,2*S*)-1-((*tert*-butoxycarbonyl)amino)-2-vinylcyclopropane-1-carboxylate (38)

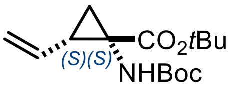

General isomerization procedure was followed using tert-butyl (1*R*,2*S*)-1-((*tert*-butoxycarbonyl)amino)-2-vinylcyclopropane-1-carboxylate **37** (28.3 mg, 0.100 mmol, >99% ee) and Nickel dimer (0.9 mg, 1 mol%). The reaction mixture was stirred for 10 min at room temperature. Filtration over a short plug of silica eluting with Et_2O afforded the crude compound as a diastereomeric mixture as a viscous oil (27 mg, 0.096 mmol, 96%, 14:86 *cis*/*trans* (^{1}H NMR)). *Note: The diastereomers can either be separated by using silica flash column chromatography (4:1 pentane:Et_2O) or crystallization from hot hexane (100 mg/1 mL).*

R_f (*trans*-isomer) = 0.32 (4:1 pentane:Et_2O, PMA). **$[\alpha]_D^{25}$** = –37.6 (*c* 1.0 $CHCl_3$, 99% ee). **M.p.** = 101.6 – 104.1 °C. **^{1}H NMR** (600 MHz, $CDCl_3$) δ 5.52 (dt, *J* = 18.2, 9.4 Hz, 1H), 5.25 (d, *J* = 17.2 Hz, 1H), 5.14 (d, *J* = 10.3 Hz, 1H), 4.90 (s, 0.6H, NH major rotamer), 4.69 (s, 0.3H, NH minor rotamer), 2.27 (q, *J* = 9.0, 8.6 Hz, 1H), 1.86 – 1.79 (m, 1H), 1.44 (s, 18H), 1.22 – 1.16 (m, 1H). **^{13}C NMR** (151 MHz, $CDCl_3$) δ 171.5, 156.1, 134.5, 117.9, 81.5, 79.8, 40.2, 30.8, 28.4, 28.1, 22.7. **IR** (neat, cm^{-1}): 3338, 3092, 2979, 2935, 2324, 2199, 2169, 2112, 1968, 1704, 1640, 1515, 1364, 1346, 1252, 1159, 1085, 1059, 991, 951, 905, 847, 762, 735, 697, 662. **HRMS** (ESI): *m/z* $[M+Na]^+$ calculated for $C_{15}H_{25}O_4NNa$: 306.1676, found 306.1682.

6. Scale up of isomerization

trans-ethyl 2-vinylcyclopropane-1-carboxylate (16)

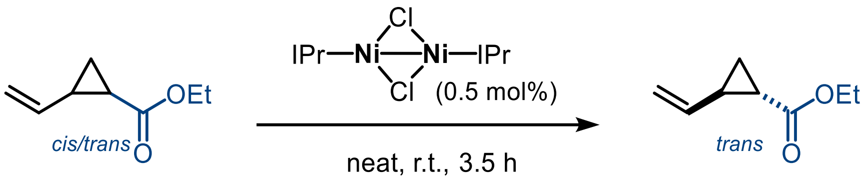

Inside an argon-filled glovebox, a 20 mL screw-cap vial equipped with a magnetic stir bar was charged with ethyl 2-vinylcyclopropane-1-carboxylate (1.000 g, 7.130 mmol, 30:70 *cis/trans* (^{1}H NMR)) and [Ni(μ-Cl)(IPr)]$_2$ **1** (33.4 mg, 0.5 mol%). The reaction vial was stirred inside the glovebox at room temperature for 3.5 hours (reaction progress was monitored by GC-MS). The reaction mixture was then removed from the glovebox and diluted with Et_2O (5 mL). Several spatula tips of ammonium pyrrolidine-1-dithiocarboxylic acid were added and the mixture stirred for additional 30 min to precipitate nickel.[13] Purification by filtration over a short silica plug washing with Et_2O afforded the title product as a colorless oil (934 mg, 6.660 mmol, 93%, 8:92 *cis/trans* (^{1}H NMR)). **R_f** = 0.2 (50:1 pentane:Et_2O, PMA). **^{1}H NMR** (600 MHz, $CDCl_3$) δ 5.39 (ddd, *J* = 17.1, 10.2, 8.4 Hz, 1H), 5.16 (d, *J* = 17.0 Hz, 1H), 4.98 (d, *J* = 10.3 Hz, 1H), 4.13 (q, *J* = 7.1 Hz, 2H), 2.01 (tdd, *J* = 8.8, 6.1, 4.0 Hz, 1H), 1.63 (dt, *J* = 8.8, 4.6 Hz, 1H), 1.36 (dt, *J* = 9.2, 4.7 Hz, 1H), 1.26 (t, *J* = 7.1 Hz, 3H), 0.96 (ddd, *J* = 8.4, 6.2, 4.4 Hz, 1H). **^{13}C NMR** (151 MHz, $CDCl_3$) δ 173.5, 138.3, 114.9, 60.7, 25.7, 22.0, 15.7, 14.4. **IR** (neat, cm^{-1}): 3441, 3086, 2983, 2347, 2066, 1723, 1639, 1449, 1384, 1269, 1171, 1092, 1037, 985, 906, 850, 818, 738, 690. **HRMS** (ESI): *m/z* $[M+Na]^+$ calculated for $C_8H_{12}O_2Na$: 163.0730, found 163.0732. *Note: Caution, compound is volatile.*

7. Mechanistic studies

7.1. Study on driving force

Inside an argon-filled glovebox, an oven dried 4 mL screw-cap vial equipped with a magnetic stirring bar was charged with the corresponding (*cis*, *cis/trans*, or *trans*) 1-methoxy-4-(2-vinylcyclopropyl)benzene (17.4 mg, 0.100 mmol, 1.0 equiv.), anhydrous 1,4-dioxane (0.25 mL, 0.4 M) and [Ni(μ-Cl)(IPr)]$_2$ **1** (1-5 mol%), in that order. The reaction vial was then sealed and allowed to stir inside the glovebox at the indicated temperature for the indicated time. Upon completion the reaction mixture was removed from the glovebox and quenched by the addition of wet pentane (*i.e.* technical grade pentane that had been distilled and stored on the bench). A spatula tip of ammonium pyrrolidine-1-dithiocarboxylic acid was added and the mixture stirred for additional 15 min to precipitate nickel.[13] The mixture was filtered through a plug of silica, rinsed with Et_2O and the filtrate was concentrated under reduced pressure. The crude was analyzed by quantitative ^{1}H NMR spectroscopy. Regardless of the initial *cis/trans* ratio and even with increased catalyst loadings, higher temperatures or longer reaction times the same diastereomeric ratio was obtained after isomerization.

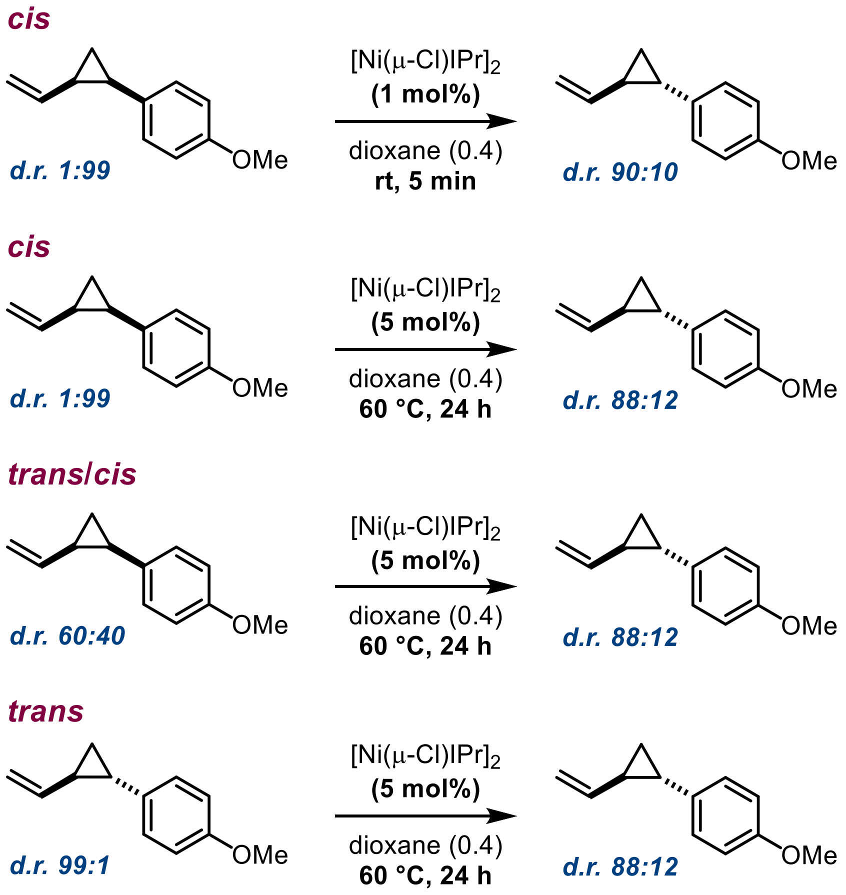

7.2. Isomerization reaction using mononuclear Nickel(I) complex

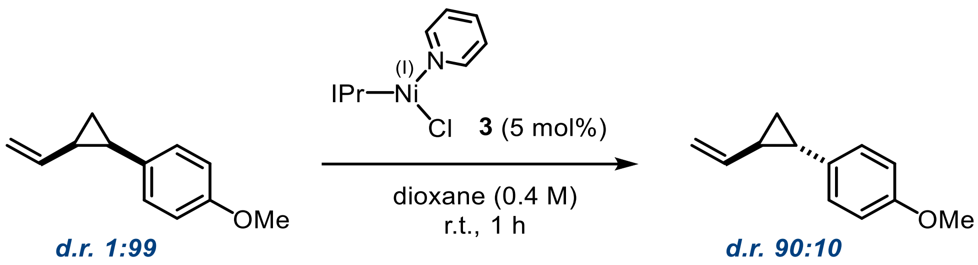

Inside an argon-filled glovebox, an oven dried 4 mL screw-cap vial equipped with a magnetic stirring bar was charged with 1-methoxy-4-(*cis*-2-vinylcyclopropyl)benzene (17.4 mg, 0.100 mmol, 1.0 equiv.), anhydrous 1,4-dioxane (0.25 mL, 0.4 M) and Ni(Cl)(IPr)(pyridine) **3** (2.8 mg, 5 mol%), in that order. The reaction vial was then sealed and allowed to stir inside the glovebox at room temperature for 1 h. Upon completion the reaction mixture was removed from the glovebox and quenched by the addition of wet pentane (*i.e.* technical grade pentane that had been distilled and stored on the bench). A spatula tip of ammonium pyrrolidine-1-dithiocarboxylic acid was added and the mixture stirred for additional 15 min to precipitate nickel.[13] The mixture was filtered through a plug of silica, rinsed with Et_2O and the filtrate was concentrated under reduced pressure. The crude was analyzed by quantitative ^1H NMR spectroscopy using ethylene carbonate (2.0 mg, 0.023 mmol) as internal standard. The *trans* (0.089 mmol, 89%) and *cis* (0.010 mmol, 10%) isomers were obtained in similar ratio as under standard conditions using [Ni(μ-Cl)(IPr)]$_2$ **1**.

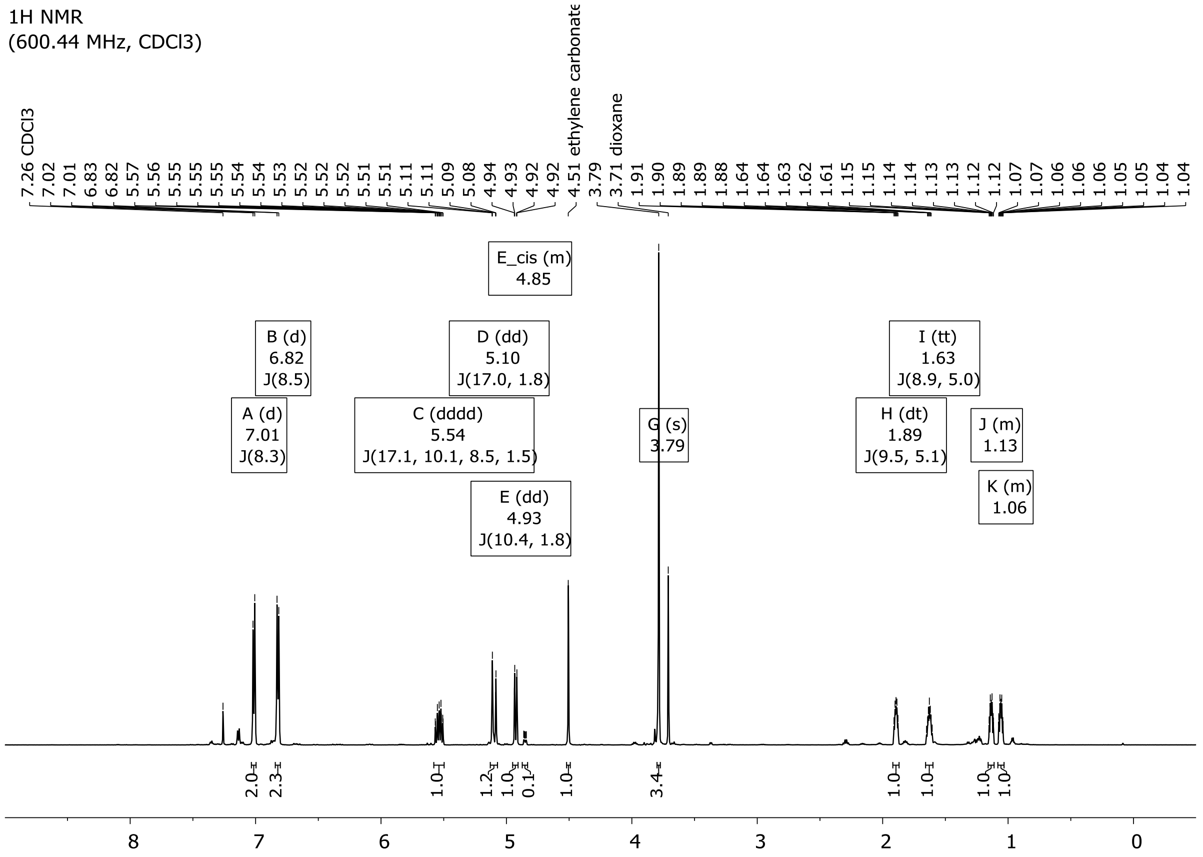

Figure S1. Quantitative ^1H NMR analysis of reaction crude using ethylene carbonate (4.51 ppm) as internal standard.

7.3. Reactivity comparison Ni(I) vs. Ni(0)

7.3.1. Solvent effect on isomerization

General Procedure for Ni$^{(I)}$ solvent screen: Inside an argon-filled glovebox, an oven dried 4 mL screw-cap vial equipped with a magnetic stirring bar was charged with 1-methoxy-4-(*cis*-2-vinylcyclopropyl)benzene **S2** (8.7 mg, 0.050 mmol, 1.0 equiv.), anhydrous solvent (0.125 mL, 0.4 M) and [Ni(μ-Cl)(IPr)]$_2$ **1** (2.4 mg, 5 mol%) in that order. The reaction vial was then sealed and allowed to stir outside the glovebox at room temperature for 5 minutes. Upon completion the reaction mixture was quenched by the addition of wet pentane (*i.e.* technical grade pentane that had been distilled and stored on the bench). A spatula tip of ammonium pyrrolidine-1-dithiocarboxylic acid was added and the mixture stirred for additional 15 min to precipitate nickel.[13] The mixture was filtered through a plug of silica, rinsed with Et_2O and the filtrate was concentrated under reduced pressure. The crude was analyzed by quantitative ^1H NMR spectroscopy using ethylene carbonate (2.0 mg, 0.023 mmol) as internal standard.

Table S10. Solvent effect on isomerization mediated by Ni$^{(I)}$ dimer **1**.

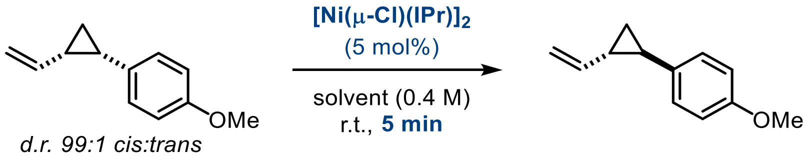

entry	solvent	d.r. (*cis*:*trans*)	yield[a]
**1**	dioxane	10:90	98
**2**	THF	10:90	97
**3**	Et_2O	11:89	94
**4**	benzene	11:89	96
**5**	toluene	11:89	97

a) Quantitative ^1H NMR yield using ethylene carbonate as internal standard.

General Procedure for Ni(0) solvent screen: Inside an argon-filled glovebox, an oven dried 4 mL screw-cap vial equipped with a magnetic stirring bar was charged with 1-methoxy-4-(*cis*-2-vinylcyclopropyl)benzene **S2** (8.7 mg, 0.050 mmol, 1.0 equiv.), anhydrous solvent (0.125 mL, 0.4 M), Ni(cod)$_2$ (0.7 mg, 5 mol%) and IPr-ligand (1.94 mg, 10 mol%) in that order. The reaction vial was then sealed and allowed to stir inside the glovebox at room temperature for 1 hour. Upon completion the reaction mixture was removed from the glovebox and quenched by the addition of wet pentane (*i.e.* technical grade pentane that had been distilled and stored on the bench). A spatula tip of ammonium pyrrolidine-1-dithiocarboxylic acid was added and the mixture stirred for additional 15 min to precipitate nickel.[13] The mixture was filtered through a plug of silica, rinsed with Et_2O and the filtrate was concentrated under reduced pressure. The crude was analyzed by quantitative ^1H NMR spectroscopy using ethylene carbonate (2.0 mg, 0.023 mmol) as internal standard.

Table S11. Solvent effect on isomerization mediated by Ni(0)/IPr-ligand (1:2).

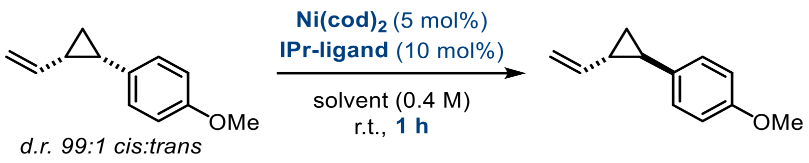

entry	solvent	d.r. (*cis*:*trans*)	yield[a]
**1**	dioxane	15:85	99
**2**	THF	61:39	99
**3**	Et_2O	65:35	95
**4**	benzene	64:36	97
**5**	toluene	60:40	94

a) Quantitative ^{1}H NMR yield using ethylene carbonate as internal standard.

While for Ni(I) dimer, the isomerization was equally efficient in the various solvents, for Ni(0), there was enhanced isomerization seen in dioxane. Our EPR studies (see section 7.4) indicate that Ni(I) was formed in dioxane (Table S11, entry 1).

7.3.2. Reactivity with internal vinylcyclopropanes

General Procedure for Ni(0) isomerization: Inside an argon-filled glovebox, an oven dried 4 mL screw-cap vial equipped with a magnetic stirring bar was charged with 1-fluoro-4-(2-((Z)-prop-1-en-1-yl)cyclopropyl)benzene (17.6 mg, 0.100 mmol, 1.0 equiv., 73:27 cis/trans (^{1}H NMR)), anhydrous dioxane (0.25 mL, 0.4 M), $Ni(cod)_2$ (1.38 mg, 5 mol%) and IPr-ligand (1.94 mg, 5 mol% or 3.89 mg, 10 mol%) in that order. The reaction vial was then sealed and allowed to stir inside the glovebox at indicated temperature for indicated time. Upon completion the reaction mixture was removed from the glovebox and quenched by the addition of wet pentane (*i.e.* technical grade pentane that had been distilled and stored on the bench). A spatula tip of ammonium pyrrolidine-1-dithiocarboxylic acid was added and the mixture stirred for additional 15 min to precipitate nickel.[13] The mixture was filtered through a plug of silica, rinsed with Et_2O and the filtrate was concentrated under reduced pressure. The crude was analyzed by quantitative ^{1}H NMR spectroscopy using ethylene carbonate (2.0 mg, 0.023 mmol) as internal standard.

Table S12. Alkenyl cyclopropane isomerization: comparison of Ni(I)-dimer and Ni(0)/IPr system.

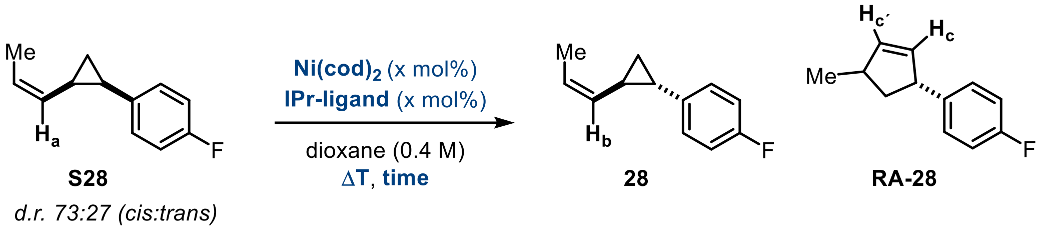

entry	catalyst	conditions	d.r. (*cis*:*trans*)[a]	yield (S28+28)[b]	yield RA-28[b]
**1**	-	starting material **S28**	73:27	-	-
**2**	Ni(I) dimer **1** (5 mol%)	r.t., 2 h	11:89	96%[c]	-
**3**	$Ni(cod)_2$ (5 mol%) IPr (5 mol%)	r.t., 2 h	72:28	96%	-
**4**	$Ni(cod)_2$ (5 mol%) IPr (10 mol%)	r.t., 2 h	72:28	95%	4%
**5**	$Ni(cod)_2$ (5 mol%) IPr (5 mol%)	60 °C, 24 h	21:79	38%	27%
**6**	$Ni(cod)_2$ (5 mol%) IPr (10 mol%)	60 °C, 24 h	23:77	61%	11%
**7**	Ni(I) dimer **1** (5 mol%)	60 °C, 24 h	14:86	81%	-

a) Quantitative ^{1}H NMR ratio, E and Z diastereomers are neglected for clarity; b) quantitative ^{1}H NMR yield using ethylene carbonate as internal standard; c) isolated yield.

With an additional substituent on the vinyl unit, the reactivity of Ni(0) is significantly lower and side-products resulting from cyclopentene formation are seen. By contrast, under Ni(I) dimer catalysis, no cyclopentene is seen regardless of the reaction time or temperature.

7.3.3. Computational study on Ni(0) reactivity

For Ni(0), computational studies predict a minimum of 10 kcal/mol greater activation free energy barrier than for the Ni(I) pathway (see Figure S2).

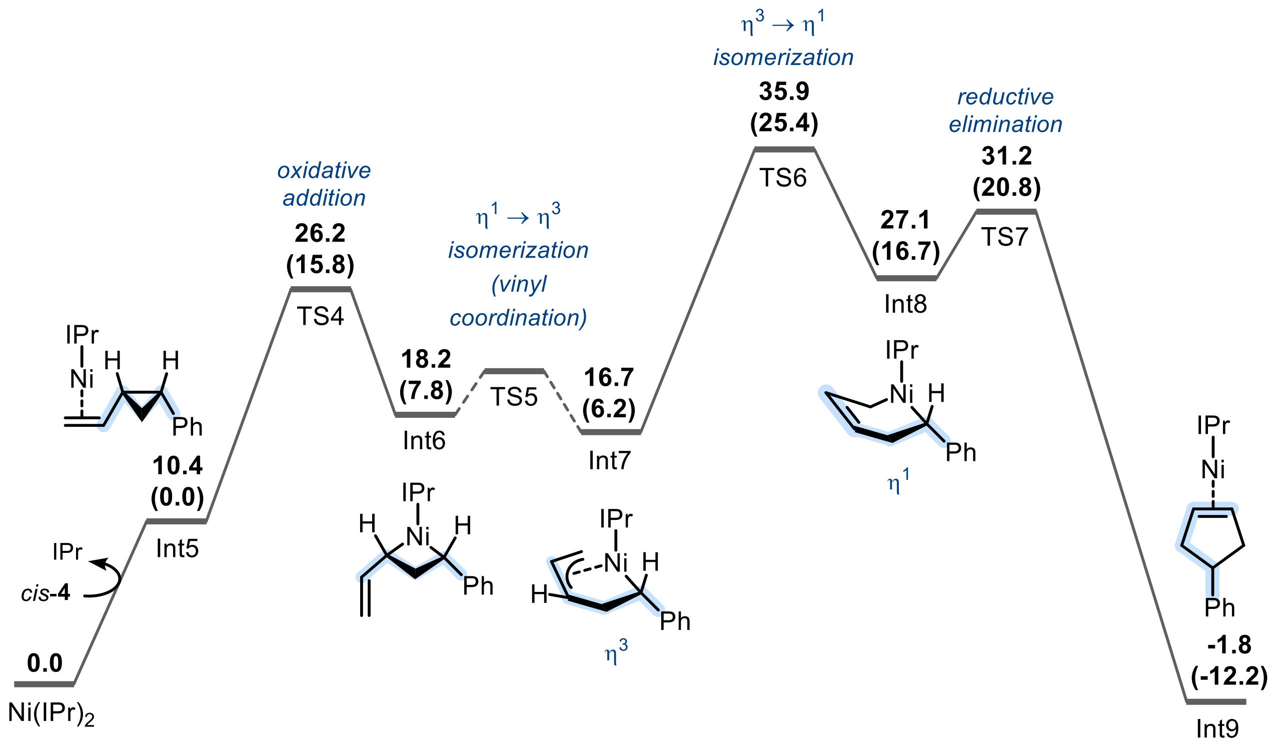

Figure S2. Computed pathway for Ni(0). Values refer to Gibbs free energies (in kcal/mol) calculated at the CPCM (dioxane) M06L/def2-TZVP//MN15/6-31G(d)(SDD) level of theory. Values in brackets are relative to **Int5** (i.e. not accounting for ligand dissociation).

7.4. EPR spectroscopic studies

Table S13. Summary of EPR data.

entry	[Ni]	substrate added?	solvent	comment	g-values[a]	HStrain[a]
**1**	Ni$^{(I)}$ dimer **1**	no	toluene, dioxane	in line with reported[22]	no signal	-
**2**	Ni$^{(I)}$ dimer **1**	olefin	PhCl	Reference[22]	[2.4495 2.1548 2.0202]	[313 115 192]
**3**	Ni$^{(I)}$ dimer **1**	yes	toluene, dioxane	in line with reported[22]	[2.4526 2.1560 2.0323]	[250 110 117]
**4**	NiCl(IPr)(py) **3**	no	toluene	two species (0.52 : 1 ratio)	[2.3610 2.2501 2.1414]<br>[2.4552 2.2082 2.0510]	[383 300 402]<br>[272 198 194]
**5**	NiCl(IPr)(py) **3**	no	toluene	Reference[12]	[2.452 2.235 2.042][12]	-
**6**	NiCl(IPr)(py) **3**	yes	toluene	two species (2.22 : 1 ratio)	[2.5493 2.3400 1.9887]<br>[2.4724 2.2099 2.0430]	[389 288 119]<br>[240 223 195]
**7**	NiCl(IPr)$_2$	no	toluene	no change if substrate is added	[2.5418 2.4021 1.9911]	[204 180 190]
**8**	Ni(cod)$_2$/IPr	yes	toluene	no signal	-	-
**9**	Ni(cod)$_2$/IPr	yes	dioxane	weak signal; two species (0.52 : 1 ratio)	[2.5657 2.3169 1.9938]<br>[2.4933 2.2024 2.0270]	[183 168 41]<br>[204 112 60]

[a]g-values and HStrain were simulated using EasySpin-5.2.35[8] in MATLAB (version R2021a).

EPR study of [Ni(μ-Cl)IPr]$_2$ **1**

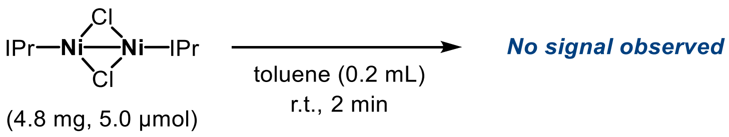

Inside an Argon-filled glovebox, [Ni(μ-Cl)(IPr)]$_2$ **1** (4.8 mg, 5.0 μmol) was dissolved in dry toluene (0.20 mL, 0.05 M). The solution was stirred for 2 min at ambient temperature inside the glovebox before being transferred inside an EPR capillary tube and sealed inside the glove box. After removing from the glovebox, the capillary tube was frozen immediately by immerging it in liquid nitrogen. No paramagnetic species was detected.

EPR study of isomerization reaction with [Ni(μ-Cl)IPr]$_2$ **1**

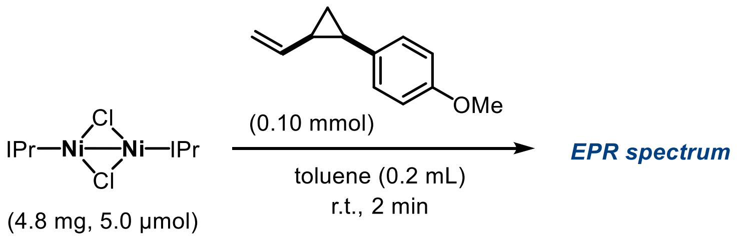

Inside an Argon-filled glovebox, [Ni(μ-Cl)(IPr)]$_2$ **1** (4.8 mg, 5.0 μmol, 10 mol% Ni) was placed to a 4 mL screw-capped vial and a stock solution of vinylcyclopropane (17.4 mg, 0.100 mmol) in dry toluene (0.20 mL, 0.5 M) was added. The solution was stirred for 2 min at ambient temperature inside the glovebox before being transferred inside an EPR capillary tube and sealed inside the glove box. After removing from the glovebox, the capillary tube was frozen immediately by immerging it in liquid nitrogen. A new paramagnetic species was detected.

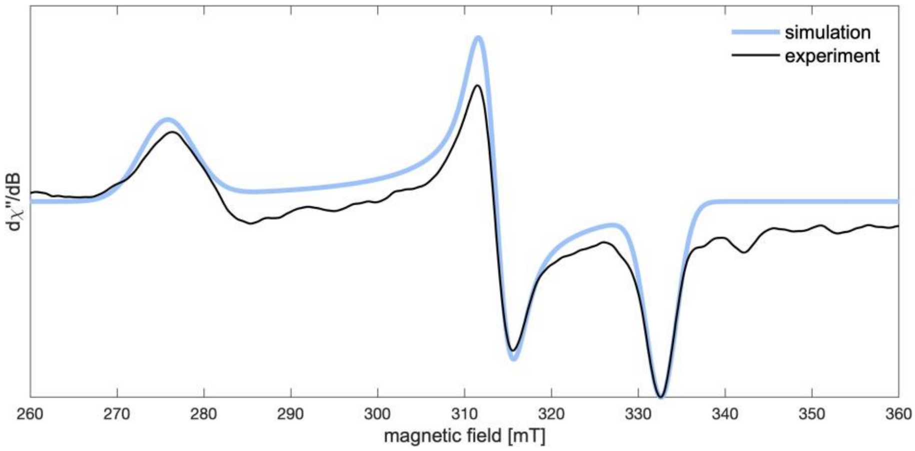

Simulation parameters:

g = [2.4526 2.1560 2.0323]

HStrain = [250 110 117]

Figure S3. CW EPR spectrum of **1** + VCP at 77 K of a frozen solution in toluene at X-band (9.464 GHz).

EPR study of Ni(Cl)(IPr)(pyridine) **3**

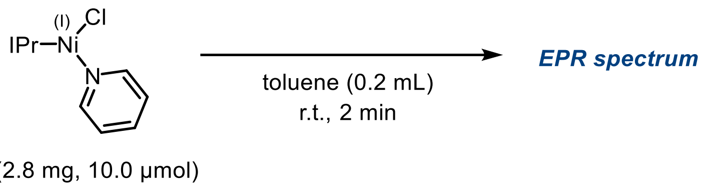

Inside an Argon-filled glovebox, Ni(Cl)(IPr)(pyridine) **3** (2.8 mg, 10.0 µmol) was dissolved in dry toluene (0.20 mL, 0.05 M). The solution was stirred for 2 min at ambient temperature inside the glovebox before being transferred inside an EPR capillary tube and sealed inside the glove box. After removing from the glovebox, the capillary tube was frozen immediately by immerging it in liquid nitrogen. A paramagnetic species was detected.

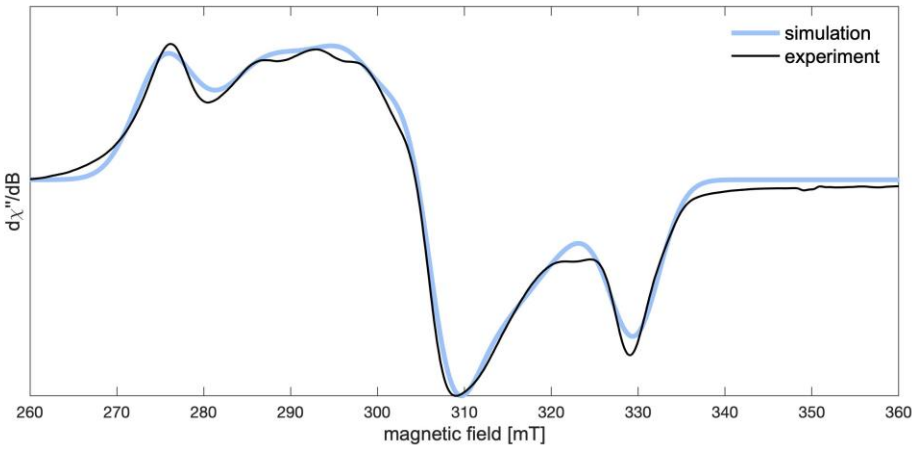

Simulation parameters:

g_A = [2.3610 2.2501 2.1414]

$HStrain_A$ = [383 300 402]

$weight_A$ = 0.5216

g_B = [2.4552 2.2082 2.0510]

$HStrain_B$ = [272 198 194]

$weight_B$ = 1

Figure S4. CW EPR spectrum of **3** at 77 K of a frozen solution in toluene at X-band (9.464 GHz).

EPR study of isomerization reaction with Ni(Cl)(IPr)(pyridine) **3**

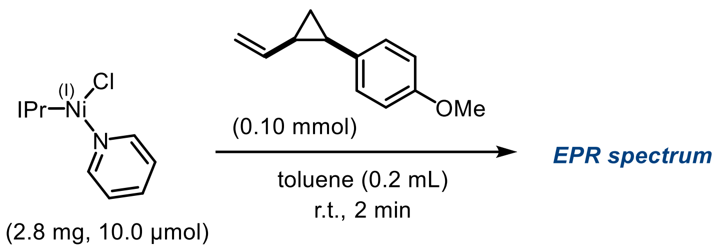

Inside an Argon-filled glovebox, Ni(Cl)(IPr)(pyridine) **3** (2.8 mg, 10.0 µmol, 10 mol% Ni) was placed to a 4 mL screw-capped vial and a stock solution of vinylcyclopropane (14.7 mg, 0.100 mmol) in dry toluene (0.20 mL, 0.5 M) was added. The solution was stirred for 2 min at ambient temperature inside the glovebox before being transferred inside an EPR capillary tube and sealed inside the glove box. After removing from the glovebox, the capillary tube was frozen immediately by immerging it in liquid nitrogen. A new paramagnetic species was detected.

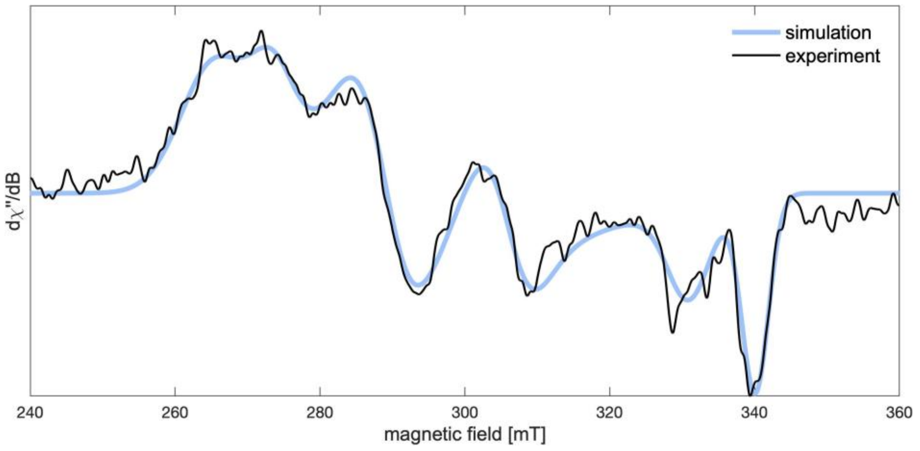

Simulation parameters:

g_A = [2.5493 2.3400 1.9887]

$HStrain_A$ = [389 288 119]

$weight_A$ = 2.2175

g_B = [2.4724 2.2099 2.0430]

$HStrain_B$ = [240 223 195]

$weight_B$ = 1

Figure S5. CW EPR spectrum of **3** + VCP at 77 K of a frozen solution in toluene at X-band (9.464 GHz).

EPR study of *in situ*-formed Ni(Cl)(IPr)$_2$

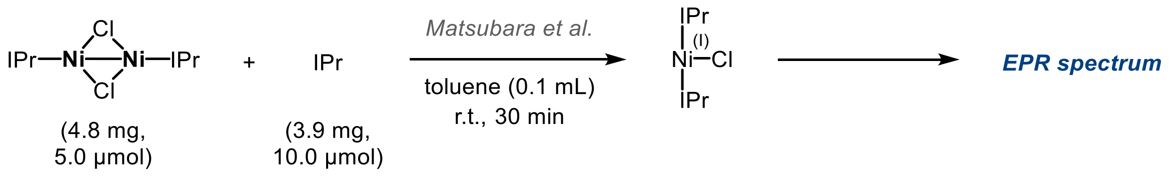

Ni(Cl)(IPr)$_2$ was formed *in situ* according to Matsubara *et al.*[23]: Inside an Argon-filled glovebox, [Ni(µ-Cl)(IPr)]$_2$ **1** (4.8 mg, 5.0 µmol, 10 mol% Ni) and IPr ligand (3.9 mg, 10.0 µmol) were weighed into a 4 mL screw-capped vial and dissolved in dry toluene (0.10 mL, 1.0 M). The solution was stirred for 30 min at ambient temperature inside the glovebox before being transferred inside an EPR capillary tube and sealed inside the glove box. After removing from the glovebox, the capillary tube was frozen immediately by immerging it in liquid nitrogen. A new paramagnetic species was detected.

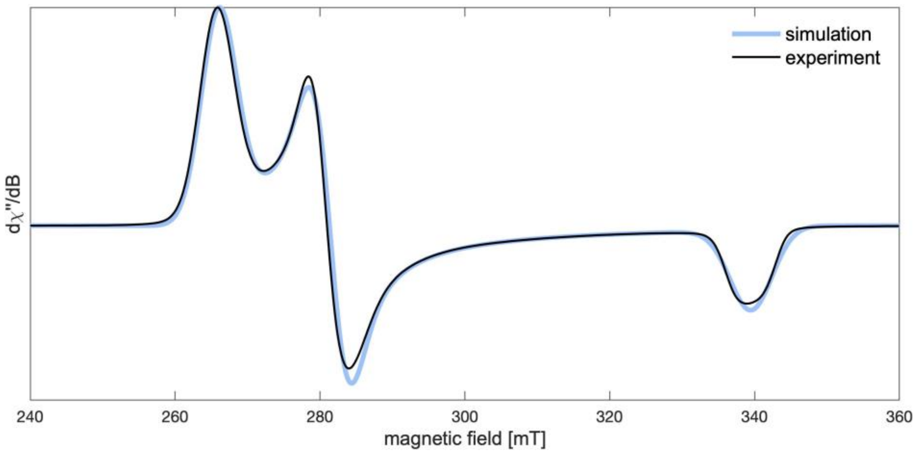

Simulation parameters:

g = [2.5418 2.4021 1.9911]

HStrain = [204 180 190]

Figure S6. CW EPR spectrum of Ni(Cl)(IPr)$_2$ at 77 K of a frozen solution in toluene at X-band (9.464 GHz).

EPR study of isomerization reaction with *in situ*-formed Ni(Cl)(IPr)$_2$

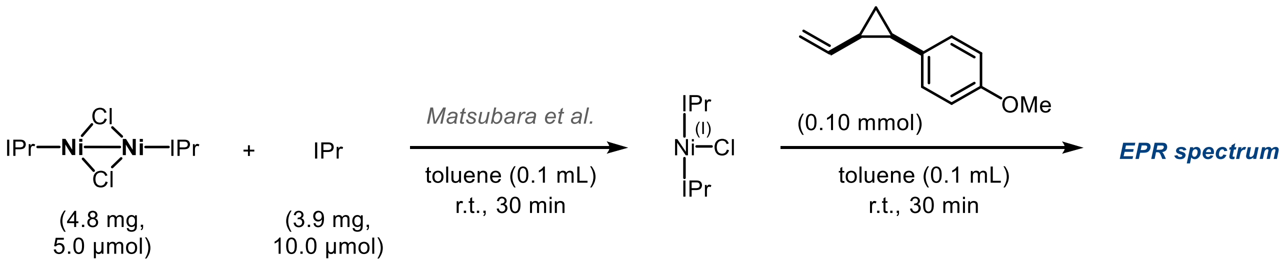

Ni(Cl)(IPr)$_2$ was formed *in situ* according to Matsubara *et al.*[23]: Inside an Argon-filled glovebox, [Ni(µ-Cl)(IPr)]$_2$ **1** (4.8 mg, 5.0 µmol, 10 mol% Ni) and IPr ligand (3.9 mg, 10.0 µmol) were weighed into a 4 mL screw-capped vial and dissolved in dry toluene (0.10 mL, 1.0 M). The solution was stirred for 30 min at ambient temperature inside the glovebox before a stock solution of vinylcyclopropane (14.7 mg, 0.100 mmol) in dry toluene (0.10 mL) was added. The solution was stirred for 30 min at ambient temperature inside the glovebox before being transferred inside an EPR capillary tube and sealed inside the glove box. After removing from the glovebox, the capillary tube was frozen immediately by immerging it in liquid nitrogen. The recorded EPR spectrum showed no change compared to the spectrum recorded without the addition of vinylcyclopropane.

EPR study of isomerization reaction with Ni$^{(0)}$(IPr)$_2$

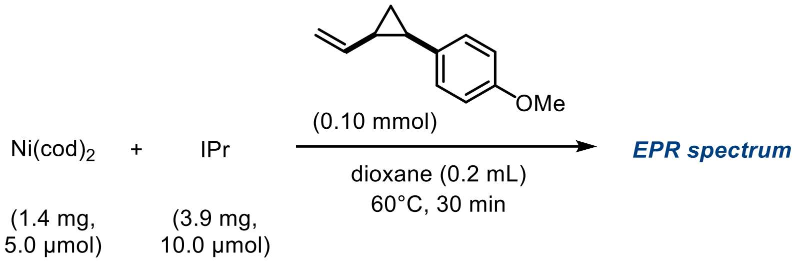

Inside an Argon-filled glovebox, Ni(cod)$_2$ (1.4 mg, 5.0 µmol) and IPr ligand (3.9 mg, 10.0 µmol) were weighed into a 4 mL screw-capped vial and dissolved in dry dioxane (0.10 mL, 1.0 M). The solution was stirred for 2 min at ambient temperature inside the glovebox before a stock solution of vinylcyclopropane (14.7 mg, 0.100 mmol) in dry dioxane (0.10 mL) was added. The solution was stirred

for 30 min at 60°C inside the glovebox before allowing to cool and being transferred inside an EPR capillary tube and sealed inside the glove box. After removing from the glovebox, the capillary tube was frozen immediately by immerging it in liquid nitrogen. The recorded EPR spectrum showed a very weak signal. Notably, no signal was observed when the reaction was performed in toluene intead of dioxane.

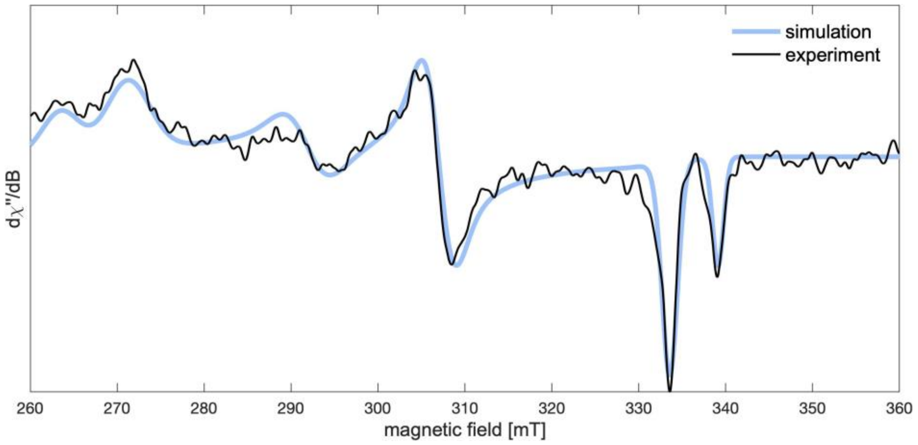

Simulation parameters:

g_A = [2.5657 2.3169 1.9938]

$HStrain_A$ = [183 168 41]

$weight_A$ = 0.5212

g_B = [2.4933 2.2024 2.0270]

$HStrain_B$ = [204 112 60]

$weight_B$ = 1

Figure S7. CW EPR spectrum of $Ni(IPr)_2$ + VCP at 77 K of a frozen solution in dioxane at X-band (9.464 GHz).

7.5. Isomerization without vinyl handle

7.5.1. Synthesis and characterization of (*cis*-2-methylcyclopropyl)benzene (27)

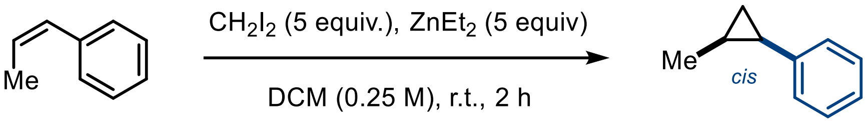

Synthesized following literature procedure.[24] Under argon atmosphere, a flame dried round bottom flask was charged with dry DCM (40 mL), *cis*-1-phenyl-1-propene (1.3 mL, 10.0 mmol, 1.0 equiv.) and diiodomethane (4.03 mL, 50.0 mmol, 5.0 equiv.). The solution was cooled to -10 °C and a solution of diethylzinc (50.0 mL, 50.0 mmol, 5 equiv., 1 M in hexane) was added dropwise over 10 min. The reaction was then allowed to warm up to room temperature over 2 h. Next, the reaction was cooled to 0 °C and sat. aqueous NaEDTA (20 mL) solution was added dropwise. The resulting suspension was then diluted with Et_2O (100 mL) and the phases were separated. The organic layer was further washed with H_2O (20 mL), brine (20 mL), dried over $MgSO_4$, filtered and concentrated under reduced pressure. The crude product was further purified by HPLC (*Column: LiChrosorb Si (250x25)mm, 7 µm; Mobile phase: n-hexane; Flow rate: 18 mL/min; Pressure: 30 bar*) to afford (*cis*-2-methylcyclopropyl)benzene (207 mg, 1.57 mmol, 16%) as a colorless oil. $\mathbf{R_f}$ = 0.9 (hexane). **^{1}H NMR** (600 MHz, $CDCl_3$) δ 7.28 (dd, *J* = 7.6 Hz, 2H), 7.23 – 7.16 (m, 3H), 2.09 (td, *J* = 8.7, 5.9 Hz, 1H), 1.15 (dddd, *J* = 14.8, 12.2, 8.7, 6.2 Hz, 1H), 0.99 (td, *J* = 8.4, 4.9 Hz, 1H), 0.81 (d, *J* = 6.3 Hz, 3H), 0.59 (q, *J* = 5.5 Hz, 1H). **^{13}C NMR** (151 MHz, $CDCl_3$) δ 139.7, 129.4, 128.0, 125.7, 21.2, 13.7, 12.8, 11.0. **IR** (neat, cm^{-1}): 3063, 3005, 2952, 2872, 2333, 2186, 2104, 1998, 1943, 1879, 1802, 1748, 1603, 1496, 1450, 1388, 1353, 1304, 1229, 1170, 1070, 1029, 994, 909, 864, 841, 755, 725, 696. **MS** (70eV, EI): *m/z* (%): 132 (37%) [M^+], 117 (100), 103 (6), 91 (29), 77 (9), 65 (7), 58 (1), 51 (7). The data are in agreement with those previously reported in the literature.[25]

7.5.2. Isomerization of (*cis*-2-methylcyclopropyl)benzene (27)

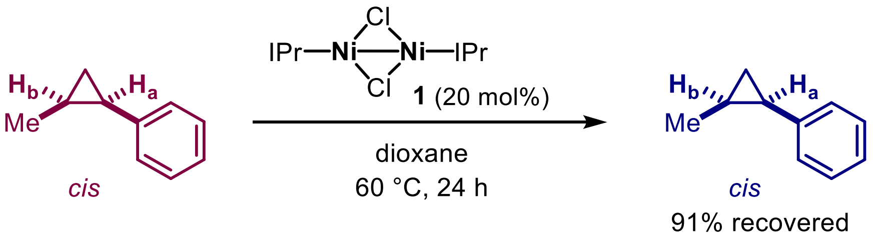

Inside an argon filled glovebox (*cis*-2-methylcyclopropyl)benzene **27** (26.4 mg, 0.200 mmol) was placed to an oven dried 4 mL screw-cap vial and dissolved in dry dioxane (0.5 mL, 0.4 M). Then, $[Ni(\mu\text{-}Cl)(IPr)]_2$ **1** (38.6 mg, 20 mol%) was added. The reaction vial was then sealed and allowed to stir in a pre-heated aluminium block inside the glovebox for 24 h at 60 °C. The mixture was removed from the glovebox, diluted with wet hexane (*i.e.* technical grade hexane that had been distilled and stored on the bench) and a spatula tip of ammonium pyrrolidine-1-dithiocarboxylic acid was added and the mixture stirred for additional 15 min to precipitate nickel.[13] It was filtered over plug of silica diluted with pentane and solvent was removed under reduced pressure. The crude was dissolved in a minimum amount of pentane and filtered again over a short plug of silica diluted with pentane and solvent was removed under reduced pressure. The recovered substrate **27** (24 mg, 0.181 mmol, 91%) was analyzed by ^{1}H NMR spectroscopy showing no isomerization took place (see Figure S8).

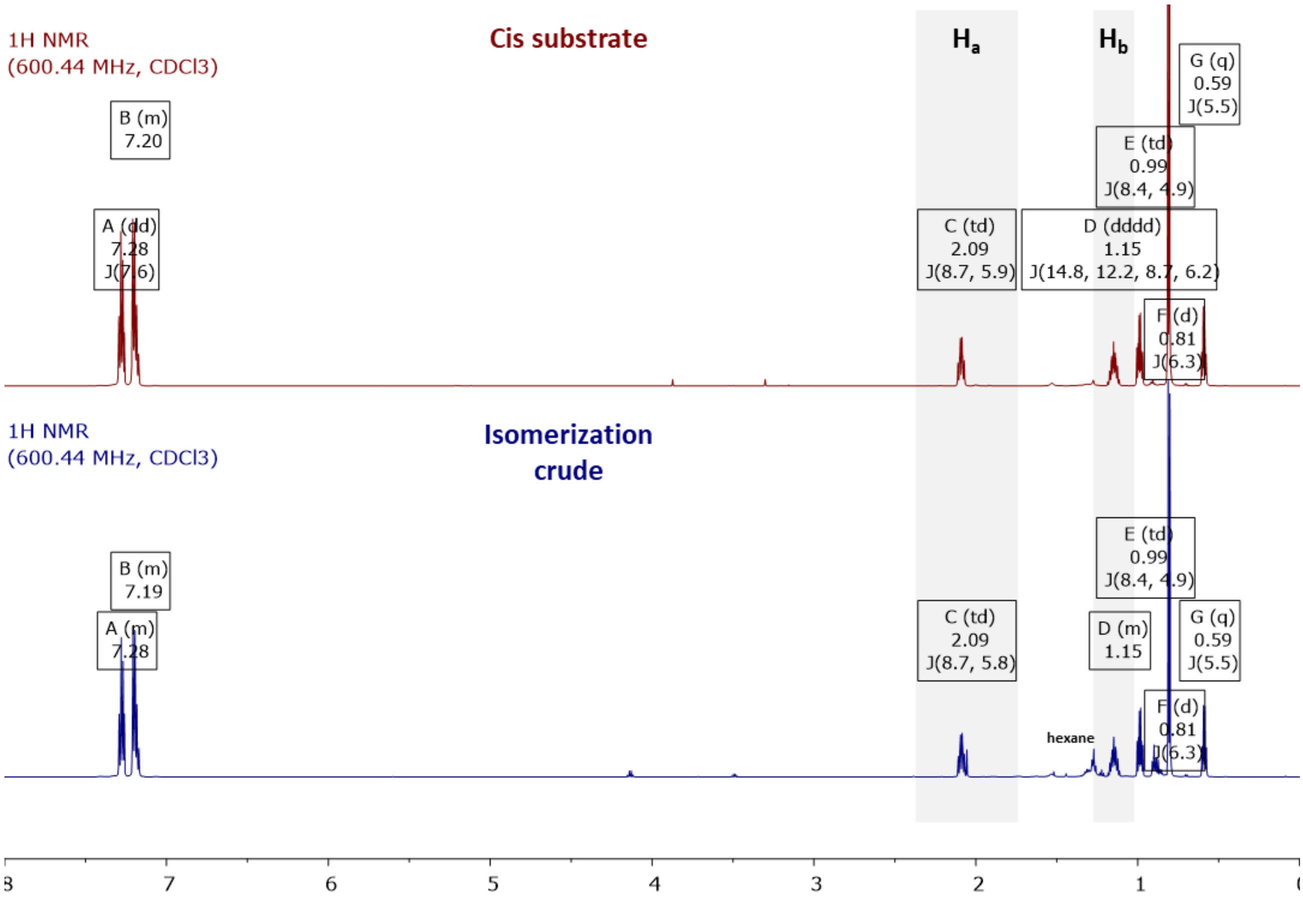

Figure S8. Isomerization study on (*cis*-2-methylcyclopropyl)benzene **27**: no isomerization was observed.

8. Enantioinvertive vinylcyclopropane isomerization

8.1. Synthesis and characterization of enantiopure vinylcyclopropanes

The enantiopure vinylcyclopropanes were synthesized according to reaction sequence shown below.

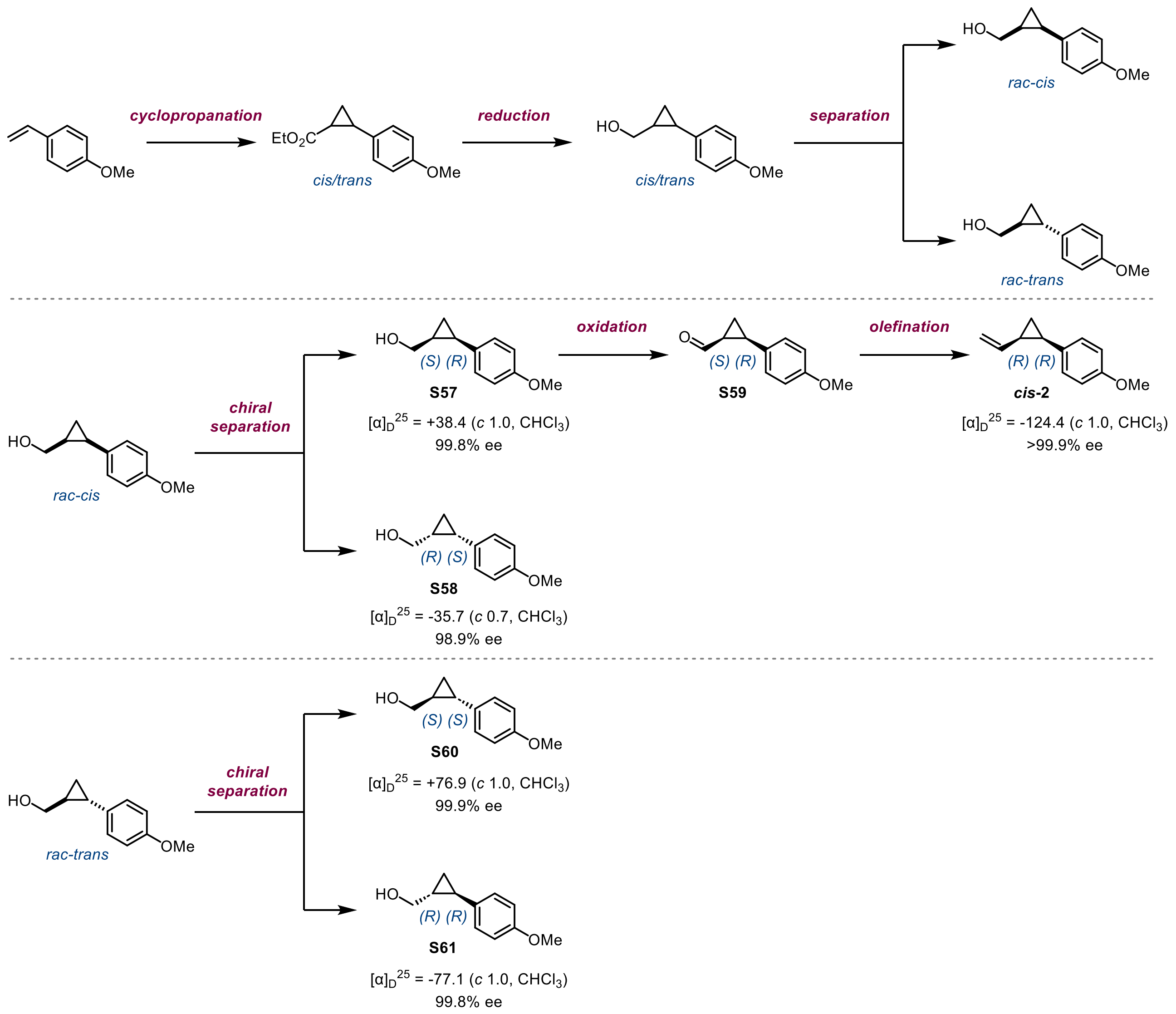

Ethyl 2-(4-methoxyphenyl)cyclopropane-1-carboxylate

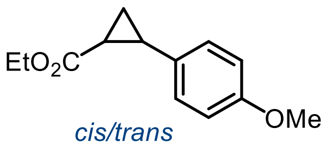

An oven dried flask equipped with a magnetic stirring bar was charged with 4-vinylanisole (30 mmol, 4.03 g, 1.0 equiv.), $Rh_2(OAc)_4$ (0.3 mmol, 133 mg, 1 mol%) and dry DCM (30 mL, 1 M). Next, a solution of diazo ethylacetate (3.42 g, 30 mmol, 1.0 equiv.) in DCM (15 ml, 2 M) was added via syringe pump and over the course of 12 hours. The green mixture was stirred for further 12 hours before it was filtered through a short pad of silica gel. The mixture was concentrated under reduced pressure and flash column chromatography (10:1 pentane:Et_2O) afforded the title product as a diastereomeric mixture as a yellowish solid (4.62 g, 21 mmol, 70%, 63:37 *trans*/*cis* (^{1}H NMR)). **R_f** (*trans*) = 0.30 (10:1 hexane:Et_2O). **R_f** (*cis*) = 0.22 (10:1 hexane:Et_2O). **^{1}H NMR** (600 MHz, $CDCl_3$) δ 7.21 – 7.16 (m, 2H, *cis*), 7.06 – 7.01 (m, 2H, *trans*), 6.84 – 6.78 (m, 2H *cis* + 2H *trans*), 4.17 (q, *J* = 7.1 Hz, 2H, *trans*), 3.89 (q, *J* = 7.1

Hz, 2H, *cis*), 3.78 (s, 3H, *trans*), 3.77 (s, 3H, *cis*), 2.56 – 2.45 (m, 1H *cis* + 1H *trans*), 2.03 (ddd, *J* = 9.2, 7.8, 5.6 Hz, 1H, *cis*), 1.82 (ddd, *J* = 8.4, 5.2, 4.1 Hz, 1H, *trans*), 1.69 – 1.62 (m, 1H, *cis*), 1.58 – 1.52 (m, 1H, *trans*), 1.35 – 1.28 (m, 1H, *cis*), 1.28 (t, *J* = 7.2 Hz, 3H, *trans*), 1.27 – 1.19 (m, 1H, *trans*), 1.02 (t, *J* = 7.1 Hz, 3H, *cis*). **^{13}C NMR** (151 MHz, $CDCl_3$) δ 173.7, 171.2, 158.5, 158.4, 132.2, 130.4, 128.7, 127.5, 114.0, 113.5, 60.8, 60.3, 55.5, 55.3, 25.8, 25.0, 24.0, 21.8, 16.9, 14.4, 14.3, 11.4. **MS** (70eV, EI): *trans isomer* (GC retention time 8.85 min), *m/z* (%): 220 (40) $[M]^+$, 191 (20), 175 (18), 163 (16), 147 (100), 131 (18), 115 (23), 91 (27). **MS** (70eV, EI): *cis isomer* (GC retention time 8.48 min), *m/z* (%): 220 (41) $[M]^+$, 191 (20), 175 (22), 163 (16), 147 (100), 131 (18), 115 (23), 91 (26). The data are in accordance with the data previous reported in literature.[26]

8.1.1. General procedure for reduction

Diisobutylaluminium hydride (48 mL, 1.0 M in toluene, 2.2 equiv.) was added dropwise over the course of 15 min to a stirred solution of ethyl 2-(4-methoxyphenyl)cyclopropane-1-carboxylate (4.62 g, 20.970 mmol, 1.0 equiv.) in DCM (25 mL, 1 M) at −78 °C. After completion of the addition the mixture was stirred for 1 h at 0 °C and HCl (1 M, 30 mL) was then slowly added. The aqueous layer was extracted with DCM (2 x 100 mL) and the combined organic extracts were dried over Na_2SO_4, filtered, and concentrated under reduced pressure. Filtration over a short plug of silica afforded the title product as a diastereomeric mixture. Purification by silica gel flash column chromatography (6:4:0.5 hexane:DCM:MeOH) afforded *cis*-2-(4-methoxyphenyl)cyclopropyl)methanol (600 mg, 3.370 mmol, 16%) as a colorless oil, *trans*-2-(4-methoxyphenyl)cyclopropyl)methanol (1.11 g, 6.220 mmol, 30%) as a white solid and a mixture of diastereomers (1.76 g, 9.860 mmol, 47%) as a colorless oil. Chiral separation of the pure fractions performed on preparative HPLC afforded the single enantiomers (*see chapter 13 for HPLC information).*

((1*S*,2*R*)-2-(4-methoxyphenyl)cyclopropyl)methanol (S57)

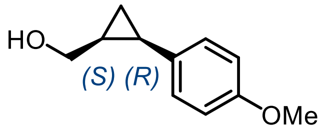

The title compound was obtained after enantiomeric separation of the corresponding *cis* racemate (600 mg, 3.370 mmol) as a colorless oil (213 mg, 1.2 mmol, 36%, 99.8% ee). **R_f** = 0.38 (6:4:0.5, hexane:DCM:MeOH). **$[\alpha]_D^{25}$** = +38.4 (*c* 1.0, $CHCl_3$). **^{1}H NMR** (600 MHz, $CDCl_3$) δ 7.17 (d, *J* = 8.4 Hz, 2H), 6.83 (d, *J* = 8.6 Hz, 2H), 3.79 (s, 3H), 3.47 (dd, *J* = 11.6, 6.3 Hz, 1H), 3.25 (dd, *J* = 11.6, 8.5 Hz, 1H), 2.23 (td, *J* = 8.4, 6.0 Hz, 1H), 1.44 (dtd, *J* = 14.4, 8.5, 5.8 Hz, 1H), 1.22 (s, 1H), 1.01 (td, *J* = 8.4, 5.2 Hz, 1H), 0.80 (q, *J* = 5.6 Hz, 1H). **^{13}C NMR** (151 MHz, $CDCl_3$) δ 158.2, 130.3, 130.0, 113.9, 63.1, 55.4, 20.7, 20.0, 7.8. **MS** (70eV, EI): *cis isomer* (GC retention time 7.893 min), *m/z* (%): 178.1 (21) $[M]^+$, 160.1 (13), 159.1 (15), 147.1 (100), 115.1 (25), 91.1 (34), 77.1 (12). **IR** (neat, cm^{-1}): 3364, 2936, 1886, 1611, 1511, 1459, 1410, 1294, 1243, 1177, 1026, 832, 798. **HRMS** (APCI): *m/z*: calculated for $[M+Na]^+$ $C_{11}H_{14}O_2Na$: 201.0886, found 201.0878.

Note: The absolute configuration was assigned by comparison of optical rotation with close analog (1*S*,2*R*)-2-phenylcyclopropylmethanol $[\alpha]_D^{25}$ = +38.4 (*c* 3.8, $CHCl_3$; e.r. 86:14).[27]

((1*R*,2*S*)-2-(4-methoxyphenyl)cyclopropyl)methanol (S58)

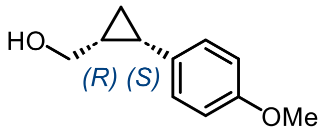

The title compound was obtained after enantiomeric separation of the corresponding *cis* racemate (600 mg, 3.370 mmol) as a colorless oil (236 mg, 1.3 mmol, 39%, 98.9% ee). **R_f** = 0.38 (6:4:0.5 hexane:DCM:MeOH). **$[\alpha]_D^{25}$** = –35.7 (*c* 0.7, $CHCl_3$). **^{1}H NMR** (600 MHz, $CDCl_3$) δ 7.17 (d, *J* = 8.4 Hz, 2H), 6.83 (d, *J* = 8.6 Hz, 2H), 3.79 (s, 3H), 3.47 (dd, *J* = 11.6, 6.3 Hz, 1H), 3.25 (dd, *J* = 11.6, 8.5 Hz, 1H), 2.23 (td, *J* = 8.4, 6.0 Hz, 1H), 1.44 (dtd, *J* = 14.4, 8.5, 5.8 Hz, 1H), 1.22 (s, 1H), 1.01 (td, *J* = 8.4, 5.2 Hz, 1H), 0.80 (q, *J* = 5.6 Hz, 1H). **^{13}C NMR** (151 MHz, $CDCl_3$) δ 158.2, 130.3, 130.0, 113.9, 63.1, 55.4, 20.7, 20.0, 7.8. **MS** (70eV, EI): *cis isomer* (GC retention time 7.893 min), *m/z* (%): 178.1 (21) $[M]^+$, 160.1 (13), 159.1 (15), 147.1 (100), 115.1 (25), 91.1 (34), 77.1 (12). **IR** (neat, cm^{-1}): 3365, 2930, 1883, 1611, 1511, 1459, 1410, 1294, 1243, 1177, 1027, 832, 798. **HRMS** (APCI): *m/z*: calculated for $[M+Na]^+$ $C_{11}H_{14}O_2Na$: 201.0886, found 201.0877.

Note: The absolute configuration was assigned by comparison of optical rotation with close analog (1*R*,2*S*)-2-phenylcyclopropylmethanol $[\alpha]_D^{25}$ = -30.1 (*c* 1.0, $CHCl_3$; e.r. 77.5:22.5).[28]

((1*S*,2*S*)-2-(4-methoxyphenyl)cyclopropyl)methanol (S60)

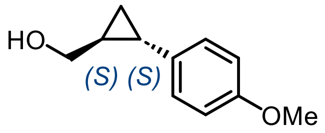

The title compound was obtained after enantiomeric separation of the corresponding *trans* racemate (1.11 g, 6.220 mmol) as a white solid (528 mg, 2.96 mmol, 48%, 99.9% ee). **R_f** = 0.35 (6:4:0..5 hexane:DCM:MeOH). **M.p.** = 57.9 – 59.3 °C. **$[\alpha]_D^{25}$** = +76.9 (*c* 1.0, $CHCl_3$). **^{1}H NMR** (600 MHz, $CDCl_3$) δ 7.01 (d, *J* = 8.6 Hz, 2H), 6.81 (d, *J* = 8.6 Hz, 2H), 3.78 (s, 3H), 3.65 – 3.57 (m, 2H), 1.79 (dt, *J* = 9.3, 4.9 Hz, 1H), 1.46 (s, 1H), 1.43 – 1.34 (m, 1H), 0.89 (ddt, *J* = 13.9, 8.8, 5.1 Hz, 2H). **^{13}C NMR** (151 MHz, $CDCl_3$) δ 157.9, 134.5, 127.1, 114.0, 66.8, 55.5, 24.9, 20.7, 13.4. **MS** (70eV, EI): *trans isomer* (GC retention time 8.045 min), *m/z* (%): 178.1 (25) $[M]^+$, 160.1 (16), 159.1 (17), 147.1 (100), 115.1 (26), 91.1 (31), 77.1 (11). **IR** (neat, cm^{-1}): 3304, 2927, 1613, 1511, 1458, 1361, 1291, 1248, 1178, 1113, 1074, 1026, 919, 885, 847, 814. **HRMS** (ESI): *m/z*: calculated for $[M]^+$ $C_{11}H_{14}O_2$: 178.0988, found 178.0990. The data are in agreement with those previously reported in the literature.[19]

Note: The absolute configuration was assigned by comparison of optical rotation with reported values $[\alpha]_D^{25}$ = +21.1 (*c* 0.4, $CHCl_3$; e.r. 99:1).[29]

((1*R*,2*R*)-2-(4-methoxyphenyl)cyclopropyl)methanol (S61)

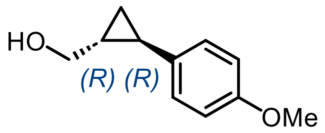

The title compound was obtained after enantiomeric separation of the corresponding *trans* racemate (1.11 g, 6.220 mmol) as a white solid (490 mg, 2.75 mmol, 44%, 99.8% ee). **R_f** = 0.35 (6:4:0.5 hexane:DCM:MeOH). **M.p.** = 57.9 – 59.3 °C. **$[\alpha]_D^{25}$** = –77.1 (*c* 1.0, $CHCl_3$). **^{1}H NMR** (600 MHz, $CDCl_3$) δ 7.01 (d, *J* = 8.6 Hz, 2H), 6.81 (d, *J* = 8.6 Hz, 2H), 3.78 (s, 3H), 3.61 (qd, *J* = 11.2, 6.8 Hz, 2H), 1.79 (dt, *J* = 9.3, 4.9 Hz, 1H), 1.47 (s, 1H), 1.39 (dtdd, *J* = 8.5, 6.8, 5.7, 4.5 Hz, 1H), 0.89 (ddt, *J* = 13.9, 8.8, 5.1 Hz, 2H). **^{13}C NMR** (151 MHz, $CDCl_3$) δ 157.9, 134.5, 127.1, 114.0, 66.8, 55.5, 24.9, 20.7, 13.4. **MS** (70eV, EI): *trans isomer* (GC retention time 8.045 min), *m/z* (%): 178.1 (25) $[M]^+$, 160.1 (16), 159.1 (17), 147.1 (100), 115.1 (26), 91.1 (31), 77.1 (11). **IR** (neat, cm^{-1}): 3305, 2929, 1613, 1512, 1458, 1361, 1291, 1249, 1179, 1112, 1074, 1026, 918, 885, 847, 813. **HRMS** (ESI): *m/z*: calculated for $[M]^+$ $C_{11}H_{14}O_2$: 178.0988, found 178.0990. The data are in agreement with those previously reported in the literature.[19]

Note: The absolute configuration was assigned by comparison of optical rotation with reported values $[\alpha]_D^{25}$ = -21.5 (*c* 0.4, $CHCl_3$; e.r. 99:1).[29]

8.1.2. General procedure for DMP oxidation

The cyclopropyl alcohol (1.0 equiv.) was weighed into an oven dried flask and dissolved in dry DCM (10 mL, 0.1 M). The solution was cooled to 0 °C before DMP (1.4 equiv.) was added and the reaction mixture was stirred for 1 h at 0 °C and then for 2 h at room temperature. The reaction was quenched by adding 10 mL of an aqueous 1:1 mixture of sat. $NaHCO_3$ and sat. $Na_2S_2O_3$ and the resulting mixture was stirred until both phases were clear. Next, the phases were separated, and the aqueous phase was extracted with DCM (3 x 10 mL). The combined organic layers were dried over Na_2SO_4, filtered, and concentrated under reduced pressure. The crude aldehydes were obtained quantitatively in high purity and were used directly for the next step without further purification.

(1*S*,2*R*)-2-(4-methoxyphenyl)cyclopropane-1-carbaldehyde (S59)

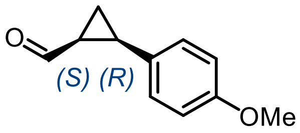

Following general procedure using ((1*S*,2*R*)-2-(4-methoxyphenyl)cyclopropyl)methanol **S57** (160.0 mg, 0.897 mmol). The title compound was obtained as a yellowish solid (156.6 mg, 0.888 mmol, 99%). **^{1}H NMR** (600 MHz, $CDCl_3$) δ 8.68 (d, *J* = 6.7 Hz, 1H), 7.23 (d, *J* = 8.3 Hz, 2H), 6.83 (dd, *J* = 8.5, 1.3 Hz, 2H), 3.79 (s, 3H), 2.77 (q, *J* = 8.1 Hz, 1H), 2.09 (tdd, *J* = 8.2, 6.7, 5.2 Hz, 1H), 1.83 (dt, *J* = 6.8, 5.3 Hz, 1H), 1.56 (td, *J* = 8.2, 5.5 Hz, 1H). **^{13}C NMR** (151 MHz, $CDCl_3$) δ 201.7, 158.8, 130.4, 127.9, 114.1, 55.4, 29.8, 26.0, 12.0. **IR** (neat, cm^{-1}): 2938, 2836, 1699, 1612, 1513, 1457, 1297, 1245, 1176, 1030, 946, 829. **HRMS** (EI): *m/z*: calculated for $[M]^+$ $C_{11}H_{12}O_2$: 176.0832, found: 176.0832. *Note:* The absolute configuration was assigned based on the employed starting material.

8.1.3. General procedure for Wittig olefination

An oven dried flask was charged with methyltriphenylphosphonium bromide (1.2 equiv.) before it was evacuated and backfilled with argon (3x). It was suspended in dry THF (10 mL, 0.1 M), cooled to 0 °C and ^{n}BuLi (1.1 equiv., 2.5 M in hexane) was added dropwise and the resulting mixture stirred for 30 min at 0 °C. Then, a solution of the cyclopropyl aldehyde (1.0 equiv., 0.5 M in THF) was added and the reaction mixture stirred for 1 h at 0 °C followed by 1 h at room temperature. The mixture was quenched by adding 5 mL of sat. aqueous NH_4Cl and phases were separated. The aqueous phase was extracted with DCM (2 x 10 mL). The combined organic layers were dried over Na_2SO_4, filtered, and concentrated under reduced pressure. The crude vinylcyclopropane was purified by silica column chromatography (50:1 pentane:Et_2O).

1-methoxy-4-((1*R*,2*R*)-2-vinylcyclopropyl)benzene (*cis*-2)

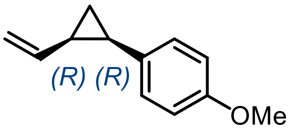

Following general procedure using (1*S*,2*R*)-2-(4-methoxyphenyl)cyclopropane-1-carbaldehyde **S59** (156.6 mg, 0.888 mmol). Column chromatography (50:1 pentane:Et_2O) afforded the title compound as a colorless oil (110.0 mg, 0.631 mmol, 71%, 99.9% ee). **R_f** = 0.38 (50:1 hexane:Et_2O). **$[\alpha]_D^{25}$** = –124.4 (*c* 1.0, $CHCl_3$). **^{1}H NMR** (400 MHz, $CDCl_3$) δ 7.17 – 7.10 (m, 2H), 6.86 – 6.79

(m, 2H), 5.14 – 5.06 (m, 2H), 4.89 – 4.81 (m, 1H), 3.79 (s, 3H), 2.29 (td, J = 8.5, 6.4 Hz, 1H), 1.88 – 1.75 (m, 1H), 1.23 (td, J = 8.4, 5.1 Hz, 1H), 0.96 (q, J = 5.5 Hz, 1H). **^{13}C NMR** (101 MHz, $CDCl_3$) δ 158.1, 138.6, 130.9, 130.3, 113.9, 113.6, 55.4, 22.8, 22.6, 12.0. **IR** (neat, cm^{-1}): 3074, 3002, 2953, 2834,1633, 1612, 1512, 1459, 1295, 1245, 1177, 1034, 985, 893, 831, 795. **MS** (70eV, EI): *cis isomer* (GC retention time 6.900 min), *m/z* (%): 174.1 (100) $[M]^+$, 173.1 (46), 159.1 (90), 158.1 (43), 145.1 (18), 144.1 (71), 143.1 (42), 131.1 (28), 128.1 (51), 115.1 (49), 108.1 (22), 91.1 (47), 77.1 (25), 65.1 (17). **HRMS** (EI): *m/z*: calculated for $[M]^+$ $C_{12}H_{14}O$: 174.1039, found: 174.1040. *Note:* The absolute configuration was assigned based on the employed starting material.

8.2. Stereoinversive isomerization and downstream derivatization

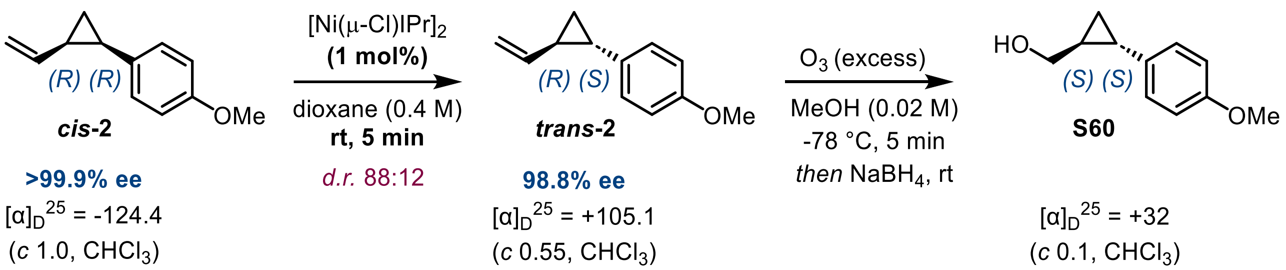

Isomerization. Inside an argon-filled glovebox, an oven dried 4 mL screw-cap vial equipped with a magnetic stirring bar was charged with 1-methoxy-4-((1*R*,2*R*)-2-vinylcyclopropyl)benzene ***cis*-2** (17.4 mg, 0.100 mmol, 1.0 equiv.), anhydrous 1,4-dioxane (0.25 mL, 0.4 M) and $[Ni(\mu\text{-}Cl)(IPr)]_2$ (1.0 mg, 1 mol%), in that order. The reaction vial was then sealed and allowed to stir inside the glovebox at the indicated temperature for the indicated time. Upon completion the reaction mixture was removed from the glovebox and quenched by the addition of wet pentane (*i.e.* technical grade pentane that had been distilled and stored on the bench). A spatula tip of ammonium pyrrolidine-1-dithiocarboxylic acid was added and the mixture stirred for additional 15 min to precipitate nickel.[13] Purification by filtration over a short silica plug washing with Et_2O and concentration under reduced pressure afforded ***trans*-2** as a low-melting solid (17.1 mg, 0.098 mmol, 98%, 12:88 *cis/trans* (^{1}H NMR), 98.8% ee). **$[\alpha]_D^{25}$** = +105.1 (*c* 0.55 $CHCl_3$).

Derivatization According to Taylor and coworkers,[30] a dry Schlenk tube was charged with ***trans*-2** (17.1 mg, 0.098 mmol, *from previous step*) was dissolved in dry MeOH (5 mL, 0.02 M) fitted with a stir bar and cooled to -78°C. Ozone was bubbled through the cooled solution until a blue color persisted in the flask (usually 5-10 min). Then, $NaBH_4$ (3.8 mg, 0.100 mmol) was added in one portion to the solution and the reaction was allowed to warm to room temperature. TLC analysis showed complete conversion of the starting material after 120 minutes. The mixture was diluted with Et_2O, filtered over a short silica plug washing with Et_2O and concentrated under reduced pressure. Purification by silica gel column chromatography (6:4:0.5 hexanes:EtOAc:MeOH) afforded ((*1S,2S*)-2-(4-methoxyphenyl)cyclopropyl) methanol) **S52** as a colorless oil (12.0 mg, 0.067 mmol, 69%). **$[\alpha]_D^{25}$** = +32 (*c* 0.1, $CHCl_3$). *Note:* Characterization data and optical rotation values match our previously prepared compound (see section 8.1.1).

9. Dynamic thermodynamic resolution

9.1. Thermodynamic resolution of vinyl cyclopropyl-Weinreb amide (23)

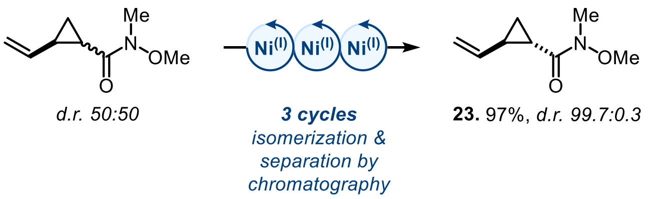

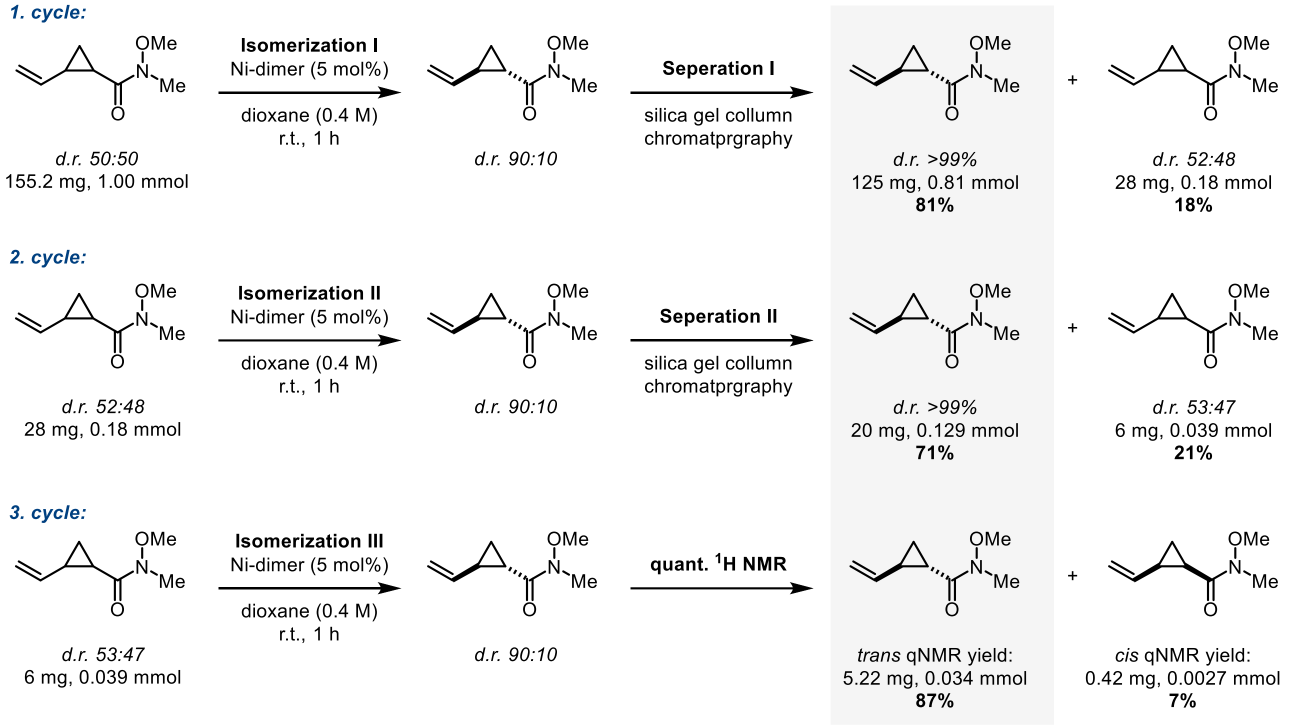

Step 1, isomerization: Inside an argon-filled glovebox *N*-methoxy-*N*-methyl-2-vinylcyclopropane-1-carboxamide **S23** (155.2 mg, 1.0 mmol, d.r. 50:50 (^{1}H NMR)) was dissolved in dioxane (2.5 mL, 0.4 M) and Nickel dimer (48 mg, 5 mol%) was added. The reaction mixture was stirred for 60 min at room temperature. The reaction mixture was removed from the glovebox and 6 mL Et_2O were added followed by 4 spatula tips of ammonium pyrrolidine-1-dithiocarboxylic acid and the mixture was stirred for additional 15 min to precipitate nickel.[13] The mixture was filtered through a plug of silica, rinsing with Et_2O and the filtrate was concentrated under reduced pressure affording the crude compound as a diastereomeric mixture (10:90 *cis/trans* (^{1}H NMR)).

Step 2, silica chromatography: The diastereomers were then separated by flash silica column chromatography (4:1 pentane:Et_2O) affording two main fractions: the pure *trans* diastereomer (125 mg, 0.81 mmol, 81%, d.r. >1:99 *cis/trans* (^{1}H NMR)) as a colorless oil and a mixture of both diastereomers (28 mg, 0.18 mmol, 18%, 48:52 *cis/trans* (^{1}H NMR)).

Iteration: The mixture of diastereomers was subjected to two additional iterations of isomerization and separation (3 cycles in total) and the *trans* enriched product fractions were combined to give **23** as a colorless oil (150.22 mg, 0.967 mmol, 97%, 1:99 *cis/trans* (^{1}H NMR)).

9.2. Thermodynamic resolution of vinyl-ACCA ester (37)

9.2.1. *trans*-enrichment by flash silica column chromatography

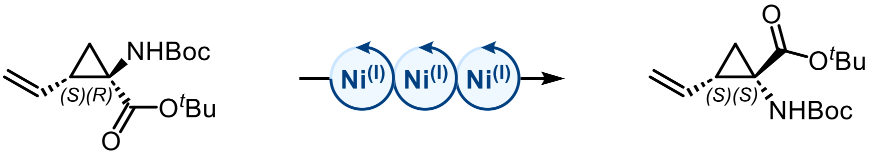

37. *d.r. 1:99*, >99% ee
1 g, 3.53 mmol

3 cycles
isomerization & separation by chromatography

38. 86%, *d.r. 99:1*, 99% ee

Step 1, isomerization: Inside an argon-filled glovebox *tert*-butyl (1*R*,2*S*)-1-((*tert*-butoxycarbonyl)amino)-2-vinylcyclopropane-1-carboxylate **37** (1.0 g, 3.53 mmol, >99% ee) was dissolved in dioxane (8.8 mL, 0.4 M) and Nickel dimer **1** (34 mg, 1 mol%) was added. The reaction mixture was stirred for 10 min at room temperature. The reaction mixture was removed from the glovebox and 6 mL Et_2O were added followed by 4 spatula tips of ammonium pyrrolidine-1-dithiocarboxylic acid and the mixture was stirred for additional 15 min to precipitate nickel.[13] The mixture was filtered through a plug of silica, rinsing with Et_2O and the filtrate was concentrated under reduced pressure affording the crude compound as a diastereomeric mixture as dark green solid.

Step 2, silica chromatography: The diastereomers were then separated by flash silica column chromatography (4:1 pentane:Et_2O) affording two main fractions: the pure (1*S*,2*S*)-diastereomer **38** as a white solid and a mixture of both diastereomers.

Iteration: The mixture of diastereomers was subjected to two additional iterations of isomerization and separation (3 cycles in total) and the (1*S*,2*S*)-enriched product fractions were combined to give *tert*-butyl (1*S*,2*S*)-1-((*tert*-butoxycarbonyl)amino)-2-vinylcyclopropane-1-carboxylate (**38**) as a white solid (861 mg, 3.04 mmol, 86%, 99% ee, 1:99 *cis*/*trans* (^{1}H NMR)).

9.2.2. *trans*-enrichment by crystallization

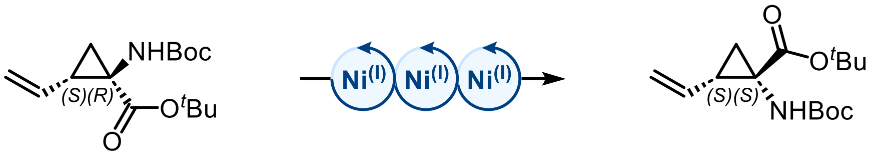

37. *d.r. 1:99*, >99% ee
1 g, 3.53 mmol

3 cycles
isomerization & separation by crystallization

38. 91%, *d.r. 99:1*, >99% ee

Step 1, isomerization: Inside an argon-filled glovebox *tert*-butyl (1*R*,2*S*)-1-((*tert*-butoxycarbonyl)amino)-2-vinylcyclopropane-1-carboxylate **37** (1.0 g, 3.5 mmol, >99% ee) was dissolved in dioxane (8.8 mL, 0.4 M) and Nickel dimer (34 mg, 1 mol%) was added. The reaction mixture was stirred for 10 min at room temperature. The reaction mixture was removed from the glovebox and 6 mL Et_2O were added followed by 4 spatula tips of ammonium pyrrolidine-1-dithiocarboxylic acid and the mixture was stirred for additional 15 min to precipitate nickel.[13] The mixture was filtered through a

plug of silica, rinsing with Et_2O and the filtrate was concentrated under reduced pressure affording the crude compound as a diastereomeric mixture as dark green solid.

Step 2, crystallization: The crude was dissolved in refluxing hexane (100 mg/1 mL) for 15 minutes and let cool down to room temperature before it was stored in the fridge overnight. The mother liquid was decantated and the solid washed with a minimum amount of hexane (2x1 mL) to afford the (1*S*,2*S*)-diastereomer **38** as colourless crystals. The combined hexane phases were concentrated to afford the crude compound as a diastereomeric mixture.

Iteration: The mixture of diastereomers was subjected to two additional cycles of isomerization and separation (3 cycles in total) and the crystallized product portions were combined to give *tert*-butyl (1*S*,2*S*)-1-((*tert*-butoxycarbonyl)amino)-2-vinylcyclopropane-1-carboxylate (**38**) as colourless crystals (905 mg, 3.19 mmol, 91%, >99% ee, 1:99 *cis*/*trans* (^{1}H NMR)).

10. Synthesis of *trans*-divinylcyclopropane starting materials

10.1. Synthesis of (1*S*,2*S*)-1-((*E*)-hex-1-en-1-yl)-2-vinylcyclopropane [(-)-dictyopterene A] (39)

1,3-dioxoisoindolin-2-yl (1*R*,2*S*)-2-((*E*)-hex-1-en-1-yl)cyclopropane-1-carboxylate (*S*39)

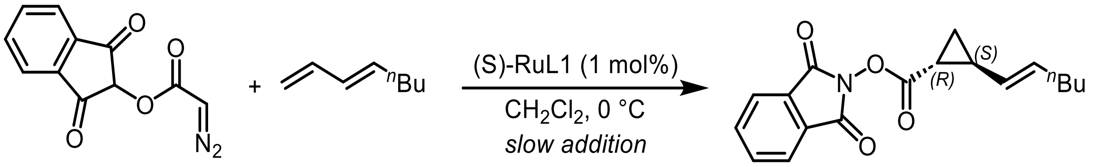

Prepared according to literature procedure.[31] Under argon atmosphere, (*E*)-octa-1,3-diene[31] (808.0 mg, 6.6 mmol, 1.0 equiv.) and (*S*)-RuL[31] (41.7 mg, 1 mol%, 0.01 equiv.) were dissolved in dry DCM (27 mL) and cooled to 0 °C. A solution of 1,3-dioxoisoindolin-2-yl 2-diazoacetate[31] (1.75 g, 7.6 mmol, 1.2 equiv.) in dry DCM (40 mL) was added over the course of 40 minutes using a syringe pump and it was stirred for 1.5 h. The volatiles were removed under reduced pressure and the crude was purified by flash silica gel column chromatography (6:1 pentane:EtOAc) to afford the title product **S39** as a colorless oil (1.72 g, 5.5 mmol, 83%, 7:1 *trans*/*cis* (^{1}H NMR)). **R_f** = 0.4 (6:1 pentane:EtOAc). **$[\alpha]_D^{25}$** = -216.9 (*c* = 1.0, $CHCl_3$). **^{1}H NMR** (400 MHz, $CDCl_3$) δ 7.91 – 7.85 (m, 2H *cis* + 2H *trans*), 7.81 – 7.76 (m, 2H *cis* + 2H *trans*), 5.78 (dd, *J* = 14.9, 7.3 Hz, 1H, *cis*), 5.69 (dt, *J* = 14.5, 6.8 Hz, 1H, *trans*), 5.42 – 5.30 (m, 1H, *cis*), 5.08 (dd, *J* = 15.3, 8.0 Hz, 1H, *trans*), 2.27 – 2.17 (m, 1H *cis* + 1H *trans*), 2.06 – 1.97 (m, 2H *cis* + 2H *trans*), 1.90 (dt, *J* = 8.7, 4.5 Hz, 1H, *trans*), 1.56 (dq, *J* = 9.3, 4.9 Hz, 1H, *trans*), 1.49 – 1.42 (m, 1H, *cis*), 1.43 – 1.28 (m, 4H *cis* + 4H *trans*), 1.29 (m, 1H, *trans*), 0.96 – 0.83 (m, 3H *cis* + 3H *trans*). **^{13}C NMR** (151 MHz, $CDCl_3$) δ 170.1, 168.5, 162.2, 135.2, 134.9, 134.9, 133.4, 129.1, 129.1, 128.1, 124.6, 124.1, 124.0, 32.4, 32.2, 31.6, 31.5, 29.8, 27.3, 26.3, 22.3, 22.3, 18.9, 17.8, 17.5, 16.0, 14.1. The data are in agreement with those previously reported in the literature.[31]

(1*S*,2*S*)-1-((*E*)-hex-1-en-1-yl)-2-vinylcyclopropane [(-)-Dictyopterene A] (39)

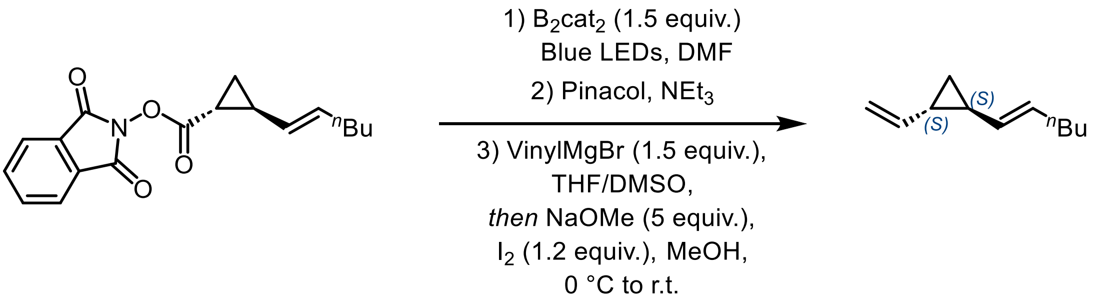

Prepared according to literature procedure.[31] In a glovebox, ten 8 mL vials were charged with a magnetic stirring bar, redox active ester **S39** (63.0 mg, 0.2 mmol, 1.0 equiv.) and B_2cat_2 (71.3 mg, 0.3 mmol, 1.5 equiv.) and degassed DMF (2.0 mL, 0.1 M). The vials were capped and stirred under blue LED lights for 14 hours. After cooling down to room temperature, a solution of pinacol (94.5 mg, 0.8 mmol, 4.0 equiv.) in Et_3N (0.7 mL) was added to each vial and stirred for 1.5 h. Then, all ten reaction mixtures were combined and diluted with pentane, water and sat. NH_4Cl. The aqueous phase was extracted with pentane (2x), the combined organic phases dried over Na_2SO_4, filtered, and concentrated to give the

crude cyclopropylboronate, which was then dissolved in dry THF/DMSO (1:1, 15 mL in total) under Argon. The solution was cooled to 0 °C and stirred for 10 min. Then, vinyl magnesium bromide (3.75 mL, 0.8 M in THF, 3.0 mmol, 1.5 equiv.) was added dropwise and the mixture was stirred for another 10 min at 0 °C and then stirred at r.t. for 50 min. The mixture was then cooled to 0°C and a solution of NaOMe in MeOH (1.9 mL, 5.4 M in MeOH, 10 mmol, 5.0 equiv.) was added. Subsequently, a solution of iodine in MeOH (6 mL, 0.5 M in MeOH, 3.0 mmol, 1.5 equiv.) was added dropwise. After the addition was completed, the mixture was stirred for further 30 minutes at 0 °C. Then, aqueous $Na_2S_2O_3$ was added to quench the reaction. Subsequently, pentane was added, and phases were separated. The aqueous phase was extracted pentane (2x). The combined organic phases were dried over Na_2SO_4, filtered, and concentrated. The crude was purified by flash silica gel column chromatography (pentane) to give the title product **39** as a colorless oil (50.0 mg, 0.330 mmol, 17%, 88% ee). **R_f** = 0.9 (pentane). **$[\alpha]_D^{25}$** = -74.6 (*c* = 0.27, $CHCl_3$). **^{1}H NMR** (400 MHz, $CDCl_3$) δ 5.49 (dt, *J* = 15.2, 6.8 Hz, 1H), 5.44 – 5.34 (m, 1H), 5.09 – 4.96 (m, 2H), 4.86 (dd, *J* = 10.2, 1.7 Hz, 1H), 1.97 (tt, *J* = 7.1, 3.9 Hz, 2H), 1.43 – 1.35 (m, 2H), 1.35 – 1.29 (m, 4H), 0.95 – 0.85 (m, 3H), 0.83 – 0.72 (m, 2H). **^{13}C NMR** (151 MHz, CDCl3) δ 141.0, 131.7, 129.3, 112.0, 32.3, 31.9, 24.4, 23.7, 22.4, 14.9, 14.1. The data are in agreement with those previously reported in the literature.[31]

10.2. General Procedure G: Pd(I) catalyzed synthesis of *trans*-divinylcyclopropanes

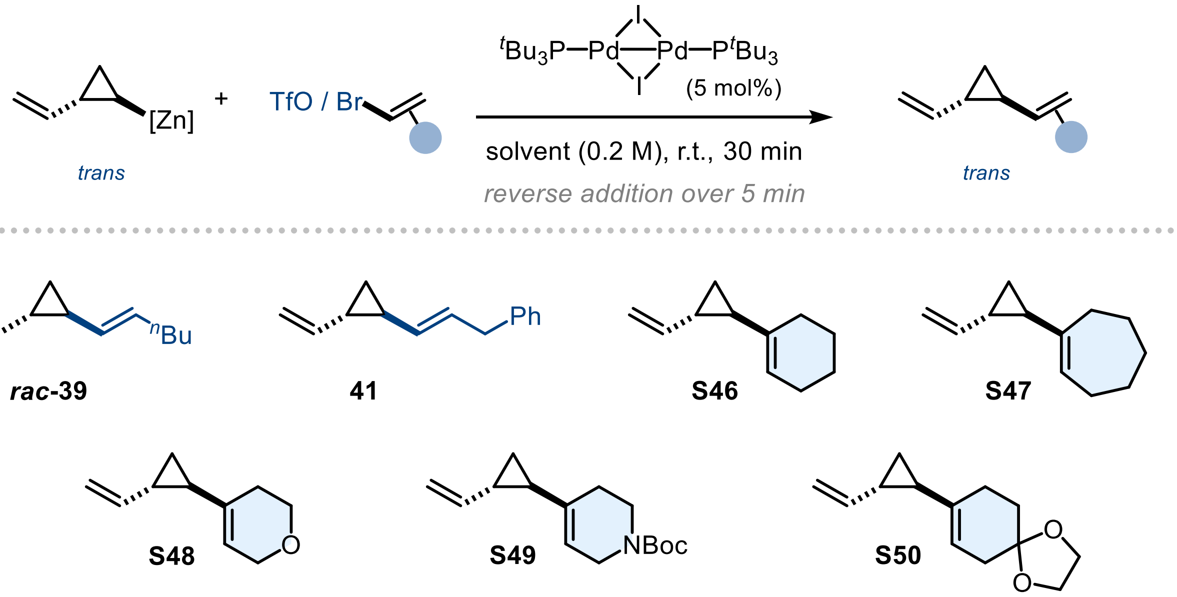

***rac*-39** **41** **S46** **S47**

S48 **S49** **S50**

Synthesized according to modified literature procedure.[6] In an argon filled glovebox, vinyl (pseudo)halide (1.0 equiv.) was placed into an oven dried 4 mL screw cap vial equipped with a magnetic stir bar. It was dissolved in dry toluene or THF or NMP (0.2 M) and Pd(I)-iodo-dimer (5 mol%) was added. The vial was sealed, brought outside and connected to the Schlenk line. An oven dried round bottom flask equipped with a magnetic stirring bar was evacuated and backfilled with argon (3x) and the freshly prepared solution of *trans*-vinylcyclopropyl zincate was added to the flask. Next, the prepared Palladium vinyl halide solution was added slowly to the organozincate over a course of 5 min using a syringe pump. The reaction mixture was stirred for additional 30 min, before it was quenched by the addition of wet pentane or hexane. A spatula tip of ammonium pyrrolidine-1-dithiocarboxylic acid was added and the mixture was stirred for additional 15 min to precipitate palladium.[13] The

mixture was filtered through a plug of silica, washing with Et_2O and the filtrate was concentrated under reduced pressure. The crude material was further purified by silica gel column chromatography.

10.2.1. Characterization data of *trans*-divinylcyclopropanes

trans-1-((_E_)-hex-1-en-1-yl)-2-vinylcyclopropane [(_rac_)-dictyopterene A] (_rac_-39)

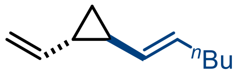

Prepared following general procedure G using (*E*)-1-bromohex-1-ene[6] (81.5 mg, 0.5 mmol, 1.0 equiv.), freshly prepared (*trans*-2-vinylcyclopropyl)zinc(II) chloride (1.0 mmol, 2.0 equiv.) and dry THF (0.2 M). Flash column chromatography (pentane) afforded the title product ***rac*-39** as a colorless liquid (50.3 mg, 0.335 mmol, 67%). $\mathbf{R_f}$ = 0.8 (pentane). **^{1}H NMR** (400 MHz, $CDCl_3$) δ 5.50 (dt, *J* = 15.1, 6.8 Hz, 1H), 5.40 (ddd, *J* = 18.3, 10.2, 8.2 Hz, 1H), 5.09 – 4.96 (m, 2H), 4.86 (dd, *J* = 10.3, 1.6 Hz, 1H), 1.98 (qd, *J* = 7.1, 1.5 Hz, 2H), 1.43 – 1.25 (m, 8H, overlap with pentane), 0.95 – 0.88 (m, 1H, overlap with pentane), 0.84 – 0.74 (m, 2H). **^{13}C NMR** (101 MHz, $CDCl_3$) δ 141.0, 131.7, 129.3, 112.0, 32.3, 31.9, 24.4, 23.7, 22.4, 14.9, 14.1. **MS** (70eV, EI): *m/z* (%): 150 (1) [M^+], 122 (1), 108 (3), 91 (34), 79 (100), 66 (15). *Note: Residual pentane could not be completely removed due to the volatility of the product.* The data are in agreement with those previously reported in the literature.[32]

((_E_)-3-(_trans_-2-vinylcyclopropyl)allyl)benzene (41)

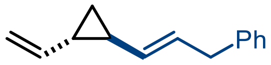

Prepared following general procedure G using (*E*)-(3-bromoallyl)benzene[6] (492.0 mg, 2.5 mmol, 1.0 equiv.), freshly prepared (*trans*-2-vinylcyclopropyl)zinc(II) chloride (5.0 mmol, 2.0 equiv.) and dry toluene (12.5 mL, 0.2 M). Flash column chromatography (pentane → 100:1 pentane:Et_2O) afforded the title product **41** as a colorless liquid (281.0 mg, 1.525 mmol, 61%). $\mathbf{R_f}$ = 0.5 (100:1 pentane:Et_2O, PMA). **^{1}H NMR** (600 MHz, $CDCl_3$) δ 7.32 – 7.26 (m, 2H), 7.22 – 7.16 (m, 3H), 5.66 (dt, *J* = 15.2, 6.9 Hz, 1H), 5.40 (ddd, *J* = 16.9, 10.3, 8.3 Hz, 1H), 5.12 (ddt, *J* = 15.2, 8.2, 1.5 Hz, 1H), 5.05 (dd, *J* = 17.1, 1.6 Hz, 1H), 4.87 (dd, *J* = 10.2, 1.7 Hz, 1H), 3.33 (d, *J* = 6.8 Hz, 2H), 1.45 – 1.39 (m, 2H), 0.84 – 0.80 (m, 2H). **^{13}C NMR** (151 MHz, $CDCl_3$) δ 140.9, 140.8, 133.4, 128.6, 128.5, 127.6, 126.1, 112.2, 39.0, 24.5, 23.7, 15.0. **HRMS** (EI): *m/z*: calculated for $[M]^+$ $C_{14}H_{16}$: 184.1247, found: 184.1247.

1-((_trans_)-2-vinylcyclopropyl)cyclohex-1-ene (S46)

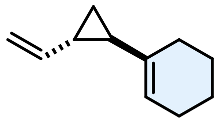

Prepared following general procedure G using 1-bromocyclohex-1-ene (161.0 mg, 1.0 mmol, 1.0 equiv.), freshly prepared (*trans*-2-vinylcyclopropyl)zinc(II) chloride (1.5 mmol, 1.5 equiv.) and dry THF (5.0 mL, 0.2 M). Flash silica gel column chromatography (pentane) afforded the title product **S46** as a colorless liquid (38.0 mg, 0.205 mmol, 20%, 80wt % in pentane). $\mathbf{R_f}$ = 0.8 (pentane, PMA). **^{1}H NMR** (600 MHz, $CDCl_3$) δ 5.44 (ddd, *J* = 17.0, 10.2, 8.6 Hz, 2H), 5.04 (dd, *J* = 17.1, 1.5 Hz, 1H), 4.85 (dd, *J* = 10.3, 1.7 Hz, 1H), 2.01 – 1.95 (m, 2H), 1.88 – 1.81 (m, 2H), 1.64 – 1.56 (m, 2H), 1.58 – 1.52 (m, 2H), 1.45 (tt, *J* = 8.8, 4.9 Hz, 1H), 1.29 – 1.20 (m, 1H), 0.93 (ddd, *J* = 8.5, 6.0, 4.6 Hz, 1H), 0.67 (dt, *J* = 8.7, 5.0 Hz, 1H). **^{13}C NMR** (151 MHz, $CDCl_3$) δ 141.8, 136.9, 120.4, 111.8, 27.7, 26.8, 25.3, 23.0, 22.8, 22.5, 12.6. **HRMS** (EI): *m/z* $[M]^+$ calculated for $C_{11}H_{16}$: 148.1252, found 148.1253. *Note: Residual pentane could not be completely removed due to the volatility of the product.*

1-((*cis*)-2-vinylcyclopropyl)cyclohept-1-ene (S47)

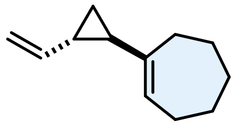

Prepared following general procedure G using 1-bromocyclohept-1-ene (87.5 mg, 0.500 mmol, 1.0 equiv.), freshly prepared (*trans*-2-vinylcyclopropyl)zinc(II) chloride (1.0 mmol, 2.0 equiv.) and dry THF (2.5 mL, 0.2 M). Flash column chromatography (pentane) afforded the title product **S47** as a colorless liquid (34.0 mg, 0.210 mmol, 42%). $\mathbf{R_f}$ = 0.7 (hexane, PMA). **^{1}H NMR** (400 MHz, $CDCl_3$) δ 5.59 (t, *J* = 6.6 Hz, 1H), 5.50 – 5.39 (m, 1H), 5.04 (dd, *J* = 17.1, 1.7 Hz, 1H), 4.85 (dd, *J* = 10.3, 1.7 Hz, 1H), 2.08 (td, *J* = 7.0, 3.5 Hz, 2H), 1.99 – 1.95 (m, 2H), 1.72 (p, *J* = 5.9 Hz, 2H), 1.49 – 1.36 (m, 6H), 0.87 (ddd, *J* = 8.0, 6.4, 4.7 Hz, 1H), 0.68 (dt, *J* = 7.9, 5.1 Hz, 1H). **^{13}C NMR** (101 MHz, $CDCl_3$) δ 143.8, 141.6, 125.5, 111.8, 32.8, 30.7, 28.9, 28.3, 27.4, 27.0, 22.4, 12.7. **IR** (neat, cm^{-1}): 3078, 3000, 2920, 2849, 2690, 2489, 2330, 2156, 2107, 2001, 1966, 1725, 1634, 1446, 1351, 1274, 1220, 1073, 1016, 984, 892, 838, 793, 764, 723, 666. **HRMS** (EI): *m/z*: calculated for $[M]^+$ $C_{12}H_{18}$: 162.1403, found: 162.1405. *Note: Caution, compound is volatile.*

4-((*trans*)-2-vinylcyclopropyl)-3,6-dihydro-2*H*-pyran (S48)

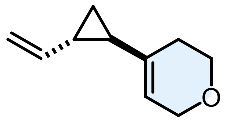

Prepared following general procedure G using 4-bromo-3,6-dihydro-2*H*-pyran (81.5 mg, 0.500 mmol, 1.0 equiv.), freshly prepared (*trans*-2-vinylcyclopropyl)zinc(II) chloride (1.0 mmol, 2.0 equiv.) an dry THF (2.5 mL, 0.2 M). Flash column chromatography (pentane) afforded the title product **S48** as a colorless oil (136.0 mg, 0.280 mmol, 56%). $\mathbf{R_f}$ = 0.12 (pentane, PMA). **^{1}H NMR** (600 MHz, $CDCl_3$) δ 5.48 – 5.39 (m, 2H), 5.06 (dd, *J* = 17.0, 1.7 Hz, 1H), 4.88 (dd, *J* = 10.3, 1.6 Hz, 1H), 4.11 (dt, *J* = 2.6, 1.3 Hz, 2H), 3.77 (t, *J* = 5.5 Hz, 2H), 2.04 – 1.92 (m, 2H), 1.49 (td, *J* = 8.5, 4.3 Hz, 1H), 1.38 (dt, *J* = 9.8, 5.3 Hz, 1H), 0.96 (ddd, *J* = 8.6, 5.9, 4.8 Hz, 1H), 0.74 (dt, *J* = 8.8, 5.1 Hz, 1H). **^{13}C NMR** (151 MHz, $CDCl_3$) δ 141.1, 135.2, 119.0, 112.3, 65.7, 64.4, 27.1, 26.7, 22.6, 12.6. **IR** (neat, cm^{-1}): 2924, 2252, 2075, 1704, 1456, 1377, 1197, 1101, 913, 731. **HRMS** (EI): *m/z* $[M]^+$ calculated for $C_{10}H_{14}O$: 150.1045, found 150.1036.

***tert*-butyl 4-((*trans*)-2-vinylcyclopropyl)-3,6-dihydropyridine-1(2*H*)-carboxylate (S49)**

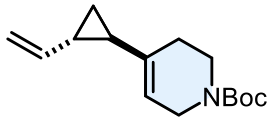

Prepared following general procedure G using *tert*-butyl 4-(((trifluoro-methyl)sulfonyl)oxy)-3,6-dihydropyridine-1(2*H*)-carboxylate (165.6 mg, 0.500 mmol, 1.0 equiv.), freshly prepared (*trans*-2-vinylcyclopropyl)zinc(II) chloride (1.0 mmol, 2.0 equiv.) and dry NMP (2.5 mL, 0.2 M). NMP was removed by flash column chromatography (5:1 pentane:Et_2O). Final flash column chromatography (5:1 pentane:Et_2O) afforded the title product **S49** as a colorless liquid (64.0 mg, 0.260 mmol, 52%). $\mathbf{R_f}$ = 0.4 (5:1 pentane:Et_2O, PMA). **^{1}H NMR** (600 MHz, $CDCl_3$) δ 5.43 (ddd, *J* = 17.4, 10.2, 8.6 Hz, 1H), 5.36 (s, 1H), 5.05 (dd, *J* = 17.1, 1.6 Hz, 1H), 4.88 (dd, *J* = 10.3, 1.6 Hz, 1H), 3.85 (s, 2H), 3.47 (t, *J* = 5.8 Hz, 2H), 1.96 (s, 2H), 1.46 (s, 10H), 1.41 – 1.36 (m, 1H), 0.94 (ddd, *J* = 8.7, 5.9, 4.9 Hz, 1H). **^{13}C NMR** (101 MHz, $CDCl_3$) δ 155.0, 141.0, 136.1 (br), 117.2 (br), 112.4, 79.6, 43.4 (br), 28.6, 26.7, 22.6, 12.7. **IR** (neat, cm^{-1}): 3079, 2974, 2925, 2837, 2669, 2332, 2083, 1994, 1694, 1638, 1537, 1453, 1415, 1365, 1283, 1240, 1166, 1110, 1027, 983, 959, 897, 864, 796, 768, 711. **HRMS** (EI): *m/z* $[M\text{-}tBu]^+$ calculated for $C_{11}H_{14}NO_2$: 192.1025, found 192.1021.

8-((*trans*)-2-vinylcyclopropyl)-1,4-dioxaspiro[4.5]dec-7-ene (S50)

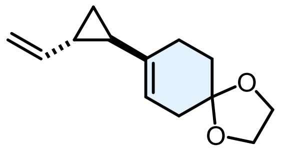

Prepared following general procedure G using 1,4-dioxaspiro[4.5]dec-7-en-8-yl trifluoromethanesulfonate[7] (144.1 mg, 0.500 mmol, 1.0 equiv.), freshly prepared (*trans*-2-vinylcyclopropyl)zinc(II) chloride (1.0 mmol, 2.0 equiv.) and dry NMP (2.5 mL, 0.2 M). NMP was removed by flash column chromatography (10:1 pentane:Et_2O). Final flash column chromatography (10:1 pentane:Et_2O) afforded the title product **S50** as a colorless liquid (58.0 mg, 0.357 mmol, 71%). $\mathbf{R_f}$ = 0.4 (10:1 pentane:Et_2O, PMA). **^{1}H NMR** (600 MHz, $CDCl_3$) δ 5.42 (ddd, *J* = 17.0, 10.3, 8.5 Hz, 1H), 5.34 (ddt, *J* = 3.9, 2.4, 1.3 Hz, 1H), 5.03 (dd, *J* = 17.1, 1.6 Hz, 1H), 4.85 (dd, *J* = 10.2, 1.7 Hz, 1H), 3.97 (s, 4H), 2.28 – 2.22 (m, 2H), 2.10 (tt, *J* = 6.4, 1.9 Hz, 2H), 1.75 (t, *J* = 6.5 Hz, 2H), 1.48 (ddt, *J* = 13.5, 8.7, 4.3 Hz, 1H), 1.38 (dt, *J* = 9.9, 5.3 Hz, 1H), 0.94 (ddd, *J* = 8.4, 6.1, 4.9 Hz, 1H), 0.71 (dt, *J* = 8.6, 5.0 Hz, 1H). **^{13}C NMR** (101 MHz, $CDCl_3$) δ 141.4, 136.7, 117.4, 112.1, 108.3, 64.5, 35.7, 31.2, 26.9, 26.0, 22.8, 13.0. **IR** (neat, cm^{-1}): 3077, 2882, 2673, 2331, 2236, 2211, 2087, 1997, 1923, 1800, 1634, 1550, 1474, 1429, 1367, 1339, 1305, 1250, 1211, 1115, 1055, 1013, 988, 948, 896, 862, 795, 757, 702, 658. **HRMS** (ESI): *m/z*: calculated for $[M+Na]^+$ $C_{13}H_{18}O_2Na$: 229.1199, found: 229.1205.

10.3. Synthesis of ((*trans*-2-vinylcyclopropyl)methylene)cyclohexane (51)

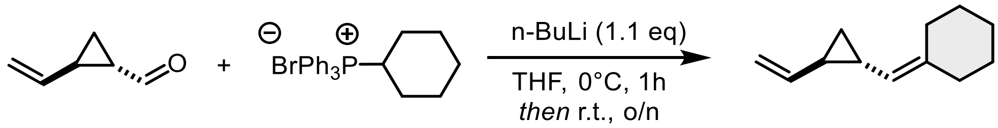

Under argon, an oven dried flask was charged with cyclohexyltriphenylphosphonium bromide (510.4 mg, 1.2 mmol, 1.2 equiv.) and suspended in dry THF (10 mL, 0.1 M) and cooled to 0°C. Then, *n*-BuLi (0.44 mL, 1.1 mmol, 1.1 equiv.) was added dropwise and the reaction mixture was stirred at 0°C for 30 min. A solution of 2-vinylcyclopropane-1-carbaldehyde[33] (115.3 mg, 1.0 mmol, 1.0 equiv.) in THF (0.5 M) was added and it was stirred at 0°C for 1h and then overnight. At the next day, the reaction was quenched by addition of sat. NH_4Cl (10 mL) and the aqueous phayse was extracted with Et_2O (2x20 mL). Combined organic layers were dried over Na_2SO_4, filtered and carefully concentrated under reduced pressure (600 mbar). Flash column chromatography (pentane) afforded the title product **51** as a colorless oil (122.0 mg, 0.750 mmol, 75%, 93:7 *trans*/*cis* (^{1}H NMR)). $\mathbf{R_f}$ = 0.8 (pentane). **^{1}H NMR** (600 MHz, $CDCl_3$) δ 5.55 (ddd, *J* = 17.1, 10.3, 9.0 Hz, 1H, *cis*), 5.43 (ddd, *J* = 17.0, 10.3, 8.6 Hz, 1H, *trans*), 5.11 (dd, *J* = 17.0, 2.0 Hz, 1H, *cis*), 5.04 (dd, *J* = 17.1, 1.6 Hz, 1H, *trans*), 4.97 (dd, *J* = 10.2, 2.0 Hz, 1H, *cis*), 4.85 (dd, *J* = 10.3, 1.7 Hz, 1H, *trans*), 4.81 – 4.78 (m, 1H, *cis*), 4.55 (d, *J* = 8.9 Hz, 1H, *trans*), 2.28 – 2.19 (m, 2H *cis*, 2H *trans*), 2.04 (td, *J* = 6.0, 1.3 Hz, 2H *cis*, 2H *trans*), 1.76 (qd, *J* = 8.5, 5.9 Hz, 1H, *cis*), 1.65 (qd, *J* = 8.7, 5.6 Hz, 1H, *cis*), 1.59 – 1.47 (m, 7H, *trans*, 6H *cis*), 1.34 (tt, *J* = 8.7, 4.7 Hz, 1H, *trans*), 1.10 (td, *J* = 8.3, 4.7 Hz, 1H, *cis*), 0.83 (dt, *J* = 8.5, 4.9 Hz, 1H, *trans*), 0.71 (ddd, *J* = 8.2, 5.5, 4.5 Hz, 1H, *trans*), 0.47 (td, *J* = 5.8, 4.7 Hz, 1H, *cis*). **^{13}C NMR** (151 MHz, $CDCl_3$) δ 141.8 (*cis*), 141.3 (*trans*), 139.7 (*trans*), 138.9 (*cis*), 123.6 (*trans*), 119.9 (*cis*), 114.0 (*cis*), 111.8 (*trans*), 37.1 (*cis*), 37.0 (*trans*), 29.4 (*trans*), 29.4 (*cis*), 28.8 (*cis*), 28.6 (*trans*), 27.9 (*cis*), 27.8 (*trans*), 27.0 (*cis*), 27.0 (*trans*), 26.0 (*cis*), 24.6 (*trans*), 19.8 (*trans*), 17.1 (*cis*), 15.5 (*trans*), 14.9 (*cis*). **IR** (neat, cm^{-1}): 3075, 2998, 2925, 2852, 2664, 2327, 2061, 2010, 1975, 1913, 1792, 1634, 1557, 1444, 1341, 1295, 1263, 1233, 1198, 1152, 1072, 1037, 984, 926, 892, 857, 801, 695, 664. **HRMS** (EI): *m/z* $[M]^+$ calculated for $C_{12}H_{18}$: 162.1403, found 162.1404. *Note: Residual solvent could not be completely removed due to the volatility of the product.*

10.4. Synthesis of *trans*-vinylcyclopropyl silyl enol ethers

10.4.1.Synthesis and characterization data of *trans*-vinylcyclopropylketones

The following compounds were synthesized according to General procedure C (details on page S18).

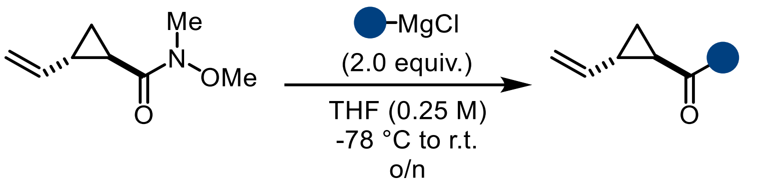

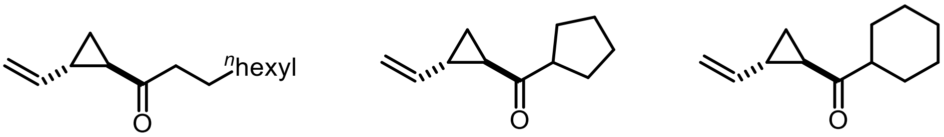

1-(*trans*-2-vinylcyclopropyl)nonan-1-one

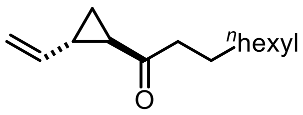

General procedure C was followed using *N*-methoxy-*N*-methyl-((*trans*)-2-vinylcyclopropane)-1-carboxamide (310.4 mg, 2.0 mmol, 8:92 *cis/trans* (^{1}H NMR)). Flash silica gel column chromatography (50:1 pentane:Et_2O) afforded the title product as a colorless oil (354.1 mg, 1.700 mmol, 85%, 8:92 *cis/trans* (^{1}H NMR)). **R$_f$** = 0.3 (50:1 pentane:Et_2O). **^{1}H NMR** (600 MHz, $CDCl_3$) δ 5.40 (ddd, *J* = 17.0, 10.3, 8.5 Hz, 1H), 5.14 (dd, *J* = 17.1, 1.4 Hz, 1H), 4.97 (dd, *J* = 10.3, 1.4 Hz, 1H), 2.54 (td, *J* = 7.3, 1.9 Hz, 2H), 2.03 – 1.92 (m, 2H), 1.63 – 1.56 (m, 2H), 1.43 (ddd, *J* = 8.9, 5.2, 3.9 Hz, 1H), 1.31 – 1.23 (m, 10H), 0.98 (ddd, *J* = 8.1, 6.3, 4.0 Hz, 1H), 0.87 (t, *J* = 7.0 Hz, 3H). **^{13}C NMR** (151 MHz, $CDCl_3$) δ 209.5, 138.7, 114.7, 44.1, 32.0, 29.6, 29.5, 29.4, 29.3, 28.4, 24.2, 22.8, 17.6, 14.2. **IR** (neat, cm^{-1}): 3004, 2925, 2856, 2324, 2159, 1697, 1638, 1458, 1385, 1305, 1201, 1129, 1083, 987, 902, 838, 722. **HRMS** (ESI): *m/z* [M+Na]$^+$ calculated for $C_{14}H_{24}ONa$: 231.1719, found: 231.1716.

Cyclohexyl((*trans*)-2-vinylcyclopropyl)methanone

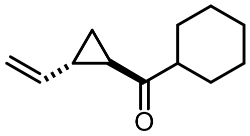

General procedure C was followed using *N*-methoxy-*N*-methyl-((*trans*)-2-vinylcyclopropane)-1-carboxamide (155.0 mg, 1.000 mmol, 1.0 equiv, 8:92 *cis/trans* (^{1}H NMR)). Flash silica gel column chromatography (100:1 pentane:Et_2O) afforded the title compound as a colorless oil (115.0 mg, 0.645 mmol, 65%, 3:97 *cis/trans* (^{1}H NMR)). **R$_f$** = 0.4 (50:1 pentane:Et_2O). **^{1}H NMR** (400 MHz, $CDCl_3$) δ 5.41 (ddd, *J* = 17.1, 10.2, 8.5 Hz, 1H), 5.14 (dd, *J* = 17.1, 1.5 Hz, 1H), 4.97 (dd, *J* = 10.3, 1.5 Hz, 1H), 2.54 – 2.44 (m, 1H), 2.01 (ddd, *J* = 8.0, 5.2, 3.8 Hz, 1H), 1.92 (ddt, *J* = 11.8, 8.1, 5.9 Hz, 3H), 1.82 – 1.75 (m, 2H), 1.72 – 1.64 (m, 1H), 1.41 (ddd, *J* = 8.9, 5.2, 3.9 Hz, 1H), 1.39 – 1.15 (m, 5H), 0.97 (ddd, *J* = 8.0, 6.3, 3.9 Hz, 1H). **^{13}C NMR** (101 MHz, $CDCl_3$) δ 212.0, 138.8, 114.7, 51.8, 28.5, 28.4, 26.1, 25.9, 25.8, 17.5. **IR** (neat, cm^{-1}): 3372, 3084, 3004, 2928, 2854, 2663, 2330, 2205, 2095, 1993, 1801, 1690, 1637, 1446, 1387, 1288, 1262, 1233, 1186, 1145, 1093, 1011, 900, 815. **HRMS** (EI): *m/z* [M]$^+$ calculated for $C_{12}H_{18}$: 178.1352, found 178.1358.

Cyclopentyl((*trans*)-2-vinylcyclopropyl)methanone

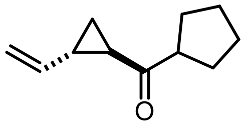

General procedure C was followed using *N*-methoxy-*N*-methyl-((*trans*)-2-vinylcyclopropane)-1-carboxamide (310 mg, 2.0 mmol, 1.0 equiv, 8:92 *cis*/*trans* (^{1}H NMR)). Flash silica gel column chromatography (50:1 pentane:Et_2O) afforded the title compound as a colorless oil (218.0 mg, 66%, 1.33 mmol, 6:94 *cis*/*trans* (^{1}H NMR)). **R_f** = 0.2 (50:1 pentane:Et_2O). **^{1}H NMR** (400 MHz, $CDCl_3$) δ 5.49 – 5.34 (m, 1H), 5.13 (dd, *J* = 17.1, 1.5 Hz, 1H), 4.97 (dd, *J* = 10.2, 1.5 Hz, 1H), 3.00 (p, *J* = 8.0 Hz, 1H), 2.01 – 1.92 (m, 2H), 1.89 – 1.74 (m, 3H), 1.61 (ddt, *J* = 14.6, 7.1, 1.9 Hz, 4H), 1.47 – 1.38 (m, 1H), 0.98 (ddd, *J* = 7.8, 6.3, 3.9 Hz, 1H). **^{13}C NMR** (101 MHz, $CDCl_3$) δ 211.3 (*trans*), 209.8 (*cis*), 138.7 (*trans*), 135.6 (*cis*), 115.7 (*trans*), 114.6 (*cis*), 53.0 (*cis*), 52.4, (*trans*), 28.9 (*trans*), 28.7 (*trans*), 28.6 (*trans*), 28.6 (*cis*), 28.5 (*trans*), 28.2 (*cis*), 27.9 (*cis*), 27.7 (*cis*), 26.2 (*trans*), 26.1 (*trans*), 26.0 (*cis*), 17.6 (*trans*), 14.8 (*cis*). **IR** (neat, cm^{-1}): 3373, 3183, 3084, 2952, 2869, 2656, 2332, 2089, 1990, 1808, 1754, 1690, 1637, 1523, 1445, 1384, 1300, 1203, 1111, 1055, 987, 902, 828, 749, 701, 660. **HRMS** (EI): *m/z* $[M]^+$ calculated for $C_{11}H_{16}O$: 164.1196, found 164.1195.

10.4.2. General procedure H: synthesis of *trans*-vinylcyclopropyl silyl enol ethers

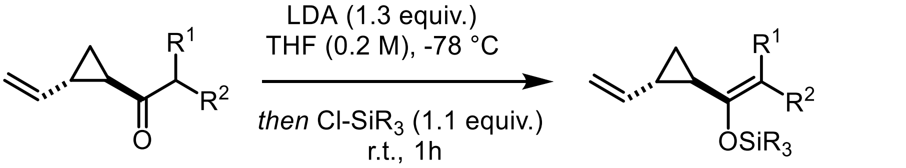

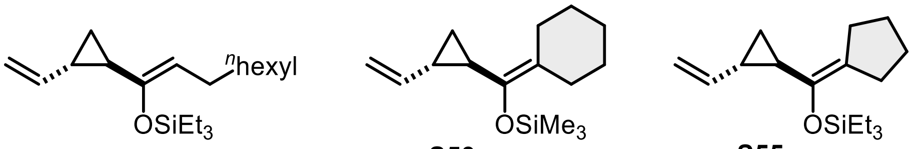

S43 **S53** **S55**

An oven dried Schlenk flask equipped with a magnetic stir bar was evacuated and backfilled with argon three times. A solution of the corresponding 1-(2-vinylcyclopropyl)ketone (0.5-1.0 mmol, 1.0 equiv.) in dry THF (0.2 M) was added and it was cooled to -78°C. Lithium diisopropylamide (1.3 equiv., 1.0 M in THF/hexane) was added dropwise to the reaction mixture and it was stirred for 1 h at -78 °C. Then, dry chlorotriethylsilane or chlorotrimethylsilane (1.2 equiv.) was added dropwise at -78°C. The mixture was allowed to warm up to room temperature and stirred for 1 h before it was quenched by addition of sat. aqueous $NaHCO_3$ (10 mL). The aqueous layer was extracted with Et_2O (2 x 10 mL) and the combined organic layers were washed with brine (5 mL), dried over Na_2SO_4, filtered, and concentrated under reduced pressure. The crude mixture was further purified by flash silica gel column chromatography.

10.4.3. Characterization data of *trans*-vinylcyclopropyl silyl enol ethers

Triethyl((1-((*trans*)-2-vinylcyclopropyl)non-1-en-1-yl)oxy)silane (43)

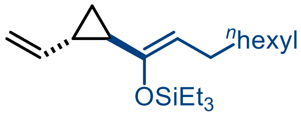

General procedure H was followed using 1-(*trans*-2-vinylcyclopropyl)nonan-1-one (208.3 mg, 1.0 mmol, 1.0 equiv.) and chlorotriethylsilane (0.2 mL, 1.2 equiv.). Flash silica gel column chromatography (100:1 pentane Et_3N)

afforded the title product **43** as a colorless oil (280.7 mg, 0.870 mmol, 87%, 56:44 *E*/*Z*). $\mathbf{R_f}$ = 0.9 (100:1 pentane:Et_3N). **^{1}H NMR** (600 MHz, CD_2Cl_2) δ 5.50 – 5.36 (m, 1H, *E* + 1H, *Z*), 5.08 (dd, *J* = 7.8, 1.7 Hz, 1H, *E*), 5.05 (dd, *J* = 7.7, 1.7 Hz, 1H, *Z*), 4.91 – 4.84 (m, 1H, *E* + 1H, *Z*), 4.63 (t, *J* = 7.6 Hz, 1H, *E*), 4.38 (t, *J* = 7.0 Hz, 1H, *Z*), 2.04 (q, *J* = 7.2 Hz, 2H, *E*), 2.00 – 1.95 (m, 2H, *Z*), 1.72 (tt, *J* = 8.8, 4.9 Hz, 1H, *Z*), 1.67 (ddd, *J* = 8.8, 5.7, 4.3 Hz, 1H, *E*), 1.50 (tt, *J* = 9.0, 5.0 Hz, 1H, *Z*), 1.39 – 1.23 (m, 12H, *E* + 12H, *Z*), 1.11 (ddd, *J* = 8.6, 5.6, 4.0 Hz, 1H, *E*), 1.02 – 0.93 (m, 9H, *E* + 9H, *Z*), 0.91 – 0.85 (m, 3H, *E* + 3H, *Z*), 0.73 – 0.63 (m, 6H, *E* + 6H, *Z*). **^{13}C NMR** (151 MHz, CD_2Cl_2) δ 150.0, 148.8, 140.9, 140.8, 112.1, 111.8, 106.0, 105.5, 31.9, 31.9, 30.8, 29.9, 29.4, 29.2, 29.1, 26.1, 25.3, 25.2, 23.9, 22.7, 22.7, 21.3, 20.9, 13.9, 13.1, 11.9, 6.6, 6.5, 5.5, 5.1. **IR** (neat, cm^{-1}): 2922 (s), 2657 (w), 2328 (m), 2105 (s), 1994 (m), 1657 (m), 1460 (m), 1384 (m), 1237 (s), 1094 (s), 1005 (s), 894 (m), 852 (m), 809 (w), 725 (s), 673 (m). **HRMS** (EI): *m/z* $[M]^+$ calculated for $C_{20}H_{38}OSi$: 322.2692, found 322.2686.

(Cyclohexylidene((*trans*)-2-vinylcyclopropyl)methoxy)trimethylsilane (S53)

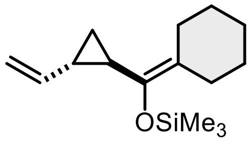

General procedure H was followed using cyclohexyl-2-vinylcyclopropylmethanone (106.9 mg, 0.600 mmol, 1.0 equiv.) and chlorotrimethylsilane (71.7 mg, 0.66 mmol, 1.1 equiv.). Flash silica gel column chromatography (100:3 pentane:Et_3N) afforded the title product **S53** as a colorless oil (123.2 mg, 0.492 mmol, 82%). $\mathbf{R_f}$ = 0.9 (100:3 pentane:Et_3N). **^{1}H NMR** (400 MHz, CD_2Cl_2) δ 5.43 (ddd, *J* = 16.9, 10.2, 8.5 Hz, 1H), 5.06 (ddd, *J* = 17.0, 1.7, 0.7 Hz, 1H), 4.88 (dd, *J* = 10.3, 1.8 Hz, 1H), 2.19 (t, *J* = 5.8 Hz, 2H), 2.11 (t, *J* = 6.1 Hz, 2H), 1.60 – 1.41 (m, 8H), 1.01 – 0.93 (m, 1H), 0.73 (ddd, *J* = 8.6, 5.6, 4.4 Hz, 1H), 0.16 (s, 9H). **^{13}C NMR** (101 MHz, CD_2Cl_2) δ 141.6, 140.4, 120.5, 112.2, 29.5, 28.4, 28.2, 27.8, 27.3, 23.7, 22.7, 14.1, 0.7. **IR** (neat, cm^{-1}): 3081, 2959, 2923, 2850, 2664, 2328, 2187, 2079, 1991, 1958, 1798, 1695, 1662, 1635, 1446, 1368, 1323, 1252, 1208, 1176, 1119, 1088, 1021, 977, 930, 840, 753, 694, 673. **HRMS** (EI): *m/z* $[M]^+$ calculated for $C_{15}H_{26}O_{Si}$: 250.1747, found 250.1753.

(Cyclopentylidene((*trans*)-2-vinylcyclopropyl)methoxy)triethylsilane (S55)

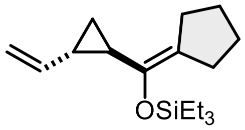

General procedure H was followed using cyclopentyl-((*trans*)-2-vinylcyclopropyl)methanone (176.5 mg, 1.07 mmol, 1.0 equiv.) and. chlorotriethylsilane (177.4 mg, 1.18 mmol, 1.1 equiv.). The mixture was stirred for 4 h at room temperature. Flash silica gel column chromatography (100:1 pentane Et_3N) afforded the title product **S55** as a colorless oil (274.0 mg, 0.983 mmol, 92%). $\mathbf{R_f}$ = 0.5 (100:1 pentane:Et_3N). **^{1}H NMR** (400 MHz, CD_2Cl_2) δ 5.45 (ddd, *J* = 17.0, 10.2, 8.6 Hz, 1H), 5.06 (dd, *J* = 17.1, 1.9 Hz, 1H), 4.86 (dd, *J* = 10.2, 1.8 Hz, 1H), 2.37 – 2.14 (m, 4H), 1.61 (ddtd, *J* = 13.0, 9.8, 6.5, 3.3 Hz, 5H), 1.56 – 1.49 (m, 1H), 0.98 (t, *J* = 7.9 Hz, 10H), 0.76 – 0.63 (m, 7H). **^{13}C NMR** (101 MHz, CD_2Cl_2) δ 141.6, 140.7, 121.8, 112.1, 30.3, 29.7, 27.6, 27.1, 24.0, 22.4, 13.1, 7.1, 6.1. **IR** (neat, cm^{-1}): 3082, 2952, 2877, 2734, 2653, 2330, 2191, 2107, 1990, 1937, 1795, 1675, 1635, 1457, 1414, 1376, 1311, 1239, 1168, 1092, 1037, 1004, 926, 892, 841, 804, 731, 674. **HRMS** (EI): *m/z* $[M]^+$ calculated for $C_{17}H_{30}OSi$: 278.2060, found 278.2061.

11. *Trans* to *cis* isomerization/Cope sequence

11.1. General procedure for *trans* to *cis* isomerization/Cope sequence

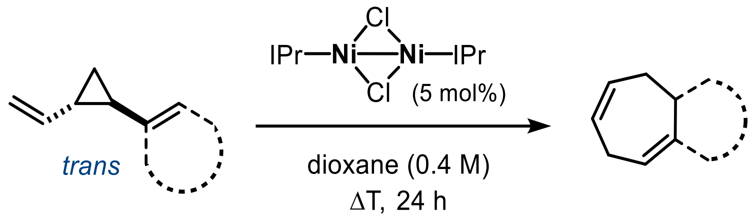

Inside an argon-filled glovebox, an oven dried 4 mL screw-cap vial equipped with a magnetic stir bar was charged with the corresponding divinylcyclopropane (1.0 equiv.), anhydrous 1,4-dioxane (0.4 M) and [Ni(μ-Cl)(IPr)]$_2$ **1** (5 mol%), in that order. The reaction vial was then sealed and allowed to stir inside the glovebox at the indicated temperature for the indicated time. Upon completion the reaction mixture was removed from the glovebox and quenched by the addition of wet pentane (*i.e.* technical grade pentane that had been distilled and stored on the bench). A spatula tip of ammonium pyrrolidine-1-dithiocarboxylic acid was added and the mixture stirred for additional 15 min to precipitate nickel.[13] The mixture was filtered through a plug of silica, rinsing with Et_2O and the filtrate was concentrated under reduced pressure.

Note: In most cases, the colour of the reaction mixture after completion is dark red. After addition of ammonium pyrrolidine-1-dithiocarboxylic acid and subsequent mixing, the colour changes rapidly to yellow. After further mixing (15 min) the solution becomes clear and precipitation occurs.

11.2. Characterization data of cyclized products

(+)-Dictyopterene C' (32)

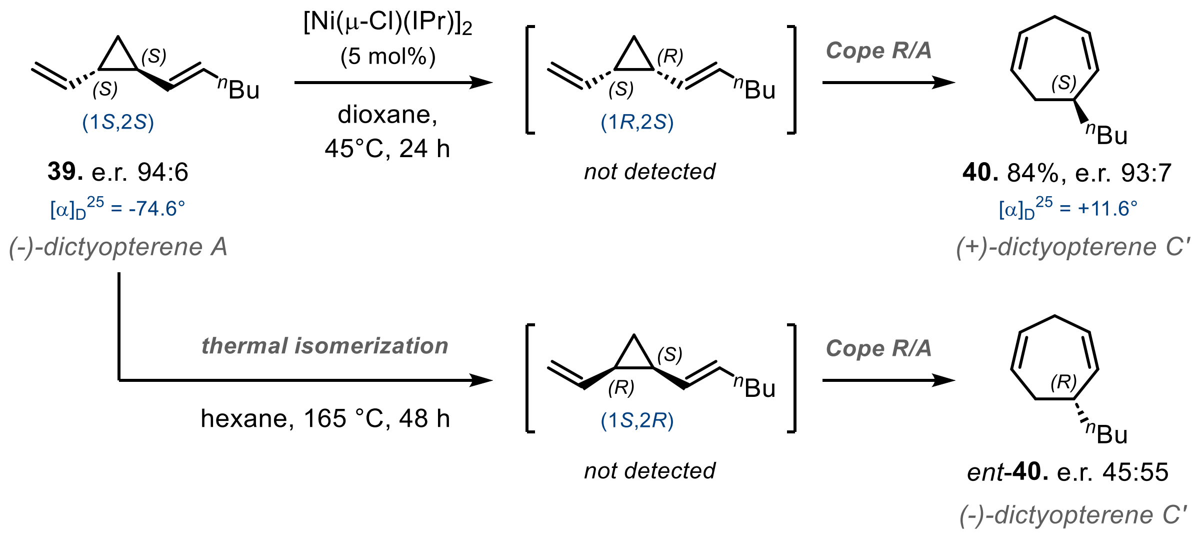

General procedure was followed using (-)-dictyopterene A **39** (16.7 mg, 0.110 mmol) and Nickel dimer (4.8 mg, 5 mol%). The reaction mixture was stirred at 45 °C for 24 h. Purification by filtration over a short silica plug washing with pentane afforded the title product **40** as a colourless liquid (14.0 mg, 84%, 86% ee). **[α]$_{25}$^{D}** = +11.6 (c = 0.65, $CHCl_3$). **^{1}H NMR** (400 MHz, $CDCl_3$) δ 5.71 (dt, *J* = 11.0, 5.2 Hz, 1H), 5.62

(dt, J = 8.1, 4.6 Hz, 3H), 3.04 – 2.89 (m, 1H), 2.70 (dt, J = 19.7, 5.7 Hz, 1H), 2.50 – 2.39 (m, 1H), 2.22 (ddt, J = 16.0, 6.3, 3.0 Hz, 1H), 2.18 – 2.01 (m, 1H), 1.41 – 1.26 (m, 6H), 0.92 – 0.88 (m, 3H). **^{13}C NMR** (101 MHz, $CDCl_3$) δ 137.0, 130.1, 128.3, 27.4, 37.3, 36.2, 33.0, 29.6, 28.5, 23.0, 14.3.

The thermal isomerization reaction was perfomed in accordance with literature procedure.[34] In an argon filled glovebox, a 8 ml pressure tube equipped with a magnetic stirring bar, was charged with (-)-dictyopterene A **39** (10 mg, 0.06 mmol) and dry hexane (2 mL, 0.03 M). The reaction mixture was stirred at 165 °C for 48 h. Evaporation of the solvent afforded the crude product (-)-Dictyopterene C′ ***ent*-40** as a colorless liquid in 10% ee. *Note*: Under thermal conditions the terminal vinyl moiety is favored to undergo rotation.[35,36]

6-benzylcyclohepta-1,4-diene (42)

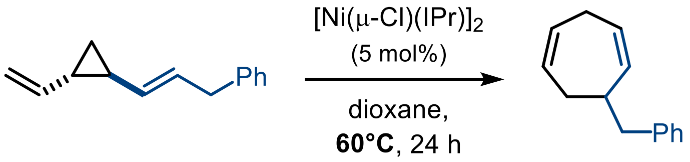

General procedure was followed using ((*E*)-3-(*trans*-2-vinylcyclopropyl)allyl)benzene **41** (18.4 mg, 0.100 mmol) and Nickel dimer (4.8 mg, 5 mol%). Pyridine (0.8 mg, 10 mol%) was added as additive and the reaction mixture was stirred at 60 °C for 24 h. Purification by filtration over a short silica plug washing with Et_2O afforded the title product **42** as a colourless oil (18.0 mg, 0.098 mmol, 98%). **R_f** = 0.4 (hexane). **^{1}H NMR** (600 MHz, $CDCl_3$) δ 7.33 – 7.27 (m, 2H), 7.24 – 7.17 (m, 3H), 5.73 – 5.65 (m, 2H), 5.65 – 5.61 (m, 2H), 2.95 (dd, J = 19.4, 2.6 Hz, 1H), 2.81 – 2.70 (m, 3H), 2.63 (dd, J = 13.1, 7.8 Hz, 1H), 2.30 – 2.23 (m, 1H), 2.15 – 2.07 (m, 1H). **^{13}C NMR** (151 MHz, $CDCl_3$) δ 140.9, 135.7, 129.7, 129.2, 128.9, 128.3, 127.6, 126.0, 42.4, 39.2, 32.2, 28.6. **HRMS** (EI): *m/z* $[M]^+$ calculated for $C_{14}H_{16}$: 184.1247, found 184.1238. The data are in agreement with those previously reported in the literature.[6]

Triethyl((7-heptylcyclohepta-1,4-dien-1-yl)oxy)silane (44)

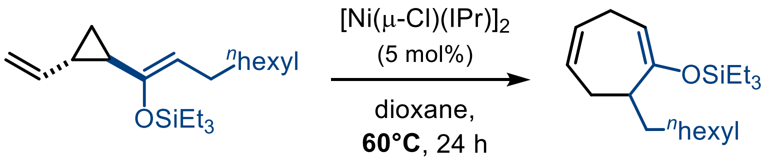

General procedure was followed using triethyl((1-(*trans*-2-vinylcyclopropyl)non-1-en-1-yl)oxy)silane **43** (64.5 mg, 0.200 mmol, 1.0 equiv.) and Nickel dimer (9.7 mg, 5 mol%). The reaction mixture was stirred at 60 °C for 24 h. Purification by filtration over a short plug of activated basic aluminium oxide washing with Et_2O afforded the title product **44** as colorless oil (0.184 mmol, 92% (determined by quantitative ^{1}H NMR)). **^{1}H NMR** (600 MHz, CD_2Cl_2) δ 5.76 (dtd, J = 10.4, 4.5, 1.9 Hz, 1H), 5.67 (dtd, J = 10.8, 6.0, 2.0 Hz, 1H), 4.81 (t, J = 5.7 Hz, 1H), 2.81 – 2.72 (m, 1H), 2.57 (dt, J = 19.2, 6.3 Hz, 1H), 2.34 – 2.25 (m, 2H), 2.17 – 2.09 (m, 1H), 1.69 – 1.61 (m, 1H), 1.37 – 1.28 (m, 11H), 0.97 (t, J = 7.9 Hz, 9H), 0.89 (t, J = 7.0 Hz, 3H), 0.66 (q, J = 8.0 Hz, 6H). *Note*: The product was used in the next step without further purification and analysis.

2-Heptylcyclohept-4-en-1-one (45)

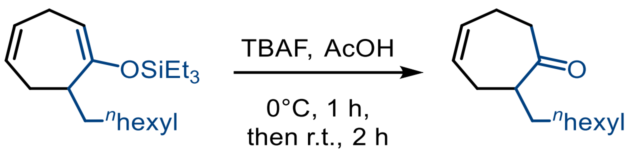

A 10 mL round bottom flask was charged with crude triethyl((7-heptylcyclohepta-1,4-dien-1-yl)oxy)silane **44** (0.200 mmol, 1.0 equiv.). It was dissolved in THF (0.5 mL, 0.4 M) and cooled to 0 °C. To this mixture, AcOH (57.2 µL, 1.0 mmol, 5.0 equiv.) and TBAF (1.0 mL, 1.0 mmol, 5.0 equiv., 1M in THF) were added and stirred at 0 °C for 1h and then at r.t. for 2h. The solvent was removed under reduced pressure and the crude was purified by flash column chromatography to afford the title compound **45** (40.9 mg, 0.196 mmol, 98%) as a colorless oil. $\mathbf{R_f}$ = 0.15 (50:1 pentane:Et_2O). **^{1}H NMR** (400 MHz, $CDCl_3$) δ 5.74 (qt, *J* = 10.7, 5.8 Hz, 2H), 2.79 (ddt, *J* = 10.7, 6.7, 3.2 Hz, 1H), 2.71 (ddd, *J* = 15.5, 11.3, 4.2 Hz, 1H), 2.54 – 2.42 (m, 2H), 2.32 (dt, *J* = 16.5, 4.1 Hz, 1H), 2.26 – 2.18 (m, 1H), 2.17 – 2.07 (m, 1H), 1.71 (p, *J* = 7.2 Hz, 1H), 1.38 – 1.16 (m, 11H), 0.87 (t, *J* = 6.7 Hz, 3H). **^{13}C NMR** (101 MHz, $CDCl_3$) δ 215.0, 129.6, 129.0, 51.3, 42.2, 32.0, 31.2, 30.9, 29.8, 29.3, 27.4, 24.4, 22.8, 14.2. **IR** (neat, cm^{-1}): 2924, 2664, 2103, 1705, 1457, 1376, 1197, 1100, 912, 722. **HRMS** (EI): *m/z* $[M]^+$ calculated for $C_{14}H_{24}O$: 208.1827, found 208.1822.

2,3,4,4a,5,8-hexahydro-1*H*-benzo[7]annulene (46)

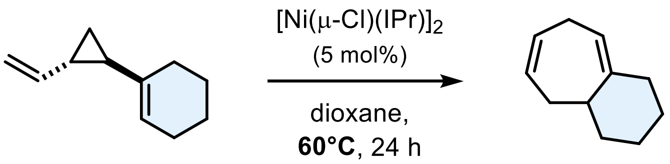

General procedure was followed using 1-(*trans*-2-vinylcyclopropyl)cyclohex-1-ene **S46** (18.5 mg, 0.100 mmol, 1.0 equiv.) and Nickel dimer (4.8 mg, 5 mol%). The reaction mixture was stirred at 60 °C for 24 h. Purification by filtration over a short silica plug washing with Et_2O afforded the title product **46** as a colourless liquid (0.097 mmol, 97% (determined by quantitative ^{1}H NMR)). **^{1}H NMR** (600 MHz, $CDCl_3$) δ 5.96 (dt, *J* = 9.8, 6.1 Hz, 1H), 5.89 – 5.81 (m, 1H), 5.38 – 5.32 (m, 1H), 2.87 – 2.78 (m, 1H), 2.58 (dtt, *J* = 17.0, 6.6, 1.8 Hz, 1H), 2.28 – 2.22 (m, 2H), 2.11 – 2.01 (m, 2H), 1.96 – 1.87 (m, 1H), 1.77 – 1.68 (m, 2H), 1.67 – 1.60 (m, 1H), 1.43 – 1.33 (m, 1H), 1.33 – 1.20 (m, 2H). **^{13}C NMR** (151 MHz, $CDCl_3$) δ 144.5, 132.8, 130.3, 118.5, 39.7, 39.2, 35.5, 32.7, 28.5, 26.7, 25.8. **HRMS** (EI): *m/z* $[M]^+$ calculated for $C_{11}H_{16}$: 148.1252, found 148.1248. *Note: Residual dioxane could not be completely removed due to the volatility of the product.*

1,2,3,4,5,5a,6,9-octahydroheptalene (47)

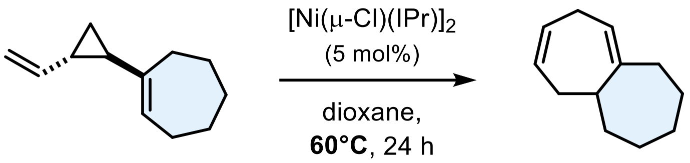

General procedure was followed using 1-((*trans*)-2-vinylcyclopropyl)cyclohept-1-ene **S47** (16.2 mg, 0.100 mmol) and Nickel dimer (4.8 mg, 5 mol%). The reaction mixture was stirred at 60 °C for 24 h. Purification by filtration over a short silica plug washing with Et_2O afforded the title product **47** as a colourless oil (15 mg, 92%). **R_f** = 0.5 (hexane). **^{1}H NMR** (600 MHz, $CDCl_3$) δ 5.64 (dd, *J* = 8.3, 4.3 Hz, 1H), 5.60 – 5.49 (m, 2H), 3.14 – 3.05 (m, 1H), 3.06 – 2.97 (m, 1H), 2.37 (dt, *J* = 18.1, 7.7 Hz, 1H), 2.18 – 2.13 (m, 2H), 2.01 (ddt, *J* = 8.0, 5.5, 2.6 Hz, 2H), 1.89 – 1.77 (m, 2H), 1.76 – 1.63 (m, 2H), 1.42 – 1.32 (m, 1H), 1.33 – 1.17 (m, 3H). **^{13}C NMR** (151 MHz, $CDCl_3$) δ 149.5, 129.8, 126.7, 124.6, 40.7, 35.4, 35.0, 34.8, 31.8, 31.0, 28.3, 26.8. **IR** (neat, cm^{-1}): 3387, 3027, 2922, 2854, 2692, 2326, 2159, 2100, 1990, 1723, 1691, 1448, 1392, 1230, 1169, 983, 847, 741, 660. *Note: Caution, compound is volatile.*

1,3,4,6,9,9a-Hexahydrocyclohepta[*c*]pyran (48)

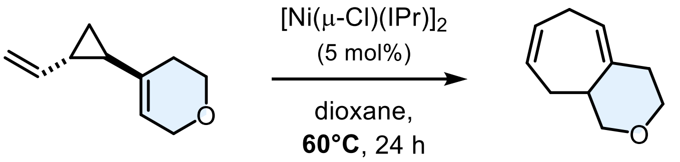

General procedure was followed using 4-(*trans*-2-vinylcyclopropyl)-3,6-dihydro-2*H*-pyran **S48** (15.0 mg, 0.100 mmol, 1.0 equiv.) and Nickel dimer (4.8 mg, 5 mol%). The reaction mixture was stirred at 60 °C for 24 h. Flash column chromatography (50:1 pentane:Et_2O) afforded the title product **48** as a colorless oil (14.2 mg, 0.095 mmol, 95%). **R_f** = 0.20 (50:1 pentane:Et_2O). **^{1}H NMR** (600 MHz, CD_2Cl_2) δ 5.96 – 5.89 (m, 1H), 5.87 – 5.80 (m, 1H), 5.41 (dd, *J* = 8.1, 3.1 Hz, 1H), 3.89 (dd, *J* = 10.7, 5.2 Hz, 1H), 3.74 (dd, *J* = 10.7, 4.9 Hz, 1H), 3.33 (ddd, *J* = 12.8, 10.7, 2.6 Hz, 1H), 3.11 (t, *J* = 10.7 Hz, 1H), 3.00 – 2.93 (m, 1H), 2.53 (dt, *J* = 17.4, 7.3 Hz, 1H), 2.37 – 2.20 (m, 3H), 2.01 – 1.98 (m, 1H), 1.97 – 1.93 (m, 1H). **^{13}C NMR** (151 MHz, CD_2Cl_2) δ 140.2, 132.9, 130.1, 120.9, 74.0, 70.0, 39.9, 38.7, 27.9, 26.2. **IR** (neat, cm^{-1}): 2931, 2200, 1652, 1383, 1216, 1104, 1028, 972, 758, 664. **HRMS** (EI): *m/z* $[M]^+$ calculated for $C_{10}H_{14}O$: 150.1045, found 150.1040.

***tert*-butyl 1,3,4,6,9,9a-hexahydro-2*H*-cyclohepta[c]pyridine-2-carboxylate (49)**

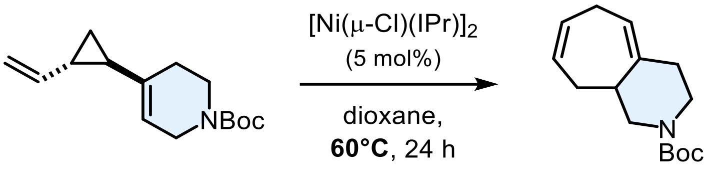

General procedure was followed using tert-butyl 4-((*trans*)-2-vinylcyclopropyl)-3,6-dihydropyridine-1(2*H*)-carboxylate **S49** (24.9 mg, 0.100 mmol) and Nickel dimer (4.8 mg, 5 mol%). The reaction mixture was stirred at 60 °C for 24 h. Flash column chromatography (100:2:1 pentane:Et_2O:acetone) afforded the title product **49** as a colorless oil (0.073 mmol, 73% (determined by quantitative ^{1}H NMR). **R_f** = 0.2

(100:2:1 pentane:Et_2O:acetone). **^{1}H NMR** (600 MHz, $CDCl_3$) δ 5.87 (dt, *J* = 11.1, 5.8 Hz, 1H), 5.79 (dt, *J* = 9.0, 6.0 Hz, 1H), 5.50 (t, *J* = 5.8 Hz, 1H), 4.01 – 3.82 (m, 2H), 2.86 – 2.78 (m, 2H), 2.72 – 2.63 (m, 2H), 2.41 – 2.36 (m, 2H), 2.28 – 2.21 (m, 1H), 2.18 – 2.12 (m, 2H), 2.12 – 2.06 (m, 1H), 1.46 (s, 9H). **^{13}C NMR** (151 MHz, $CDCl_3$) δ 155.1, 140.3, 129.4, 122.0, 121.0, 79.6, 45.8, 38.5, 37.1, 36.2, 29.7, 28.6, 26.1. **IR** (neat, cm^{-1}): 3321, 2974, 2930, 2160, 2030, 1665, 1421, 1366, 1243, 1160, 1120, 1024, 867, 768, 725. **HRMS** (EI): *m z*: calculated for [M+H -*t*Bu]$^+$ $C_{11}H_{15}O_2N$: 193.1097, found 193.1098.

1,3,4,6,9,9a-hexahydrospiro[benzo[7]annulene-2,2'-[1,3]dioxolane] (50)

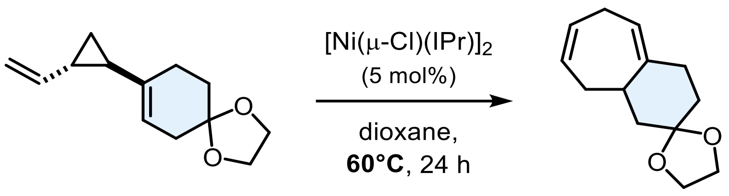

General procedure was followed using 8-((trans)-2-vinylcyclopropyl)-1,4-dioxaspiro[4.5]dec-7-ene **S50** (20.6 mg, 0.100 mmol) and Nickel dimer (4.8 mg, 5 mol%). The reaction mixture was stirred at 60 °C for 24 h. Flash column chromatography (10:1 pentane:Et_2O) afforded the title product **50** as a colorless oil (18.0 mg, 0.087 mmol, 87%). **R_f** = 0.19 (10:1 pentane:Et_2O). **^{1}H NMR** (600 MHz, $CDCl_3$) δ 5.96 (dt, *J* = 9.8, 6.0 Hz, 1H), 5.83 (dt, *J* = 9.8, 6.9 Hz, 1H), 5.43 (t, *J* = 5.7 Hz, 1H), 4.00 – 3.91 (m, 4H), 2.84 – 2.75 (m, 1H), 2.69 – 2.60 (m, 1H), 2.35 (d, *J* = 11.7 Hz, 1H), 2.30 (ddd, *J* = 13.8, 7.2, 3.9 Hz, 1H), 2.22 (ddd, *J* = 13.7, 9.3, 6.6 Hz, 2H), 2.07 (ddd, *J* = 13.6, 4.8, 2.9 Hz, 1H), 1.80 – 1.73 (m, 1H), 1.67 (ddd, *J* = 12.8, 4.2, 2.9 Hz, 1H), 1.58 (t, *J* = 12.8 Hz, 1H), 1.53 (td, *J* = 13.4, 4.9 Hz, 1H). **^{13}C NMR** (151 MHz, $CDCl_3$) δ 141.8, 133.0, 129.8, 119.8, 109.2, 64.5, 64.4, 42.9, 36.4, 35.9, 35.5, 31.9, 25.8. **IR** (neat, cm^{-1}): 3353, 3025, 2936, 2880, 2327, 2089, 1992, 1721, 1441, 1353, 1279, 1251, 1120, 1061, 945, 923, 830, 761, 731, 687. **HRMS** (EI): *m/z*: calculated for [M]$^+$ $C_{13}H_{18}O_2$: 206.1301, found: 206.1297.

Spiro[5.6]dodeca-7,10-diene (52)

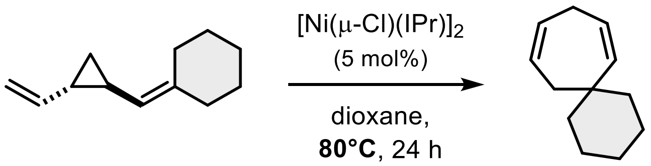

General procedure was followed using 2-vinylcyclopropylmethylenecyclohexane **51** (37.7 mg, 0.232 mmol) and Nickel dimer (11.4 mg, 5 mol%). The reaction mixture was stirred at 80 °C for 24 h. Flash column chromatography (pentane) afforded the title product **52** as a colorless liquid (25.5 mg, 0.157 mmol, 68%). **R_f** = 0.8 (pentane). **^{1}H NMR** (400 MHz, $CDCl_3$) δ 5.77 – 5.67 (m, 2H), 5.53 (d, *J* = 12.0 Hz, 1H), 5.49 – 5.42 (m, 1H), 2.82 (ddt, *J* = 4.4, 2.7, 1.3 Hz, 2H), 2.23 (dd, *J* = 4.7, 1.4 Hz, 2H), 1.52 – 1.40 (m, 8H), 1.38 – 1.30 (m, 2H). **^{13}C NMR** (101 MHz, $CDCl_3$) δ 140.3, 129.8, 128.6, 124.7, 38.2, 37.9, 37.5, 29.4, 26.5, 22.1. **IR** (neat, cm^{-1}): 3011, 2922, 2850, 2665, 2327, 2081, 1992, 1654, 1448, 1314, 1262, 1162, 1132, 1098, 1039, 1005, 973, 930, 904, 823, 800, 734, 698, 660. **HRMS** (EI): *m/z* [M]$^+$ calculated for $C_{12}H_{18}$: 162.1403, found 162.1404.

Spiro[5.6]dodec-10-en-7-one (54)

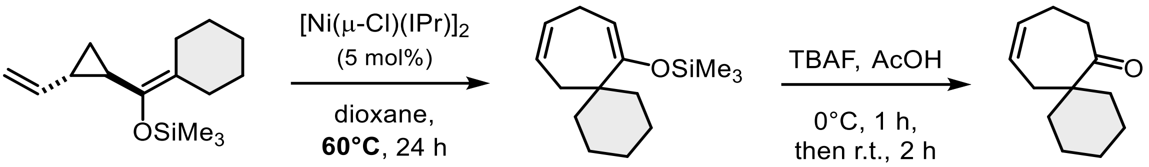

S53 **53** **54**

General procedure was followed using cyclohexylidene-2-vinylcyclopropylmethoxytrimethylsilane **S53** (45.1 mg, 0.18 mmol) and Nickel dimer (8.7 mg, 5 mol%). The reaction mixture was stirred at 60 °C for 24 h. The mixture was filtered through a plug of activated basic aluminium oxide and the obtained crude **53** analysed by quantitative ^{1}H NMR (0.137 mmol, 76% yield). The crude was dissolved in THF (0.5 mL) and cooled to 0°C. To this mixture, AcOH (57.2 µL, 1 mmol, 5.0 equiv.) and TBAF (1M in THF, 1 mL, 1.0 mmol, 5.0 equiv.) were added and stirred at 0 °C for 1h and then at r.t. for 2h. The solvent was evaporated and the crude purified by flash column chromatography (50:1 pentane:Et_2O) to afford the title product **54** as a colorless liquid (22.6 mg, 0.127 mmol, 71% *over 2 steps*). $\mathbf{R_f}$ = 0.4 (20:1 pentane:Et_2O). **^{1}H NMR** (600 MHz, $CDCl_3$) δ 5.77 – 5.69 (m, 2H), 2.67 (t, *J* = 6.7 Hz, 2H), 2.35 – 2.22 (m, 4H), 1.80 (ddd, *J* = 12.9, 9.0, 3.8 Hz, 2H), 1.52 – 1.33 (m, 8H). **^{13}C NMR** (151 MHz, $CDCl_3$) δ 217.1, 130.3, 127.3, 54.2, 37.9, 32.9, 32.7, 26.8, 25.9, 22.0. **IR** (neat, cm^{-1}): 3020, 2925, 2855, 2666, 2331, 2099, 2004, 1695, 1447, 1349, 1311, 1199, 1175, 1141, 1086, 1002, 909, 835, 774, 730, 668. **HRMS** (EI): *m/z* $[M]^+$ calculated for $C_{12}H_{18}O$: 178.1352, found 178.1353.

Spiro[4.6]undec-9-en-6-one (56)

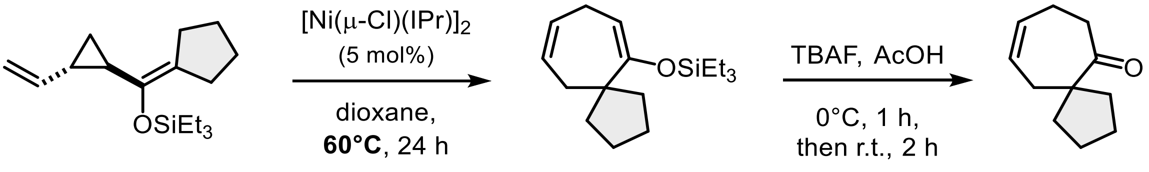

S55 **55.** 77% **56.** 79%

General procedure was followed using cyclopentylidene-2-vinylcyclopropylmethoxytriethylsilane **S55** (55.7 mg, 0.200 mmol) and Nickel dimer (9.7 mg, 5 mol%). The reaction mixture was stirred at 60 °C for 24 h. The mixture was filtered through a plug of activated basic aluminium oxide, concentrated and the obtained crude **55** analysed by quantitative ^{1}H NMR (0.154 mmol, 77%). The crude was dissolved in THF (0.5 mL) and cooled to 0°C. To this, AcOH (57.2 µL, 1 mmol, 5.0 equiv.) and TBAF (1.0 mL, 1.0 mmol, 5.0 equiv. 1M in THF) were added and stirred at 0 °C for 1h and then at r.t. for 2h. The solvent was evaporated and the crude purified by flash column chromatography (50:1 pentane:Et_2O) to afford the title product **56** as a colorless liquid (26.0 mg, 0.158 mmol, 79% *over 2 steps*). $\mathbf{R_f}$ = 0.4 (20:1 pentane:Et_2O) **^{1}H NMR** (600 MHz, $CDCl_3$) δ 5.72 (dtt, *J* = 12.6, 6.1, 1.8 Hz, 1H), 5.64 (ddd, *J* = 11.2, 4.8, 3.6 Hz, 1H), 2.77 (t, *J* = 6.7 Hz, 2H), 2.36 (dt, *J* = 6.6, 2.0 Hz, 2H), 2.33 (dd, *J* = 6.3, 1.5 Hz, 2H), 1.97 (ddd, *J* = 13.1, 9.5, 4.0 Hz, 2H), 1.72 – 1.59 (m, 4H), 1.49 – 1.43 (m, 2H). **^{13}C NMR** (151 MHz, $CDCl_3$) δ 217.0, 129.9, 127.7, 61.5, 39.5, 36.7, 35.5, 27.1, 25.6. **IR** (neat, cm^{-1}): 3019, 2948, 2867, 2332, 2112, 1999, 1696, 1442, 1348, 1309, 1192, 1138, 1080, 994, 937, 901, 777, 731. **HRMS** (EI): *m/z* $[M]^+$ calculated for $C_{12}H_{16}O$: 164.1196, found 164.1199.

12. Computational Details

All calculations were performed with the Gaussian 16 program package (revision A.03).[37] Gas phase structural optimizations and frequency calculations were performed with MN15[38] along with 6-31G(d) basis set on C, H, N, and Cl atoms, and the effective core potential (ECP) SDD on Ni. Single point energy calculations were performed with M06L[39] and def2-TZVP basis set on all atoms. Solvation energies were described using CPCM model for 1,4-dioxane (unless otherwise stated). Frequency calculations were performed to confirm the structures minima (no imaginary frequencies) or transition states (exactly one imaginary frequency). Intrinsic reaction coordinate (IRC) analysis was used to confirm that the obtained transition states connect the corresponding reactants and products. Conformational searches were conducted manually on all of the species.

Orbital analyses were performed using ORCA 5.0.3.[40] at the B3LYP/def2-TZVP level of theory. A broken symmetry solution of Int2 was obtained using the brokensym feature in ORCA (keyword "%scf brokensym 2,1 end"). All shown orbitals are localized molecular orbitals obtained using the Pipek-Mezey localization scheme. Visualization of geometries and orbitals was done using the Chimera software.[41]

12.1. Method survey

12.1.1.Geometry optimization

The crystal structures of Ni$^{(I)}$ complexes **1** and **3** as well as related Ni$^{(II)}$ complexes (Figure S9) were used as starting points for geometry optimizations using MN15, MN15L, B3LYP-D3 and TPSS-D3. The obtained DFT geometries were then compared with those of the x-ray structures (Table S14). Notably, Ni$^{(II)}$ dimer [Ni(μ-Cl)(Cl)(IPr)]$_2$ has been reported to predominantly adopt a square-planar geometry (CCDC 769018), although tetrahedral distortion can occur (CCDC 899079).[42]

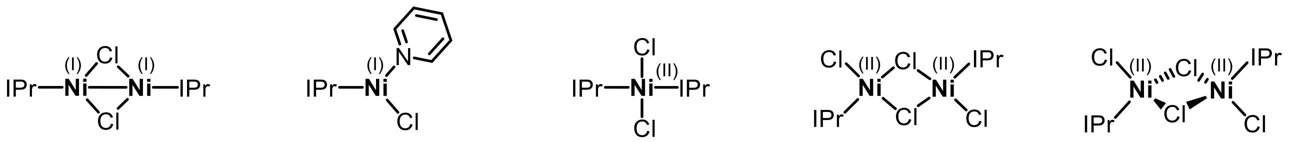

1 [CCDC 265407] | **3** [CCDC 1472757] | *square-planar* [CCDC 205515] | *square-planar* [CCDC 769018] | *tetrahedral* [CCDC 899079]

Figure S9. Ni(I) and Ni(II) complexes with known X-ray structures used for benchmarking DFT functionals for geometry optimization.

While B3LYP-D3 and TPSS-D3 performed well in describing bond lengths, angles and dihedral angles were not well reproduced. In particular, the Ni$^{(II)}$ dimer adopted a bent geometry about the Ni centers. In contrast, both MN15 and MN15L describe the considered Ni complexes reasonably well both in terms of bond lengths and (dihedral) angles, with MN15 giving slightly smaller deviations for Ni$^{(II)}$ complexes. Additionally, when optimizing the unfavored tetrahedral geometry of the Ni$^{(II)}$ dimer with MN15 a change in geometry about the Ni center to the more favored square-planar geometry occurred, while all efforts in optimizing the tetrahedral geometry with MN15L did not converge to a stable geometry. Therefore, MN15 was chosen for all further geometry optimizations.

Table S14. Comparison of DFT-optimized geometries with those of the respective crystal structures.

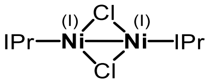

**1** [CCDC 265407]	**Ni-Ni**		**Ni-Cl**		**Ni-NHC**	
**xrd**	**2.51939**		**2.225453**		**1.87795**	
MN15	2.29194	-0.227	2.240805	0.015	1.91520	0.037
MN15L	2.34365	-0.176	2.230655	0.005	1.85876	-0.019
B3LYP-D3	2.30053	-0.219	2.228283	0.003	1.87732	-0.001
TPSS-D3	2.33990	-0.179	2.21777	-0.008	1.84920	-0.029

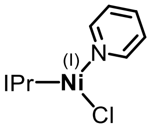

**3** [CCDC 1472757]	**Ni-py**		**Ni-Cl**		**Ni-NHC**		**py-Ni-NHC angle**		**Cl-Ni-NHC angle**	
**xrd**	**1.99443**		**2.19187**		**1.89523**		**110.8922**		**143.9932**	
MN15	2.06711	0.073	2.21882	0.027	1.96396	0.069	108.7717	-2.1	146.5092	2.5
MN15L	2.02092	0.026	2.21114	0.019	1.89822	0.003	113.0695	2.2	144.8195	0.8
B3LYP-D3	2.04391	0.049	2.21158	0.020	1.91283	0.018	111.0882	0.2	145.4882	1.5
TPSS-D3	1.98073	-0.014	2.21921	0.027	1.88731	-0.008	111.7770	0.9	145.5354	1.5

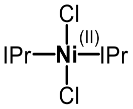

square-planar

[CCDC 205515]			**Ni-Cl**		**Ni-NHC**		**NHC dihedral**	
**xrd**			**2.15876**		**1.93313**		**40.9193**	
MN15			2.19949	0.041	1.94980	0.017	43.1321	2.2
MN15L			2.20899	0.050	1.93157	-0.002	49.4954	8.6
B3LYP-D3			2.219425	0.061	1.94695	0.014	26.5459	-14.4
TPSS-D3			2.20901	0.050	1.93027	-0.003	30.6305	-10.3

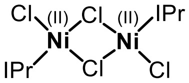

square-planar

[CCDC 769018]	**Ni-Ni**		**Ni-Cl**		**Ni-NHC**		**NHC dihedral**		**Cl-Ni-NHC dihedral**	
**xrd**	**3.25391**		**2.15151**		**1.88050**		**180.000**		**62.5874**	
MN15	3.28028	0.026	2.16454	0.013	1.84963	-0.031	180.000	0.0	71.3206	8.7
MN15L	3.31402	0.060	2.17281	0.021	1.83798	-0.043	179.989	0.0	68.9144	6.3
B3LYP-D3	3.21313	-0.041	2.18604	0.035	1.87589	-0.005	140.685	-39.3	72.0115	9.4
TPSS-D3	3.24565	-0.008	2.17550	0.024	1.84884	-0.033	148.165	-31.8	72.8010	10.2

12.1.2.Energies

Based on geometry optimizations at MN15/6-31G(d)(SDD for Ni) different methods were compared for the calculation of energies. Due to the presence of both open- and closed-shell species and the associated difficulties in describing both species adequatly a wide variety of methods was tested. Particular attention was paid to the amount of HF-exchange, since this has been shown to greatly affect the description of open-shell species.[43,44] Figure S10 depicts an overview of the tested methods.

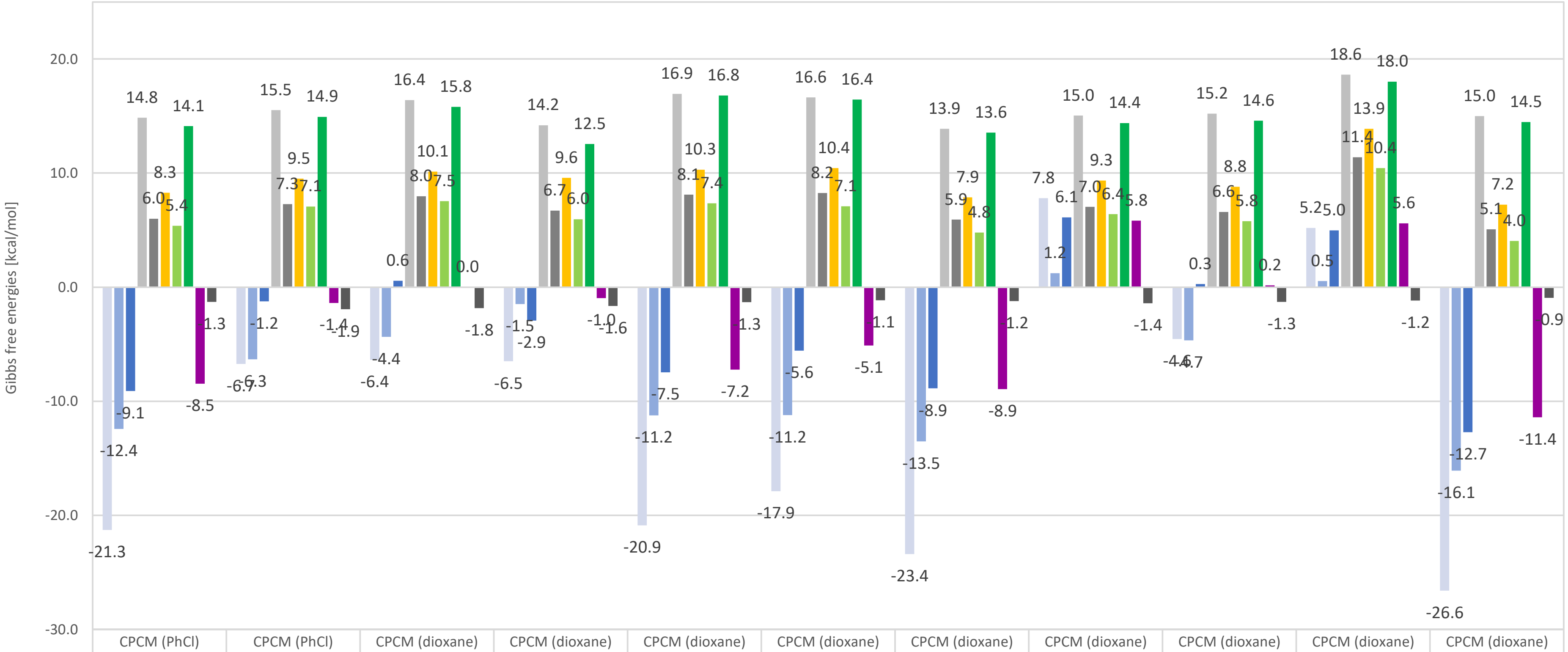

	CPCM (PhCl) MN15/def2TZVP	CPCM (PhCl) M06L/def2TZVP	CPCM (dioxane) M06L/def2TZVP	CPCM (dioxane) MN15L/def2TZVP	CPCM (dioxane) M06/def2TZVP	CPCM (dioxane) B3LYP-D3/def2TZVP	CPCM (dioxane) PBE0-D3/def2TZVP	CPCM (dioxane) TPSS-D3/def2TZVP	CPCM (dioxane) TPSSh-D3/def2TZVP	CPCM (dioxane) B97-D3/def2TZVP	CPCM (dioxane) wB97XD/def2TZVP
triplet Ni(I) dimer	-21.3	-6.7	-6.4	-6.5	-20.9	-17.9	-23.4	7.8	-4.6	5.2	-26.6
Ni(I) pyridine (3)	-12.4	-6.3	-4.4	-1.5	-11.2	-11.2	-13.5	1.2	-4.7	0.5	-16.1
Int1	-9.1	-1.2	0.6	-2.9	-7.5	-5.6	-8.9	6.1	0.3	5.0	-12.7
TS1	14.8	15.5	16.4	14.2	16.9	16.6	13.9	15.0	15.2	18.6	15.0
Int2	6.0	7.3	8.0	6.7	8.1	8.2	5.9	7.0	6.6	11.4	5.1
TS2	8.3	9.5	10.1	9.6	10.3	10.4	7.9	9.3	8.8	13.9	7.2
Int3	5.4	7.1	7.5	6.0	7.4	7.1	4.8	6.4	5.8	10.4	4.0
TS3	14.1	14.9	15.8	12.5	16.8	16.4	13.6	14.4	14.6	18.0	14.5
Int4	-8.5	-1.4	0.0	-1.0	-7.2	-5.1	-8.9	5.8	0.2	5.6	-11.4
dG_rxn	-1.3	-1.9	-1.8	-1.6	-1.3	-1.1	-1.2	-1.4	-1.3	-1.2	-0.9

Figure S10. Overview of tested methods for energy calculation (based on geometries obtained at MN15/6-31G(d)(SDD for Ni)). All energies are Gibbs free energies (in kcal/mol) relative to Ni[(I)] dimer **1**.

Based on the EPR-silent nature of the $Ni^{(I)}$ dimer **1**, its ground-state is expected to be a closed shell singlet. However, only the non-hybrid funtionals (TPSS-D3, B97-D3) correctly predict the singlet to be favoured over the triplet (i.e. positive singlet-triplet gap). Despite their lack of HF exchange local Minnesota functionals (MN15L, M06L) predict a slightly negative singlet-triplet gap, whereas all hybrid functionals even more greatly favor the triplet state.[43-45]

Experimentally, the formation of pyridine-coordinated $Ni^{(I)}$ monomer **3** occurs by subjecting $Ni^{(I)}$ dimer **1** to an excess of pyridine,[12] hence the process should either be exergonic or slightly endergonic (excess pyridine can drive the equilibrium). In this context, the local Minnesota functionals offer a good compromise with slightly exergonic or within error margins thermoneutral processes, whereas other non-hybrid funtionals suggest unreasonably endergonic processes.

Barriers for ring-opening (i.e. TS1, light grey bars in Figure S10) as well as their endergonicities (i.e. Int2, grey bars in Figure S10) are relatively consistent within the surveyed methods, as are the thermodynamic driving forces for the isomerization (i.e. dG_rxn, black bars in Figure S10). All methods predict barriers that can be overcome at ambient temperature (i.e. 13.9 – 18.6 kcal/mol) and thermodynamic driving forces in line with the observed *trans*/*cis* ratio (i.e. slight preference for *trans*; -0.9 – -1.9 kcal/mol). Consistently the ring-opened intermediates (Int2 and Int3) are always less stabilized than the ring-closed intermediates (Int1 and Int4). Moreover, the ring-opened *trans*-intermediate Int3 is always slightly more stable than its *cis*-analog Int2 and the TS for ring-closing (TS3) is always slightly lower than that of ring-opening (TS1). The barrier for isomerization (TS2) is very facile (lower than 3 kcal/mol) for all of the tested methods.

While the absolute values of the energies for all species with unpaired electrons on Ni (i.e. triplet $Ni^{(I)}$ dimer ^{3}**1**, $Ni^{(I)}$ pyridine complex **3**, Int1, Int4) are very method dependent, the local Minnesota functionals MN15L and M06L offer reasonable energies. Finally, the M06L functional was chosen as it gives the most reasonable compromise for the relative stability of open and closed-shell species, i.e. minimized overstabilization of the triplet Ni(I) dimer, while also minimizing destabilization of the Ni(I) intermediates (e.g. Int1 vs. Ni(I) dimer **1**).

12.2. Detailed pathway

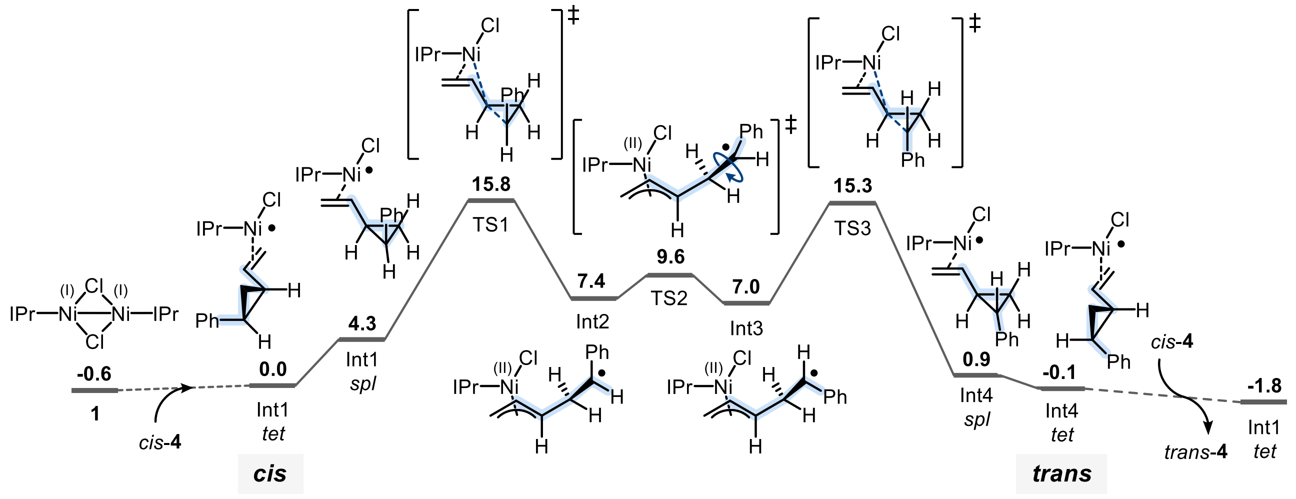

Figure S11. Full pathway of Ni(I)-mediated vinylcyclopropane isomerization. Values refer to Gibbs free energies (in kcal/mol) calculated at the CPCM (dioxane) M06L/def2-TZVP//MN15/6-31G(d)(SDD) level of theory. The descriptions "*tet*" and "*spl*" correspond to tetrahedral and square-planar geometries about Ni, respectively. The intermediates with square-planar geometry are formed *en route* to the transition states for ring-opening (TS1) or, in reverse, after ring-closing (TS3). Both transition states also possess square-planar geometry about Ni.

12.2.1. Orbital analyses

Comment on the broken-symmetry nature of Int2. Since the electronic structure is intimately linked to the geometry we have also optimized intermediate Int2 towards a broken-symmetry solution. To accomplish this, the intermediate was first optimized as a quartet (with MN15/6-31G(d)(SDD), i.e. the same level of theory as for all other geometry optimizations) and then the quartet wave function and geometry were used as input for a reoptimization to the doublet. This resulted in the same intermediate (within 0.4 kcal/mol, no visible geometry differences) that was obtained via IRC calculations from the ring-opening transition state (TS1). The corresponding quartet is at least 10 kcal/mol higher in energy than the doublet (depending on level of theory), and was therefore excluded as an alternative. Although the orbital analysis (based on the doublet geometry) has been performed as a broken-symmetry calculation using B3LYP, the overall multiplicity remains a doublet. Within the ORCA software the broken-symmetry calculation is accomplished by an initial calculation in the higher spin state (quartet in this case), localization of the spin on the respective centers, followed by convergence to the lower spin state wave function (in this case doublet).

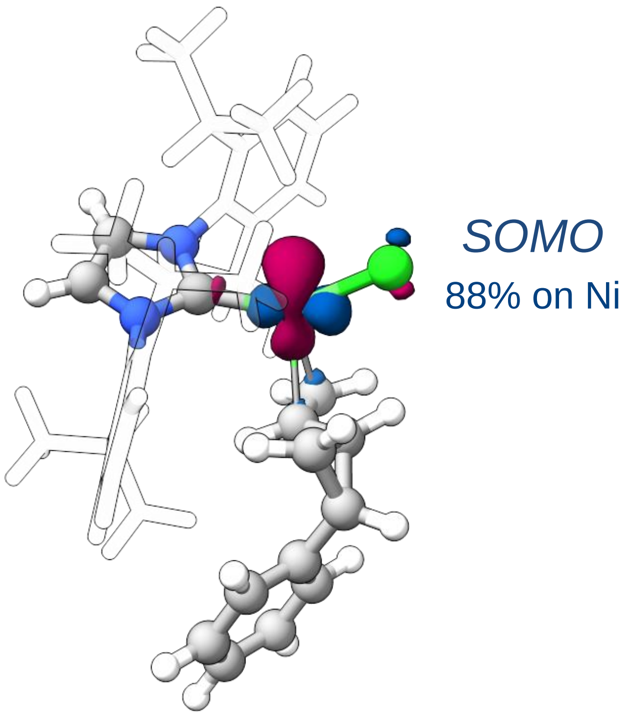

Figure S12. SOMO of the tetrahedral Ni(I) intermediate formed upon coordination of *cis*-**4** (Int1-*tet*). This intermediate has a tetrahedral geometry about Ni, i.e. the olefin resides perpendicular to the NHC-Ni-Cl plane. The displayed orbital has been obtained after localization using the Pipek-Mezey scheme. For clarity, the aryl moieties of the IPr ligand are displayed as wireframe.

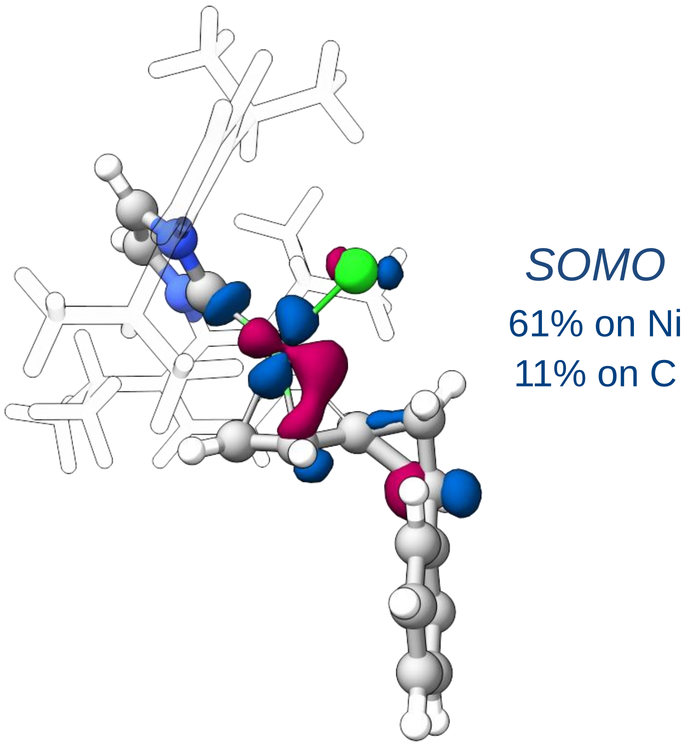

Figure S13. SOMO of the square-planar Ni(I) intermediate formed upon coordination of *cis*-**4** (Int1-*spl*). This intermediate possesses a square-planar geometry about Ni (i.e. the olefin resides in plane with the other ligands) and is 4.3 kcal/mol higher in energy than the corresponding tetrahedral conformation (Int1-*tet*, above). It is formed *en route* to the ring-opening transition state TS1, which also has a square-planar geometry about Ni. The displayed orbital has been obtained after localization using the Pipek-Mezey scheme. For clarity, the aryl moieties of the IPr ligand are displayed as wireframe.

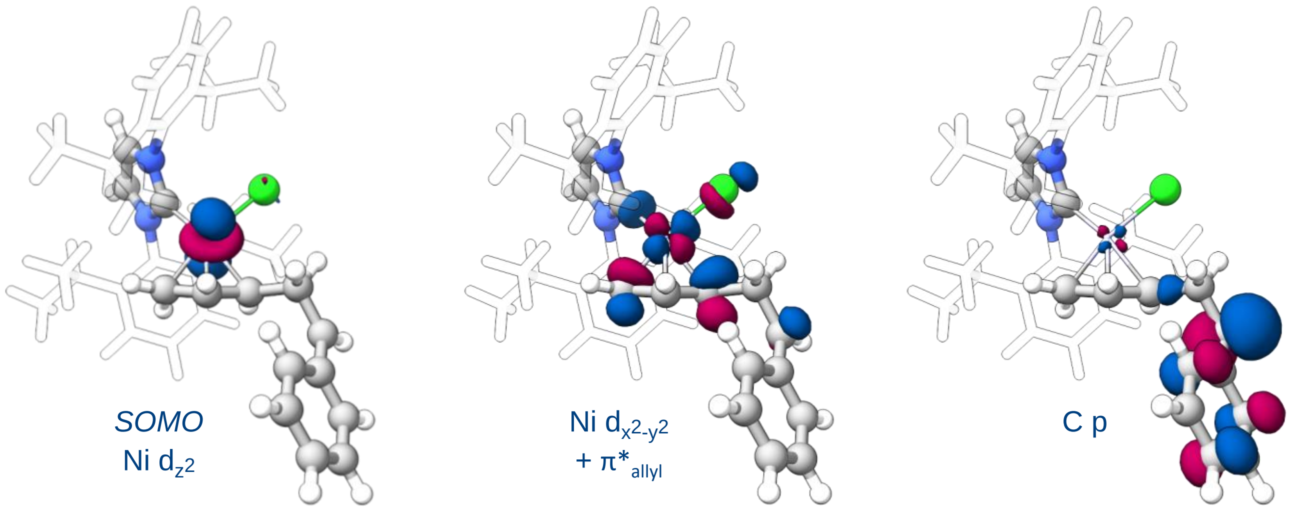

Figure S14. Relevant orbitals of the ring-opened Ni(II) intermediate (Int2): the SOMO corresponds to the d(z²) of Ni (left), the orbitals presented in the middle and right side are those of the antiferrromagnetically coupled alpha and beta spins, respectively. The displayed orbitals have been obtained after localization using the Pipek-Mezey scheme. For clarity, the aryl moieties of the IPr ligand are displayed as wireframe.

12.3. Mechanistic alternatives

12.3.1. Ni(I)/Ni(III) pathway

For an oxidative addition to take place the substrate needs to coordinate in an endo-fashion at Ni(I) (as opposed to the favoured exo-coordination in **Int1**). The corresponding ring-opening **TS1'** is slightly higher in energy compared to **TS1** (*i.e.* the exo analog). The intermediate resulting from this formal oxidative addition is however not a cyclic Ni(III), but instead also an open Ni(II), in analogy to **Int2** of the exo-pathway (see Figure S15).

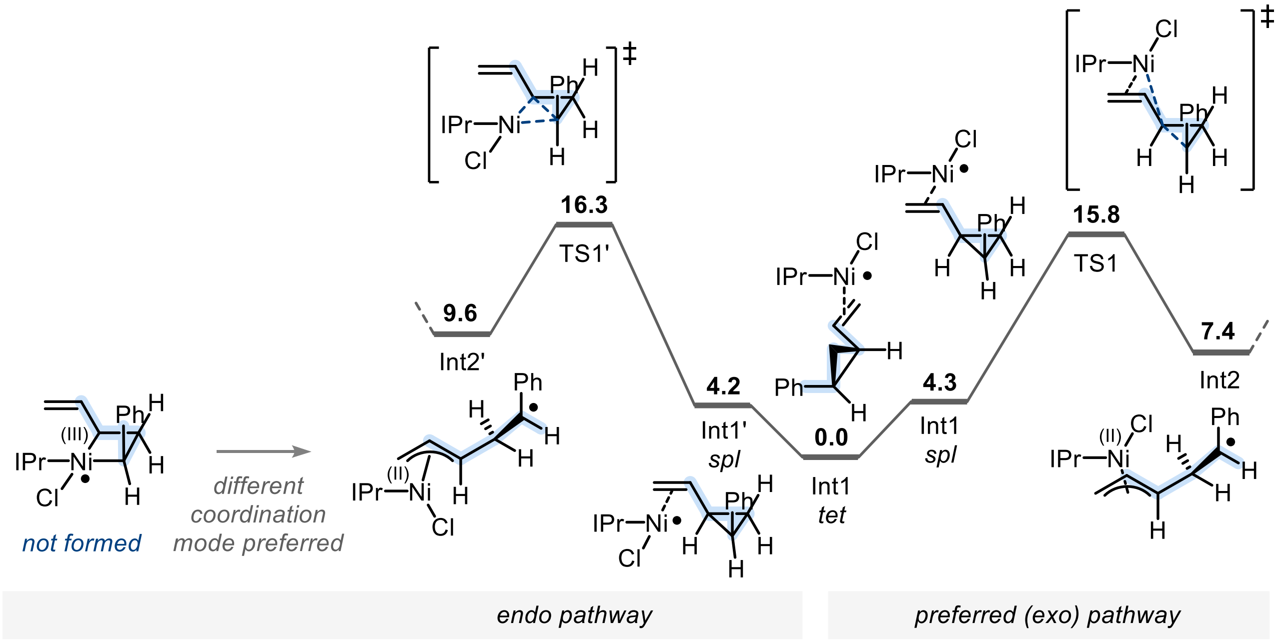

Figure S15. Comparison of alternative Ni(I)-pathways for vinylcyclopropane isomerization. Values refer to Gibbs free energies (in kcal/mol) calculated at the CPCM (dioxane) M06L/def2-TZVP//MN15/6-31G(d)(SDD) level of theory. The descriptions "*tet*" and "*spl*" correspond to tetrahedral and square-planar geometries about Ni, respectively. Both transition states also possess square-planar geometry about Ni.

12.3.2. Other isomers of intermediate Int2

To support the proposed conformational locking of the allyl carbons alternative coordinations of **Int2** were explored. Both alternative η[1]-coordinations (on either of the terminal carbons of the allyl) are energetically severly disfavored, and lie even higher than the transition state for ring-closing **TS3** (15.3 kcal/mol relative to **Int1**).

Note: **Int2'$_{eta1}$** could not be fully optimized, but its energy was estimated based on scans.

Note2: No η[1]-analog of **Int1** could be located. All efforts in this regard directly resulted in ring-opening (to form **Int2**), indicating that no olefin isomerization can take place at the ring-closed stage either as an η[2]-coordination is strongly preferred.

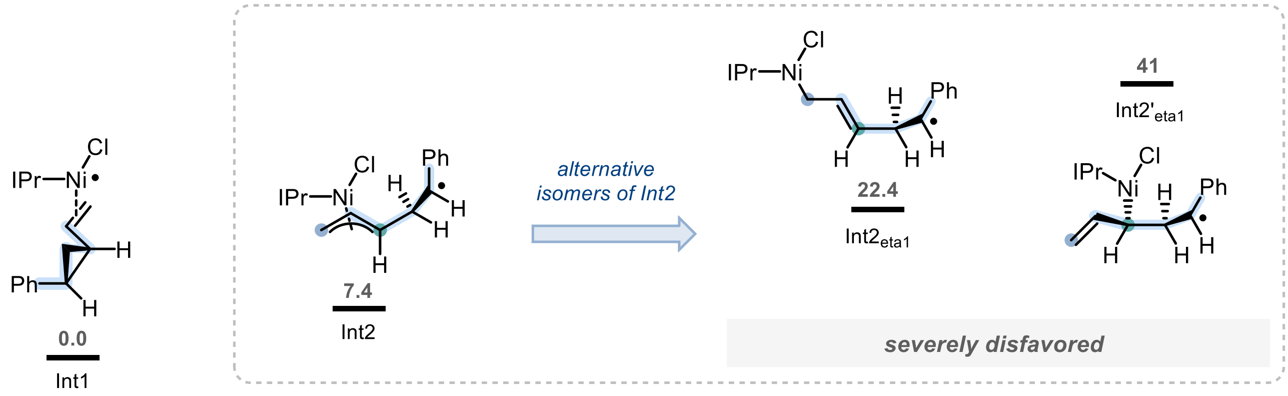

Figure S16. Alternative coordination modes of intermediate **Int2**. Values refer to Gibbs free energies (in kcal/mol) calculated at the CPCM (dioxane) M06L/def2-TZVP//MN15/6-31G(d)(SDD) level of theory.

12.4. XYZ Coordinates and Energies of Optimized Structures

Table S15. Overview of energies of all structures.

Structure	thermal corr. to G	CPCM (PhCl) MN15/def2TZVP	CPCM (PhCl) M06L/def2TZVP	CPCM (dioxane) M06L/def2TZVP	CPCM (dioxane) MN15L/def2TZVP	CPCM (dioxane) M06/def2TZVP	CPCM (dioxane) B3LYP-D3/def2TZVP	CPCM (dioxane) PBE0-D3/def2TZVP	CPCM (dioxane) TPSS-D3/def2TZVP	CPCM (dioxane) TPSSh-D3/def2TZVP	CPCM (dioxane) B97-D3/def2TZVP	CPCM (dioxane) wB97XD/def2TZVP
**1**	1.045385	-6255.59668252	-6257.86370025	-6257.85789894	-6255.96583417	-6256.32691273	-6258.72252082	-6254.84333333	-6259.08904499	-6258.74947388	-6258.25948790	-6257.61931445
^{3}**1**	1.046774	-6255.63198224	-6257.87582803	-6257.86945884	-6255.97758820	-6256.36156609	-6258.75240377	-6254.88201595	-6259.07799818	-6258.75812416	-6258.25261418	-6257.66310827
**3**	0.593403	-3375.89149594	-3377.28811211	-3377.28072900	-3376.14924693	-3376.37180446	-3377.78479206	-3375.53677550	-3377.98375407	-3377.79401212	-3377.34872313	-3377.12815995
pyridine	0.062148	-248.06628994	-248.33913354	-248.33776293	-248.15691158	-248.18338838	-248.39861182	-248.08648825	-248.43411766	-248.40476675	-248.21278325	-248.28579264
cis-**4**	0.161302	-425.95005852	-426.43760003	-426.43666476	-426.11371706	-426.15488489	-426.54670810	-425.99870669	-426.60710213	-426.56175517	-426.21668729	-426.35263647
**Int1**-*tet*	0.695871	-3553.77329174	-3555.38179390	-3555.37509681	-3554.11168101	-3554.34059877	-3555.92720927	-3553.44489416	-3556.15224252	-3555.94643055	-3555.34887534	-3555.19291869
**Int1**-*spl*	0.698602			-3555.37093364	-3554.10508132							
**TS1**	0.693727	-3553.73296255	-3555.35293555	-3555.34769708	-3554.08223648	-3554.29956809	-3555.88969452	-3553.40646427	-3556.13588858	-3555.92048841	-3555.32496092	-3555.14661655
**Int2**	0.694756	-3553.74765028	-3555.36666021	-3555.36159945	-3554.09478160	-3554.31425453	-3555.90364536	-3553.41976696	-3556.14924200	-3555.93481322	-3555.33708965	-3555.16302225
η^1-**Int2**	0.692987			-3555.33648218	-3554.07131581							
η^1-**Int2'**	0.692317			-3555.30643692								
**TS2**	0.696719	-3553.74641108	-3555.36552660	-3555.36068215	-3554.09258168	-3554.31313857	-3555.90255217	-3553.41903915	-3556.14794754	-3555.93369688	-3555.33552044	-3555.16196818
**Int3**	0.696384	-3553.75071575	-3555.36906789	-3555.36448863	-3554.09802036	-3554.31749795	-3555.90756733	-3553.42364373	-3556.15230469	-3555.93814503	-3555.34067274	-3555.16672781
**TS3**	0.695127	-3553.73554266	-3555.35527946	-3555.35004622	-3554.08627105	-3554.30117719	-3555.89139313	-3553.40840215	-3556.13833641	-3555.92287547	-3555.32733492	-3555.14883956
**Int4**-*spl*	0.697301			-3555.37503774	-3554.11043273							
**Int4**-*tet*	0.693799	-3553.77017165	-3555.37995334	-3555.37319861	-3554.10647644	-3554.33815102	-3555.92440812	-3553.44290162	-3556.15063930	-3555.94453530	-3555.34581420	-3555.18879037
trans-**4**	0.161587	-425.95241357	-426.44098445	-426.43988103	-426.11662173	-426.15726802	-426.54880983	-426.00095114	-426.60962870	-426.56408496	-426.21884730	-426.35440371
**Int1'**-*spl*	0.697633			-3555.37010681								
**TS1'**	0.698361			-3555.35159176								
**Int2'**	0.699608			-3555.36348819								
**Ni$^{(0)}$(IPr)$_2$**	1.056396			-3828.97175001								
**IPr**	0.515108			-1160.28735805								
**Int5**	0.694991			-3095.09683193								
**TS4**	0.700930			-3095.07765443								
**Int6**	0.699230			-3095.08868722								
**Int7**	0.700830			-3095.09273229								
**TS6**	0.699620			-3095.06095433								
**Int8**	0.702298			-3095.07759719								
**TS7**	0.695549			-3095.06424229								
**Int9**	0.699578			-3095.12088741								

Ni[(I)] dimer (1)

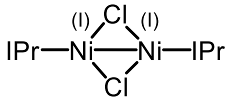

Ni	1.08882	0.00007	0.00002
Ni	-1.19313	-0.00001	-0.00015
Cl	-0.02540	-1.19346	-1.51473
C	2.97864	0.00043	0.00085
C	5.14810	0.48126	-0.47687
C	5.14760	-0.47932	0.48193
C	-3.10365	-0.00016	-0.00043
C	-5.27567	-0.63731	-0.23372
C	-5.27579	0.63674	0.23240
N	3.81237	-0.76156	0.75576
N	3.81315	0.76285	-0.75271
N	-3.94260	1.00377	0.37759
N	-3.94240	-1.00419	-0.37864
H	5.95694	-0.98113	0.99321
H	5.95795	0.98337	-0.98705
H	-6.08569	1.30986	0.47460
H	-6.08544	-1.31053	-0.47611
C	3.28996	-1.62718	1.77659
C	2.76258	-2.87538	1.39845
C	3.24632	-1.14271	3.09214
C	2.23687	-3.67891	2.41428
C	2.71978	-1.98927	4.07450
C	2.22475	-3.24600	3.74015
H	1.82049	-4.65182	2.16496
H	2.68509	-1.65074	5.10823
H	1.81188	-3.88911	4.51414
C	3.29194	1.62757	-1.77488
C	2.76309	2.87564	-1.39837
C	3.25092	1.14231	-3.09021
C	2.23828	3.67810	-2.41552
C	2.72527	1.98785	-4.07394
C	2.22859	3.24433	-3.74114
H	1.82078	4.65085	-2.16747
H	2.69258	1.64864	-5.10753
H	1.81641	3.88662	-4.51618
C	-3.43949	2.26502	0.85223
C	-3.47457	2.52102	2.23463
C	-2.85294	3.14374	-0.07142
C	-2.90008	3.71232	2.68460
C	-2.28920	4.32296	0.42993
C	-2.30763	4.60414	1.79177
H	-2.89977	3.93918	3.74783
H	-1.81876	5.02140	-0.26072
H	-1.85493	5.52083	2.16274
C	-3.43908	-2.26527	-0.85352
C	-3.47403	-2.52097	-2.23598
C	-2.85244	-3.14412	0.06996
C	-2.89941	-3.71211	-2.68618
C	-2.28853	-4.32316	-0.43162
C	-2.30691	-4.60406	-1.79352
H	-2.89902	-3.93872	-3.74947
H	-1.81804	-5.02168	0.25889
H	-1.85410	-5.52063	-2.16467
C	2.79207	-3.33640	-0.05072
H	2.66897	-2.44818	-0.68453
C	1.64527	-4.28649	-0.40071
H	1.62830	-4.45796	-1.48359
H	1.76121	-5.26376	0.08828
H	0.67847	-3.85325	-0.11894
C	4.15061	-3.97467	-0.37263
H	4.31660	-4.85834	0.25793
H	4.18774	-4.29099	-1.42255
H	4.97553	-3.27231	-0.19779
C	3.75445	-0.24535	-3.45319
H	3.89676	-0.81340	-2.52343
C	5.10689	-0.14817	-4.17094
H	5.00301	0.42718	-5.10068
H	5.85776	0.35433	-3.54834
H	5.48311	-1.14578	-4.42832
C	2.74082	-1.01870	-4.30364
H	1.76707	-1.06734	-3.80424
H	2.60605	-0.55354	-5.28886
H	3.10015	-2.04189	-4.47020
C	2.79041	3.33773	0.05048
H	2.66756	2.44986	0.68482
C	4.14799	3.97762	0.37329
H	4.97376	3.27594	0.19970
H	4.31371	4.86100	-0.25775
H	4.18382	4.29469	1.42303
C	1.64226	4.28690	0.39851
H	0.67624	3.85233	0.11618
H	1.62401	4.45931	1.48122
H	1.75761	5.26386	-0.09124
C	3.74827	0.24513	3.45662
H	3.88913	0.81448	2.52743
C	5.10131	0.14871	4.17337
H	5.47644	1.14645	4.43185
H	4.99863	-0.42801	5.10240
H	5.85246	-0.35203	3.54971
C	2.73445	1.01622	4.30889
H	3.09238	2.03986	4.47567
H	1.76001	1.06372	3.81073
H	2.60171	0.55024	5.29399
C	-2.79344	2.85955	-1.56365
H	-3.29300	1.90175	-1.75472
C	-3.53486	3.94060	-2.36081
H	-4.58033	4.02983	-2.04294
H	-3.05754	4.92064	-2.23075
H	-3.51713	3.70044	-3.43110
C	-1.34060	2.73937	-2.04557
H	-0.77832	1.99064	-1.46734
H	-1.30846	2.44671	-3.10349
H	-0.81771	3.70085	-1.94570
C	-4.02292	1.49507	3.21529
H	-4.79976	0.91053	2.70449
C	-4.66211	2.12668	4.45509
H	-5.16260	1.35385	5.05012
H	-3.90606	2.59101	5.10062
H	-5.40055	2.89225	4.18901
C	-2.90437	0.52599	3.62819
H	-3.29602	-0.25026	4.29981
H	-2.44374	0.04681	2.75636
H	-2.10825	1.07279	4.15065
C	-2.79297	-2.86023	1.56225
H	-3.29256	-1.90248	1.75352
C	-3.53434	-3.94148	2.35917
H	-3.05698	-4.92147	2.22889
H	-3.51662	-3.70157	3.42951
H	-4.57981	-4.03070	2.04128
C	-1.34012	-2.74012	2.04415
H	-0.81719	-3.70154	1.94391
H	-0.77788	-1.99119	1.46615
H	-1.30793	-2.44784	3.10217
C	-4.02229	-1.49481	-3.21648
H	-4.79906	-0.91025	-2.70561
C	-4.66153	-2.12616	-4.45639
H	-5.40010	-2.89165	-4.19043
H	-5.16186	-1.35318	-5.05134
H	-3.90553	-2.59053	-5.10195
C	-2.90358	-0.52584	-3.62917
H	-2.44282	-0.04699	-2.75723
H	-2.10758	-1.07265	-4.15182
H	-3.29509	0.25068	-4.30056
Cl	-0.02558	1.19353	1.51471

Zero-point correction = 1.154205 (Hartree/Particle)
Thermal correction to Energy = 1.223281
Thermal correction to Enthalpy = 1.224226
Thermal correction to Gibbs Free Energy = 1.045385
Sum of electronic and zero-point Energies = -3578.433960
Sum of electronic and thermal Energies = -3578.364884
Sum of electronic and thermal Enthalpies = -3578.363940
Sum of electronic and thermal Free Energies = -3578.542780
E(CPCM(PhCl) MN15/def2-TZVP) = -6255.59668252
E(CPCM(PhCl) M06L/def2-TZVP) = -6257.86370025
E(CPCM(dioxane) M06L/def2-TZVP) = -6257.85789894
E(CPCM(dioxane) MN15L/def2-TZVP) = -6255.96583417
E(CPCM(dioxane) M06/def2-TZVP) = -6256.32691273
E(CPCM(dioxane) B3LYP-D3/def2-TZVP) = -6258.72252082

E(CPCM(dioxane) PBE0-D3/def2-TZVP) = -6254.84333333
E(CPCM(dioxane) TPSS-D3/def2-TZVP) = -6259.08904499
E(CPCM(dioxane) TPSSh-D3/def2-TZVP) = -6258.74947388
E(CPCM(dioxane) B97-D3/def2-TZVP) = -6258.25948790
E(CPCM(dioxane) wB97XD/def2-TZVP) = -6257.61931445

Ni(I) dimer, triplet (³1)

Ni	1.31774	0.90928	0.05669
Ni	-1.45027	0.56326	0.30257
Cl	0.18651	-1.13311	0.18989
C	3.11451	0.40884	-0.45242
C	4.83992	-0.20706	-1.80662
C	5.34863	-0.01620	-0.56491
C	-3.20139	-0.29375	0.39970
C	-5.04381	-1.59536	0.67744
C	-5.47879	-0.34890	0.35942
N	4.27971	0.35679	0.24790
N	3.47572	0.04926	-1.71706
N	-4.33917	0.42655	0.20154
N	-3.65523	-1.54128	0.68918
H	6.35482	-0.10398	-0.18031
H	5.30867	-0.50056	-2.73513
H	-6.47377	0.05289	0.23347
H	-5.58064	-2.50809	0.89276
C	4.31882	0.48186	1.68051
C	3.87315	-0.61032	2.44943
C	4.77111	1.68525	2.24359
C	3.92242	-0.47102	3.84043
C	4.80399	1.77250	3.63909
C	4.38820	0.70295	4.42866
H	3.58659	-1.29096	4.47030
H	5.14997	2.68766	4.11296
H	4.41854	0.78857	5.51253
C	2.47772	-0.28405	-2.69819
C	1.77913	0.75563	-3.33674
C	2.18130	-1.64269	-2.89671
C	0.77830	0.39228	-4.24181
C	1.18656	-1.95359	-3.83012
C	0.48786	-0.94863	-4.49116
H	0.20927	1.16578	-4.75136
H	0.94150	-2.99698	-4.02048
H	-0.29809	-1.20929	-5.19763
C	-4.24838	1.81651	-0.15405
C	-4.33311	2.77668	0.86814
C	-3.95721	2.14150	-1.48807
C	-4.12983	4.11298	0.51386
C	-3.76191	3.49306	-1.79285
C	-3.84698	4.46833	-0.80375
H	-4.17110	4.88274	1.28105
H	-3.52496	3.77907	-2.81621
H	-3.67989	5.51239	-1.05737
C	-2.76890	-2.62894	1.00488
C	-2.44777	-3.54278	-0.01134
C	-2.19622	-2.67059	2.28391
C	-1.50904	-4.53368	0.28804
C	-1.26732	-3.68539	2.53791
C	-0.92200	-4.60320	1.55036
H	-1.21610	-5.24533	-0.48106
H	-0.79192	-3.73996	3.51628
H	-0.18470	-5.37469	1.76250
C	3.39505	-1.90117	1.79980
H	2.91362	-1.63997	0.84984
C	2.33587	-2.64140	2.61865
H	1.91735	-3.45467	2.01189
H	2.75753	-3.08256	3.53281
H	1.50693	-1.97555	2.88440
C	4.58470	-2.82623	1.50449
H	5.08610	-3.11679	2.43758
H	4.23819	-3.73975	1.00271
H	5.32503	-2.34271	0.85524
C	2.90397	-2.75646	-2.15211
H	3.45707	-2.30881	-1.31547
C	3.91391	-3.44310	-3.08224
H	3.39202	-3.90660	-3.93026
H	4.64316	-2.73191	-3.48932
H	4.46002	-4.23007	-2.54747
C	1.94717	-3.79574	-1.55289
H	1.20293	-3.32355	-0.90268
H	1.42158	-4.35839	-2.33747
H	2.52141	-4.52250	-0.96180
C	2.14171	2.21232	-3.08947
H	2.44630	2.30889	-2.03787
C	3.33858	2.59853	-3.97033
H	4.20434	1.95330	-3.77372
H	3.07510	2.49937	-5.03177
H	3.63569	3.63830	-3.78611
C	0.97363	3.17629	-3.30109
H	0.10995	2.88871	-2.68971
H	1.27564	4.18696	-3.00219
H	0.66977	3.22345	-4.35584
C	5.12245	2.87162	1.36082
H	5.53441	2.48456	0.41859
C	6.16789	3.80162	1.98130
H	6.48070	4.55247	1.24663
H	5.75672	4.34391	2.84218
H	7.05607	3.25256	2.31564
C	3.84383	3.65887	1.03114
H	4.07044	4.50557	0.37062
H	3.08935	3.02755	0.54303
H	3.39746	4.05072	1.95431
C	-3.81285	1.08796	-2.57396
H	-4.01797	0.10093	-2.13638
C	-4.81740	1.32068	-3.70928
H	-5.84928	1.32300	-3.33919
H	-4.63401	2.28368	-4.20304
H	-4.72313	0.53419	-4.46783
C	-2.37877	1.06516	-3.11558
H	-1.64432	0.89360	-2.31380
H	-2.26352	0.26472	-3.85796
H	-2.12880	2.02002	-3.59937
C	-4.52918	2.37181	2.31989
H	-4.98215	1.37072	2.34048
C	-5.45880	3.32058	3.08279
H	-5.65702	2.92345	4.08525
H	-5.00118	4.30935	3.21129
H	-6.41676	3.45310	2.56665
C	-3.16158	2.28549	3.01370
H	-3.27717	1.96541	4.05813
H	-2.49561	1.57787	2.50158
H	-2.66591	3.26516	2.99835
C	-2.51934	-1.65158	3.36529
H	-3.25400	-0.93742	2.96771
C	-3.13976	-2.33129	4.59249
H	-2.43428	-3.04164	5.04285
H	-3.39354	-1.58438	5.35473
H	-4.05065	-2.88211	4.32982
C	-1.26653	-0.85878	3.76074
H	-0.52054	-1.52034	4.22190
H	-0.80196	-0.38531	2.88540
H	-1.52299	-0.08046	4.49089
C	-3.01314	-3.40018	-1.41573
H	-3.95560	-2.83841	-1.35383
C	-3.31944	-4.74865	-2.07448
H	-3.94843	-5.38101	-1.43694
H	-3.83943	-4.58956	-3.02660
H	-2.39789	-5.30075	-2.29824
C	-2.03466	-2.59124	-2.27919
H	-1.83603	-1.60231	-1.84912
H	-1.06844	-3.11126	-2.33523
H	-2.42570	-2.46906	-3.29965
Cl	-0.26656	2.56268	0.33585

Zero-point correction = 1.153957 (Hartree/Particle)
Thermal correction to Energy = 1.223020
Thermal correction to Enthalpy = 1.223964
Thermal correction to Gibbs Free Energy = 1.046774
Sum of electronic and zero-point Energies = -3578.475536
Sum of electronic and thermal Energies = -3578.406473
Sum of electronic and thermal Enthalpies = -3578.405529
Sum of electronic and thermal Free Energies = -3578.582719
E(CPCM(PhCl) MN15/def2-TZVP) = -6255.63198224
E(CPCM(PhCl) M06L/def2-TZVP) = -6257.87582803
E(CPCM(dioxane) M06L/def2-TZVP) = -6257.86945884
E(CPCM(dioxane) MN15L/def2-TZVP) = -6255.97758820

E(CPCM(dioxane) M06/def2-TZVP) = -6256.36156609
E(CPCM(dioxane) B3LYP-D3/def2-TZVP) = -6258.75240377
E(CPCM(dioxane) PBE0-D3/def2-TZVP) = -6254.88201595
E(CPCM(dioxane) TPSS-D3/def2-TZVP) = -6259.07799818
E(CPCM(dioxane) TPSSh-D3/def2-TZVP) = -6258.75812416
E(CPCM(dioxane) B97-D3/def2-TZVP) = -6258.25261418
E(CPCM(dioxane) wB97XD/def2-TZVP) = -6257.66310827

[Ni^(I)(Cl)(IPr)(pyridine)] (3)

Ni	0.35755	0.11922	1.33122
Cl	1.53197	0.35875	3.19844
N	1.44086	0.23014	-1.37233
N	-0.62719	0.74707	-1.52420
N	-1.29346	-0.98501	1.90375
C	0.31218	0.35981	-0.61742
C	1.20918	0.52187	-2.71058
H	1.99239	0.46664	-3.45332
C	-0.10295	0.84865	-2.80905
H	-0.71063	1.14021	-3.65378
C	2.68472	-0.21094	-0.79563
C	3.46177	0.71606	-0.07982
C	4.63846	0.24123	0.50697
H	5.25971	0.91993	1.08394
C	5.01250	-1.09418	0.38390
H	5.92972	-1.44214	0.85307
C	4.21229	-1.99205	-0.31865
H	4.51079	-3.03497	-0.39022
C	3.02165	-1.56938	-0.91691
C	3.04905	2.17660	0.03003
H	1.95197	2.20570	0.11706
C	3.61339	2.87385	1.26865
H	4.70155	3.00524	1.19498
H	3.37511	2.30841	2.17596
H	3.17119	3.87369	1.35563
C	3.45601	2.93537	-1.24210
H	3.00543	2.50010	-2.14228
H	4.54713	2.90936	-1.36158
H	3.14592	3.98578	-1.17841
C	2.08088	-2.55704	-1.59181
H	1.52693	-2.02489	-2.37841
C	1.05621	-3.07542	-0.56951
H	1.57487	-3.59940	0.24498
H	0.35526	-3.77675	-1.04295
H	0.48221	-2.25015	-0.12799
C	2.80809	-3.73207	-2.25082
H	3.24737	-4.40011	-1.49939
H	3.61060	-3.39293	-2.91606
H	2.09936	-4.32789	-2.83772
C	-2.00975	0.91298	-1.18769
C	-2.42900	2.12529	-0.61500
C	-3.78384	2.24418	-0.28191
H	-4.14134	3.16539	0.17394
C	-4.67628	1.19979	-0.52206
H	-5.72676	1.31608	-0.26515
C	-4.22606	0.00608	-1.08224
H	-4.92681	-0.80927	-1.25463
C	-2.87758	-0.16749	-1.41455
C	-1.45374	3.24621	-0.29615
H	-0.50673	3.03229	-0.80922
C	-1.17518	3.28180	1.21374
H	-0.75292	2.33083	1.57105
H	-2.10351	3.47600	1.76904
H	-0.45916	4.07715	1.45519
C	-1.95880	4.60862	-0.78443
H	-2.85756	4.92065	-0.23747
H	-2.20018	4.59256	-1.85344
H	-1.19251	5.37360	-0.61408
C	-2.39503	-1.48735	-1.99691
H	-1.29983	-1.45384	-2.07550
C	-2.74756	-2.67286	-1.09077
H	-2.39828	-3.60742	-1.54769
H	-3.83205	-2.75835	-0.94326
H	-2.27521	-2.57595	-0.10674
C	-2.96829	-1.68450	-3.40609
H	-4.06502	-1.72689	-3.37557
H	-2.60537	-2.62226	-3.84347
H	-2.68276	-0.85972	-4.07062
C	-2.56208	-0.55056	1.87894
H	-2.71946	0.44966	1.48143
C	-3.63168	-1.32627	2.32025
H	-4.64080	-0.92784	2.26455
C	-3.37403	-2.60367	2.81376
H	-4.18551	-3.23876	3.16041
C	-2.05251	-3.04914	2.86524
H	-1.80133	-4.03001	3.25761
C	-1.04311	-2.20561	2.41028
H	0.00655	-2.49095	2.45194

Zero-point correction = 0.667510 (Hartree/Particle)
Thermal correction to Energy = 0.707154
Thermal correction to Enthalpy = 0.708098
Thermal correction to Gibbs Free Energy = 0.593403
Sum of electronic and zero-point Energies = -2037.110310
Sum of electronic and thermal Energies = -2037.070666
Sum of electronic and thermal Enthalpies = -2037.069722
Sum of electronic and thermal Free Energies = -2037.184417
E(CPCM(PhCl) MN15/def2-TZVP) = -3375.89149594
E(CPCM(PhCl) M06L/def2-TZVP) = -3377.28811211
E(CPCM(dioxane) M06L/def2-TZVP) = -3377.28072900
E(CPCM(dioxane) MN15L/def2-TZVP) = -3376.14924693
E(CPCM(dioxane) M06/def2-TZVP) = -3376.37180446
E(CPCM(dioxane) B3LYP-D3/def2-TZVP) = -3377.78479206
E(CPCM(dioxane) PBE0-D3/def2-TZVP) = -3375.53677550
E(CPCM(dioxane) TPSS-D3/def2-TZVP) = -3377.98375407
E(CPCM(dioxane) TPSSh-D3/def2-TZVP) = -3377.79401212
E(CPCM(dioxane) B97-D3/def2-TZVP) = -3377.34872313
E(CPCM(dioxane) wB97XD/def2-TZVP) = -3377.12815995

pyridine

C	-1.14241	-0.72179	-0.00020
C	-1.19845	0.67230	-0.00012
C	0.00012	1.38374	-0.00001
C	1.19857	0.67210	0.00017
C	1.14228	-0.72198	0.00018
N	-0.00012	-1.41769	-0.00007
H	0.00018	2.47124	0.00035
H	-2.06067	-1.30829	-0.00034
H	-2.15806	1.18182	-0.00025
H	2.15829	1.18141	0.00032
H	2.06043	-1.30864	0.00023

Zero-point correction = 0.089560 (Hartree/Particle)
Thermal correction to Energy = 0.093853
Thermal correction to Enthalpy = 0.094797
Thermal correction to Gibbs Free Energy = 0.062148
Sum of electronic and zero-point Energies = -247.871295
Sum of electronic and thermal Energies = -247.867002
Sum of electronic and thermal Enthalpies = -247.866058
Sum of electronic and thermal Free Energies = -247.898707
E(CPCM(PhCl) MN15/def2-TZVP) = -248.066289942
E(CPCM(PhCl) M06L/def2-TZVP) = -248.339133535
E(CPCM(dioxane) M06L/def2-TZVP) = -248.337762929
E(CPCM(dioxane) MN15L/def2-TZVP) = -248.156911582
E(CPCM(dioxane) M06/def2-TZVP) = -248.183388383
E(CPCM(dioxane) B3LYP-D3/def2-TZVP) = -248.398611824
E(CPCM(dioxane) PBE0-D3/def2-TZVP) = -248.086488250
E(CPCM(dioxane) TPSS-D3/def2-TZVP) = -248.434117659
E(CPCM(dioxane) TPSSh-D3/def2-TZVP) = -248.404766745
E(CPCM(dioxane) B97-D3/def2-TZVP) = -248.212783249
E(CPCM(dioxane) wB97XD/def2-TZVP) = -248.285792642

***cis*-4**

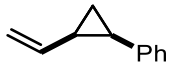

C	-2.09662	0.73513	-0.60783
H	-1.29015	0.72932	-1.34376
C	-2.97131	1.74369	-0.58818
H	-3.78283	1.77646	0.13751
H	-2.91017	2.56761	-1.29399
C	-2.12719	-0.41615	0.32097
C	-0.82657	-1.02397	0.82104
H	-2.95088	-0.40371	1.03377

C	-1.66965	-1.77378	-0.16305
H	-1.30869	-1.82582	-1.18948
H	-2.21552	-2.65297	0.16841
C	0.49267	-0.43982	0.41679
C	1.38901	-1.15842	-0.38026
C	0.85535	0.84193	0.85221
C	2.62659	-0.61486	-0.72977
H	1.11001	-2.15299	-0.72449
C	2.08958	1.38810	0.50570
H	0.14977	1.41263	1.45390
C	2.98013	0.65920	-0.28628
H	3.31356	-1.18712	-1.34919
H	2.35579	2.38563	0.84788
H	3.94267	1.08540	-0.55930
H	-0.86557	-1.38068	1.85069

Zero-point correction = 0.197264 (Hartree/Particle)
Thermal correction to Energy = 0.207024
Thermal correction to Enthalpy = 0.207969
Thermal correction to Gibbs Free Energy = 0.161302
Sum of electronic and zero-point Energies = -425.576590
Sum of electronic and thermal Energies = -425.566830
Sum of electronic and thermal Enthalpies = -425.565886
Sum of electronic and thermal Free Energies = -425.612553
E(CPCM(PhCl) MN15/def2-TZVP) = -425.950058515
E(CPCM(PhCl) M06L/def2-TZVP) = -426.437600029
E(CPCM(dioxane) M06L/def2-TZVP) = -426.436664763
E(CPCM(dioxane) MN15L/def2-TZVP) = -426.113717064
E(CPCM(dioxane) M06/def2-TZVP) = -426.154884887
E(CPCM(dioxane) B3LYP-D3/def2-TZVP) = -426.546708096
E(CPCM(dioxane) PBE0-D3/def2-TZVP) = -425.998706694
E(CPCM(dioxane) TPSS-D3/def2-TZVP) = -426.607102131
E(CPCM(dioxane) TPSSh-D3/def2-TZVP) = -426.561755165
E(CPCM(dioxane) B97-D3/def2-TZVP) = -426.216687290
E(CPCM(dioxane) wB97XD/def2-TZVP) = -426.352636467

Int1-*tet*

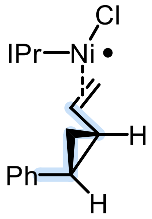

Ni	-0.76616	-0.49927	-1.15336
C	3.38013	-0.52781	-2.60937
C	1.89030	-0.76113	-2.40023
H	1.30961	-1.06118	-3.27065
C	1.42613	-1.33766	-1.11949
C	0.56484	-2.37748	-1.01505
H	0.32832	-2.80694	-0.04389
H	0.15642	-2.86762	-1.89802
H	1.87041	-0.91300	-0.21743
C	-0.99798	0.24204	0.64545
Cl	-1.61186	-0.71698	-3.19479
N	-2.17218	0.02293	1.30528
N	-0.29542	1.03635	1.50025
C	-2.21132	0.67758	2.52797
C	-1.02631	1.32462	2.65035
H	-3.07424	0.63138	3.17707
H	-0.63830	1.96764	3.42667
C	-3.27418	-0.62799	0.64431
C	-4.19506	0.17809	-0.04149
C	-3.32946	-2.03216	0.63424
C	-5.21384	-0.46679	-0.75099
C	-4.36664	-2.62945	-0.08608
C	-5.29875	-1.85451	-0.77465
H	-5.93241	0.12944	-1.31013
H	-4.44004	-3.71320	-0.12372
H	-6.08902	-2.33929	-1.34250
C	0.97539	1.63237	1.19021
C	2.14380	1.06136	1.72929
C	0.99283	2.77544	0.37371
C	3.35545	1.71193	1.47812
C	2.23230	3.38958	0.15124
C	3.39838	2.87480	0.70922
H	4.27840	1.29569	1.87457
H	2.27924	4.27605	-0.47854
H	4.35104	3.36774	0.52741
C	-2.32133	-2.85969	1.41256
H	-1.37364	-2.30328	1.42174
C	-2.05512	-4.22606	0.77679
H	-2.91654	-4.89646	0.89160
H	-1.83584	-4.12994	-0.29362
H	-1.20124	-4.70872	1.26782
C	-2.78837	-3.02205	2.86606
H	-2.06518	-3.60998	3.44468
H	-2.91097	-2.04883	3.35732
H	-3.75515	-3.54115	2.89559
C	-4.08079	1.69275	-0.08332
H	-3.27774	2.00136	0.59893
C	-5.37189	2.37058	0.38968
H	-6.21057	2.12818	-0.27499
H	-5.64398	2.05614	1.40436
H	-5.25141	3.46061	0.38543
C	-3.70009	2.15696	-1.49477
H	-3.51809	3.24106	-1.50263
H	-2.80488	1.63721	-1.85866
H	-4.51101	1.94156	-2.20306
C	2.09887	-0.20671	2.56917
H	1.18098	-0.75112	2.29922
C	3.29162	-1.13502	2.31032
H	3.13473	-2.08787	2.82985
H	3.43962	-1.34320	1.24459
H	4.22527	-0.70562	2.69585
C	2.03837	0.14134	4.06364
H	2.93210	0.71186	4.34876
H	1.15823	0.74490	4.31068
H	2.00836	-0.77150	4.67077
C	-0.24938	3.32031	-0.31268
H	-1.12791	2.78580	0.07167
C	-0.45452	4.81425	-0.03774
H	0.35016	5.41663	-0.47827
H	-1.39803	5.14763	-0.48708
H	-0.48886	5.02716	1.03698
C	-0.16282	3.05165	-1.82238
H	0.74458	3.50850	-2.24187
H	-0.13620	1.97407	-2.03950
H	-1.03094	3.47794	-2.33918
C	4.37735	-0.93397	-1.56800
C	5.15180	0.01896	-0.89796
C	4.57398	-2.29020	-1.26961
C	6.10973	-0.37024	0.04098
H	4.99538	1.07315	-1.11986
C	5.52792	-2.68304	-0.33134
H	3.96351	-3.03457	-1.77855
C	6.30155	-1.72249	0.32574
H	6.70785	0.38444	0.54848
H	5.66759	-3.73887	-0.11088
H	3.70984	-0.69394	-3.63553
C	2.46421	0.63637	-2.39382
H	2.51245	1.13169	-1.42302
H	7.04712	-2.02780	1.05626
H	2.22545	1.29018	-3.22843

Zero-point correction = 0.775554 (Hartree/Particle)
Thermal correction to Energy = 0.820369
Thermal correction to Enthalpy = 0.821313
Thermal correction to Gibbs Free Energy = 0.695871
Sum of electronic and zero-point Energies = -2214.816385
Sum of electronic and thermal Energies = -2214.771570
Sum of electronic and thermal Enthalpies = -2214.770626
Sum of electronic and thermal Free Energies = -2214.896068
E(CPCM(PhCl) MN15/def2-TZVP) = -3553.77329174
E(CPCM(PhCl) M06L/def2-TZVP) = -3555.38179390
E(CPCM(dioxane) M06L/def2-TZVP) = -3555.37509681
E(CPCM(dioxane) MN15L/def2-TZVP) = -3554.11168101
E(CPCM(dioxane) M06/def2-TZVP) = -3554.34059877
E(CPCM(dioxane) B3LYP-D3/def2-TZVP) = -3555.92720927
E(CPCM(dioxane) PBE0-D3/def2-TZVP) = -3553.44489416
E(CPCM(dioxane) TPSS-D3/def2-TZVP) = -3556.15224252
E(CPCM(dioxane) TPSSh-D3/def2-TZVP) = -3555.94643055
E(CPCM(dioxane) B97-D3/def2-TZVP) = -3555.34887534

E(CPCM(dioxane) wB97XD/def2-TZVP) = -3555.19291869

Int1-*spl*

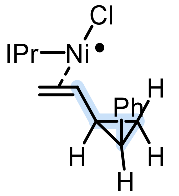

Ni	-0.35406	-0.19115	-0.24882
C	-4.33152	-0.34352	-1.66585
C	-2.62021	0.08487	-1.01446
H	-2.38205	1.09746	-1.34559
C	-2.29939	-0.23423	0.36776
C	-1.62533	0.63779	1.20561
H	-1.41666	0.35158	2.23329
H	-1.53798	1.69866	0.96465
H	-2.55781	-1.23411	0.72217
C	-3.02359	-0.91417	-2.03566
C	1.40654	0.40992	0.35385
Cl	0.22744	-1.43654	-2.02496
N	1.80395	1.71465	0.36945
N	2.54526	-0.28329	0.62414
C	3.16153	1.83446	0.64417
C	3.62391	0.57467	0.81940
H	3.66149	2.79180	0.67826
H	4.60846	0.20195	1.05947
C	0.87712	2.77574	0.08392
C	0.36117	2.86138	-1.22409
C	0.46952	3.62688	1.12648
C	-0.66959	3.78266	-1.44582
C	-0.55002	4.54373	0.84561
C	-1.13230	4.60261	-0.41881
H	-1.10626	3.86573	-2.43770
H	-0.90072	5.21369	1.62575
H	-1.93632	5.30933	-0.61042
C	2.68552	-1.71672	0.58550
C	2.09445	-2.49869	1.58947
C	3.43553	-2.27744	-0.46588
C	2.31069	-3.88085	1.54880
C	3.63943	-3.65990	-0.44802
C	3.08803	-4.45791	0.55073
H	1.86053	-4.50956	2.31530
H	4.22548	-4.11876	-1.24161
H	3.25259	-5.53278	0.54129
C	1.07818	3.52830	2.52016
H	2.15263	3.33403	2.40640
C	0.47959	2.35086	3.30086
H	-0.60253	2.49220	3.42313
H	0.64037	1.40034	2.77958
H	0.93261	2.28399	4.29769
C	0.93315	4.81658	3.33394
H	1.52272	4.73938	4.25445
H	1.27713	5.69492	2.77551
H	-0.10996	4.98517	3.63041
C	0.97340	2.07072	-2.37180
H	1.26388	1.07950	-2.00648
C	2.24183	2.79308	-2.85186
H	1.99751	3.79679	-3.22469
H	2.97321	2.89975	-2.04041
H	2.71433	2.22884	-3.66504
C	0.01647	1.83030	-3.53880
H	0.48357	1.14042	-4.24959
H	-0.91394	1.36036	-3.19678
H	-0.22315	2.75936	-4.07424
C	1.21028	-1.92244	2.68189
H	1.16960	-0.83349	2.56138
C	-0.21776	-2.46026	2.52187
H	-0.90145	-1.96855	3.22688
H	-0.57684	-2.29009	1.49869
H	-0.25017	-3.54049	2.71634
C	1.75742	-2.22742	4.08082
H	1.78966	-3.30912	4.26368
H	2.77230	-1.83400	4.20900
H	1.11347	-1.77974	4.84779
C	4.01621	-1.45075	-1.60493
H	3.55712	-0.45254	-1.57597
C	5.53782	-1.31034	-1.45378
H	6.00996	-2.30121	-1.49153
H	5.95200	-0.70833	-2.27178
H	5.82625	-0.84358	-0.50457
C	3.69206	-2.05771	-2.97589
H	4.20899	-3.01546	-3.12020
H	2.61532	-2.21203	-3.08615
H	4.03560	-1.37972	-3.76732
H	-2.82747	-1.95035	-1.75576
C	-5.32756	-0.95313	-0.77785
C	-6.53256	-0.27028	-0.51265
C	-5.12572	-2.19738	-0.15046
C	-7.48574	-0.79885	0.35309
H	-6.70860	0.69117	-0.99322
C	-6.07752	-2.72195	0.72042
H	-4.21673	-2.76198	-0.34788
C	-7.26051	-2.02712	0.97976
H	-8.40539	-0.24998	0.54281
H	-5.89558	-3.68300	1.19610
H	-8.00100	-2.44025	1.66016
H	-4.66989	0.52912	-2.22191
H	-2.65397	-0.71781	-3.04232

Zero-point correction = 0.776562 (Hartree/Particle)
Thermal correction to Energy = 0.820731
Thermal correction to Enthalpy = 0.821676
Thermal correction to Gibbs Free Energy = 0.698602
Sum of electronic and zero-point Energies = -2214.814056
Sum of electronic and thermal Energies = -2214.769886
Sum of electronic and thermal Enthalpies = -2214.768942
Sum of electronic and thermal Free Energies = -2214.892016
E(CPCM(dioxane) M06L/def2-TZVP) = -3555.37093364
E(CPCM(dioxane) MN15L/def2-TZVP) = -3554.10508132

TS1

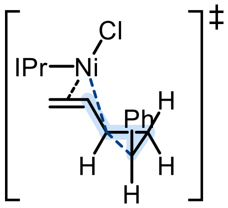

Ni	-0.37973	-0.20000	-0.26179
C	-4.41147	-0.38995	-1.72223
C	-2.53674	0.05665	-1.01725
H	-2.37466	1.08861	-1.33617
C	-2.32558	-0.25104	0.34990
C	-1.62253	0.62065	1.18569
H	-1.43139	0.33953	2.21828
H	-1.56832	1.68624	0.95443
H	-2.58504	-1.25176	0.69984
C	-3.05861	-0.90647	-2.02786
C	1.38255	0.38929	0.33189
Cl	0.20596	-1.44967	-2.03939
N	1.78003	1.69722	0.35275
N	2.52316	-0.29940	0.60757
C	3.13817	1.81834	0.62839
C	3.60151	0.55928	0.80386
H	3.63667	2.77646	0.66299
H	4.58590	0.18663	1.04475
C	0.85550	2.76029	0.06785
C	0.33995	2.84654	-1.24012
C	0.44844	3.61172	1.11054
C	-0.69069	3.76785	-1.46175
C	-0.57101	4.52877	0.82976
C	-1.15344	4.58766	-0.43460
H	-1.12715	3.85123	-2.45375
H	-0.92150	5.19887	1.60991
H	-1.95734	5.29462	-0.62615
C	2.66449	-1.73216	0.56948
C	2.07337	-2.51377	1.57364
C	3.41450	-2.29260	-0.48180
C	2.28964	-3.89589	1.53301

C	3.61842	-3.67499	-0.46388
C	3.06696	-4.47292	0.53490
H	1.83946	-4.52464	2.29948
H	4.20441	-4.13394	-1.25749
H	3.23148	-5.54782	0.52543
C	1.05707	3.51331	2.50431
H	2.13153	3.31906	2.39058
C	0.45865	2.33592	3.28513
H	-0.62349	2.47723	3.40730
H	0.61924	1.38561	2.76367
H	0.91172	2.26927	4.28197
C	0.91209	4.80162	3.31807
H	1.50167	4.72440	4.23858
H	1.25603	5.68002	2.75968
H	-0.13103	4.97018	3.61458
C	0.95220	2.05565	-2.38760
H	1.24128	1.06410	-2.02212
C	2.22069	2.77806	-2.86764
H	1.97649	3.78185	-3.24047
H	2.95206	2.88468	-2.05618
H	2.69325	2.21383	-3.68081
C	-0.00449	1.81555	-3.55481
H	0.46255	1.12541	-4.26535
H	-0.93480	1.34557	-3.21296
H	-0.24391	2.74463	-4.09044
C	1.18919	-1.93742	2.66604
H	1.14821	-0.84856	2.54528
C	-0.23890	-2.47507	2.50584
H	-0.92235	-1.98347	3.21119
H	-0.59748	-2.30370	1.48255
H	-0.27139	-3.55542	2.69985
C	1.73633	-2.24240	4.06498
H	1.76862	-3.32411	4.24791
H	2.75122	-1.84899	4.19317
H	1.09241	-1.79467	4.83197
C	3.99510	-1.46574	-1.62078
H	3.53573	-0.46771	-1.59187
C	5.51671	-1.32528	-1.46959
H	5.98891	-2.31614	-1.50733
H	5.93092	-0.72326	-2.28760
H	5.80513	-0.85849	-0.52037
C	3.67098	-2.07267	-2.99173
H	4.18785	-3.03047	-3.13610
H	2.59408	-2.22637	-3.10141
H	4.01449	-1.39464	-3.78316
H	-2.86233	-1.94836	-1.76431
C	-5.35443	-0.96951	-0.80378
C	-6.56031	-0.28356	-0.52697
C	-5.14666	-2.21468	-0.16532
C	-7.50825	-0.81200	0.33881
H	-6.73725	0.67598	-1.01060
C	-6.09684	-2.73677	0.70504
H	-4.23901	-2.77964	-0.36550
C	-7.28193	-2.04244	0.96462
H	-8.42783	-0.26411	0.53111
H	-5.91499	-3.69725	1.18179
H	-8.02203	-2.45638	1.64463
H	-4.71381	0.52920	-2.22026
H	-2.68906	-0.71246	-3.03733

Zero-point correction = 0.773256 (Hartree/Particle)
Thermal correction to Energy = 0.817565
Thermal correction to Enthalpy = 0.818509
Thermal correction to Gibbs Free Energy = 0.693727
Sum of electronic and zero-point Energies = -2214.774926
Sum of electronic and thermal Energies = -2214.730618
Sum of electronic and thermal Enthalpies = -2214.729674
Sum of electronic and thermal Free Energies = -2214.854455
E(CPCM(PhCl) MN15/def2-TZVP) = -3553.73296255
E(CPCM(PhCl) M06L/def2-TZVP) = -3555.35293555
E(CPCM(dioxane) M06L/def2-TZVP) = -3555.34769708
E(CPCM(dioxane) MN15L/def2-TZVP) = -3554.08223648
E(CPCM(dioxane) M06/def2-TZVP) = -3554.29956809
E(CPCM(dioxane) B3LYP-D3/def2-TZVP) = -3555.88969452
E(CPCM(dioxane) PBE0-D3/def2-TZVP) = -3553.40646427
E(CPCM(dioxane) TPSS-D3/def2-TZVP) = -3556.13588858
E(CPCM(dioxane) TPSSh-D3/def2-TZVP) = -3555.92048841
E(CPCM(dioxane) B97-D3/def2-TZVP) = -3555.32496092
E(CPCM(dioxane) wB97XD/def2-TZVP) = -3555.14661655

Int2

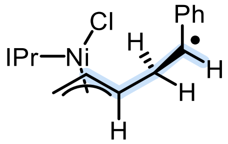

Ni	-0.34846	-0.26365	-0.07686
C	-4.51088	-1.08276	-1.55819
C	-2.40367	-0.34437	-0.50531
H	-2.36060	0.62867	-1.00877
C	-2.13987	-0.37800	0.85916
C	-1.30499	0.62095	1.42378
H	-0.95175	0.48968	2.44464
H	-1.40747	1.65755	1.09351
H	-2.30387	-1.31349	1.39838
C	-3.08898	-1.45534	-1.26382
C	1.42784	0.41849	0.21020
Cl	0.17795	-1.50901	-1.84983
N	1.77537	1.73448	0.08152
N	2.61738	-0.21834	0.38586
C	3.15224	1.91039	0.16469
C	3.67911	0.68239	0.37324
H	3.61570	2.88059	0.05835
H	4.69846	0.35521	0.51251
C	0.78868	2.75819	-0.13545
C	0.09925	2.75244	-1.36277
C	0.50183	3.66496	0.90121
C	-0.99459	3.61703	-1.48724
C	-0.58837	4.52293	0.71652
C	-1.34888	4.47693	-0.45034
H	-1.56664	3.62387	-2.41135
H	-0.85333	5.23074	1.49694
H	-2.20588	5.13686	-0.56232
C	2.82033	-1.64308	0.46776
C	2.40313	-2.33682	1.61402
C	3.47369	-2.28076	-0.60461
C	2.69073	-3.70465	1.68816
C	3.75469	-3.64317	-0.47109
C	3.37249	-4.35243	0.66433
H	2.37486	-4.26444	2.56694
H	4.26842	-4.15867	-1.27981
H	3.59560	-5.41372	0.74408
C	1.32203	3.70133	2.18616
H	2.37638	3.55902	1.91498
C	0.93582	2.55902	3.13401
H	-0.12322	2.64174	3.41156
H	1.08967	1.57992	2.66731
H	1.53650	2.60575	4.05072
C	1.22258	5.03872	2.92448
H	1.95184	5.06271	3.74207
H	1.41871	5.88892	2.26099
H	0.22948	5.17399	3.37153
C	0.60041	1.93849	-2.54621
H	1.01660	0.99432	-2.17955
C	1.72668	2.72491	-3.23568
H	1.35222	3.68708	-3.61003
H	2.55398	2.92846	-2.54345
H	2.12341	2.15555	-4.08486
C	-0.48841	1.56750	-3.55233
H	-0.07276	0.87596	-4.29295
H	-1.32409	1.05335	-3.06275
H	-0.87070	2.44711	-4.08825
C	1.63430	-1.68561	2.75036
H	1.50244	-0.62243	2.51973
C	0.23783	-2.31285	2.84881
H	-0.38522	-1.76818	3.57101
H	-0.25501	-2.29711	1.86849
H	0.30219	-3.35831	3.17823
C	2.38232	-1.79577	4.08353
H	2.51674	-2.84574	4.37343
H	3.37407	-1.33282	4.02589
H	1.81522	-1.29996	4.88101
C	3.87691	-1.55923	-1.88317

H	3.36745	-0.58610	-1.90472
C	5.39627	-1.34001	-1.92891
H	5.91397	-2.30854	-1.91874
H	5.68115	-0.81567	-2.84922
H	5.76677	-0.75960	-1.07596
C	3.43888	-2.32803	-3.13641
H	3.99760	-3.26702	-3.24367
H	2.36908	-2.54971	-3.10214
H	3.64455	-1.72304	-4.02816
H	-3.01963	-2.38371	-0.67924
C	-5.60175	-1.24240	-0.67113
C	-6.89842	-0.79774	-1.05671
C	-5.47629	-1.84111	0.61394
C	-7.99052	-0.94102	-0.21709
H	-7.01706	-0.33775	-2.03657
C	-6.57602	-1.97927	1.44830
H	-4.50387	-2.19794	0.94402
C	-7.84020	-1.53256	1.04476
H	-8.96891	-0.59224	-0.53996
H	-6.45245	-2.44058	2.42569
H	-8.69721	-1.64446	1.70375
H	-4.71291	-0.58930	-2.50737
H	-2.53523	-1.63034	-2.19388

Zero-point correction = 0.774631 (Hartree/Particle)
Thermal correction to Energy = 0.819035
Thermal correction to Enthalpy = 0.819979
Thermal correction to Gibbs Free Energy = 0.694756
Sum of electronic and zero-point Energies = -2214.784431
Sum of electronic and thermal Energies = -2214.740028
Sum of electronic and thermal Enthalpies = -2214.739084
Sum of electronic and thermal Free Energies = -2214.864307
E(CPCM(PhCl) MN15/def2-TZVP) = -3553.74765028
E(CPCM(PhCl) M06L/def2-TZVP) = -3555.36666021
E(CPCM(dioxane) M06L/def2-TZVP) = -3555.36159945
E(CPCM(dioxane) MN15L/def2-TZVP) = -3554.09478160
E(CPCM(dioxane) M06/def2-TZVP) = -3554.31425453
E(CPCM(dioxane) B3LYP-D3/def2-TZVP) = -3555.90364536
E(CPCM(dioxane) PBE0-D3/def2-TZVP) = -3553.41976696
E(CPCM(dioxane) TPSS-D3/def2-TZVP) = -3556.14924200
E(CPCM(dioxane) TPSSh-D3/def2-TZVP) = -3555.93481322
E(CPCM(dioxane) B97-D3/def2-TZVP) = -3555.33708965
E(CPCM(dioxane) wB97XD/def2-TZVP) = -3555.16302225

η¹-Int2

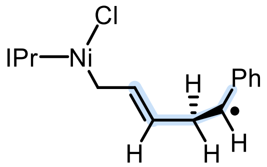

Ni	-0.94908	-0.63473	-0.96923
C	-1.32285	0.36819	0.55343
Cl	-1.29894	-1.73408	-2.81788
N	-2.53842	0.23104	1.14884
N	-0.68565	1.30231	1.31308
C	-2.66474	1.05928	2.25492
C	-1.49708	1.73980	2.35688
H	-3.56754	1.08790	2.84755
H	-1.16357	2.49357	3.05529
C	-3.58884	-0.56189	0.56249
C	-4.48746	0.08446	-0.30397
C	-3.63854	-1.93711	0.82932
C	-5.49888	-0.69078	-0.87673
C	-4.67695	-2.66755	0.24228
C	-5.60130	-2.05177	-0.59627
H	-6.20268	-0.22776	-1.56510
H	-4.74694	-3.73686	0.43208
H	-6.39428	-2.63956	-1.05205
C	0.61965	1.83236	1.03309
C	1.67830	1.50455	1.89949
C	0.79629	2.63291	-0.10966
C	2.93715	2.04879	1.62345
C	2.08389	3.12352	-0.35962
C	3.14299	2.84869	0.50129
H	3.77373	1.81739	2.28082
H	2.25337	3.73308	-1.24521
H	4.13371	3.24446	0.29094
C	-2.59600	-2.64230	1.67773
H	-1.79793	-1.92392	1.90850
C	-1.96489	-3.80181	0.89486
H	-2.68024	-4.62501	0.76868
H	-1.64807	-3.47337	-0.10274
H	-1.09530	-4.19648	1.43516
C	-3.20069	-3.13126	2.99908
H	-2.44336	-3.64540	3.60317
H	-3.60694	-2.29945	3.58742
H	-4.01742	-3.83972	2.80805
C	-4.30877	1.54040	-0.70379
H	-3.61733	2.01668	0.00539
C	-5.62038	2.33120	-0.66978
H	-6.32925	1.95947	-1.41983
H	-6.10320	2.27109	0.31238
H	-5.42899	3.38648	-0.89749
C	-3.67163	1.60413	-2.10012
H	-3.44065	2.64328	-2.37410
H	-2.74667	1.01180	-2.15161
H	-4.35844	1.19116	-2.85040
C	1.51885	0.54347	3.06665
H	0.54494	0.04412	2.96841
C	2.60842	-0.53951	3.04274
H	2.37088	-1.33094	3.76315
H	2.71388	-0.99035	2.04792
H	3.58410	-0.12333	3.32555
C	1.54695	1.29127	4.40580
H	2.50084	1.82247	4.52185
H	0.74127	2.03161	4.47782
H	1.44525	0.58983	5.24241
C	-0.31637	2.95238	-1.09473
H	-1.25612	2.53259	-0.71368
C	-0.51456	4.46520	-1.25107
H	0.37181	4.93901	-1.69192
H	-1.36161	4.66169	-1.92012
H	-0.71453	4.94923	-0.28818
C	-0.02360	2.30106	-2.45429
H	0.90804	2.69956	-2.87980
H	0.08394	1.21042	-2.36893
H	-0.83644	2.50955	-3.16080
C	0.59599	-1.41233	-0.15393
C	1.68786	-0.68827	-0.81841
H	0.59562	-1.33310	0.93816
C	2.21717	-1.00378	-2.01532
H	2.07015	0.20144	-0.31749
H	1.84015	-1.86811	-2.56381
C	3.34771	-0.21639	-2.63418
C	4.62622	-1.00073	-2.56158
H	3.44100	0.74404	-2.10292
H	3.11444	0.01348	-3.68206
C	5.44354	-1.10361	-1.41012
H	4.90785	-1.59708	-3.42710
C	5.16497	-0.40905	-0.19889
C	6.60530	-1.92587	-1.43380
C	5.99711	-0.53468	0.90385
H	4.28805	0.22829	-0.13452
C	7.43062	-2.04298	-0.32695
H	6.83714	-2.46855	-2.34895
C	7.13510	-1.34921	0.85452
H	5.75764	0.00812	1.81743
H	8.31173	-2.67883	-0.37651
H	7.78210	-1.44349	1.72277
H	0.46735	-2.44673	-0.48865

Zero-point correction = 0.773373 (Hartree/Particle)
Thermal correction to Energy = 0.818461
Thermal correction to Enthalpy = 0.819405
Thermal correction to Gibbs Free Energy = 0.692987
Sum of electronic and zero-point Energies = -2214.760928
Sum of electronic and thermal Energies = -2214.715840
Sum of electronic and thermal Enthalpies = -2214.714896
Sum of electronic and thermal Free Energies = -2214.841314
E(CPCM(dioxane) M06L/def2-TZVP) = -3555.33648218
E(CPCM(dioxane) MN15L/def2-TZVP) = -3554.07131581

η1-Int2'

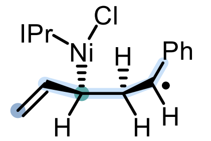

Note: structure was obtained through scans and is not fully optimized

C	-3.67717700	-1.74915700	-1.82485600
C	-1.94336900	-0.38828100	-0.94460200
H	-2.25229400	0.27595500	-1.76496000
C	-2.31890000	-1.86025300	-1.24542300
H	-2.28747500	-2.46995200	-0.33600200
C	-4.89565100	-1.71798400	-1.10097400
C	-6.11429900	-1.47916900	-1.79492200
C	-4.97442600	-1.93421800	0.30211800
C	-7.32944500	-1.45480500	-1.13085200
H	-6.07389500	-1.31424600	-2.87042700
C	-6.19641000	-1.90781700	0.95957600
H	-4.06610700	-2.13786900	0.86338600
C	-7.38026300	-1.66650100	0.25361000
H	-8.24563700	-1.27011300	-1.68676300
H	-6.23185900	-2.07950200	2.03294500
H	-8.33355300	-1.64672200	0.77525600
H	-3.73673700	-1.57803300	-2.89885000
H	-1.62166500	-2.31864900	-1.96163400
C	-2.44979200	0.11677300	0.33645100
H	-2.18061800	-0.45728000	1.22700200
C	-3.21383800	1.22592900	0.45872200
H	-3.48748400	1.80893700	-0.41771000
H	-3.57977200	1.56118500	1.42656700
Ni	0.05130800	-0.31496000	-0.96356900
C	1.24781300	0.39058800	0.42008600
Cl	0.99500000	-1.02637400	-2.82108300
N	1.35711200	1.69458000	0.80896100
N	2.33747100	-0.20275900	0.97675200
C	2.50531400	1.91204300	1.56340400
C	0.41136100	2.69372300	0.38907500
C	3.11996700	0.70984100	1.67884700
C	2.74235100	-1.56337700	0.76258300
H	2.77592200	2.89393800	1.92532600
C	0.35851500	3.02398000	-0.97799300
C	-0.44575500	3.26020000	1.35017300
H	4.02579000	0.40962900	2.18449000
C	2.08033200	-2.57843600	1.45801900
C	3.80486700	-1.81310900	-0.12530000
C	-0.66222700	3.89129300	-1.38479600
C	1.43196600	2.56028100	-1.95180000
C	-1.43667800	4.13415300	0.89194000
C	-0.31349900	2.91282000	2.82768800
C	2.53560400	-3.89045100	1.28204200
C	0.89388200	-2.29477800	2.36280900
C	4.23463100	-3.13714700	-0.25642200
C	4.47072800	-0.71407300	-0.94260900
C	-1.55927300	4.42597300	-0.46448500
H	-0.74960900	4.15573600	-2.43528000
H	1.74298200	1.54395400	-1.68032400
C	2.65365700	3.48413100	-1.82750300
C	0.95199500	2.49325600	-3.40119000
H	-2.12546600	4.58863700	1.59881000
H	0.75625200	2.82518500	3.05489400
C	-0.96250700	1.56425600	3.14996900
C	-0.88696400	3.99532000	3.74933600
C	3.61143400	-4.16894100	0.44394900
H	2.03807800	-4.70004300	1.81311200
H	0.70697100	-1.21210100	2.35862300
C	-0.35496100	-2.98053800	1.79737700
C	1.15865800	-2.72870300	3.80908200
H	5.05939400	-3.36269600	-0.92923400
H	3.84813200	0.18902400	-0.87913900
C	5.87061800	-0.39780500	-0.39868700
C	4.55910500	-1.08952700	-2.42793000
H	-2.34901300	5.09229800	-0.80366100
H	2.38046600	4.51659400	-2.08278100
H	3.05818600	3.48141900	-0.80727300
H	3.44811100	3.15879700	-2.51078200
H	1.72611800	2.02349400	-4.01783200
H	0.04791000	1.88009200	-3.48871800
H	0.75377200	3.49227400	-3.81292800
H	-2.03218200	1.60318600	2.91244000
H	-0.51417000	0.74894700	2.56818300
H	-0.85020300	1.34024000	4.21826500
H	-0.62181300	3.77462800	4.78981800
H	-0.50751600	4.99351600	3.50111900
H	-1.98234900	4.02124100	3.69284500
H	3.95681000	-5.19303800	0.32448800
H	-1.25490800	-2.67999500	2.35264200
H	-0.48002200	-2.71585300	0.74086800
H	-0.26834400	-4.07295300	1.86264500
H	1.32775400	-3.81140800	3.86947200
H	2.04002000	-2.22649100	4.22384900
H	0.29528100	-2.49025500	4.44210800
H	6.50810200	-1.28967400	-0.46651800
H	6.34066200	0.39853600	-0.98836300
H	5.85261300	-0.08178600	0.65069100
H	5.25898700	-1.91932300	-2.59071000
H	3.57565800	-1.37049300	-2.81904100
H	4.93079600	-0.23142500	-3.00162900

Zero-point correction = 0.772257 (Hartree/Particle)
Thermal correction to Energy = 0.816085
Thermal correction to Enthalpy = 0.817029
Thermal correction to Gibbs Free Energy = 0.692317
Sum of electronic and zero-point Energies = -2214.741548
Sum of electronic and thermal Energies = -2214.697719
Sum of electronic and thermal Enthalpies = -2214.696775
Sum of electronic and thermal Free Energies = -2214.821487
E(CPCM(dioxane) M06L/def2-TZVP) = -3555.30643692

TS2

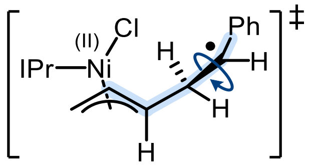

Ni	-0.33015	0.09504	0.17261
C	-4.75991	0.58848	-0.36242
C	-2.32795	0.70629	0.08745
H	-2.06539	1.59298	-0.50288
C	-1.85131	0.63325	1.39139
C	-0.64624	1.30497	1.72067
H	-0.17699	1.09689	2.68011
H	-0.45312	2.29916	1.31013
H	-2.22118	-0.16788	2.03442
C	-3.45503	-0.14160	-0.43921
C	1.59388	0.12909	0.10884
Cl	-0.58382	-1.31662	-1.53235
N	2.33035	1.23951	-0.19750
N	2.51410	-0.87206	0.15310
C	3.67809	0.93302	-0.35053
C	3.79424	-0.39324	-0.11267
H	4.41091	1.67963	-0.62058
H	4.65376	-1.04645	-0.10608
C	1.71809	2.53181	-0.35193
C	0.85467	2.71914	-1.44790
C	1.94283	3.51865	0.62520
C	0.10712	3.90232	-1.47958
C	1.18550	4.69215	0.53439
C	0.25400	4.86853	-0.48709
H	-0.58770	4.07272	-2.29784
H	1.31793	5.47594	1.27493
H	-0.33941	5.77902	-0.52491
C	2.24275	-2.27605	0.33547
C	1.83445	-2.74451	1.59366
C	2.44055	-3.13554	-0.76250
C	1.66000	-4.12453	1.74953
C	2.27270	-4.50622	-0.54770
C	1.89010	-5.00173	0.69585
H	1.34121	-4.51122	2.71609
H	2.43006	-5.19425	-1.37568
H	1.75905	-6.07174	0.83867

C	2.94841	3.31705	1.75356
H	3.82876	2.81427	1.33210
C	2.38653	2.40861	2.85428
H	1.48438	2.85823	3.28948
H	2.11959	1.42068	2.46333
H	3.12589	2.27808	3.65404
C	3.43222	4.63251	2.36927
H	4.26417	4.43389	3.05429
H	3.77426	5.34115	1.60615
H	2.63834	5.11424	2.95415
C	0.83291	1.74049	-2.61233
H	0.96990	0.72446	-2.22735
C	2.01252	2.06500	-3.54278
H	1.91912	3.08316	-3.94367
H	2.97117	1.99525	-3.01252
H	2.03597	1.36370	-4.38555
C	-0.48011	1.73220	-3.39413
H	-0.45966	0.91448	-4.12241
H	-1.33656	1.55456	-2.73281
H	-0.63777	2.67025	-3.94388
C	1.54379	-1.83007	2.77069
H	1.73546	-0.79517	2.46540
C	0.05940	-1.93443	3.14340
H	-0.20838	-1.18327	3.89862
H	-0.56626	-1.78569	2.25424
H	-0.17012	-2.92570	3.55611
C	2.43761	-2.14555	3.97508
H	2.26391	-3.16697	4.33712
H	3.49977	-2.05464	3.72061
H	2.22005	-1.45788	4.80161
C	2.81983	-2.64048	-2.15135
H	2.66620	-1.55308	-2.18339
C	4.29071	-2.95642	-2.45997
H	4.45177	-4.04261	-2.43975
H	4.55948	-2.59480	-3.45998
H	4.98153	-2.50740	-1.73690
C	1.93117	-3.25631	-3.23972
H	2.12046	-4.33241	-3.34708
H	0.87354	-3.09989	-3.01245
H	2.15651	-2.78802	-4.20583
H	-3.48371	-1.08325	0.12502
C	-6.02377	0.01467	-0.09417
C	-7.17621	0.85013	-0.02668
C	-6.22108	-1.38020	0.11249
C	-8.43217	0.32944	0.23563
H	-7.04885	1.92005	-0.18474
C	-7.48405	-1.89131	0.37345
H	-5.37198	-2.05641	0.05365
C	-8.59945	-1.04727	0.43993
H	-9.29228	0.99377	0.28252
H	-7.60662	-2.96158	0.52479
H	-9.58542	-1.45601	0.64501
H	-4.73252	1.66226	-0.54776
H	-3.21180	-0.42712	-1.47626

Zero-point correction = 0.774311 (Hartree/Particle)
Thermal correction to Energy = 0.817880
Thermal correction to Enthalpy = 0.818824
Thermal correction to Gibbs Free Energy = 0.696719
Sum of electronic and zero-point Energies = -2214.783352
Sum of electronic and thermal Energies = -2214.739783
Sum of electronic and thermal Enthalpies = -2214.738839
Sum of electronic and thermal Free Energies = -2214.860944
Zero-point correction = 0.774311 (Hartree/Particle)
Thermal correction to Energy = 0.817880
Thermal correction to Enthalpy = 0.818824
Thermal correction to Gibbs Free Energy = 0.696719
Sum of electronic and zero-point Energies = -2214.783352
Sum of electronic and thermal Energies = -2214.739783
Sum of electronic and thermal Enthalpies = -2214.738839
Sum of electronic and thermal Free Energies = -2214.860944
E(CPCM(PhCl) MN15/def2-TZVP) = -3553.74641108
E(CPCM(PhCl) M06L/def2-TZVP) = -3555.36552660
E(CPCM(dioxane) M06L/def2-TZVP) = -3555.36068215
E(CPCM(dioxane) MN15L/def2-TZVP) = -3554.09258168
E(CPCM(dioxane) M06/def2-TZVP) = -3554.31313857
E(CPCM(dioxane) B3LYP-D3/def2-TZVP) = -3555.90255217
E(CPCM(dioxane) PBE0-D3/def2-TZVP) = -3553.41903915
E(CPCM(dioxane) TPSS-D3/def2-TZVP) = -3556.14794754
E(CPCM(dioxane) TPSSh-D3/def2-TZVP) = -3555.93369688
E(CPCM(dioxane) B97-D3/def2-TZVP) = -3555.33552044
E(CPCM(dioxane) wB97XD/def2-TZVP) = -3555.16196818

Int3

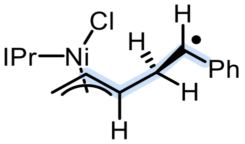

Ni	-0.03176	-0.55137	-0.68431
C	3.78617	-2.40368	-1.68084
C	1.96684	-1.29026	-1.14043
H	2.42536	-0.61909	-0.40708
C	1.30077	-0.71268	-2.21734
C	0.85172	0.62066	-2.08775
H	0.22683	1.05408	-2.86602
H	1.45333	1.32863	-1.51436
H	0.92762	-1.35632	-3.01679
C	2.44502	-2.70705	-1.10294
C	-1.28211	0.69478	0.12576
Cl	-0.58525	-2.43887	0.40736
N	-0.88667	1.77750	0.86385
N	-2.63141	0.66658	0.29308
C	-1.96572	2.40012	1.48287
C	-3.06671	1.70766	1.10958
H	-1.84393	3.25933	2.12677
H	-4.11206	1.84460	1.34323
C	0.50442	2.12314	0.97659
C	1.34479	1.22044	1.65686
C	0.97989	3.27709	0.32964
C	2.72485	1.44133	1.56866
C	2.36626	3.46773	0.29748
C	3.23040	2.54119	0.87827
H	3.41366	0.74255	2.03760
H	2.77759	4.34057	-0.20219
H	4.30565	2.68853	0.81200
C	-3.50342	-0.38078	-0.17131
C	-3.77405	-0.50107	-1.54266
C	-4.05979	-1.24639	0.78904
C	-4.67438	-1.49575	-1.94089
C	-4.96834	-2.20890	0.34105
C	-5.28003	-2.33259	-1.01011
H	-4.90072	-1.61125	-2.99956
H	-5.42355	-2.88381	1.06296
H	-5.98387	-3.09360	-1.33870
C	0.03163	4.25583	-0.35255
H	-0.86747	4.34368	0.27151
C	-0.41250	3.73300	-1.72513
H	0.45886	3.61681	-2.38318
H	-0.90725	2.75850	-1.64391
H	-1.10683	4.44007	-2.19562
C	0.62130	5.66080	-0.49968
H	-0.15514	6.35249	-0.84557
H	1.02335	6.03752	0.44798
H	1.42654	5.67842	-1.24508
C	0.76907	0.12501	2.54421
H	-0.13610	-0.27664	2.07454
C	0.37738	0.74509	3.89485
H	1.25940	1.16343	4.39782
H	-0.35550	1.55219	3.76699
H	-0.06362	-0.01674	4.54911
C	1.70311	-1.06591	2.74760
H	1.17873	-1.84311	3.31398
H	1.98259	-1.50530	1.78326
H	2.61036	-0.79307	3.30469
C	-3.11435	0.36364	-2.60331
H	-2.45307	1.08437	-2.10776
C	-2.24373	-0.51367	-3.51268
H	-1.68108	0.10405	-4.22543
H	-1.53649	-1.10246	-2.91362
H	-2.86429	-1.21310	-4.08820
C	-4.14635	1.14693	-3.42264
H	-4.81542	0.46741	-3.96595

H -4.76426 1.78705 -2.78259
H -3.64338 1.78090 -4.16321
C -3.70846 -1.18213 2.26899
H -2.82736 -0.53635 2.38773
C -4.87373 -0.59589 3.07883
H -5.75929 -1.23764 2.97851
H -4.61496 -0.54270 4.14338
H -5.15532 0.40941 2.74347
C -3.33502 -2.56092 2.82781
H -4.20055 -3.23621 2.83532
H -2.53355 -3.01281 2.23693
H -2.99429 -2.45598 3.86563
H 1.83212 -3.35700 -1.73460
C 4.89087 -1.91233 -0.93915
C 4.89857 -1.83305 0.48046
C 6.05894 -1.48802 -1.62566
C 6.01684 -1.37056 1.16042
H 4.02334 -2.14851 1.04307
C 7.17260 -1.03505 -0.93771
H 6.06699 -1.53529 -2.71338
C 7.16278 -0.97161 0.46186
H 6.00111 -1.32628 2.24758
H 8.05772 -0.72589 -1.48897
H 8.03741 -0.61645 1.00045
H 3.85663 -2.37641 -2.76657
H 2.45459 -3.11477 -0.08719
Zero-point correction = 0.774942 (Hartree/Particle)
Thermal correction to Energy = 0.819194
Thermal correction to Enthalpy = 0.820138
Thermal correction to Gibbs Free Energy = 0.696384
Sum of electronic and zero-point Energies = -2214.788560
Sum of electronic and thermal Energies = -2214.744308
Sum of electronic and thermal Enthalpies = -2214.743364
Sum of electronic and thermal Free Energies = -2214.867118
E(CPCM(PhCl) MN15/def2-TZVP) = -3553.75071575
E(CPCM(PhCl) M06L/def2-TZVP) = -3555.36906789
E(CPCM(dioxane) M06L/def2-TZVP) = -3555.36448863
E(CPCM(dioxane) MN15L/def2-TZVP) = -3554.09802036
E(CPCM(dioxane) M06/def2-TZVP) = -3554.31749795
E(CPCM(dioxane) B3LYP-D3/def2-TZVP) = -3555.90756733
E(CPCM(dioxane) PBE0-D3/def2-TZVP) = -3553.42364373
E(CPCM(dioxane) TPSS-D3/def2-TZVP) = -3556.15230469
E(CPCM(dioxane) TPSSh-D3/def2-TZVP) = -3555.93814503
E(CPCM(dioxane) B97-D3/def2-TZVP) = -3555.34067274
E(CPCM(dioxane) wB97XD/def2-TZVP) = -3555.16672781

TS3

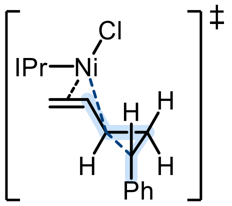

Ni -0.02664 -0.58293 -0.68753
C 3.75209 -2.37417 -1.68427
C 2.07750 -1.32641 -1.17774
H 2.50206 -0.66301 -0.42112
C 1.31907 -0.73802 -2.21798
C 0.89560 0.59321 -2.11639
H 0.25778 1.02060 -2.88649
H 1.46646 1.30066 -1.51313
H 0.92855 -1.38582 -3.00579
C 2.44909 -2.76417 -1.09401
C -1.26969 0.67537 0.12047
Cl -0.57292 -2.47011 0.40238
N -0.87461 1.75531 0.86048
N -2.61895 0.64319 0.28812
C -1.95413 2.37639 1.47971
C -3.05456 1.68299 1.10570
H -1.83413 3.23521 2.12447
H -4.09979 1.81976 1.33999
C 0.51736 2.09783 0.97120
C 1.35805 1.19419 1.65053
C 0.99370 3.25111 0.32381
C 2.73824 1.41501 1.56228
C 2.38016 3.44152 0.29135
C 3.24412 2.51503 0.87230
H 3.42686 0.71646 2.03192
H 2.79157 4.31420 -0.20856
H 4.31940 2.66241 0.80635
C -3.48932 -0.40590 -0.17691
C -3.76012 -0.52709 -1.54834
C -4.04581 -1.27224 0.78299
C -4.66039 -1.52188 -1.94670
C -4.95434 -2.23493 0.33502
C -5.26621 -2.35875 -1.01605
H -4.88662 -1.63732 -3.00543
H -5.40939 -2.90983 1.05707
H -5.97010 -3.11973 -1.34464
C 0.04535 4.22963 -0.35853
H -0.85381 4.31719 0.26543
C -0.39844 3.70640 -1.73117
H 0.47305 3.59117 -2.38929
H -0.89230 2.73129 -1.65011
H -1.09340 4.41282 -2.20169
C 0.63496 5.63459 -0.50574
H -0.14144 6.32633 -0.85166
H 1.03693 6.01130 0.44197
H 1.44028 5.65226 -1.25106
C 0.78259 0.09888 2.53825
H -0.12264 -0.30260 2.06847
C 0.39112 0.71890 3.88893
H 1.27316 1.13724 4.39193
H -0.34184 1.52590 3.76108
H -0.04986 -0.04299 4.54314
C 1.71661 -1.09218 2.74118
H 1.19231 -1.86953 3.30742
H 1.99485 -1.53078 1.77594
H 2.62423 -0.81966 3.29795
C -3.10059 0.33743 -2.60925
H -2.43921 1.05800 -2.11357
C -2.23028 -0.53987 -3.51895
H -1.66725 0.07788 -4.23146
H -1.52358 -1.12928 -2.92000
H -2.85112 -1.23868 -4.09496
C -4.13259 1.12075 -3.42857
H -4.80167 0.44115 -3.97180
H -4.75048 1.76084 -2.78849
H -3.62972 1.75472 -4.16924
C -3.69470 -1.20832 2.26302
H -2.81367 -0.56246 2.38173
C -4.86002 -0.62217 3.07286
H -5.74556 -1.26397 2.97247
H -4.60130 -0.56907 4.13744
H -5.14162 0.38313 2.73757
C -3.32129 -2.58718 2.82177
H -4.18698 -3.26231 2.82948
H -2.52000 -3.03922 2.23070
H -2.98051 -2.48219 3.85959
H 1.82689 -3.39885 -1.72934
C 4.89766 -1.92574 -0.94146
C 4.91170 -1.85951 0.47292
C 6.06352 -1.51501 -1.62986
C 6.03224 -1.39865 1.15392
H 4.03508 -2.16949 1.03698
C 7.18335 -1.06500 -0.94300
H 6.07041 -1.55814 -2.71787
C 7.17570 -0.99898 0.45449
H 6.01684 -1.35612 2.24107
H 8.06787 -0.75928 -1.49708
H 8.05083 -0.64253 0.99169
H 3.82594 -2.37003 -2.76954
H 2.46350 -3.16150 -0.07598
Zero-point correction = 0.773390 (Hartree/Particle)
Thermal correction to Energy = 0.817551
Thermal correction to Enthalpy = 0.818495
Thermal correction to Gibbs Free Energy = 0.695127
Sum of electronic and zero-point Energies = -2214.777176
Sum of electronic and thermal Energies = -2214.733015
Sum of electronic and thermal Enthalpies = -2214.732071
Sum of electronic and thermal Free Energies = -2214.855439

E(CPCM(PhCl) MN15/def2-TZVP) = -3553.73554266
E(CPCM(PhCl) M06L/def2-TZVP) = -3555.35527946
E(CPCM(dioxane) M06L/def2-TZVP) = -3555.35004622
E(CPCM(dioxane) MN15L/def2-TZVP) = -3554.08627105
E(CPCM(dioxane) M06/def2-TZVP) = -3554.30117719
E(CPCM(dioxane) B3LYP-D3/def2-TZVP) = -3555.89139313
E(CPCM(dioxane) PBE0-D3/def2-TZVP) = -3553.40840215
E(CPCM(dioxane) TPSS-D3/def2-TZVP) = -3556.13833641
E(CPCM(dioxane) TPSSh-D3/def2-TZVP) = -3555.92287547
E(CPCM(dioxane) B97-D3/def2-TZVP) = -3555.32733492
E(CPCM(dioxane) wB97XD/def2-TZVP) = -3555.14883956

Int4-*spl*

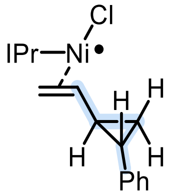

Ni	-0.04630	-0.56245	-0.67928
C	3.68335	-2.28947	-1.67507
C	2.16390	-1.31562	-1.20379
H	2.50318	-0.66186	-0.39792
C	1.30161	-0.70641	-2.20751
C	0.90193	0.61846	-2.12738
H	0.24790	1.04236	-2.88542
H	1.44365	1.32464	-1.49710
H	0.90593	-1.35403	-2.99345
C	2.42246	-2.76731	-1.07343
C	-1.28451	0.71139	0.12591
Cl	-0.58742	-2.44602	0.40853
N	-0.88950	1.78491	0.86857
N	-2.63299	0.67108	0.29463
C	-1.96896	2.40413	1.48754
C	-3.06896	1.70969	1.11282
H	-1.85049	3.26237	2.13328
H	-4.11408	1.84581	1.34786
C	0.50349	2.12479	0.97753
C	1.34413	1.22053	1.65639
C	0.98008	3.27763	0.32977
C	2.72444	1.44127	1.56814
C	2.36651	3.46779	0.29727
C	3.23038	2.54131	0.87836
H	3.41309	0.74238	2.03711
H	2.77800	4.34029	-0.20284
H	4.30563	2.68827	0.81219
C	-3.50275	-0.37917	-0.17092
C	-3.77374	-0.50074	-1.54232
C	-4.05938	-1.24579	0.78896
C	-4.67402	-1.49559	-1.94069
C	-4.96790	-2.20857	0.34099
C	-5.27987	-2.33246	-1.01006
H	-4.90027	-1.61098	-2.99939
H	-5.42295	-2.88337	1.06310
H	-5.98378	-3.09340	-1.33862
C	0.03163	4.25595	-0.35255
H	-0.86755	4.34351	0.27139
C	-0.41205	3.73258	-1.72522
H	0.45943	3.61743	-2.38339
H	-0.90637	2.75756	-1.64432
H	-1.10706	4.43884	-2.19589
C	0.62122	5.66089	-0.49982
H	-0.15515	6.35265	-0.84571
H	1.02314	6.03750	0.44792
H	1.42657	5.67854	-1.24508
C	0.76888	0.12521	2.54434
H	-0.13727	-0.27555	2.07563
C	0.37741	0.74521	3.89497
H	1.25952	1.16342	4.39783
H	-0.35551	1.55227	3.76721
H	-0.06349	-0.01673	4.54917
C	1.70282	-1.06603	2.74698
H	1.17866	-1.84318	3.31365
H	1.98171	-1.50465	1.78190
H	2.61042	-0.79339	3.30352
C	-3.11429	0.36374	-2.60327
H	-2.45320	1.08465	-2.10756
C	-2.24418	-0.51358	-3.51316
H	-1.68084	0.10397	-4.22557
H	-1.53777	-1.10402	-2.91512
H	-2.86517	-1.21201	-4.08937
C	-4.14629	1.14703	-3.42260
H	-4.81529	0.46735	-3.96577
H	-4.76416	1.78708	-2.78250
H	-3.64345	1.78097	-4.16328
C	-3.70840	-1.18202	2.26902
H	-2.82751	-0.53593	2.38795
C	-4.87374	-0.59594	3.07886
H	-5.75922	-1.23778	2.97848
H	-4.61497	-0.54284	4.14341
H	-5.15537	0.40932	2.74359
C	-3.33499	-2.56092	2.82775
H	-4.20069	-3.23597	2.83547
H	-2.53401	-3.01391	2.23700
H	-2.99421	-2.45596	3.86553
H	1.79501	-3.38886	-1.71395
C	4.88197	-1.89169	-0.93108
C	4.89836	-1.83340	0.47640
C	6.04355	-1.49123	-1.62173
C	6.02044	-1.37293	1.15961
H	4.02052	-2.14068	1.04097
C	7.16836	-1.04054	-0.93548
H	6.04987	-1.53039	-2.71012
C	7.16172	-0.97298	0.45992
H	6.00491	-1.33148	2.24691
H	8.05212	-0.73590	-1.49166
H	8.03710	-0.61553	0.99651
H	3.78238	-2.34746	-2.75733
H	2.44341	-3.16063	-0.05586

Zero-point correction = 0.775948 (Hartree/Particle)
Thermal correction to Energy = 0.820322
Thermal correction to Enthalpy = 0.821266
Thermal correction to Gibbs Free Energy = 0.697301
Sum of electronic and zero-point Energies = -2214.816747
Sum of electronic and thermal Energies = -2214.772373
Sum of electronic and thermal Enthalpies = -2214.771429
Sum of electronic and thermal Free Energies = -2214.895394
E(CPCM(dioxane) M06L/def2-TZVP) = -3555.37503774
E(CPCM(dioxane) MN15L/def2-TZVP) = -3554.11043273

Int4-*tet*

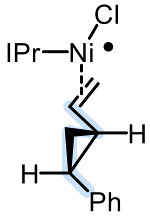

Ni	-0.33043	-1.02842	0.03187
C	3.83797	-0.43098	0.33145
C	2.52665	-1.19679	0.44758
H	2.57131	-2.27592	0.30672
C	1.50861	-0.72251	1.40497
C	0.63646	-1.51075	2.07743
H	-0.04595	-1.08473	2.81077
H	0.65185	-2.59436	1.96944
H	1.50361	0.35473	1.57582
C	-1.34266	0.64992	0.15388
Cl	-0.11549	-2.93479	-1.08946
N	-2.70138	0.56899	0.23033
N	-1.10811	1.98732	0.03537
C	-3.30279	1.81522	0.12750
C	-2.29590	2.71497	0.00238
H	-4.37589	1.94076	0.15250
H	-2.30488	3.78931	-0.11312
C	-3.35561	-0.71401	0.21918
C	-3.68546	-1.27323	-1.02568
C	-3.54852	-1.38608	1.43840
C	-4.29145	-2.53369	-1.02214

C	-4.14704	-2.64750	1.38819
C	-4.52255	-3.21170	0.17017
H	-4.56404	-2.99729	-1.96713
H	-4.31234	-3.20152	2.30860
H	-4.98266	-4.19665	0.15134
C	0.20225	2.53124	-0.20041
C	1.01524	2.86202	0.90054
C	0.62655	2.66897	-1.53209
C	2.30065	3.34192	0.62942
C	1.91703	3.16791	-1.75126
C	2.74619	3.49720	-0.68354
H	2.96371	3.59729	1.45188
H	2.28212	3.27588	-2.77000
H	3.75015	3.86946	-0.87203
C	-3.14671	-0.74717	2.75736
H	-2.26816	-0.11439	2.56406
C	-2.75517	-1.77494	3.82201
H	-3.63068	-2.32753	4.18632
H	-2.03240	-2.50068	3.43031
H	-2.30954	-1.26804	4.68640
C	-4.27745	0.15243	3.27642
H	-3.99470	0.62553	4.22495
H	-4.52344	0.94411	2.55857
H	-5.18303	-0.44399	3.44721
C	-3.41500	-0.54666	-2.33329
H	-2.66197	0.23130	-2.14018
C	-4.69766	0.13480	-2.82966
H	-5.47527	-0.61775	-3.01634
H	-5.08993	0.84766	-2.09341
H	-4.51307	0.67321	-3.76736
C	-2.84080	-1.47319	-3.40996
H	-2.56632	-0.88355	-4.29405
H	-1.95292	-1.99957	-3.04104
H	-3.58008	-2.21683	-3.73479
C	0.50139	2.72424	2.32717
H	-0.13720	1.82740	2.36467
C	1.61747	2.55781	3.36315
H	1.18241	2.30978	4.33771
H	2.32424	1.76389	3.09239
H	2.18348	3.49005	3.48729
C	-0.35673	3.94190	2.70339
H	0.24609	4.85693	2.64064
H	-1.22004	4.05468	2.03958
H	-0.72769	3.84657	3.73103
C	-0.23182	2.21189	-2.70083
H	-1.27488	2.15290	-2.36122
C	-0.18513	3.18785	-3.88167
H	0.80280	3.19276	-4.35882
H	-0.91004	2.88166	-4.64455
H	-0.41969	4.21299	-3.57245
C	0.20376	0.80833	-3.15055
H	1.25533	0.82389	-3.46997
H	0.10734	0.06497	-2.34605
H	-0.40646	0.47071	-3.99778
C	2.81008	-0.34517	-0.76035
H	2.25498	0.58678	-0.85381
H	3.00331	-0.86005	-1.69945
C	5.11608	-1.17226	0.14180
C	5.19294	-2.31597	-0.66606
C	6.27945	-0.72981	0.78237
C	6.39950	-2.99404	-0.82551
H	4.30156	-2.68319	-1.17374
C	7.48886	-1.40608	0.62263
H	6.23174	0.15705	1.41304
C	7.55409	-2.54244	-0.18286
H	6.43738	-3.88017	-1.45493
H	8.38024	-1.04364	1.12991
H	8.49483	-3.07284	-0.30897
H	3.92011	0.45142	0.96971

Zero-point correction = 0.775246 (Hartree/Particle)
Thermal correction to Energy = 0.820283
Thermal correction to Enthalpy = 0.821227
Thermal correction to Gibbs Free Energy = 0.693799
Sum of electronic and zero-point Energies = -2214.812839
Sum of electronic and thermal Energies = -2214.767802
Sum of electronic and thermal Enthalpies = -2214.766858
Sum of electronic and thermal Free Energies = -2214.894286
E(CPCM(PhCl) MN15/def2-TZVP) = -3553.77017165
E(CPCM(PhCl) M06L/def2-TZVP) = -3555.37995334
E(CPCM(dioxane) M06L/def2-TZVP) = -3555.37319861
E(CPCM(dioxane) MN15L/def2-TZVP) = -3554.10647644
E(CPCM(dioxane) M06/def2-TZVP) = -3554.33815102
E(CPCM(dioxane) B3LYP-D3/def2-TZVP) = -3555.92440812
E(CPCM(dioxane) PBE0-D3/def2-TZVP) = -3553.44290162
E(CPCM(dioxane) TPSS-D3/def2-TZVP) = -3556.15063930
E(CPCM(dioxane) TPSSh-D3/def2-TZVP) = -3555.94453530
E(CPCM(dioxane) B97-D3/def2-TZVP) = -3555.34581420
E(CPCM(dioxane) wB97XD/def2-TZVP) = -3555.18879037

***trans*-4**

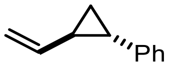

C	0.74466	0.45878	0.70171
C	1.87697	-0.35114	0.08499
H	1.56420	-1.12490	-0.61689
C	3.14539	0.33017	-0.24982
C	3.89484	0.06704	-1.32242
H	4.81967	0.60238	-1.51908
H	3.60521	-0.69718	-2.04178
H	3.46140	1.10232	0.45668
C	1.49677	-0.54654	1.52691
H	2.18409	-0.17361	2.28343
H	0.97528	-1.46055	1.80330
C	-0.66427	0.21900	0.28177
C	-1.15349	-1.06891	0.02115
C	-1.53759	1.30306	0.13171
C	-2.47430	-1.26419	-0.37740
H	-0.49615	-1.93125	0.12929
C	-2.86048	1.11086	-0.26554
H	-1.17092	2.30900	0.33064
C	-3.33539	-0.17496	-0.52248
H	-2.83264	-2.27199	-0.57474
H	-3.52008	1.96876	-0.37448
H	-4.36609	-0.32830	-0.83254
H	0.97747	1.50635	0.89270

Zero-point correction = 0.197237 (Hartree/Particle)
Thermal correction to Energy = 0.206996
Thermal correction to Enthalpy = 0.207940
Thermal correction to Gibbs Free Energy = 0.161587
Sum of electronic and zero-point Energies = -425.578180
Sum of electronic and thermal Energies = -425.568422
Sum of electronic and thermal Enthalpies = -425.567478
Sum of electronic and thermal Free Energies = -425.613831
E(CPCM(PhCl) MN15/def2-TZVP) = -425.952413566
E(CPCM(PhCl) M06L/def2-TZVP) = -426.440984449
E(CPCM(dioxane) M06L/def2-TZVP) = -426.439881029
E(CPCM(dioxane) MN15L/def2-TZVP) = -426.116621725
E(CPCM(dioxane) M06/def2-TZVP) = -426.157268016
E(CPCM(dioxane) B3LYP-D3/def2-TZVP) = -426.548809825
E(CPCM(dioxane) PBE0-D3/def2-TZVP) = -426.000951142
E(CPCM(dioxane) TPSS-D3/def2-TZVP) = -426.609628704
E(CPCM(dioxane) TPSSh-D3/def2-TZVP) = -426.564084958
E(CPCM(dioxane) B97-D3/def2-TZVP) = -426.218847302
E(CPCM(dioxane) wB97XD/def2-TZVP) = -426.354403712

Int1'-*spl*

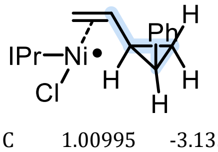

C	1.00995	-3.13371	-1.60253
H	1.59146	-3.86379	-1.04724
H	0.01281	-3.43738	-1.91187
C	1.51887	-1.90575	-1.87536
H	2.51626	-1.66097	-1.51029
C	0.74849	-0.81051	-2.45866
Ni	-0.04932	-1.02552	-0.57458
C	-0.39311	-0.99526	-3.41155
H	-0.42036	-0.29084	-4.24920
H	-0.48067	-2.01889	-3.78747

C	-1.29832	-0.67537	-2.26352
H	-1.51062	0.38429	-2.13292
C	1.74257	1.77584	1.98687
C	0.48074	2.26411	2.00491
H	2.61781	2.02455	2.56993
H	0.01990	3.04110	2.59751
C	0.54682	0.62847	0.41652
N	1.76688	0.79486	1.00083
N	-0.22770	1.56627	1.02960
H	1.23875	0.16246	-2.48891
C	-2.36915	-1.56023	-1.77340
C	-2.54116	-2.89271	-2.19262
C	-3.30858	-1.03319	-0.86099
C	-3.57631	-3.67578	-1.68421
H	-1.87135	-3.32477	-2.93318
C	-4.34730	-1.80975	-0.36651
H	-3.19749	-0.00080	-0.53800
C	-4.47870	-3.14466	-0.76288
H	-3.68066	-4.70582	-2.01736
H	-5.04882	-1.37291	0.34173
H	-5.28183	-3.76027	-0.36537
C	-1.58576	1.87317	0.67175
C	-2.61284	1.57416	1.58025
C	-1.82425	2.50994	-0.56759
C	-3.91568	1.96427	1.23580
C	-2.36769	0.86392	2.90131
C	-3.14466	2.85045	-0.87281
C	-0.68552	2.83170	-1.53063
H	-4.72916	1.75635	1.92791
C	-4.18153	2.59328	0.02603
H	-1.35375	0.44686	2.88367
C	-2.49931	1.84997	4.07041
C	-3.33128	-0.31305	3.09459
H	-3.37039	3.33105	-1.82073
H	-0.02797	1.95148	-1.55714
C	0.14319	4.02837	-1.03858
C	-1.14775	3.09204	-2.96679
H	-5.19967	2.87858	-0.22788
H	-3.51405	2.26961	4.10322
H	-1.79692	2.68894	3.98996
H	-2.31467	1.33934	5.02306
H	-3.27443	-1.00294	2.24738
H	-4.36706	0.03204	3.22029
H	-3.05502	-0.86807	3.99886
H	-0.48938	4.92185	-0.95971
H	0.95129	4.24555	-1.75127
H	0.60085	3.84108	-0.05987
H	-1.69794	4.03859	-3.04831
H	-1.79255	2.28952	-3.34673
H	-0.27105	3.16620	-3.62190
C	2.94803	0.10842	0.55399
C	3.65205	0.64988	-0.54039
C	3.33313	-1.07715	1.19692
C	4.78451	-0.03806	-0.98581
C	3.21460	1.95085	-1.19470
C	4.47608	-1.72869	0.71406
C	2.58023	-1.62048	2.39699
H	5.34999	0.34856	-1.83039
C	5.19314	-1.21852	-0.36407
H	2.12495	2.02513	-1.08177
C	3.84378	3.15480	-0.47843
C	3.53531	2.01988	-2.69100
H	4.80271	-2.64917	1.19279
H	1.58092	-1.17070	2.40698
C	3.31959	-1.23269	3.68507
C	2.37805	-3.13573	2.32051
H	6.07526	-1.74206	-0.72544
H	4.93837	3.10315	-0.54639
H	3.56744	3.18534	0.58109
H	3.51405	4.09275	-0.94346
H	3.21739	1.11419	-3.22168
H	4.61085	2.15397	-2.86484
H	3.02415	2.88189	-3.13850
H	4.32982	-1.66465	3.69381
H	2.78036	-1.60731	4.56317
H	3.41968	-0.14389	3.78250
H	3.32644	-3.68052	2.42675
H	1.90117	-3.41276	1.37427
H	1.71137	-3.45684	3.12826
Cl	-0.79900	-2.02055	1.31642

Zero-point correction = 0.775901 (Hartree/Particle)
Thermal correction to Energy = 0.820319
Thermal correction to Enthalpy = 0.821263
Thermal correction to Gibbs Free Energy = 0.697633
Sum of electronic and zero-point Energies = -2214.813890
Sum of electronic and thermal Energies = -2214.769473
Sum of electronic and thermal Enthalpies = -2214.768529
Sum of electronic and thermal Free Energies = -2214.892158
E(CPCM(dioxane) M06L/def2-TZVP) = -3555.37010681

TS1’

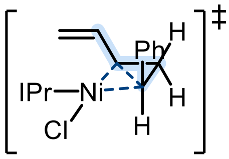

C	0.94989	-3.13928	-1.57533
H	1.48752	-3.87760	-0.98791
H	-0.03834	-3.42334	-1.92712
C	1.51898	-1.93395	-1.86345
H	2.51465	-1.72395	-1.47341
C	0.79370	-0.82755	-2.44871
Ni	-0.02571	-1.06755	-0.59228
C	-0.39893	-1.00058	-3.35086
H	-0.42521	-0.29531	-4.19038
H	-0.48085	-2.01901	-3.74564
C	-1.37773	-0.69575	-2.25769
H	-1.56693	0.36132	-2.08482
C	1.72104	1.73761	1.97542
C	0.45923	2.22505	1.99365
H	2.59698	1.98669	2.55724
H	-0.00245	3.00250	2.58497
C	0.52403	0.58705	0.40726
N	1.74519	0.75601	0.99007
N	-0.24971	1.52564	1.01968
H	1.31571	0.12640	-2.51251
C	-2.42162	-1.58536	-1.78101
C	-2.57859	-2.92342	-2.20051
C	-3.36229	-1.06994	-0.85877
C	-3.60184	-3.71563	-1.68645
H	-1.90863	-3.34520	-2.94689
C	-4.38619	-1.85770	-0.35925
H	-3.25688	-0.03714	-0.53554
C	-4.50520	-3.19412	-0.75909
H	-3.69883	-4.74662	-2.01903
H	-5.08809	-1.43181	0.35524
H	-5.29914	-3.81903	-0.35792
C	-1.60730	1.83663	0.66478
C	-2.63276	1.54036	1.57570
C	-1.84459	2.47715	-0.57251
C	-3.93506	1.93378	1.23332
C	-2.38629	0.83055	2.89682
C	-3.16461	2.82039	-0.87586
C	-0.70528	2.79780	-1.53558
H	-4.74822	1.72732	1.92624
C	-4.20084	2.56362	0.02391
H	-1.37234	0.41363	2.87850
C	-2.51689	1.81681	4.06592
C	-3.34946	-0.34654	3.09114
H	-3.39067	3.30231	-1.82302
H	-0.04917	1.91635	-1.56434
C	0.12448	3.99364	-1.04292
C	-1.16792	3.05896	-2.97133
H	-5.21883	2.85044	-0.22887
H	-3.53159	2.23640	4.09974
H	-1.81463	2.65584	3.98483
H	-2.33133	1.30624	5.01843
H	-3.29225	-1.03693	2.24439
H	-4.38540	-0.00163	3.21596
H	-3.07354	-0.90036	3.99630
H	-0.50744	4.88755	-0.96360
H	0.93260	4.21064	-1.75565

H	0.58225	3.80584	-0.06435
H	-1.71839	4.00540	-3.05249
H	-1.81217	2.25573	-3.35063
H	-0.29137	3.13342	-3.62658
C	2.92906	0.07272	0.54631
C	3.63431	0.61610	-0.54634
C	3.31610	-1.11053	1.19226
C	4.76794	-0.07083	-0.99010
C	3.19774	1.91797	-1.19979
C	4.46098	-1.76034	0.71159
C	2.56296	-1.65301	2.39272
H	5.33390	0.31612	-1.83421
C	5.17750	-1.25035	-0.36707
H	2.10759	1.99099	-1.09061
C	3.82417	3.12135	-0.47984
C	3.52502	1.99213	-2.69462
H	4.78933	-2.67939	1.19190
H	1.56370	-1.20301	2.40248
C	3.30263	-1.26484	3.68054
C	2.36030	-3.16819	2.31744
H	6.06049	-1.77303	-0.72750
H	4.91894	3.07043	-0.54519
H	3.54522	3.15087	0.57900
H	3.49512	4.05956	-0.94481
H	3.21644	1.08649	-3.23033
H	4.60067	2.13352	-2.86215
H	3.01090	2.85227	-3.14228
H	4.31267	-1.69720	3.68938
H	2.76339	-1.63879	4.55891
H	3.40322	-0.17605	3.77739
H	3.30889	-3.71332	2.41933
H	1.87904	-3.44516	1.37350
H	1.69731	-3.48925	3.12828
Cl	-0.81895	-2.04011	1.29477

Zero-point correction = 0.773737 (Hartree/Particle)
Thermal correction to Energy = 0.817771
Thermal correction to Enthalpy = 0.818715
Thermal correction to Gibbs Free Energy = 0.698361
Sum of electronic and zero-point Energies = -2214.774226
Sum of electronic and thermal Energies = -2214.730192
Sum of electronic and thermal Enthalpies = -2214.729248
Sum of electronic and thermal Free Energies = -2214.849602
E(CPCM(dioxane) M06L/def2-TZVP) = -3555.35159176

Int2'

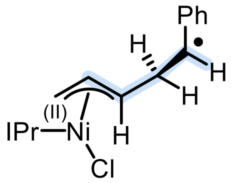

Int2'

C	0.17165	-3.27850	-0.01151
H	0.52593	-3.83656	0.85109
H	-0.87620	-3.42372	-0.27082
C	1.08447	-2.70680	-0.90383
H	2.15356	-2.74469	-0.67983
C	0.62596	-1.72920	-1.81806
Ni	0.21340	-1.22157	0.09929
C	-0.76350	-1.80280	-2.46054
H	-0.73690	-1.21861	-3.39399
H	-0.96821	-2.84242	-2.75015
C	-1.84860	-1.27583	-1.56698
H	-1.73498	-0.24916	-1.23234
C	2.08335	2.28155	0.92652
C	0.84326	2.81989	0.87233
H	3.03194	2.69152	1.24213
H	0.47857	3.80910	1.10791
C	0.66586	0.66319	0.16998
N	1.95980	0.97537	0.46681
N	-0.00669	1.82564	0.39058
H	1.37997	-1.16512	-2.36352
C	-2.98151	-1.96341	-1.06894
C	-3.33945	-3.29045	-1.43163
C	-3.81152	-1.29239	-0.12501
C	-4.46156	-3.89873	-0.88457
H	-2.73270	-3.83631	-2.15148
C	-4.92672	-1.90981	0.41422
H	-3.53207	-0.28409	0.18077
C	-5.26388	-3.21819	0.03837
H	-4.71732	-4.91444	-1.17851
H	-5.53767	-1.37824	1.14035
H	-6.13858	-3.70294	0.46432
C	-1.39310	2.07536	0.09230
C	-2.29526	2.26197	1.15080
C	-1.77388	2.18611	-1.26382
C	-3.61593	2.60145	0.82284
C	-1.90026	2.12569	2.61142
C	-3.10792	2.50030	-1.53352
C	-0.77744	1.95220	-2.39582
H	-4.33341	2.76309	1.62461
C	-4.01988	2.71961	-0.50070
H	-0.92073	1.63566	2.65668
C	-1.81658	3.50611	3.27750
C	-2.88837	1.23455	3.37483
H	-3.44429	2.57773	-2.56332
H	-0.17035	1.07637	-2.12139
C	0.17143	3.14745	-2.57309
C	-1.45054	1.62560	-3.73081
H	-5.05081	2.97362	-0.73535
H	-2.79111	4.01117	3.23364
H	-1.08195	4.15975	2.79123
H	-1.53672	3.40411	4.33288
H	-3.03190	0.28009	2.85855
H	-3.86078	1.73061	3.49856
H	-2.49534	1.02150	4.37576
H	-0.39872	4.05344	-2.81545
H	0.87010	2.95428	-3.39934
H	0.76471	3.34355	-1.67178
H	-1.96036	2.50201	-4.15187
H	-2.18441	0.81691	-3.62337
H	-0.69026	1.31067	-4.45624
C	3.04398	0.05409	0.24845
C	3.62707	0.00990	-1.03469
C	3.44560	-0.78437	1.29827
C	4.64701	-0.92138	-1.24709
C	3.18103	0.95874	-2.13745
C	4.48192	-1.69156	1.03828
C	2.81832	-0.70017	2.67756
H	5.11520	-0.99148	-2.22578
C	5.07290	-1.76395	-0.21904
H	2.10347	1.12999	-2.00496
C	3.89753	2.31253	-2.01756
C	3.40079	0.40871	-3.55035
H	4.82087	-2.35114	1.83407
H	1.85579	-0.18321	2.58621
C	3.73077	0.10603	3.61199
C	2.52178	-2.08335	3.26292
H	5.87047	-2.47994	-0.40347
H	4.98101	2.17415	-2.12740
H	3.71001	2.79303	-1.05215
H	3.55672	2.99452	-2.80714
H	3.01780	-0.61185	-3.66767
H	4.46695	0.40060	-3.81183
H	2.89314	1.05394	-4.27797
H	4.70936	-0.38339	3.71080
H	3.28489	0.18278	4.61072
H	3.90025	1.12198	3.23239
H	3.44345	-2.62655	3.51278
H	1.92925	-2.68069	2.56057
H	1.93297	-1.97594	4.18044
Cl	-0.70530	-1.17234	2.12278

Zero-point correction = 0.775145 (Hartree/Particle)
Thermal correction to Energy = 0.819036
Thermal correction to Enthalpy = 0.819980
Thermal correction to Gibbs Free Energy = 0.699608
Sum of electronic and zero-point Energies = -2214.788951
Sum of electronic and thermal Energies = -2214.745060
Sum of electronic and thermal Enthalpies = -2214.744116
Sum of electronic and thermal Free Energies = -2214.864487
E(CPCM(dioxane) M06L/def2-TZVP) = -3555.36348819

Ni$^{(0)}$(IPr)$_2$

C	-0.71644	4.09653	0.09880
C	0.48875	4.05545	0.70633
H	-1.39082	4.91489	-0.11167
H	1.10604	4.83131	1.13639
C	-0.02783	1.89761	0.08423
N	-1.01294	2.78912	-0.28211
N	0.89374	2.72358	0.68654
C	2.22493	2.36361	1.08170
C	3.22404	2.34109	0.09150
C	2.50675	2.18189	2.44352
C	4.54443	2.13907	0.50608
C	2.88453	2.51335	-1.37893
C	3.84632	2.01487	2.81489
C	1.41178	2.20631	3.49609
H	5.33838	2.11076	-0.23753
C	4.85740	1.99983	1.85775
H	1.89098	2.97665	-1.44282
C	3.86907	3.42693	-2.11548
C	2.80641	1.13993	-2.05636
H	4.09770	1.89690	3.86766
H	0.44328	2.17216	2.97836
C	1.48681	3.50150	4.31476
C	1.48600	0.98127	4.41220
H	5.89290	1.86875	2.16420
H	4.85851	2.96100	-2.20844
H	3.99154	4.38951	-1.60515
H	3.50459	3.61926	-3.13252
H	2.04959	0.50782	-1.56683
H	3.77469	0.62413	-1.99470
H	2.53937	1.24893	-3.11932
H	2.44965	3.56880	4.83896
H	0.68799	3.53330	5.06579
H	1.39118	4.38620	3.67338
H	2.44599	0.93154	4.94250
H	1.35973	0.05731	3.83482
H	0.69175	1.02834	5.16716
C	-2.20576	2.49083	-1.02709
C	-3.43308	2.49616	-0.34247
C	-2.11409	2.30268	-2.41978
C	-4.60109	2.32483	-1.09411
C	-3.47619	2.61176	1.17237
C	-3.31046	2.14269	-3.12728
C	-0.76451	2.26408	-3.12304
H	-5.56650	2.32695	-0.59484
C	-4.54162	2.16385	-2.47531
H	-2.68770	3.30848	1.48800
C	-4.80811	3.14229	1.70733
C	-3.17183	1.23608	1.78034
H	-3.28236	1.99326	-4.20303
H	-0.09091	1.66980	-2.48475
C	-0.16649	3.66833	-3.29620
C	-0.82769	1.58382	-4.49012
H	-5.45887	2.04354	-3.04770
H	-5.61153	2.40664	1.57204
H	-5.10972	4.07381	1.21292
H	-4.72405	3.33355	2.78331
H	-2.17843	0.86925	1.48261
H	-3.91159	0.50658	1.42772
H	-3.22354	1.27424	2.87732
H	-0.80902	4.27513	-3.94856
H	0.82468	3.59705	-3.76615
H	-0.04958	4.19542	-2.34350
H	-1.39106	2.18833	-5.21406
H	-1.29476	0.59386	-4.42610
H	0.18681	1.46179	-4.88766
Ni	0.00005	-0.00001	-0.02403
C	0.02793	-1.89774	0.08156
N	1.01305	-2.78864	-0.28625
N	-0.89363	-2.72471	0.68254
C	0.71653	-4.09669	0.09246
C	2.20580	-2.48910	-1.03084
C	-0.48867	-4.05661	0.70003
C	-2.22491	-2.36542	1.07803
H	1.39095	-4.91469	-0.11932
C	3.43321	-2.49567	-0.34640
C	2.11394	-2.29852	-2.42317
H	-1.10595	-4.83319	1.12881
C	-3.22377	-2.34101	0.08764
C	-2.50704	-2.18610	2.44011
C	4.60112	-2.32313	-1.09791
C	3.47652	-2.61387	1.16824
C	3.31021	-2.13734	-3.13056
C	0.76425	-2.25867	-3.12613
C	-4.54424	-2.13956	0.50225
C	-2.88400	-2.51078	-1.38303
C	-3.84668	-2.01962	2.81146
C	-1.41230	-2.21251	3.49288
H	5.56660	-2.32617	-0.59878
C	4.54147	-2.15973	-2.47882
H	2.68795	-3.31100	1.48278
C	4.80841	-3.14556	1.70211
C	3.17240	-1.23918	1.77857
H	3.28195	-1.98607	-4.20606
H	0.09065	-1.66588	-2.48643
C	0.16645	-3.66266	-3.30215
C	0.82703	-1.57536	-4.49170
H	-5.33801	-2.10982	-0.24150
C	-4.85752	-2.00272	1.85410
H	-1.89033	-2.97370	-1.44752
C	-3.86817	-3.42349	-2.12115
C	-2.80614	-1.13624	-2.05820
H	-4.09832	-1.90351	3.86437
H	-0.44370	-2.17738	2.97541
C	-1.48750	-3.50925	4.30907
C	-1.48673	-0.98920	4.41129
H	5.45866	-2.03850	-3.05114
H	5.61195	-2.40982	1.56802
H	5.10979	-4.07626	1.20601
H	4.72447	-3.33870	2.77776
H	2.17904	-0.87174	1.48149
H	3.91224	-0.50919	1.42716
H	3.22414	-1.27920	2.87549
H	0.80888	-4.26794	-3.95602
H	-0.82489	-3.59054	-3.77162
H	0.04997	-4.19179	-2.35053
H	1.39034	-2.17809	-5.21716
H	1.29395	-0.58547	-4.42548
H	-0.18760	-1.45262	-4.88873
H	-5.89308	-1.87207	2.16054
H	-4.85779	-2.95781	-2.21339
H	-3.99028	-4.38696	-1.61242
H	-3.50356	-3.61400	-3.13849
H	-2.04955	-0.50475	-1.56751
H	-3.77456	-0.62078	-1.99586
H	-2.53889	-1.24339	-3.12130
H	-2.45045	-3.57754	4.83294
H	-0.68883	-3.54250	5.06020
H	-1.39175	-4.39272	3.66602
H	-2.44673	-0.94063	4.94167
H	-1.36065	-0.06414	3.83562
H	-0.69246	-1.03752	5.16614

Zero-point correction = 1.151224 (Hartree/Particle)
Thermal correction to Energy = 1.213409
Thermal correction to Enthalpy = 1.214354
Thermal correction to Gibbs Free Energy = 1.056396
Sum of electronic and zero-point Energies = -2487.087908
Sum of electronic and thermal Energies = -2487.025722
Sum of electronic and thermal Enthalpies = -2487.024778
Sum of electronic and thermal Free Energies = -2487.182736
E(CPCM(dioxane) M06L/def2-TZVP) = -3828.97175001

IPr

C	-0.67523	0.03681	1.93680
C	0.67541	-0.03521	1.93670
H	-1.39392	0.08063	2.74325
H	1.39427	-0.07840	2.74304
C	-0.00013	-0.00034	-0.27194
N	-1.05946	0.04979	0.59647
N	1.05940	-0.04949	0.59630
C	2.42943	-0.14946	0.18003
C	3.23662	1.00164	0.24118
C	2.91521	-1.39237	-0.25252

C	4.57615	0.87696	-0.13613
C	2.63125	2.34585	0.61535
C	4.26027	-1.46582	-0.63520
C	2.00798	-2.60435	-0.37966
H	5.22965	1.74480	-0.09742
C	5.08460	-0.34711	-0.56978
H	1.87011	2.17292	1.38852
C	3.64649	3.34534	1.17147
C	1.91990	2.93295	-0.61393
H	4.66401	-2.41274	-0.98774
H	1.11021	-2.42605	0.22826
C	2.66299	-3.89727	0.11536
C	1.56512	-2.74470	-1.84261
H	6.12888	-0.42518	-0.86363
H	4.34793	3.67441	0.39402
H	4.22785	2.92117	1.99870
H	3.12691	4.23867	1.53667
H	1.19569	2.22012	-1.02467
H	2.65531	3.16331	-1.39617
H	1.39291	3.86082	-0.35249
H	3.49562	-4.20059	-0.53199
H	1.92984	-4.71283	0.10255
H	3.04674	-3.79348	1.13678
H	2.44080	-2.89959	-2.48777
H	1.04493	-1.83686	-2.16831
H	0.89185	-3.60318	-1.96390
C	-2.42948	0.14948	0.18009
C	-3.23641	-1.00176	0.24109
C	-2.91544	1.39232	-0.25247
C	-4.57589	-0.87741	-0.13657
C	-2.63085	-2.34579	0.61557
C	-4.26043	1.46551	-0.63534
C	-2.00831	2.60438	-0.37957
H	-5.22916	-1.74544	-0.09809
C	-5.08453	0.34657	-0.57022
H	-1.86937	-2.17245	1.38832
C	-3.64588	-3.34498	1.17267
C	-1.92001	-2.93344	-0.61370
H	-4.66437	2.41236	-0.98784
H	-1.11056	2.42619	0.22844
C	-2.66346	3.89722	0.11540
C	-1.56538	2.74462	-1.84251
H	-6.12875	0.42443	-0.86430
H	-4.34766	-3.67450	0.39573
H	-4.22686	-2.92031	1.99991
H	-3.12617	-4.23813	1.53816
H	-1.19594	-2.22073	-1.02495
H	-2.65566	-3.16414	-1.39562
H	-1.39290	-3.86118	-0.35206
H	-3.49629	4.20035	-0.53179
H	-1.93048	4.71293	0.10239
H	-3.04697	3.79343	1.13691
H	-2.44103	2.89941	-2.48773
H	-1.04514	1.83677	-2.16810
H	-0.89211	3.60309	-1.96386

Zero-point correction = 0.574438 (Hartree/Particle)
Thermal correction to Energy = 0.604130
Thermal correction to Enthalpy = 0.605074
Thermal correction to Gibbs Free Energy = 0.515108
Sum of electronic and zero-point Energies = -1157.939224
Sum of electronic and thermal Energies = -1157.909531
Sum of electronic and thermal Enthalpies = -1157.908587
Sum of electronic and thermal Free Energies = -1157.998554
E(CPCM(dioxane) M06L/def2-TZVP) = -1160.28735805

Int5

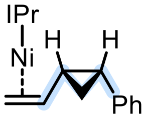

C	0.19873	-2.00590	-2.16407
H	-0.13870	-2.95450	-1.74583
H	-0.26525	-1.70962	-3.10750
C	1.37086	-1.41318	-1.72887
H	1.95255	-1.89575	-0.93783
C	2.06376	-0.31121	-2.45036
Ni	-0.19559	-0.60525	-0.78687
C	2.64674	0.83713	-1.66040
H	2.57395	1.84288	-2.06632
H	2.51850	0.78195	-0.57910
C	3.57364	-0.14005	-2.31588
C	-2.93032	1.20824	1.73064
C	-1.85837	1.98361	2.02251
H	-3.94397	1.21457	2.10484
H	-1.73389	2.80777	2.71025
C	-1.18878	0.47081	0.43998
N	-2.50218	0.29478	0.77399
N	-0.81420	1.52662	1.22340
H	4.06988	0.20425	-3.22410
H	1.63639	-0.06295	-3.42255
C	0.51758	2.05759	1.23552
C	0.79465	3.18796	0.44549
C	1.48749	1.42074	2.02408
C	2.10028	3.68673	0.46713
C	-0.27561	3.79203	-0.45039
C	2.78106	1.95958	2.01642
C	1.18316	0.18006	2.84761
H	2.35325	4.55636	-0.13498
C	3.08525	3.07967	1.24701
H	-1.25415	3.56729	-0.00418
C	-0.16143	5.31289	-0.58397
C	-0.23269	3.12788	-1.83540
H	3.55710	1.48860	2.61858
H	0.12400	-0.07657	2.71067
C	1.42598	0.43235	4.34032
C	2.01203	-1.00885	2.34793
H	4.09566	3.48181	1.25109
H	0.73446	5.59797	-1.15007
H	-0.11810	5.80625	0.39404
H	-1.02649	5.70466	-1.13154
H	-0.28994	2.03248	-1.75679
H	0.70719	3.38455	-2.34370
H	-1.06539	3.48282	-2.45745
H	2.47997	0.67261	4.53161
H	1.17721	-0.46265	4.92322
H	0.81759	1.26610	4.71024
H	3.08640	-0.83364	2.49722
H	1.83804	-1.17790	1.27667
H	1.73924	-1.92182	2.89237
C	-3.30823	-0.72660	0.16977
C	-3.44728	-1.95315	0.84163
C	-3.88266	-0.47218	-1.08448
C	-4.20360	-2.94840	0.21644
C	-2.72785	-2.21819	2.15480
C	-4.63038	-1.49978	-1.67096
C	-3.68772	0.84467	-1.81880
H	-4.32655	-3.91377	0.70238
C	-4.79117	-2.72397	-1.02804
H	-2.51670	-1.24998	2.62942
C	-3.56108	-3.04781	3.13615
C	-1.38247	-2.90537	1.87533
H	-5.08433	-1.33772	-2.64729
H	-3.12268	1.52609	-1.16773
C	-5.03089	1.50602	-2.14955
C	-2.85684	0.62581	-3.08932
H	-5.37393	-3.51145	-1.50044
H	-3.69337	-4.07645	2.77770
H	-4.55459	-2.61276	3.29581
H	-3.04904	-3.10694	4.10376
H	-0.76788	-2.30528	1.18812
H	-1.55234	-3.88631	1.41040
H	-0.82507	-3.05820	2.80952
H	-5.61932	0.88238	-2.83481
H	-4.86761	2.47331	-2.64000
H	-5.63044	1.67257	-1.24702
H	-3.37640	-0.05094	-3.78118
H	-1.88278	0.18455	-2.83640
H	-2.68831	1.57916	-3.60676
C	4.37794	-1.11102	-1.51043
C	4.80221	-2.31222	-2.09710
C	4.71557	-0.86625	-0.17497
C	5.54247	-3.24209	-1.37071

H	4.53483	-2.51471	-3.13318
C	5.45226	-1.79893	0.55874
H	4.39827	0.06467	0.29274
C	5.86879	-2.98909	-0.03579
H	5.86039	-4.16887	-1.84319
H	5.70250	-1.59106	1.59743
H	6.44261	-3.71571	0.53468

Zero-point correction = 0.772626 (Hartree/Particle)
Thermal correction to Energy = 0.815512
Thermal correction to Enthalpy = 0.816456
Thermal correction to Gibbs Free Energy = 0.694991
Sum of electronic and zero-point Energies = -1754.698337
Sum of electronic and thermal Energies = -1754.655451
Sum of electronic and thermal Enthalpies = -1754.654507
Sum of electronic and thermal Free Energies = -1754.775972
E(CPCM(dioxane) M06L/def2-TZVP) = -3095.09683193

TS4

C	2.57682	-2.58315	-2.00244
H	3.38434	-2.89498	-1.34513
H	2.13341	-3.35407	-2.63014
C	2.17881	-1.29567	-2.02854
H	2.71876	-0.58243	-1.40719
C	1.12300	-0.70640	-2.87289
Ni	-0.00900	-0.37724	-1.08412
C	0.37816	-1.52511	-3.87499
H	0.29701	-1.13841	-4.89058
H	0.57751	-2.59739	-3.86034
C	-0.63786	-1.04573	-2.88990
H	-1.20179	-0.17191	-3.22379
C	1.12280	2.02962	2.14099
C	-0.17933	2.37564	2.02868
H	1.90179	2.36611	2.81090
H	-0.79101	3.06632	2.59153
C	0.23953	0.78373	0.41993
N	1.36353	1.07185	1.15816
N	-0.69776	1.62152	0.97883
H	1.30162	0.32900	-3.16547
C	-1.38552	-1.91950	-1.96645
C	-0.93088	-3.19666	-1.56531
C	-2.65146	-1.49423	-1.49979
C	-1.73843	-4.03108	-0.79616
H	0.05343	-3.53595	-1.87677
C	-3.46685	-2.35113	-0.76383
H	-3.00455	-0.49680	-1.76642
C	-3.02266	-3.62905	-0.41917
H	-1.36896	-5.01364	-0.50974
H	-4.44995	-2.00824	-0.44837
H	-3.66045	-4.29665	0.15562
C	-2.10596	1.45110	0.76577
C	-2.76188	0.49627	1.55901
C	-2.76779	2.23363	-0.19372
C	-4.13149	0.30853	1.34437
C	-2.03190	-0.31294	2.62098
C	-4.13392	1.99986	-0.38414
C	-2.01649	3.27268	-1.00917
H	-4.66719	-0.43093	1.93839
C	-4.80909	1.04292	0.37425
H	-0.98609	0.01901	2.65803
C	-2.63708	-0.07447	4.00924
C	-2.02430	-1.80312	2.27069
H	-4.67991	2.58004	-1.12456
H	-1.15235	3.59741	-0.41303
C	-2.86467	4.50749	-1.32725
C	-1.47974	2.64362	-2.30144
H	-5.87263	0.87954	0.21441
H	-3.67702	-0.42328	4.05380
H	-2.62589	0.99011	4.27279
H	-2.06673	-0.62447	4.76831
H	-1.54422	-1.97376	1.29834
H	-3.04633	-2.20093	2.21592
H	-1.48129	-2.37044	3.03930
H	-3.65883	4.27138	-2.04702
H	-2.23768	5.28312	-1.78215
H	-3.33303	4.92353	-0.42760
H	-2.31401	2.25455	-2.90389
H	-0.80242	1.80830	-2.07126
H	-0.93831	3.38738	-2.90090
C	2.62664	0.41500	0.97957
C	3.68050	1.13468	0.38824
C	2.75019	-0.92753	1.37989
C	4.90145	0.47354	0.21854
C	3.45834	2.53863	-0.15463
C	3.99059	-1.54489	1.18799
C	1.58626	-1.66932	2.01569
H	5.73828	0.99615	-0.23721
C	5.05563	-0.85358	0.61529
H	2.78723	3.07237	0.53153
C	4.74435	3.35804	-0.27913
C	2.75444	2.44737	-1.51851
H	4.11944	-2.58427	1.48069
H	0.66898	-1.26540	1.57315
C	1.55540	-1.40219	3.52693
C	1.58080	-3.16802	1.71204
H	6.01096	-1.35303	0.47122
H	5.38971	2.96688	-1.07600
H	5.31893	3.36325	0.65474
H	4.50007	4.39400	-0.54043
H	1.79876	1.91128	-1.43929
H	3.39187	1.90283	-2.22961
H	2.56479	3.44960	-1.92420
H	2.46791	-1.78329	4.00499
H	0.69332	-1.90072	3.99019
H	1.48179	-0.32760	3.74017
H	2.39548	-3.69932	2.22289
H	1.66392	-3.34118	0.63164
H	0.63452	-3.60530	2.05521

Zero-point correction = 0.772511 (Hartree/Particle)
Thermal correction to Energy = 0.814245
Thermal correction to Enthalpy = 0.815189
Thermal correction to Gibbs Free Energy = 0.700930
Sum of electronic and zero-point Energies = -1754.678298
Sum of electronic and thermal Energies = -1754.636564
Sum of electronic and thermal Enthalpies = -1754.635620
Sum of electronic and thermal Free Energies = -1754.749879
E(CPCM(dioxane) M06L/def2-TZVP) = -3095.07765443

Int6

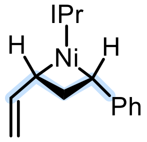

C	2.34121	2.51572	2.32313
H	3.20579	2.95507	1.83120
H	1.72944	3.18679	2.92343
C	2.08130	1.19707	2.20133
H	2.78331	0.60619	1.61070
C	0.97206	0.44078	2.78806
Ni	-0.19472	0.18664	1.18892
C	-0.03394	1.09954	3.69593
H	-0.13748	0.63756	4.68471
H	0.12674	2.17455	3.83747
C	-1.16532	0.77894	2.74957
H	-1.79658	-0.06896	3.03787
C	1.38308	-1.72139	-2.28162
C	0.11024	-2.17999	-2.23682
H	2.19486	-1.91770	-2.96797
H	-0.43642	-2.85229	-2.88250
C	0.38317	-0.75061	-0.46496
N	1.53238	-0.85925	-1.19781
N	-0.47578	-1.58310	-1.12707
H	1.21851	-0.59342	3.04841
C	-1.84275	1.76952	1.91658
C	-1.27256	3.02051	1.57408
C	-3.12413	1.47633	1.39257
C	-1.98507	3.94865	0.81838
H	-0.26708	3.25702	1.91526
C	-3.84055	2.41925	0.66222
H	-3.56048	0.50100	1.60796
C	-3.28336	3.66946	0.38071

H	-1.52501	4.90599	0.58010
H	-4.83708	2.16964	0.30023
H	-3.84299	4.40775	-0.18885
C	-1.88383	-1.57295	-0.85208
C	-2.67106	-0.65996	-1.57409
C	-2.40443	-2.43455	0.12560
C	-4.03902	-0.62160	-1.28601
C	-2.07410	0.26610	-2.62423
C	-3.77532	-2.34283	0.39400
C	-1.53394	-3.44625	0.85333
H	-4.67732	0.07888	-1.82278
C	-4.58518	-1.45003	-0.30714
H	-1.00047	0.05339	-2.71179
C	-2.70713	0.01936	-3.99848
C	-2.21126	1.73306	-2.20818
H	-4.21351	-2.98760	1.15397
H	-0.54460	-3.44287	0.37709
C	-2.11409	-4.86145	0.73708
C	-1.34066	-3.05905	2.32405
H	-5.64937	-1.39978	-0.08774
H	-3.78173	0.24279	-3.98289
H	-2.58412	-1.02374	-4.31463
H	-2.24191	0.66633	-4.75216
H	-1.74766	1.91389	-1.22923
H	-3.26637	2.02539	-2.13419
H	-1.73172	2.38397	-2.95208
H	-3.07399	-4.93961	1.26329
H	-1.43008	-5.58832	1.19107
H	-2.27855	-5.14638	-0.30852
H	-2.31077	-2.97535	2.83379
H	-0.82464	-2.09334	2.40144
H	-0.74592	-3.82036	2.84493
C	2.72738	-0.10779	-0.93258
C	3.84211	-0.78492	-0.40774
C	2.71596	1.27499	-1.18389
C	4.99105	-0.03018	-0.15067
C	3.75568	-2.25703	-0.03052
C	3.88898	1.98524	-0.90787
C	1.48756	1.96609	-1.75435
H	5.87429	-0.51433	0.25708
C	5.01397	1.34152	-0.39880
H	3.16717	-2.77637	-0.79906
C	5.11921	-2.94603	0.05282
C	3.00981	-2.40388	1.30641
H	3.91544	3.05836	-1.08059
H	0.60682	1.44864	-1.35644
C	1.47644	1.83487	-3.28401
C	1.35477	3.42775	-1.32473
H	5.91473	1.91289	-0.18691
H	5.69653	-2.58083	0.91172
H	5.71542	-2.78634	-0.85339
H	4.98159	-4.02452	0.19042
H	1.99804	-1.98048	1.25647
H	3.55694	-1.87504	2.09906
H	2.93414	-3.46178	1.58901
H	2.35618	2.32950	-3.71715
H	0.57844	2.30552	-3.70506
H	1.48873	0.78260	-3.59689
H	2.10402	4.06997	-1.80761
H	1.45455	3.52032	-0.23611
H	0.36263	3.80059	-1.61021

Zero-point correction = 0.772622 (Hartree/Particle)
Thermal correction to Energy = 0.815047
Thermal correction to Enthalpy = 0.815991
Thermal correction to Gibbs Free Energy = 0.699230
Sum of electronic and zero-point Energies = -1754.682649
Sum of electronic and thermal Energies = -1754.640224
Sum of electronic and thermal Enthalpies = -1754.639279
Sum of electronic and thermal Free Energies = -1754.756041
E(CPCM(dioxane) M06L/def2-TZVP) = -3095.08868722

Int7

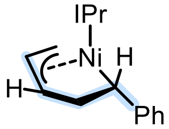

C	-1.33411	0.88121	-2.69016
H	-1.73863	1.88487	-2.58027
H	-0.33291	0.82603	-3.12537
C	-2.17550	-0.23265	-2.67847
H	-3.21272	-0.07659	-2.37078
C	-1.71919	-1.57278	-2.64716
Ni	-0.82466	-0.54842	-1.17969
C	-0.35702	-2.11977	-2.99835
H	-0.38242	-3.04004	-3.60339
H	0.25056	-1.38431	-3.54450
C	0.15206	-2.27733	-1.56050
H	-0.25022	-3.19142	-1.11107
C	0.26527	1.44062	2.37753
C	-1.00846	1.01324	2.51373
H	0.91677	1.99044	3.04223
H	-1.71569	1.10817	3.32493
C	-0.26688	0.32072	0.45419
N	0.70551	1.01248	1.12195
N	-1.31772	0.34062	1.33821
H	-2.45784	-2.32166	-2.35406
C	1.62302	-2.18105	-1.39466
C	2.37219	-3.29552	-0.97362
C	2.33703	-1.02699	-1.76970
C	3.76726	-3.26945	-0.95378
H	1.84223	-4.20540	-0.69204
C	3.72902	-0.99258	-1.73852
H	1.77760	-0.14262	-2.08608
C	4.45551	-2.11644	-1.33475
H	4.31759	-4.15383	-0.63755
H	4.24755	-0.08086	-2.02701
H	5.54280	-2.09214	-1.31814
C	2.03640	1.32709	0.67353
C	2.20440	2.24333	-0.38414
C	3.11920	0.77165	1.37031
C	3.51066	2.58793	-0.73886
C	1.00099	2.85353	-1.08254
C	4.40938	1.15799	0.98379
C	2.95253	-0.22664	2.50519
H	3.67618	3.28735	-1.55426
C	4.60643	2.05800	-0.05587
H	0.24474	2.06514	-1.16662
C	0.39585	3.99093	-0.24635
C	1.30158	3.33596	-2.50191
H	5.26664	0.73573	1.50530
H	1.88269	-0.41092	2.65789
C	3.53081	0.32834	3.81281
C	3.59250	-1.56971	2.14459
H	5.61507	2.34596	-0.34346
H	1.11292	4.81524	-0.13671
H	0.10978	3.64972	0.75697
H	-0.50496	4.38356	-0.73902
H	1.78952	2.55175	-3.09547
H	1.94884	4.22316	-2.50664
H	0.36544	3.61095	-3.00332
H	4.61113	0.49897	3.72100
H	3.37351	-0.38263	4.63277
H	3.06662	1.28333	4.09059
H	4.66570	-1.45905	1.94130
H	3.12428	-1.99182	1.25027
H	3.47596	-2.27905	2.97400
C	-2.62875	-0.11812	0.96282
C	-2.92902	-1.48788	1.07245
C	-3.54302	0.82574	0.45894
C	-4.21316	-1.89722	0.69843
C	-1.89991	-2.47329	1.60098
C	-4.82149	0.36950	0.11846
C	-3.15531	2.28212	0.25366
H	-4.48134	-2.94826	0.76662
C	-5.15415	-0.97745	0.23883
H	-0.91599	-2.13368	1.25037
C	-1.90692	-2.47695	3.13638

C	-2.11162	-3.89121	1.06695
H	-5.55674	1.07319	-0.26527
H	-2.06816	2.30836	0.08715
C	-3.48841	3.14056	1.48262
C	-3.83384	2.90148	-0.97489
H	-6.15009	-1.31568	-0.03802
H	-2.89220	-2.78403	3.51147
H	-1.68100	-1.48442	3.54401
H	-1.15979	-3.18116	3.52237
H	-2.23101	-3.88855	-0.02347
H	-2.99734	-4.36406	1.51223
H	-1.24615	-4.51557	1.31760
H	-4.56975	3.11777	1.67260
H	-3.19438	4.18320	1.30703
H	-2.97693	2.79260	2.38502
H	-4.88994	3.12527	-0.77432
H	-3.78945	2.24035	-1.84749
H	-3.34428	3.84948	-1.23084

Zero-point correction = 0.773444 (Hartree/Particle)
Thermal correction to Energy = 0.815331
Thermal correction to Enthalpy = 0.816276
Thermal correction to Gibbs Free Energy = 0.700830
Sum of electronic and zero-point Energies = -1754.682278
Sum of electronic and thermal Energies = -1754.640391
Sum of electronic and thermal Enthalpies = -1754.639446
Sum of electronic and thermal Free Energies = -1754.754892
E(CPCM(dioxane) M06L/def2-TZVP) = -3095.09273229

TS6

C	-0.77708	1.00645	-3.21583
H	-0.84507	2.04460	-3.54193
H	0.00156	0.45896	-3.76833
C	-2.04121	0.31056	-3.03522
H	-2.93979	0.93336	-2.97590
C	-2.21388	-1.02012	-2.77660
Ni	-0.33358	0.18807	-1.37341
C	-1.02913	-1.93704	-2.63046
H	-1.32231	-2.99846	-2.68578
H	-0.30059	-1.78575	-3.44384
C	-0.31698	-1.71813	-1.27859
H	-0.93850	-2.08393	-0.46090
C	0.51838	0.98724	2.53371
C	-0.74585	0.51590	2.63212
H	1.17964	1.42096	3.26907
H	-1.42702	0.45966	3.46915
C	-0.09982	0.29631	0.45656
N	0.90272	0.84510	1.20121
N	-1.10662	0.08226	1.36094
H	-3.20656	-1.40767	-2.55328
C	1.05353	-2.29322	-1.22811
C	1.33352	-3.38472	-0.38890
C	2.04326	-1.88384	-2.13991
C	2.52857	-4.09414	-0.50925
H	0.57339	-3.71149	0.32197
C	3.24355	-2.58437	-2.25248
H	1.84398	-1.02135	-2.77791
C	3.48026	-3.70778	-1.45526
H	2.71237	-4.95683	0.12794
H	3.98965	-2.26322	-2.97595
H	4.40744	-4.26676	-1.55855
C	2.16185	1.32307	0.68752
C	2.17239	2.49246	-0.10138
C	3.33348	0.63887	1.04518
C	3.41425	2.95913	-0.53895
C	0.89860	3.26540	-0.40380
C	4.55353	1.16271	0.59921
C	3.32106	-0.61762	1.90050
H	3.45905	3.85232	-1.15627
C	4.59547	2.30690	-0.18828
H	0.09341	2.53184	-0.54336
C	0.54020	4.17676	0.77890
C	0.96078	4.06389	-1.70530
H	5.47853	0.65724	0.86826
H	2.30034	-1.02453	1.89712
C	3.73466	-0.30118	3.34493
C	4.25133	-1.69257	1.33081
H	5.55122	2.69502	-0.53268
H	1.32885	4.92401	0.93907
H	0.41681	3.60673	1.70885
H	-0.39979	4.70983	0.58135
H	1.27244	3.42508	-2.53951
H	1.64440	4.92046	-1.63322
H	-0.03772	4.45641	-1.93532
H	4.74920	0.11907	3.35653
H	3.73963	-1.21486	3.95178
H	3.06976	0.42468	3.82611
H	5.30521	-1.43031	1.49381
H	4.08691	-1.82580	0.25846
H	4.07019	-2.65282	1.82832
C	-2.45944	-0.17752	0.94178
C	-2.98439	-1.47596	1.03592
C	-3.19449	0.91062	0.43926
C	-4.28062	-1.68291	0.55302
C	-2.21211	-2.60220	1.70474
C	-4.48298	0.65154	-0.03864
C	-2.67671	2.33964	0.52105
H	-4.71919	-2.67686	0.60590
C	-5.01662	-0.63405	0.00519
H	-1.14446	-2.33985	1.68165
C	-2.64005	-2.71779	3.17499
C	-2.38666	-3.95210	1.00003
H	-5.07831	1.47118	-0.43699
H	-1.61425	2.31041	0.79259
C	-3.41343	3.07788	1.64817
C	-2.78727	3.11034	-0.79695
H	-6.01903	-0.81765	-0.37428
H	-3.71120	-2.94981	3.23869
H	-2.46714	-1.78138	3.71905
H	-2.08495	-3.51743	3.68042
H	-2.17956	-3.88038	-0.07492
H	-3.40617	-4.33822	1.12473
H	-1.70423	-4.69258	1.43507
H	-4.48482	3.16021	1.42237
H	-3.00934	4.09132	1.76705
H	-3.30923	2.54947	2.60426
H	-3.82513	3.16043	-1.15176
H	-2.17204	2.64412	-1.57442
H	-2.43968	4.14231	-0.65196

Zero-point correction = 0.771542 (Hartree/Particle)
Thermal correction to Energy = 0.813189
Thermal correction to Enthalpy = 0.814133
Thermal correction to Gibbs Free Energy = 0.699620
Sum of electronic and zero-point Energies = -1754.647190
Sum of electronic and thermal Energies = -1754.605543
Sum of electronic and thermal Enthalpies = -1754.604599
Sum of electronic and thermal Free Energies = -1754.719112
E(CPCM(dioxane) M06L/def2-TZVP) = -3095.06095433

Int8

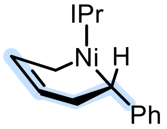

C	-0.60776	1.23470	-3.02592
H	-0.75135	2.31512	-2.84654
H	0.30847	1.09686	-3.62571
C	-1.79457	0.60511	-3.65844
H	-2.67191	1.21401	-3.88781
C	-1.77890	-0.71075	-3.92357
Ni	-0.28992	0.55206	-1.26155
C	-0.53986	-1.44503	-3.50559
H	-0.62735	-2.52899	-3.68476
H	0.33131	-1.10701	-4.09474
C	-0.21535	-1.21529	-2.01167
H	-1.01256	-1.68740	-1.40948
C	0.35706	0.92378	2.84549
C	-0.87009	0.34773	2.88048
H	0.96889	1.35313	3.62613
H	-1.56393	0.18672	3.69341
C	-0.16652	0.30120	0.70884
N	0.76684	0.88186	1.51893

N	-1.16122	-0.03771	1.57544
H	-2.60721	-1.23093	-4.40018
C	1.13007	-1.79509	-1.66845
C	1.25684	-3.14900	-1.31345
C	2.30613	-1.03170	-1.76934
C	2.50457	-3.71926	-1.05585
H	0.35732	-3.76124	-1.25399
C	3.55632	-1.59758	-1.51268
H	2.22189	0.02599	-2.02908
C	3.66146	-2.94356	-1.15230
H	2.57342	-4.76967	-0.77967
H	4.44915	-0.97988	-1.58628
H	4.63608	-3.38339	-0.95073
C	2.02187	1.33114	0.98979
C	2.00570	2.41625	0.09101
C	3.18394	0.61521	1.32507
C	3.22064	2.77242	-0.50245
C	0.73186	3.20915	-0.18730
C	4.37379	1.02692	0.71093
C	3.15348	-0.56224	2.30477
H	3.25618	3.59608	-1.20931
C	4.39052	2.08095	-0.19811
H	-0.09635	2.49897	-0.37526
C	0.32498	4.04268	1.03762
C	0.82714	4.10974	-1.41735
H	5.30080	0.50375	0.92259
H	2.84836	-0.16457	3.28592
C	4.53449	-1.19773	2.48980
C	2.14879	-1.66821	1.90386
H	5.32611	2.36868	-0.67196
H	1.10111	4.78629	1.26024
H	0.17852	3.42189	1.92851
H	-0.61356	4.57653	0.83718
H	1.16752	3.55511	-2.29928
H	1.51156	4.95027	-1.24182
H	-0.16277	4.52728	-1.63979
H	4.87053	-1.65546	1.54918
H	4.47168	-1.99035	3.24324
H	5.29117	-0.47749	2.82161
H	2.05741	-1.74212	0.81679
H	1.14944	-1.50067	2.32215
H	2.49949	-2.63789	2.27853
C	-2.45747	-0.45901	1.11836
C	-2.71862	-1.82952	0.95156
C	-3.38938	0.54347	0.81378
C	-3.99752	-2.18492	0.51377
C	-1.63682	-2.86442	1.22243
C	-4.65715	0.13782	0.38124
C	-3.04279	2.02218	0.88855
H	-4.24346	-3.23310	0.36667
C	-4.96089	-1.21258	0.24273
H	-0.68262	-2.43891	0.87438
C	-1.51130	-3.15535	2.72471
C	-1.86214	-4.17257	0.46199
H	-5.40415	0.89123	0.13640
H	-2.00307	2.12184	1.22898
C	-3.93258	2.76190	1.89297
C	-3.13078	2.65900	-0.50482
H	-5.94864	-1.51361	-0.09817
H	-2.45086	-3.57238	3.11034
H	-1.27755	-2.25163	3.29865
H	-0.71189	-3.88469	2.90551
H	-2.02446	-3.99280	-0.60826
H	-2.72763	-4.72270	0.85433
H	-0.98481	-4.82023	0.57510
H	-4.98815	2.70751	1.59675
H	-3.65209	3.82136	1.94269
H	-3.84101	2.33285	2.89804
H	-4.16930	2.68361	-0.86090
H	-2.53871	2.08756	-1.23237
H	-2.75751	3.69256	-0.47894

Zero-point correction = 0.773193 (Hartree/Particle)
Thermal correction to Energy = 0.814590
Thermal correction to Enthalpy = 0.815534
Thermal correction to Gibbs Free Energy = 0.702298
Sum of electronic and zero-point Energies = -1754.669571
Sum of electronic and thermal Energies = -1754.628174
Sum of electronic and thermal Enthalpies = -1754.627230
Sum of electronic and thermal Free Energies = -1754.740466
E(CPCM(dioxane) M06L/def2-TZVP) = -3095.07759719

TS7

C	-0.86857	-1.68497	-1.70664
H	0.16826	-1.91481	-1.36015
H	-1.50569	-2.51862	-1.38659
C	-0.90136	-1.44453	-3.18057
H	-0.32801	-2.10017	-3.83856
C	-1.62603	-0.42997	-3.66215
Ni	-0.45960	-0.21067	-0.47663
C	-2.35300	0.41216	-2.65039
H	-1.92960	1.42818	-2.62144
H	-3.41281	0.52693	-2.92966
C	-2.24552	-0.23763	-1.27021
H	-2.13468	0.56779	-0.48452
H	-1.69375	-0.20107	-4.72523
C	1.04288	0.45740	0.52250
N	1.24297	1.73736	0.95832
N	2.14314	-0.20262	0.99196
C	2.43208	1.87153	1.66592
C	0.32525	2.78258	0.60583
C	2.99863	0.63989	1.69452
C	2.34014	-1.60679	0.76699
H	2.75576	2.81817	2.07442
C	-0.65944	3.15720	1.53574
C	0.40155	3.32294	-0.68689
H	3.91623	0.28397	2.14061
C	3.18990	-2.00389	-0.28068
C	1.62816	-2.52238	1.55693
C	-1.60918	4.09730	1.12638
C	-0.73169	2.50132	2.90612
C	-0.57422	4.25840	-1.05227
C	1.49936	2.94647	-1.66997
C	3.32845	-3.37442	-0.51800
C	3.84717	-0.98062	-1.19314
C	1.79573	-3.88403	1.27780
C	0.66183	-2.08753	2.64603
H	-2.39180	4.40607	1.81520
C	-1.57258	4.63680	-0.15923
H	0.28422	2.19031	3.18632
C	-1.25004	3.44286	3.99649
C	-1.60210	1.23807	2.83269
H	-0.54693	4.69398	-2.04987
H	2.15013	2.19750	-1.19747
C	2.35583	4.17391	-2.00869
C	0.92234	2.31674	-2.94220
H	3.97300	-3.71676	-1.32419
C	2.63865	-4.30750	0.25488
H	3.96287	-0.04306	-0.63214
C	5.23435	-1.40828	-1.68019
C	2.92088	-0.70037	-2.38652
H	1.24877	-4.61818	1.86686
H	0.72495	-0.99602	2.75051
C	1.01246	-2.71438	4.00047
C	-0.77498	-2.43576	2.23560
H	-2.32476	5.36135	-0.46249
H	-2.31479	3.66789	3.85585
H	-0.70006	4.39106	4.01147
H	-1.14815	2.96732	4.97885
H	-1.21819	0.53826	2.07797
H	-2.62926	1.50775	2.54895
H	-1.63341	0.72932	3.80581
H	1.75592	4.94130	-2.51498
H	3.17581	3.89349	-2.68092
H	2.78590	4.62437	-1.10636
H	0.22898	3.00565	-3.44390
H	0.38341	1.38940	-2.70851
H	1.72923	2.07866	-3.64725
H	2.75443	-5.36967	0.05207
H	5.17042	-2.25359	-2.37695
H	5.88613	-1.70072	-0.84865
H	5.71092	-0.58173	-2.21993
H	1.93702	-0.34585	-2.05152
H	2.76882	-1.62100	-2.96751

H	3.36076	0.05705	-3.04918
H	0.92323	-3.80770	3.96555
H	0.32438	-2.35175	4.77370
H	2.03622	-2.46885	4.30585
H	-0.90411	-3.52370	2.15557
H	-1.01675	-1.98802	1.26013
H	-1.49273	-2.06013	2.97725
C	-3.38607	-1.11509	-0.86112
C	-3.75521	-1.20823	0.49001
C	-4.10644	-1.87752	-1.79445
C	-4.80269	-2.03237	0.89687
H	-3.20666	-0.61825	1.22426
C	-5.15862	-2.69875	-1.39035
H	-3.82228	-1.83562	-2.84531
C	-5.51279	-2.78202	-0.04298
H	-5.07059	-2.08331	1.95033
H	-5.70195	-3.28006	-2.13260
H	-6.33330	-3.42291	0.27124

Zero-point correction = 0.770949 (Hartree/Particle)
Thermal correction to Energy = 0.812896
Thermal correction to Enthalpy = 0.813840
Thermal correction to Gibbs Free Energy = 0.695549
Sum of electronic and zero-point Energies = -1754.668816
Sum of electronic and thermal Energies = -1754.626869
Sum of electronic and thermal Enthalpies = -1754.625925
Sum of electronic and thermal Free Energies = -1754.744215
E(CPCM(dioxane) M06L/def2-TZVP) = -3095.06424229

Int9

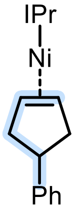

C	-2.12352	-1.34403	-2.32643
H	-1.76303	-2.33860	-2.03119
H	-2.81280	-1.48773	-3.17625
C	-1.02320	-0.36660	-2.69303
H	-0.32260	-0.57265	-3.50280
C	-1.33563	0.89842	-2.21843
Ni	0.07153	0.08279	-1.05703
C	-2.65206	0.87479	-1.47272
H	-2.65277	1.49122	-0.56247
H	-3.46822	1.24266	-2.11828
C	-2.84135	-0.62667	-1.16010
H	-2.24817	-0.83473	-0.25786
H	-0.92405	1.83570	-2.59463
C	1.26168	0.23727	0.43991
N	2.25441	-0.52716	0.98175
N	1.26195	1.35072	1.23725
C	2.85614	0.09371	2.07378
C	2.61007	-1.82832	0.49100
C	2.23401	1.28727	2.22876
C	0.39888	2.46102	0.94916
H	3.65901	-0.37442	2.62494
C	3.68542	-1.93427	-0.40796
C	1.86444	-2.93589	0.92169
H	2.38588	2.08846	2.93809
C	-0.77601	2.62042	1.70528
C	0.74463	3.30834	-0.11562
C	4.01333	-3.21010	-0.87515
C	4.40442	-0.69644	-0.91909
C	2.22640	-4.19233	0.42213
C	0.66754	-2.79455	1.84716
C	-1.64128	3.65618	1.34118
C	-1.11467	1.65868	2.83501
C	-0.15777	4.32622	-0.44670
C	2.06984	3.19371	-0.85527
H	4.83647	-3.32986	-1.57572
C	3.29164	-4.32962	-0.46305
H	4.29431	0.09714	-0.16686
C	5.90183	-0.92006	-1.14768
C	3.72930	-0.21462	-2.21226
H	1.66009	-5.07046	0.72739
H	0.63902	-1.76218	2.22107
C	0.75593	-3.73206	3.05616
C	-0.62281	-3.04072	1.05457
H	-2.56237	3.80903	1.89802
C	-1.34171	4.49308	0.26619
H	-0.17018	1.33344	3.29318
C	-1.97103	2.29414	3.93287
C	-1.80968	0.40979	2.27326
H	0.07829	5.00105	-1.26794
H	2.58905	2.29111	-0.50506
C	2.94927	4.40830	-0.52642
C	1.88330	3.05085	-2.36890
H	3.55916	-5.31399	-0.84018
H	6.08024	-1.59898	-1.99111
H	6.39103	-1.34140	-0.26172
H	6.38777	0.03170	-1.39201
H	2.65668	-0.02996	-2.05543
H	3.83294	-0.97909	-2.99442
H	4.19794	0.71166	-2.57101
H	0.73006	-4.78451	2.74647
H	-0.09712	-3.56547	3.72490
H	1.67831	-3.56995	3.62567
H	-0.66291	-4.07866	0.69682
H	-0.66739	-2.37342	0.18302
H	-1.50640	-2.85953	1.68205
H	-2.03134	5.28827	-0.00730
H	-2.98867	2.49676	3.57585
H	-1.54086	3.23603	4.29319
H	-2.05809	1.60582	4.78141
H	-1.19424	-0.06490	1.49787
H	-2.77233	0.68224	1.81715
H	-2.00195	-0.32079	3.07142
H	2.47647	5.33555	-0.87593
H	3.92547	4.32088	-1.01886
H	3.11285	4.50105	0.55404
H	1.33344	3.90384	-2.78813
H	1.33480	2.12989	-2.60232
H	2.86091	3.00685	-2.86571
C	-4.26346	-1.05736	-0.89662
C	-5.26042	-0.92022	-1.87289
C	-4.62076	-1.60201	0.34198
C	-6.57212	-1.31535	-1.61820
H	-5.00120	-0.49805	-2.84361
C	-5.93323	-1.99937	0.60366
H	-3.85288	-1.71479	1.10840
C	-6.91425	-1.85689	-0.37674
H	-7.33070	-1.20092	-2.38961
H	-6.18803	-2.42091	1.57370
H	-7.93792	-2.16546	-0.17751

Zero-point correction = 0.775110 (Hartree/Particle)
Thermal correction to Energy = 0.817121
Thermal correction to Enthalpy = 0.818066
Thermal correction to Gibbs Free Energy = 0.699578
Sum of electronic and zero-point Energies = -1754.726551
Sum of electronic and thermal Energies = -1754.684539
Sum of electronic and thermal Enthalpies = -1754.683595
Sum of electronic and thermal Free Energies = -1754.802082
E(CPCM(dioxane) M06L/def2-TZVP) = -3095.12088741

13. Chiral HPLC and GC Analyses

13.1. Chiral analysis of enantiopure cyclopropanes

Racemic ((*cis*)-2-(4-methoxyphenyl)cyclopropyl)methanol (*rac*-cis)

The compound was obtained as racemate ($\tau_{(S,R)}$ = 16.2 min, $\tau_{(R,S)}$ = 17.6 min).

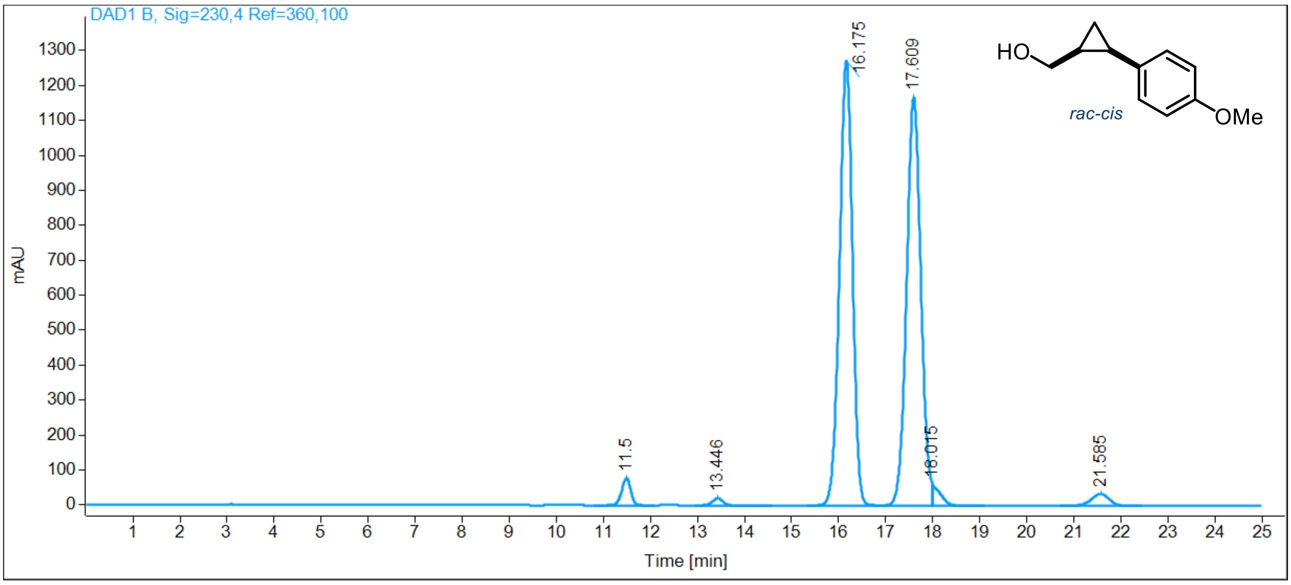

Mobile phase: 95:5 *n*-hexane/EtOH. **Column**: Chiralpak IA, (250 x 4,6) mm. **Pressure at start**: 38 bar. **Start flow**: 1.000 mL/min. **Column oven**: 30 °C.

((1*S*,2*R*)-2-(4-methoxyphenyl)cyclopropyl)methanol (S57)

The compound was obtained with 99.8% ee (major: $\tau_{(S,R)}$ = 16.1 min, minor: $\tau_{(R,S)}$ = 17.4 min).

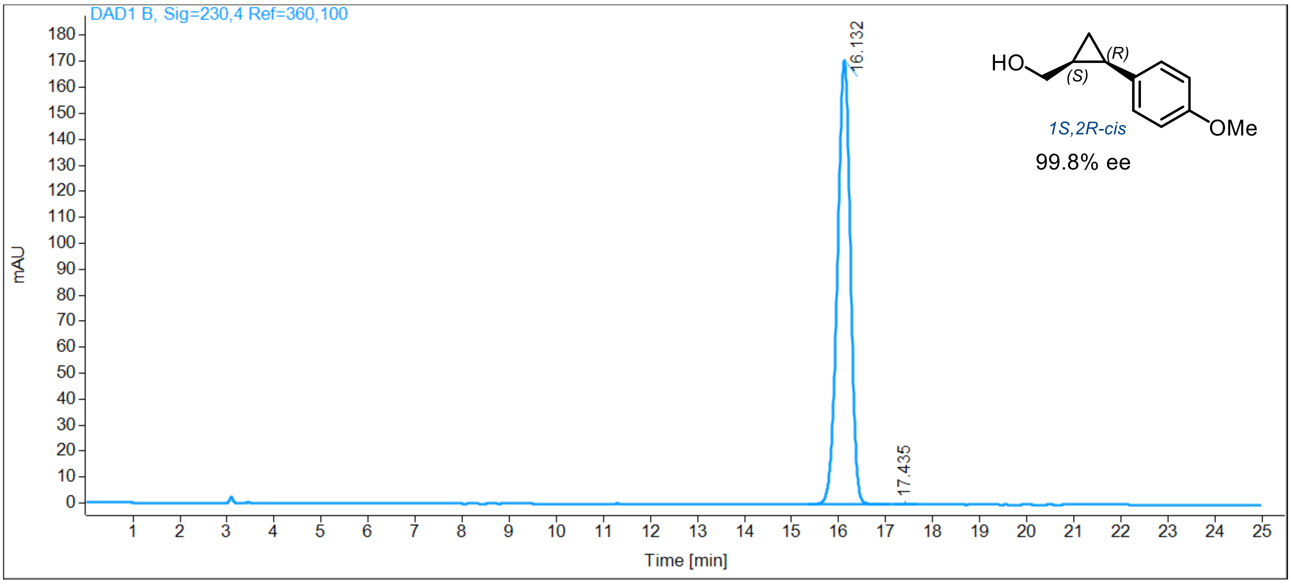

Mobile phase: 95:5 *n*-hexane/EtOH. **Column**: Chiralpak IA, (250 x 4,6) mm. **Pressure at start**: 37 bar. **Start flow**: 1.000 mL/min. **Column oven**: 30 °C.

((1*R*,2*S*)-2-(4-methoxyphenyl)cyclopropyl)methanol (S58)

The compound was obtained with 98.8% ee (minor: $\tau_{(S,R)}$ = 16.1 min, major: $\tau_{(R,S)}$ = 17.5 min).

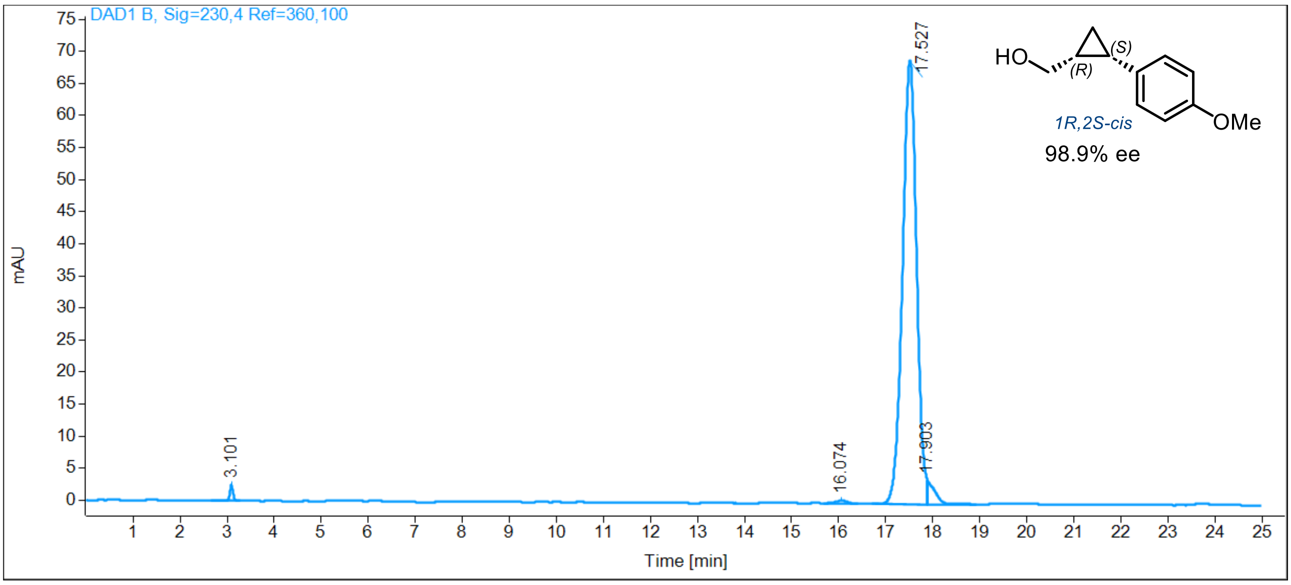

Mobile phase: 95:5 *n*-hexane/EtOH. **Column**: Chiralpak IA, (250 x 4,6) mm. **Pressure at start**: 38 bar. **Start flow**: 1.000 mL/min. **Column oven**: 30 °C.

Racemic ((*trans*)-2-(4-methoxyphenyl)cyclopropyl)methanol (*rac*-trans)

The compound was obtained as racemate ($\tau_{(S,S)}$ = 14.3 min, $\tau_{(R,R)}$ = 15.0 min).

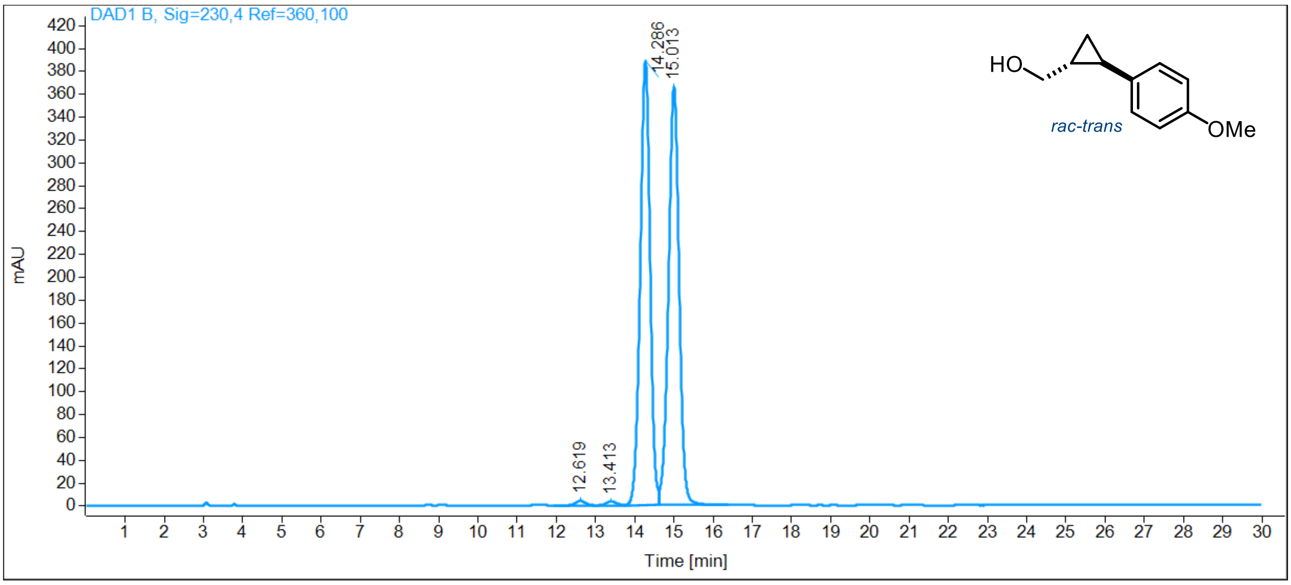

Mobile phase: 95:5 *n*-hexane/*i*-PrOH. **Column**: Chiralpak IA, (250 x 4,6) mm. **Pressure at start**: 38 bar. **Start flow**: 1.000 mL/min. **Column oven**: 29.99 °C.

((1*S*,2*S*)-2-(4-methoxyphenyl)cyclopropyl)methanol (S60)

The compound was obtained with >99.9% ee (major: $\tau_{(S,S)}$ = 14.3 min, $\tau_{(R,R)}$ = not detected).

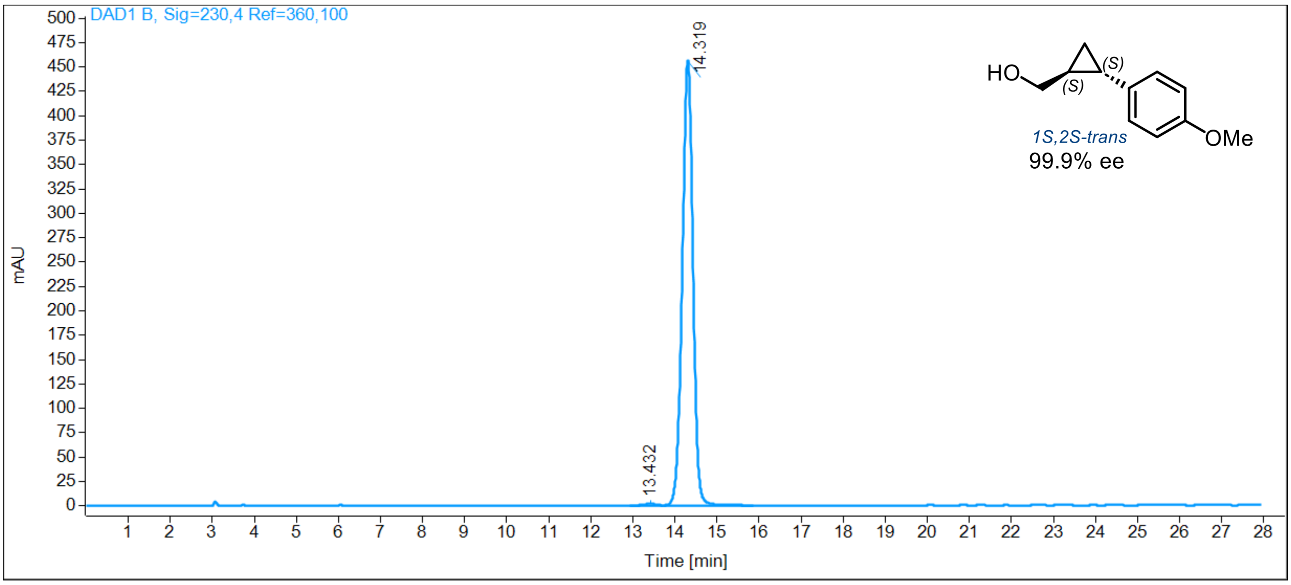

Mobile phase: 95:5 *n*-hexane/*i*-PrOH. **Column**: Chiralpak IA, (250 x 4,6) mm. **Pressure at start**: 38 bar. **Start flow**: 1.000 mL/min. **Column oven**: 30.01 °C.

((1*R*,2*R*)-2-(4-methoxyphenyl)cyclopropyl)methanol (S61)

The compound was obtained with 99.8% ee (minor: $\tau_{(S,S)}$ = 14.3 min, major: $\tau_{(R,R)}$ = 15.1 min).

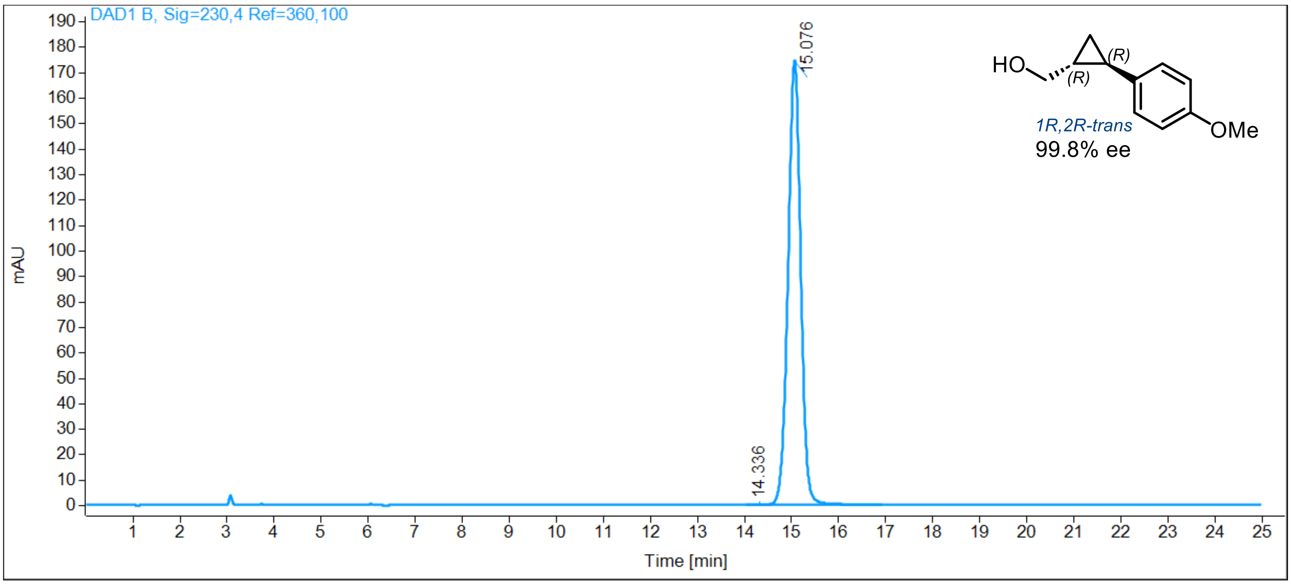

Mobile phase: 95:5 *n*-hexane/*i*-PrOH. **Column**: Chiralpak IA, (250 x 4,6) mm. **Pressure at start**: 38 bar. **Start flow**: 1.000 mL/min. **Column oven**: 29.99 °C.

Racemic 1-methoxy-4-((*cis*)-2-vinylcyclopropyl)benzene (*rac-cis*-2)

The compound was obtained as racemate ($\tau_{(S,S)}$ = 5.3 min, $\tau_{(R,R)}$ = 5.7 min).

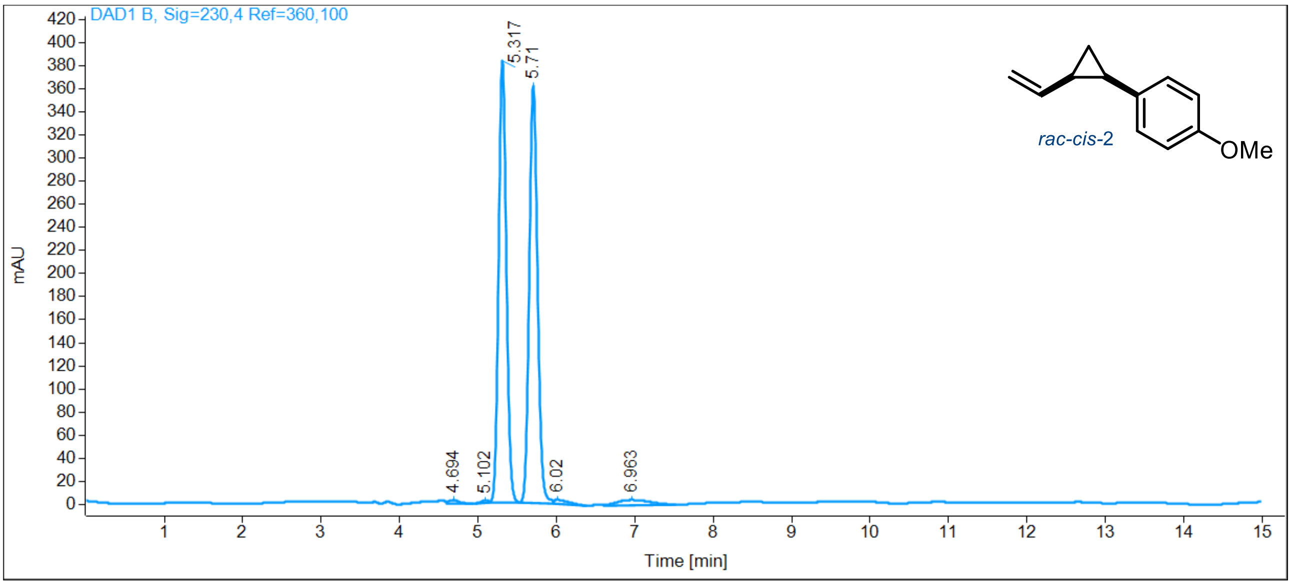

Mobile phase: 99:1 *n*-hexane/EtOH. **Column**: Chiralpak IG, (150 x 4,6) mm, 5 µm. **Pressure at start**: 13 bar. **Start flow**: 0.500 mL/min. **Column oven**: 19.99 °C.

1-methoxy-4-((1*R*,2*R*)-2-vinylcyclopropyl)benzene (*cis*-2)

The compound was obtained with 99.9% ee (minor: $\tau_{(S,S)}$ = 5.35 min, major: $\tau_{(R,R)}$ = 5.75 min).

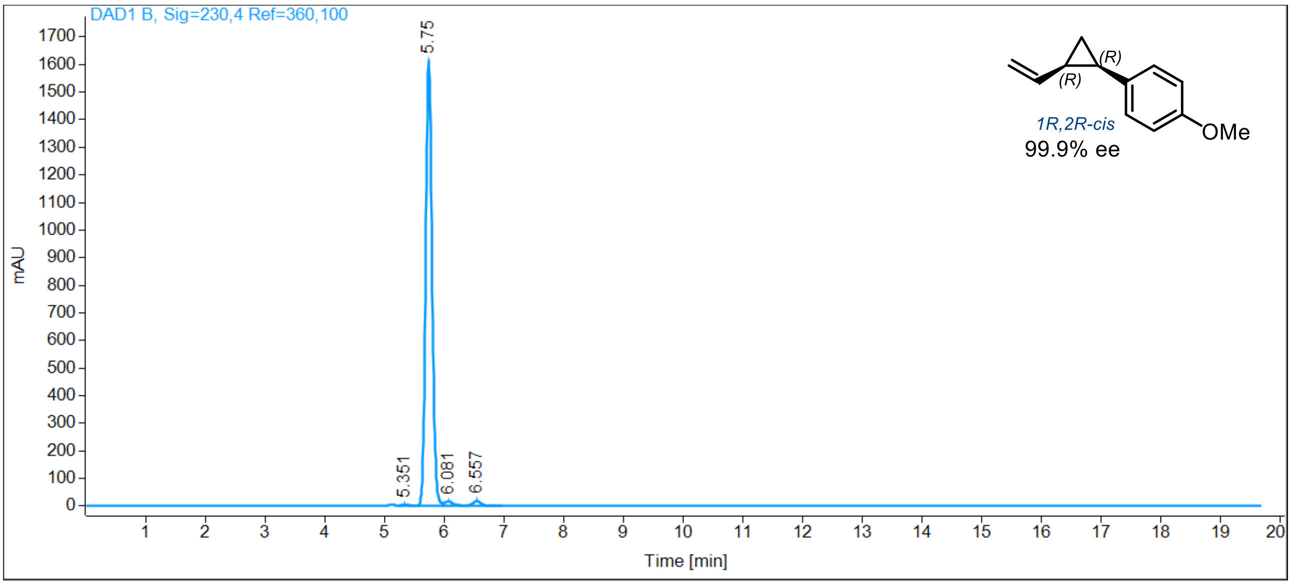

Mobile phase: 99:1 *n*-hexane/EtOH. **Column**: Chiralpak IG, (150 x 4,6) mm, 5 µm. **Pressure at start**: 13 bar. **Start flow**: 0.500 mL/min. **Column oven**: 19.99 °C.

Racemic 1-methoxy-4-((*trans*)-2-vinylcyclopropyl)benzene (*rac-trans*-2)

The compound was obtained as racemate ($\tau_{(S,S)}$ = 6.03 min, $\tau_{(R,R)}$ = 6.50 min).

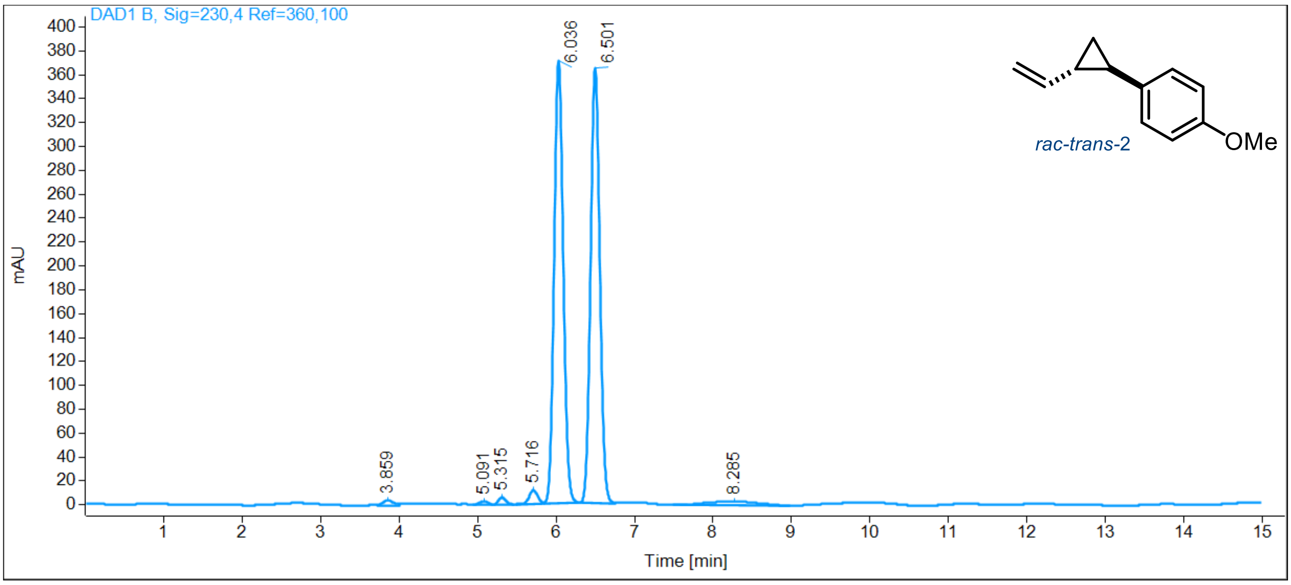

Mobile phase: 99:1 *n*-hexane/EtOH. **Column**: Chiralpak IG, (150 x 4,6) mm, 5 µm. **Pressure at start**: 13 bar. **Start flow**: 0.500 mL/min. **Column oven**: 20 °C.

Isomerization to 1-methoxy-4-((1*S*,2*R*)-2-vinylcyclopropyl)benzene (*trans*-2)

Starting material: 1-methoxy-4-((1*R*,2*R*)-2-vinylcyclopropyl)benzene. **Conditions:** [Ni(µ-Cl)(IPr)]$_2$ (1 mol%), room temperature., 5 minutes.

The *trans* isomer was obtained with 98.8% ee (major: $\tau_{(S,R)}$ = 6.061 min, minor: $\tau_{(R,S)}$ = 6.529 min). The *cis* isomer was obtained with 99.9% ee (minor: $\tau_{(S,S)}$ = not detected, major: $\tau_{(R,R)}$ = 5.747 min).

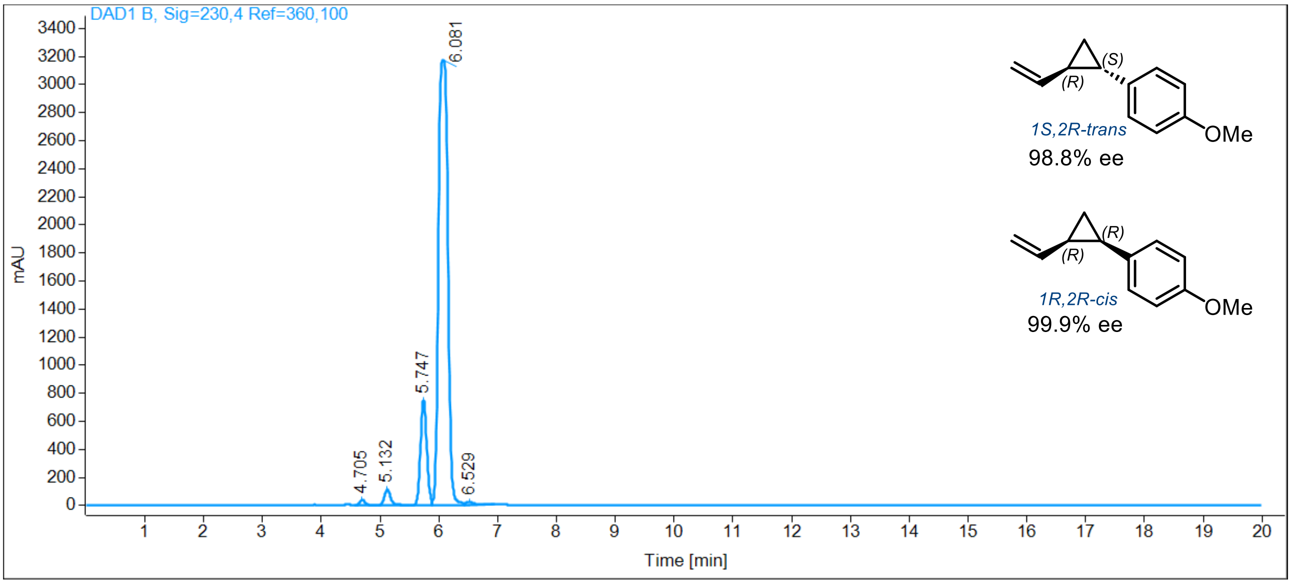

Mobile phase: 99:1 *n*-hexane/EtOH. **Column**: Chiralpak IG, (150 x 4,6) mm, 5 µm. **Pressure at start**: 13 bar. **Start flow**: 0.500 mL/min. **Column oven**: 20 °C.

13.2. Chiral analysis of *tert*-butyl-1-((*tert*-butoxycarbonyl)amino)-2-vinylcyclopropane-1-carboxylate

tert-butyl (1*RS*,2*SR*)-1-((*tert*-butoxycarbonyl)amino)-2-vinylcyclopropane-1-carboxylate (*rac*-37)

The compound was obtained as racemate ($\tau_{(S,R)}$ = 35.63 min, $\tau_{(R,S)}$ = 37.66 min).

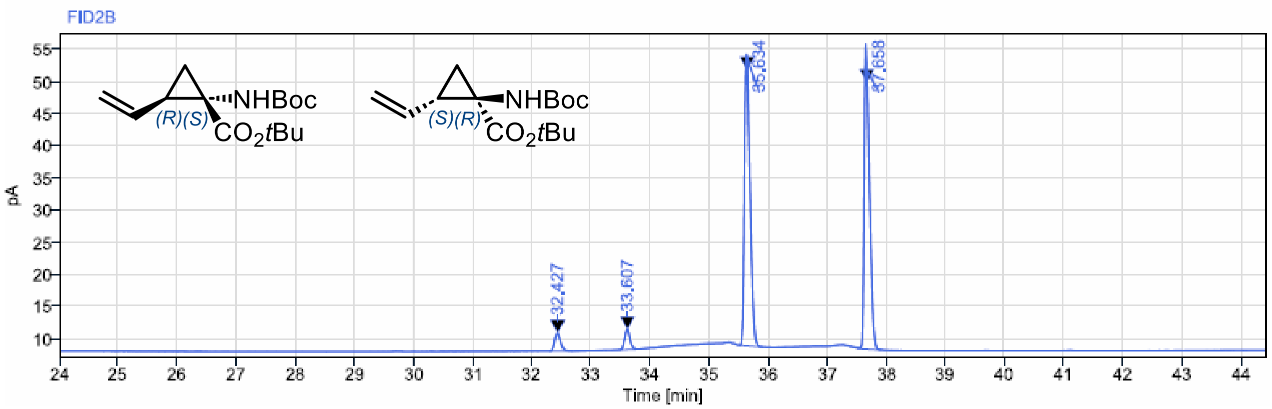

Instrument: 8890 GC. **Mobile phase**: helium. **Column**: Chirasil-dex, 25 m, 0.25 mm. **Pressure**: 0.8 bar. **Method:** 120 °C–10 min, isothermal; 1 °C/min-140 °C; 3 °C/min-190 °C–30 min, isothermal.

tert-butyl (1*R*,2*S*)-1-((*tert*-butoxycarbonyl)amino)-2-vinylcyclopropane-1-carboxylate (37)

The compound was obtained with >99% ee (minor: $\tau_{(S,R)}$ = 35.72 min, major: $\tau_{(R,S)}$ = 37.56 min).

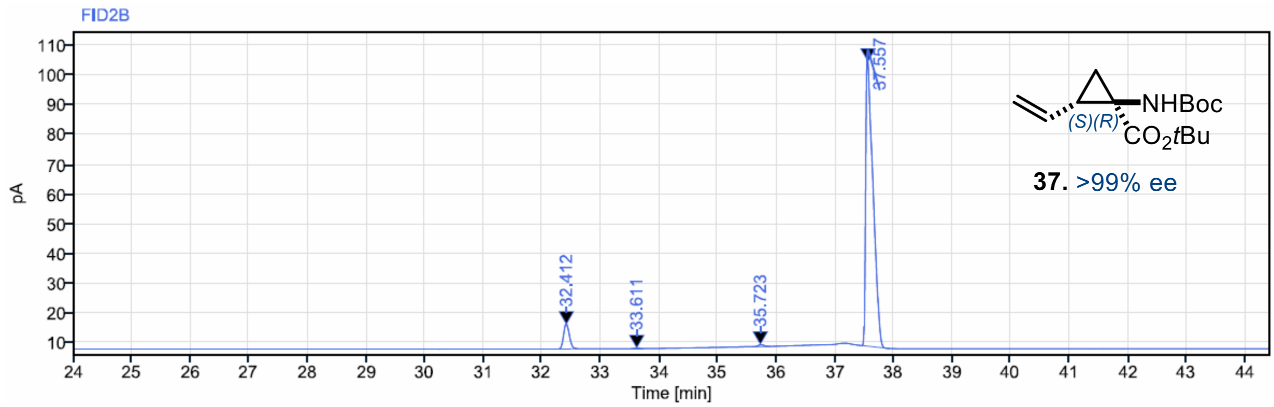

Instrument: 8890 GC. **Mobile phase**: helium. **Column**: Chirasil-dex, 25 m, 0.25 mm. **Pressure**: 0.8 bar. **Method:** 120 °C–10 min, isothermal; 1 °C/min-140 °C; 3 °C/min-190 °C–30 min, isothermal.

tert-butyl (1SR,2SR)-1-((tert-butoxycarbonyl)amino)-2-vinylcyclopropane-1-carboxylate (rac-38)

The compound was obtained as racemate ($\tau_{(R,R)}$ = 32.31 min, $\tau_{(S,S)}$ = 33.44 min) after isomerization of *rac*-**37**.

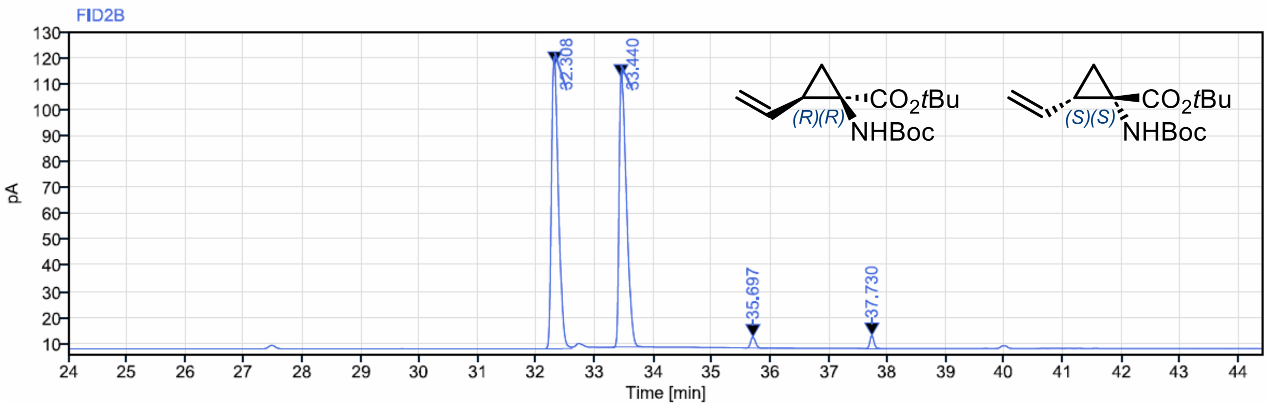

Instrument: 8890 GC. **Mobile phase**: helium. **Column**: Chirasil-dex, 25 m, 0.25 mm. **Pressure**: 0.8 bar. **Method:** 120 °C–10 min, isothermal; 1 °C/min-140 °C; 3 °C/min-190 °C–30 min, isothermal.

Isomerization to tert-butyl (1S,2S)-1-((tert-butoxycarbonyl)amino)-2-vinylcyclopropane-1-carboxylate (38)

Starting material: 1*R*,2*S*-enantiomer (**37**). **Conditions:** [Ni(μ-Cl)(IPr)]$_2$ (1 mol%), r.t., 10 min. **Diastereomer separation**: flash silica column chromatography. **Iterations**: 3 cycles.

The compound was obtained with 99% ee (minor: $\tau_{(R,R)}$ = 32.41 min, major: $\tau_{(S,S)}$ = 33.47 min).

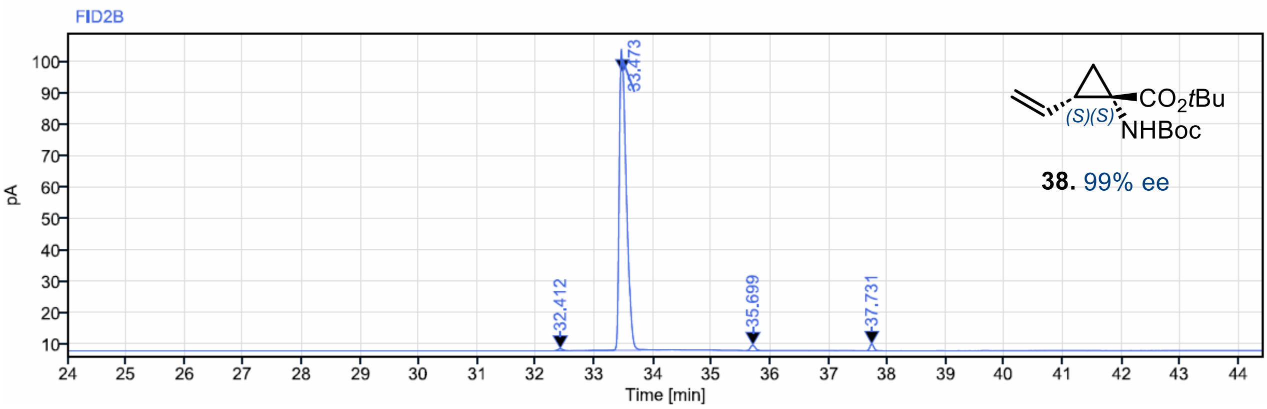

Instrument: 8890 GC. **Mobile phase**: helium. **Column**: Chirasil-dex, 25 m, 0.25 mm. **Pressure**: 0.8 bar. **Method:** 120 °C–10 min, isothermal; 1 °C/min-140 °C; 3 °C/min-190 °C–30 min, isothermal.

Isomerization to *tert*-butyl (1*S*,2*S*)-1-((*tert*-butoxycarbonyl)amino)-2-vinylcyclopropane-1-carboxylate (38)

Starting material: 1*R*,2*S*-enantiomer (**37**). **Conditions:** [Ni(μ-Cl)(IPr)]$_2$ (1 mol%), r.t., 10 min. **Diastereomer separation**: Crystallization. **Iterations**: 3 cycles.

The compound was obtained with >99% ee (minor: $\tau_{(R,R)}$ = 32.42 min, major: $\tau_{(S,S)}$ = 33.43 min).

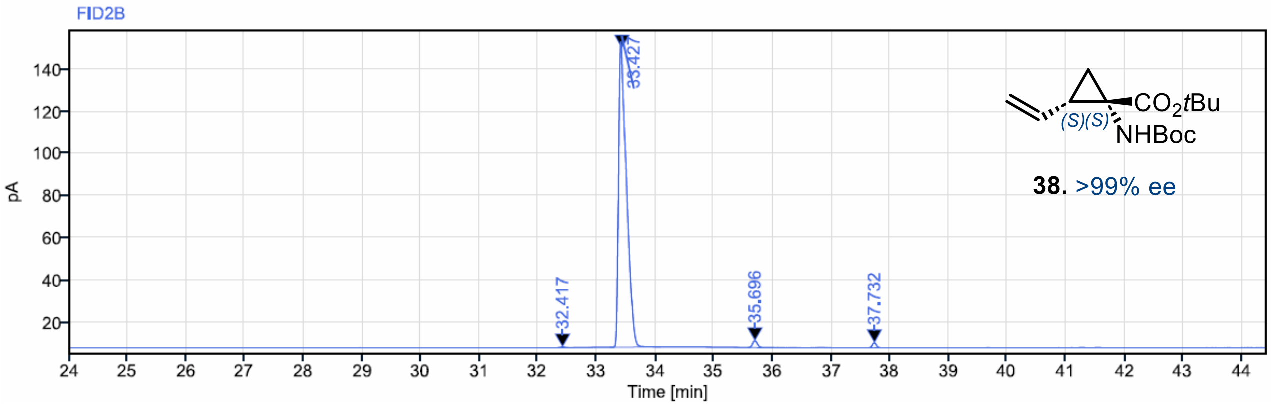

Instrument: 8890 GC. **Mobile phase**: helium. **Column**: Chirasil-dex, 25 m, 0.25 mm. **Pressure**: 0.8 bar. **Method:** 120 °C–10 min, isothermal; 1 °C/min-140 °C; 3 °C/min-190 °C–30 min, isothermal.

13.3. Chiral analysis of dictyopterenes

(*rac*)-Dictyopterene A (*rac*-39)

The compound was obtained as racemate ($\tau_{(R,R)}$ = 42.3 min, $\tau_{(S,S)}$ = 42.8 min).

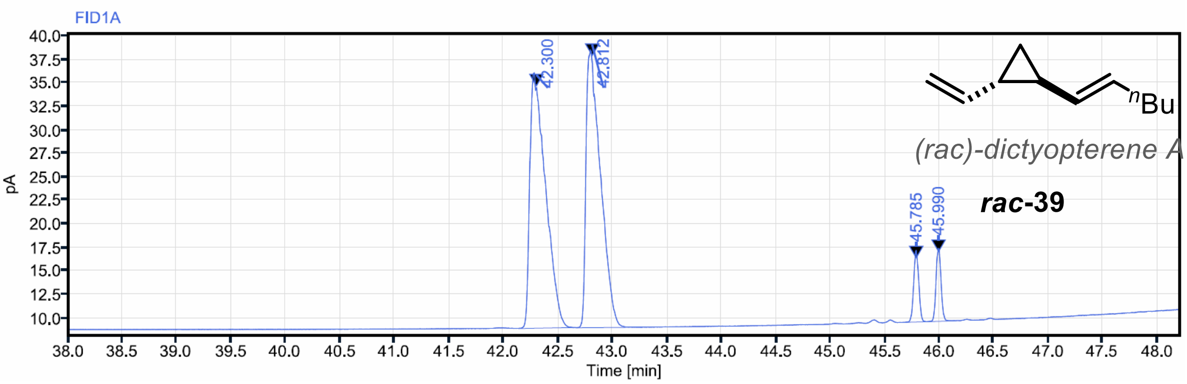

Instrument: 8890 GC. **Mobile phase**: helium. **Column**: Hydrodex-β-6TBDM, 25 m, 0.25 mm. **Pressure**: 0.8 bar. **Method:** 70 °C–40 min, isothermal; 10 °C/min-190 °C–10 min.

Note: Thermal isomerization and Cope rearrangement to the cyclized product occurred under the same analysis conditions (τ = 45.8 min, τ = 46.0 min).

(-)-Dictyopterene A (39)

The compound was obtained with 88% ee (minor: $\tau_{(R,R)}$ = 42.4 min, major: $\tau_{(S,S)}$ = 42.7 min).

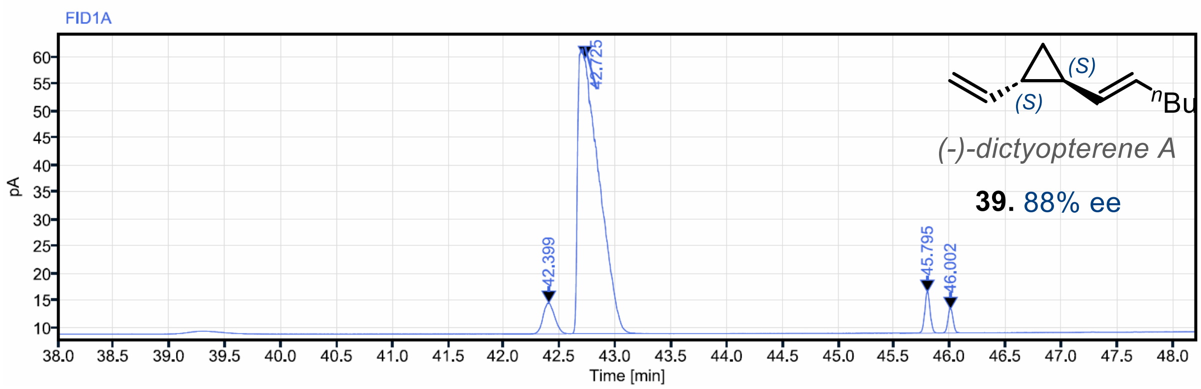

Instrument: 8890 GC. **Mobile phase**: helium. **Column**: Hydrodex-β-6TBDM, 25 m, 0.25 mm. **Pressure**: 0.8 bar. **Method:** 70 °C–40 min, isothermal; 10 °C/min-190 °C–10 min.

(*rac*)-Dictyopterene C' (*rac*-40)

The compound was obtained as racemate ($\tau_{(R)}$ = 45.73 min, $\tau_{(S)}$ = 45.95 min).

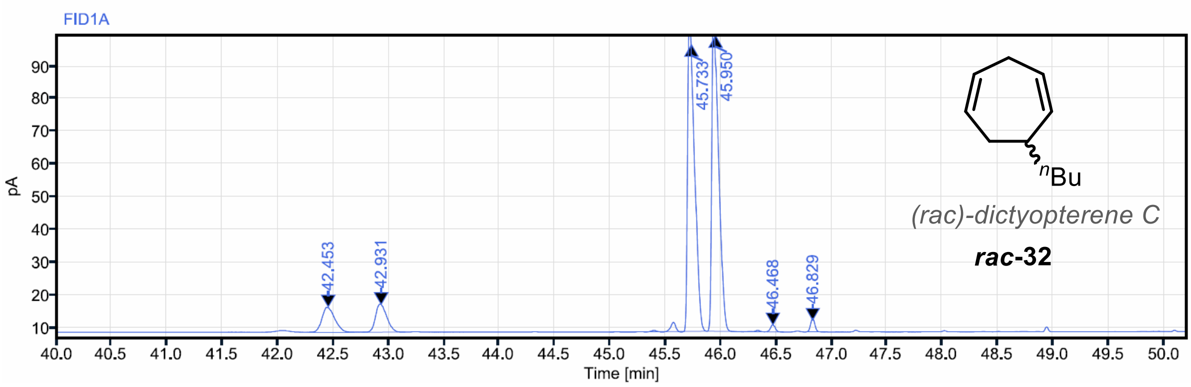

Instrument: 8890 GC. **Mobile phase**: helium. **Column**: Hydrodex-β-6TBDM, 25 m, 0.25 mm. **Pressure**: 0.8 bar. **Method:** 70 °C–40 min, isothermal; 10 °C/min-190 °C–10 min.

Isomerization/Cope to form (+)-Dictyopterene C' (40)

Starting material: (-)-dictyopterene A. **Conditions:** [Ni(μ-Cl)(IPr)]$_2$ (5 mol%), 45 °C, 24 h.

The compound was obtained with 86% ee (minor: $\tau_{(R)}$ = 45.73 min, major: $\tau_{(S)}$ = 45.95 min).

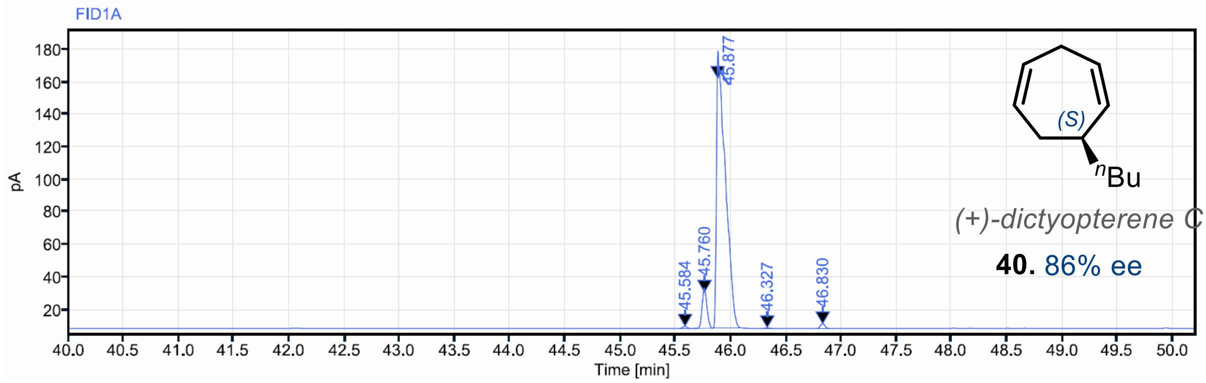

Instrument: 8890 GC. **Mobile phase**: helium. **Column**: Hydrodex-β-6TBDM, 25 m, 0.25 mm. **Pressure**: 0.8 bar. **Method:** 70 °C–40 min, isothermal; 10 °C/min-190 °C–10 min.

Thermal isomerization/Cope to form (-)-Dictyopterene C' (*ent*-40)

Starting material: (-)-dictyopterene A. **Conditions:** 165 °C, 48 h.

The compound was obtained with 10% ee (major: $\tau_{(R)}$ = 45.70 min, minor: $\tau_{(S)}$ = 45.92 min).

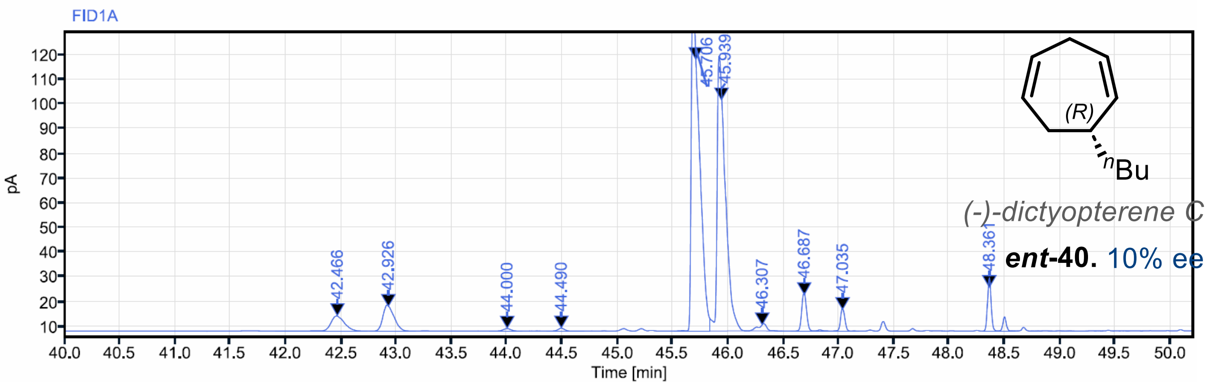

Instrument: 8890 GC. **Mobile phase**: helium. **Column**: Hydrodex-β-6TBDM, 25 m, 0.25 mm. **Pressure**: 0.8 bar. **Method:** 70 °C–40 min, isothermal; 10 °C/min-190 °C–10 min.

14. NMR Spectra

14.1. NMR spectra of vinylcyclopropanes and isomerized *trans*-products

1-methoxy-4-(2-vinylcyclopropyl)benzene (S2)

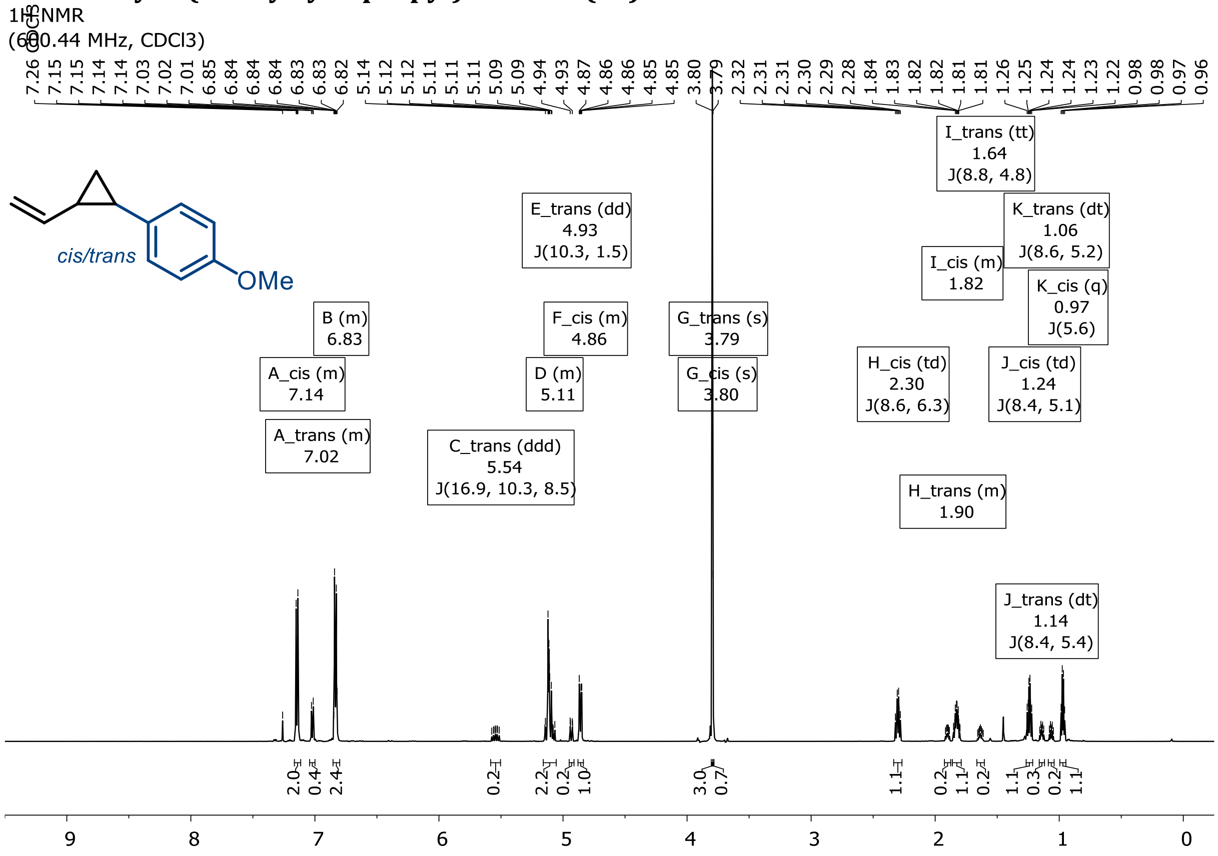

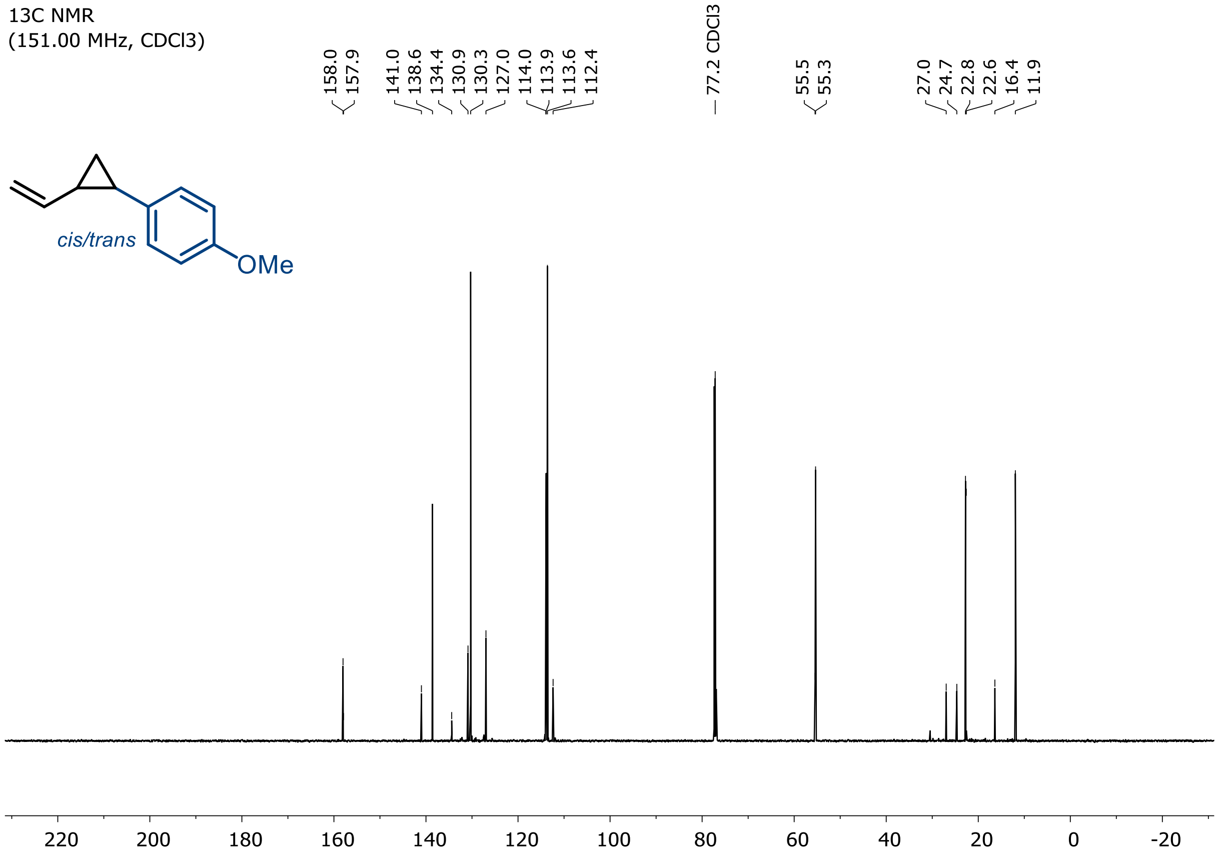

trans-1-methoxy-4-(2-vinylcyclopropyl)benzene (2)

1H NMR
(600.44 MHz, CDCl3)

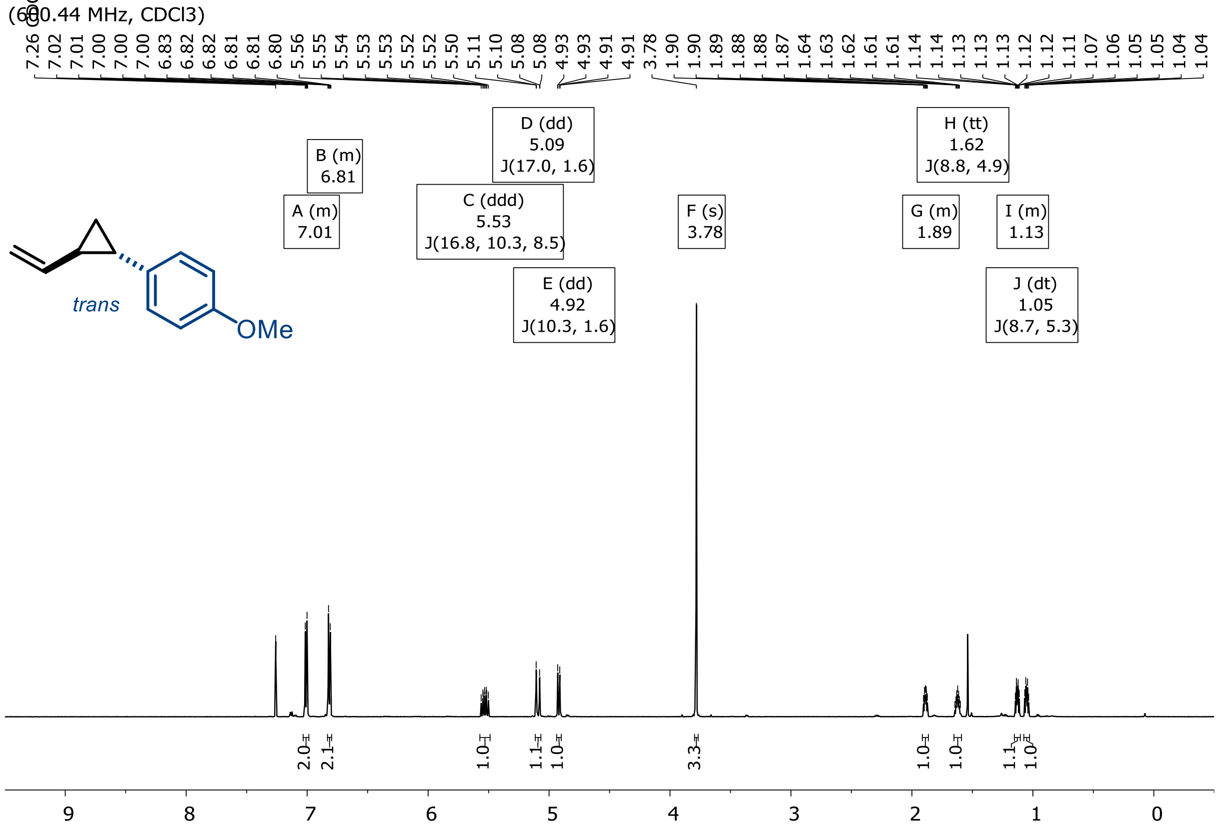

13C NMR
(151.00 MHz, CDCl3)

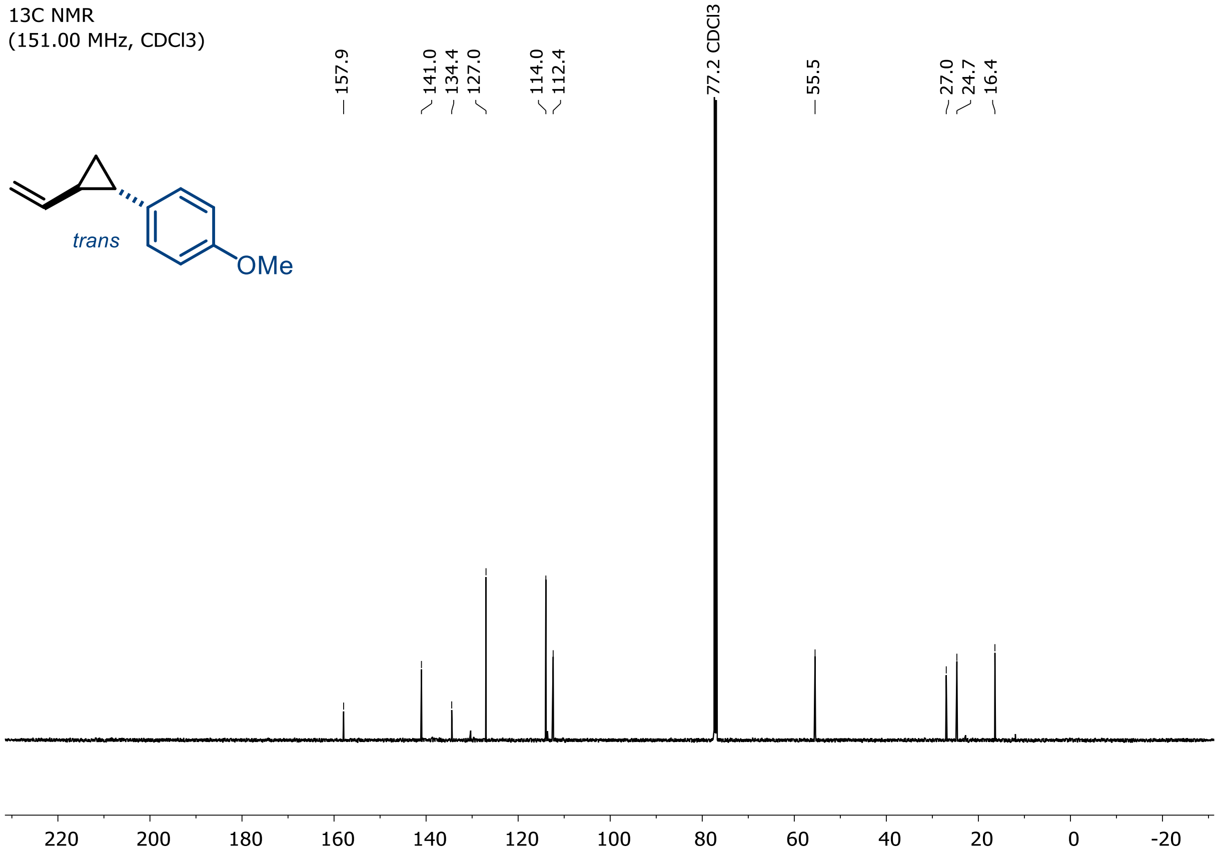

(2-vinylcyclopropyl)benzene (S4)

1H NMR
(599.86 MHz, CDCl3)

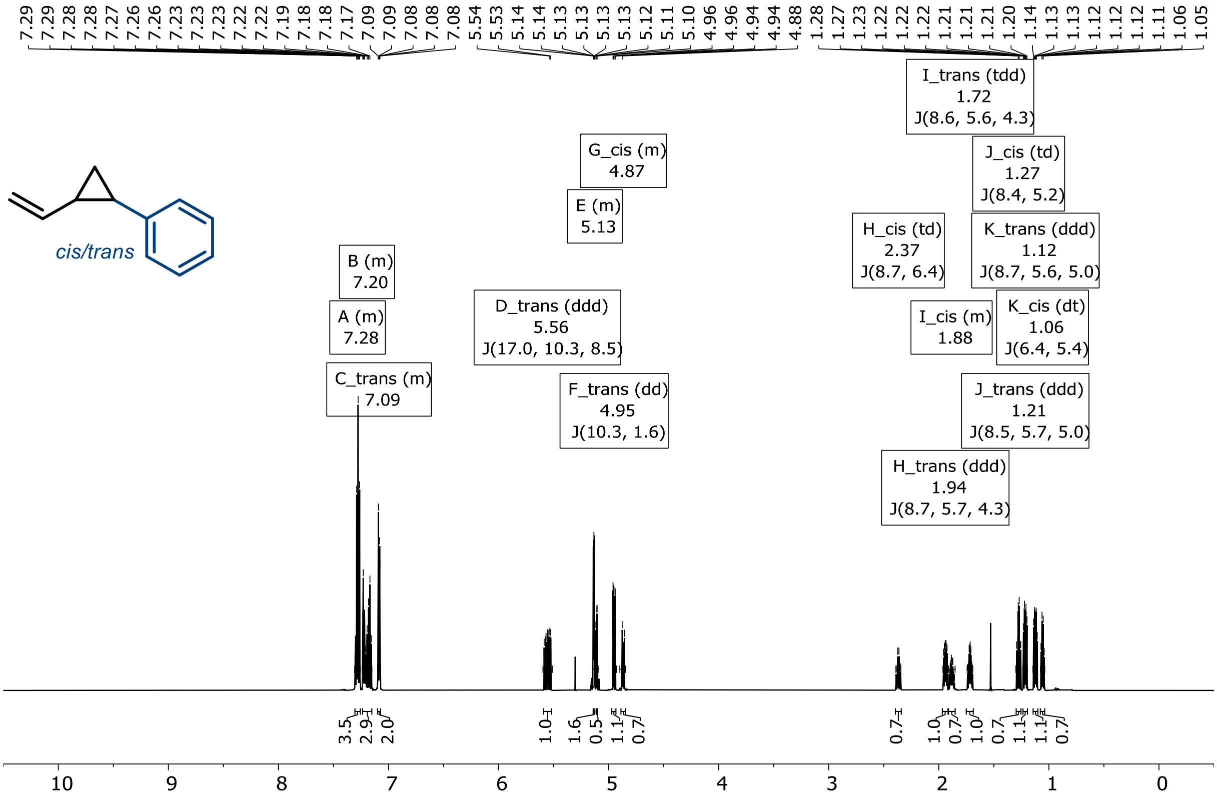

13C NMR
(150.85 MHz, CDCl3)

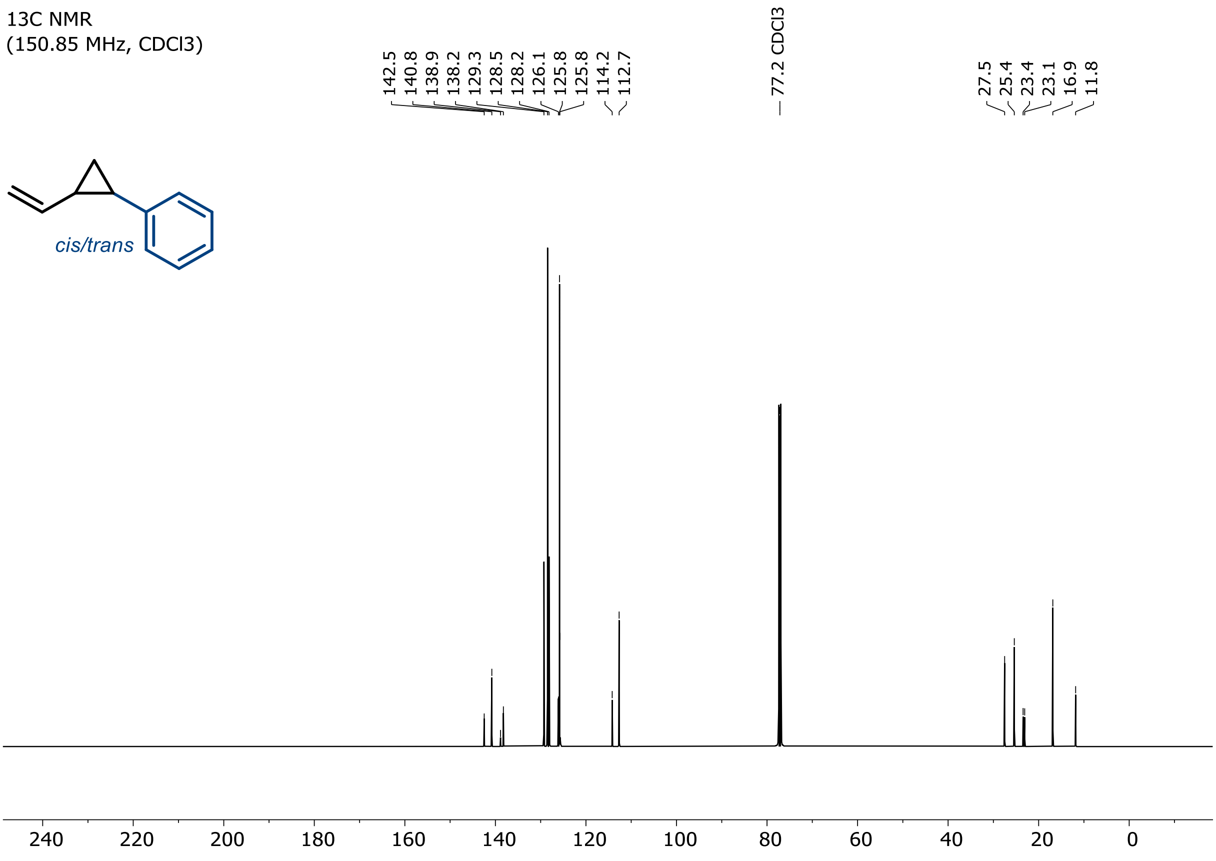

trans-(2-vinylcyclopropyl)benzene (4)

1H NMR
(399.97 MHz, $CDCl_3$)

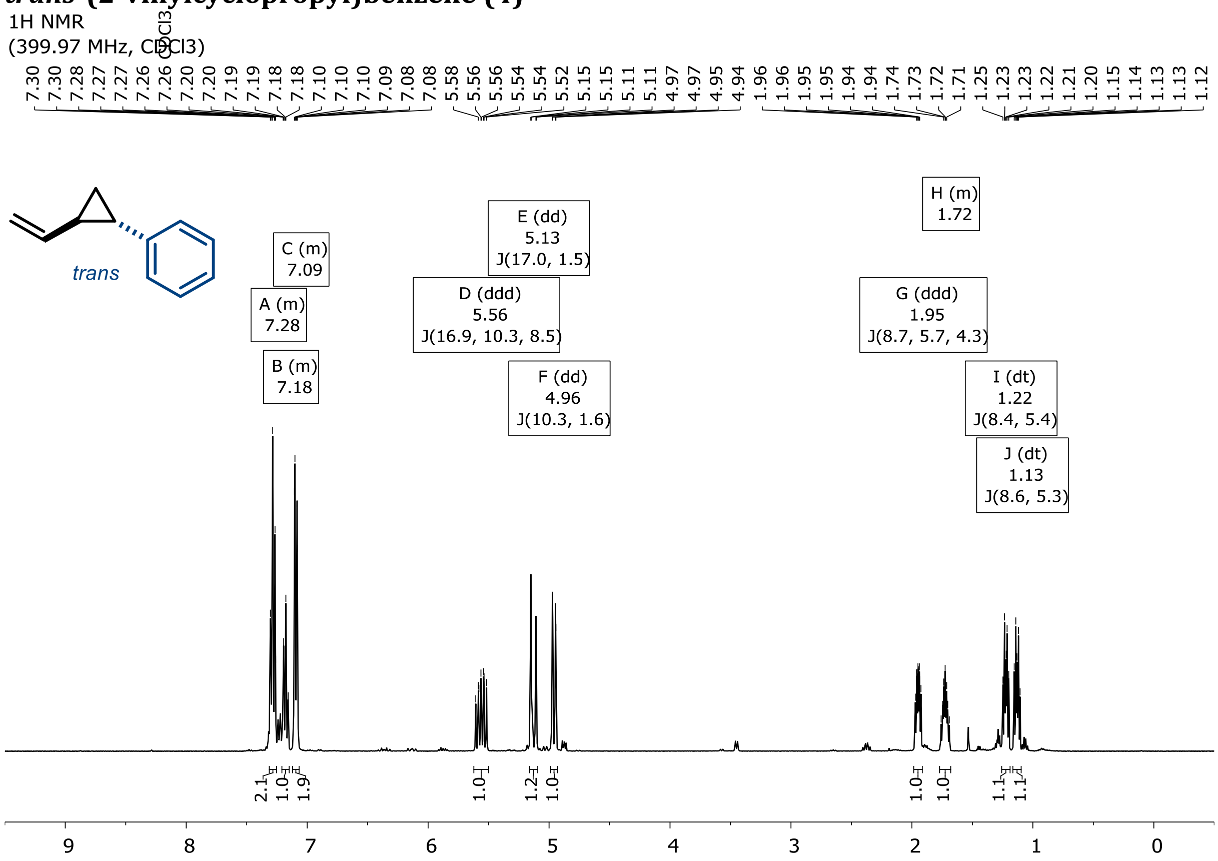

13C NMR
(151.00 MHz, $CDCl_3$)

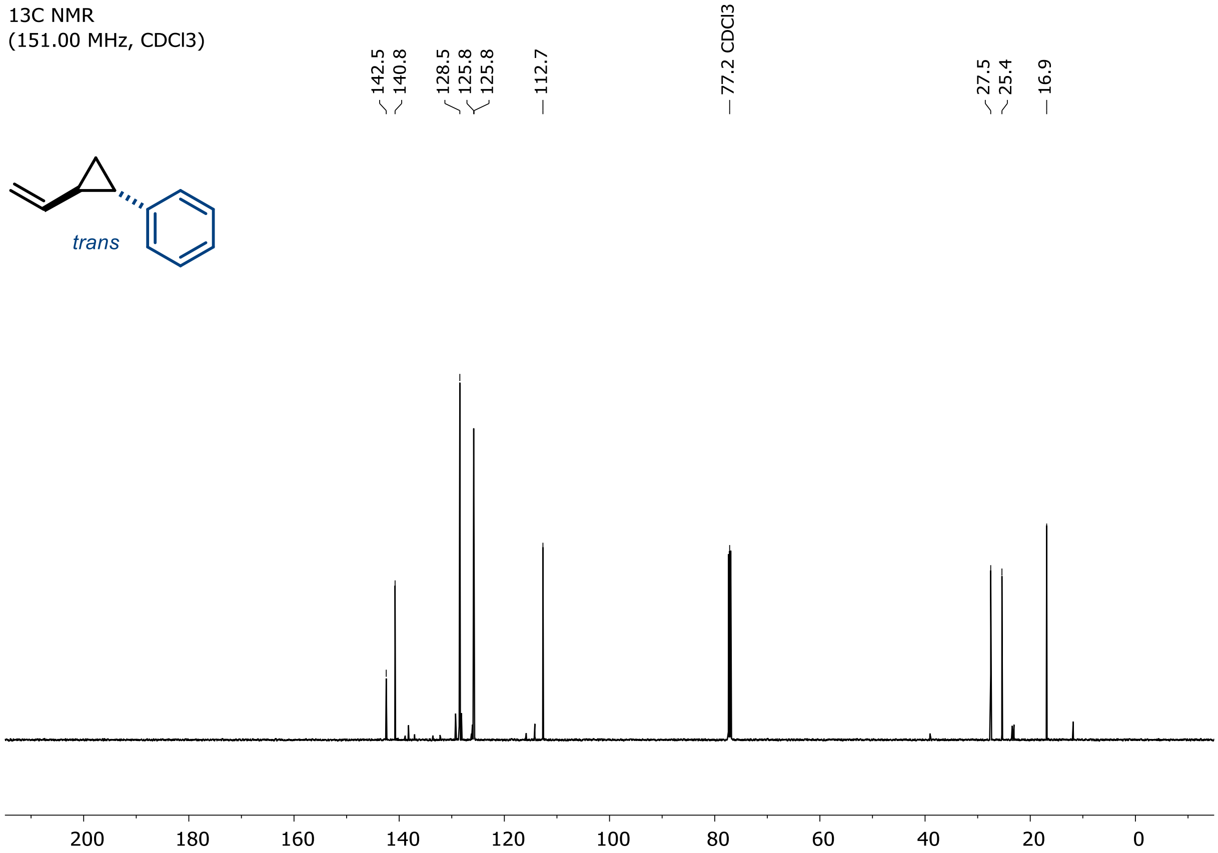

1-(*tert*-butyl)-4-(2-vinylcyclopropyl)benzene (*S*5)

1H NMR
(600.44 MHz, CDCl3)

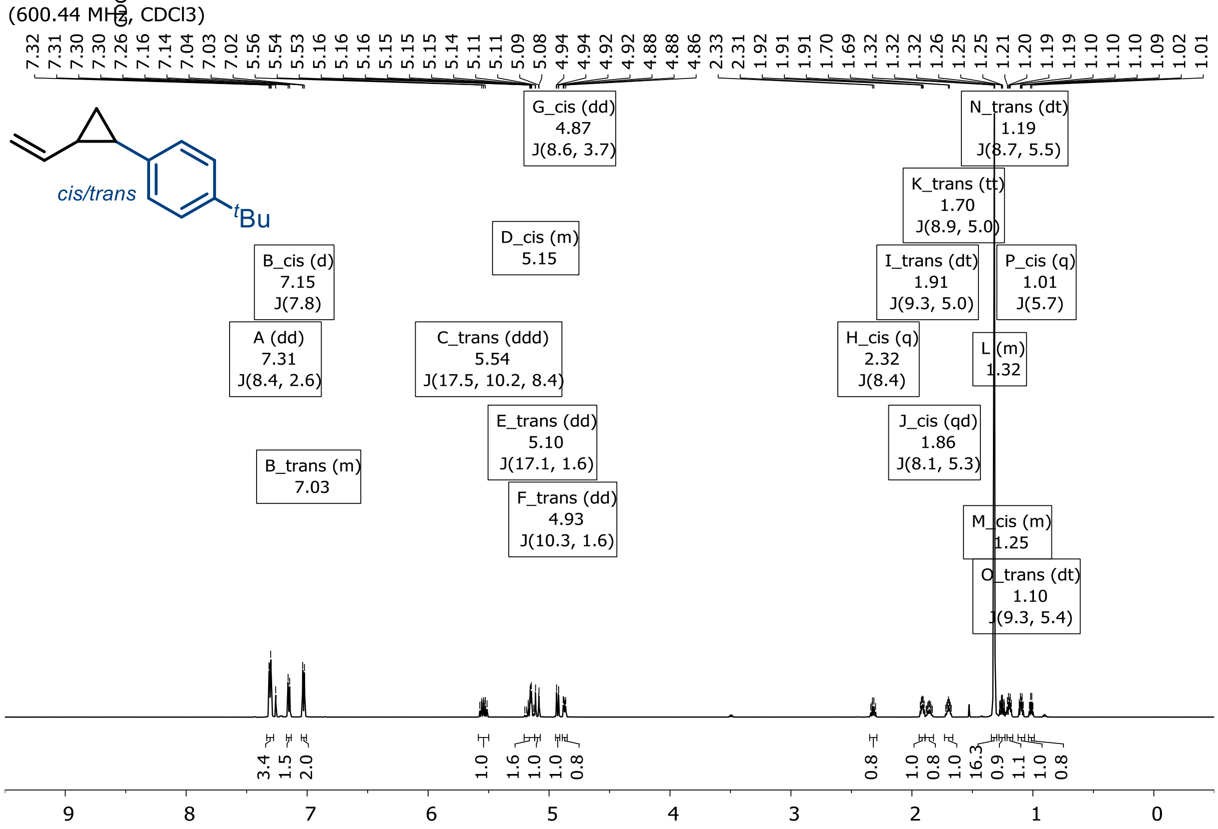

13C NMR
(151.00 MHz, CDCl3)

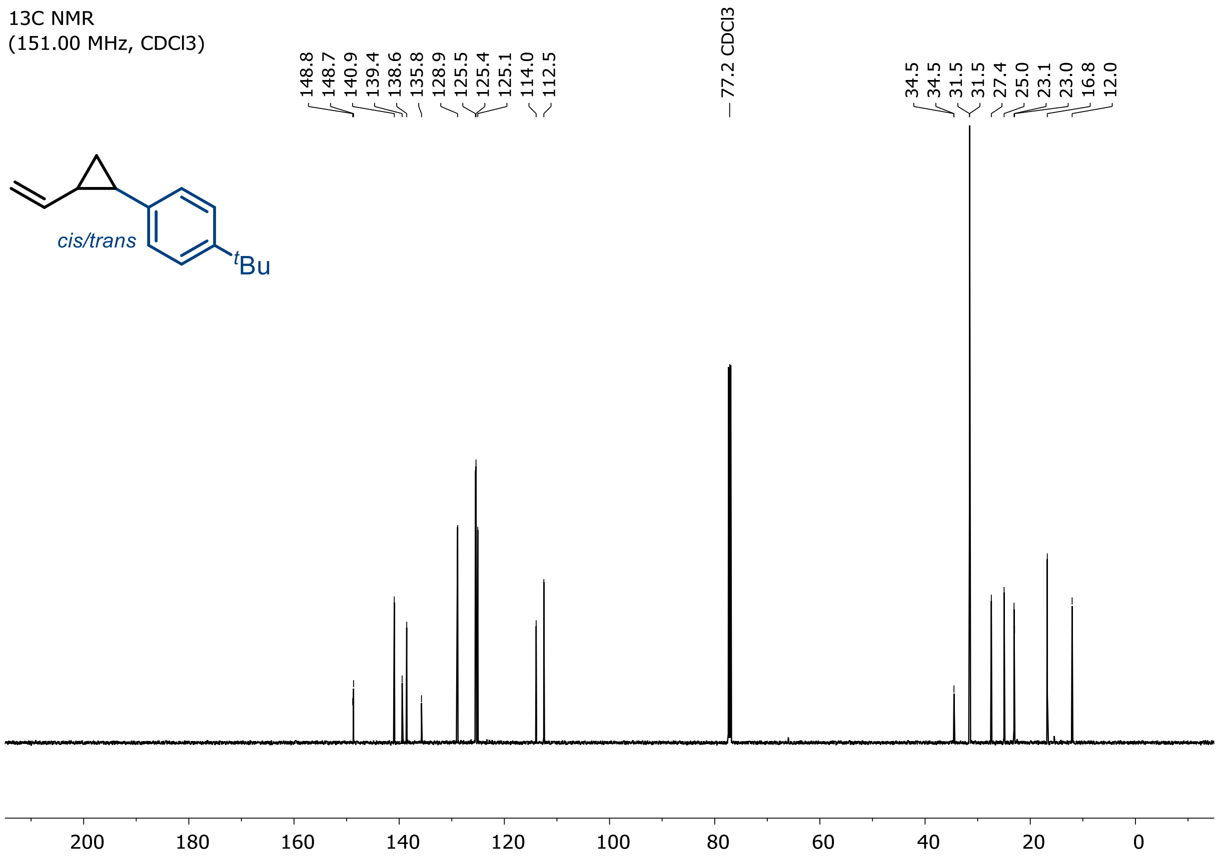

trans-1-(*tert*-butyl)-4-(2-vinylcyclopropyl)benzene (5)

1H NMR
(300.03 MHz, CDCl3)

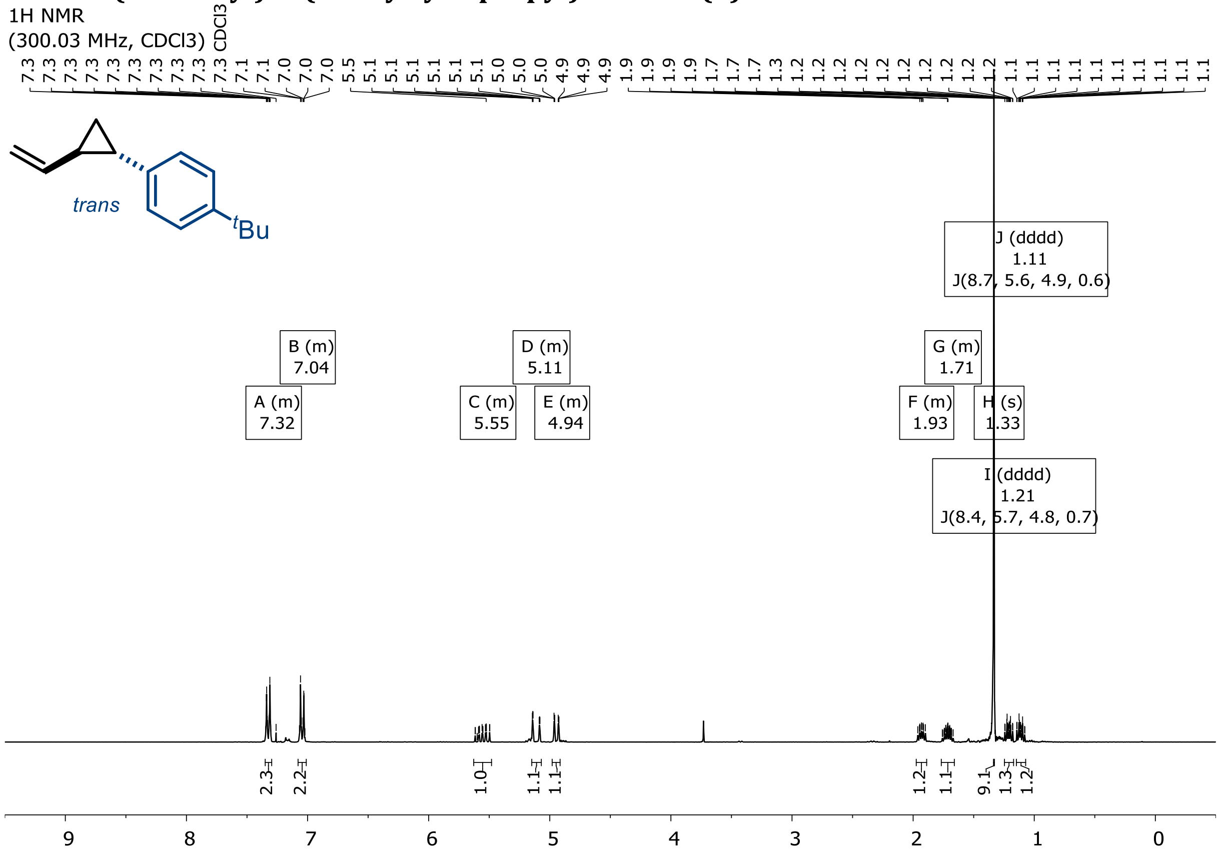

13C NMR
(151.00 MHz, CDCl3)

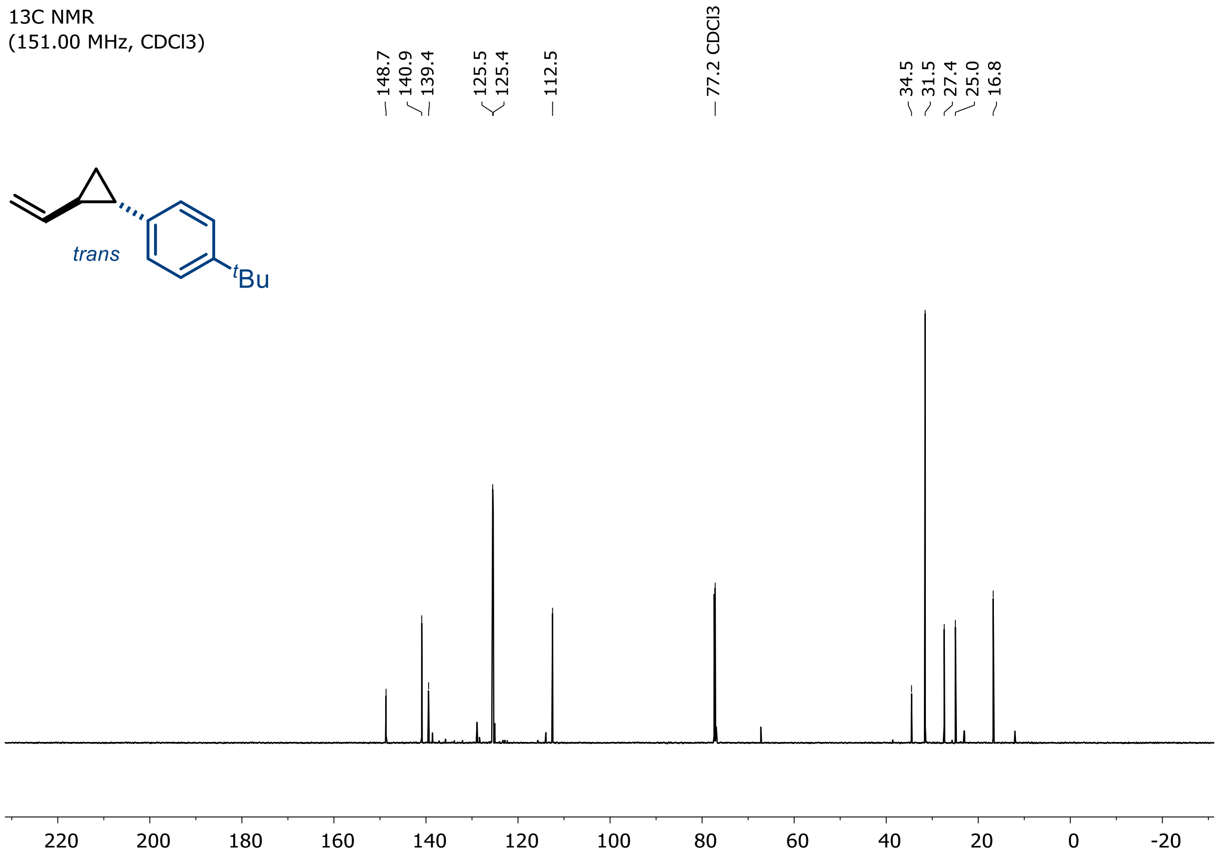

1-methyl-3-(2-vinylcyclopropyl)benzene (S6)

1H NMR
(599.86 MHz, CDCl3)

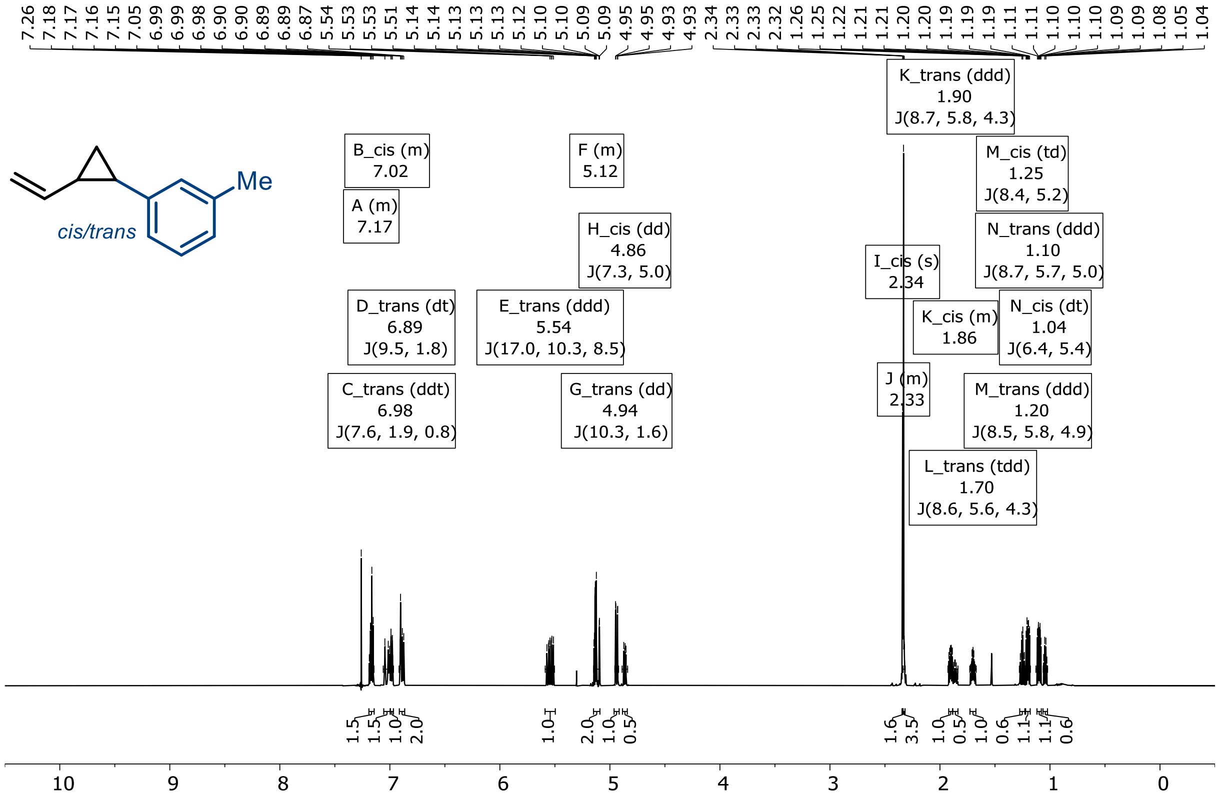

13C NMR
(150.85 MHz, CDCl3)

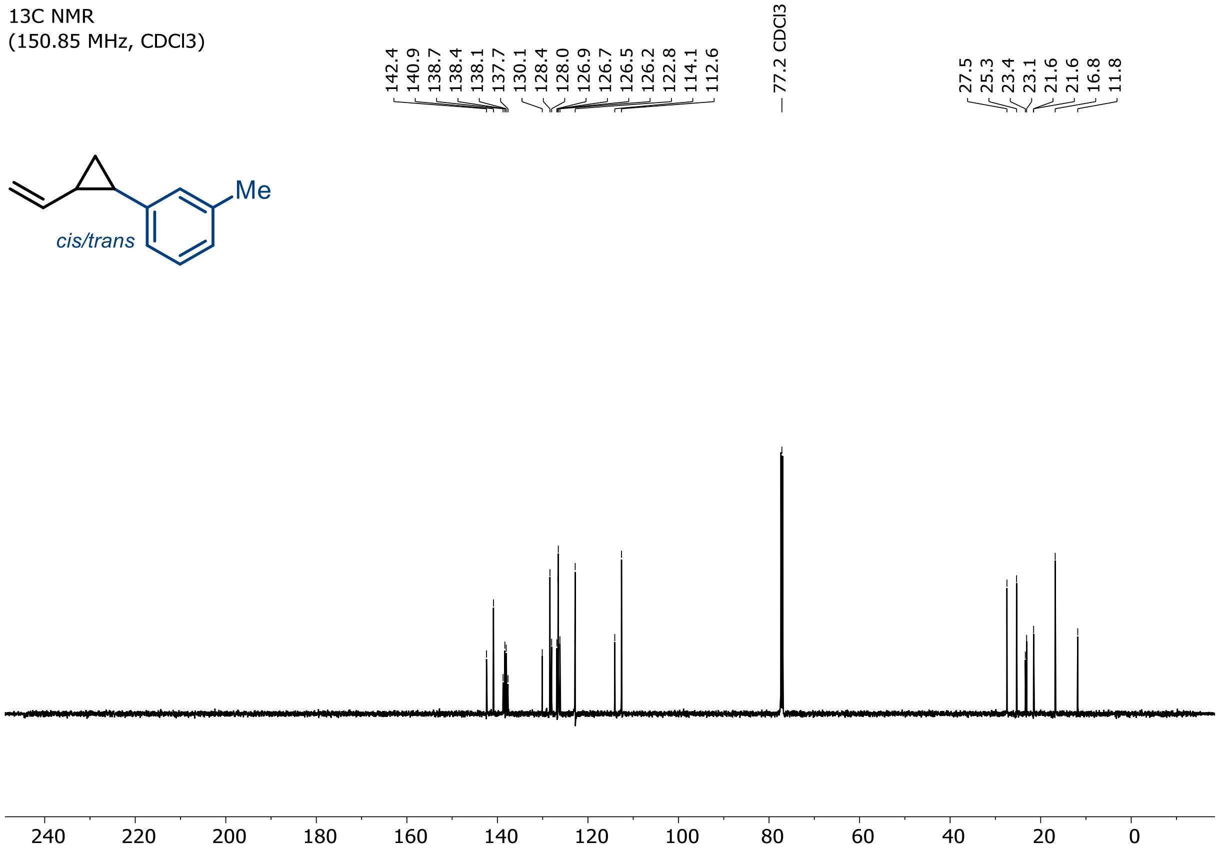

trans-1-methyl-3-(2-vinylcyclopropyl)benzene (6)

1H NMR
(600.44 MHz, CDCl3)

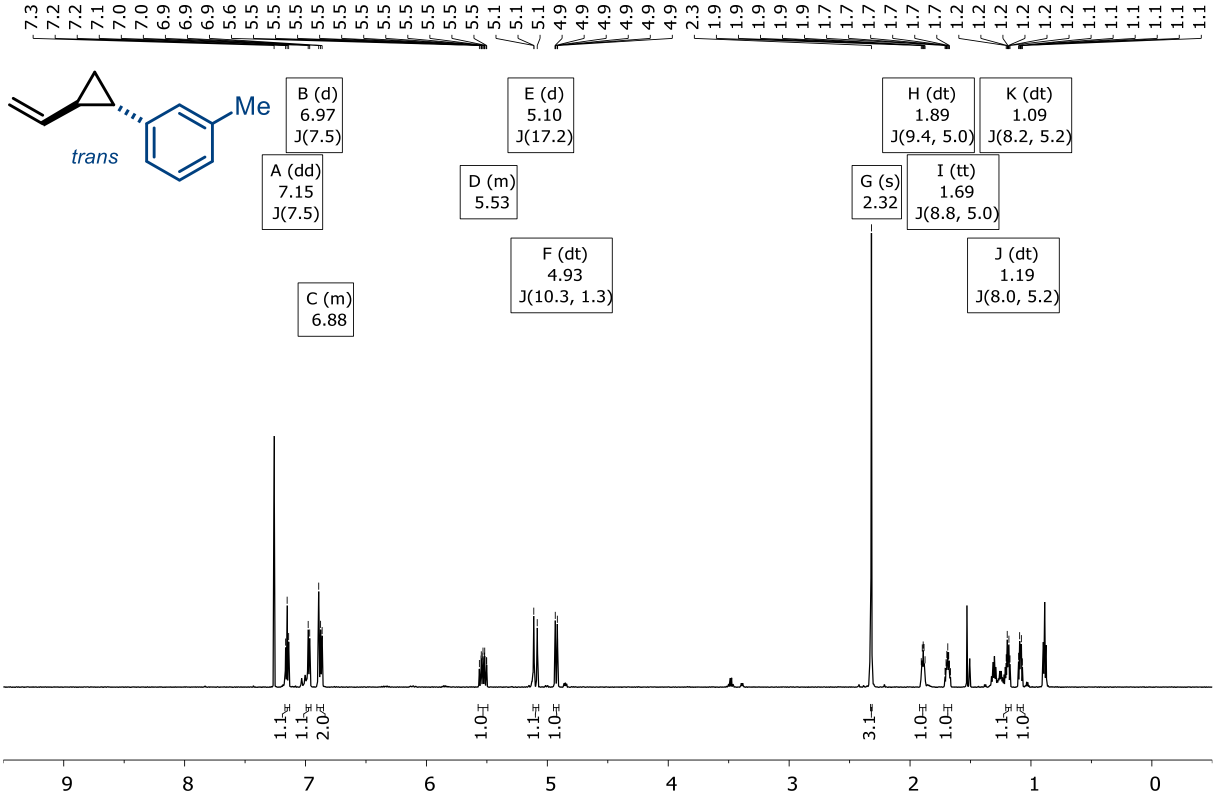

13C NMR
(151.00 MHz, CDCl3)

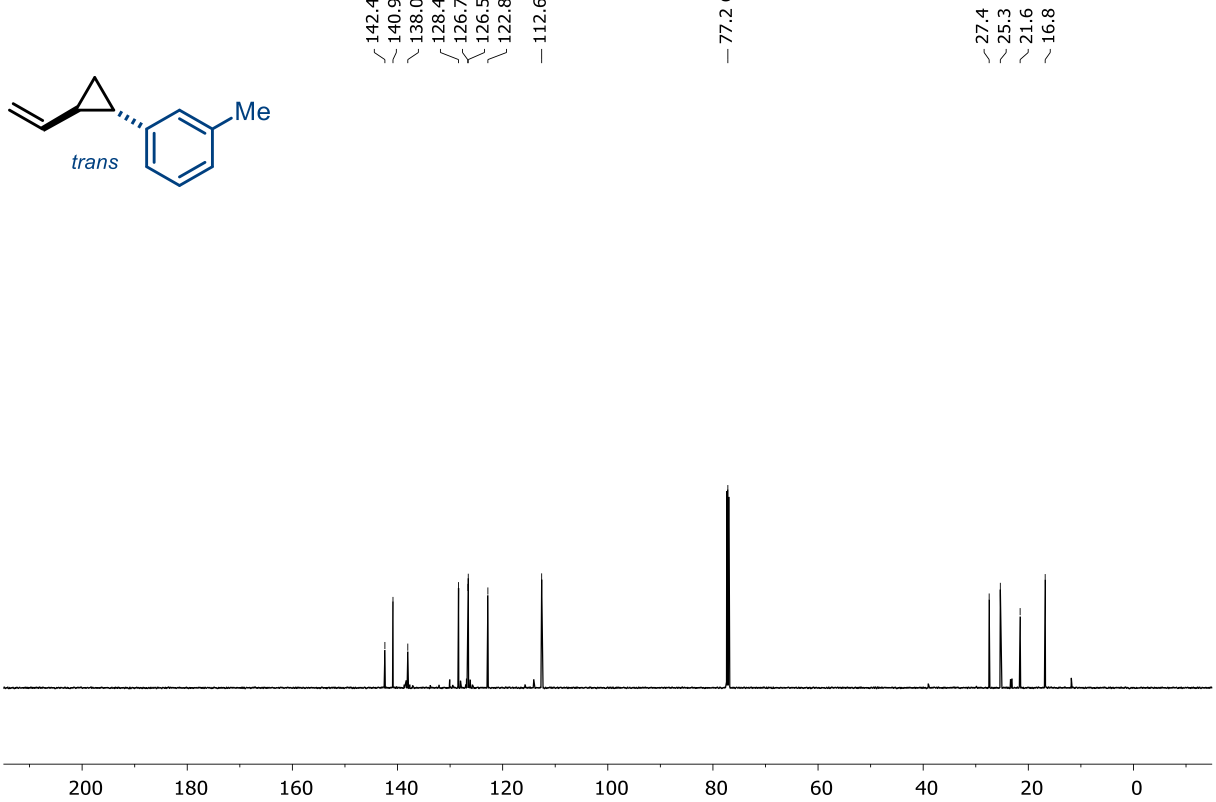

1-(trifluoromethyl)-4-(2-vinylcyclopropyl)benzene (*S7*)

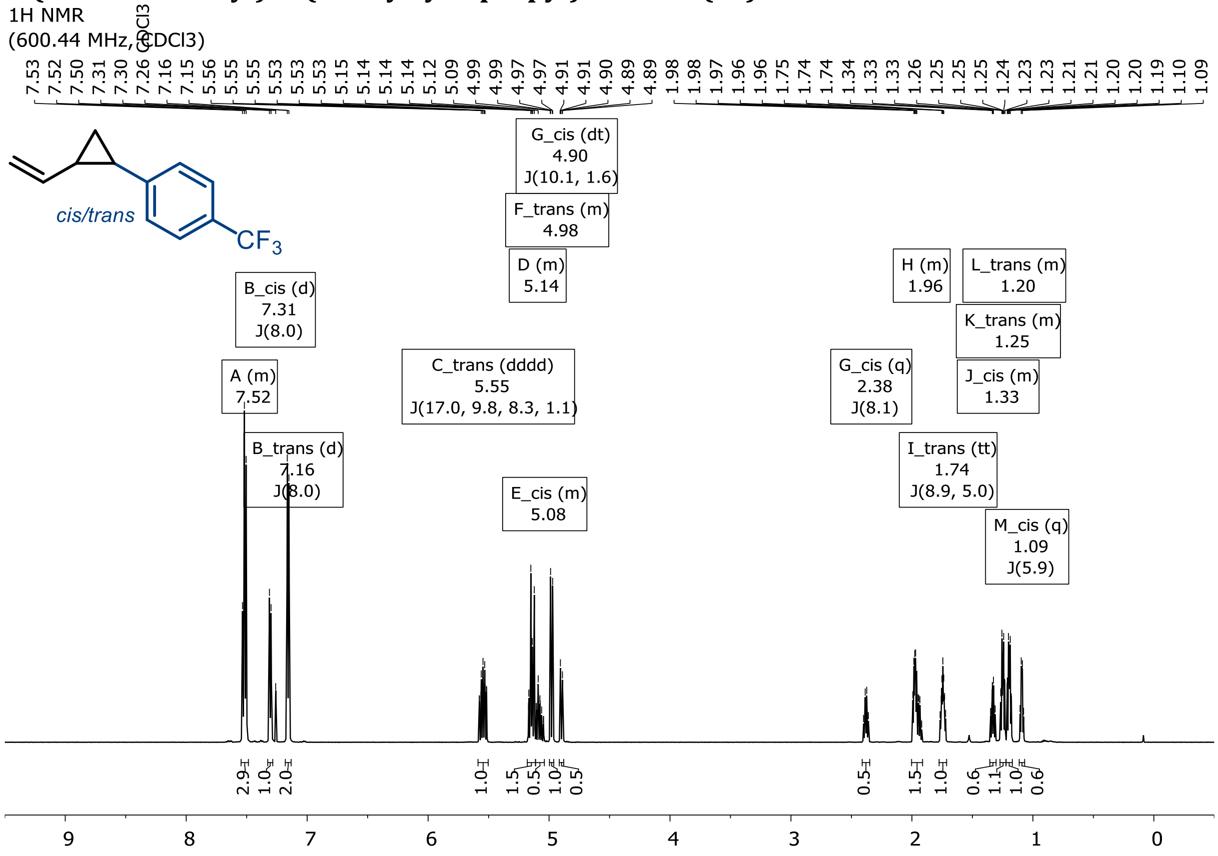

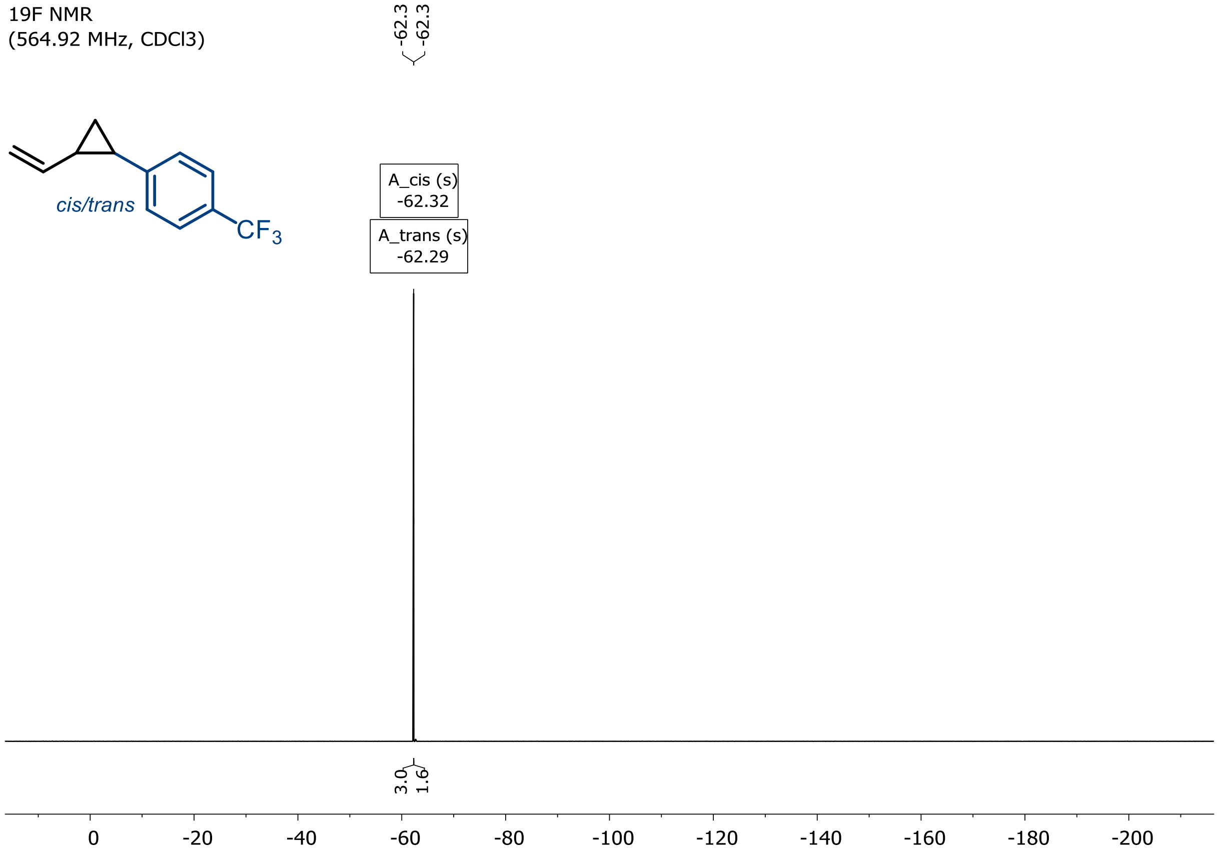

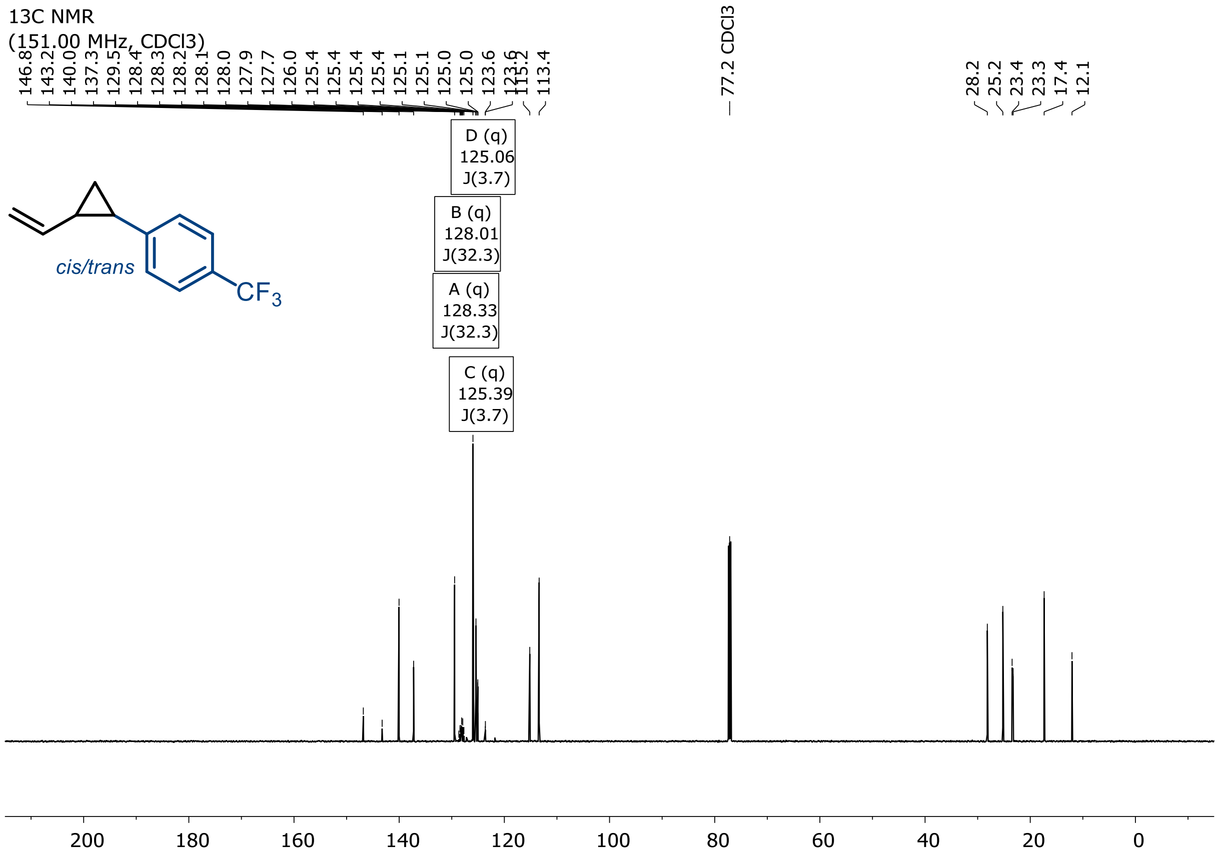

trans-1-(trifluoromethyl)-4-(2-vinylcyclopropyl)benzene (7)

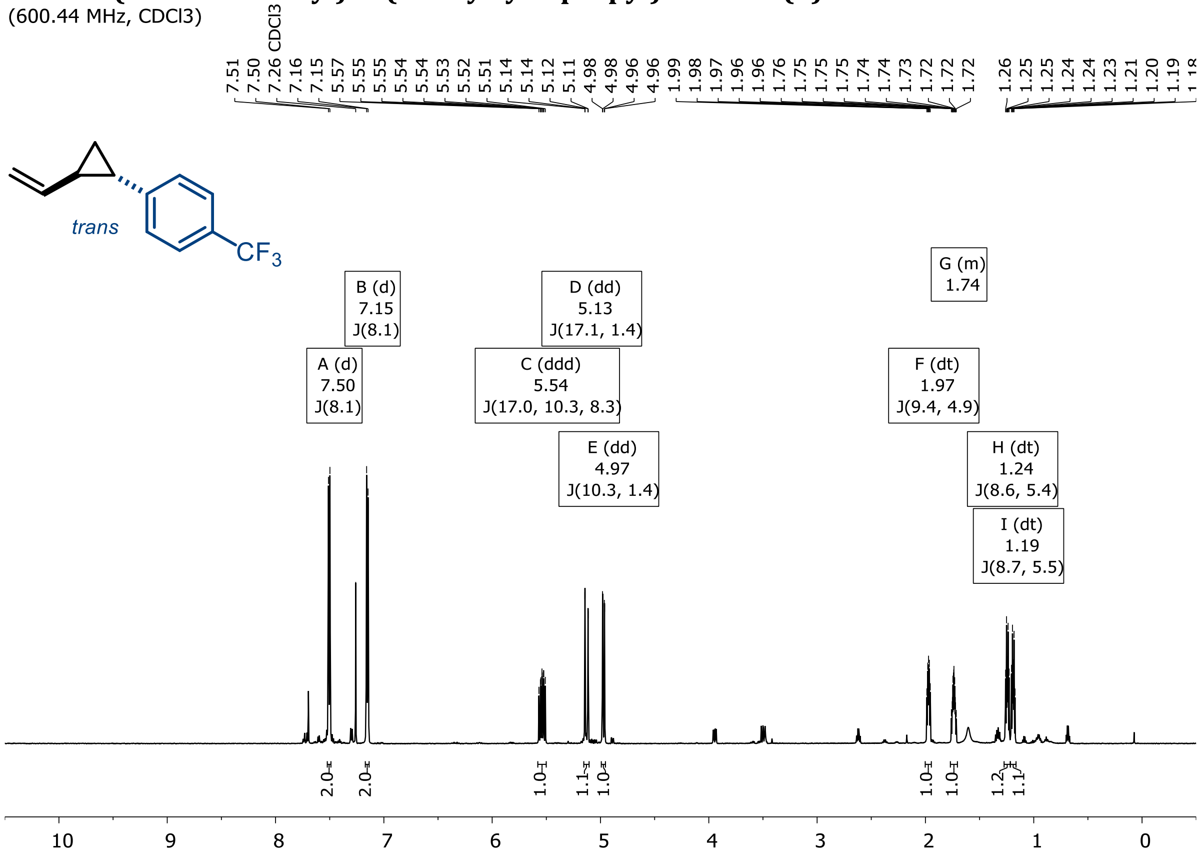

19F NMR
(282.28 MHz, CDCl3)

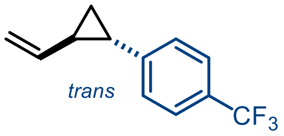

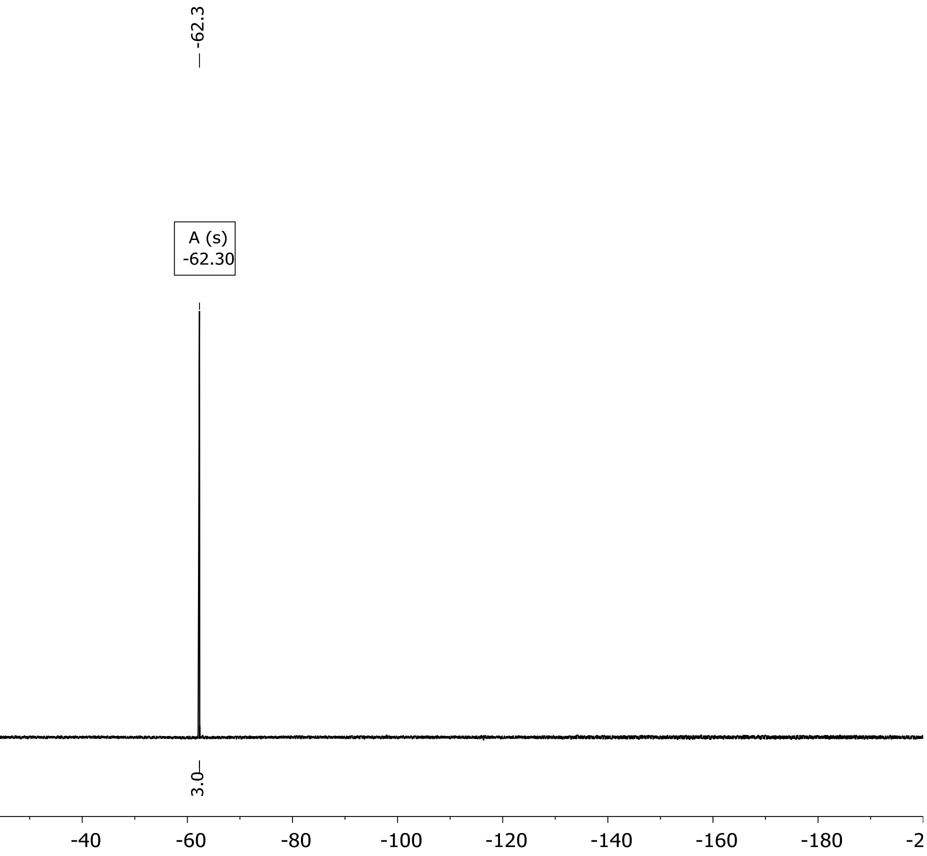

13C NMR
(151.00 MHz, CDCl3)

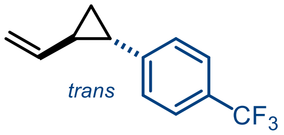

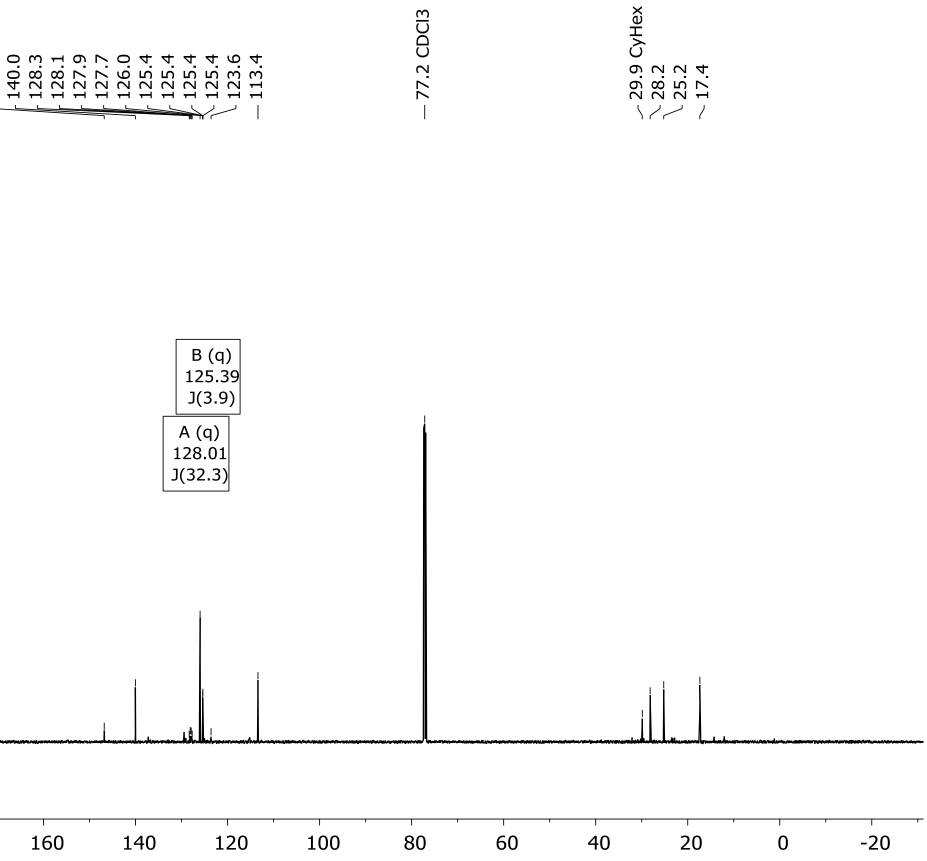

5-(*cis*-2-vinylcyclopropyl)benzo[*b*]thiophene (*S*8)

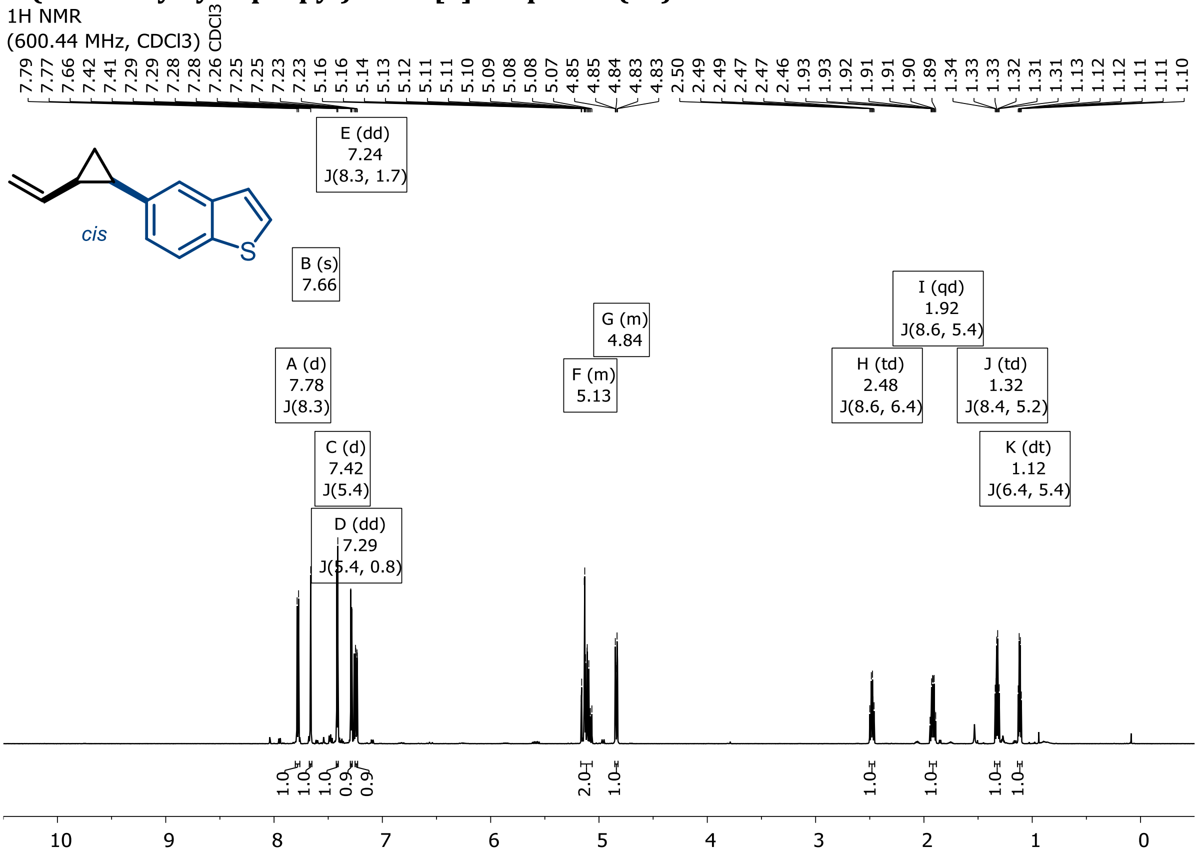

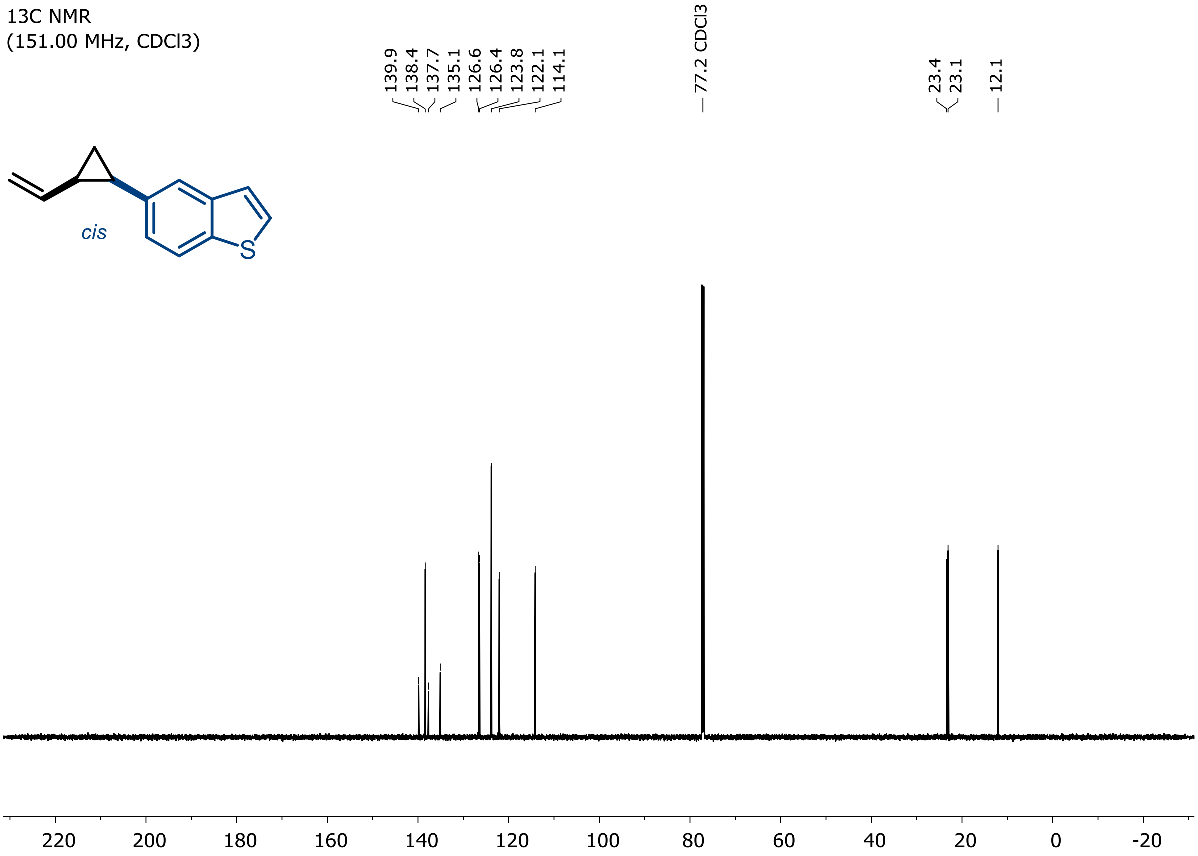

5-(*trans*-2-vinylcyclopropyl)benzo[*b*]thiophene (8)

1H NMR
(600.44 MHz, CDCl3)

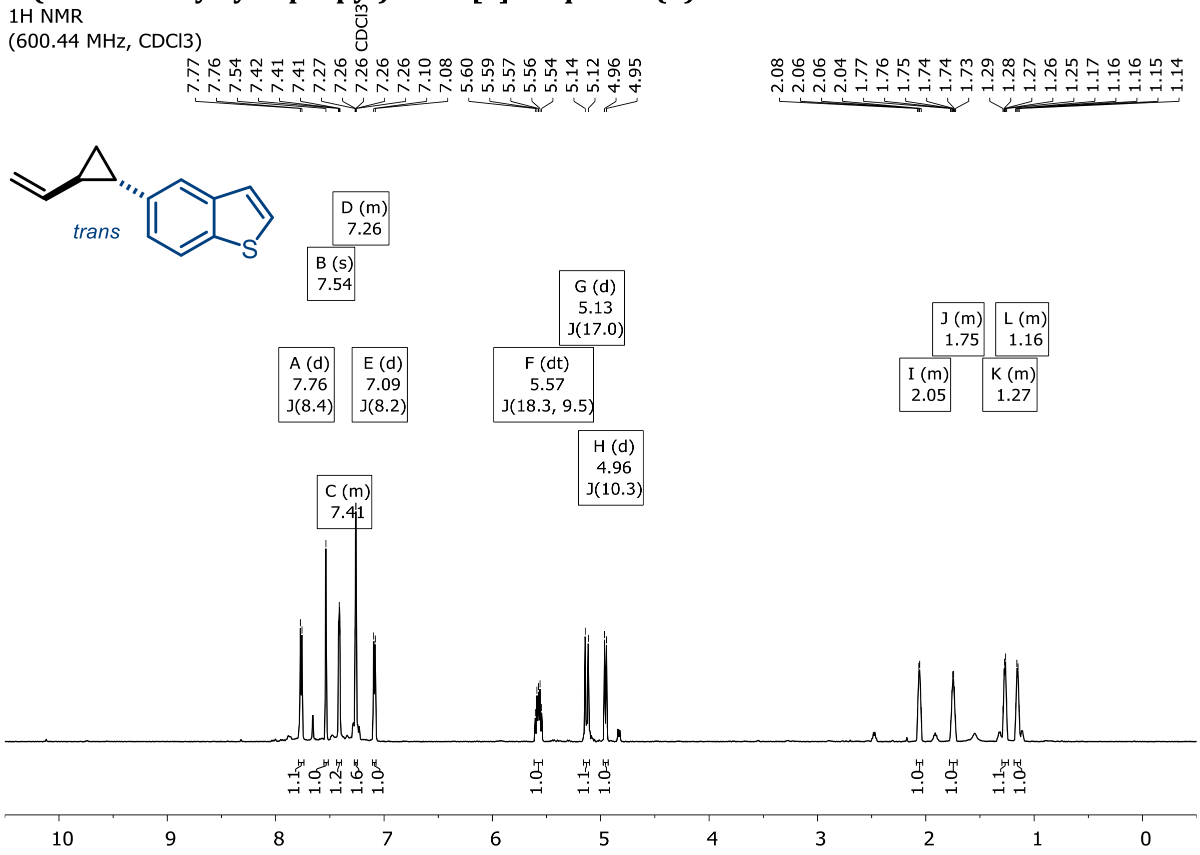

13C NMR
(151.00 MHz, CDCl3)

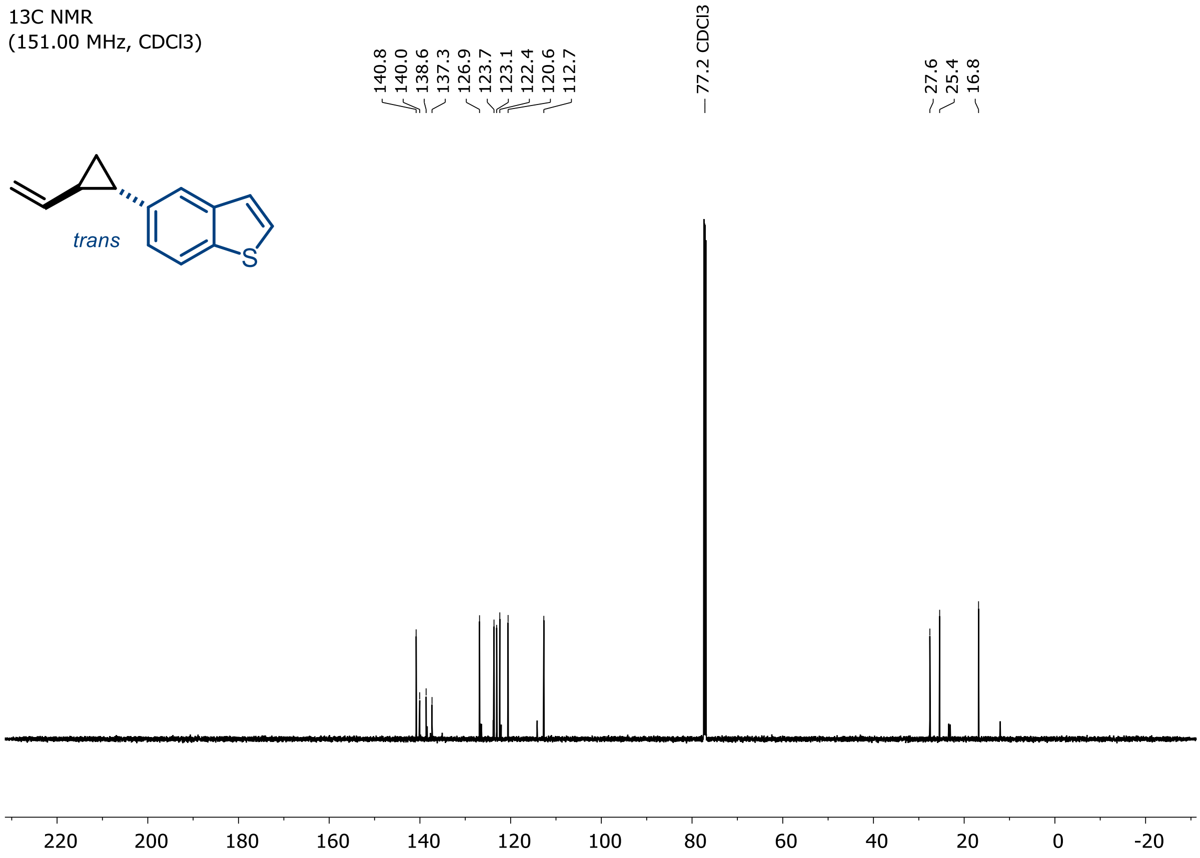

5-(*cis*-2-vinylcyclopropyl)benzofuran (S9)

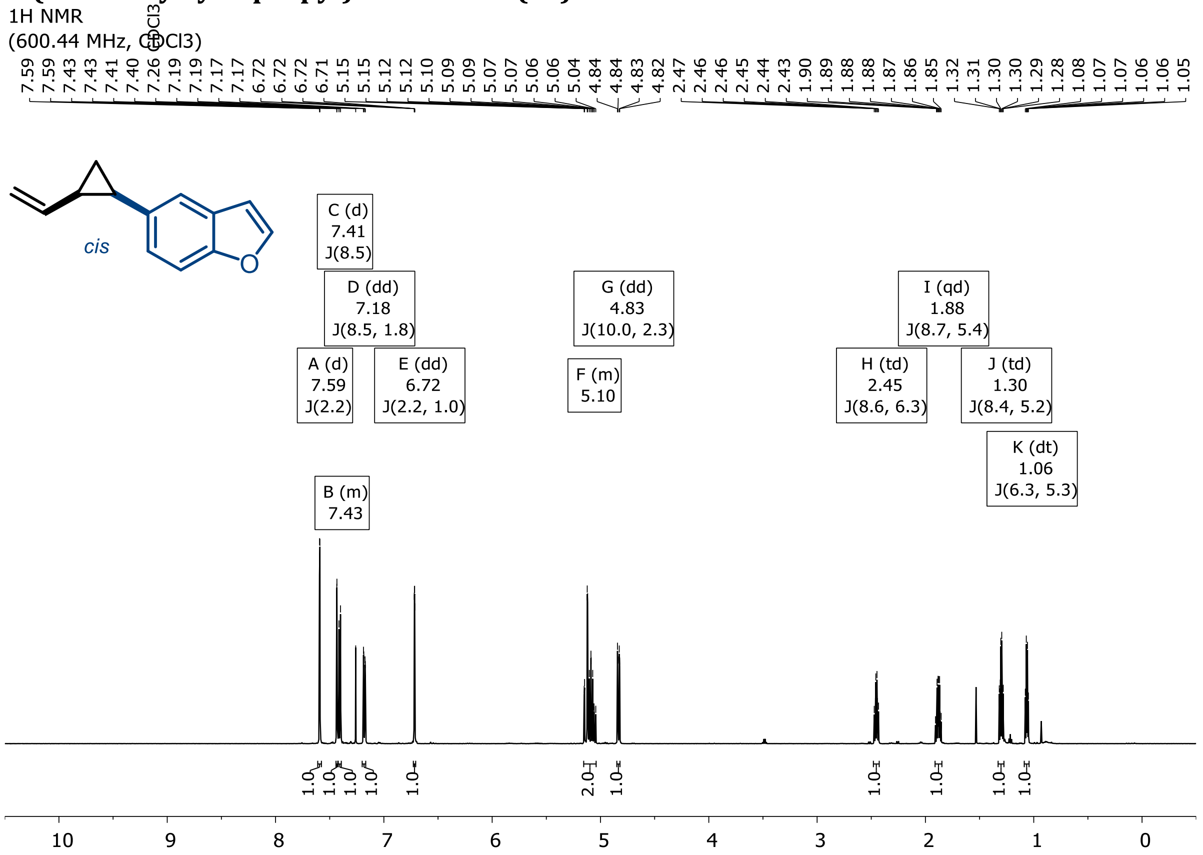

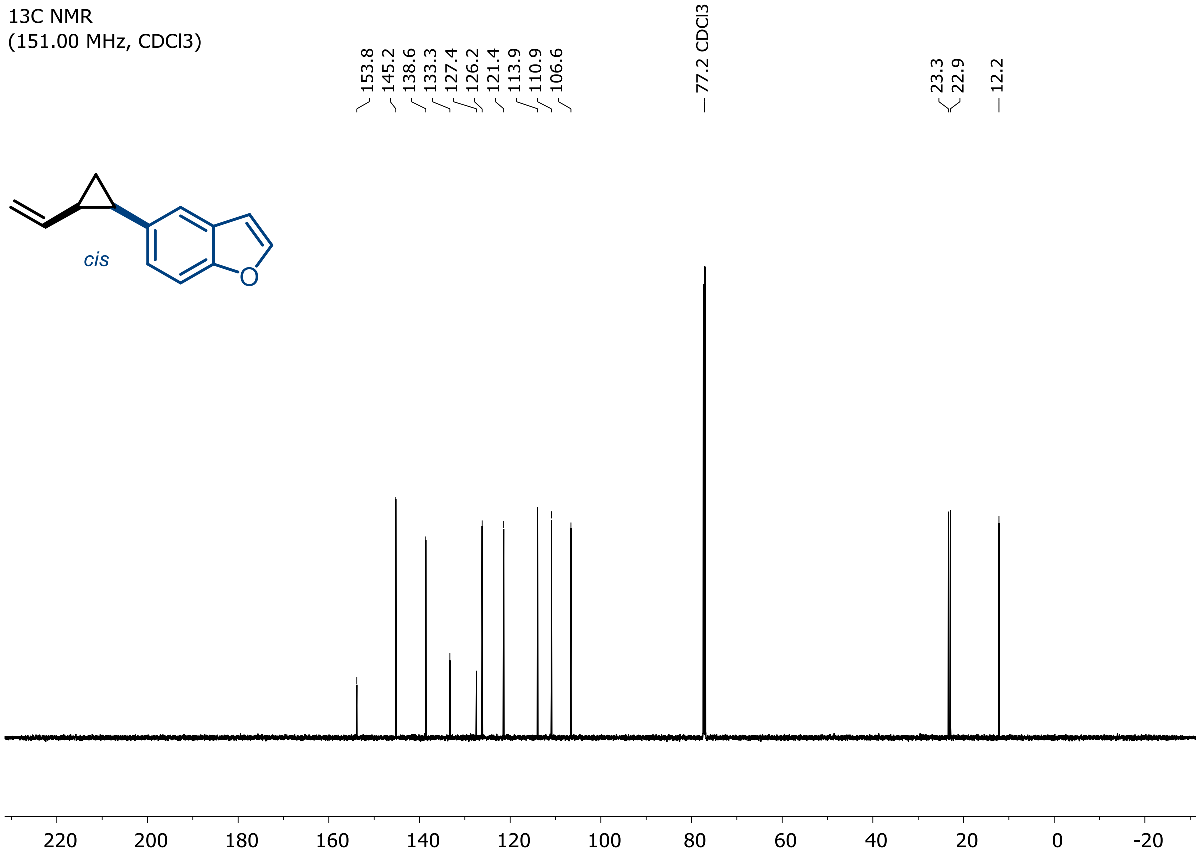

5-(*trans*-2-vinylcyclopropyl)benzofuran (9)

(600.44 MHz, CDCl3)

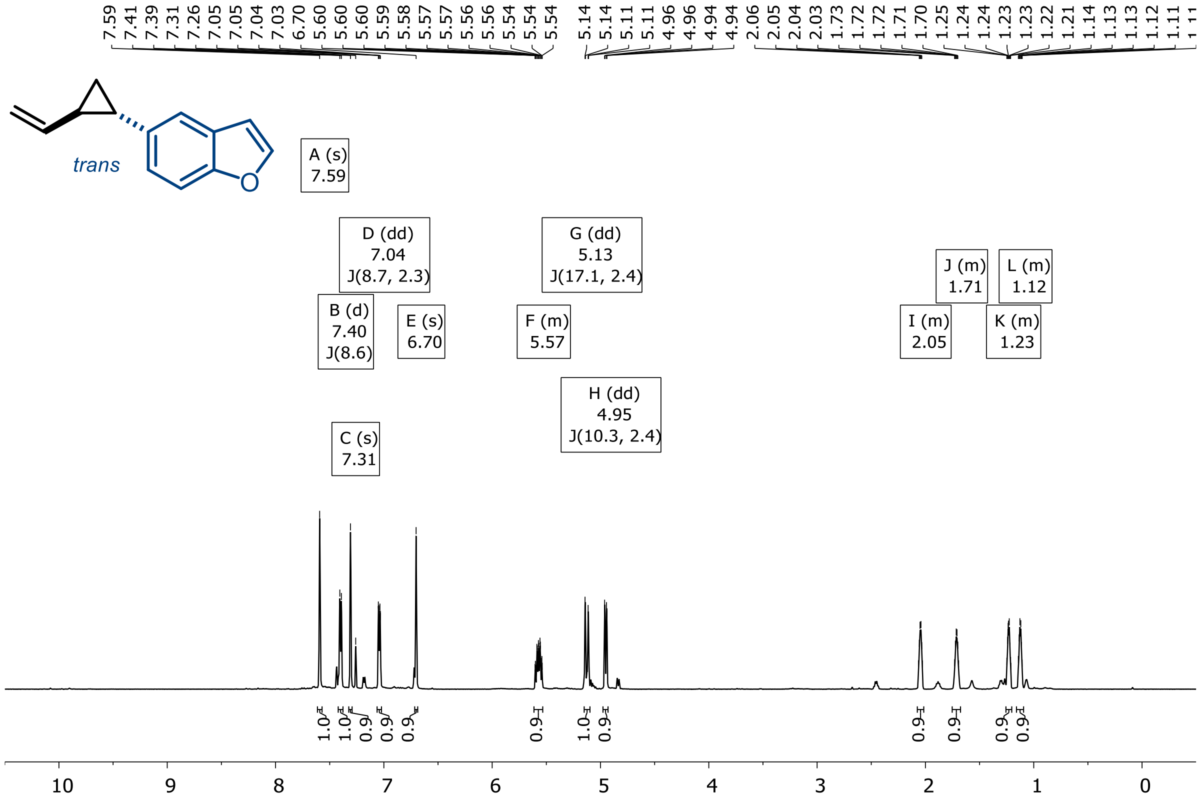

13C NMR

(151.00 MHz, CDCl3)

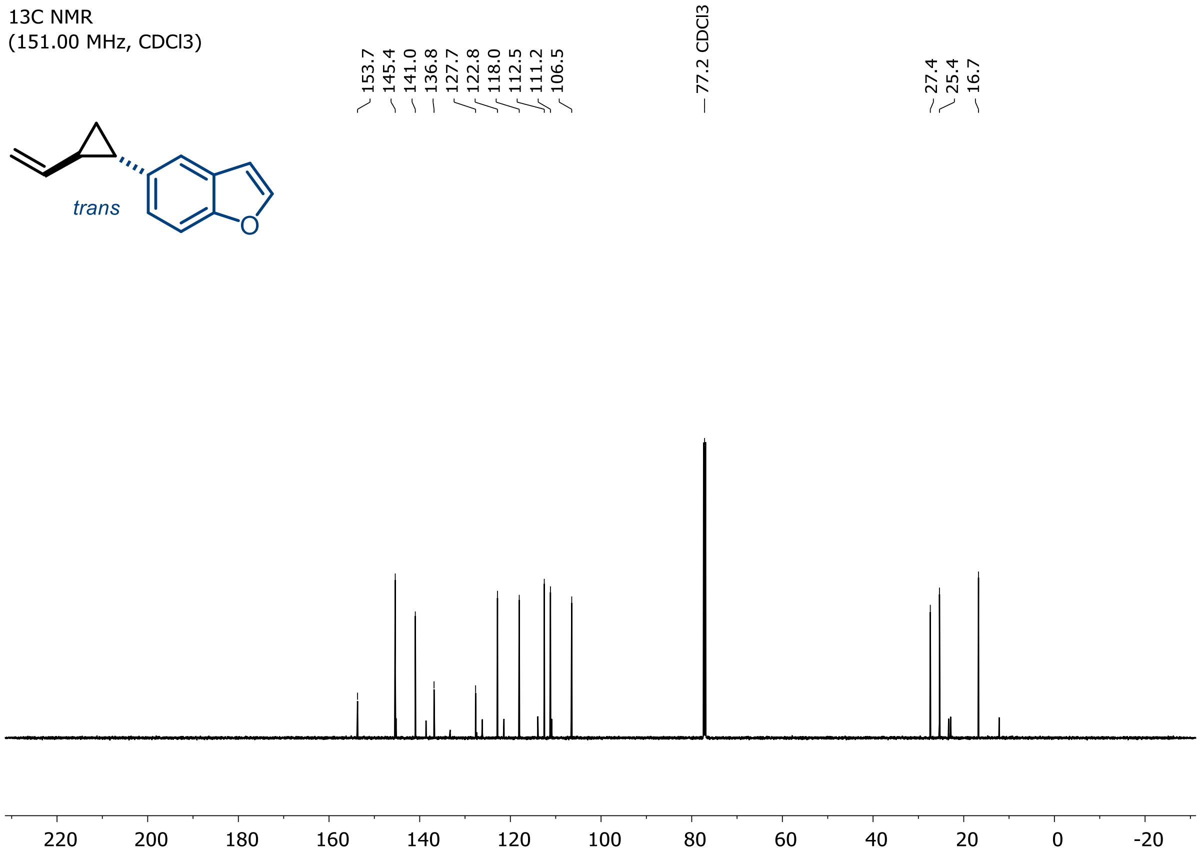

(4-hydroxyphenyl)(2-vinylcyclopropyl)methanone (S10)

(600.44 MHz, CDCl3)

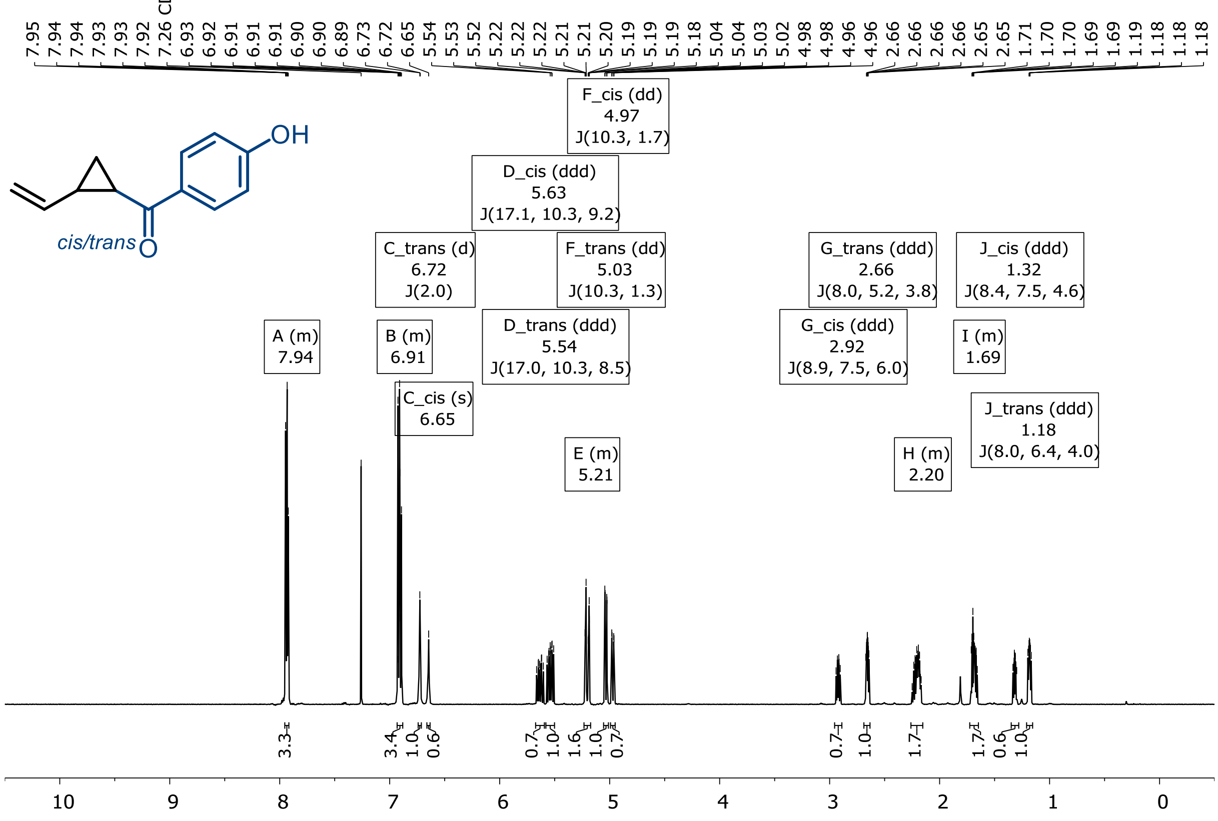

13C NMR
(151.00 MHz,)

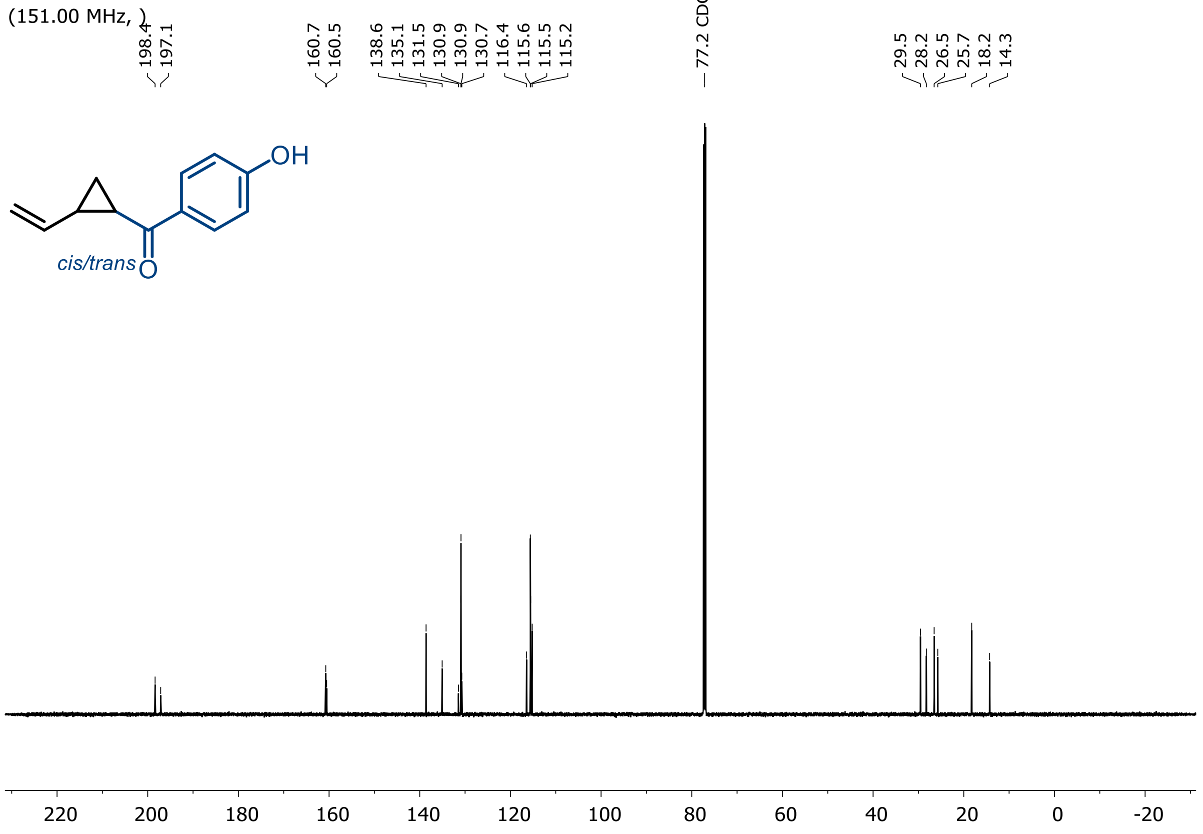

(4-hydroxyphenyl)(*trans*-2-vinylcyclopropyl)methanone (10)

(600.44 MHz, CDCl3)

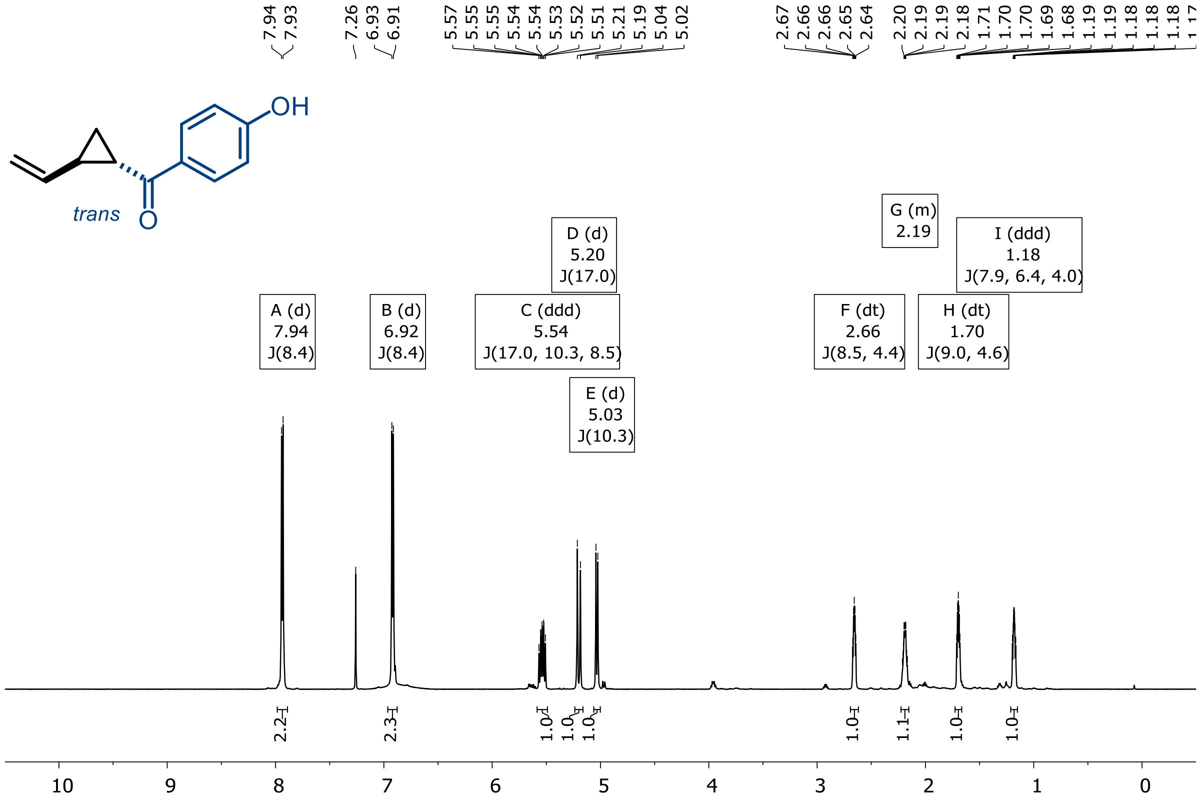

13C NMR
(151.00 MHz, CDCl3)

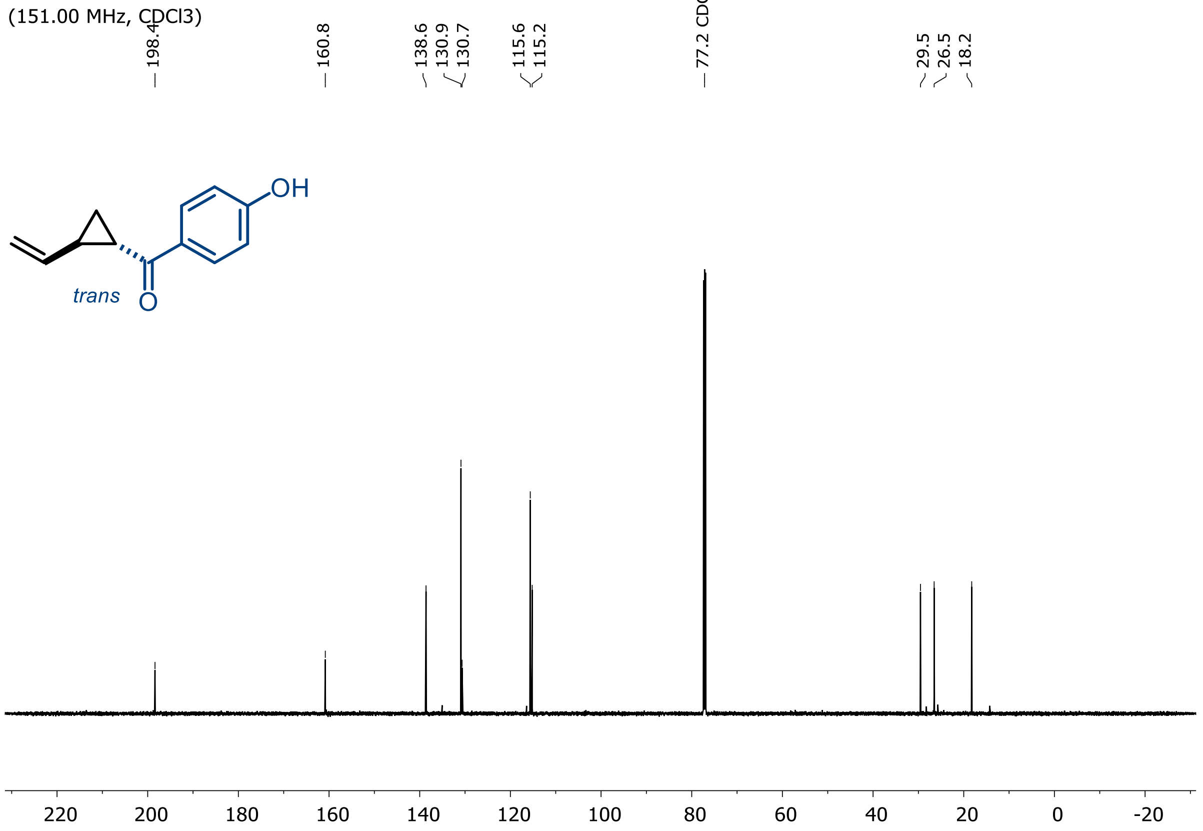

(4-aminophenyl)(2-vinylcyclopropyl)methanone (S11)

(600.44 MHz, $CDCl_3$)

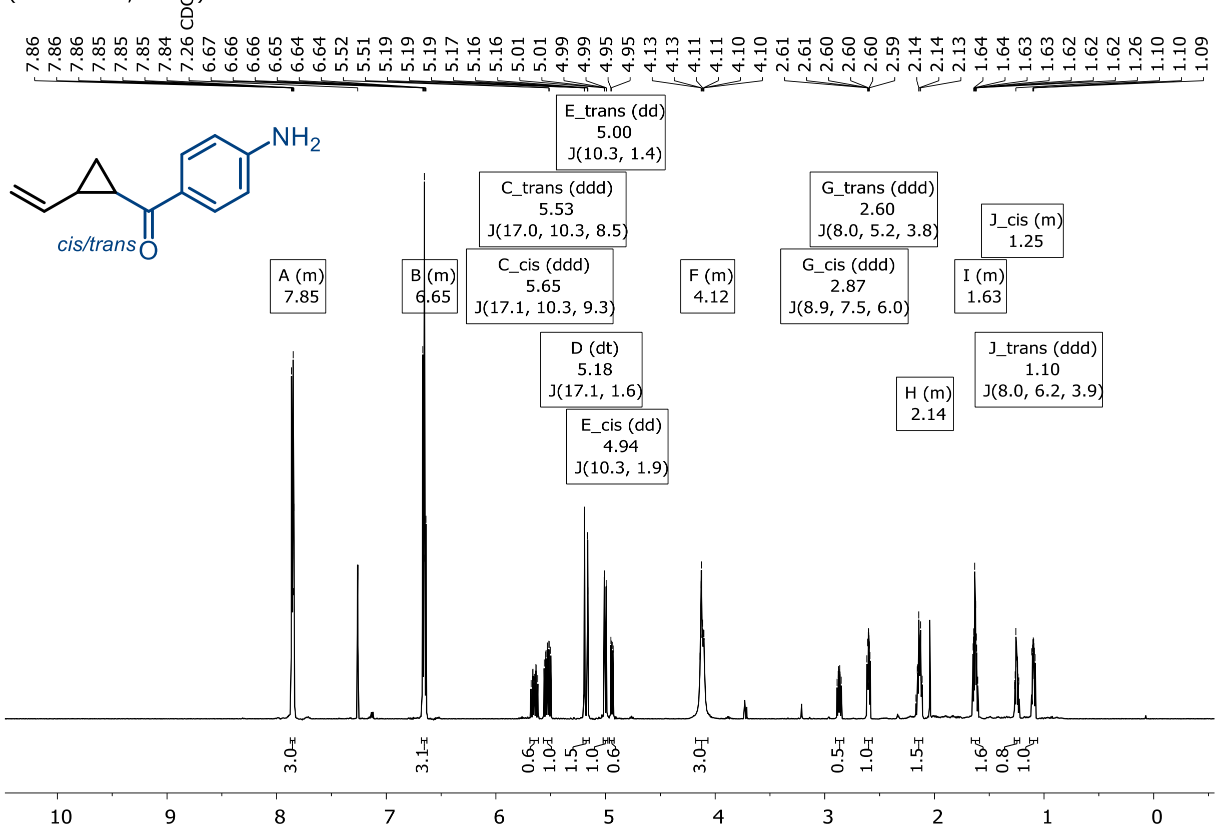

13C NMR
(151.00 MHz, $CDCl_3$)

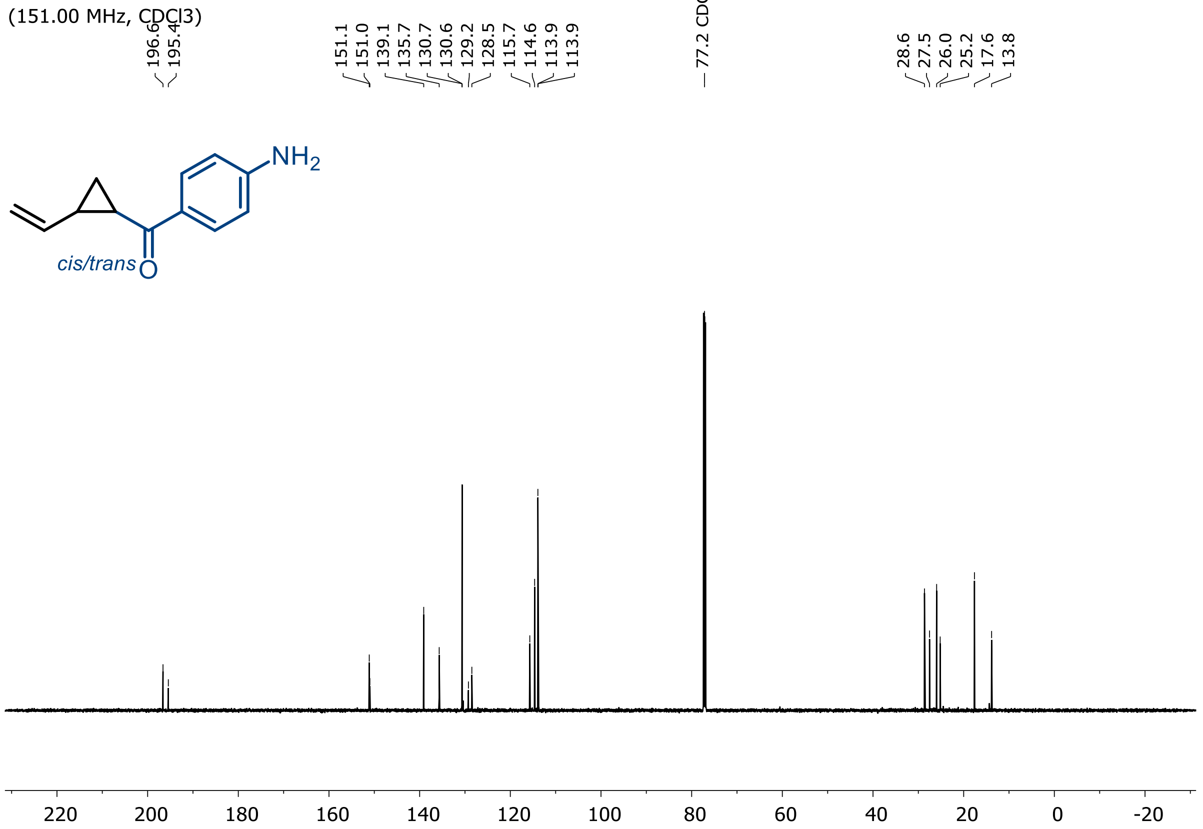

(4-aminophenyl)(*trans*-2-vinylcyclopropyl)methanone (11)

1H NMR
(399.97 MHz, CDCl3)

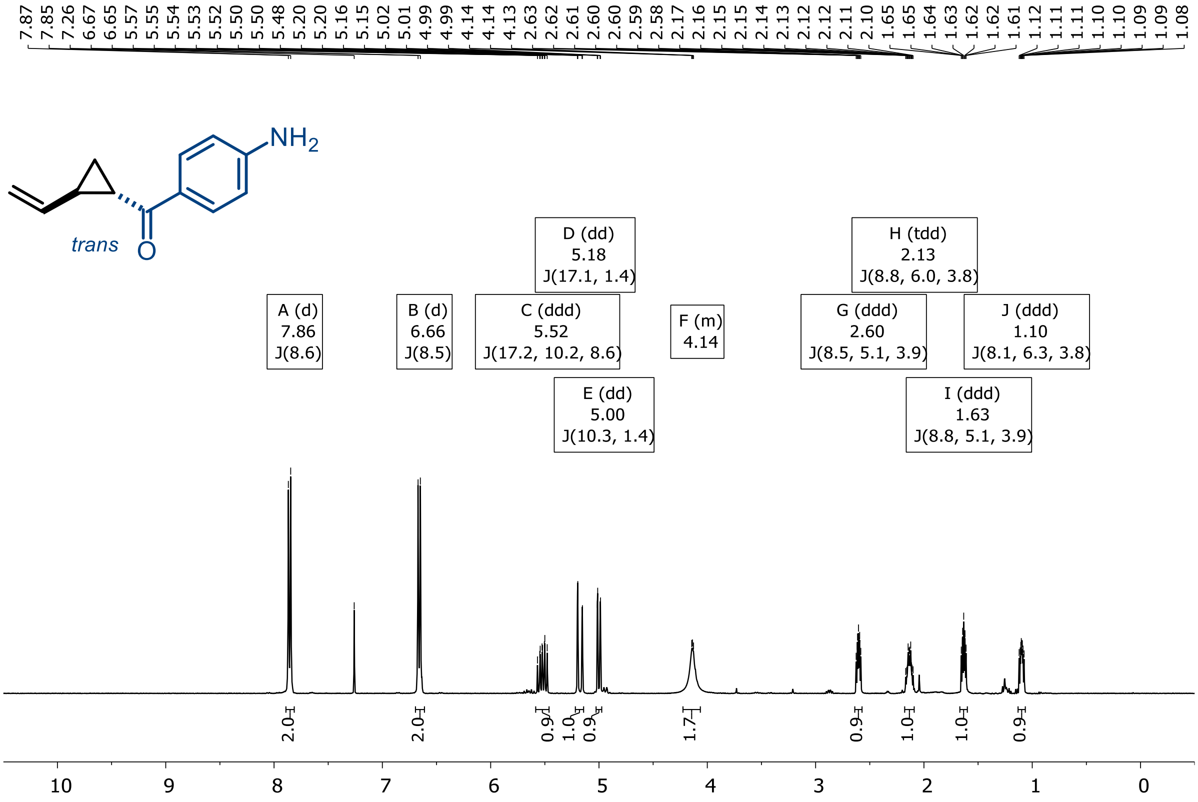

13C NMR
(100.58 MHz, CDCl3)

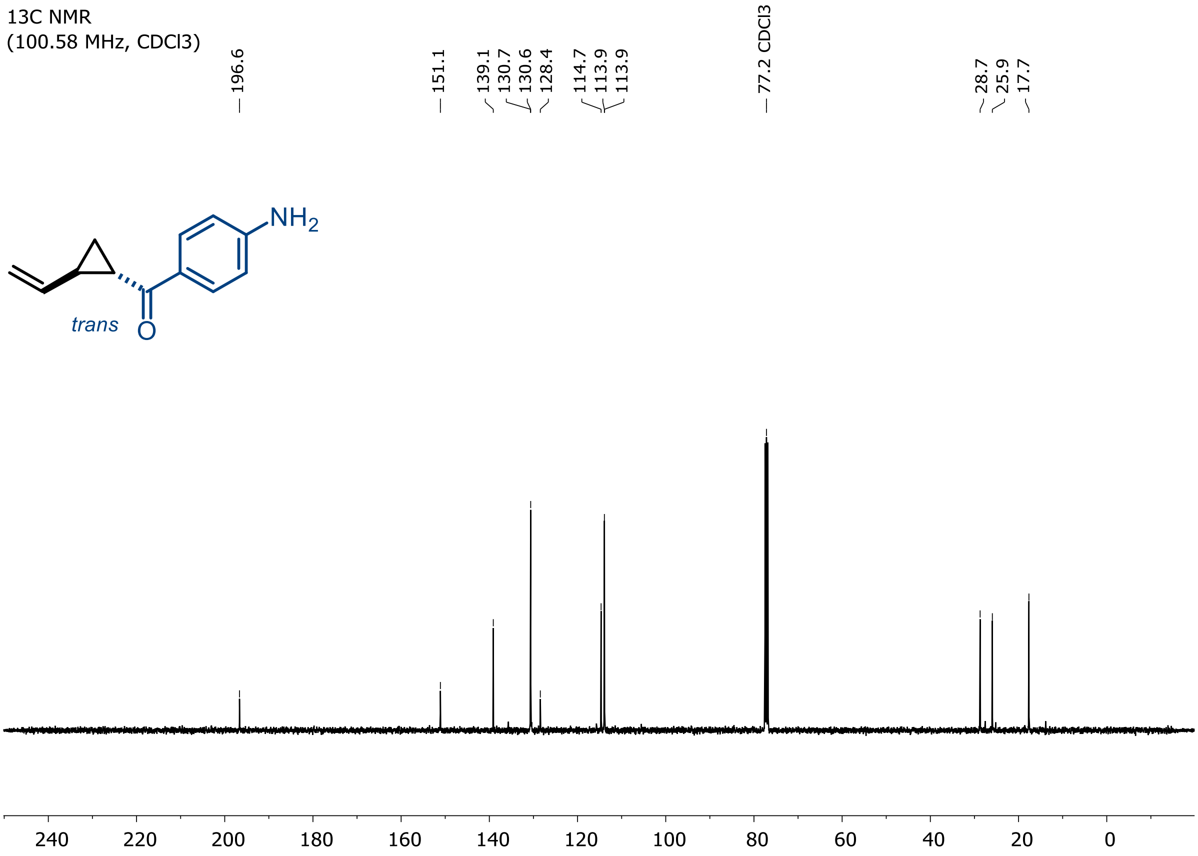

1-(2-vinylcyclopropyl)nonan-1-one (S12)

1H NMR
(600.44 MHz, CDCl3)

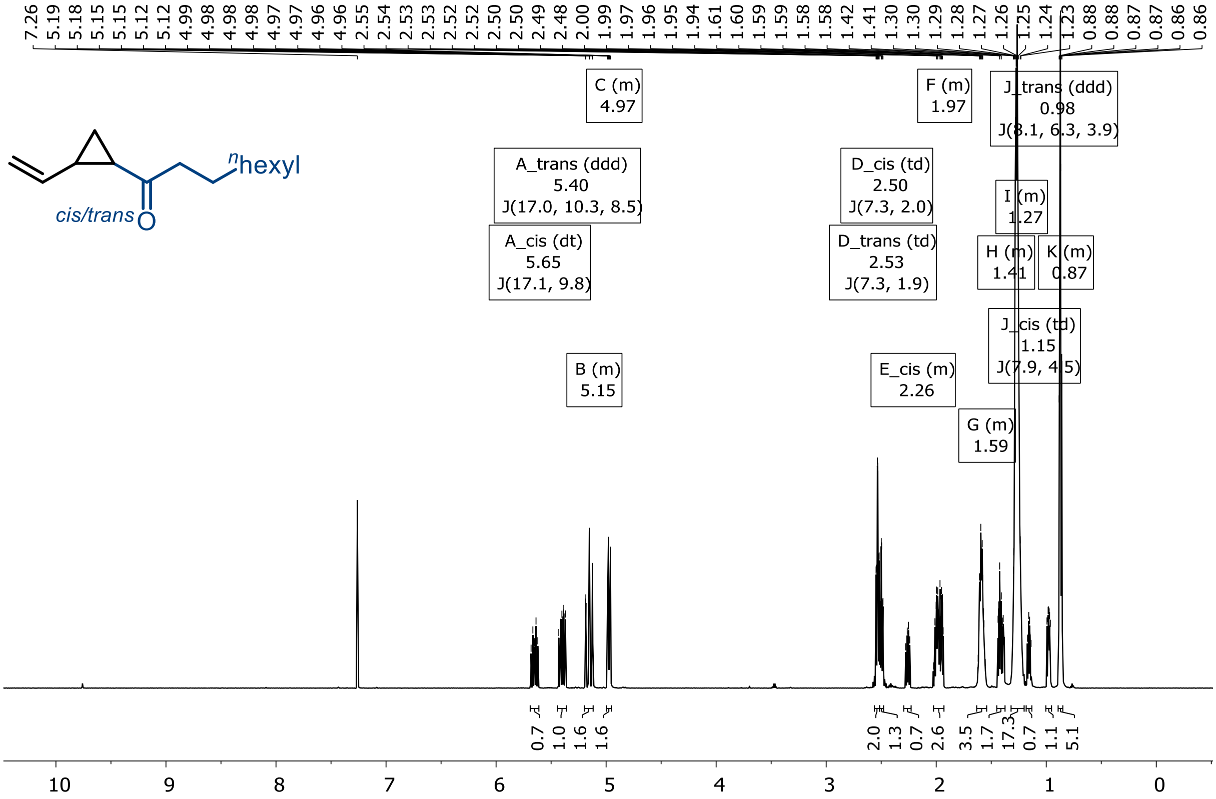

13C NMR
(151.00 MHz, CDCl3)

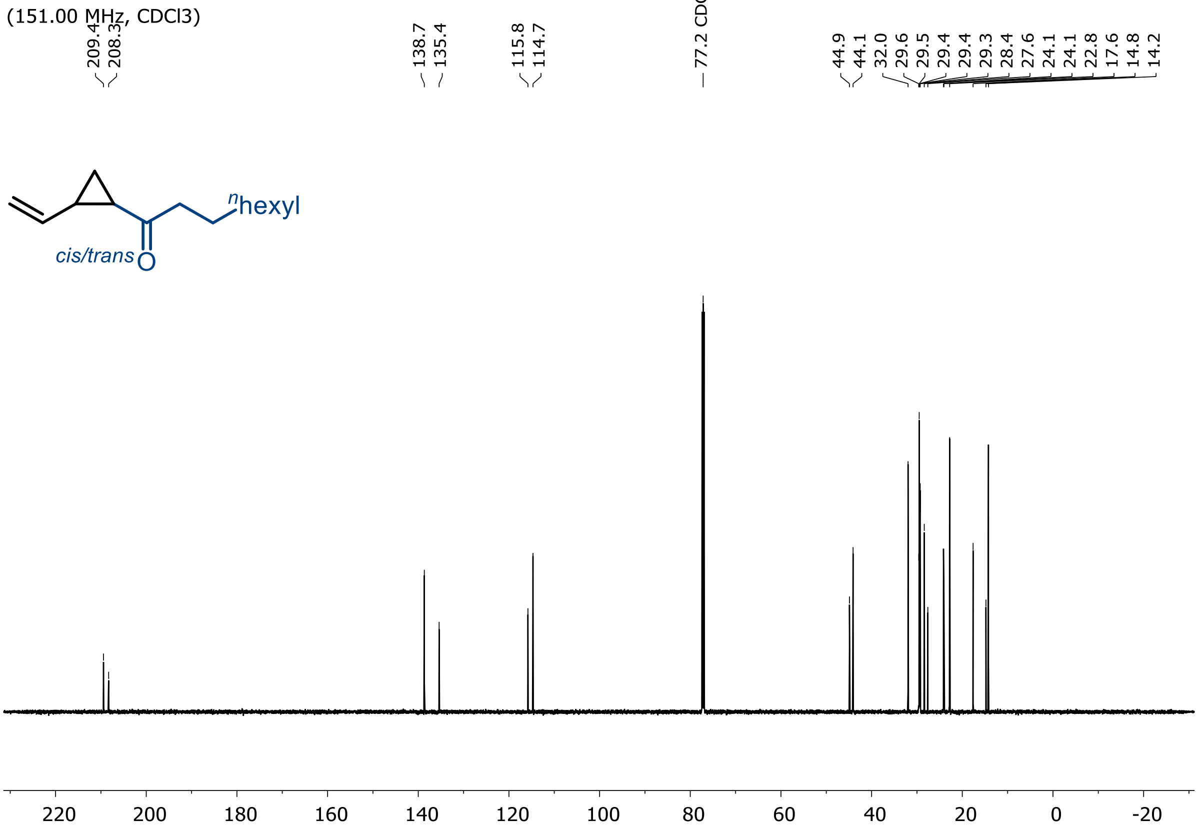

1-(*trans*-2-vinylcyclopropyl)nonan-1-one (12)

1H NMR
(600.44 MHz, CDCl3)

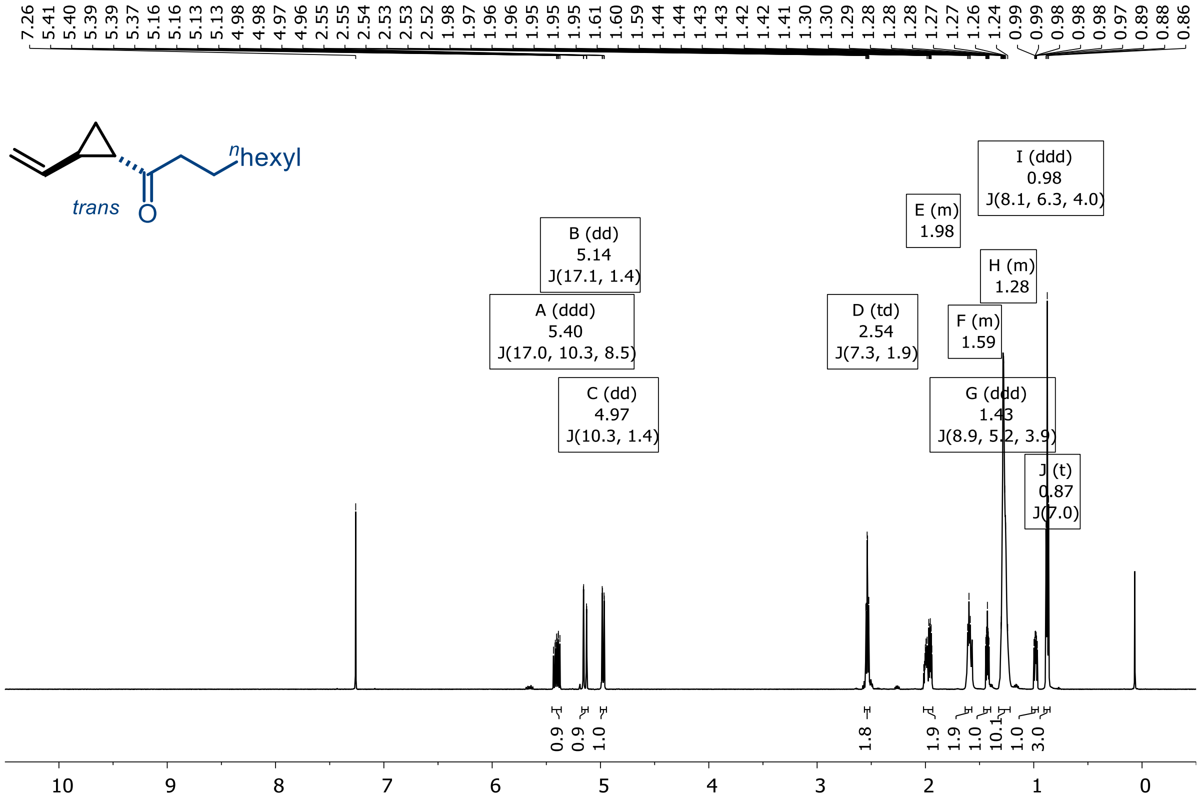

13C NMR
(151.00 MHz,)

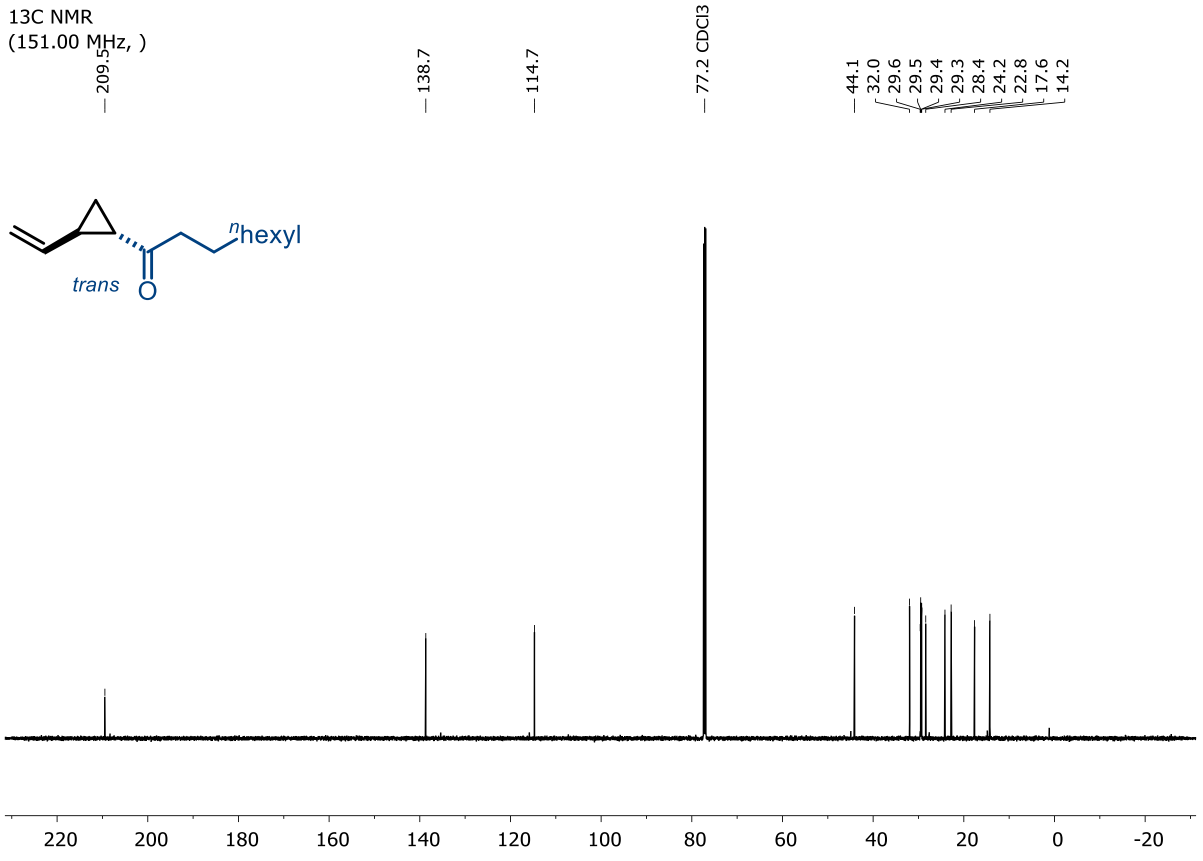

Naphthalen-1-yl 2-vinylcyclopropane-1-carboxylate (S13)

(399.97 MHz, CDCl3)

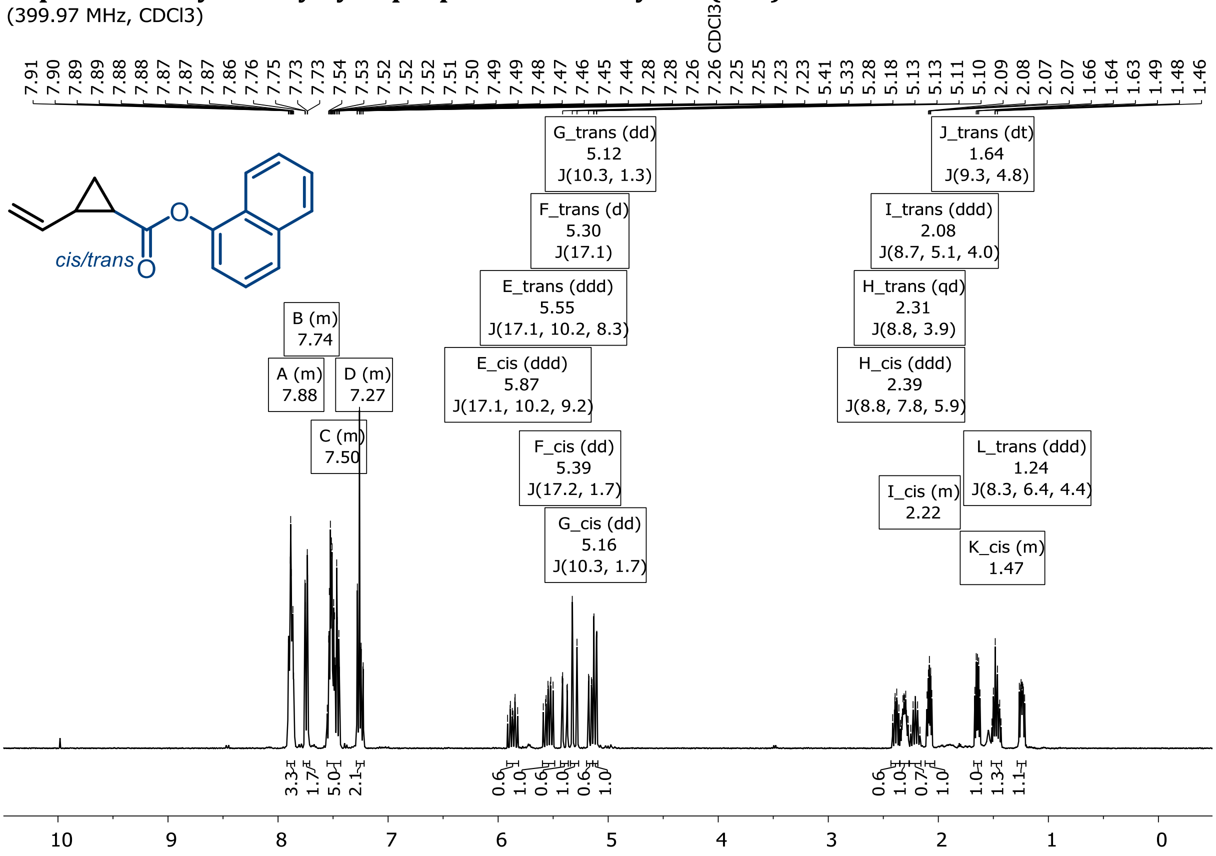

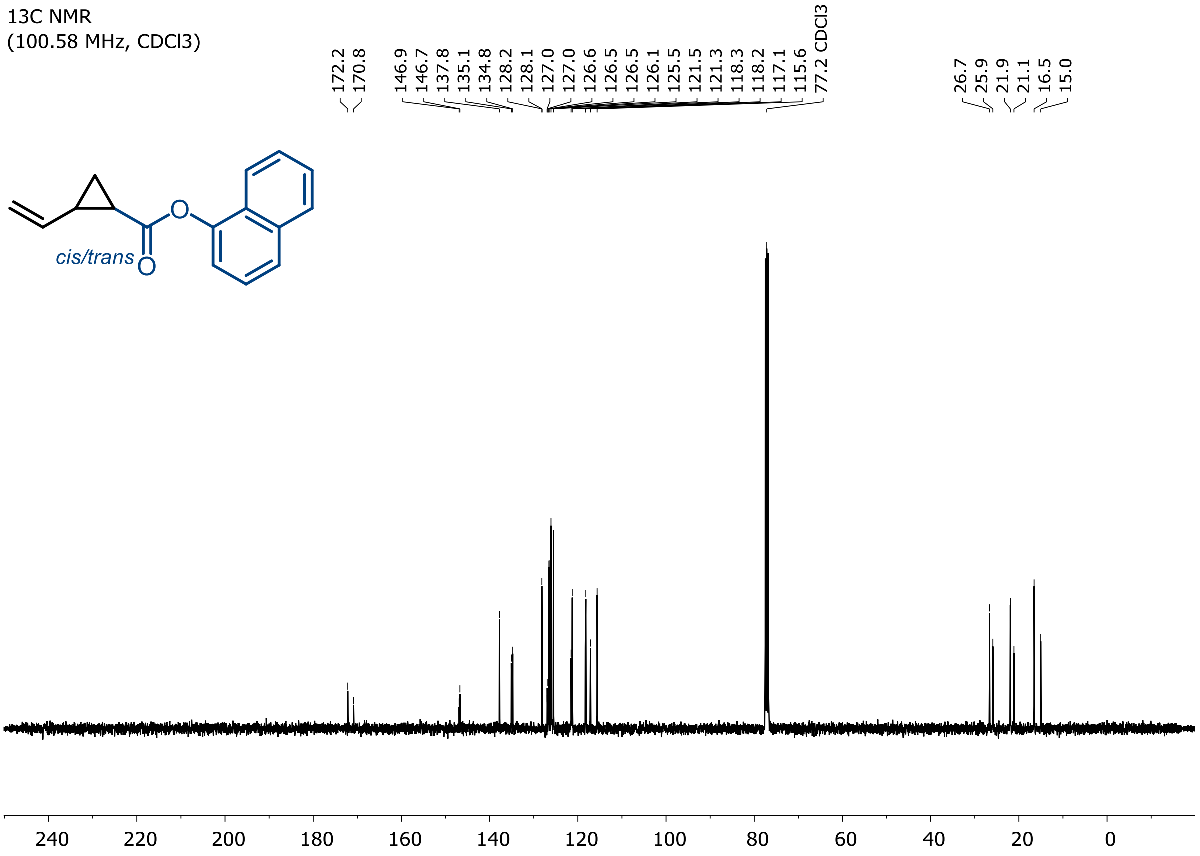

trans-Naphthalen-1-yl 2-vinylcyclopropane-1-carboxylate (13)

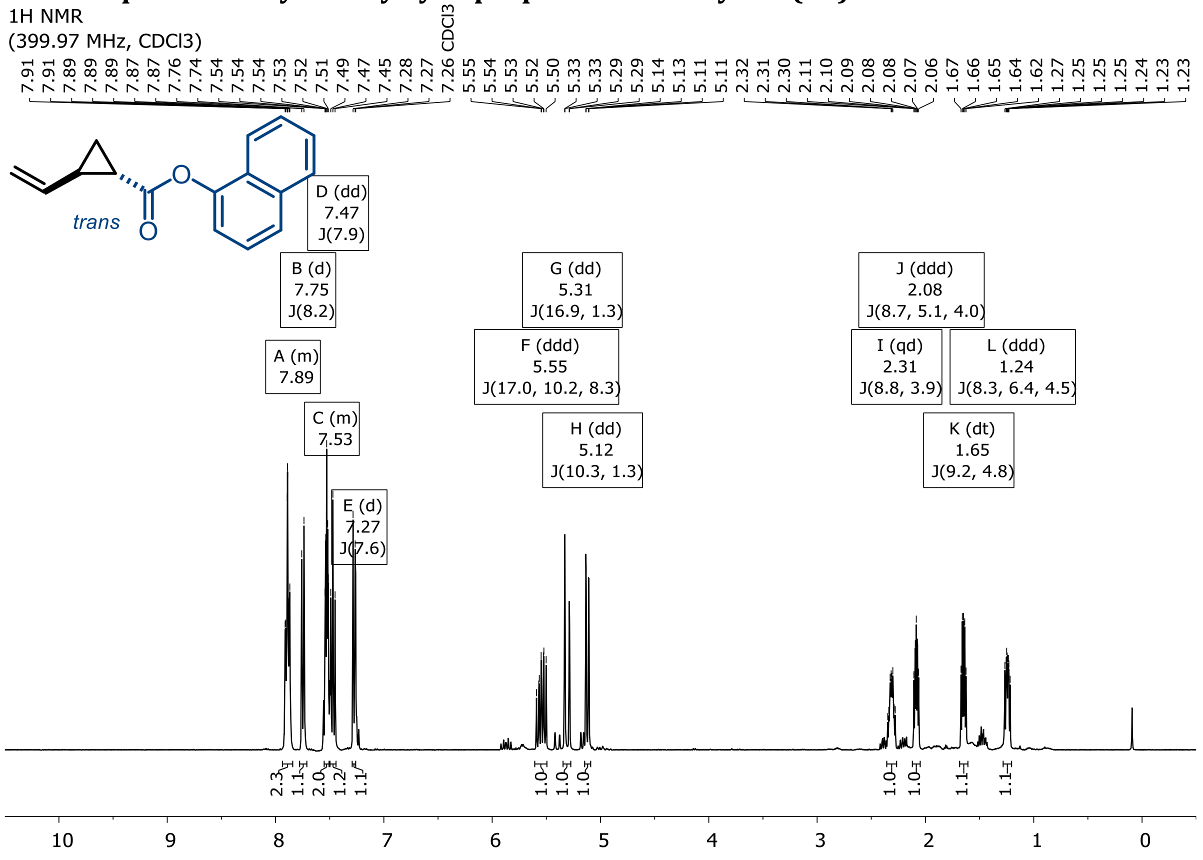

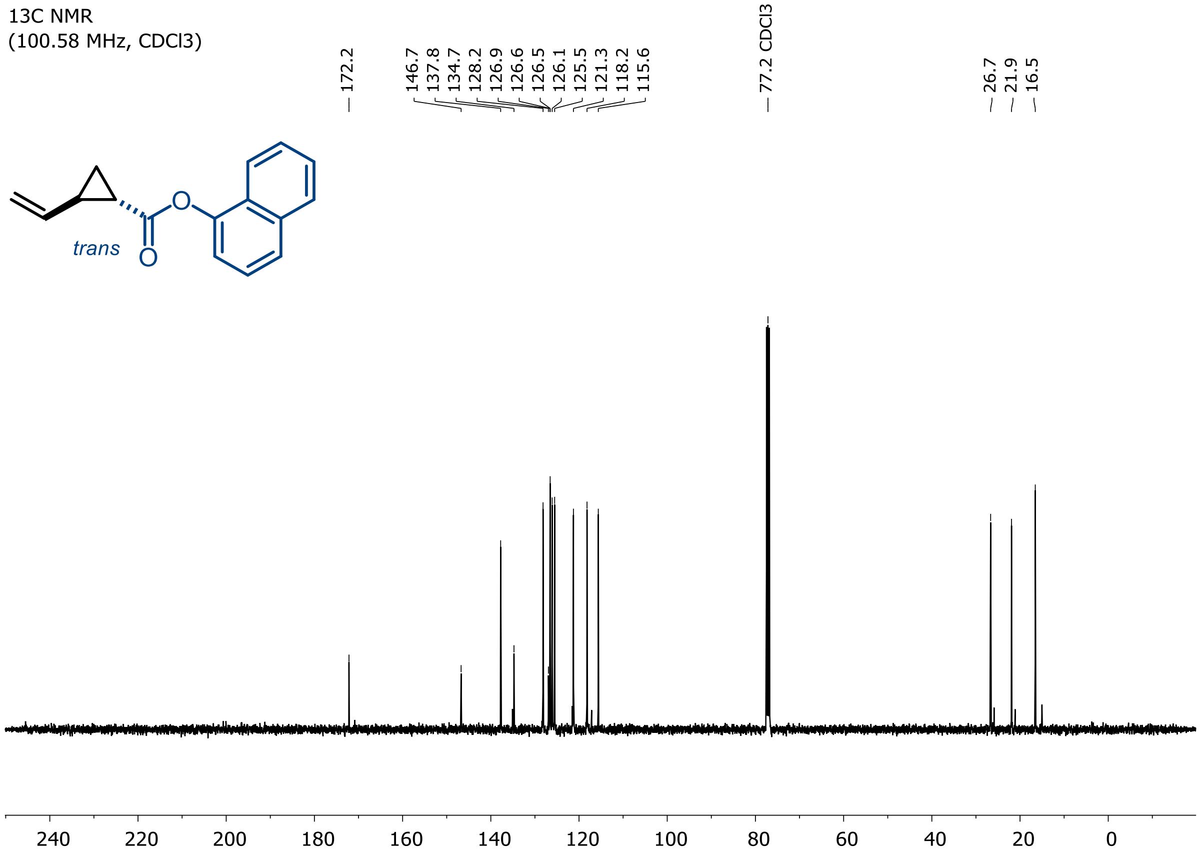

4-oxo-2-phenyl-4*H*-chromen-3-yl 2-vinylcyclopropane-1-carboxylate (S14)

1H NMR
(600.44 MHz, CDCl3)

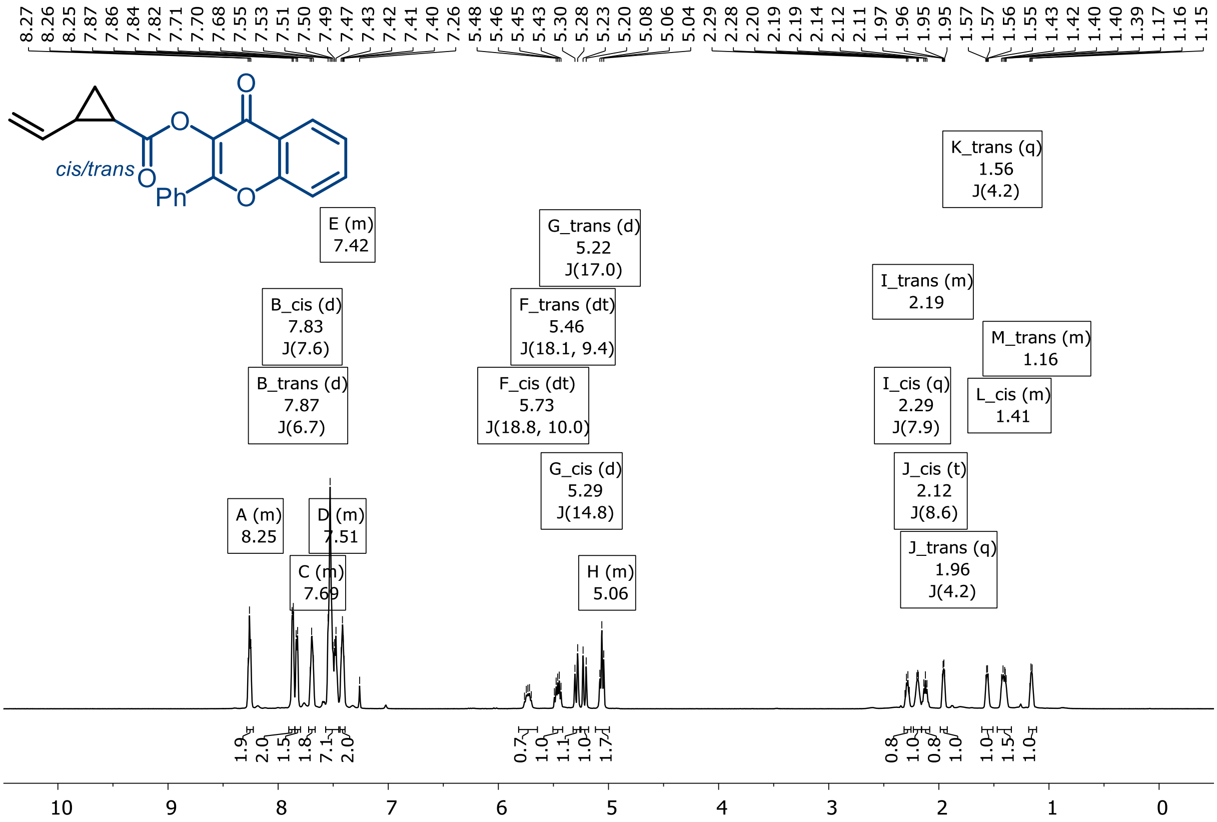

13C NMR
(151.00 MHz, CDCl3)

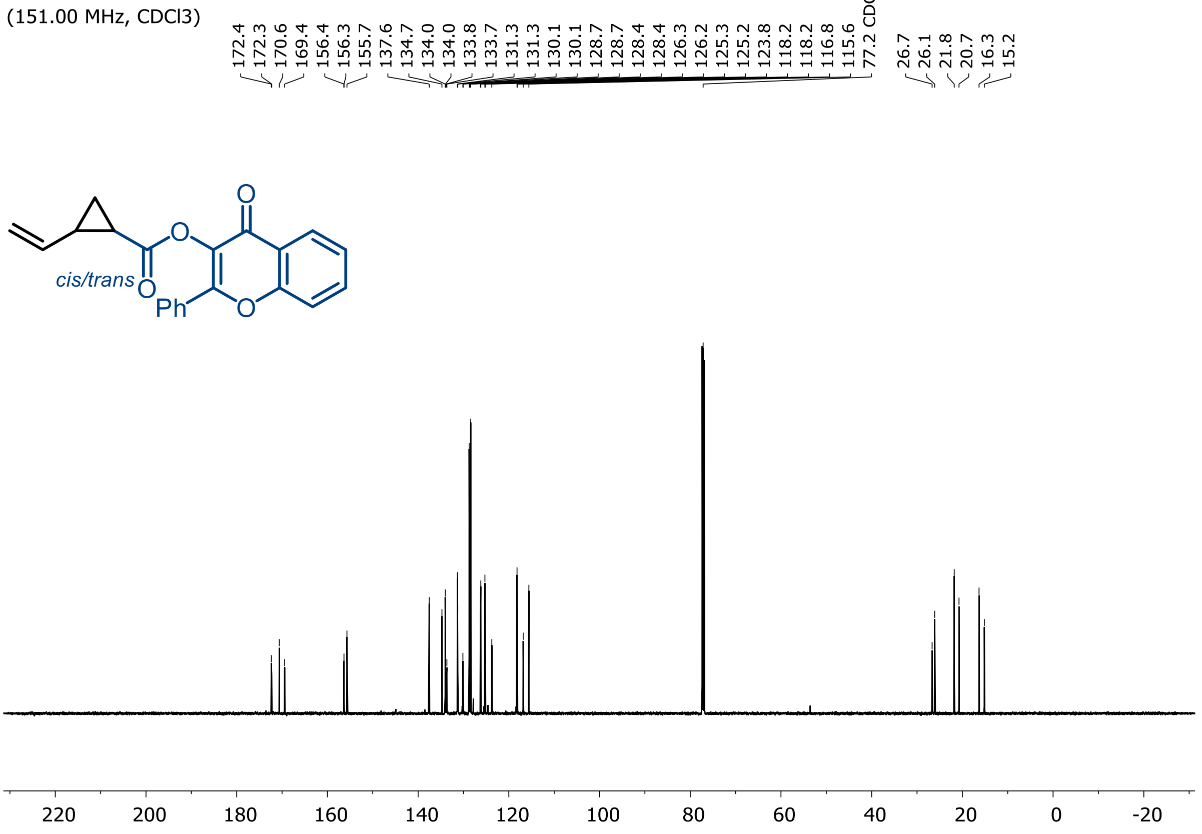

4-oxo-2-phenyl-4*H*-chromen-3-yl *trans*-2-vinylcyclopropane-1-carboxylate (14)

(600.44 MHz, CDCl3)

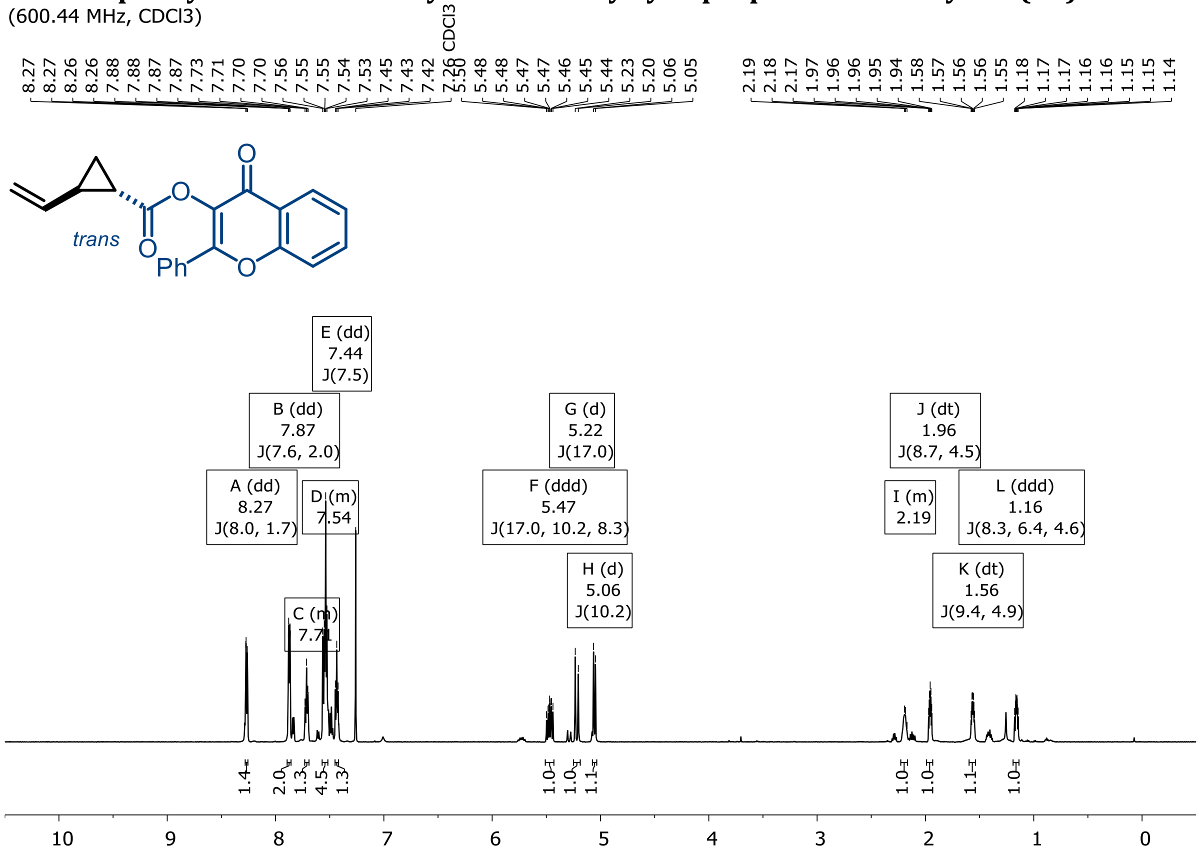

13C NMR
(151.00 MHz, CDCl3)

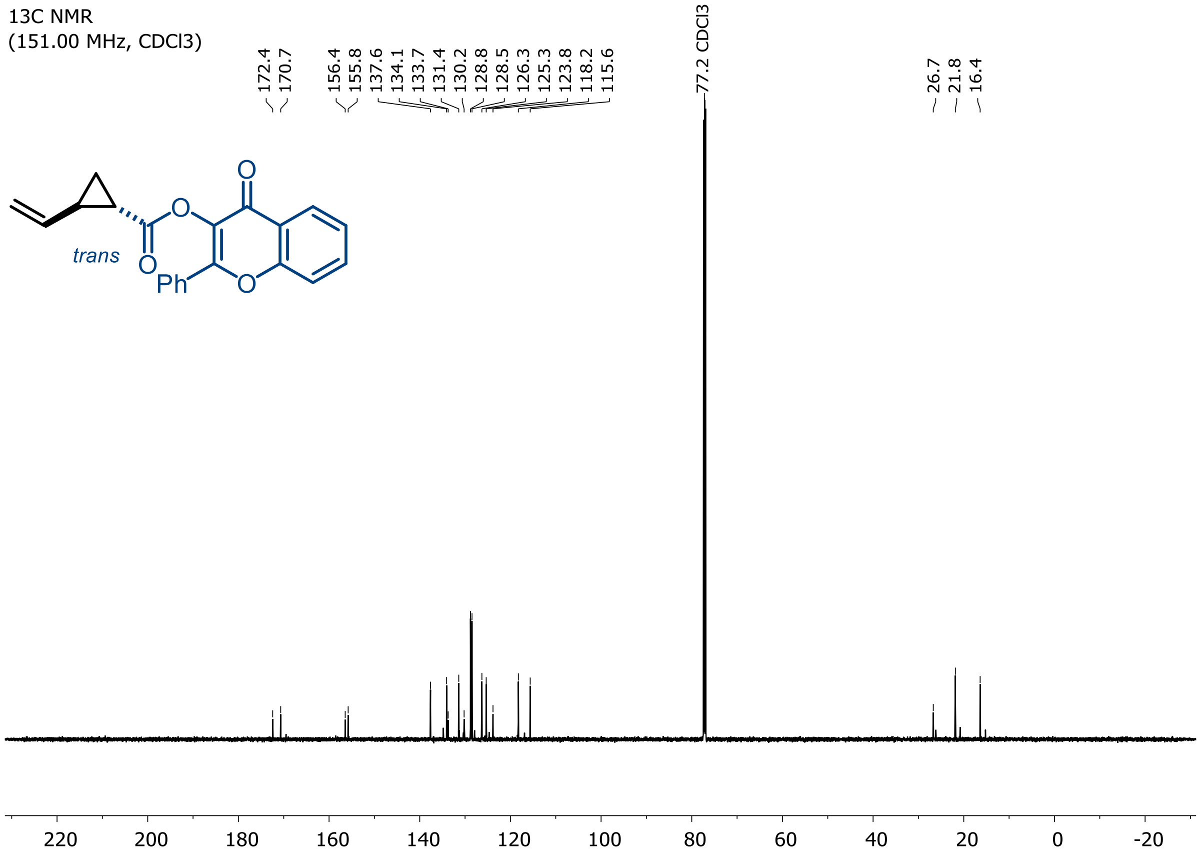

Octyl 2-vinylcyclopropane-1-carboxylate (S15)

1H NMR
(600.44 MHz, CDCl3)

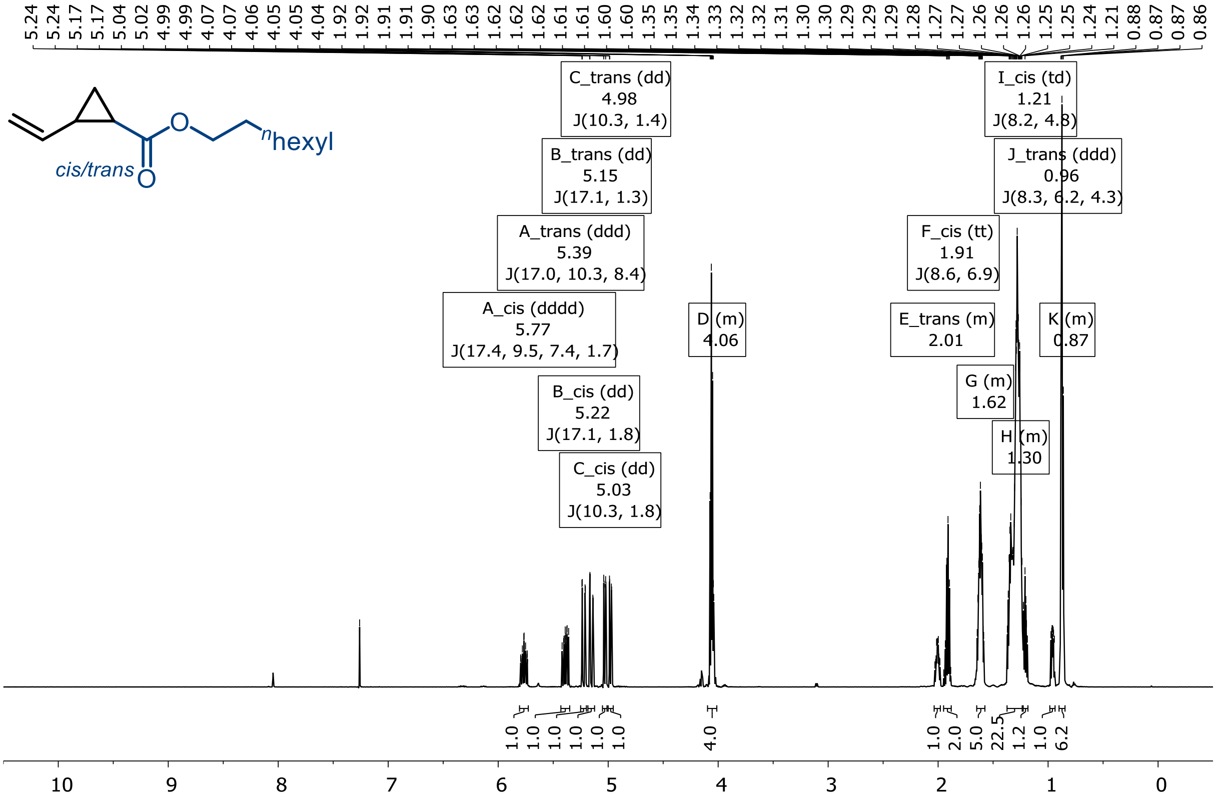

13C NMR
(151.00 MHz, CDCl3)

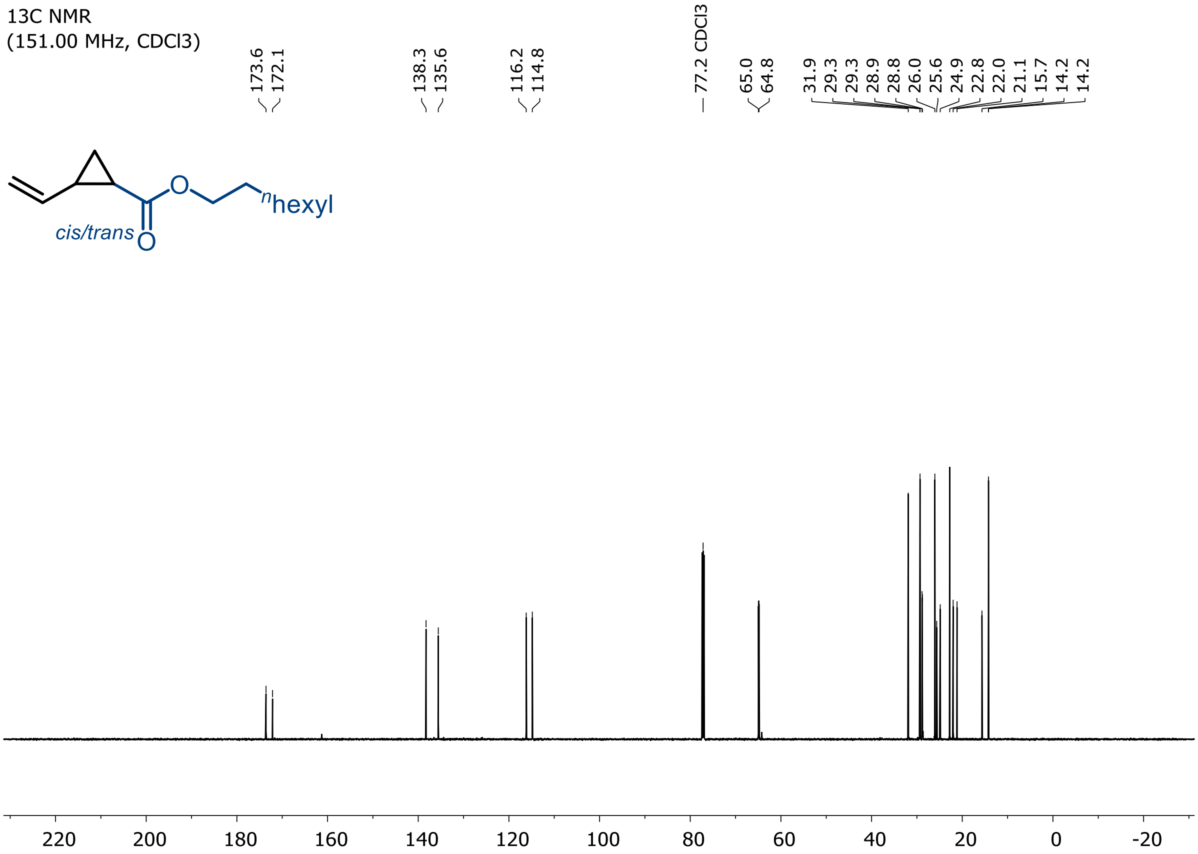

trans-Octyl 2-vinylcyclopropane-1-carboxylate (15)

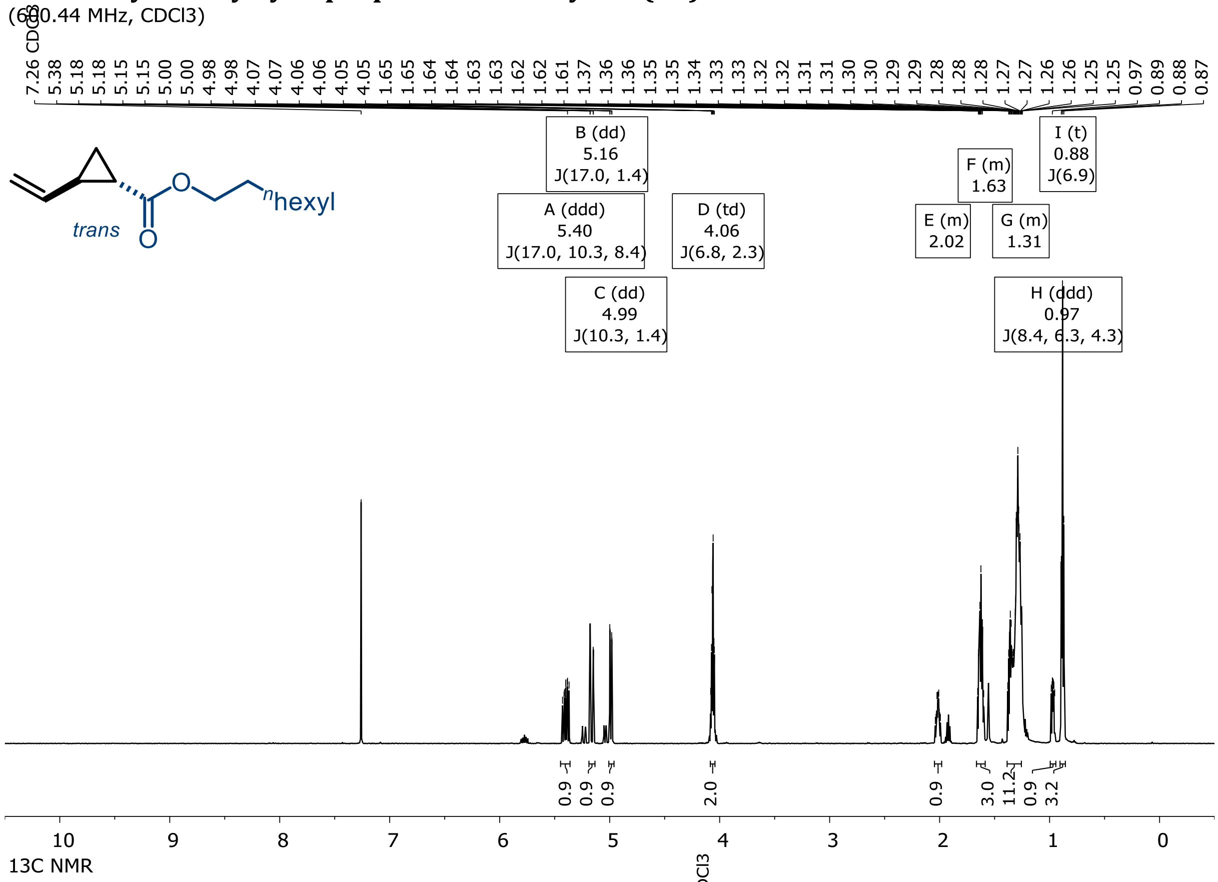

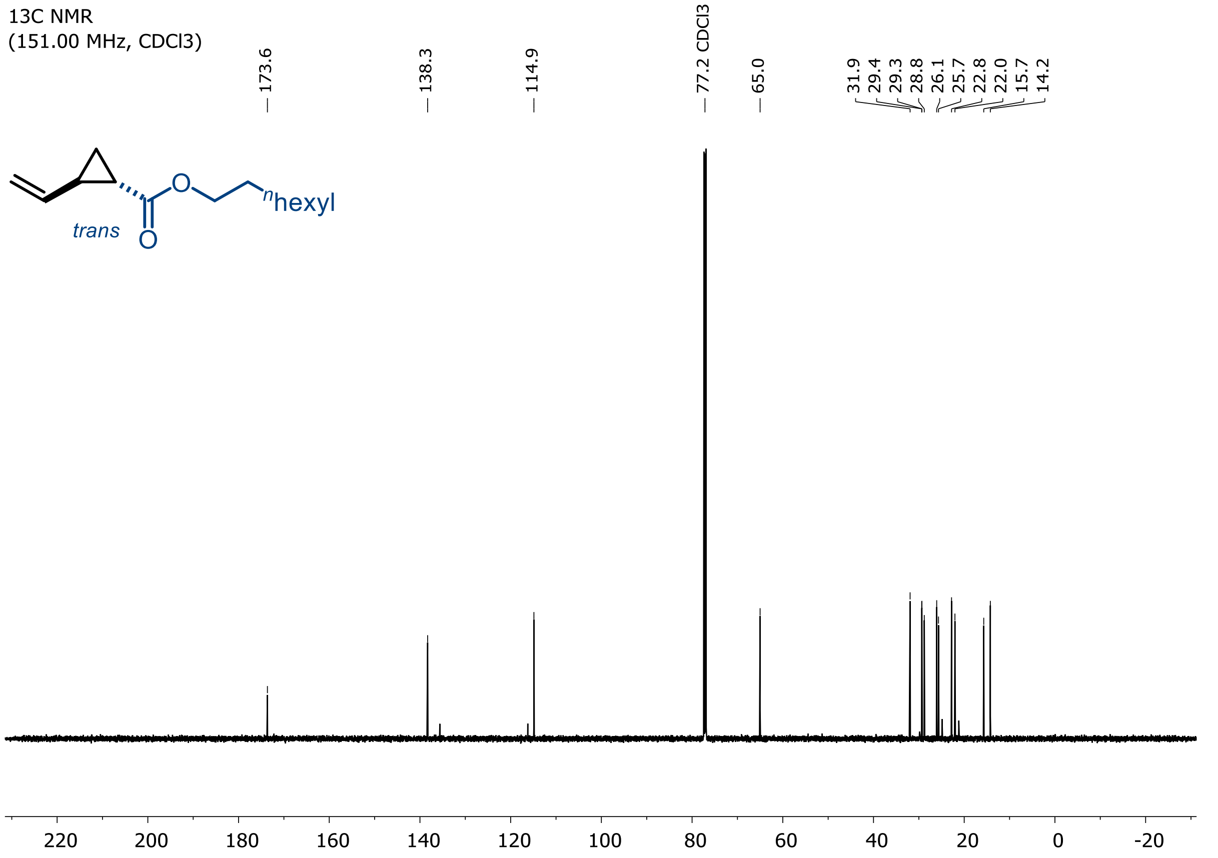

Ethyl 2-vinylcyclopropane-1-carboxylate (S16)

1H NMR
(600.44 MHz, CDCl3)

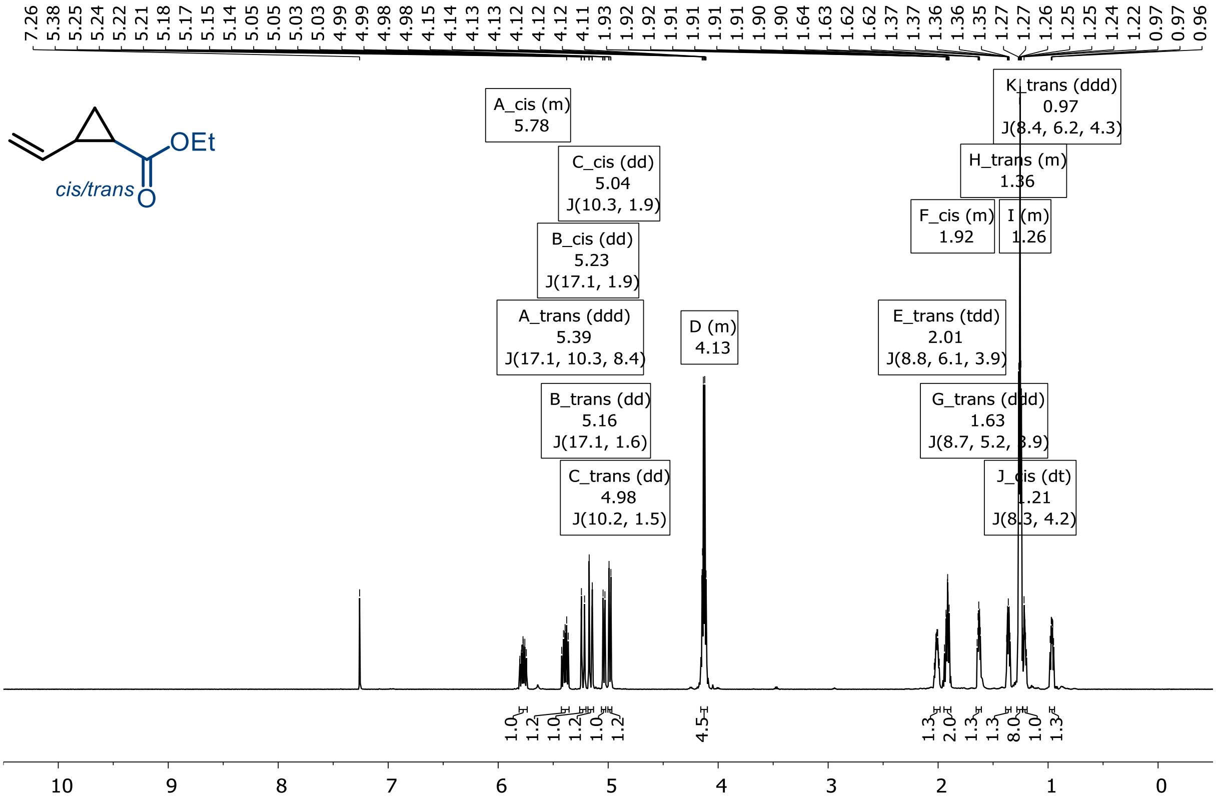

13C NMR
(151.00 MHz, CDCl3)

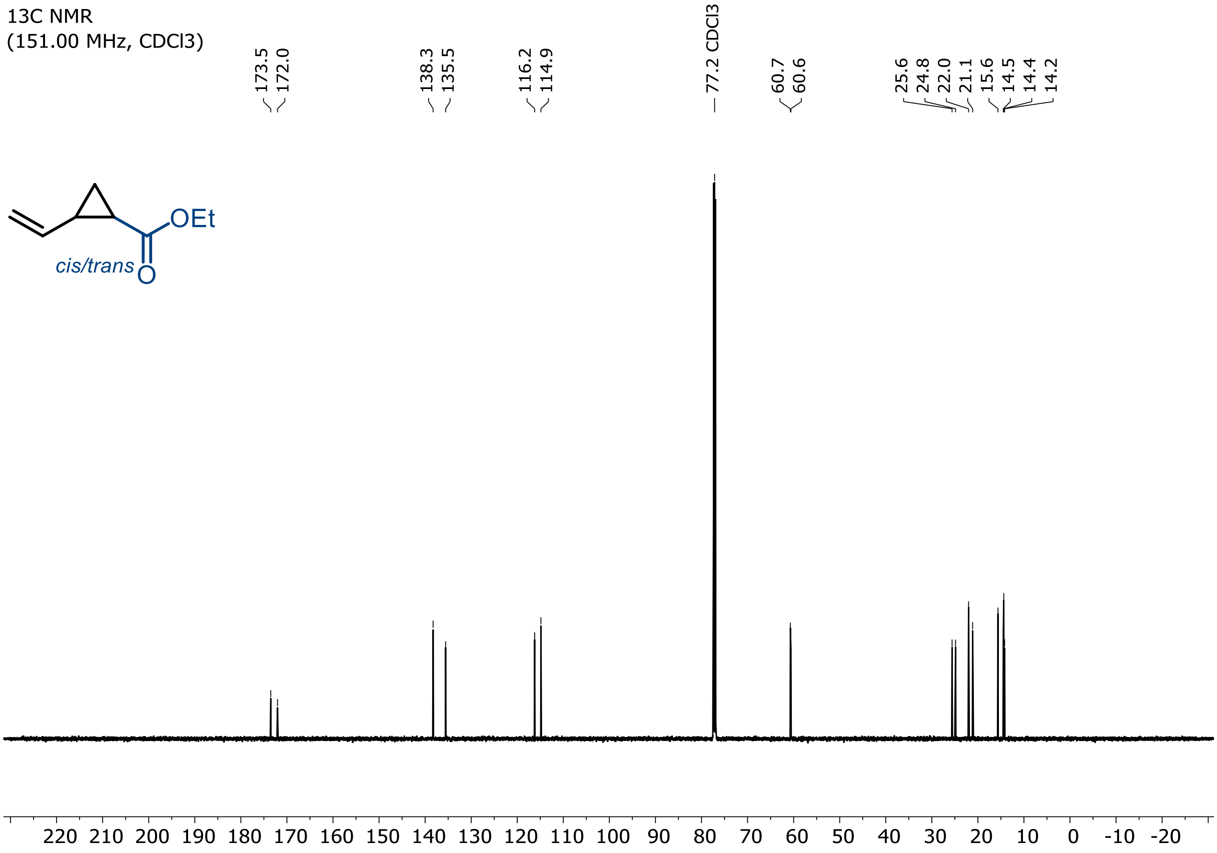

***trans*-Ethyl 2-vinylcyclopropane-1-carboxylate (16)**

1H NMR
(600.44 MHz, CDCl3)

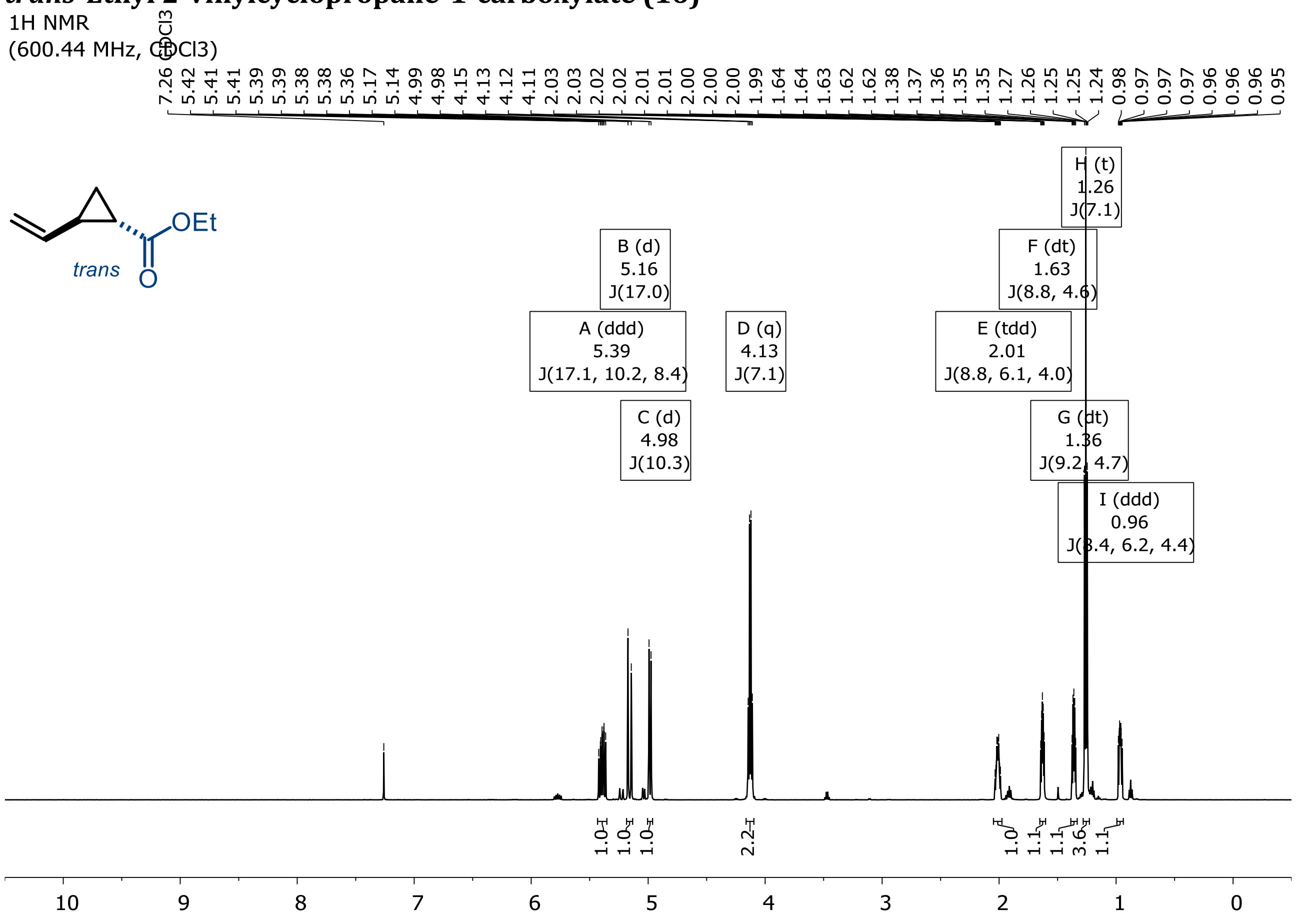

13C NMR
(151.00 MHz, CDCl3)

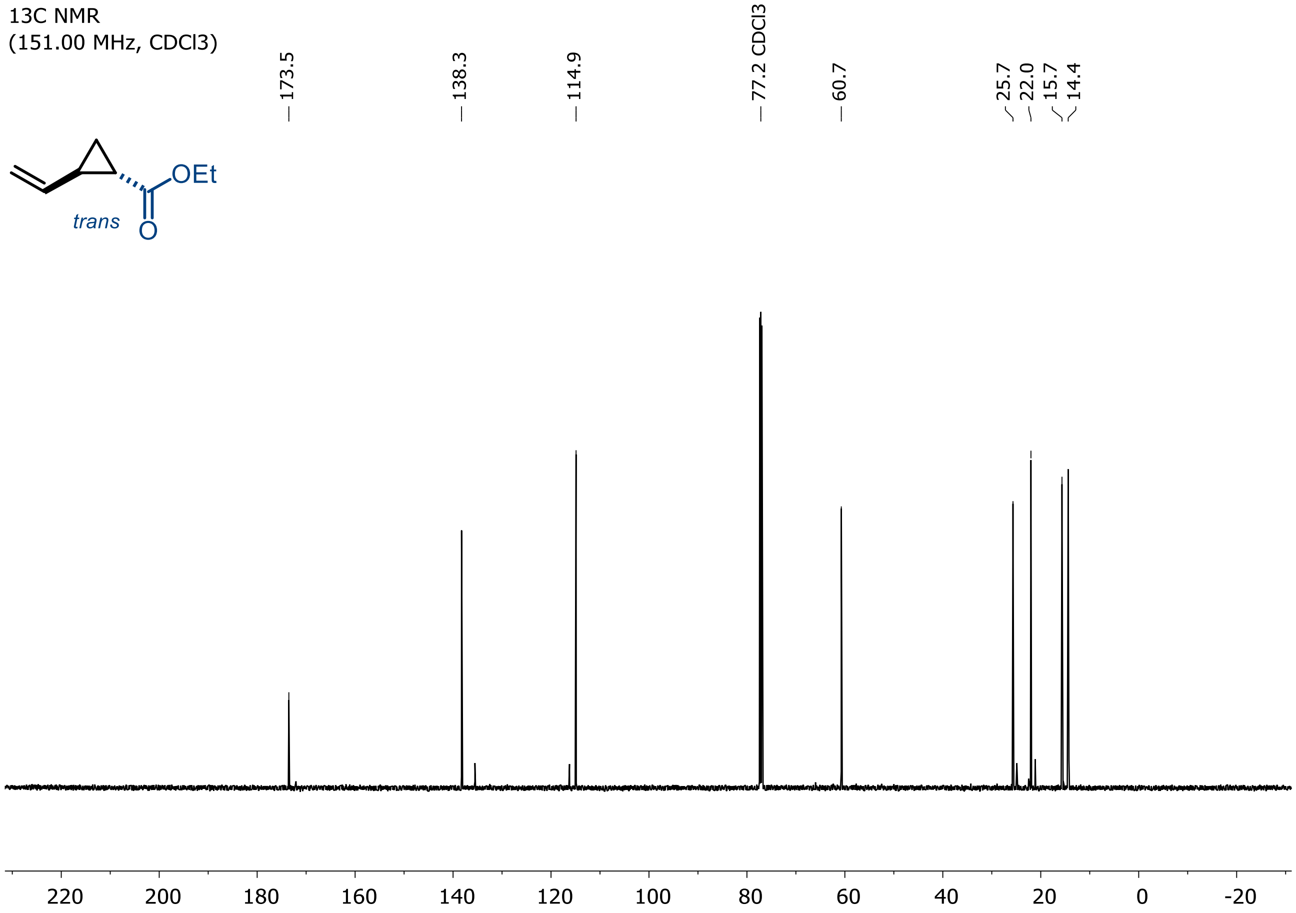

2-vinylcyclopropane-1-carboxylic acid (S17)

1H NMR
(599.86 MHz, CDCl3)

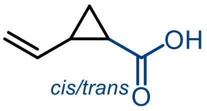

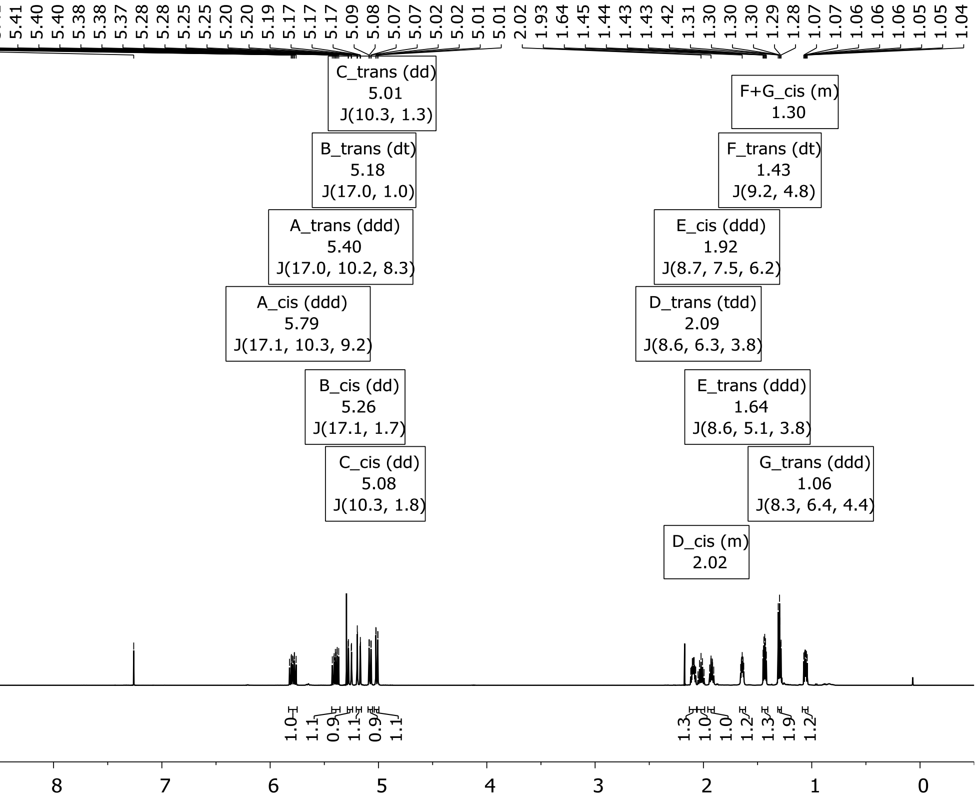

13C NMR
(150.85 MHz, CDCl3)

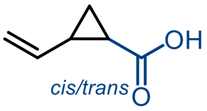

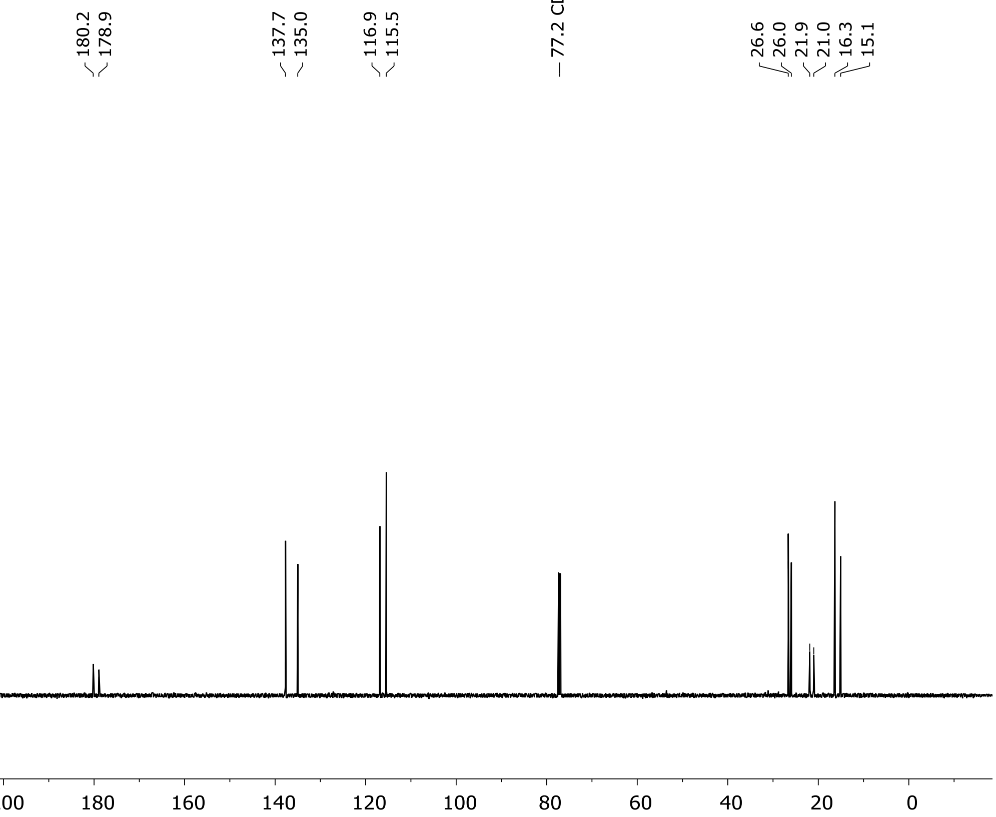

***trans*-2-vinylcyclopropane-1-carboxylic acid (17)**

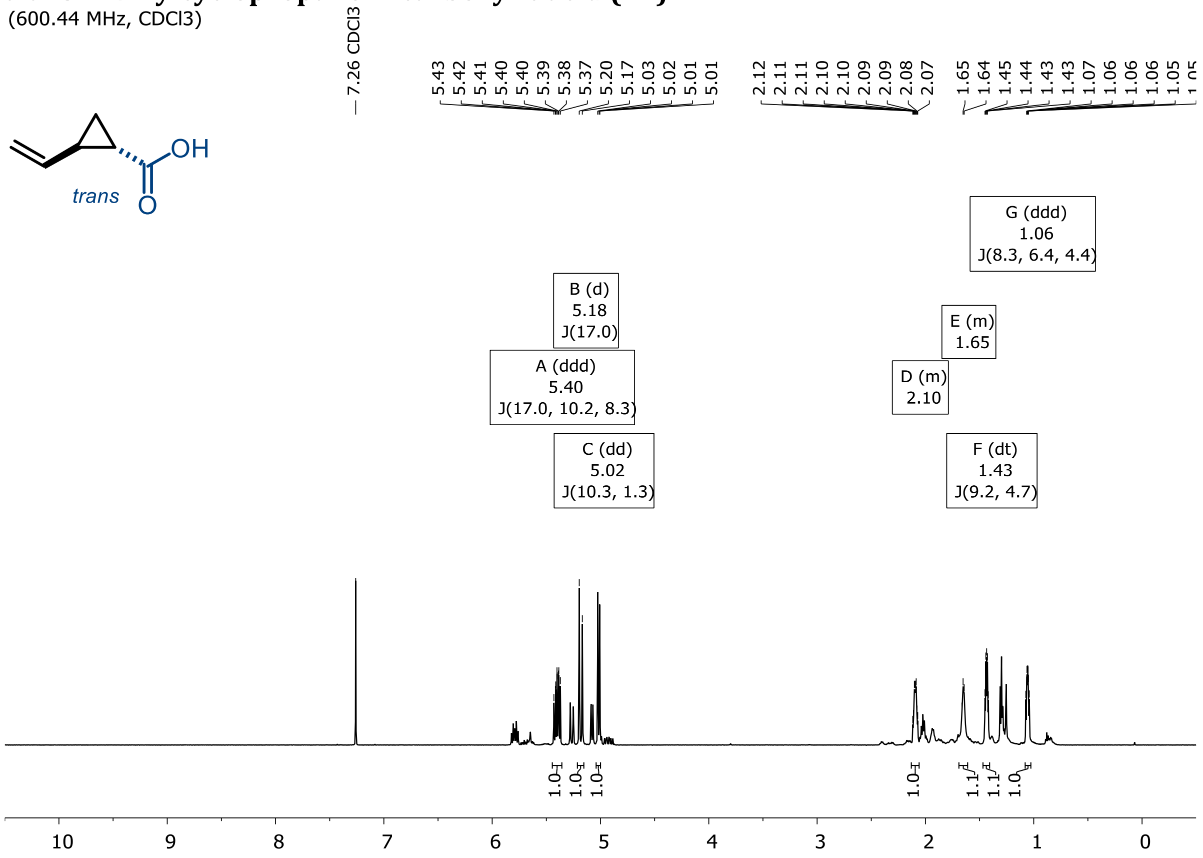

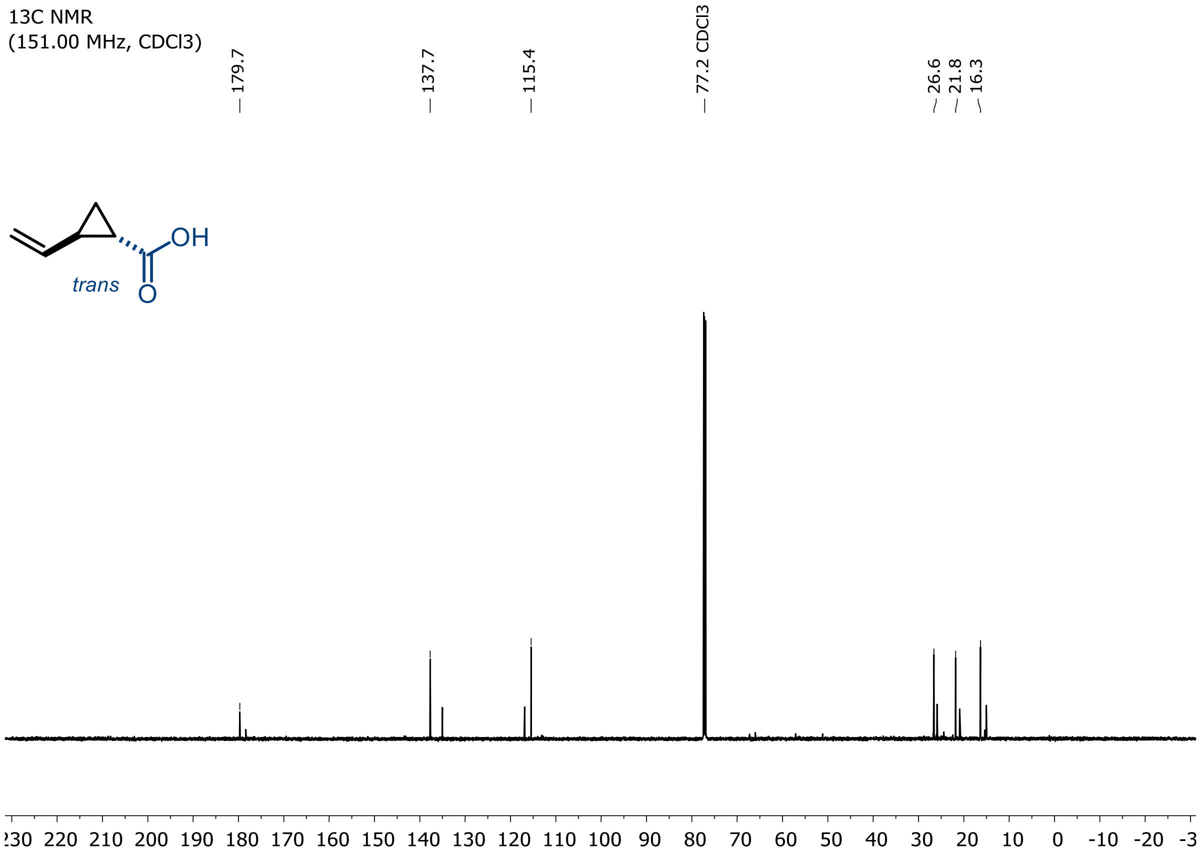

Ethyl 2,2-dimethyl-3-vinylcyclopropane-1-carboxylate

1H NMR
(600.44 MHz, CDCl3)

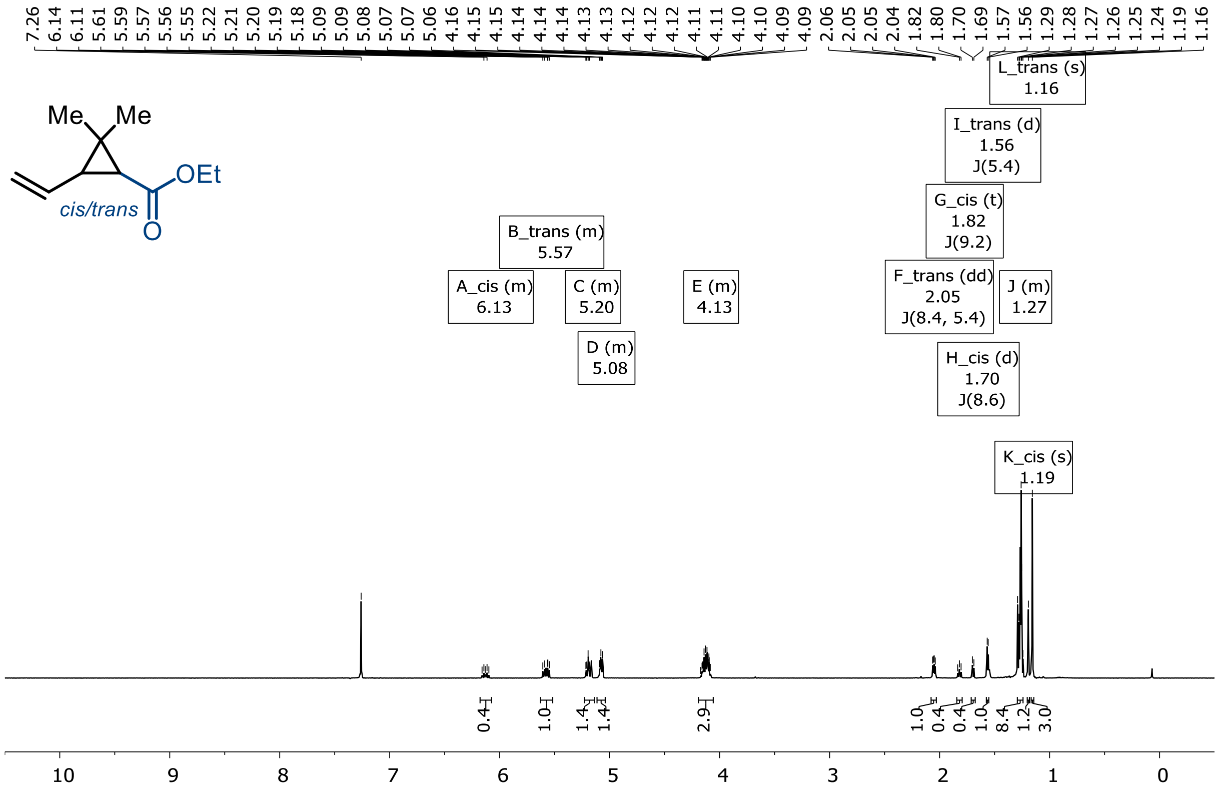

13C NMR
(151.00 MHz, CDCl3)

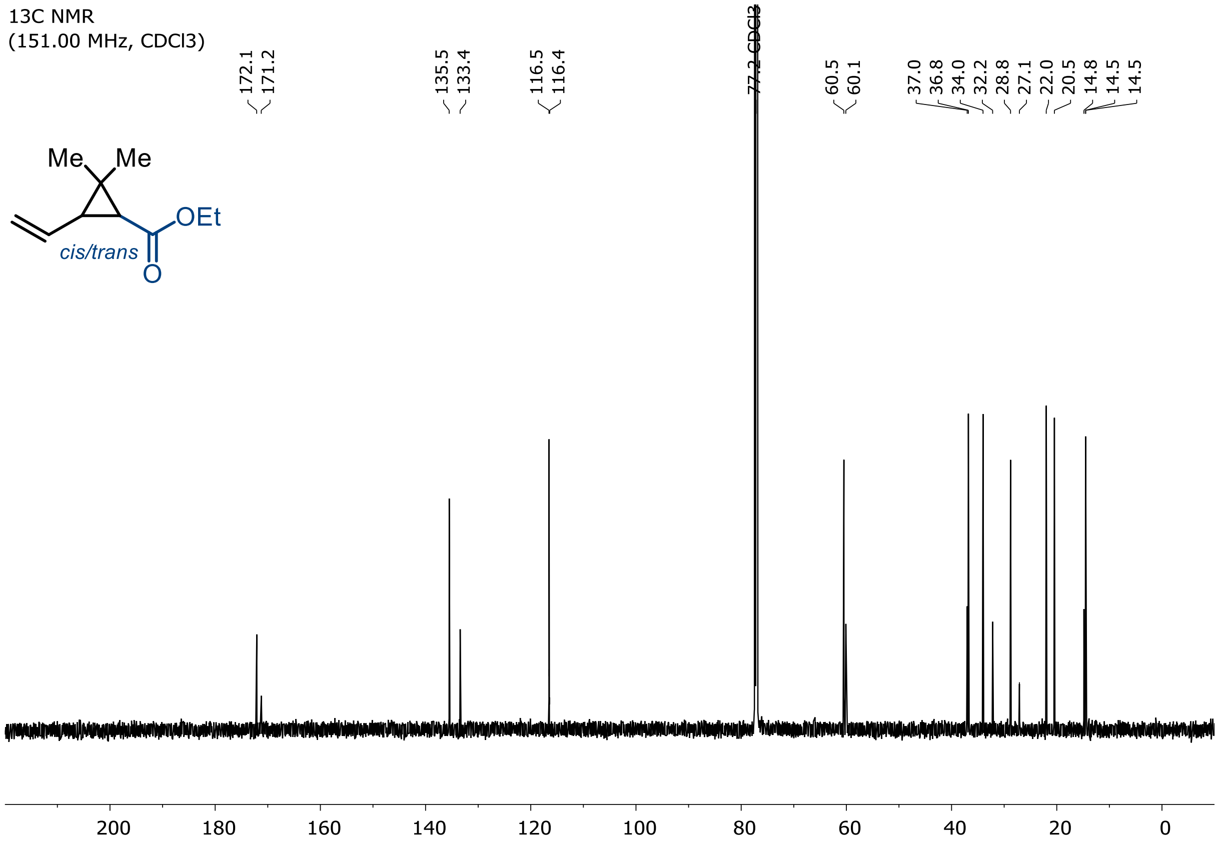

Ethyl *cis*-2,2-dimethyl-3-vinylcyclopropane-1-carboxylate (S18)

1H NMR
(600.44 MHz, CDCl3)

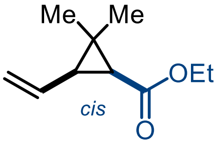

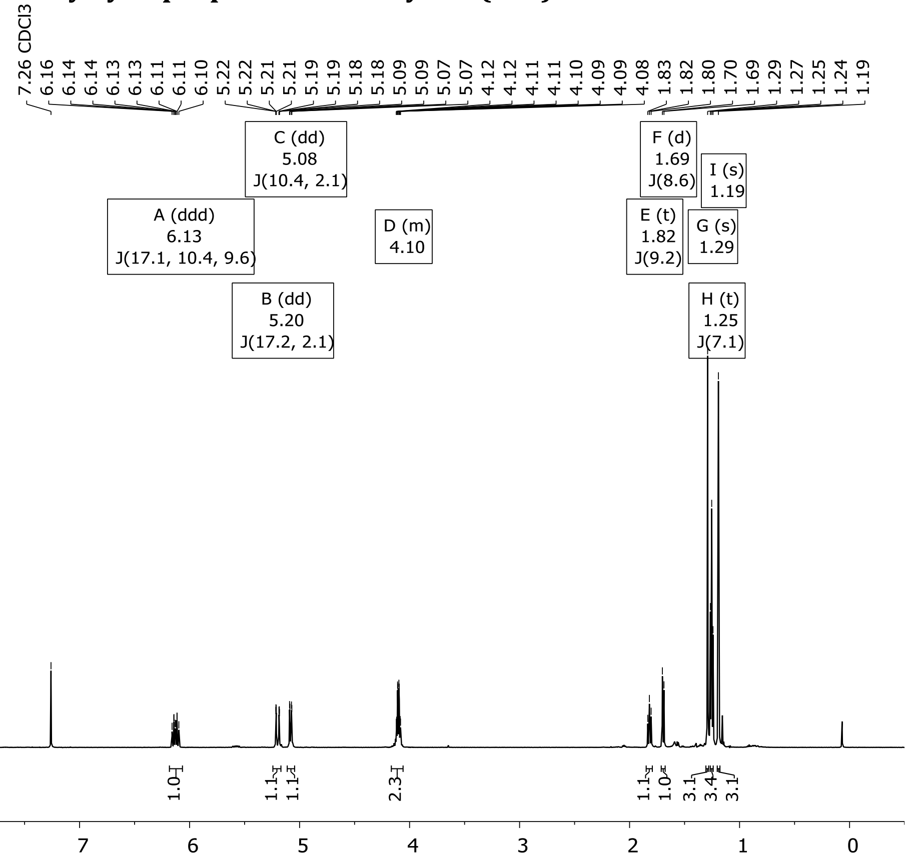

13C NMR
(151.00 MHz, CDCl3)

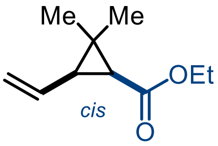

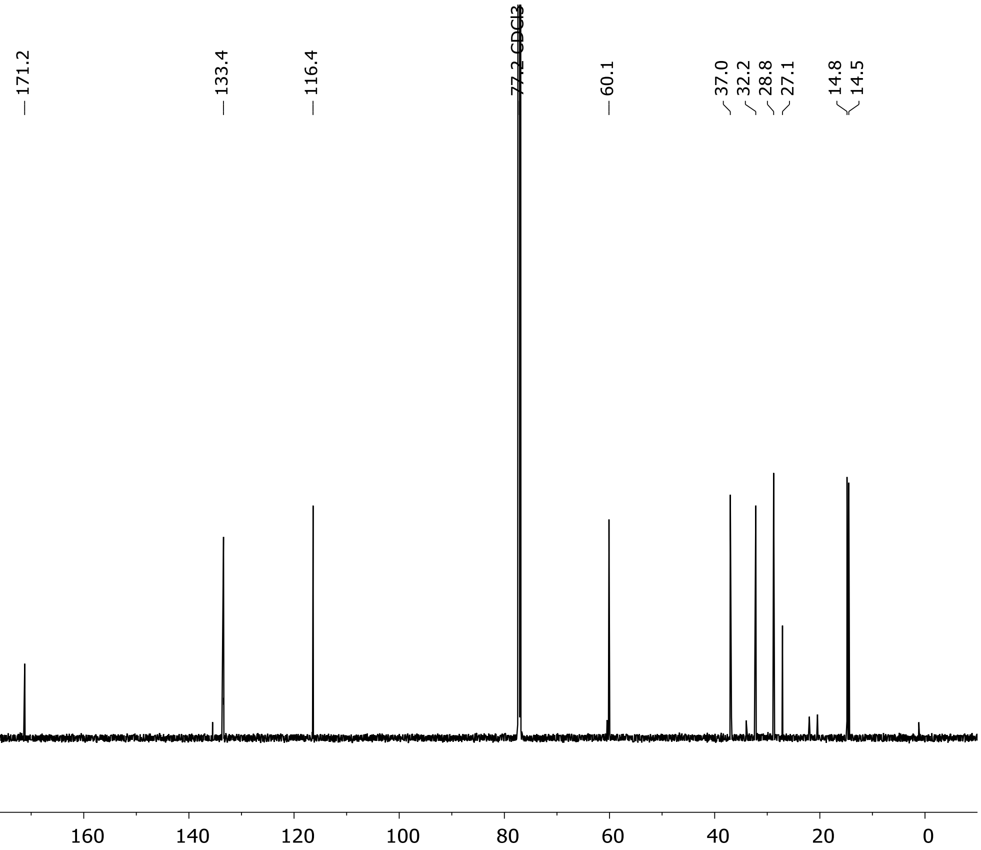

Ethyl *trans*-2,2-dimethyl-3-vinylcyclopropane-1-carboxylate (18)

1H NMR
(600.44 MHz, CDCl3)

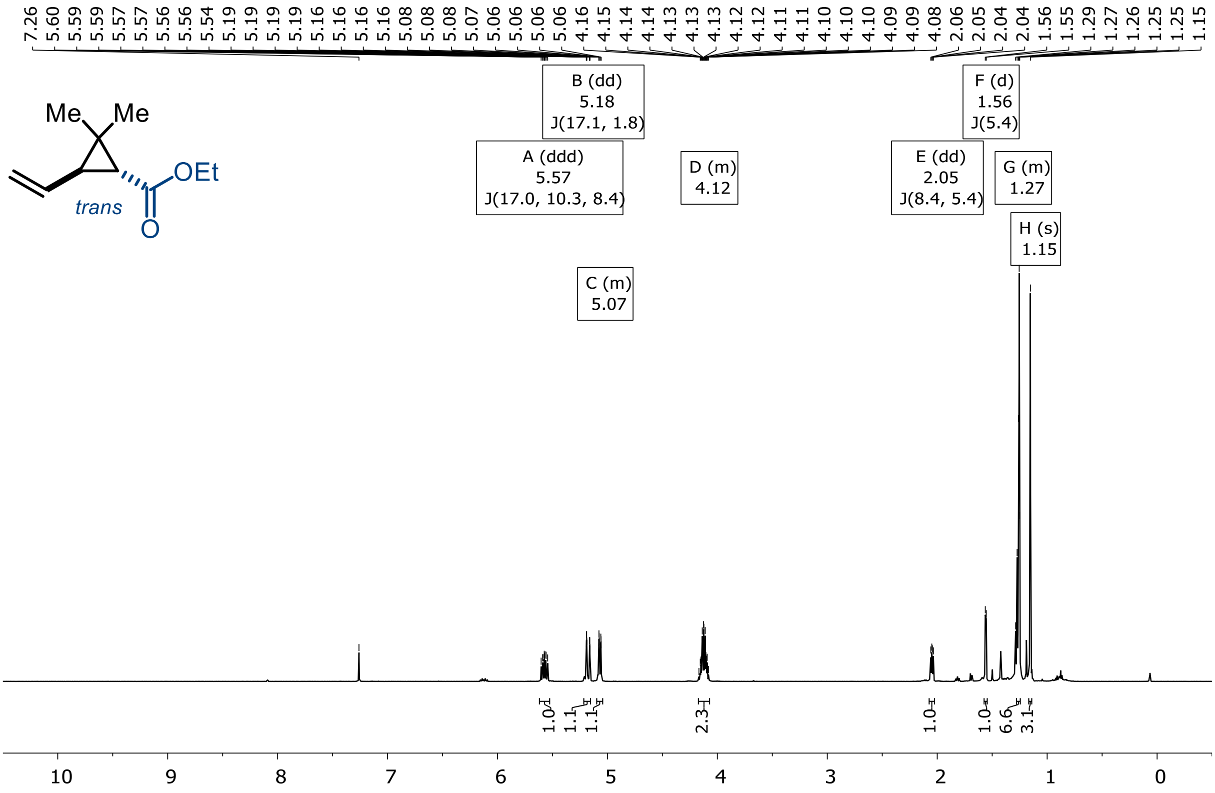

13C NMR
(151.00 MHz, CDCl3)

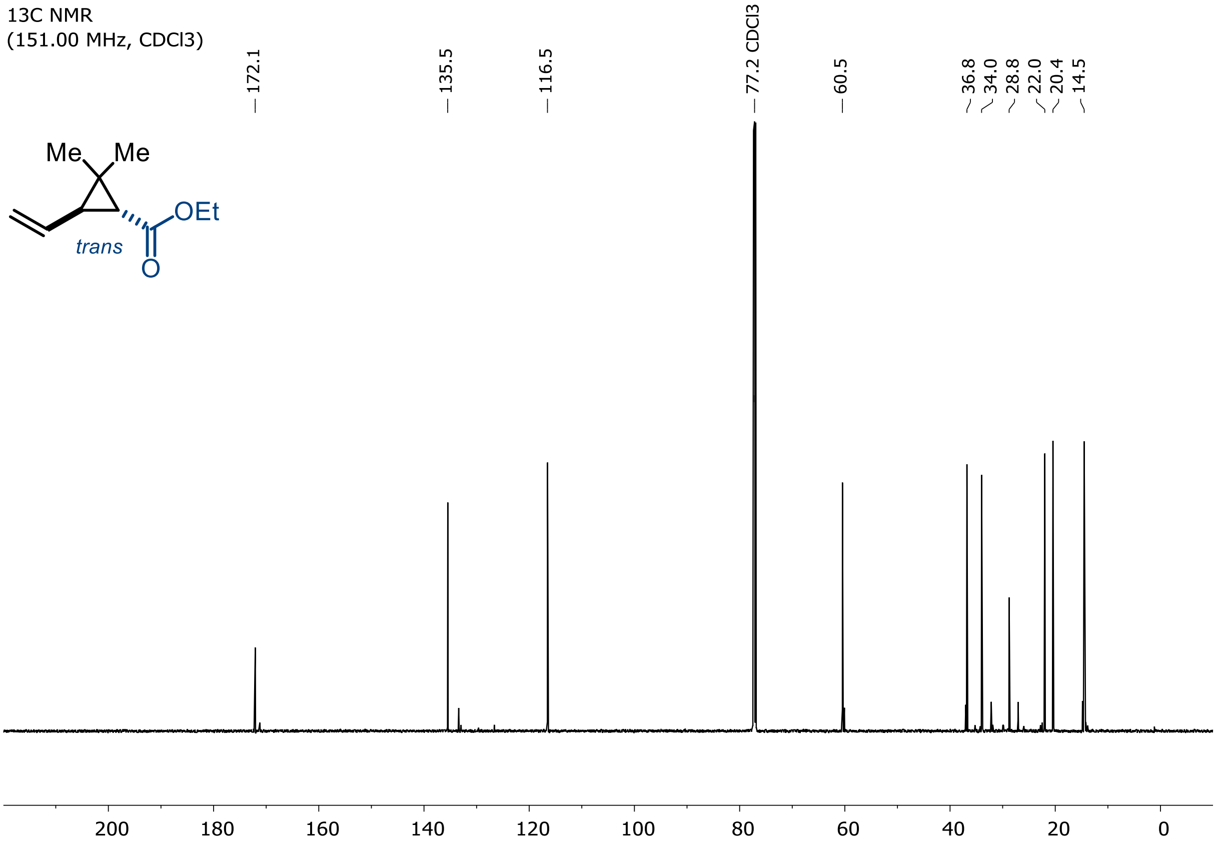

Ethyl *cis*-2-methyl-2-(prop-1-en-2-yl)cyclopropane-1-carboxylate (*S*19)

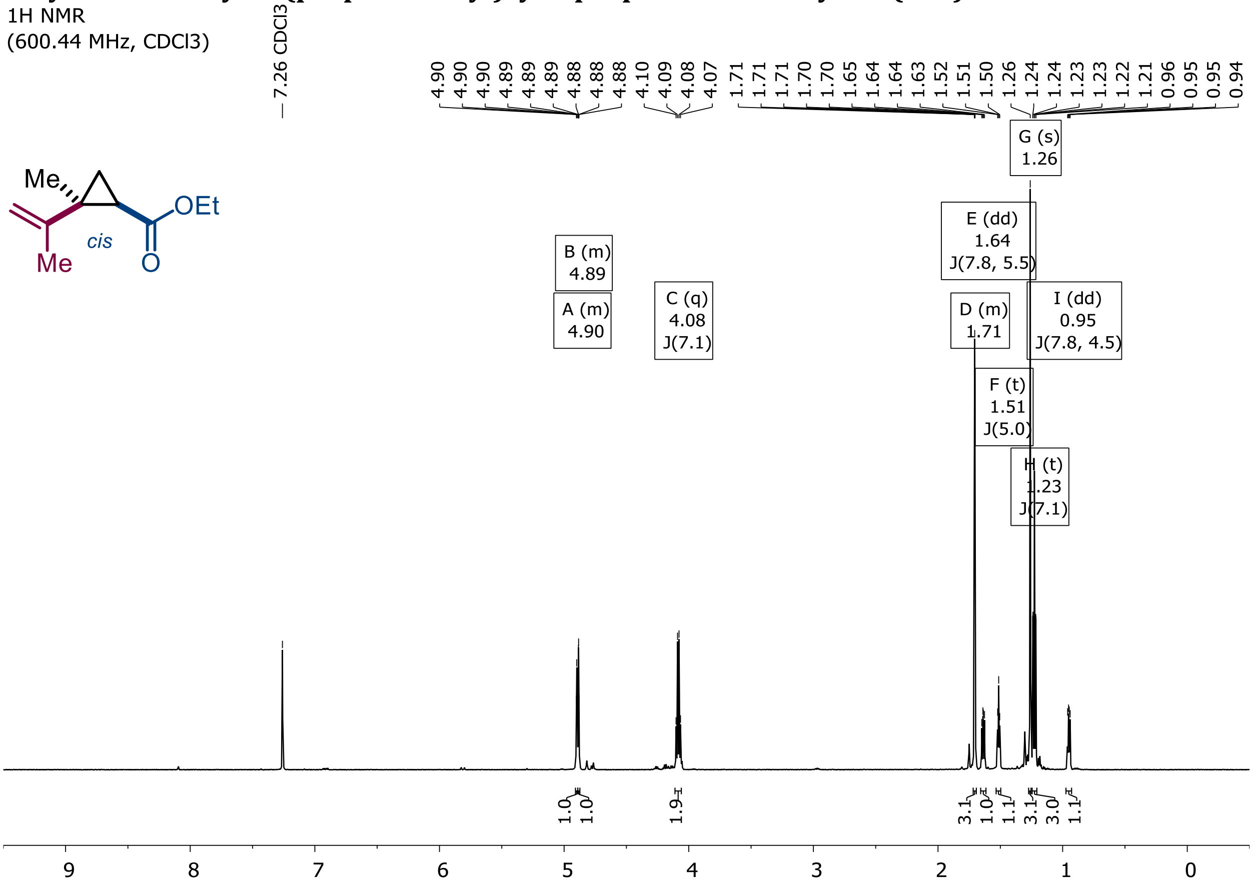

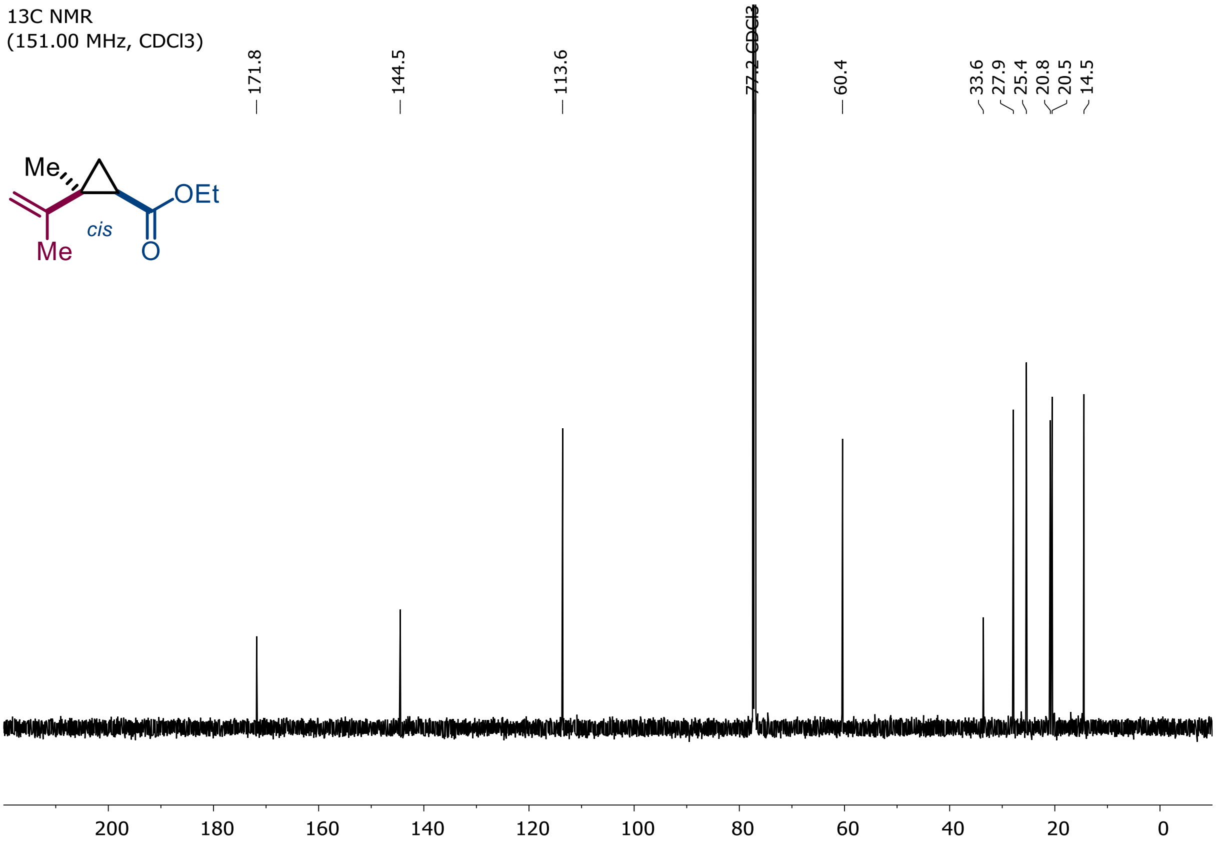

Ethyl *trans*-2-methyl-2-(prop-1-en-2-yl)cyclopropane-1-carboxylate (19)

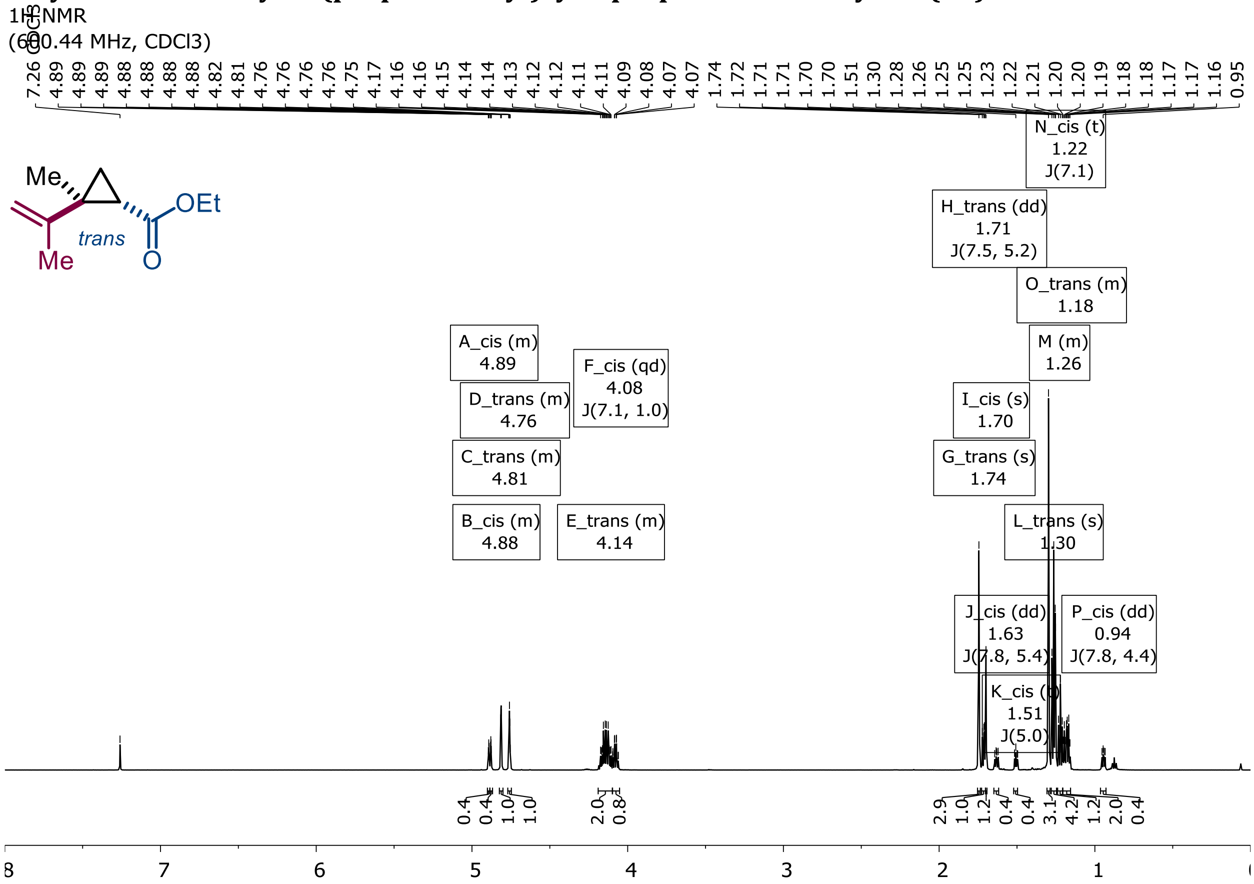

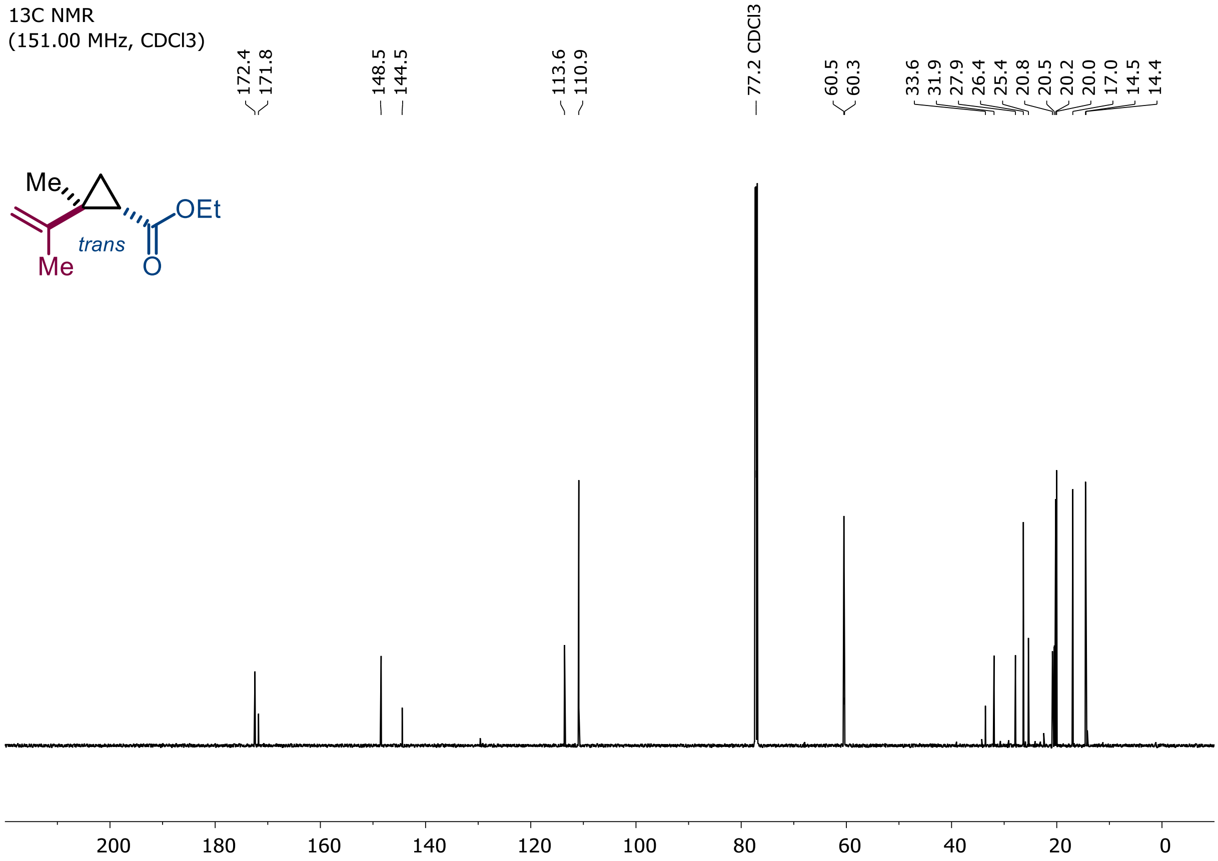

1-(5-methoxynonan-5-yl)-2-vinylcyclopropane (S20)

1H NMR
(399.97 MHz, CDCl3)

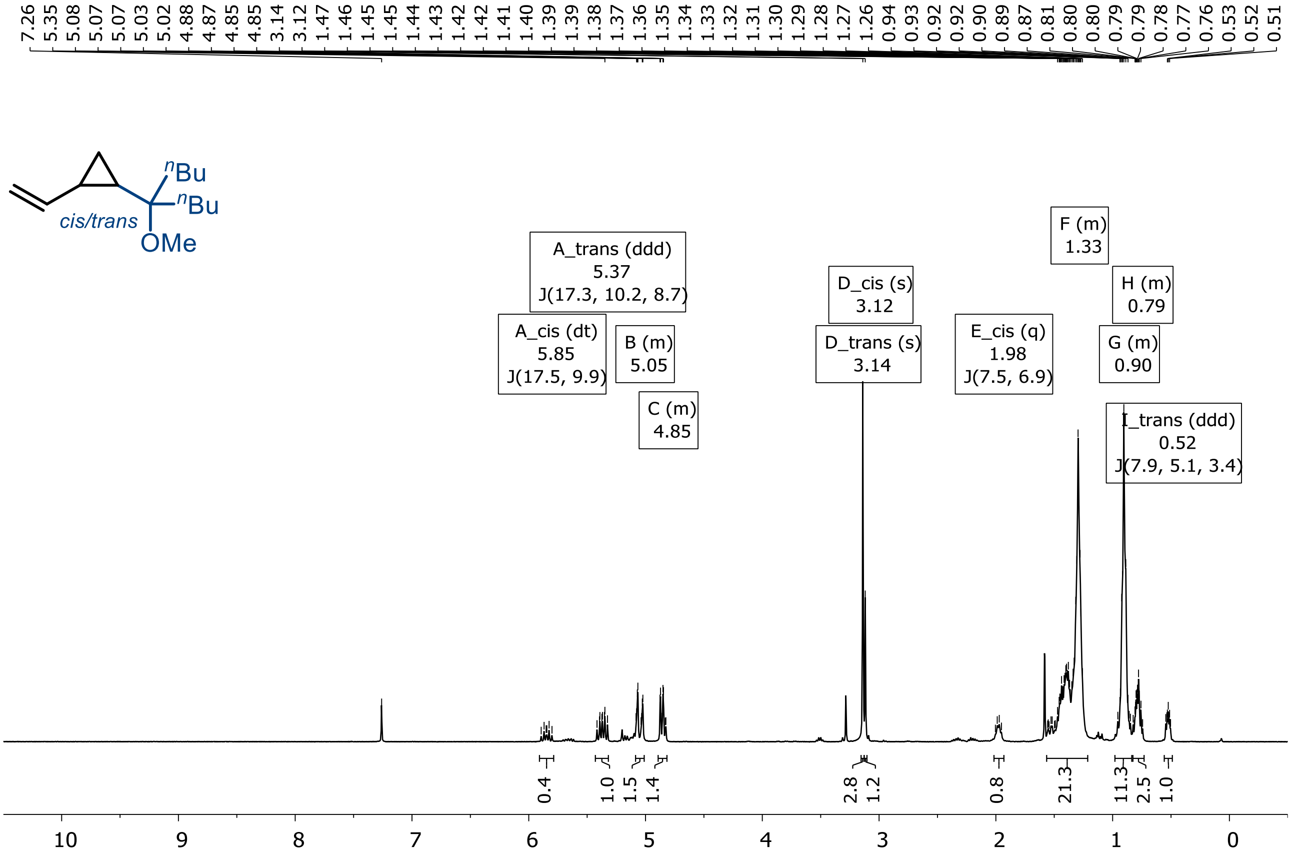

13C NMR
(100.58 MHz, CDCl3)

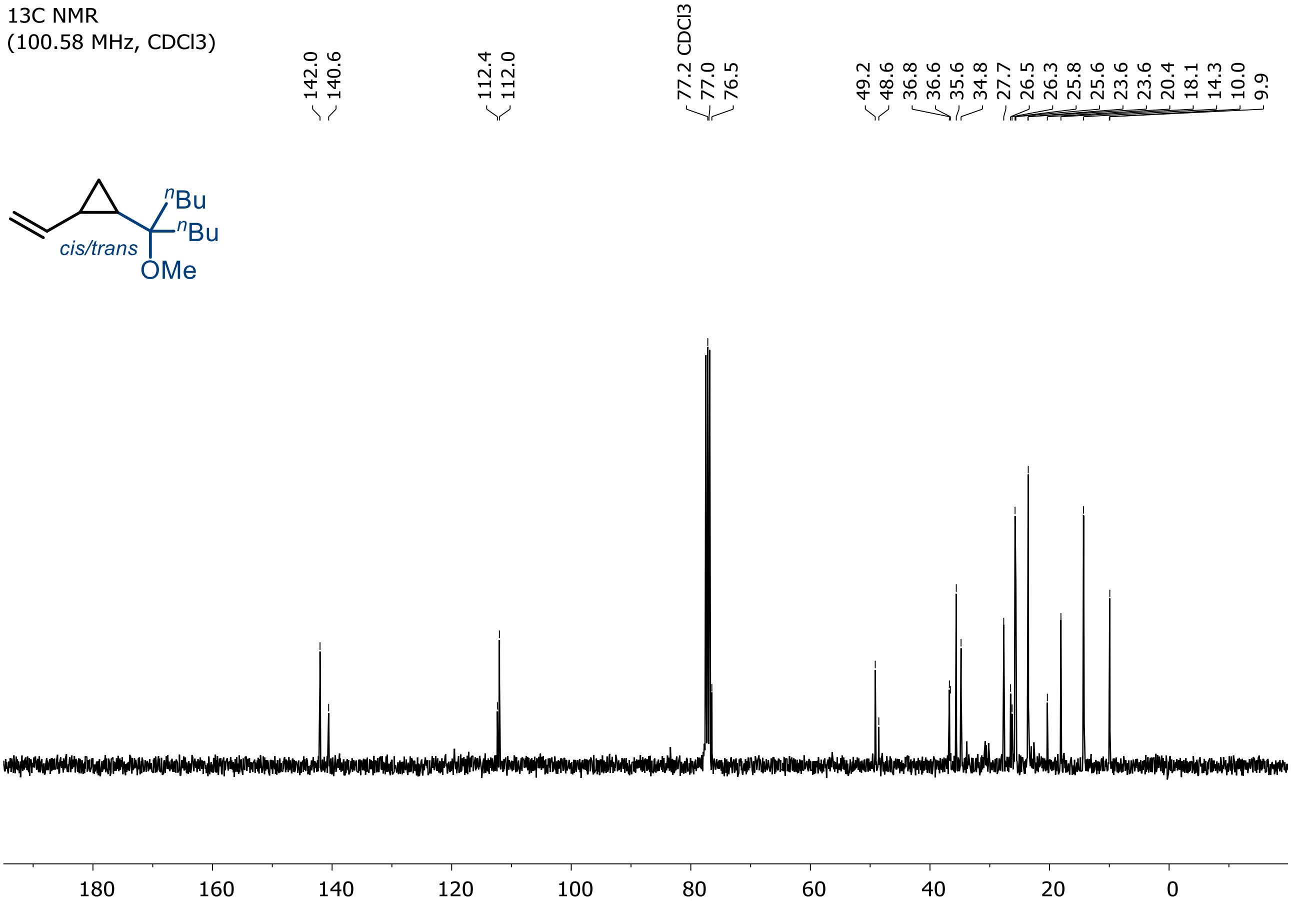

trans-1-(5-methoxynonan-5-yl)-2-vinylcyclopropane (20)

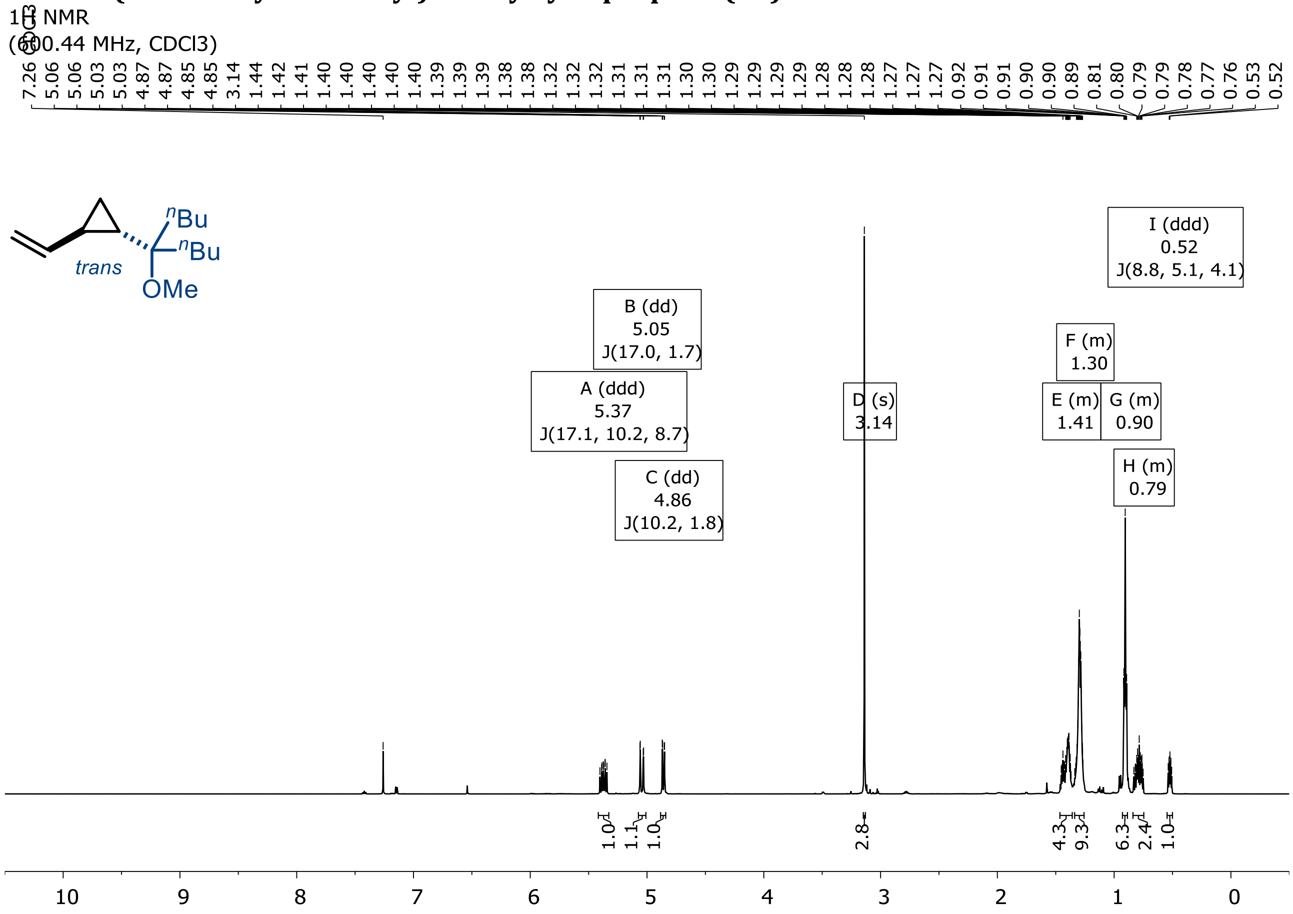

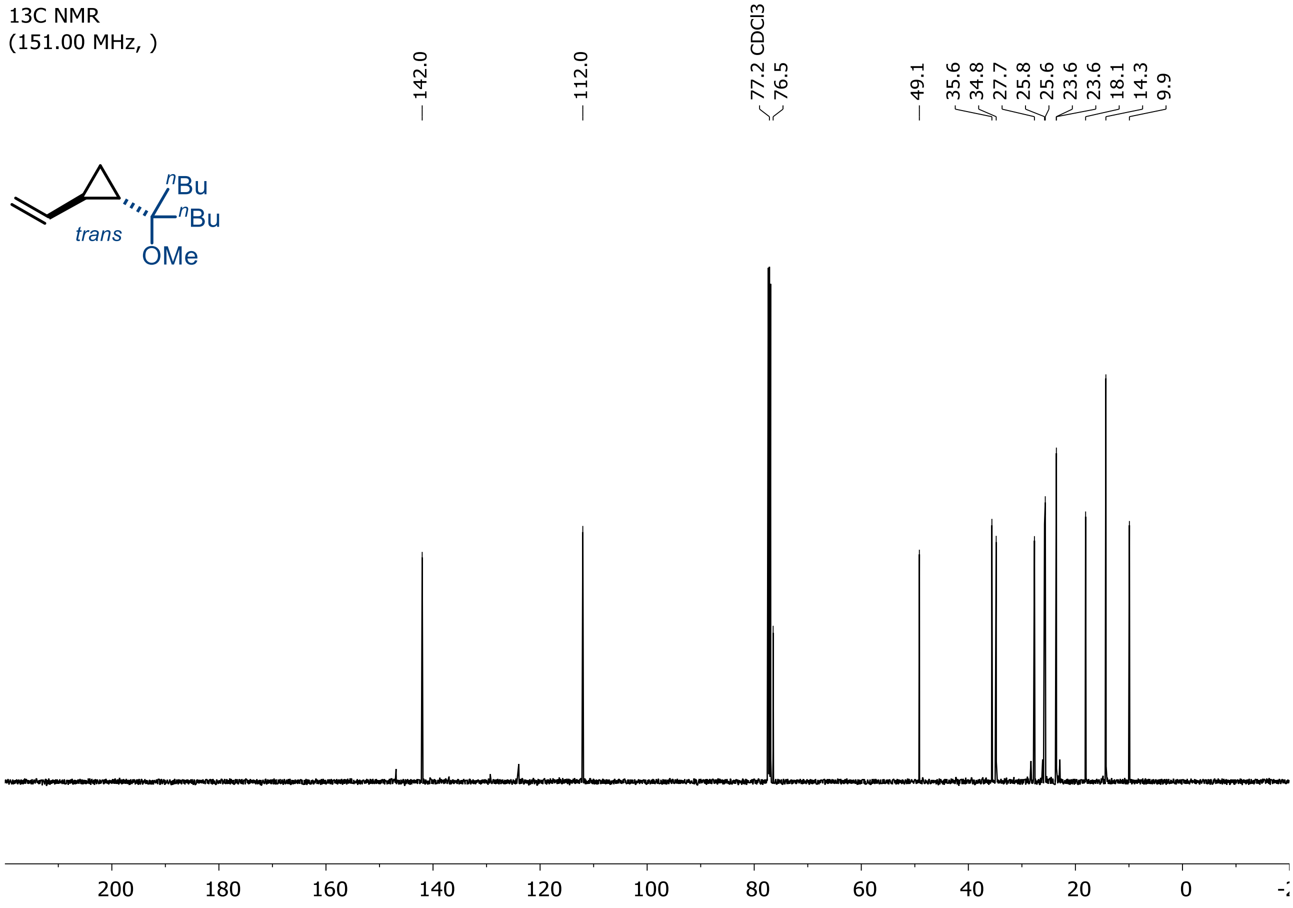

N,*N*-diethyl-2-vinylcyclopropane-1-carboxamide (*S*21)

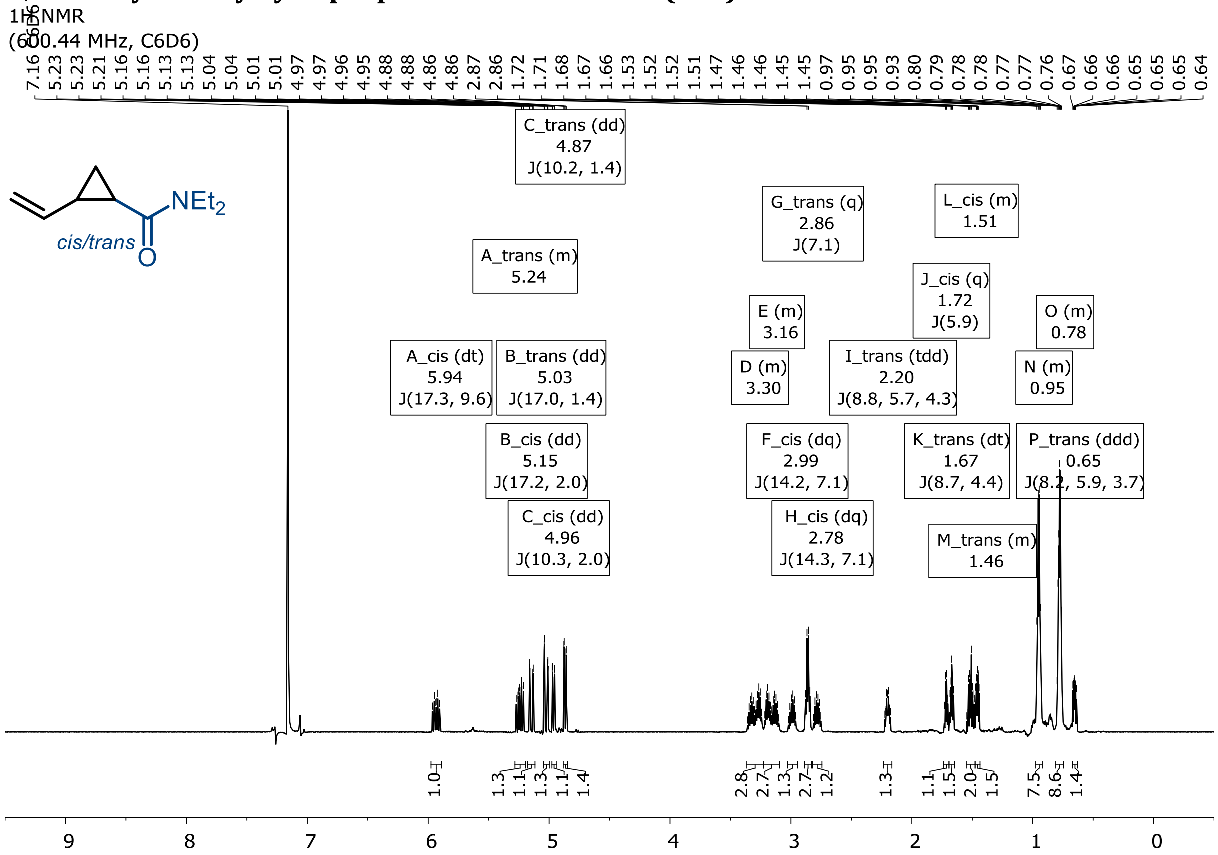

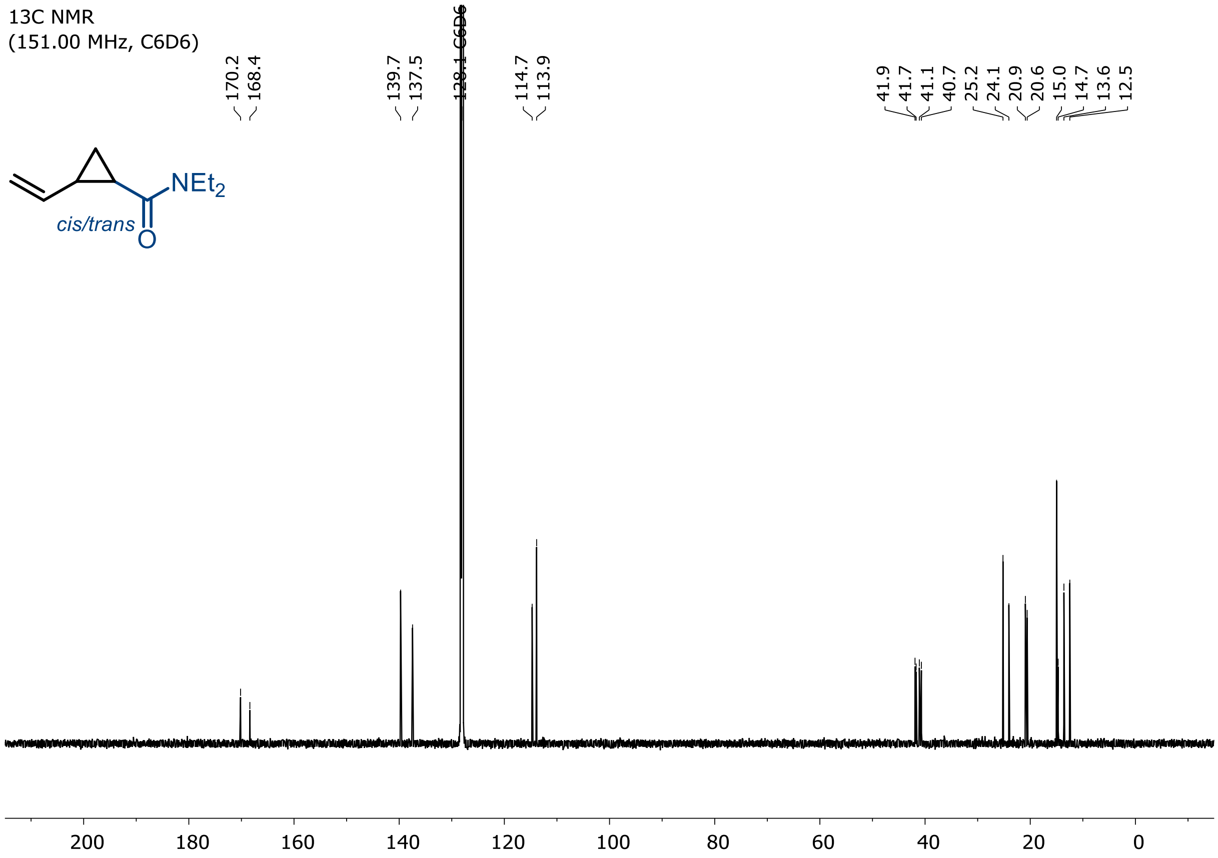

***trans-N,N*-diethyl-2-vinylcyclopropane-1-carboxamide (21)**

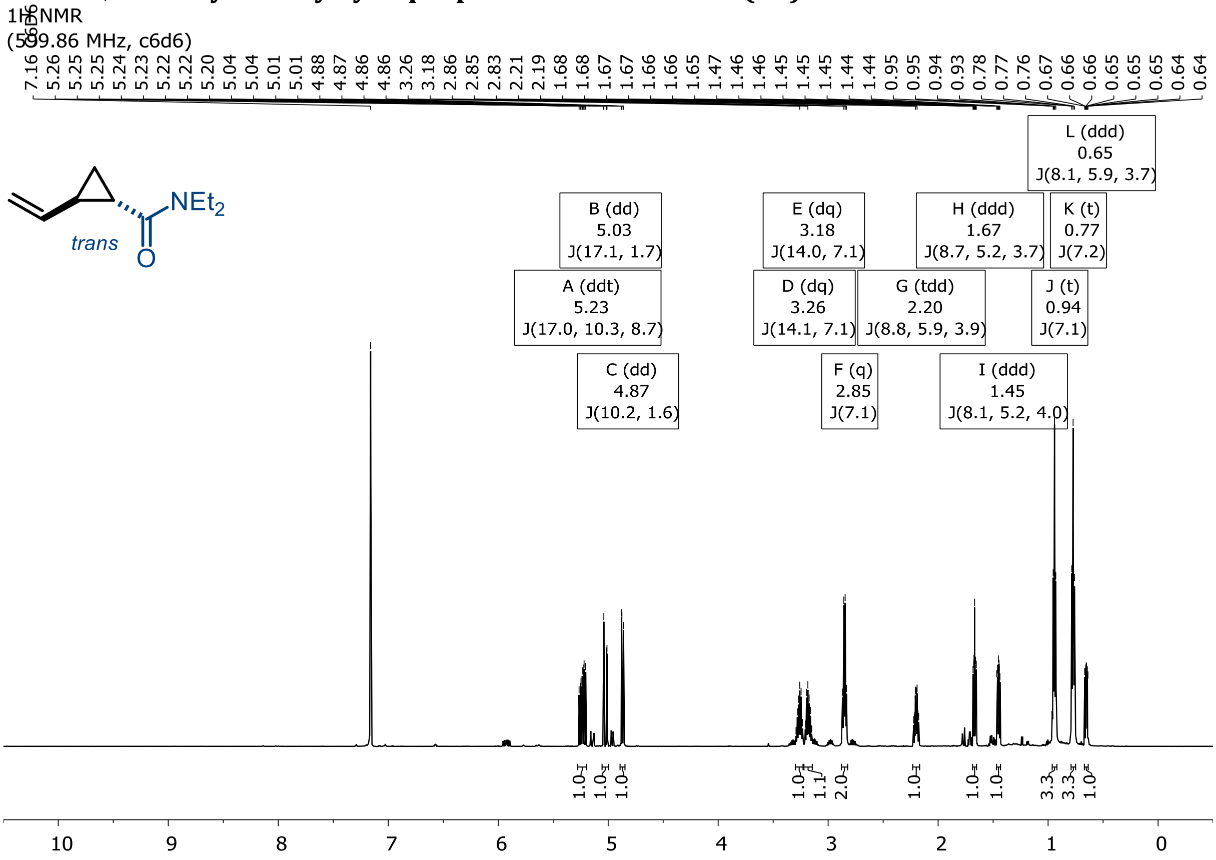

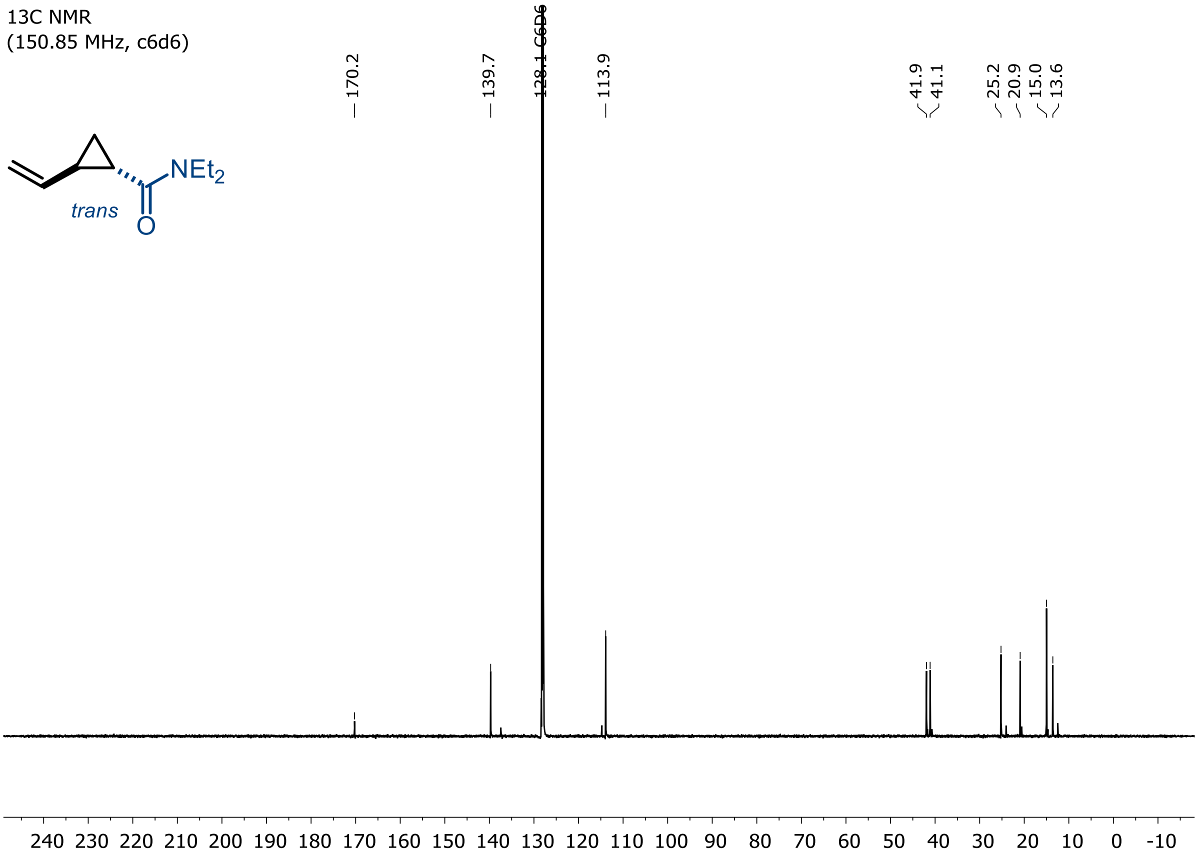

N-([1,1'-biphenyl]-4-yl)-2-vinylcyclopropane-1-carboxamide (*S*22)

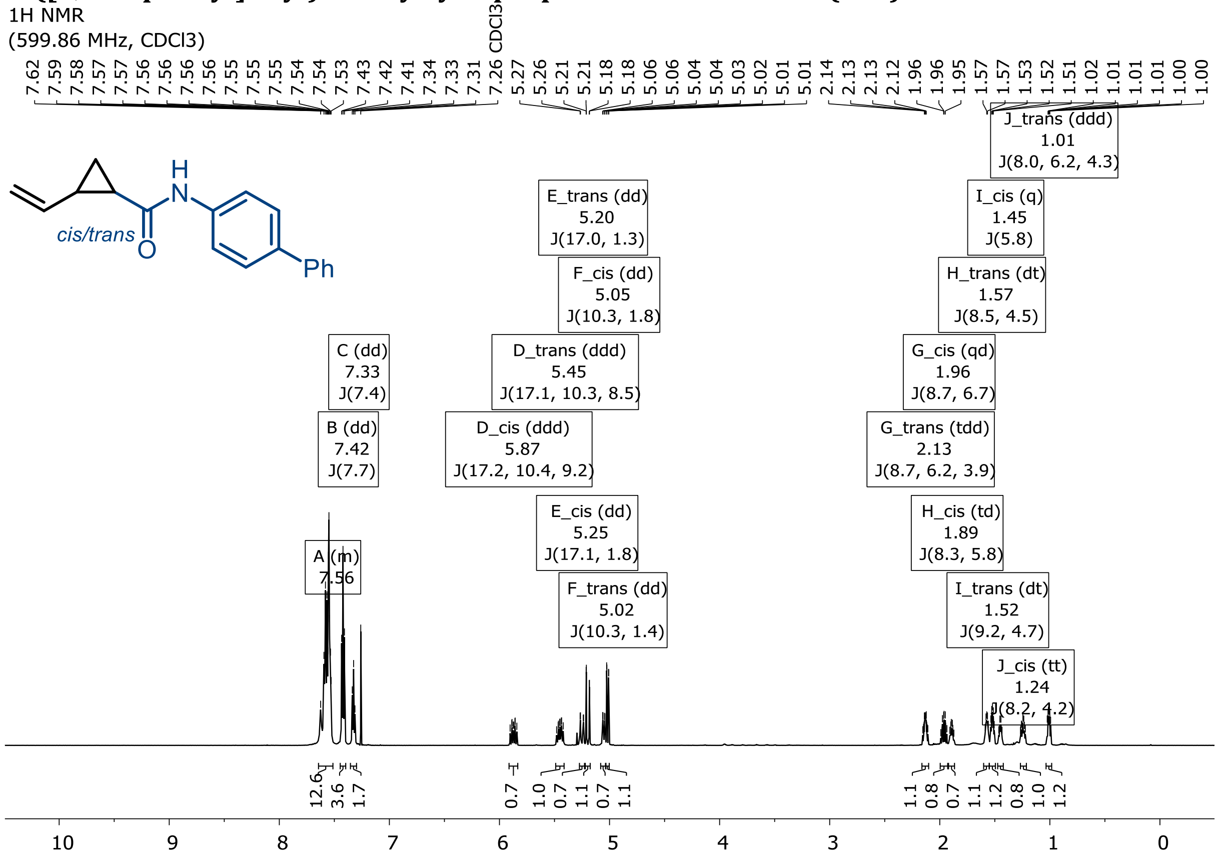

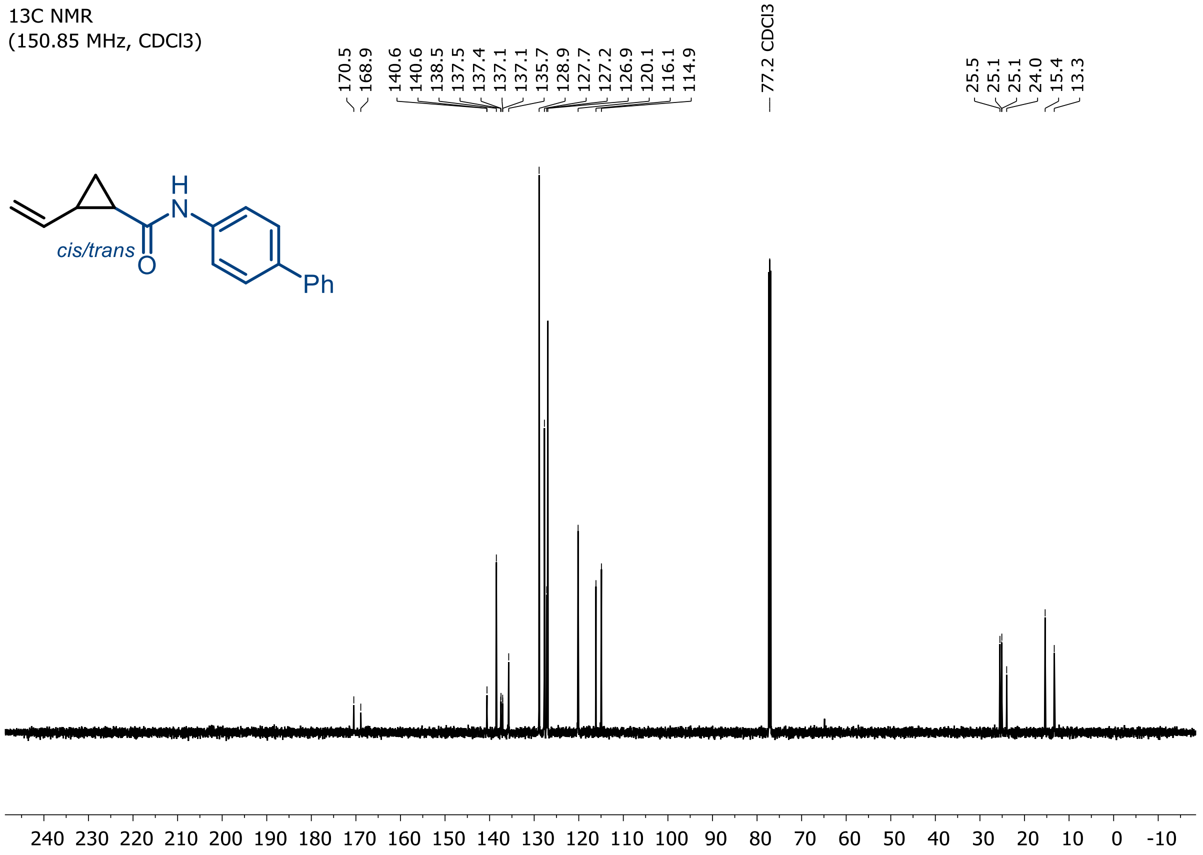

trans-N-([1,1'-biphenyl]-4-yl)-2-vinylcyclopropane-1-carboxamide (22)

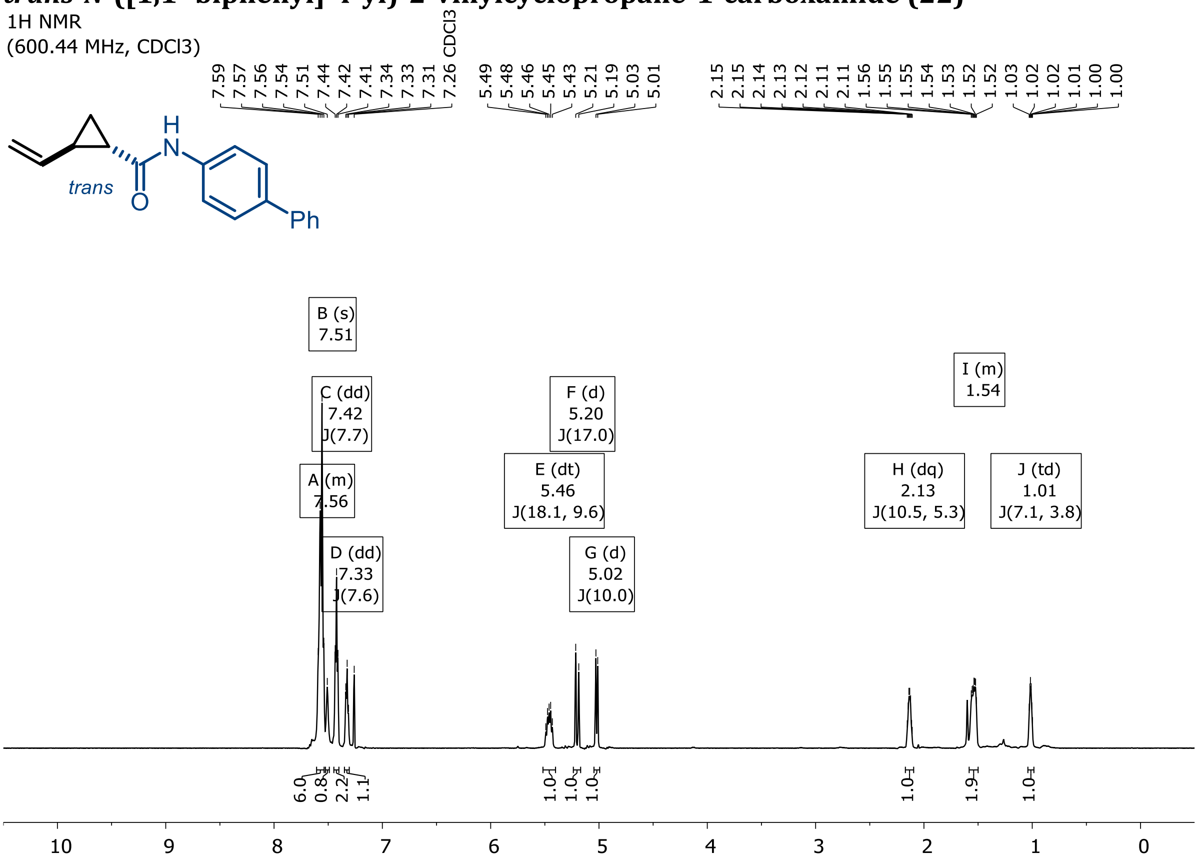

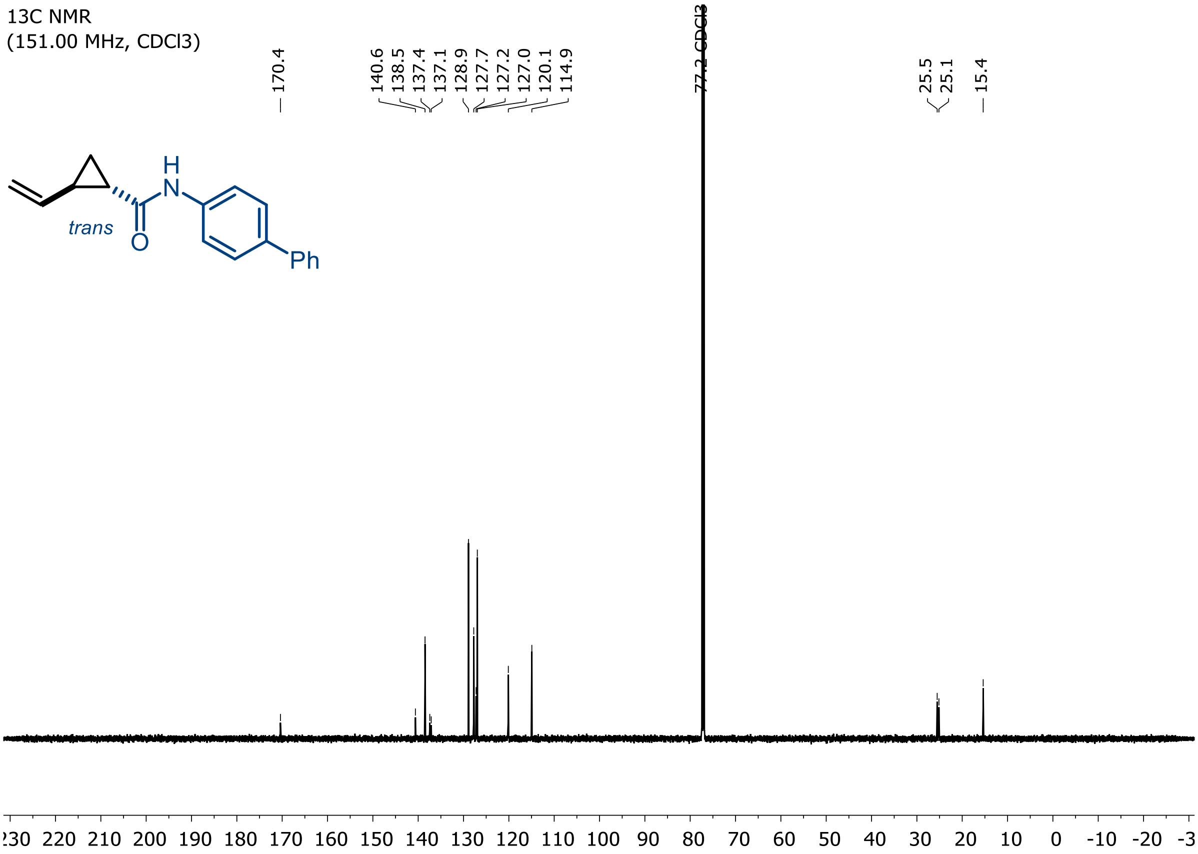

***N*-methoxy-*N*-methyl-2-vinylcyclopropane-1-carboxamide (*S23*)**

1H NMR
(399.97 MHz, CDCl3)

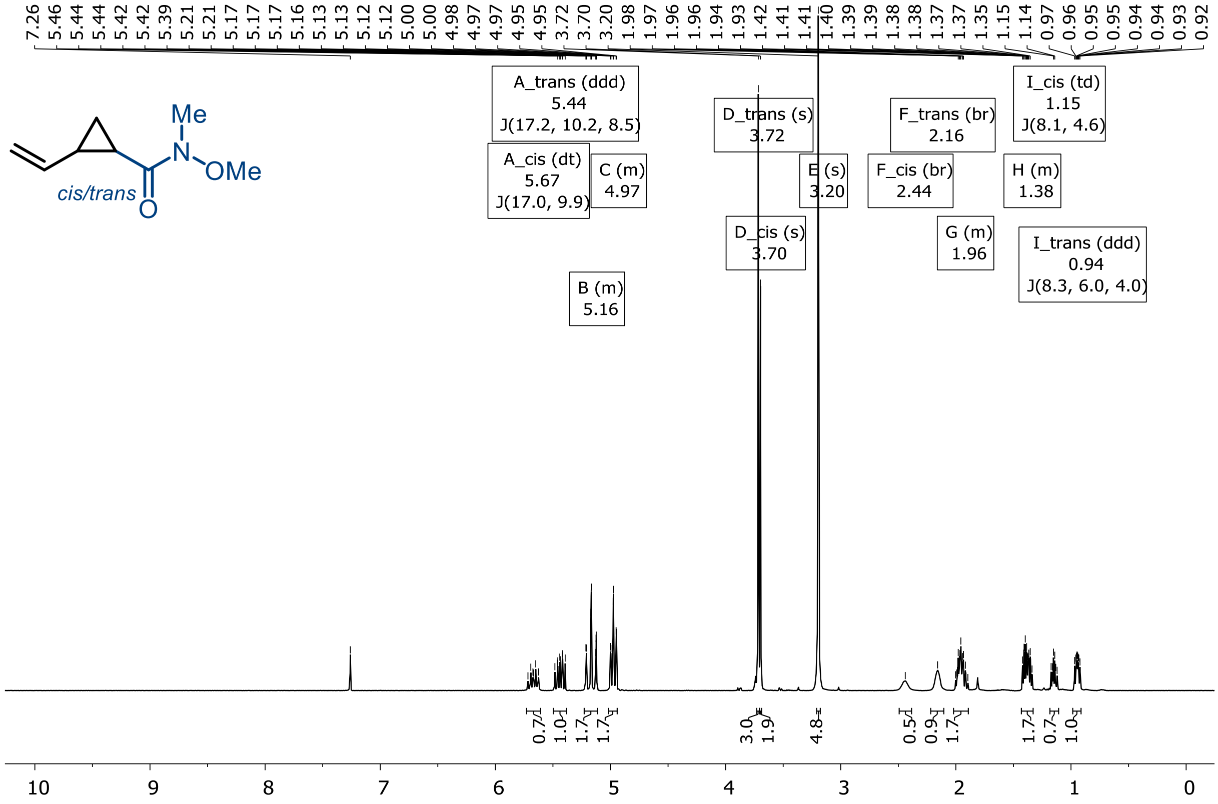

13C NMR
(100.58 MHz, CDCl3)

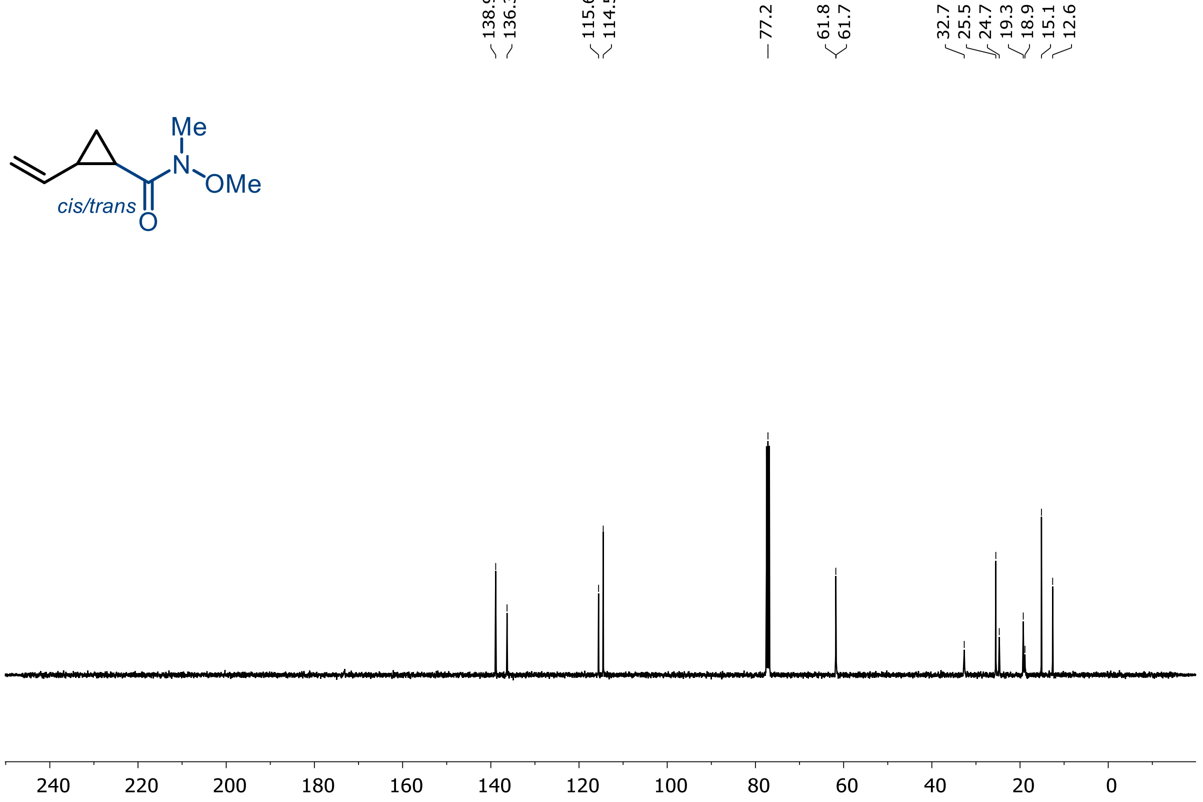

N-methoxy-*N*-methyl-*trans*-2-vinylcyclopropane-1-carboxamide (23)

(399.97 MHz, CDCl3)

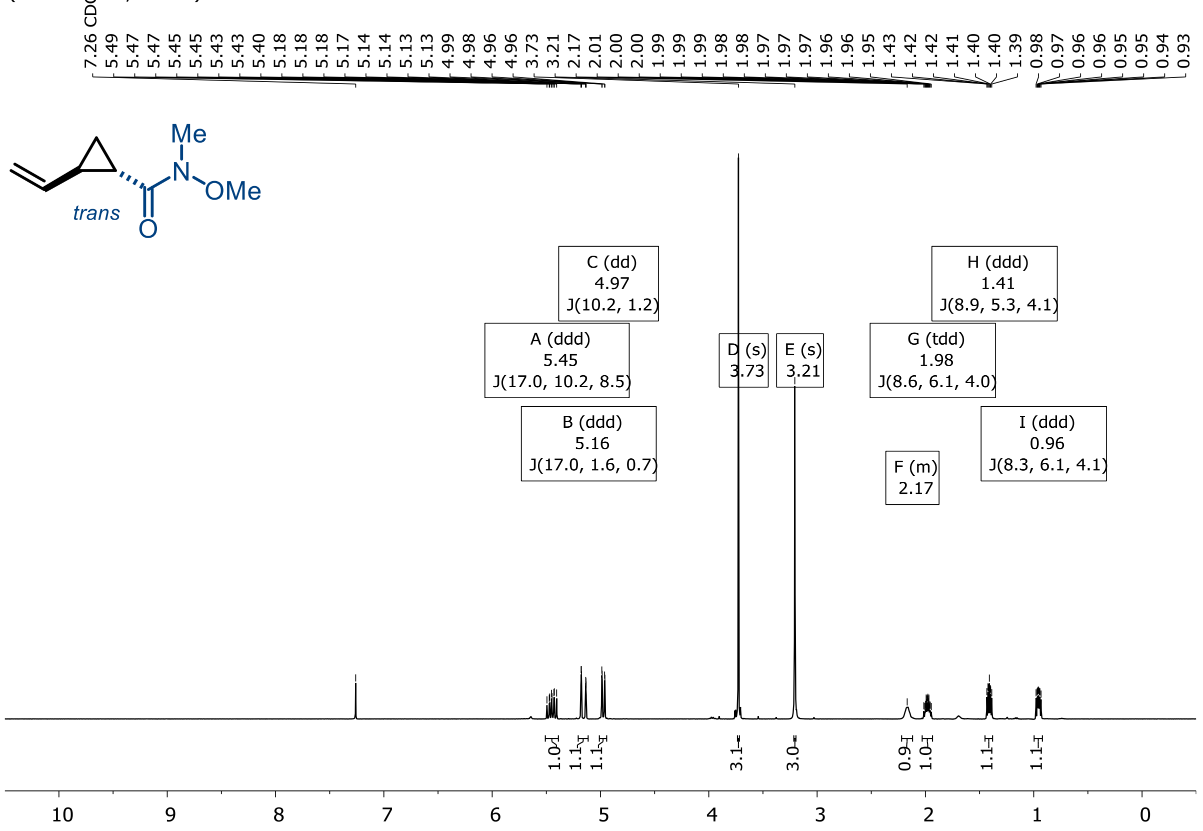

13C NMR
(100.58 MHz, CDCl3)

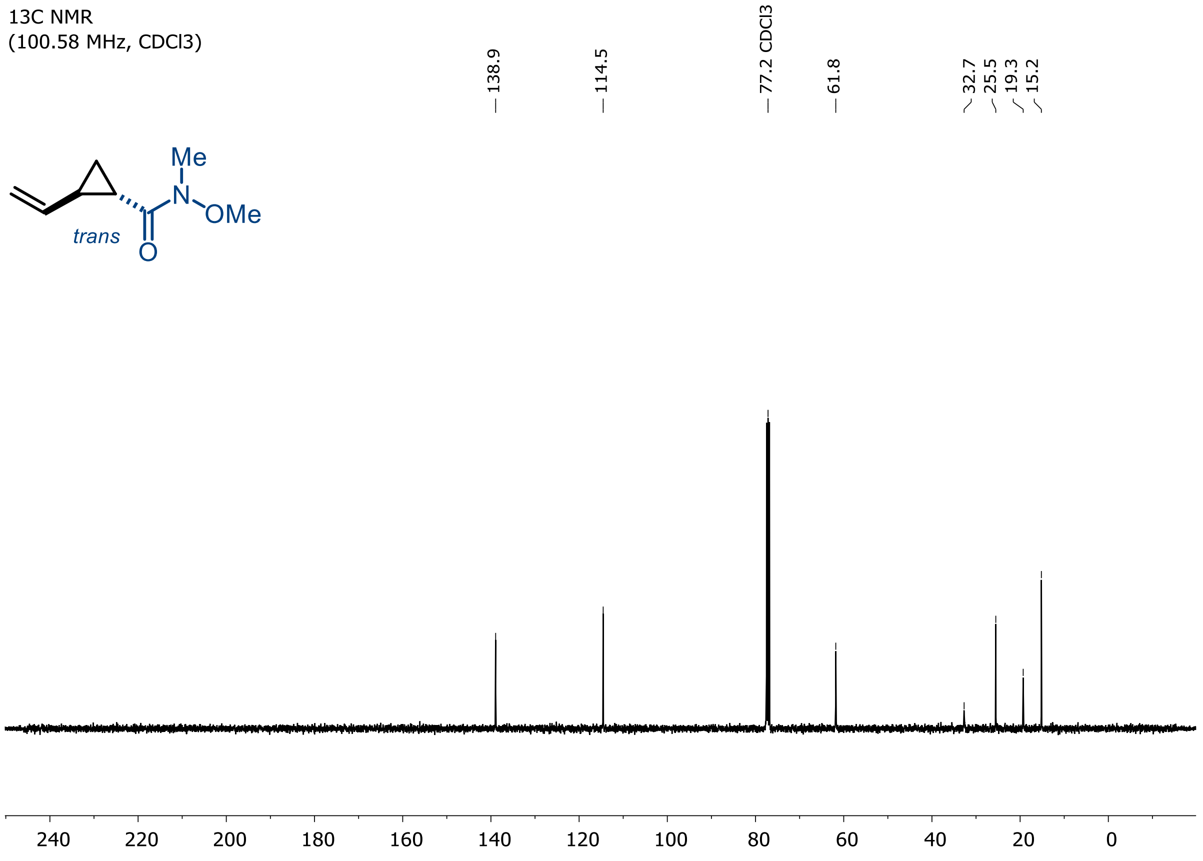

(*cis*-2-vinylcyclopropyl)triethylgermane (S24)

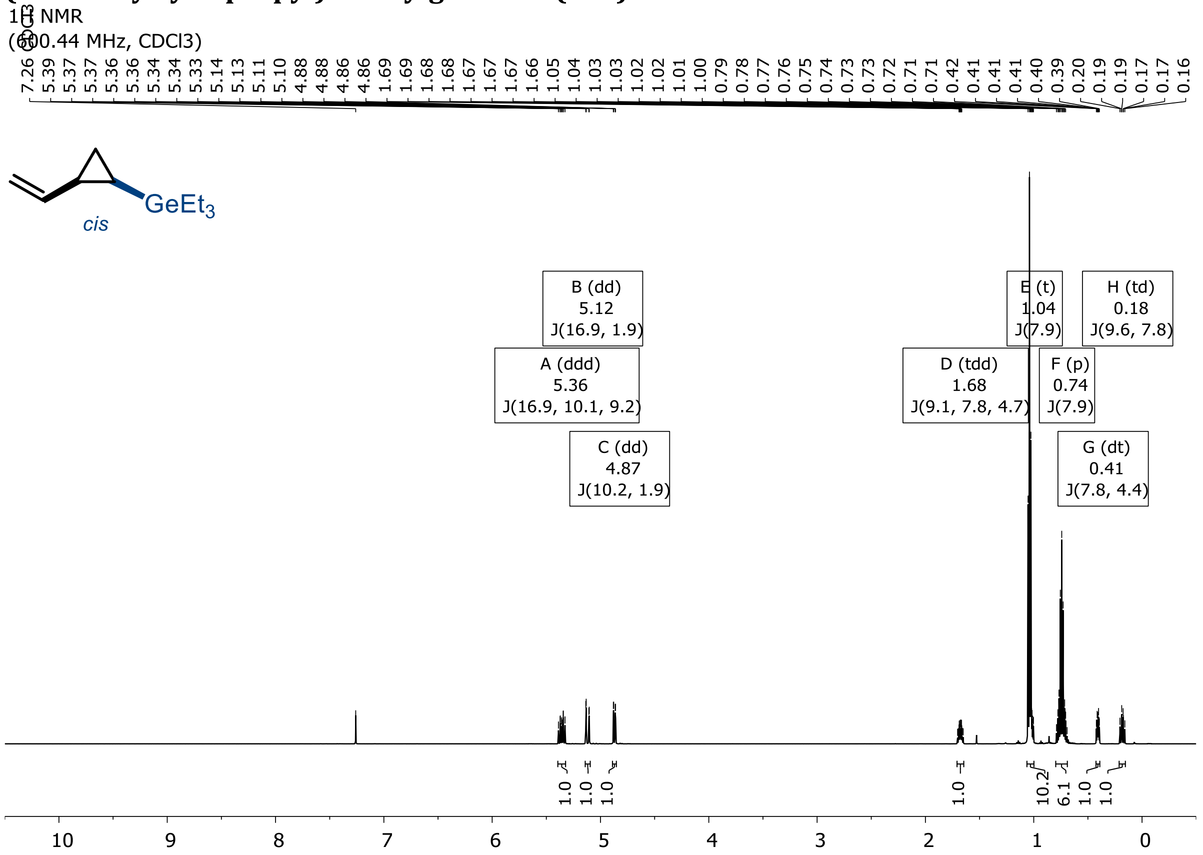

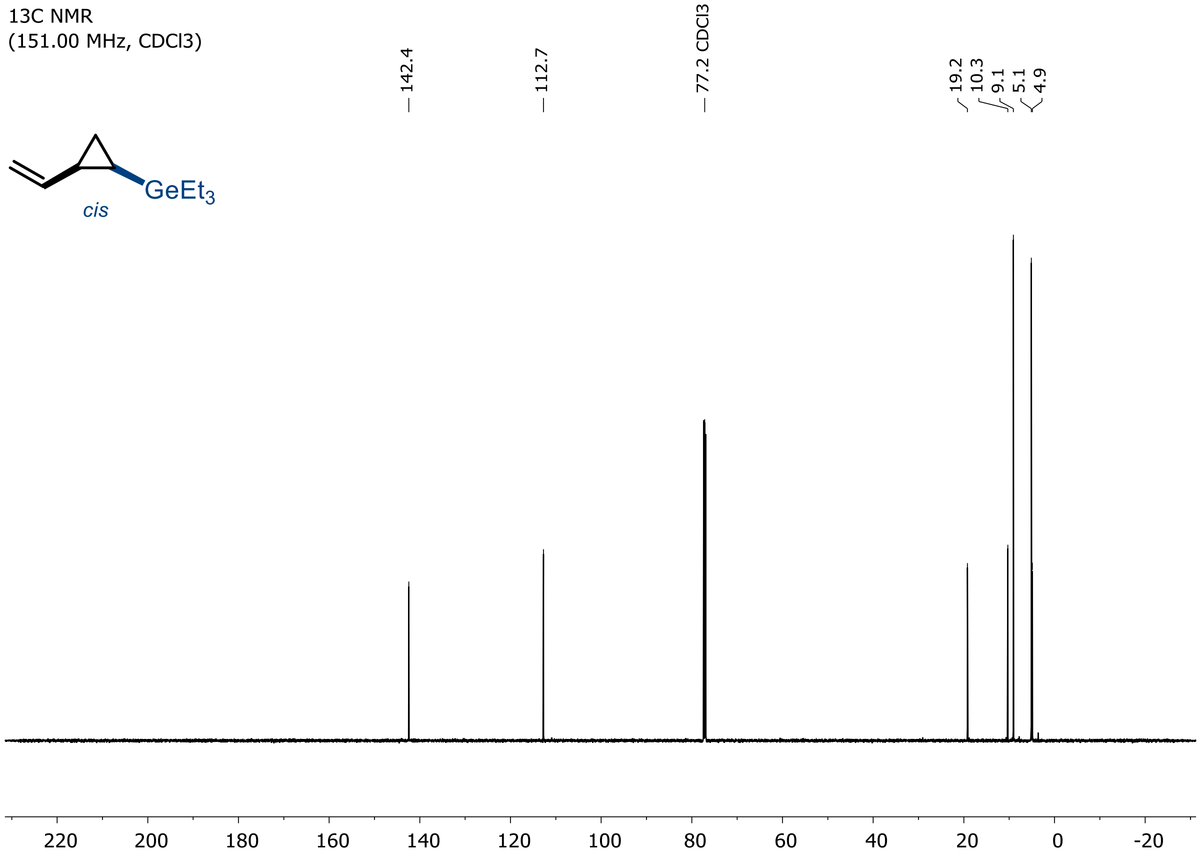

(*trans*-2-vinylcyclopropyl)triethylgermane (24)

1H NMR
(600.44 MHz, $CDCl_3$)

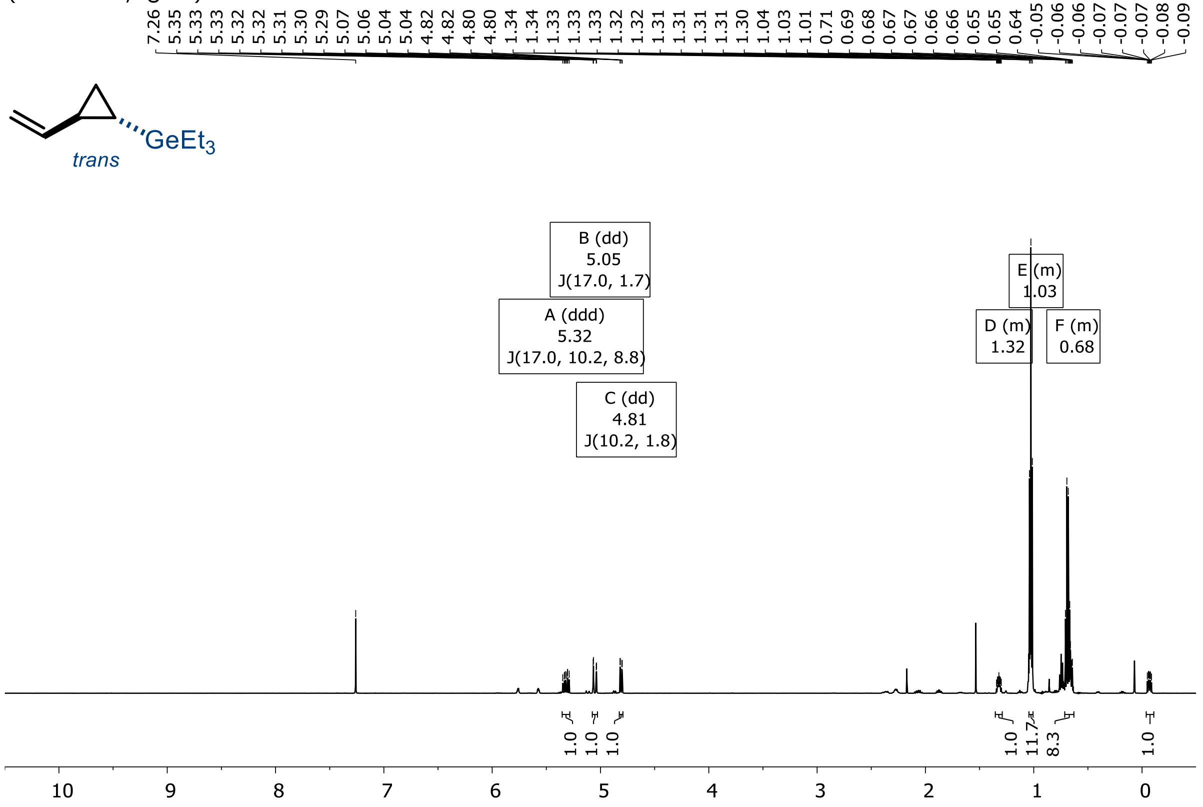

13C NMR
(151.00 MHz, $CDCl_3$)

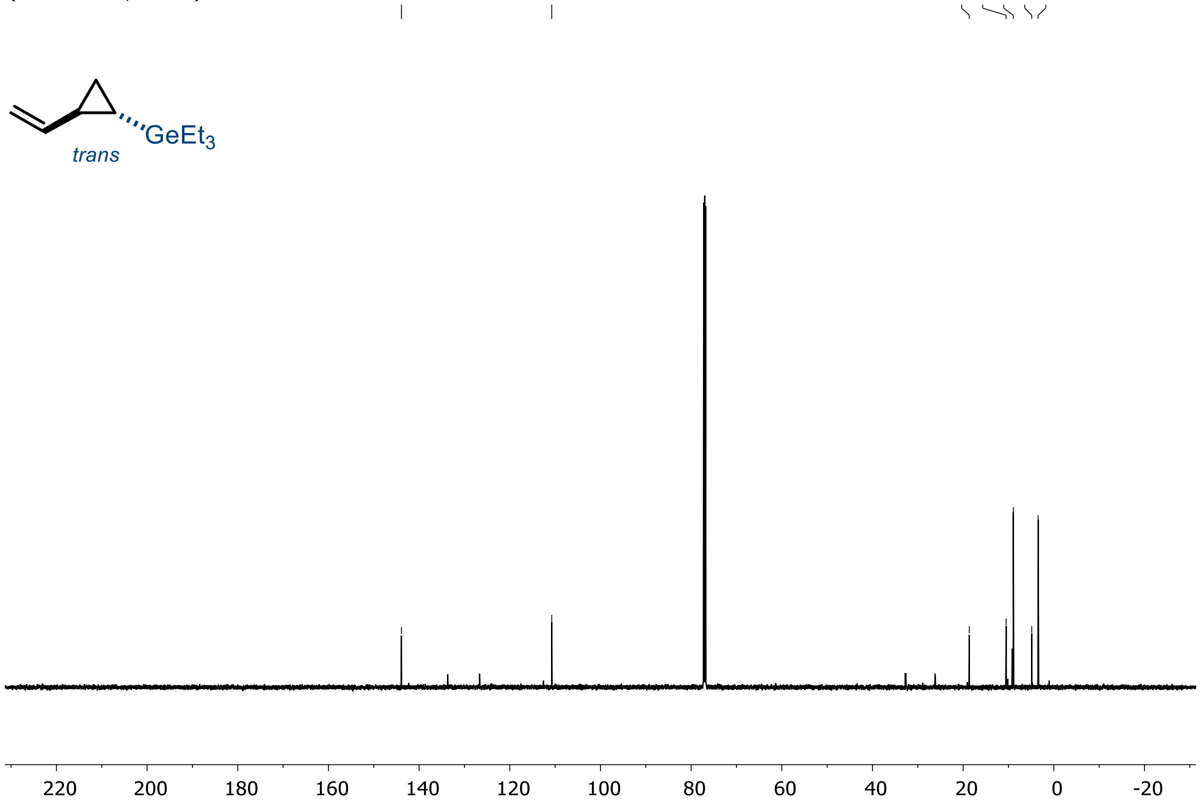

6-methyl-2-(2-vinylcyclopropyl)-1,3,6,2-dioxazaborocane-4,8-dione (S25)

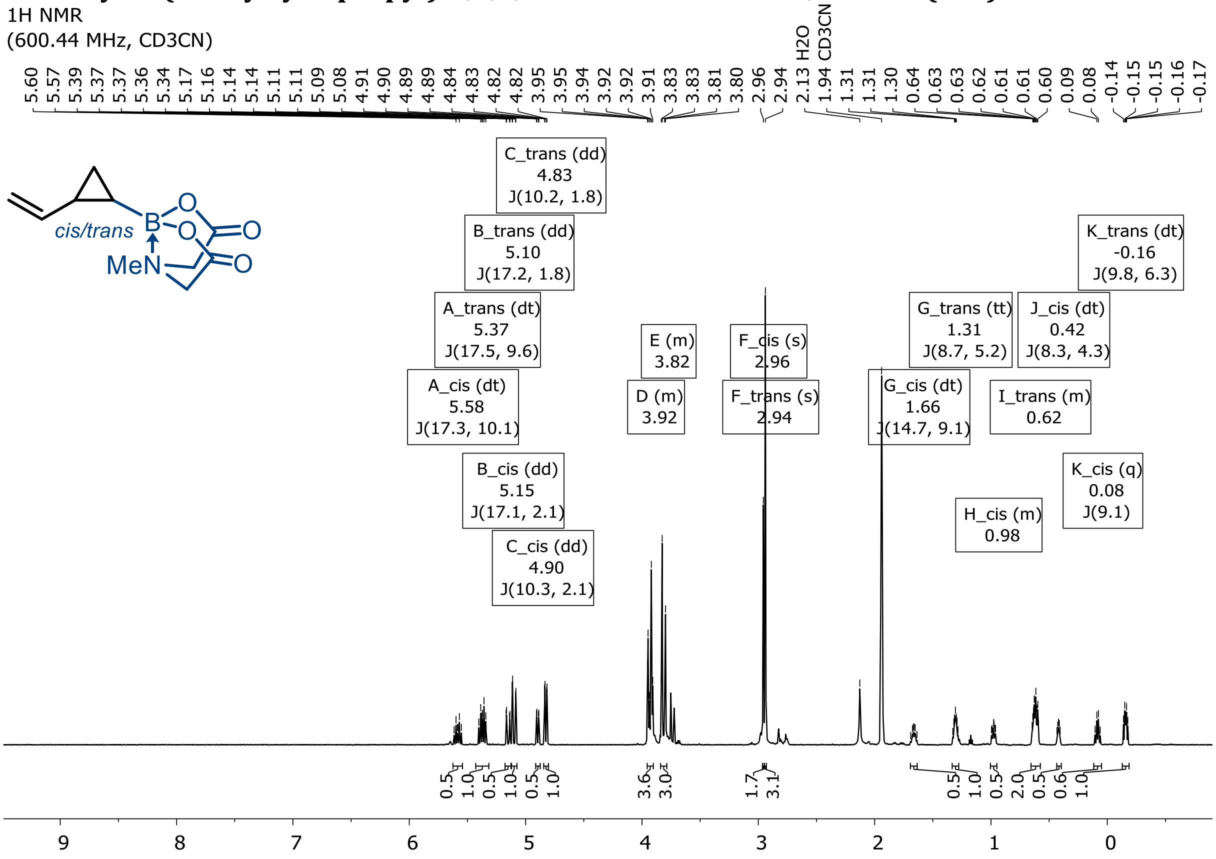

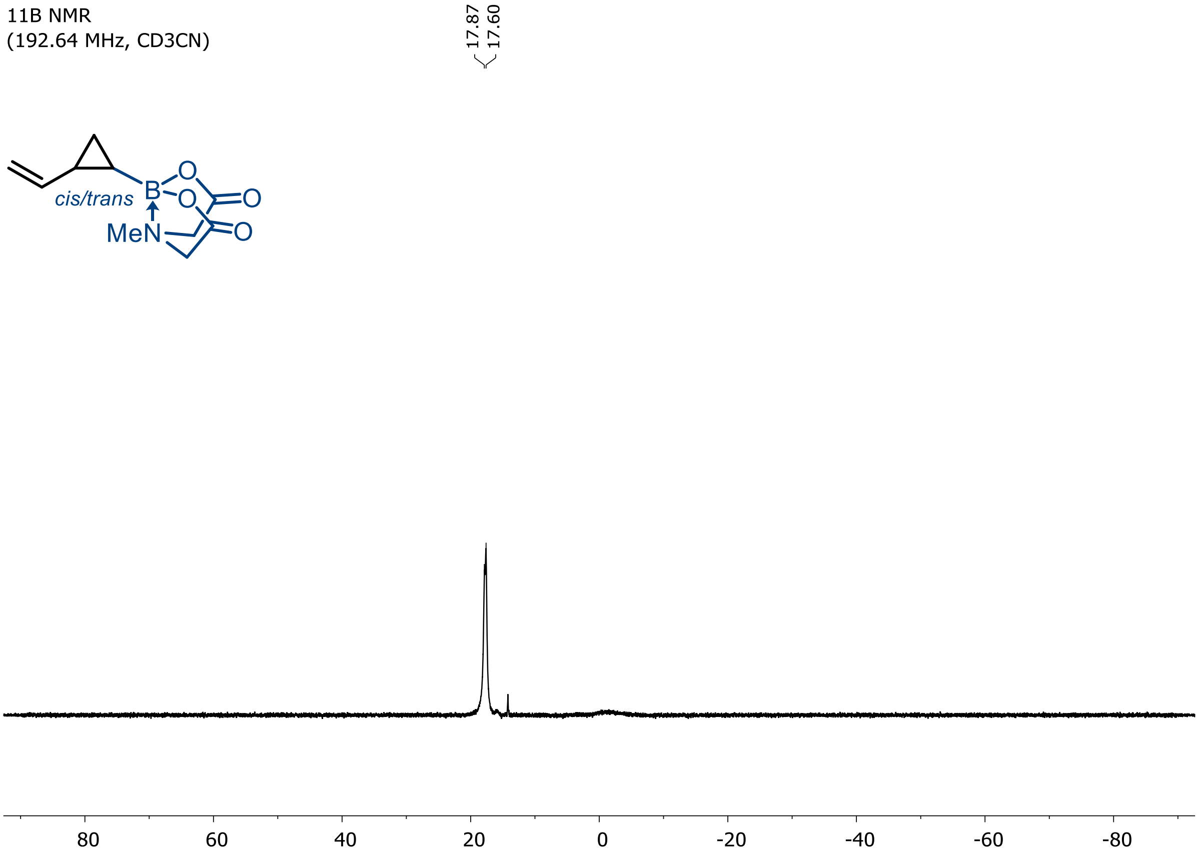

13C NMR
(151.00 MHz, CD3CN)

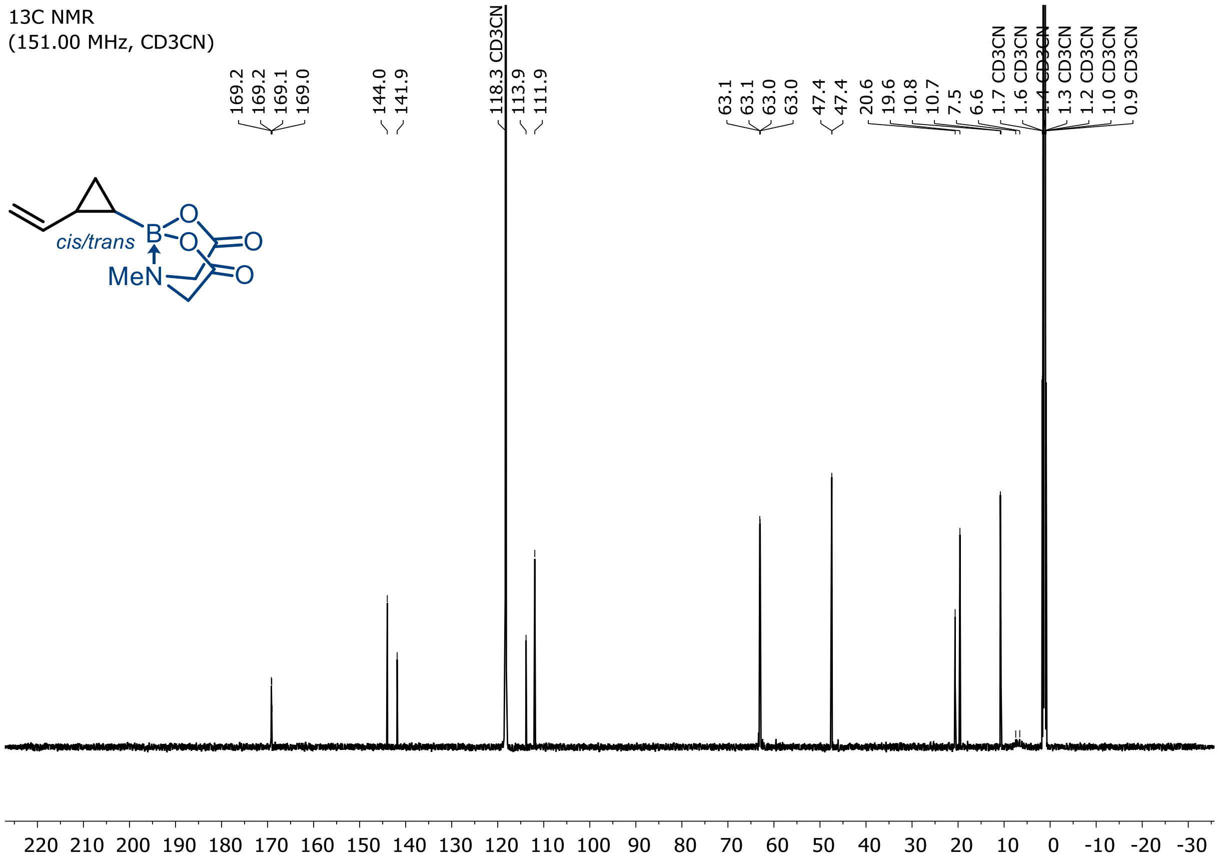

6-methyl-2-(*trans*-2-vinylcyclopropyl)-1,3,6,2-dioxazaborocane-4,8-dione (25)

1H NMR
(600.44 MHz, CD3CN)

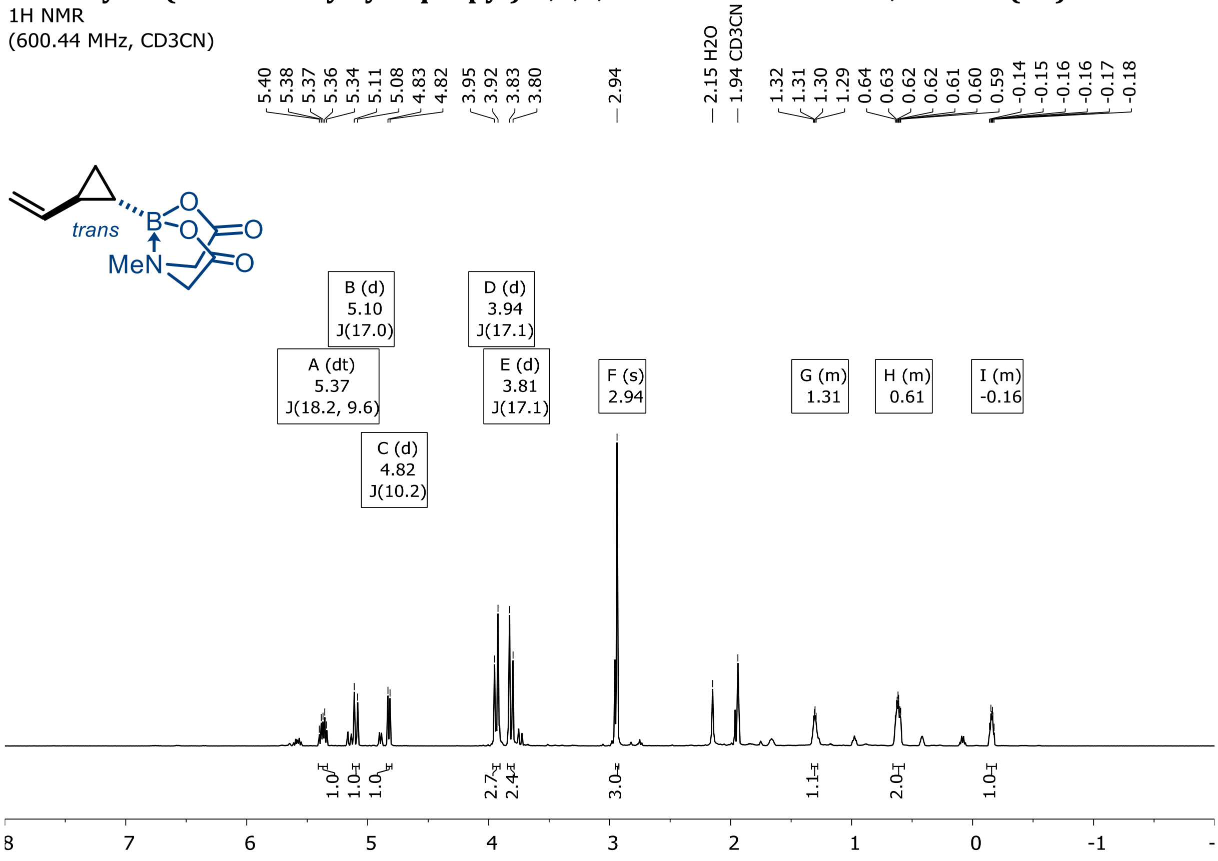

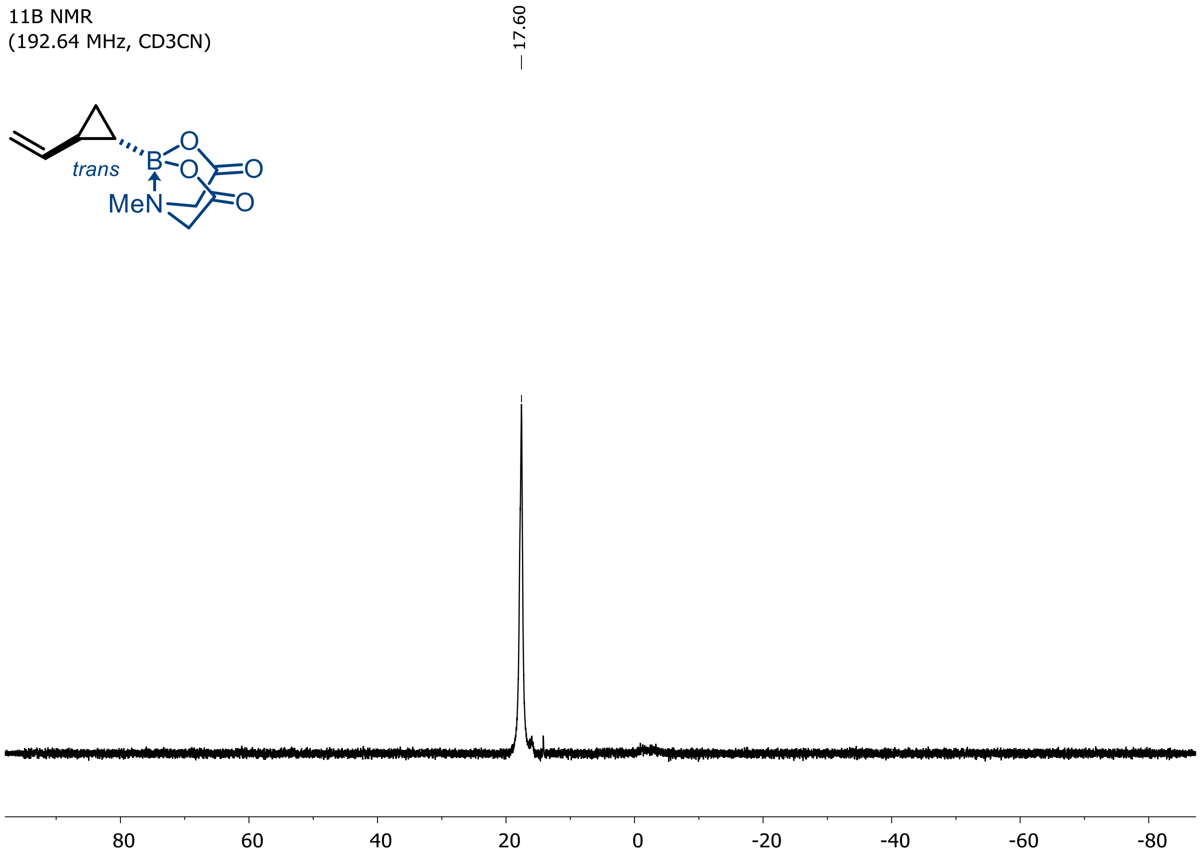
11B NMR
(192.64 MHz, CD3CN)
17.60
trans
B
O
O
O
O
MeN
80
60
40
20
0
-20
-40
-60
-80

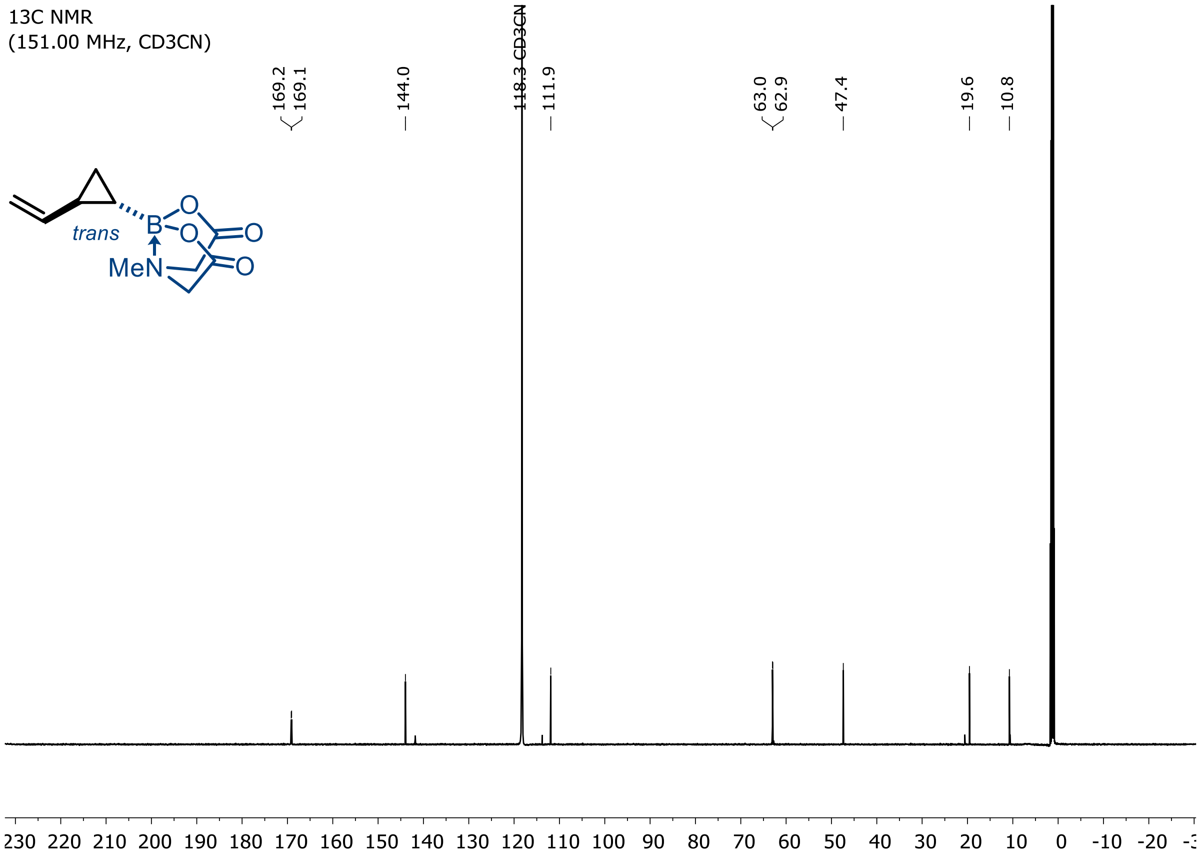
13C NMR
(151.00 MHz, CD3CN)
169.2
169.1
144.0
118.3 CD3CN
111.9
63.0
62.9
47.4
19.6
10.8
trans
B
O
O
O
O
MeN
230 220 210 200 190 180 170 160 150 140 130 120 110 100 90 80 70 60 50 40 30 20 10 0 -10 -20

4,4,5,5-tetramethyl-2-(2-vinylcyclopropyl)-1,3,2-dioxaborolane (S26)

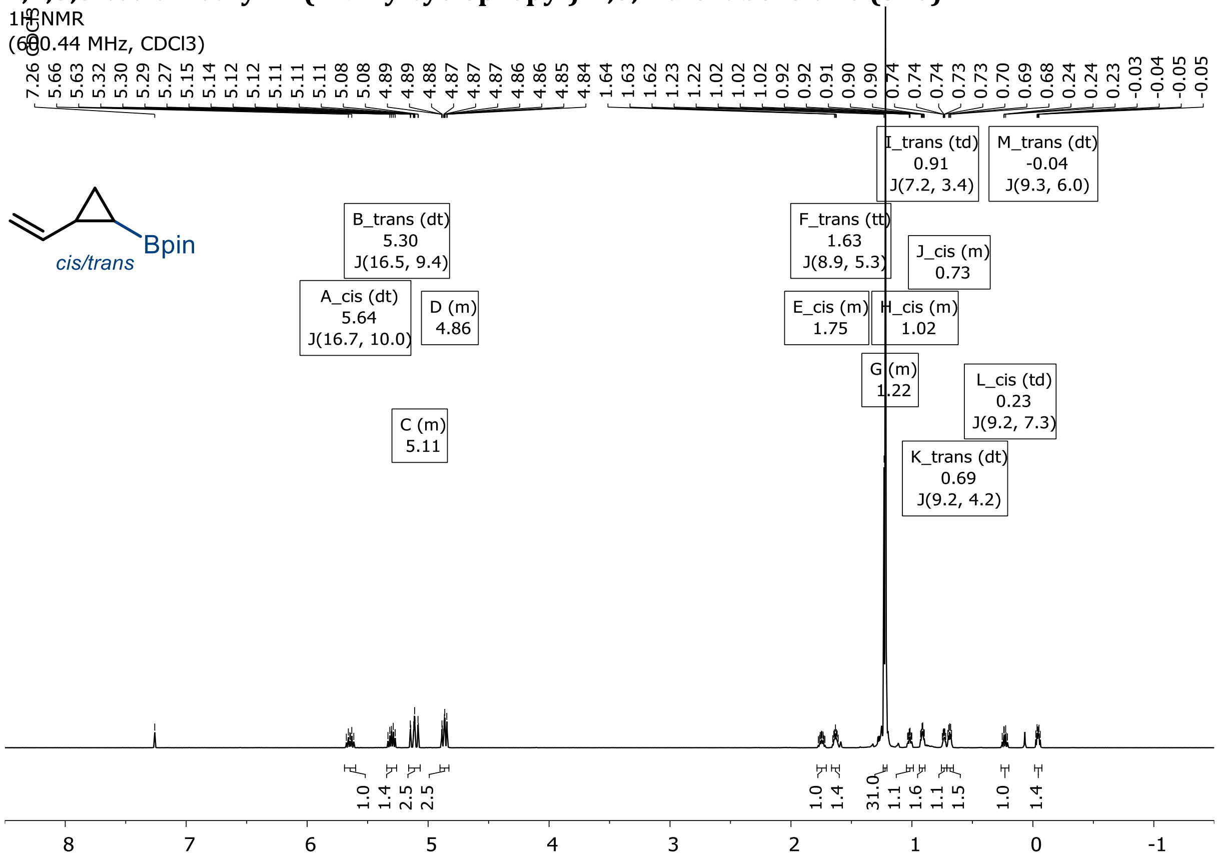

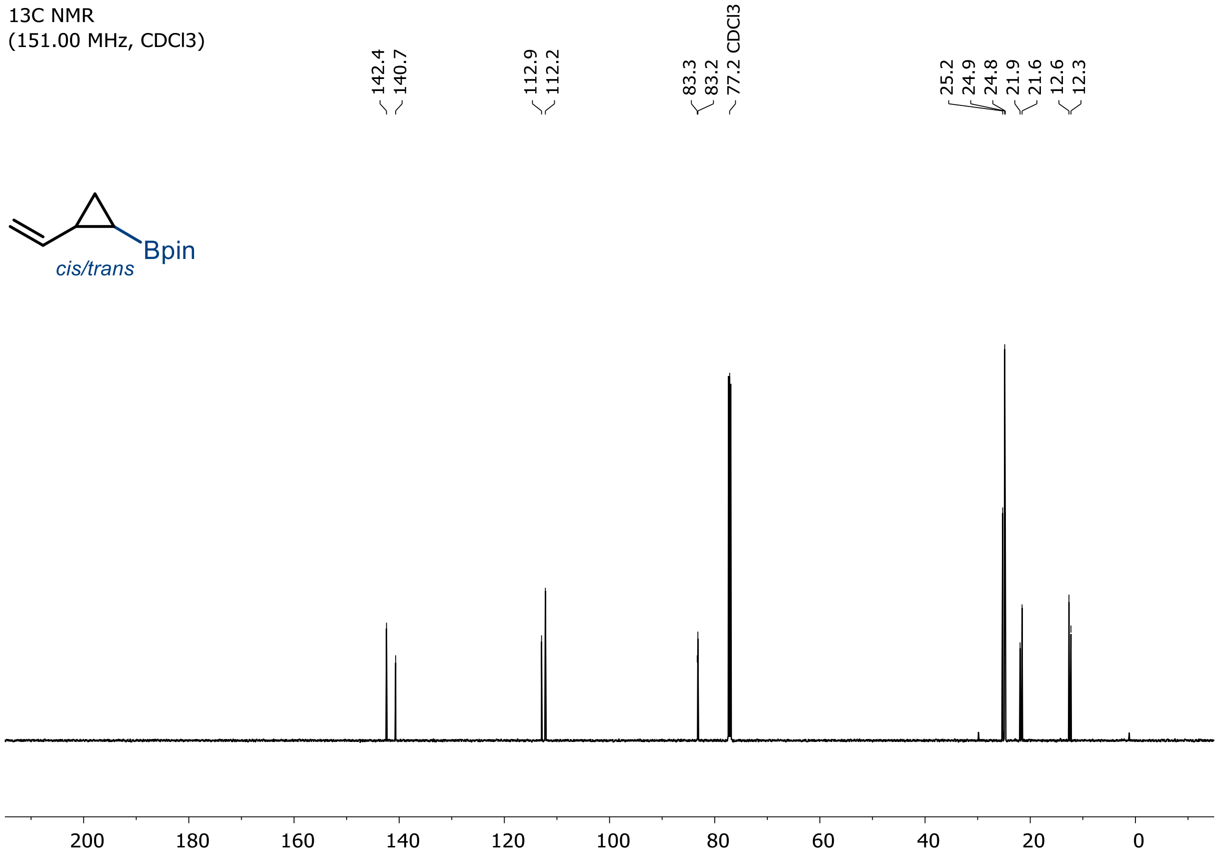

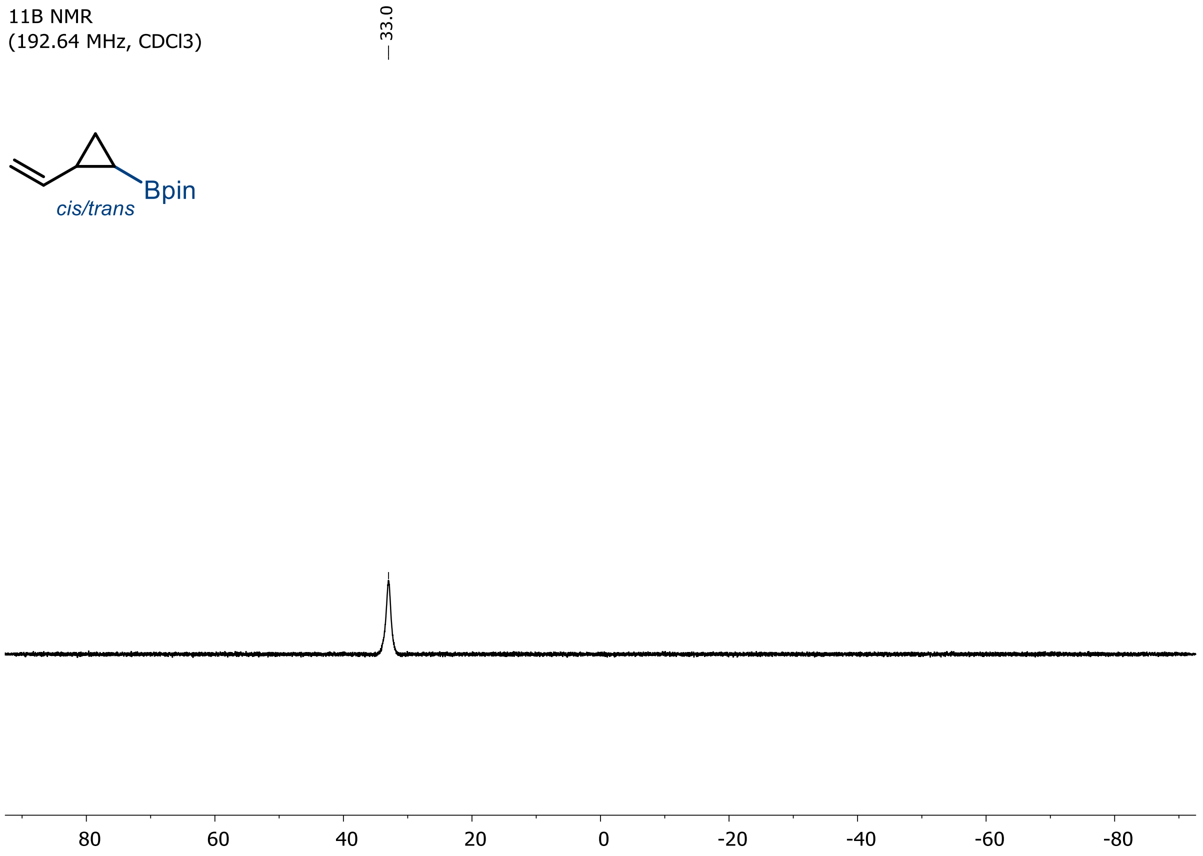

4,4,5,5-tetramethyl-2-(*trans*-2-vinylcyclopropyl)-1,3,2-dioxaborolane (26)

(600.44 MHz, CDCl3)

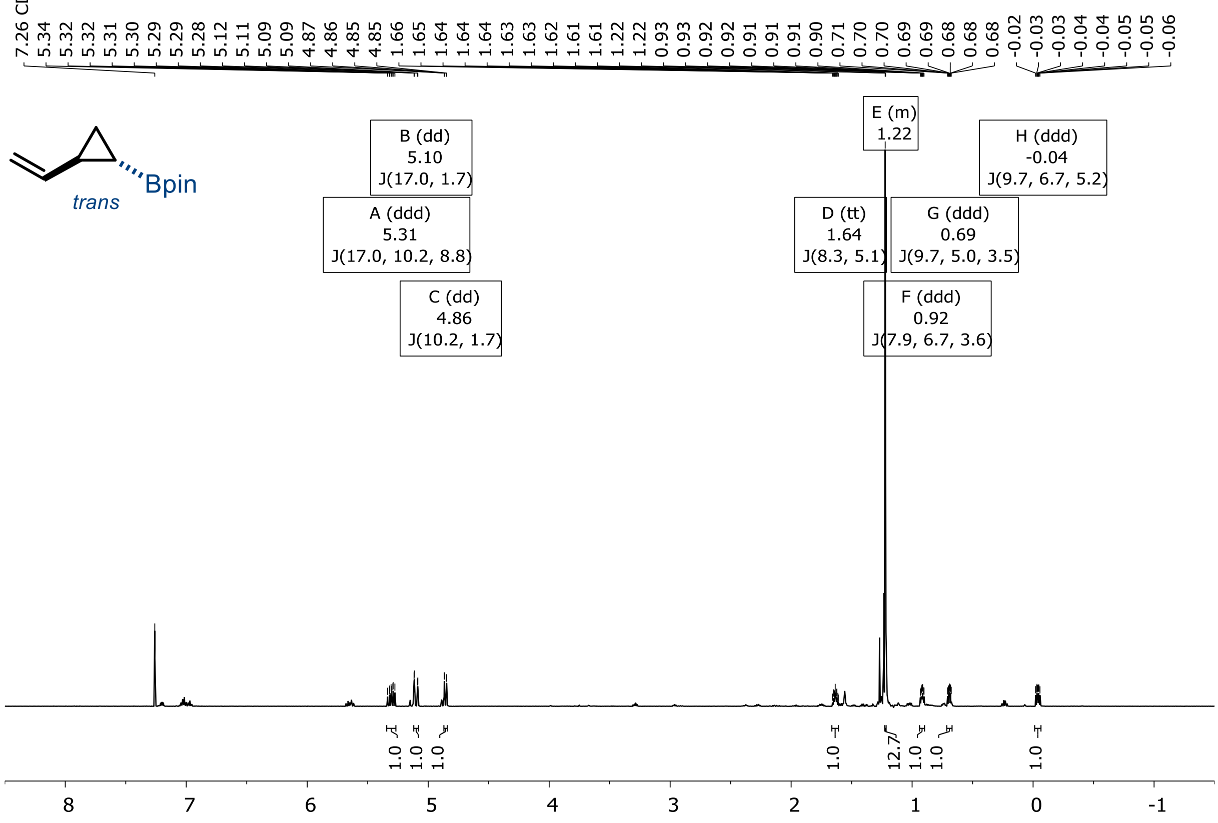

13C NMR
(151.00 MHz, CDCl3)

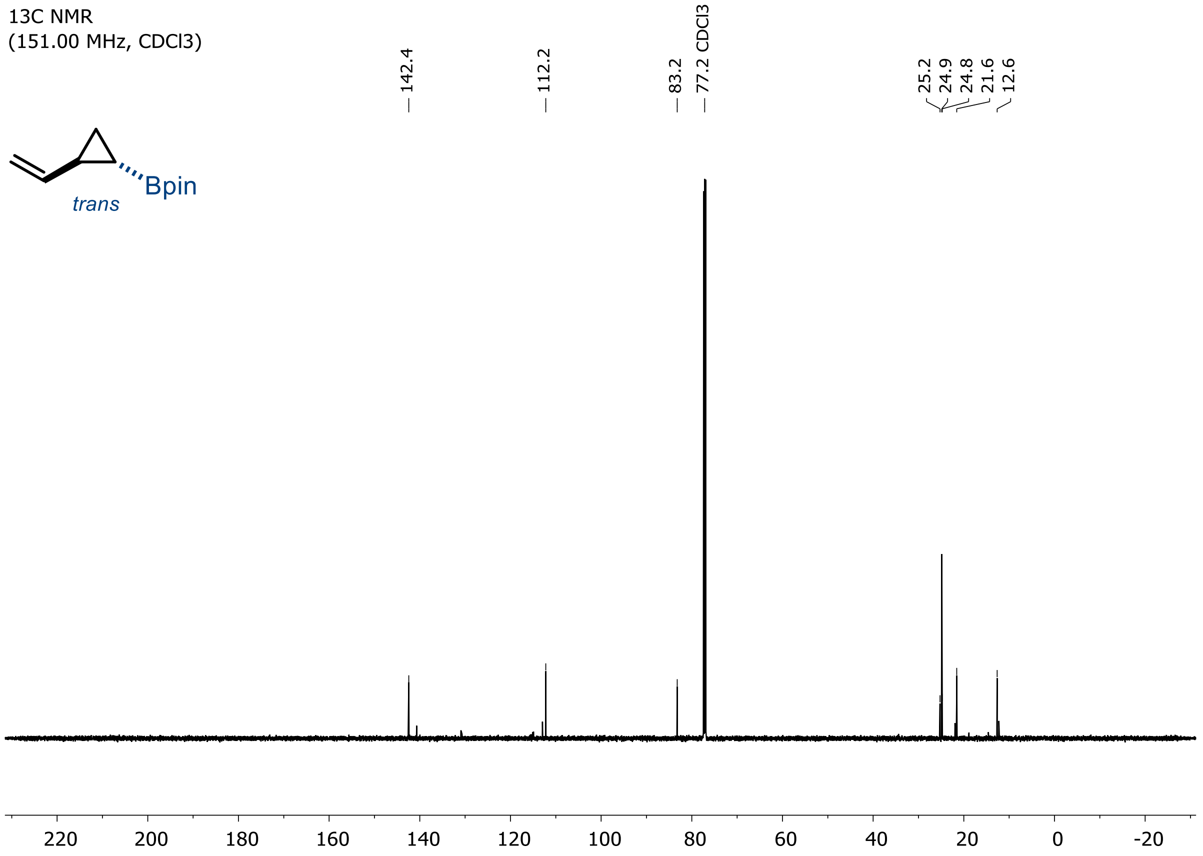

11B NMR
(192.64 MHz, CDCl3)

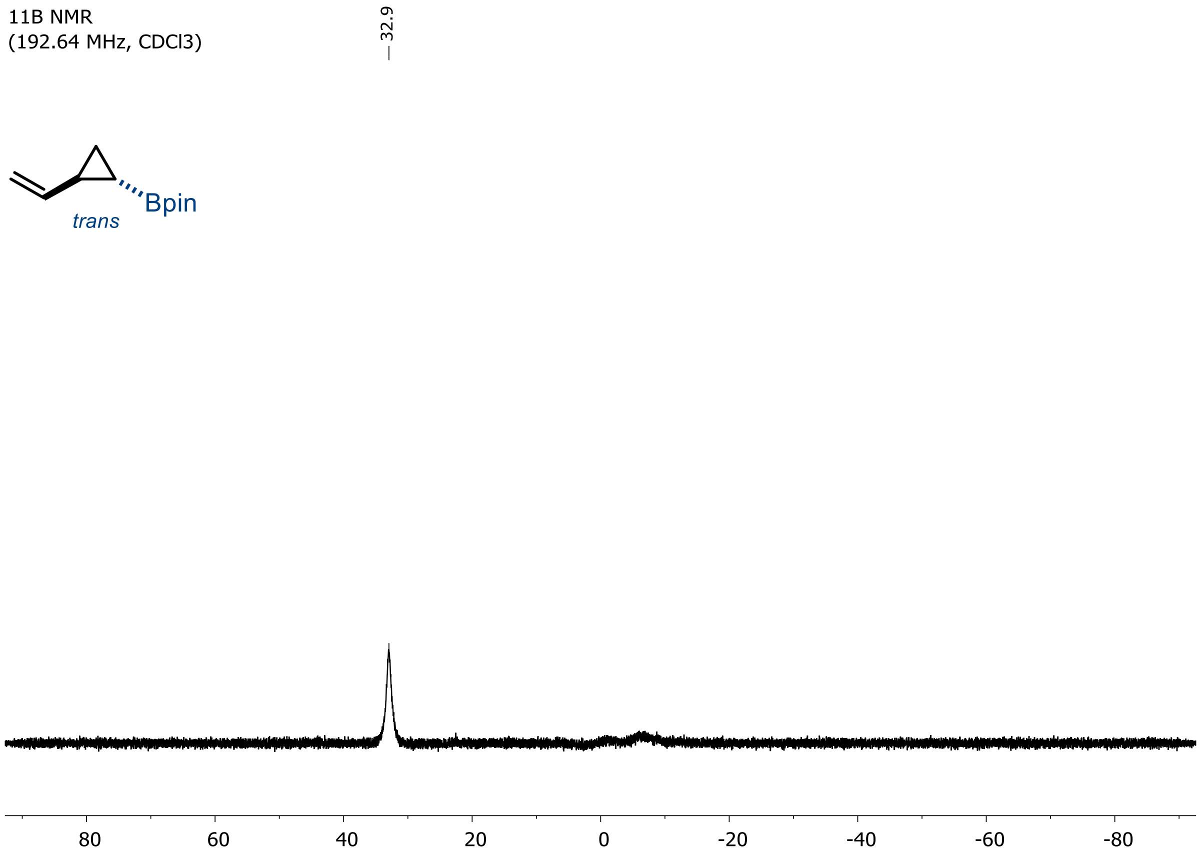

(*cis*-2-methylcyclopropyl)benzene (27)

1H NMR
(600.44 MHz, CDCl3)

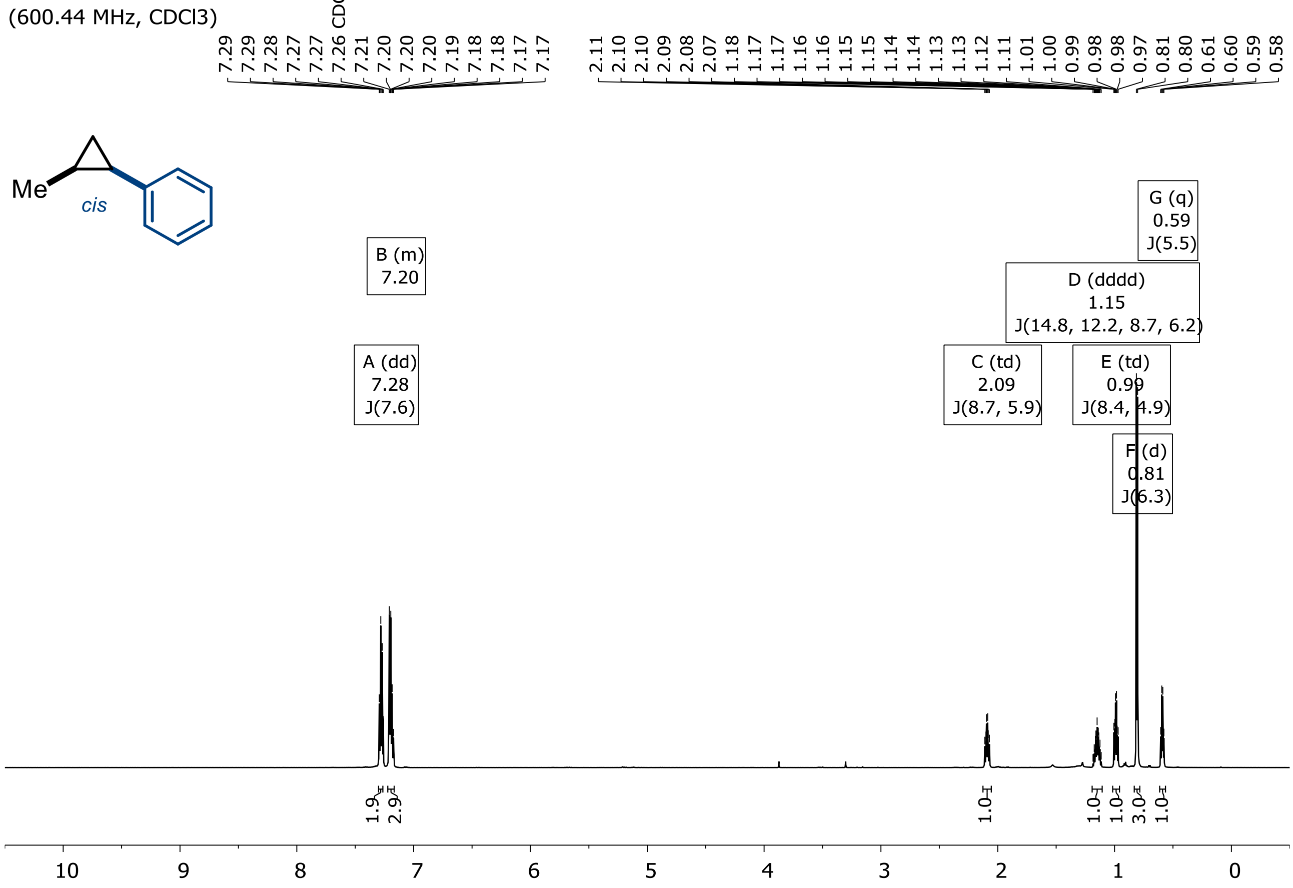

13C NMR
(151.00 MHz, CDCl3)

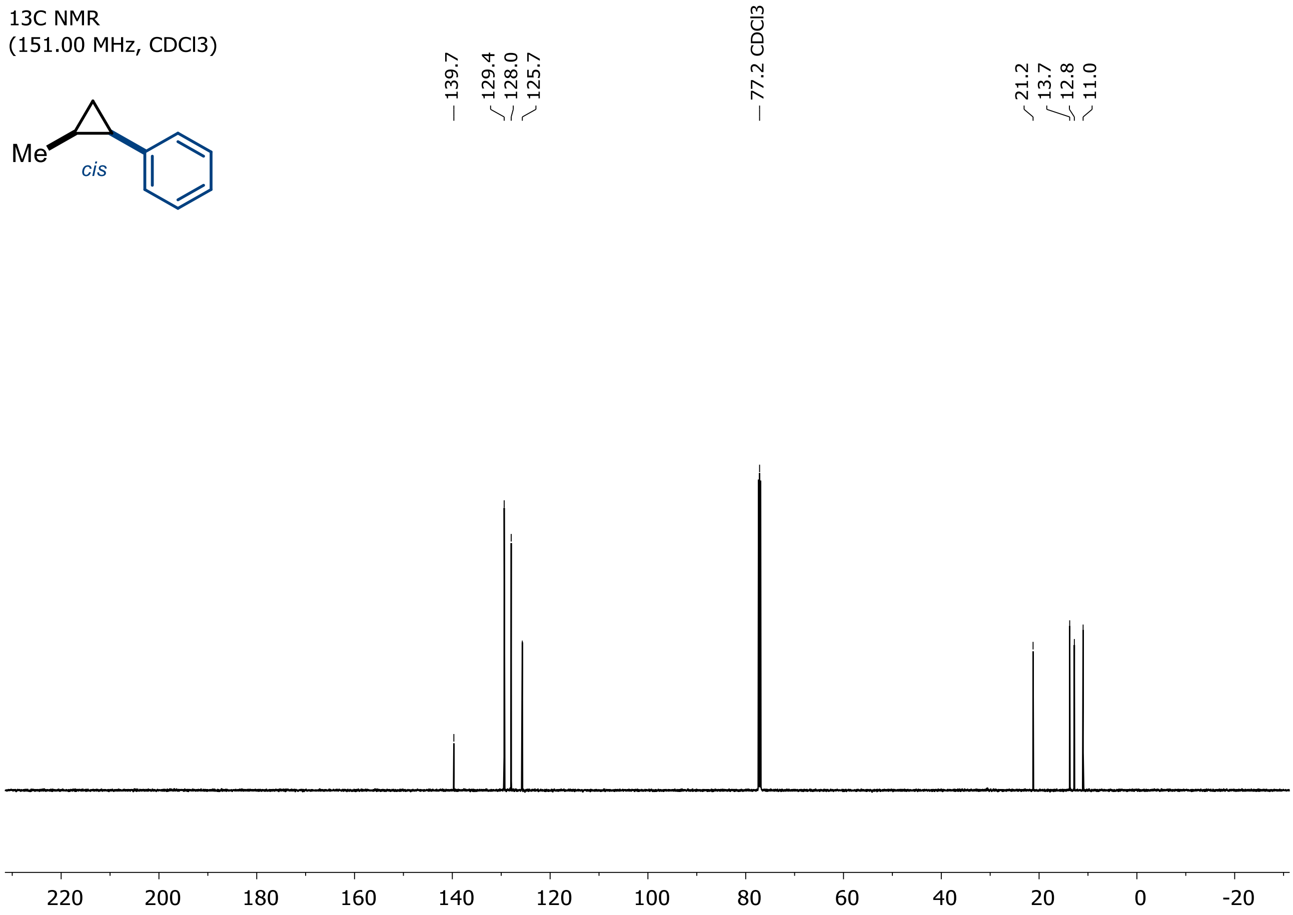

(*Z*)-1-fluoro-4-(2-(prop-1-en-1-yl)cyclopropyl)benzene (*S*28)

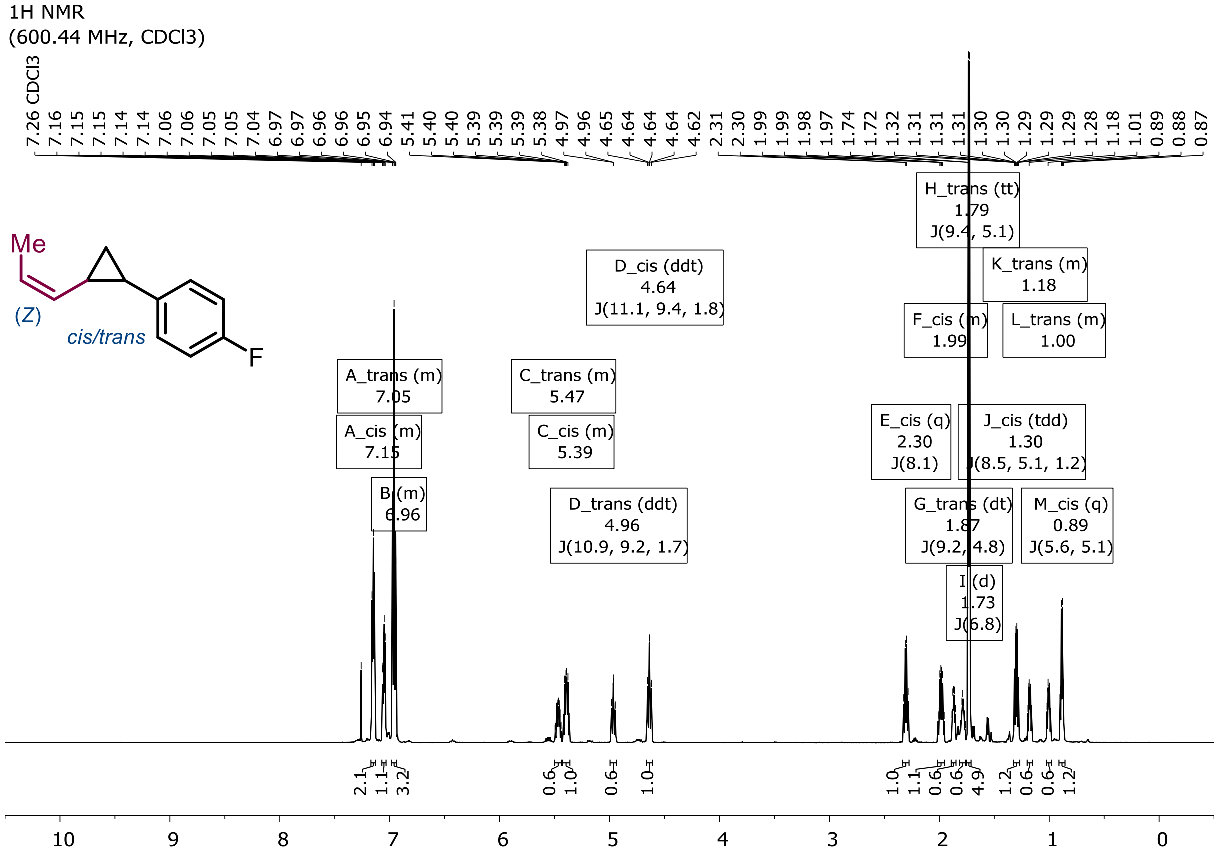

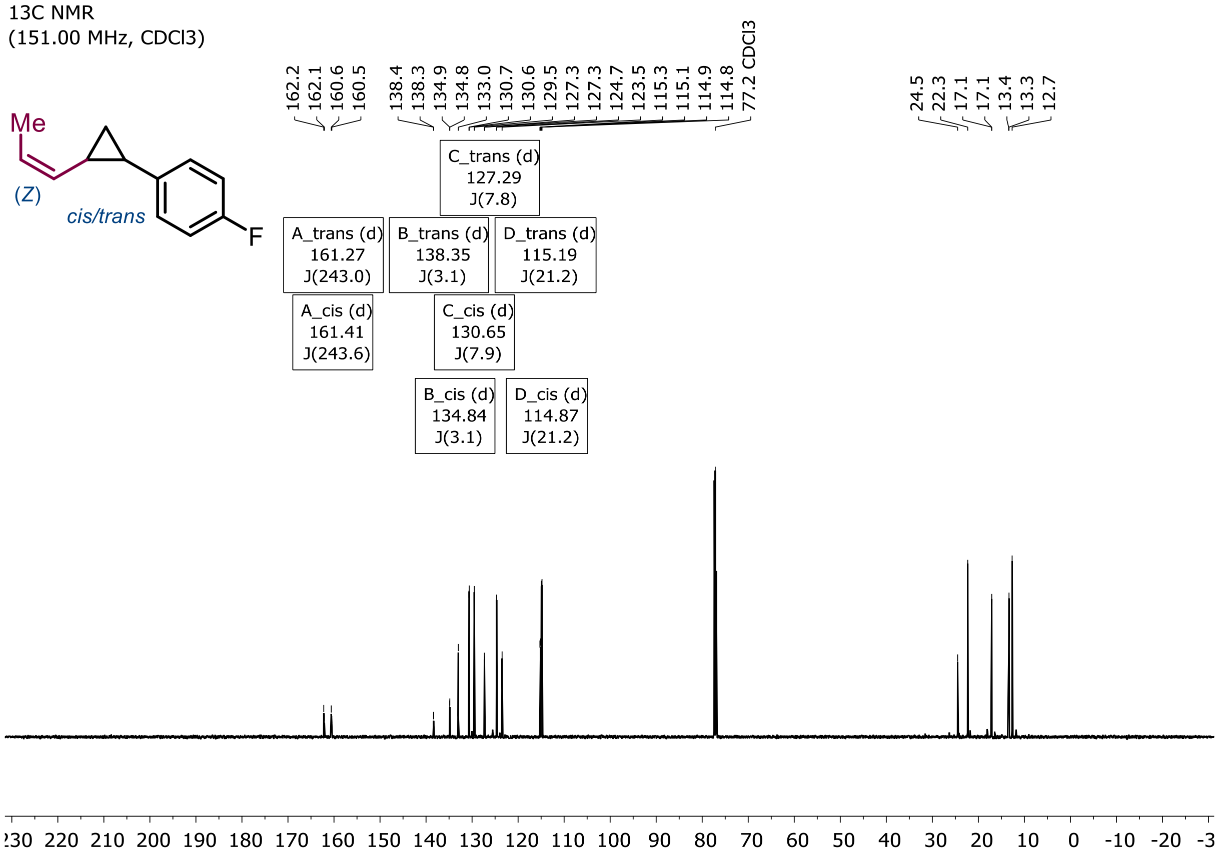

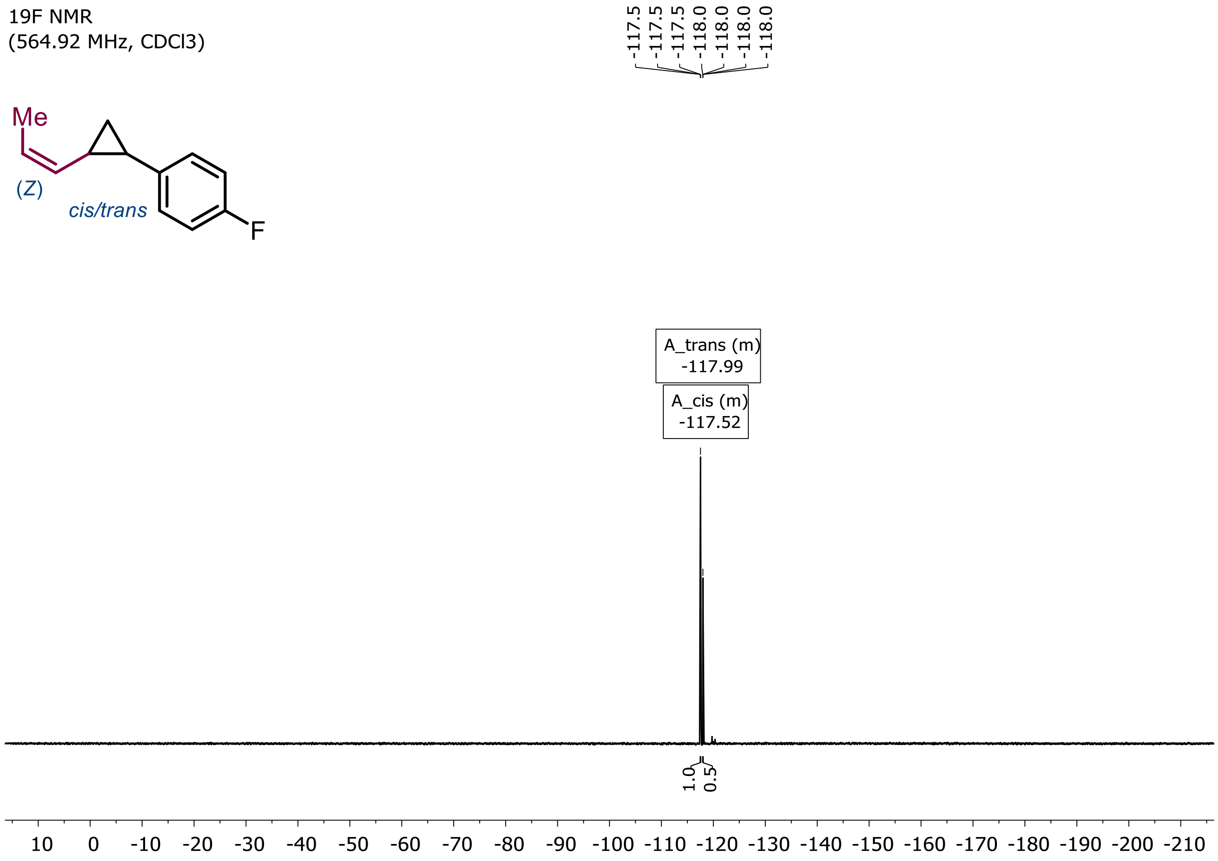

trans-(_Z_)-1-fluoro-4-(2-(prop-1-en-1-yl)cyclopropyl)benzene (28)

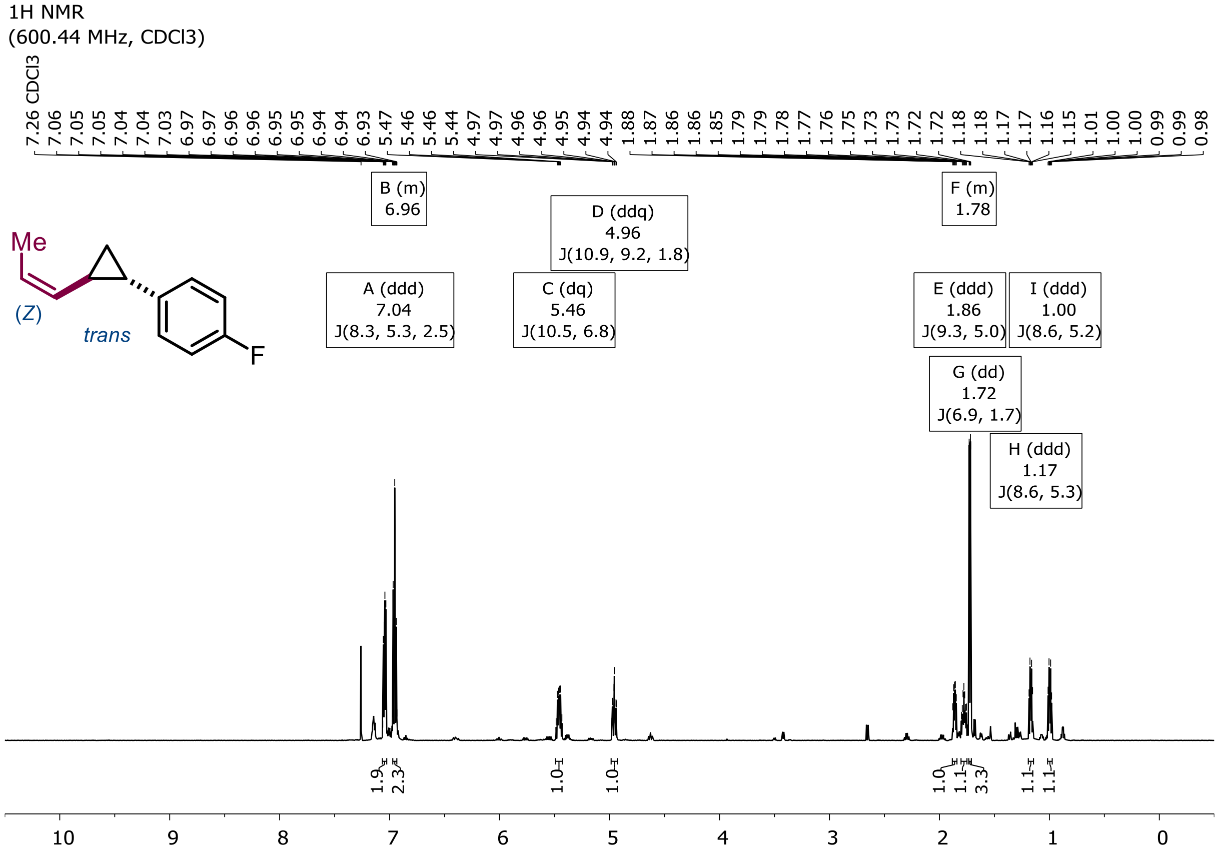

13C NMR
(151.00 MHz, CDCl3)

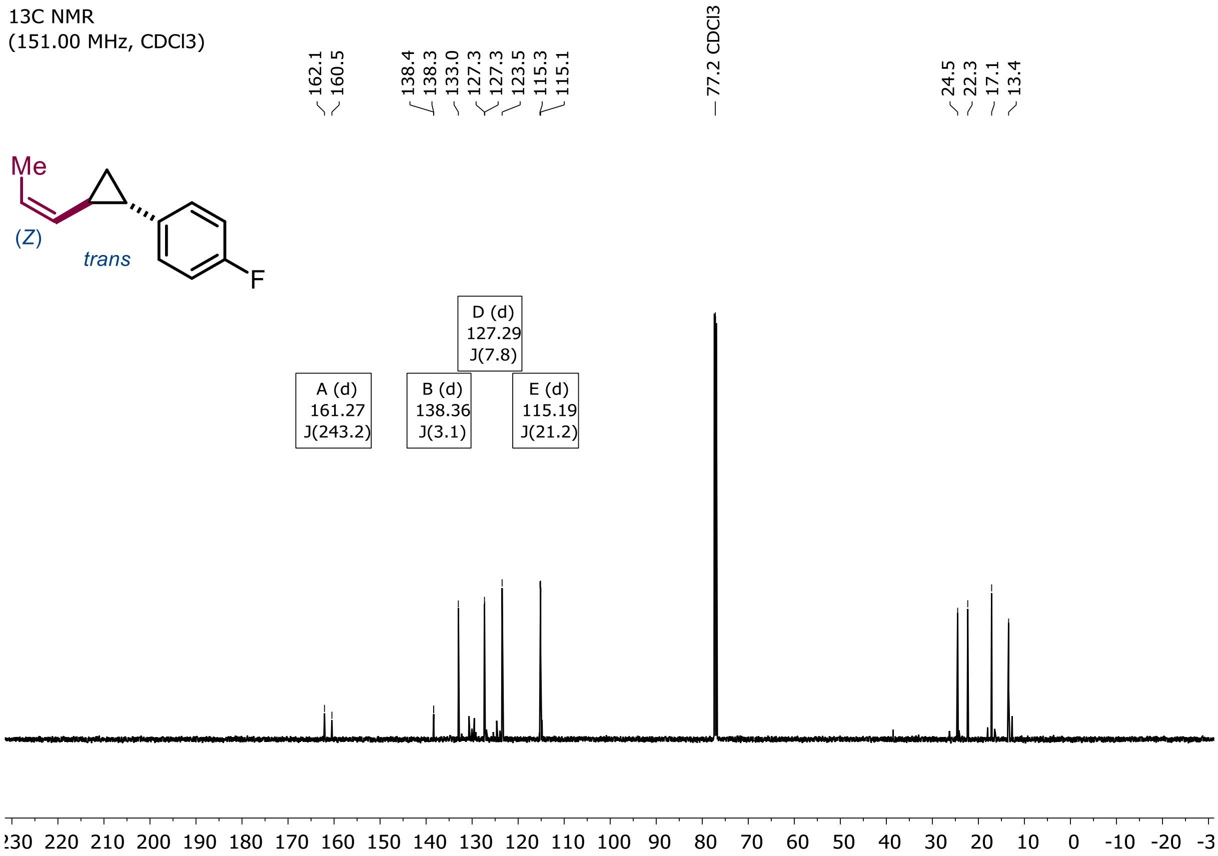

19F NMR
(564.92 MHz, CDCl3)

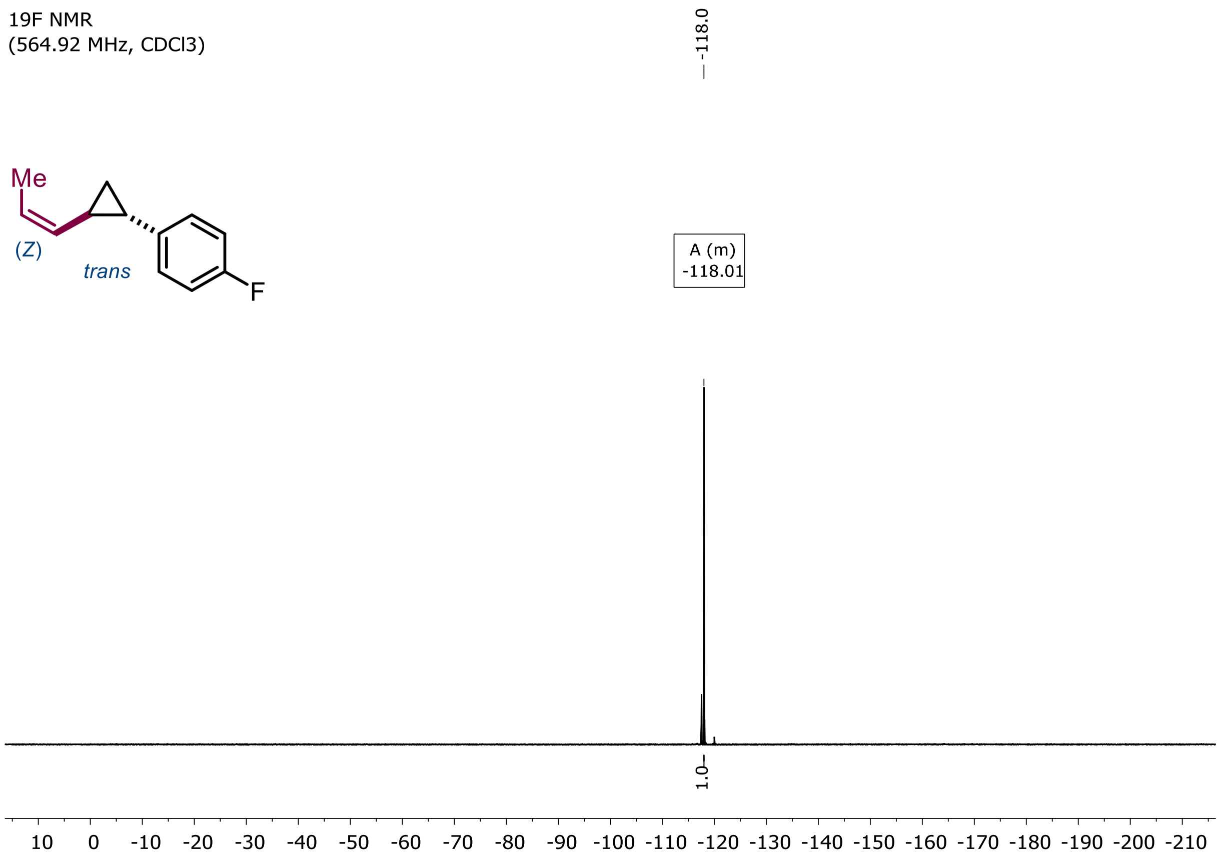

(*Z*)-5-(2-(prop-1-en-1-yl)cyclopropyl)benzo[*d*][1,3]dioxole (S29)

1H NMR
(600.44 MHz, CDCl3)

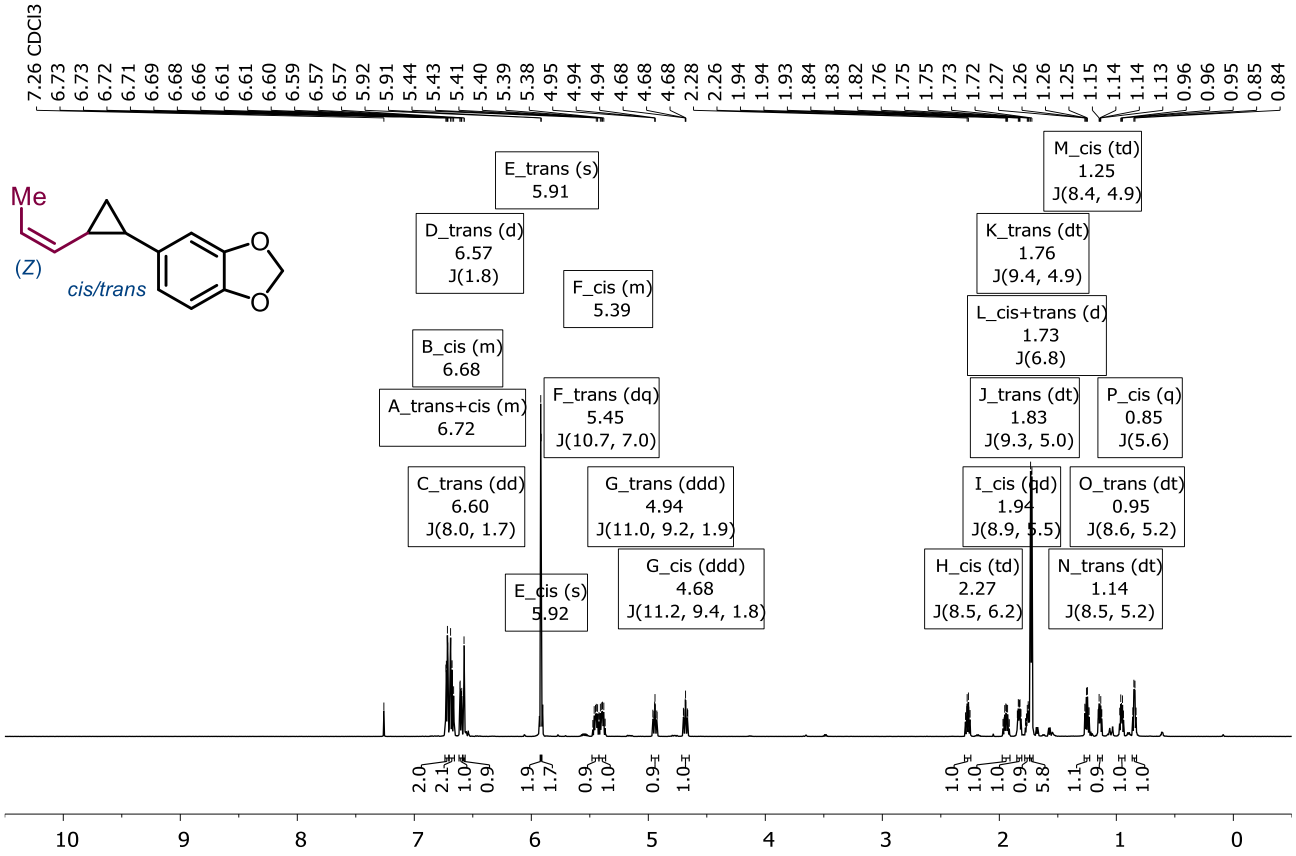

13C NMR
(151.00 MHz, CDCl3)

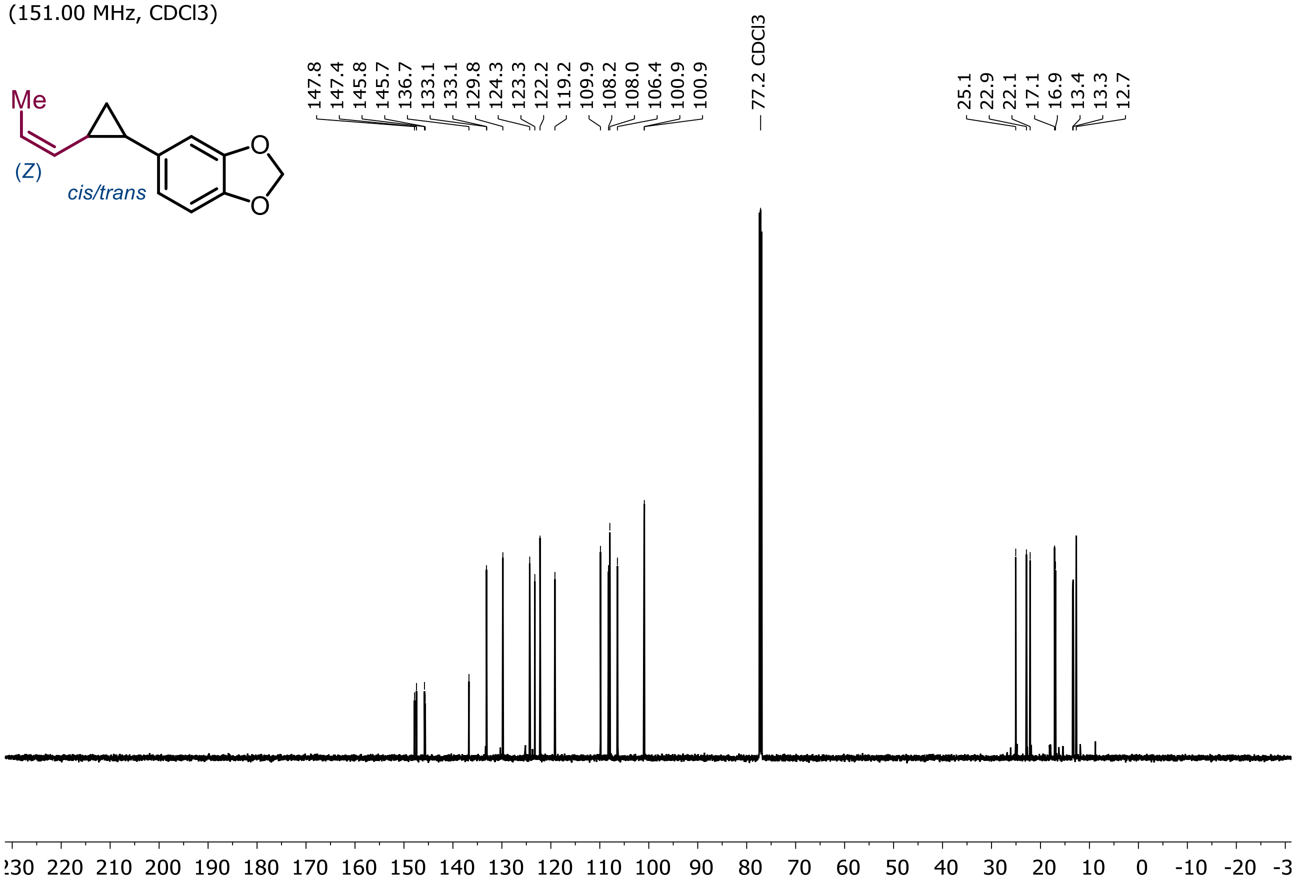

trans-(*Z*)-5-(2-(prop-1-en-1-yl)cyclopropyl)benzo[*d*][1,3]dioxole (29)

1H NMR
(599.86 MHz, CDCl3)

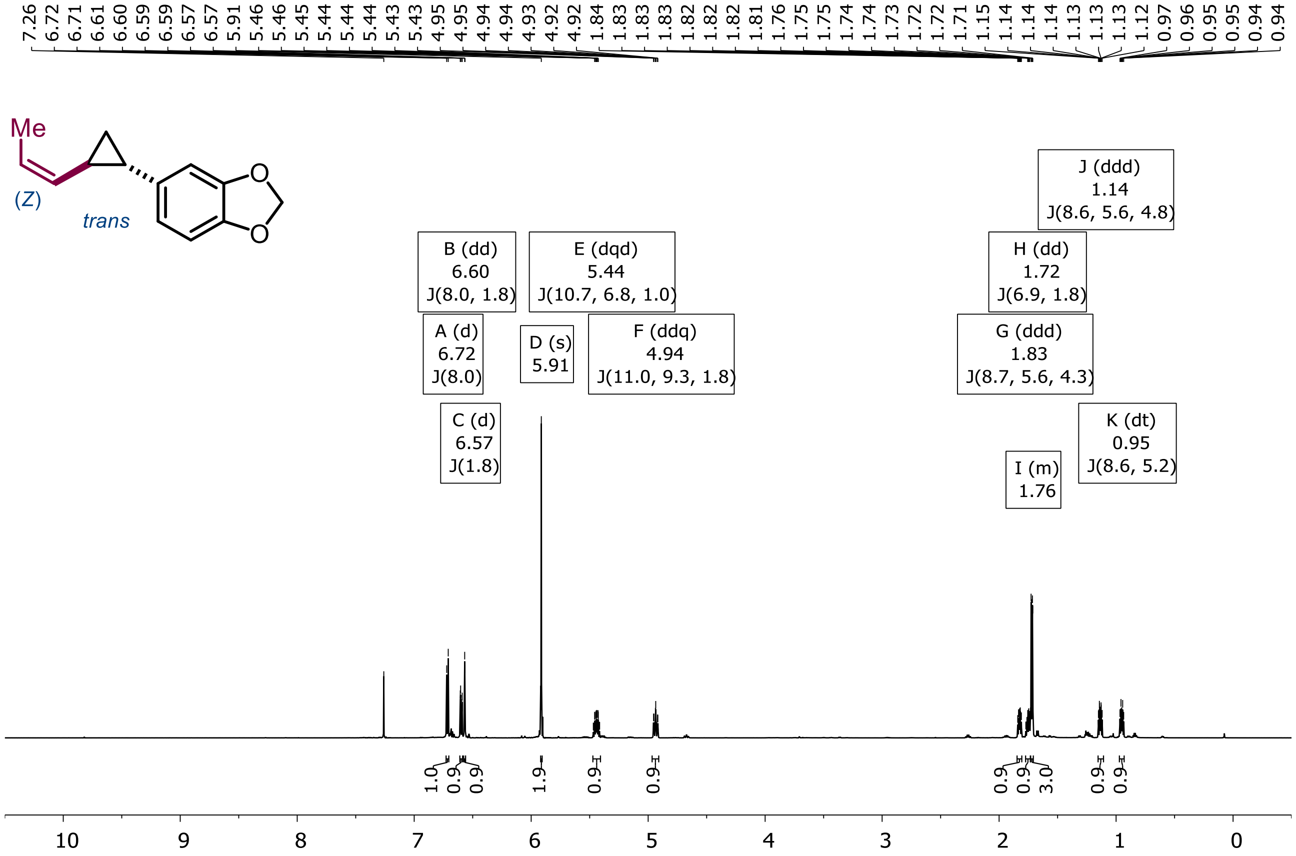

13C NMR
(150.85 MHz, CDCl3)

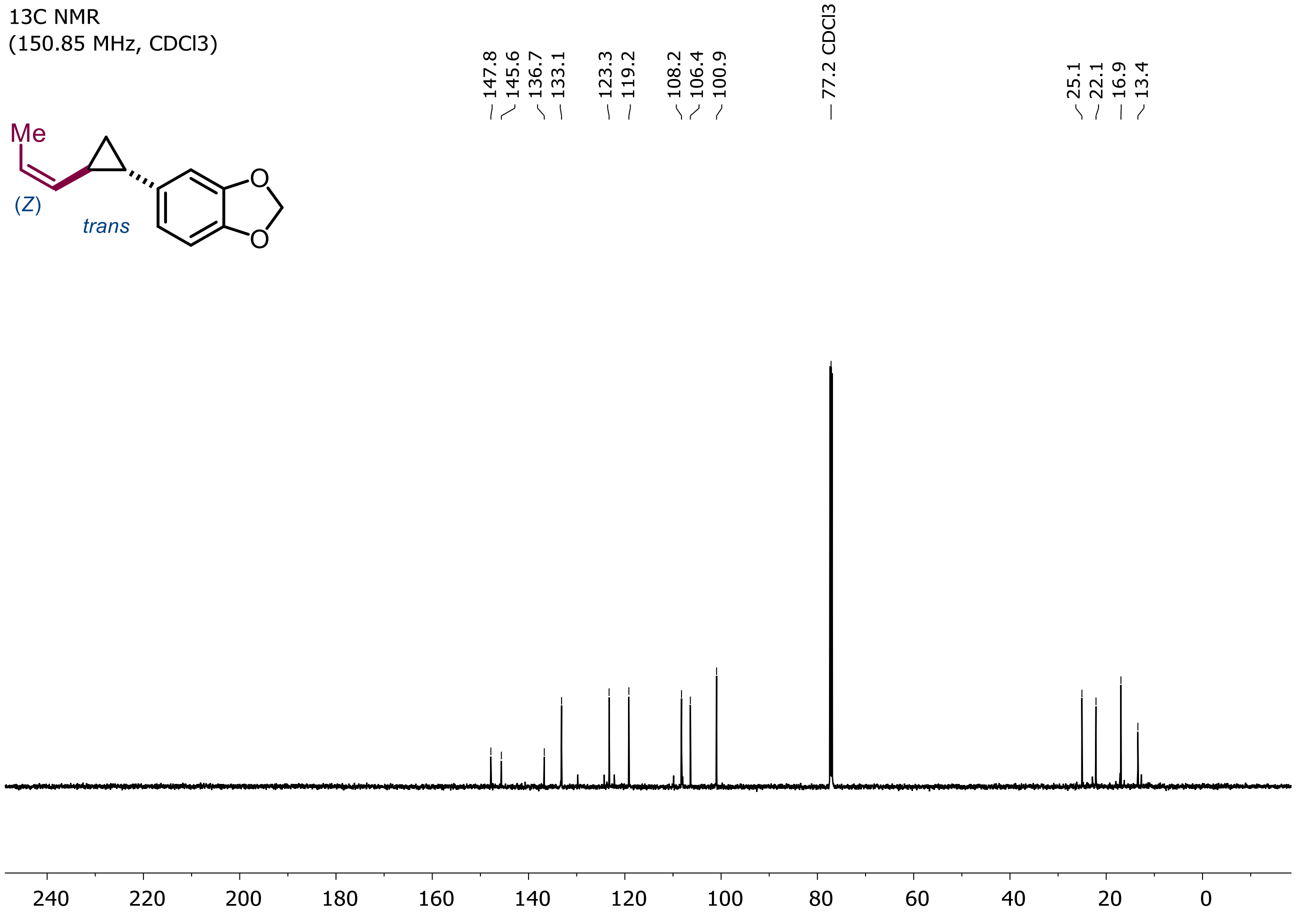

1-chloro-2-(2-(phenylethynyl)cyclopropyl)benzene

1H NMR
(600.44 MHz, CDCl3)

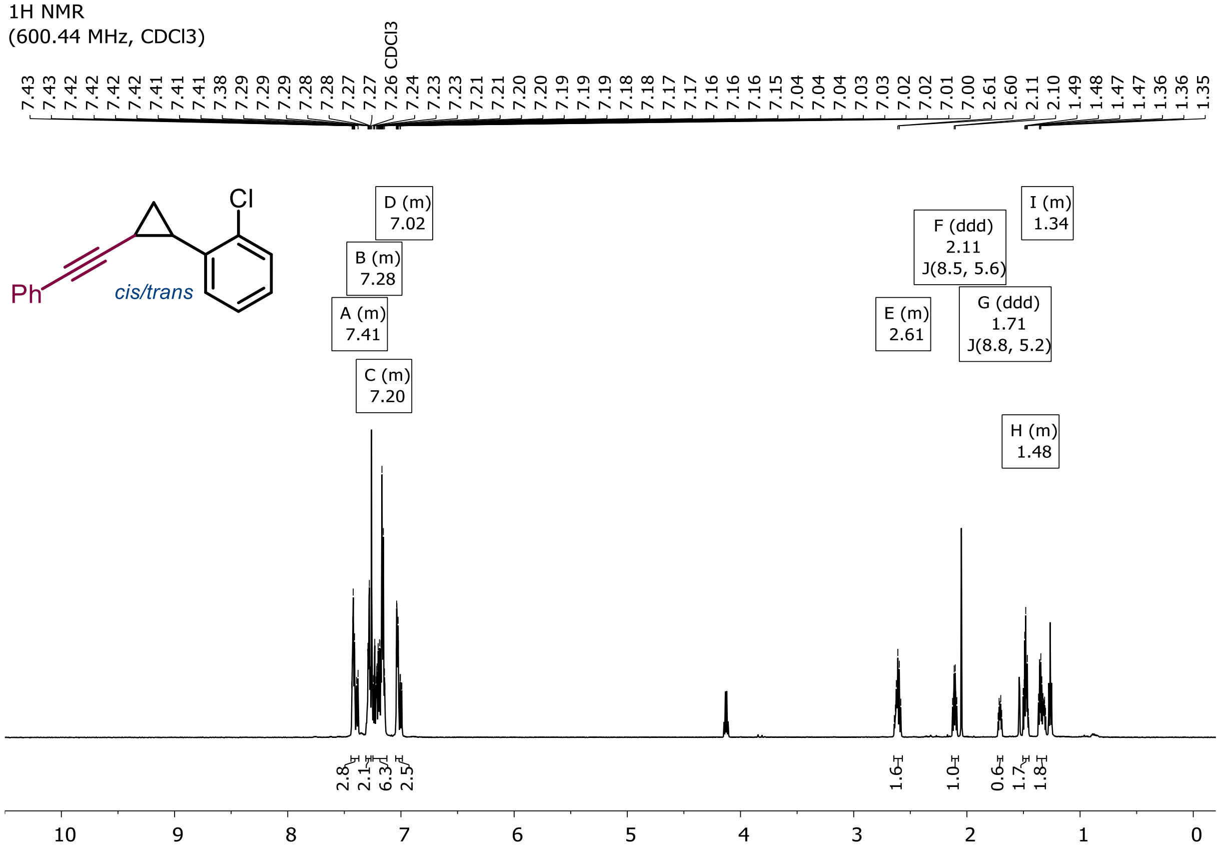

(*Z*)-1-chloro-2-(2-styrylcyclopropyl)benzene (S30)

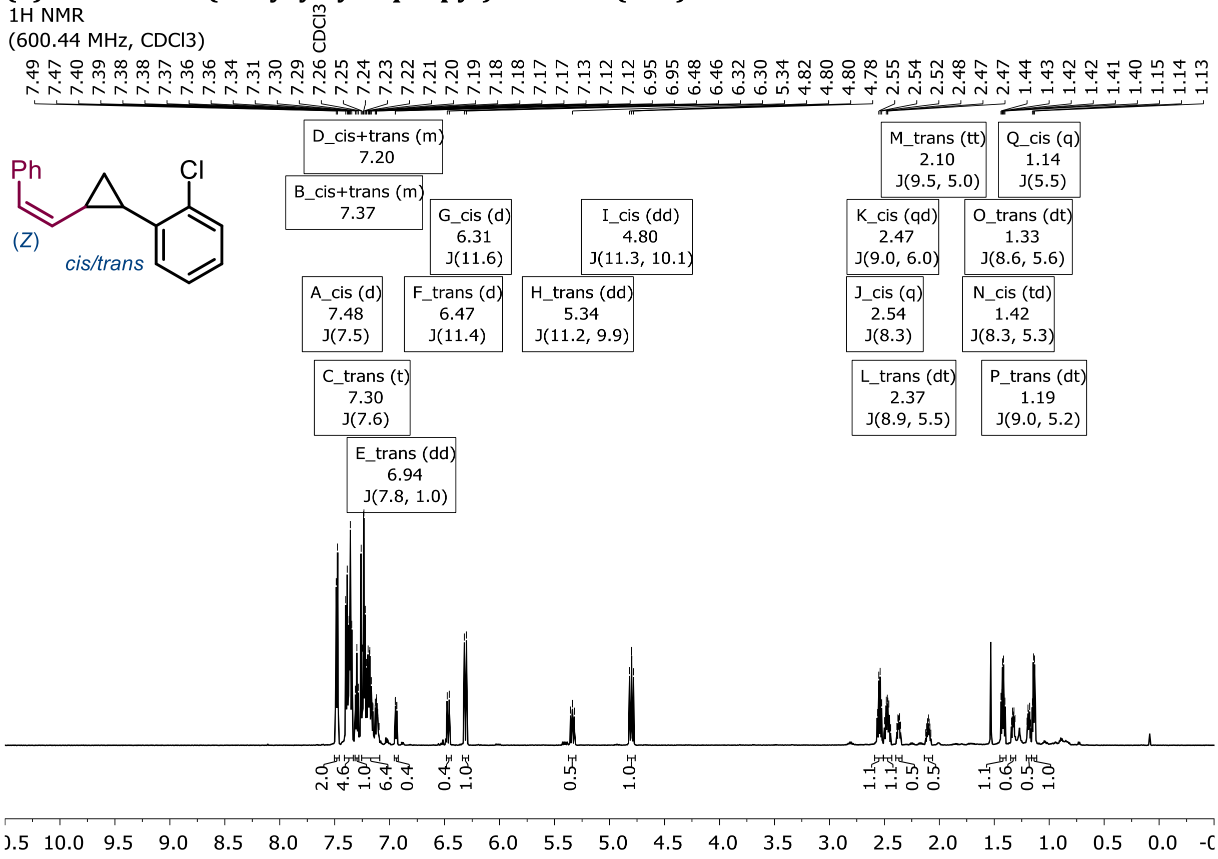

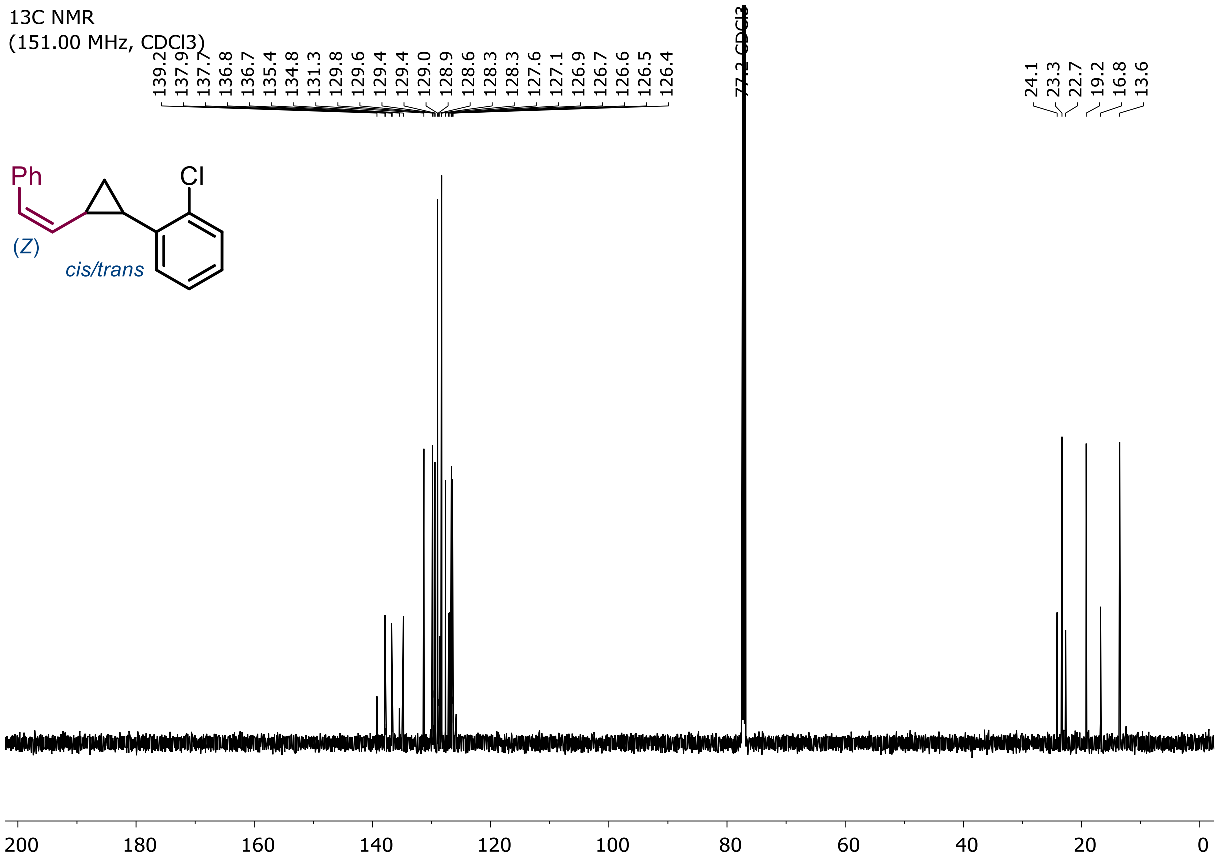

***trans*-(*Z*)-1-chloro-2-(2-styrylcyclopropyl)benzene (30)**

1H NMR
(599.86 MHz, CDCl3)

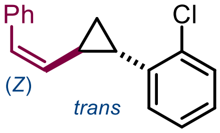

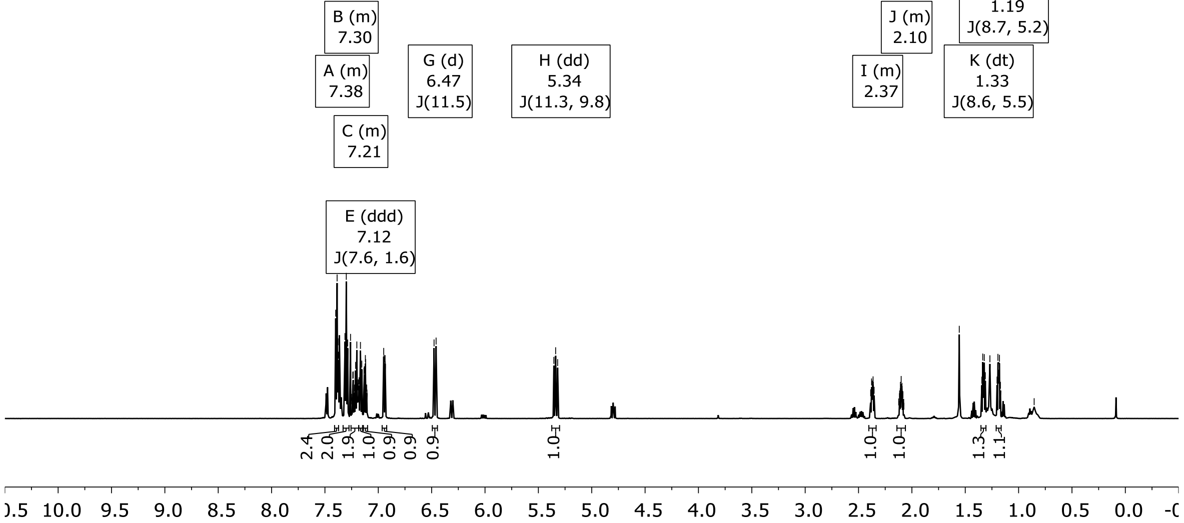

13C NMR
(150.85 MHz, CDCl3)

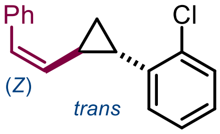

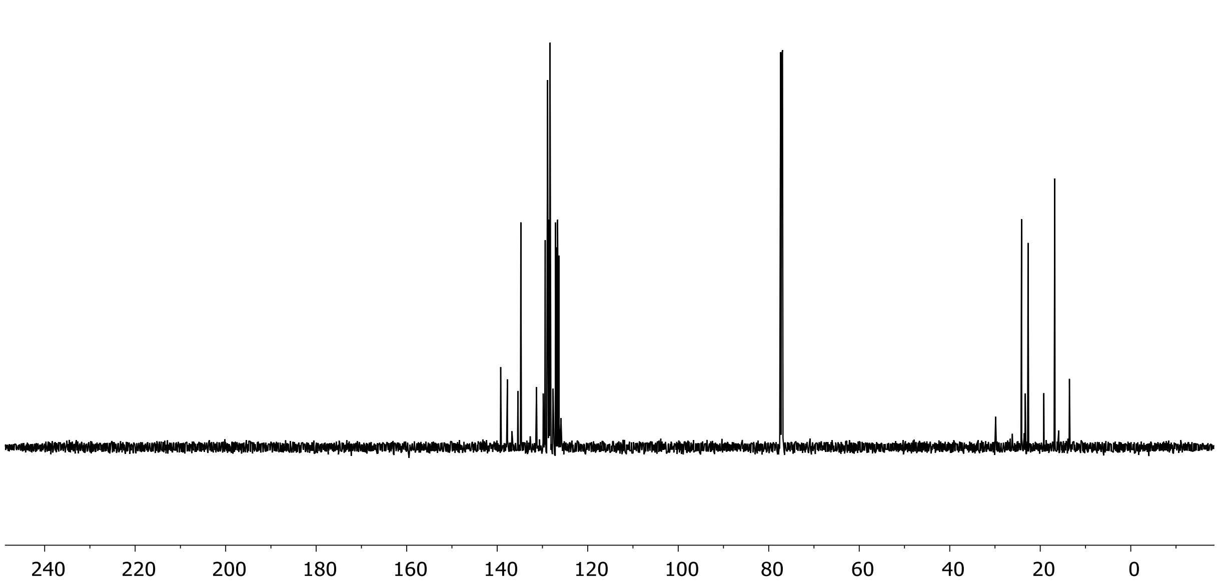

2-(2-(4-methoxyphenyl)cyclopropyl)-4,4,5,5-tetramethyl-1,3,2-dioxaborolane

1H NMR
(600.44 MHz, CDCl3)

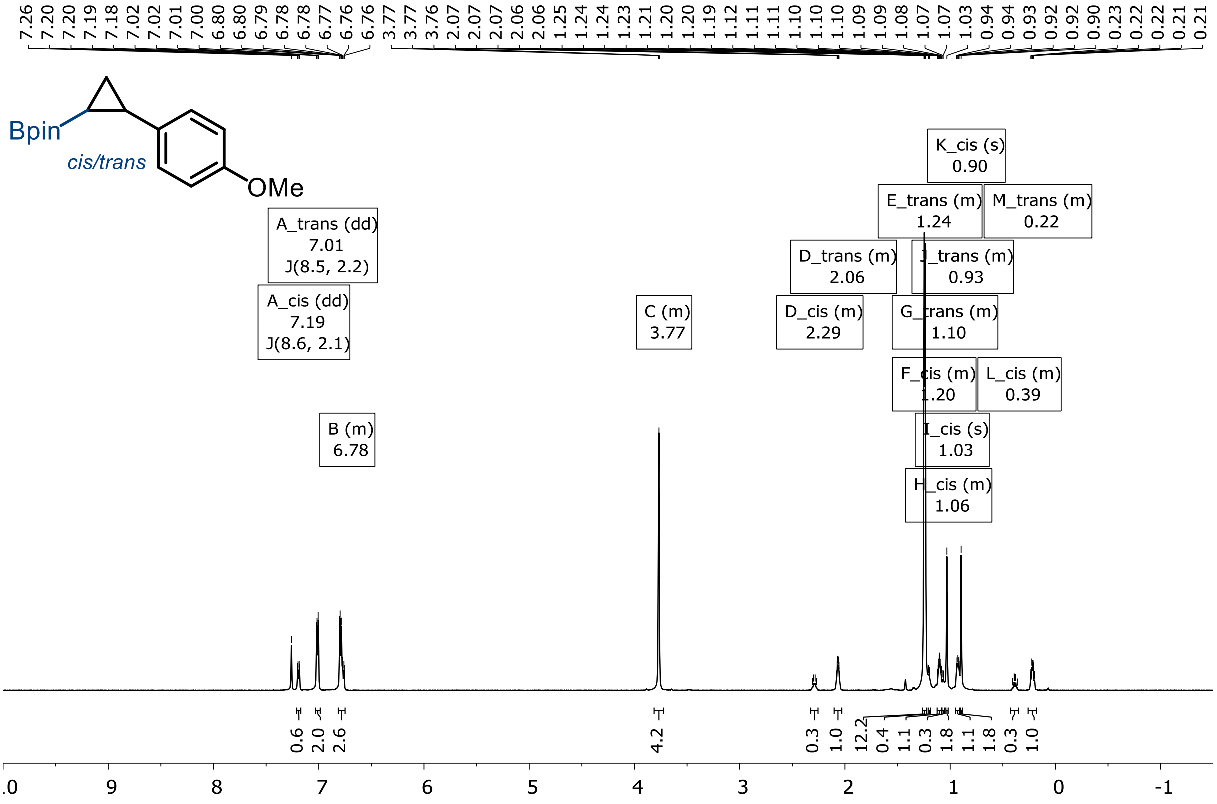

13C NMR
(151.00 MHz, CDCl3)

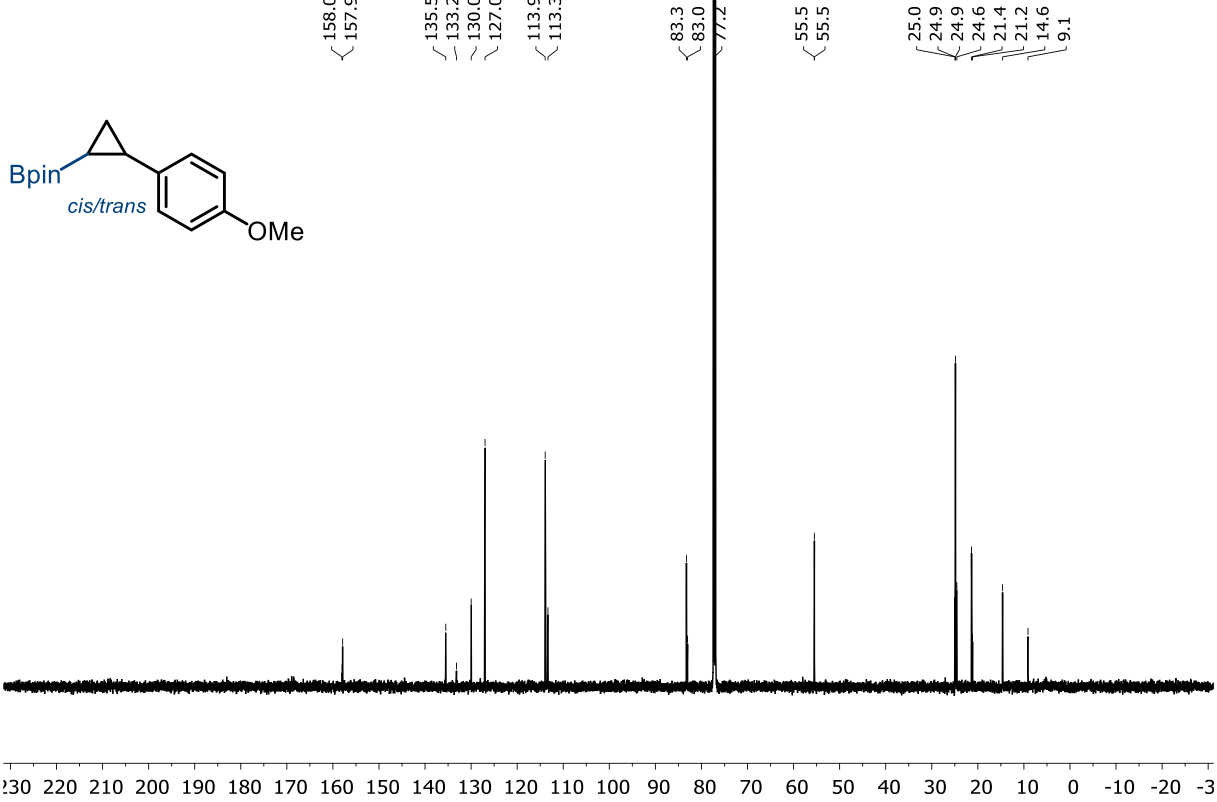

(*Z*)-1-methoxy-4-(2-(oct-1-en-1-yl)cyclopropyl)benzene (S31)

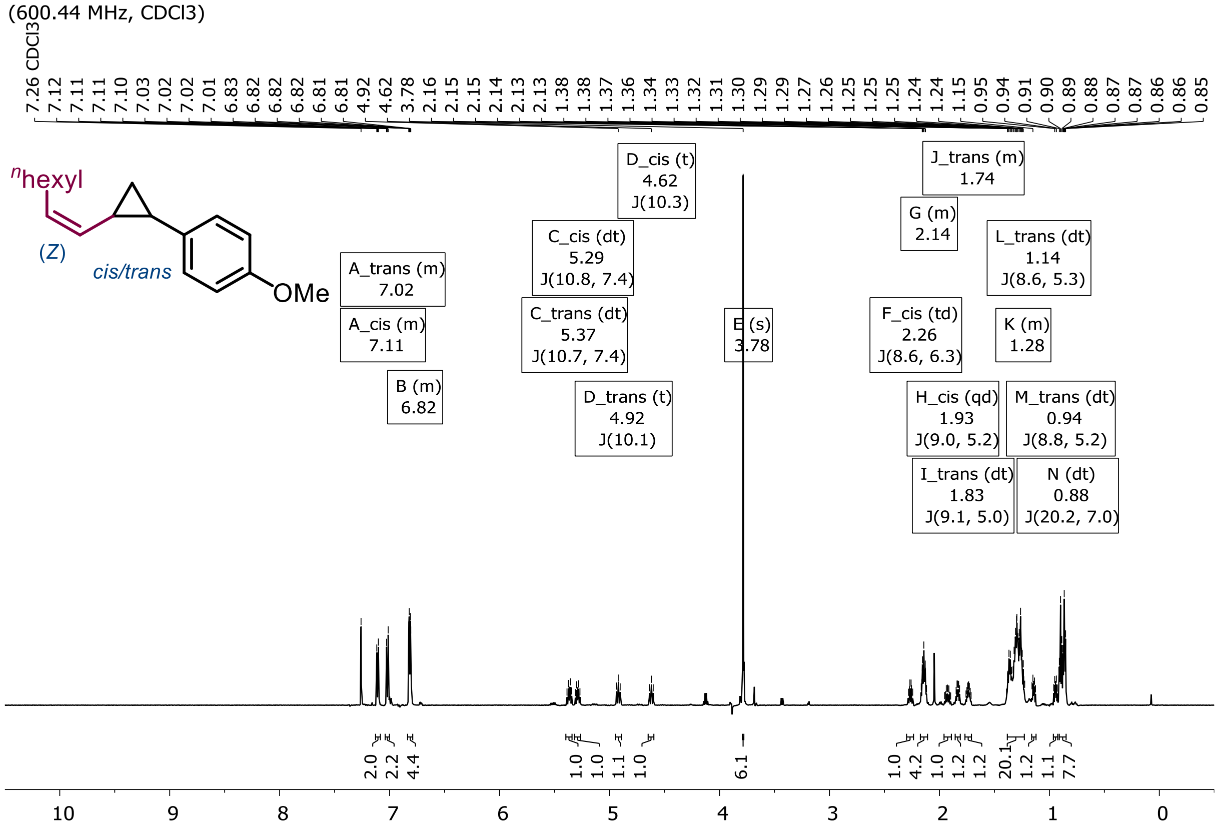

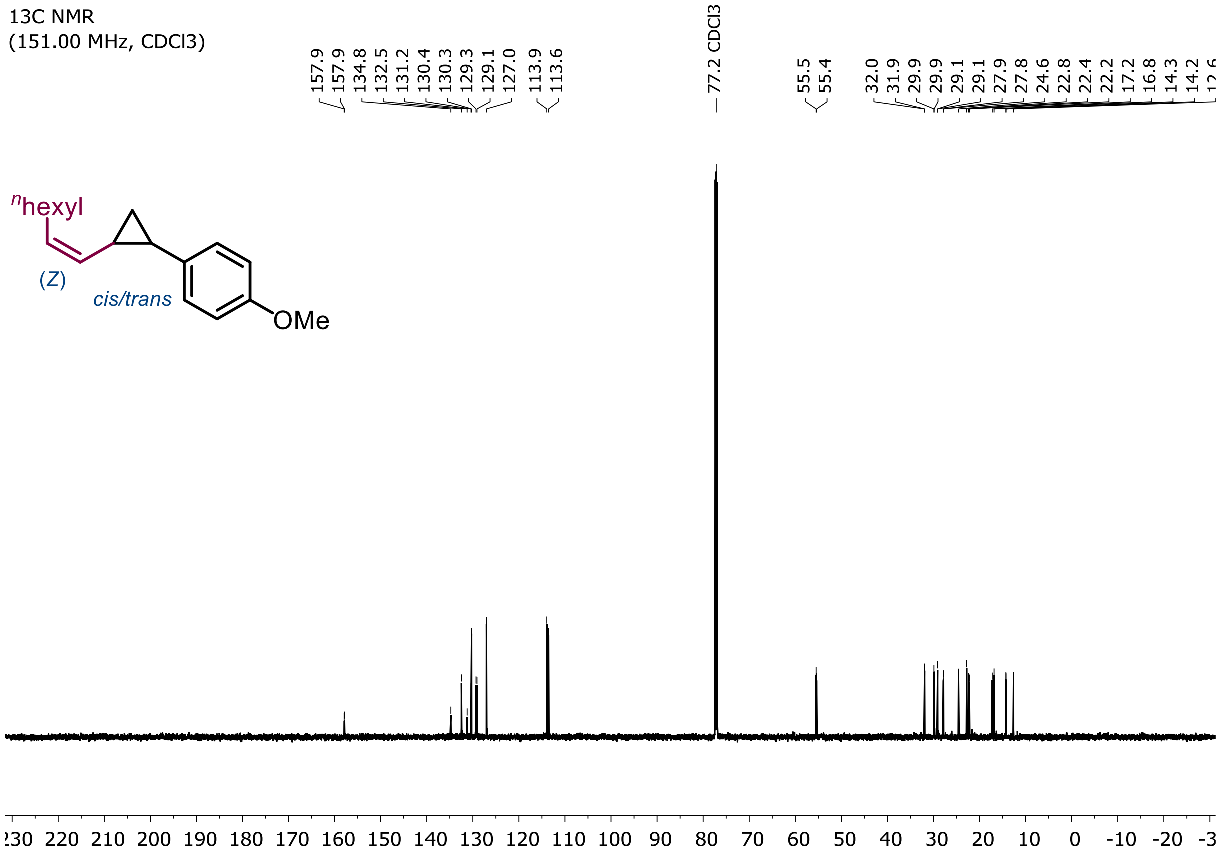

***trans*-(*Z*)-1-methoxy-4-(2-(oct-1-en-1-yl)cyclopropyl)benzene (31)**

1H NMR
(600.44 MHz, CDCl3)

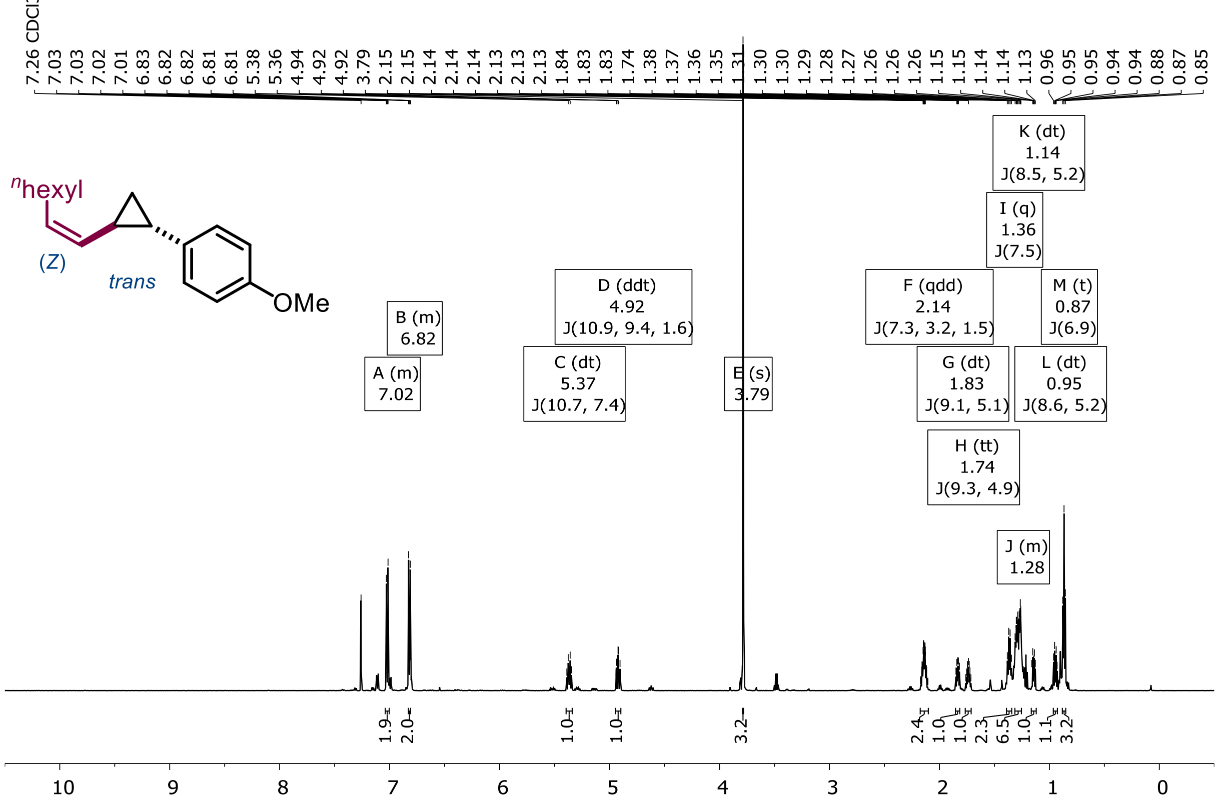

13C NMR
(151.00 MHz, CDCl3)

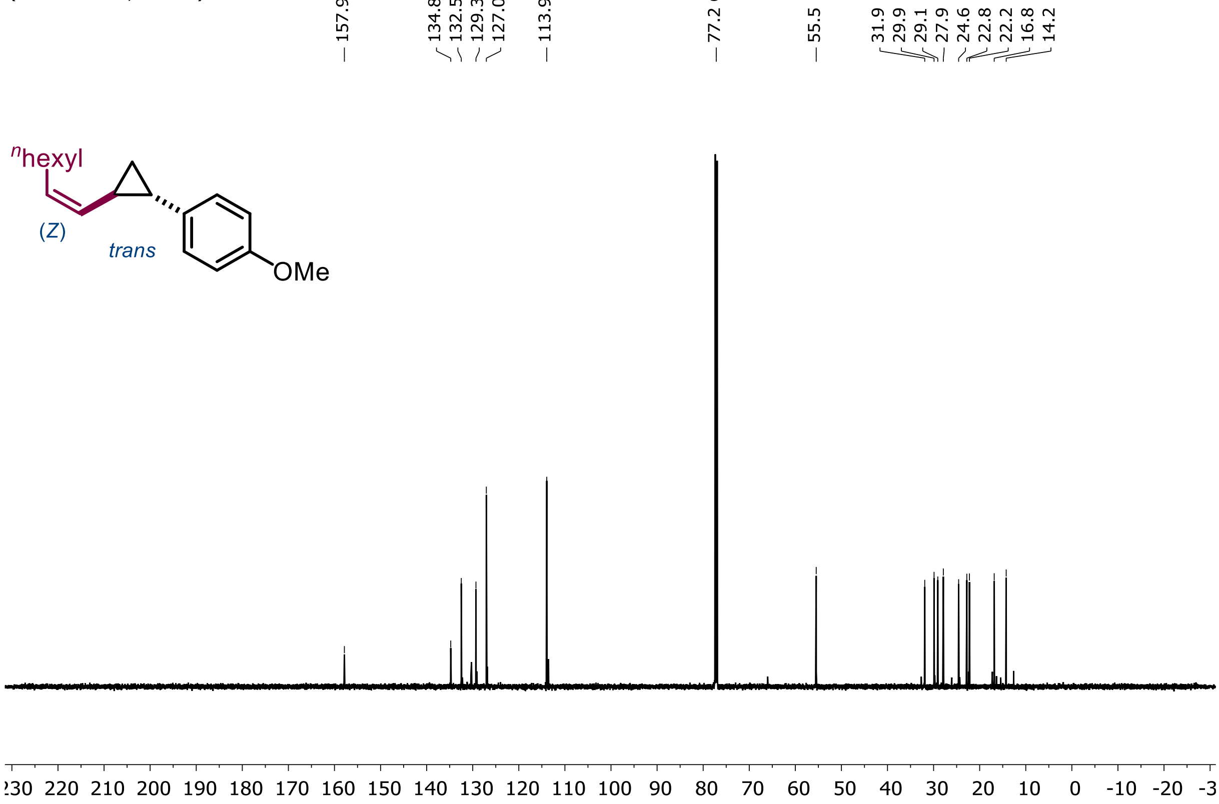

(*E*)-1-methoxy-4-(2-(oct-1-en-1-yl)cyclopropyl)benzene (S32)

1H NMR
(600.44 MHz, CDCl3)

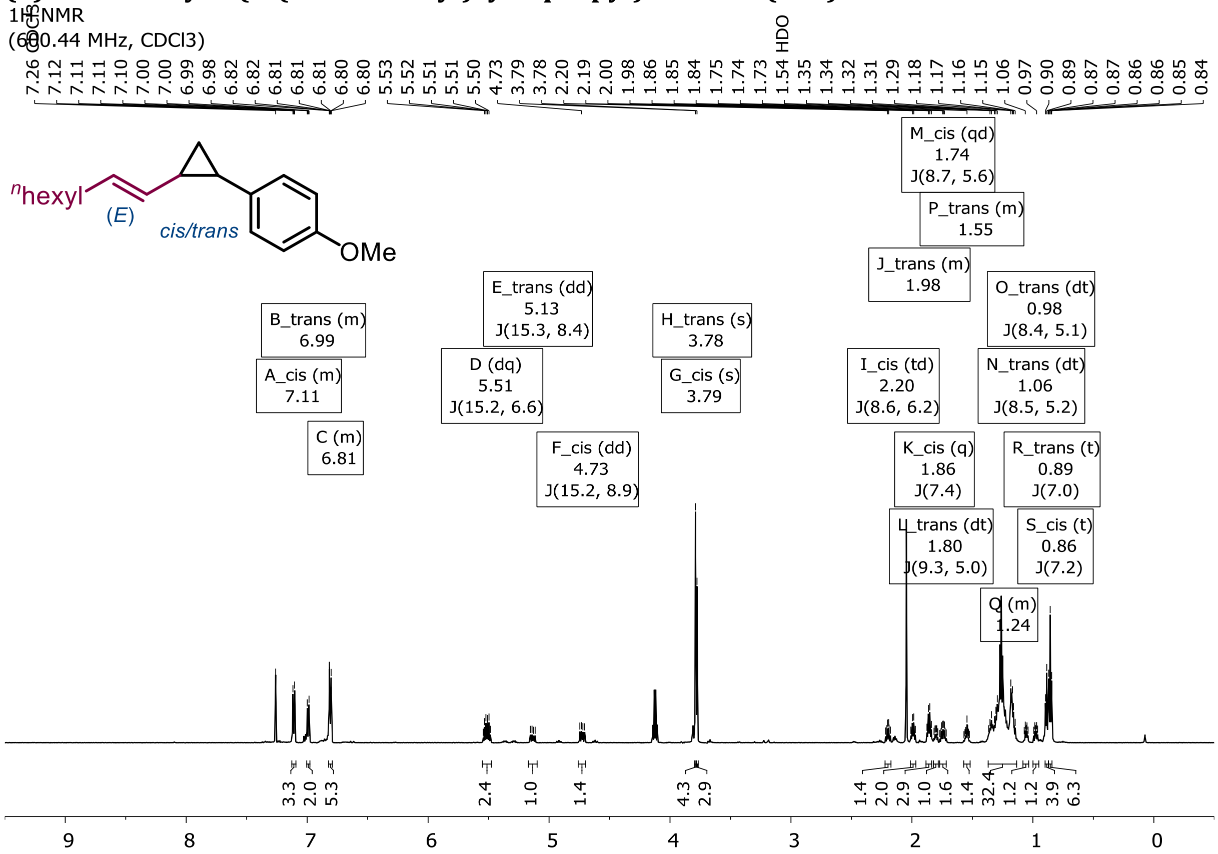

13C NMR
(151.00 MHz, CDCl3)

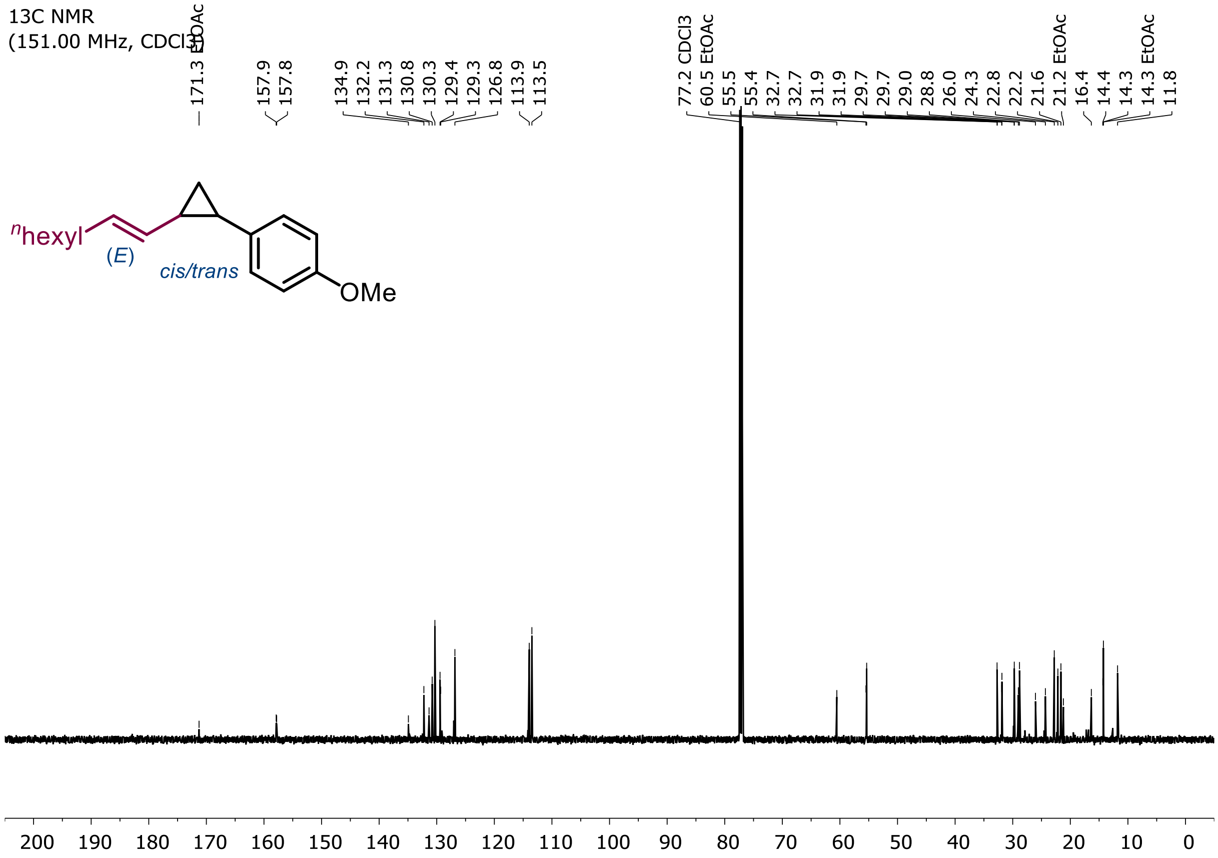

trans-(*E*)-1-methoxy-4-(2-(oct-1-en-1-yl)cyclopropyl)benzene (32)

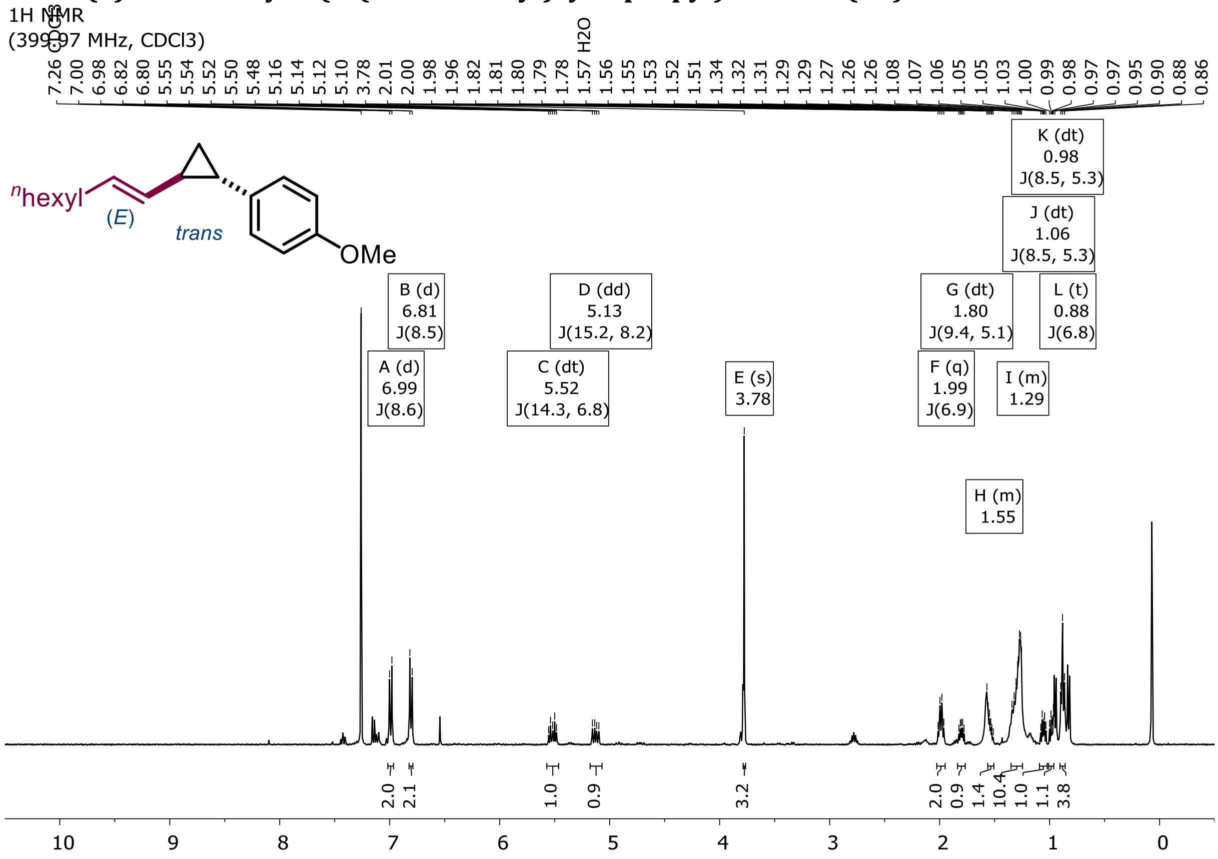

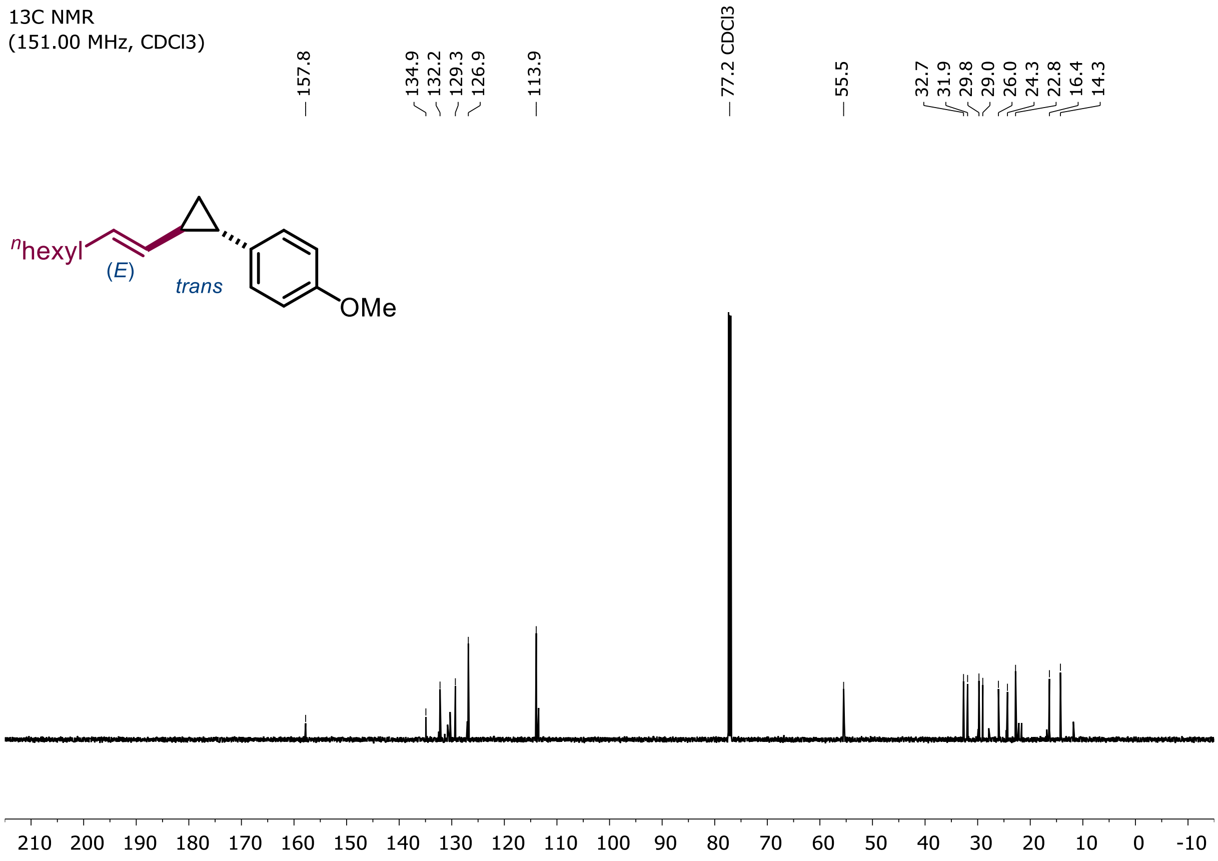

1-(2-(cyclohex-1-en-1-yl)cyclopropyl)-4-methoxybenzene (S33)

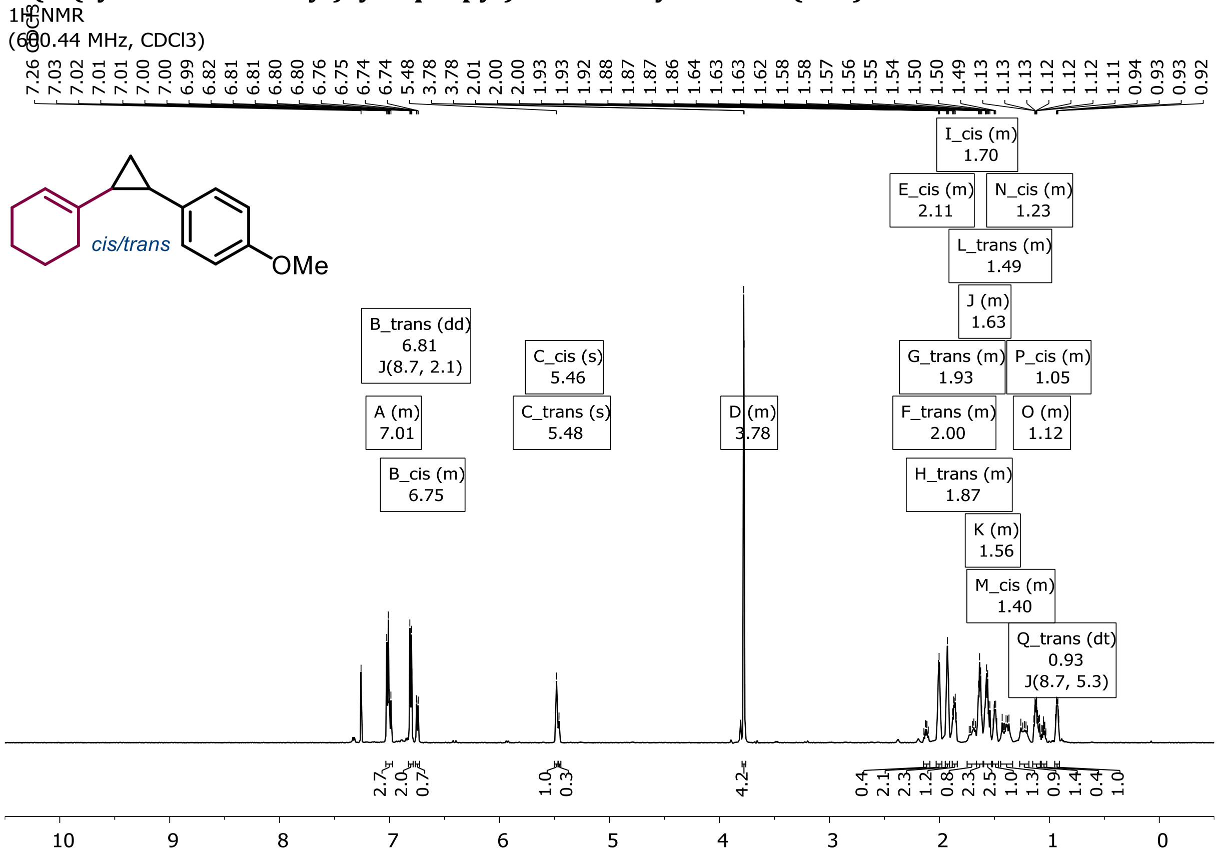

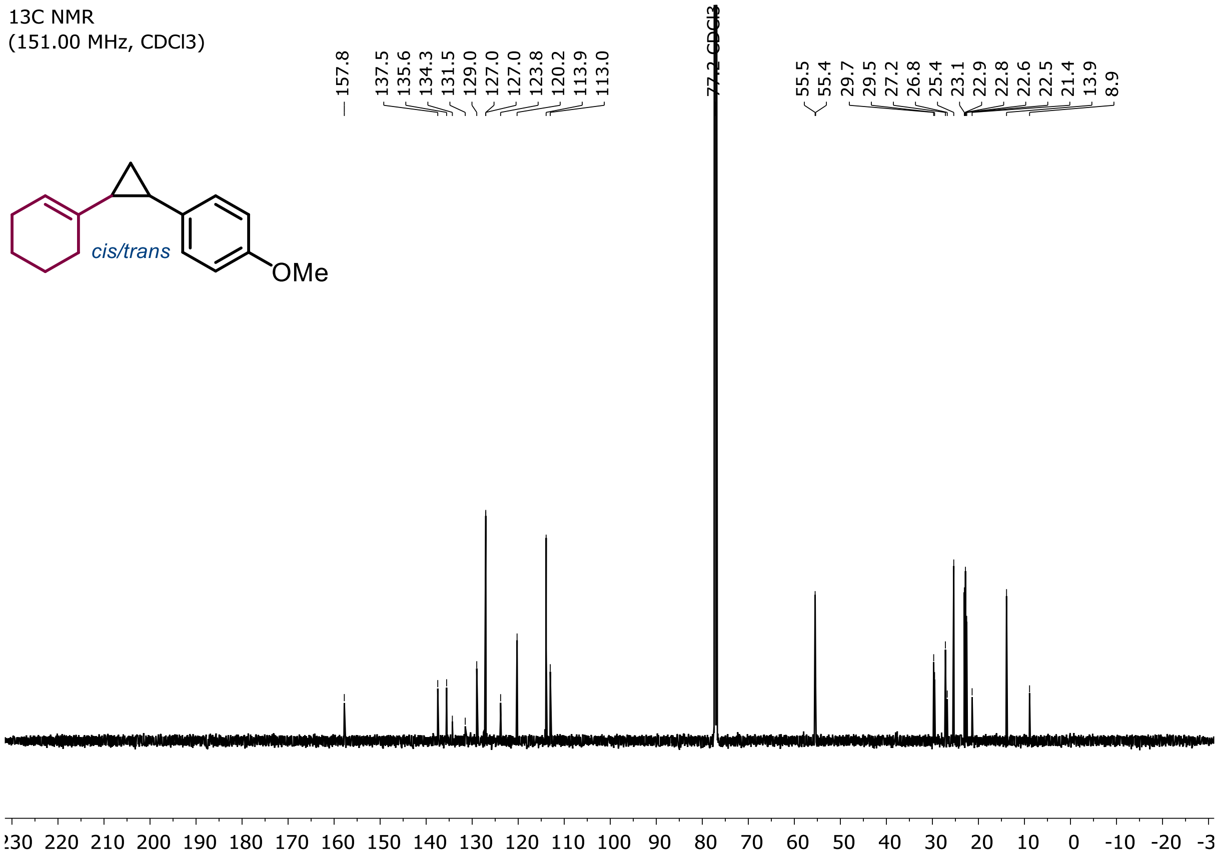

trans-1-(2-(cyclohex-1-en-1-yl)cyclopropyl)-4-methoxybenzene (33)

1H NMR
(600.44 MHz, CDCl3)

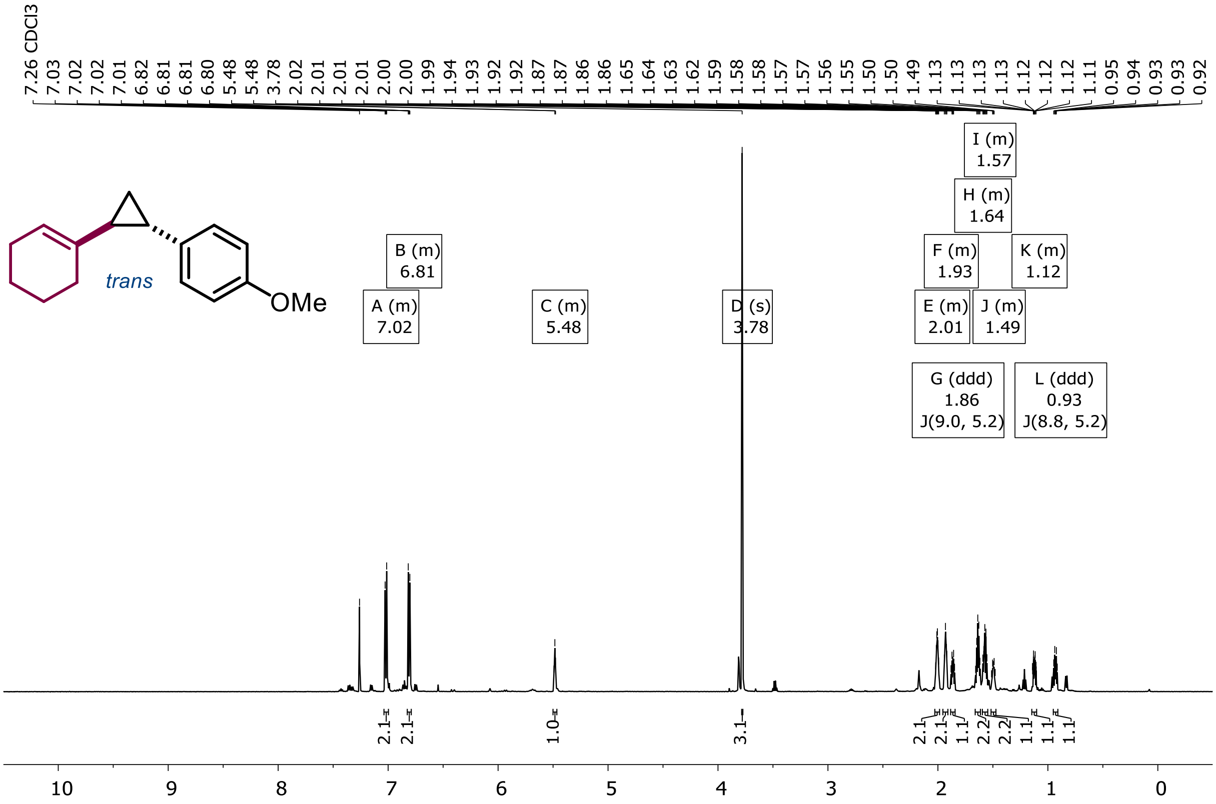

13C NMR
(151.00 MHz, CDCl3)

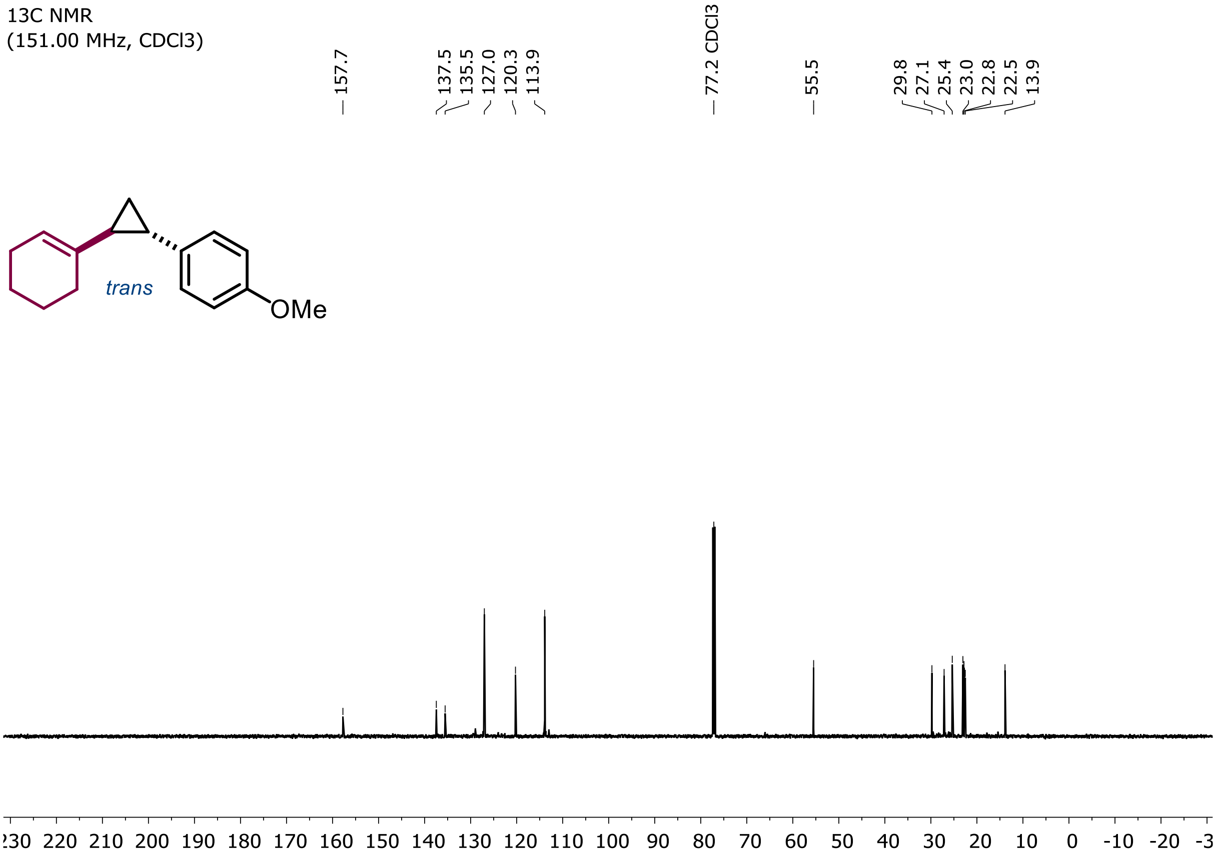

1-methoxy-4-(2-(2-methylprop-1-en-1-yl)cyclopropyl)benzene (S34)

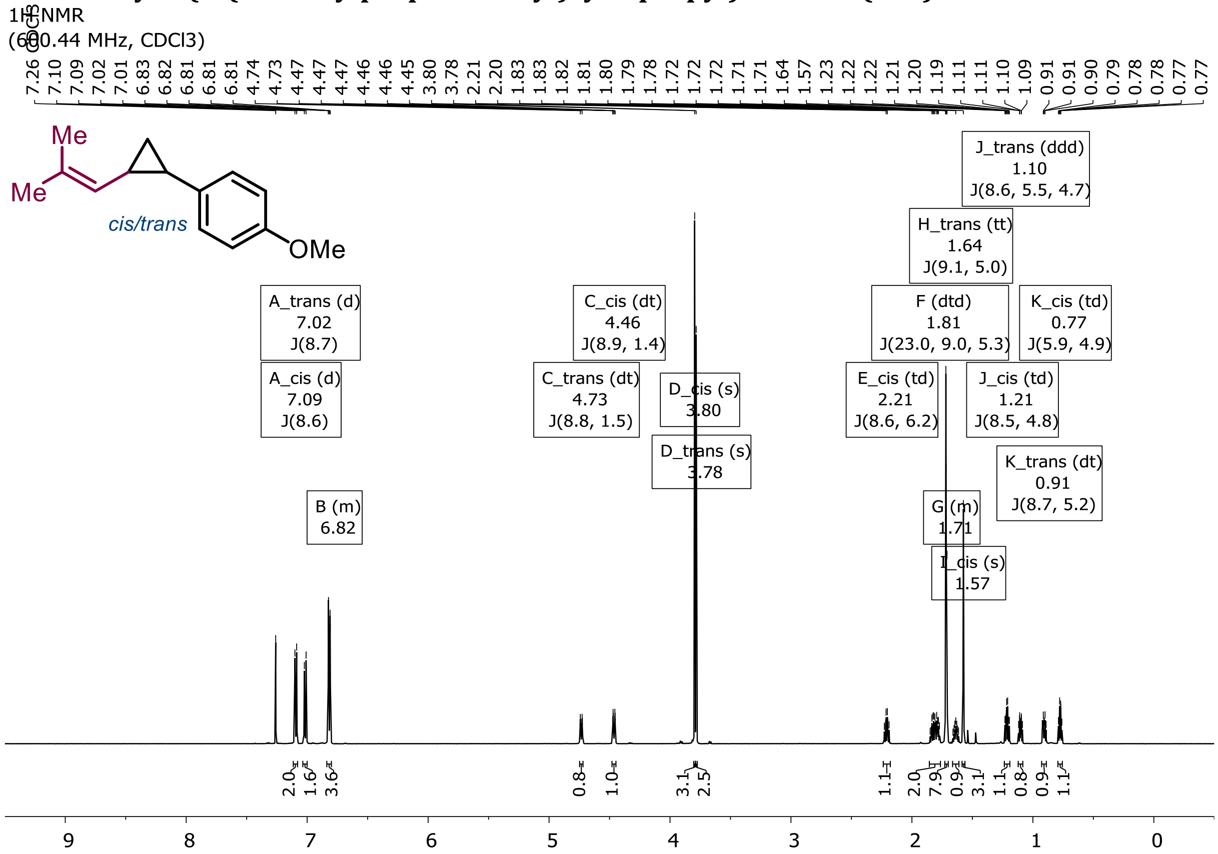

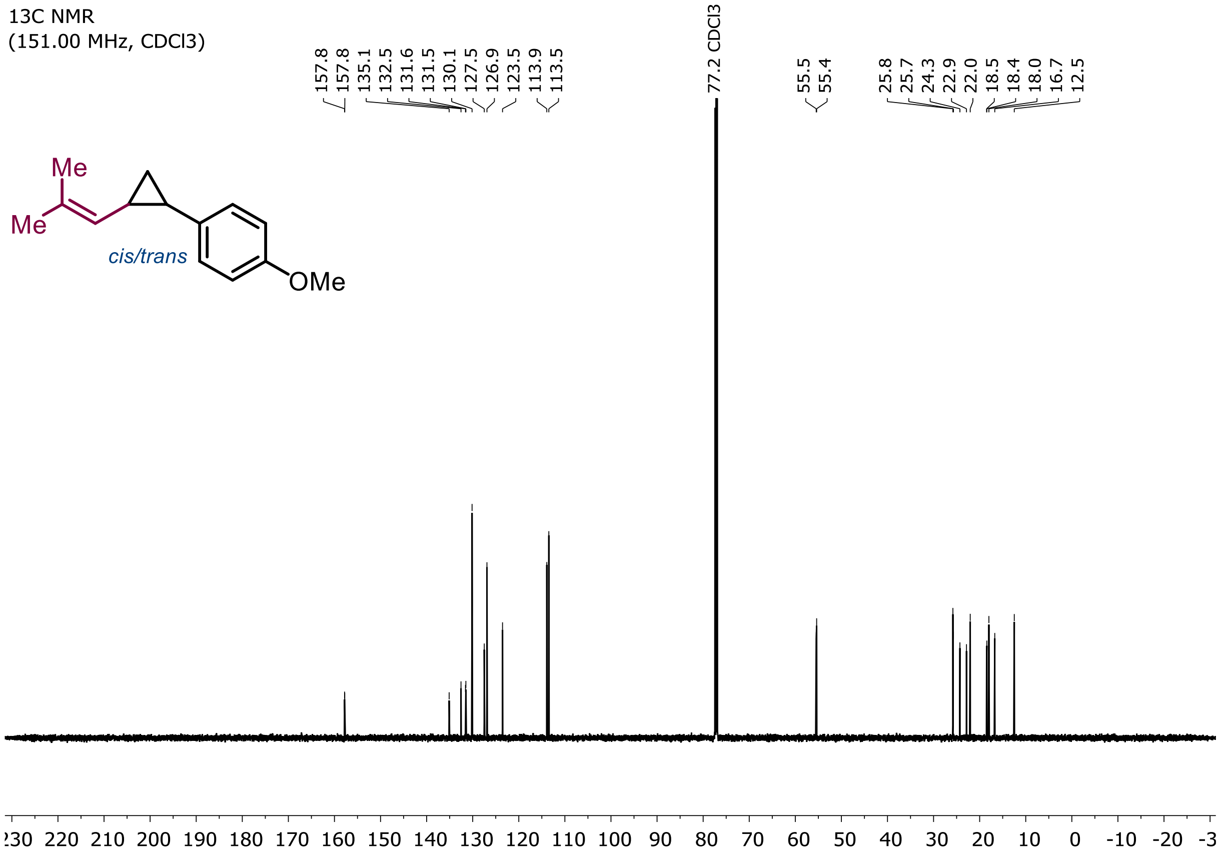

trans-1-methoxy-4-(2-(2-methylprop-1-en-1-yl)cyclopropyl)benzene (34)

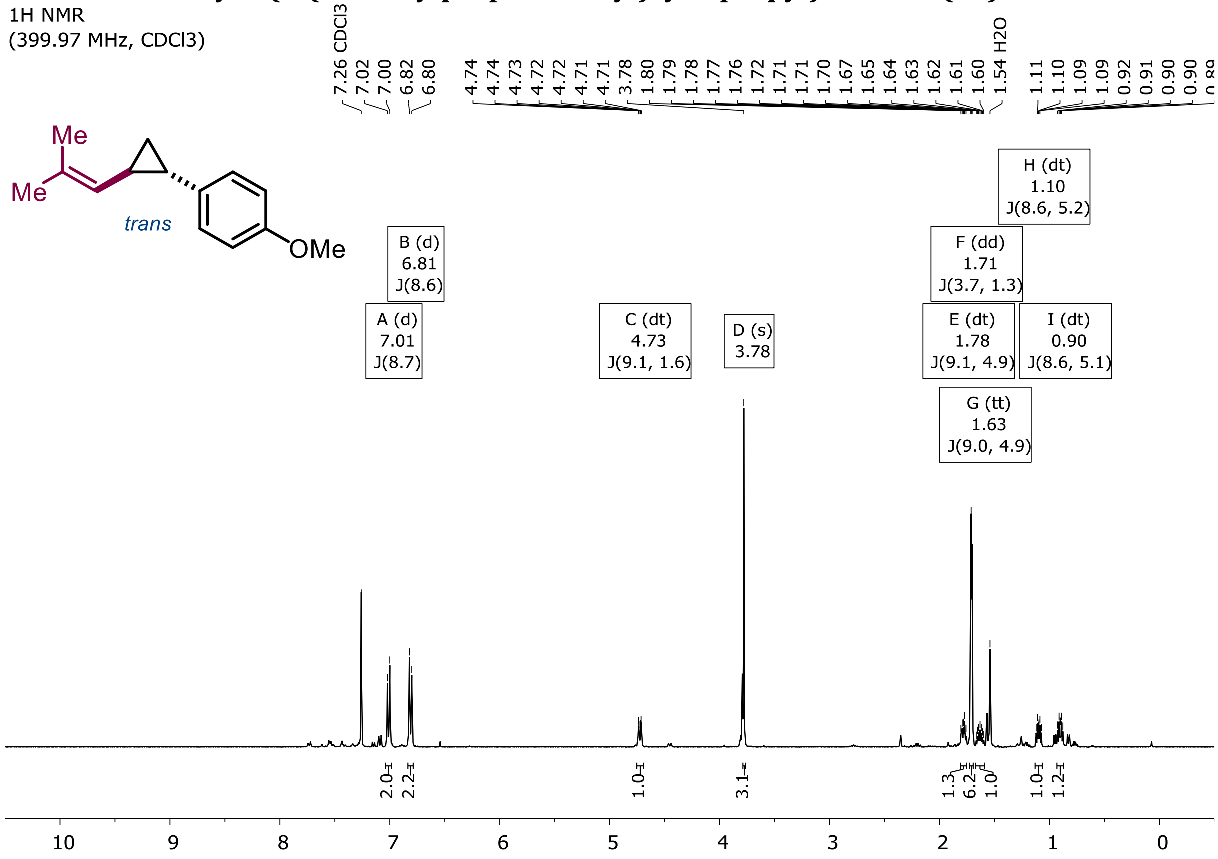

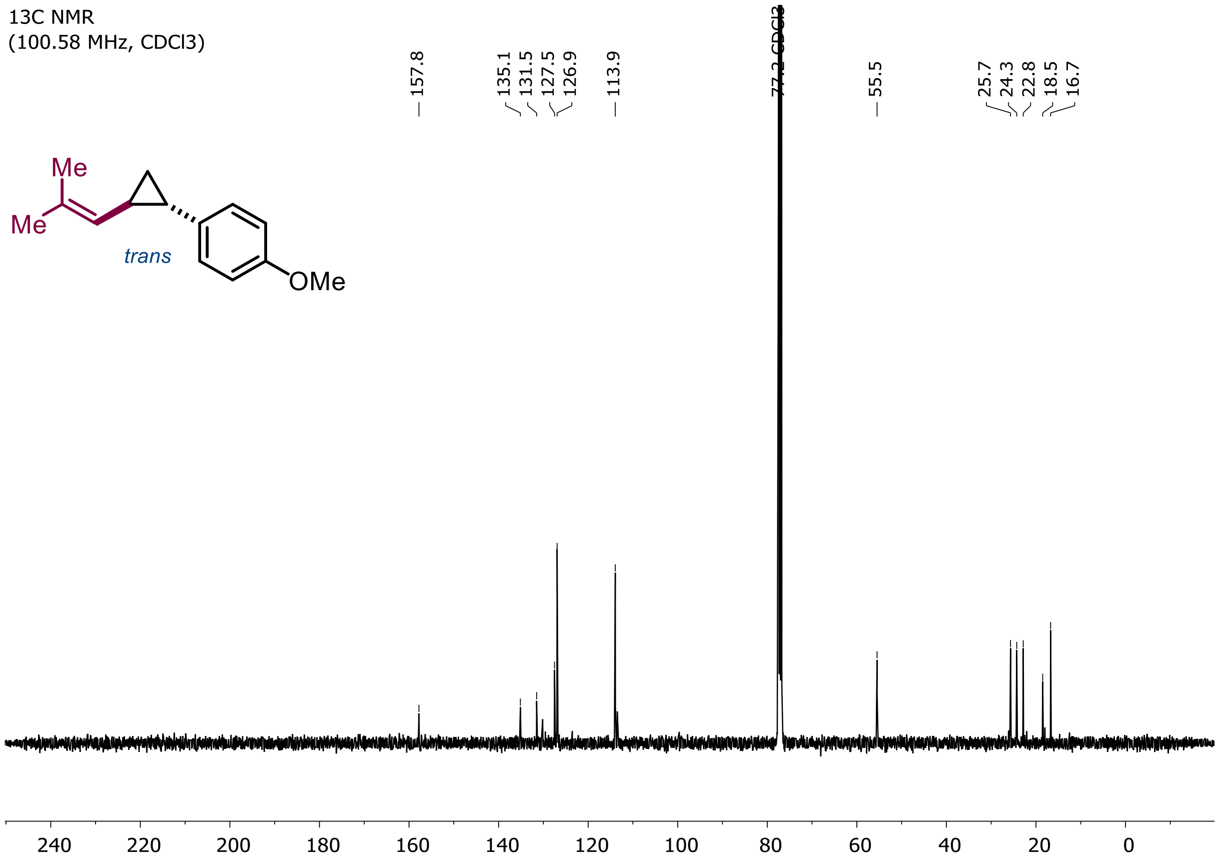

1-fluoro-4-(2-(1-phenylvinyl)cyclopropyl)benzene (S35)

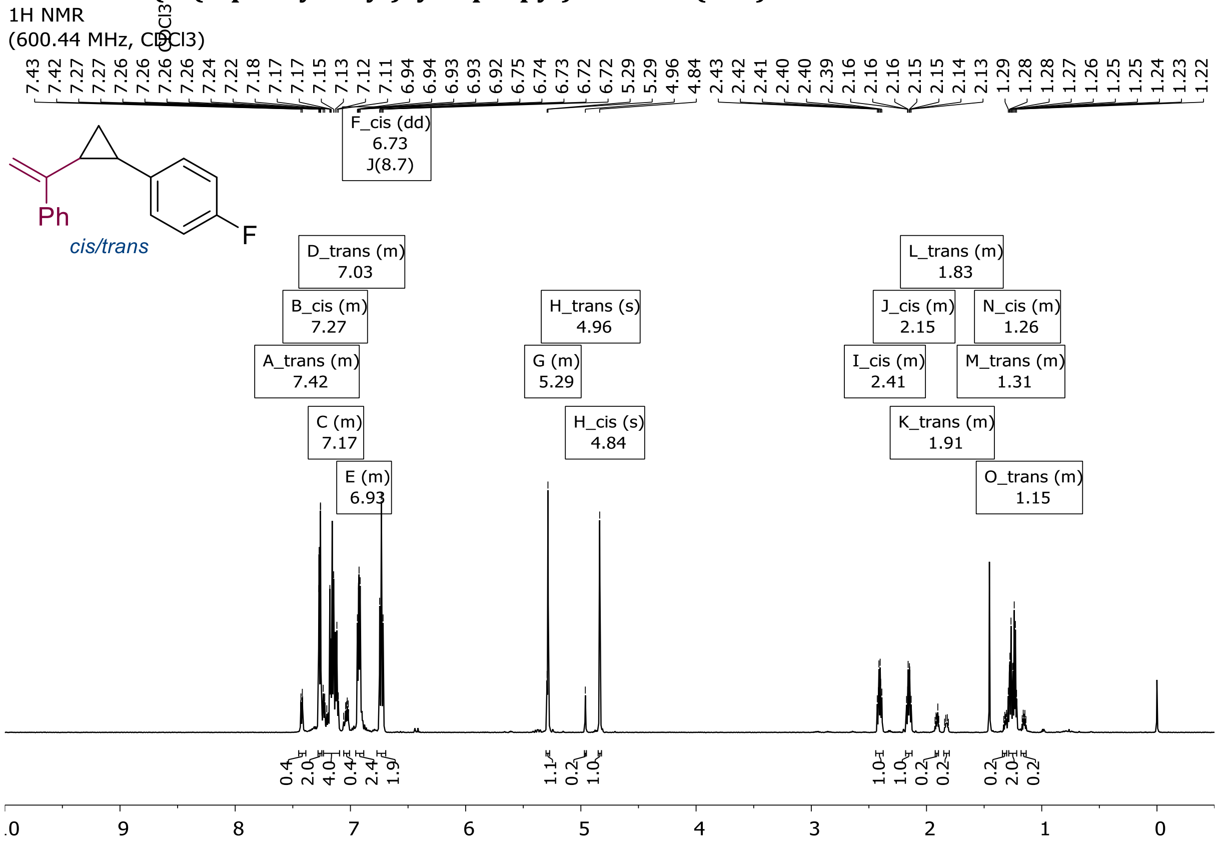

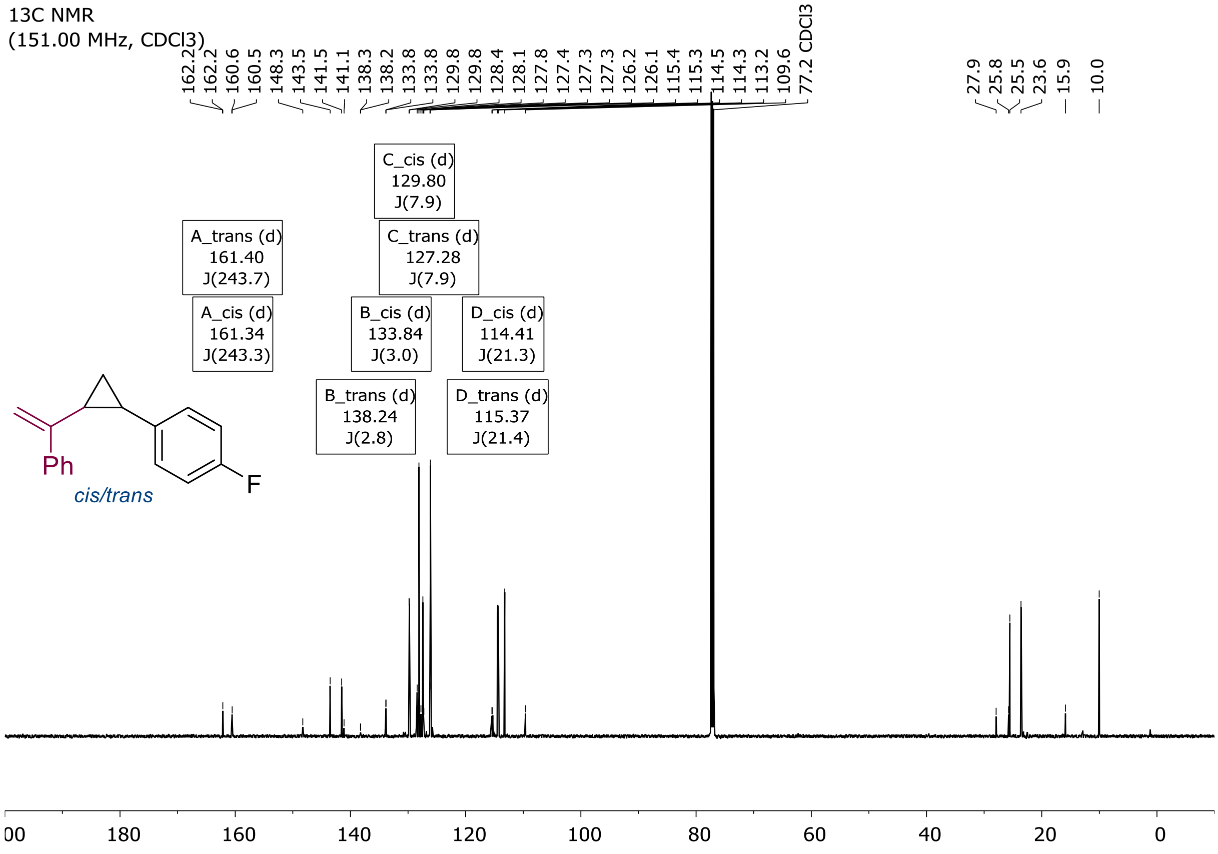

19F NMR
(564.92 MHz, CDCl3)

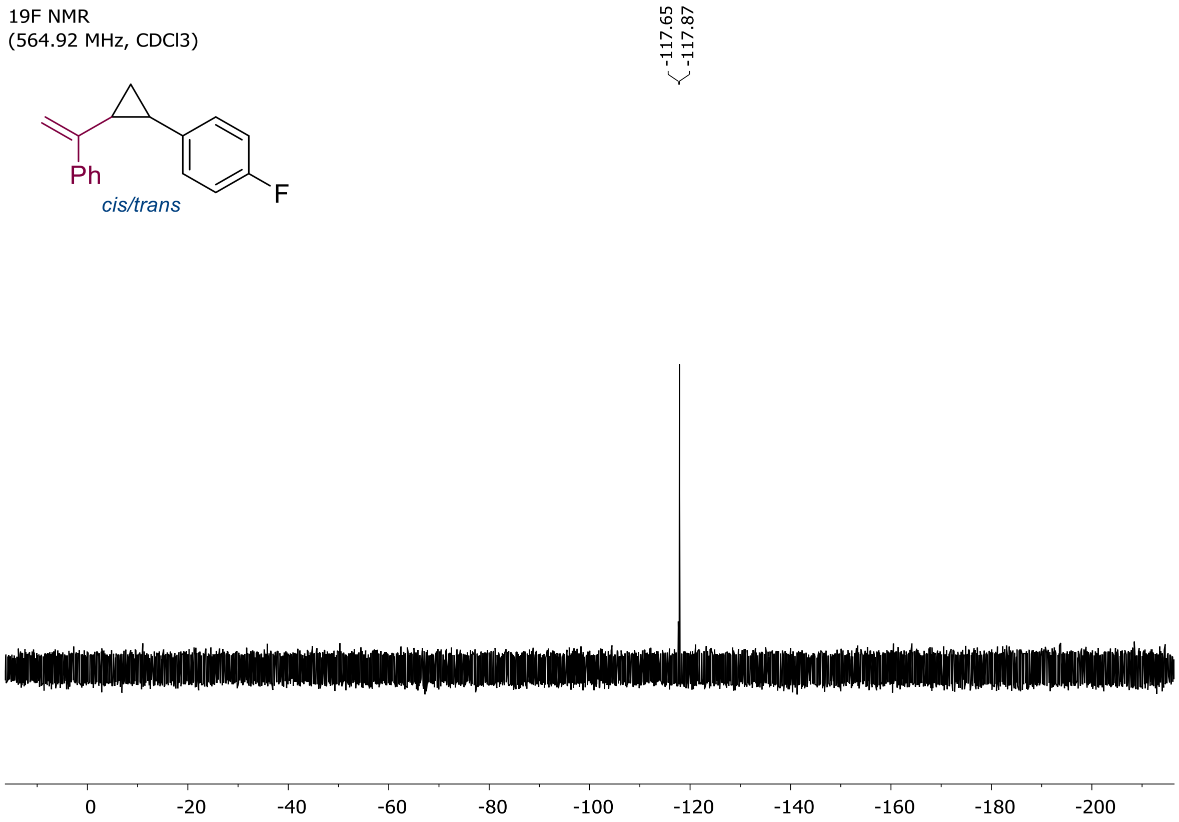

trans-1-fluoro-4-(2-(1-phenylvinyl)cyclopropyl)benzene (35)

1H NMR
(600.44 MHz, CDCl3)

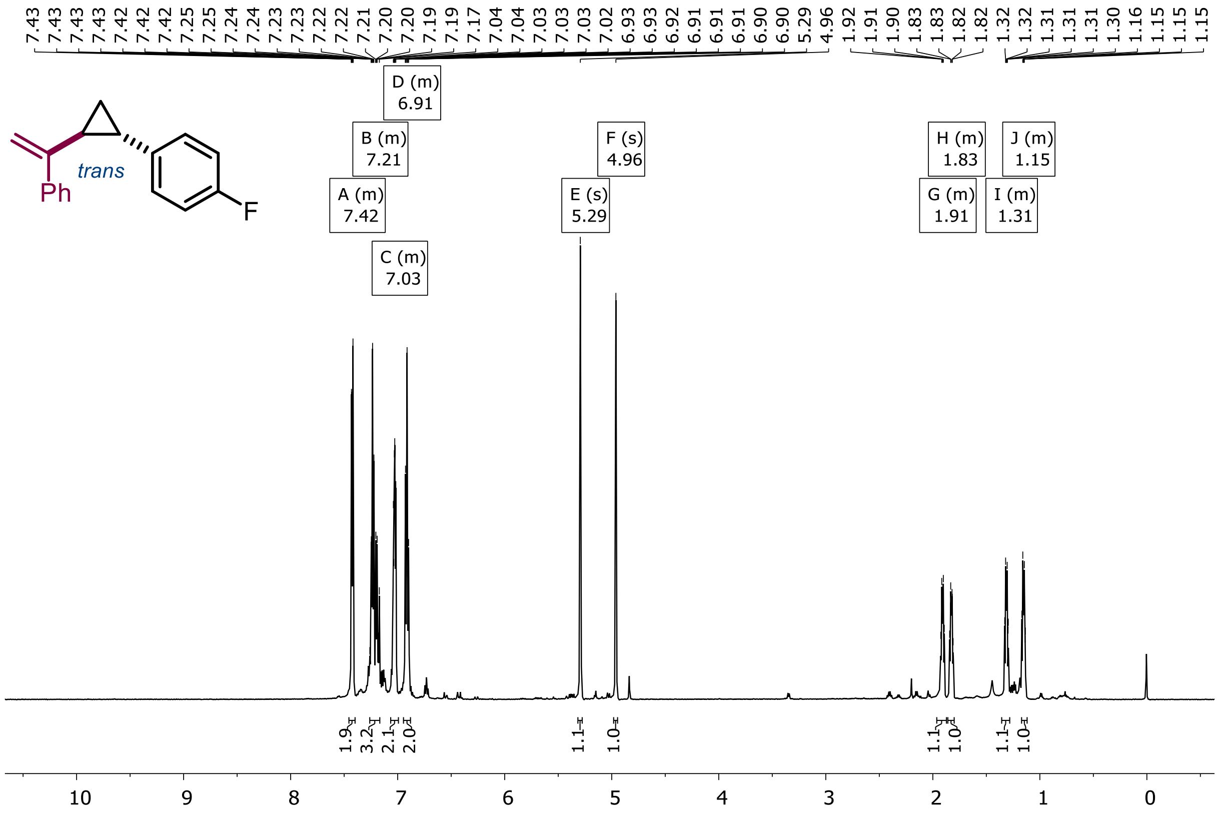

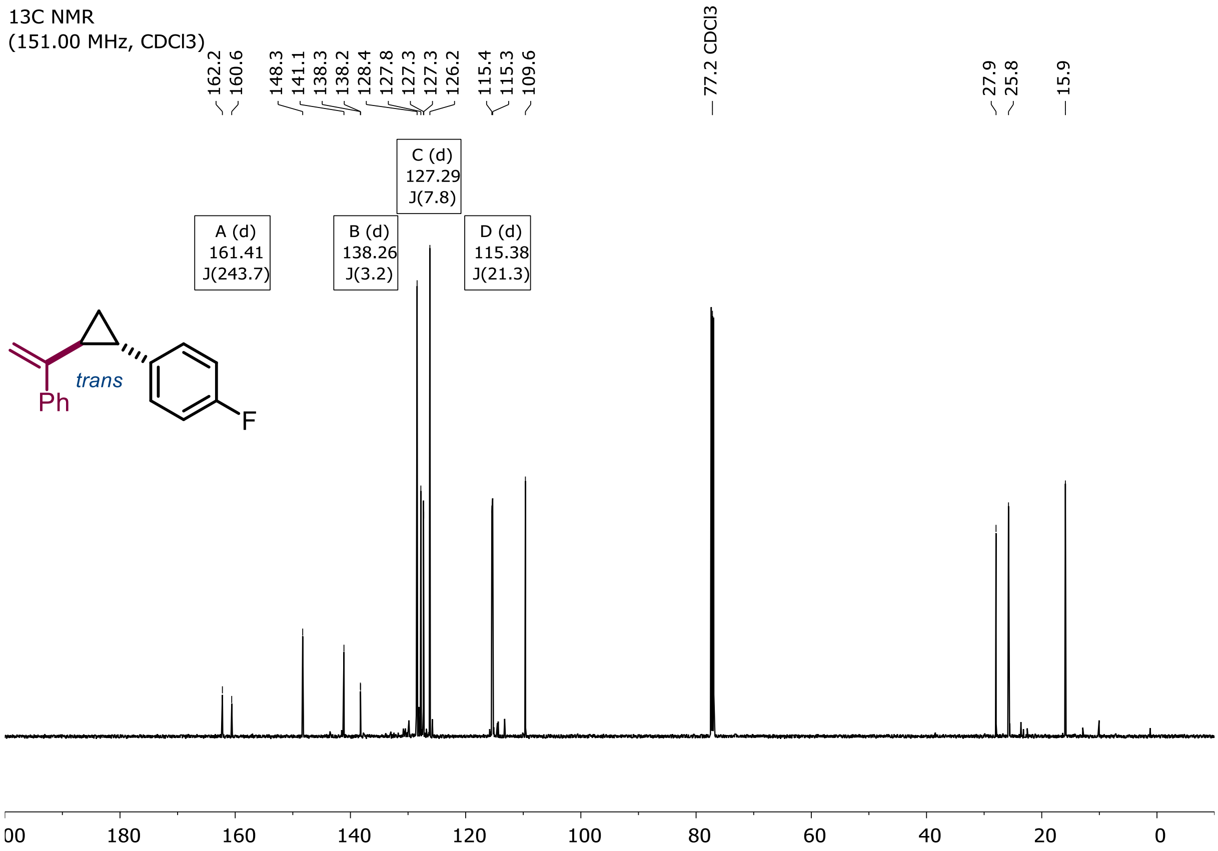
13C NMR
(151.00 MHz, CDCl3)
162.2
160.6
148.3
141.1
138.3
138.2
128.4
127.8
127.3
127.3
126.2
115.4
115.3
109.6
77.2 CDCl3
27.9
25.8
15.9
C (d)
127.29
J(7.8)
A (d)
161.41
J(243.7)
B (d)
138.26
J(3.2)
D (d)
115.38
J(21.3)
trans
Ph
F

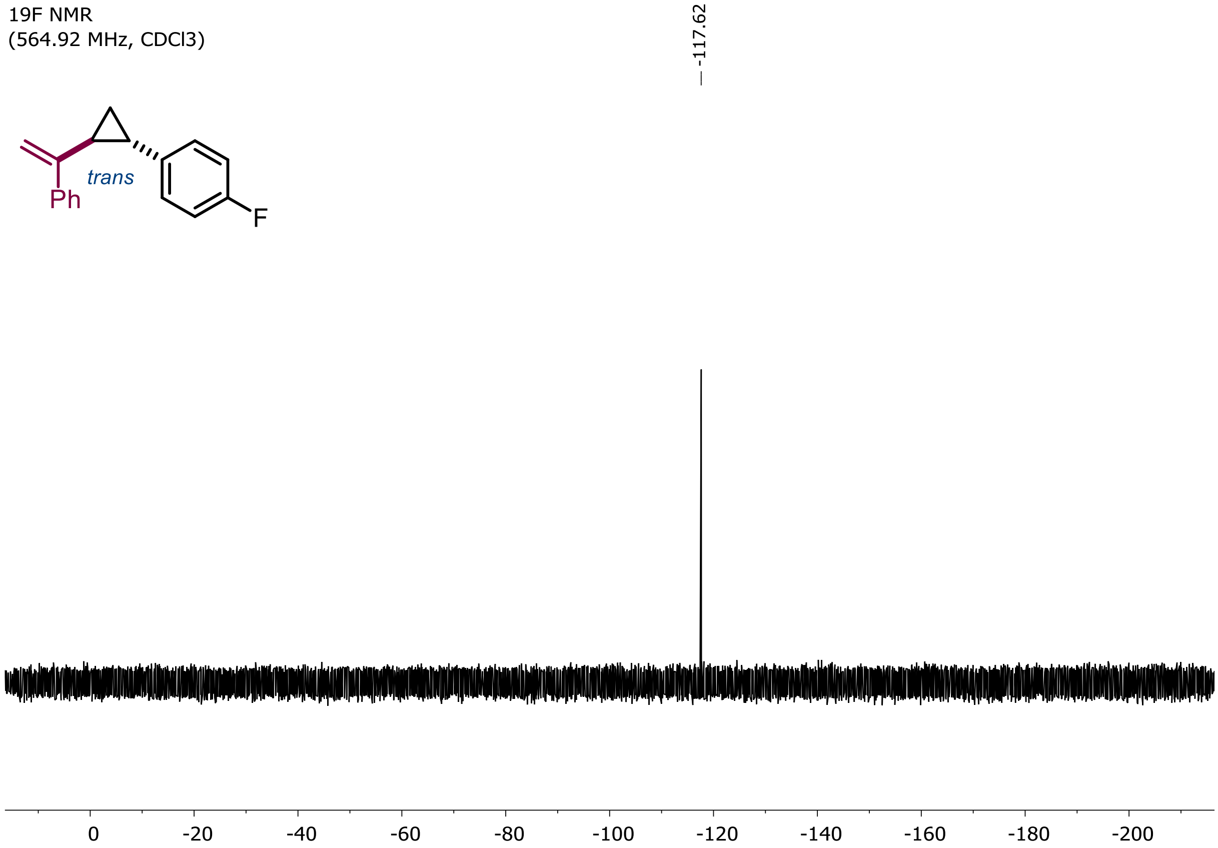
19F NMR
(564.92 MHz, CDCl3)
-117.62
trans
Ph
F

1-fluoro-4-(2-(prop-1-en-2-yl)cyclopropyl)benzene (S36)

1H NMR
(600.44 MHz, CDCl3)

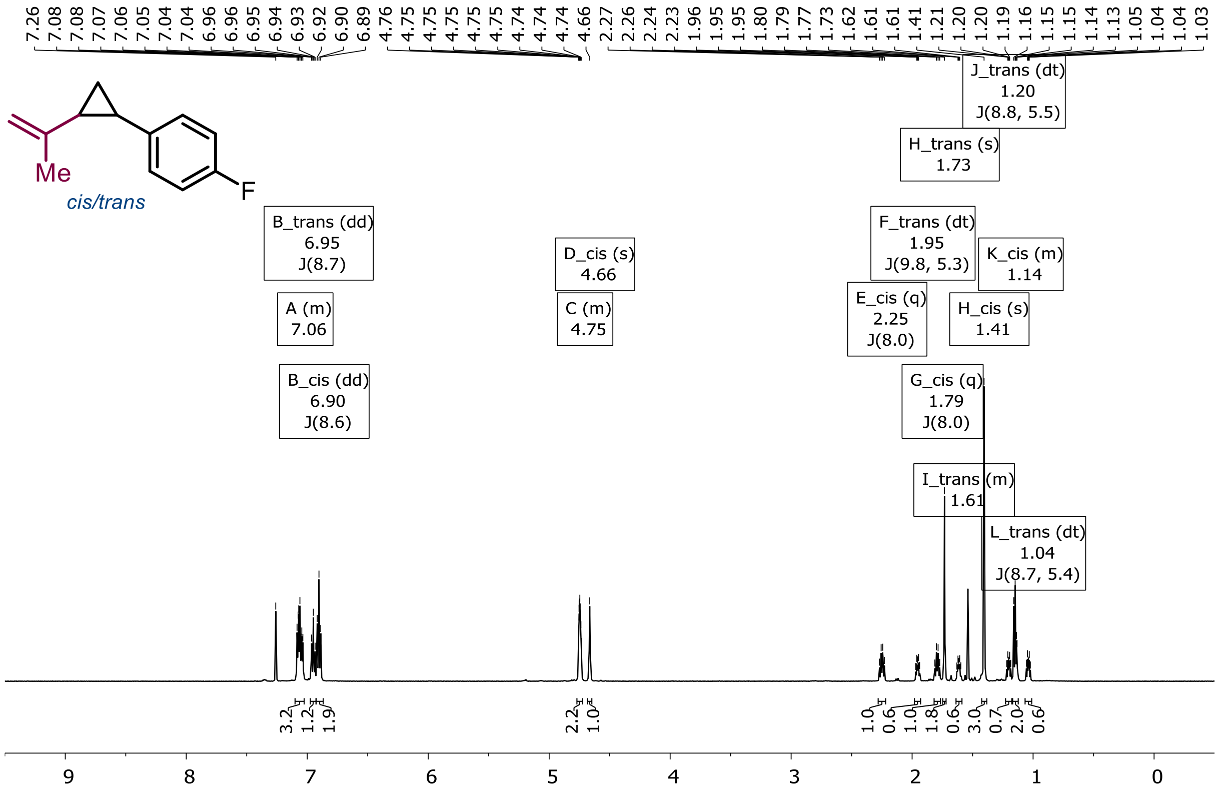

13C NMR
(151.00 MHz, CDCl3)

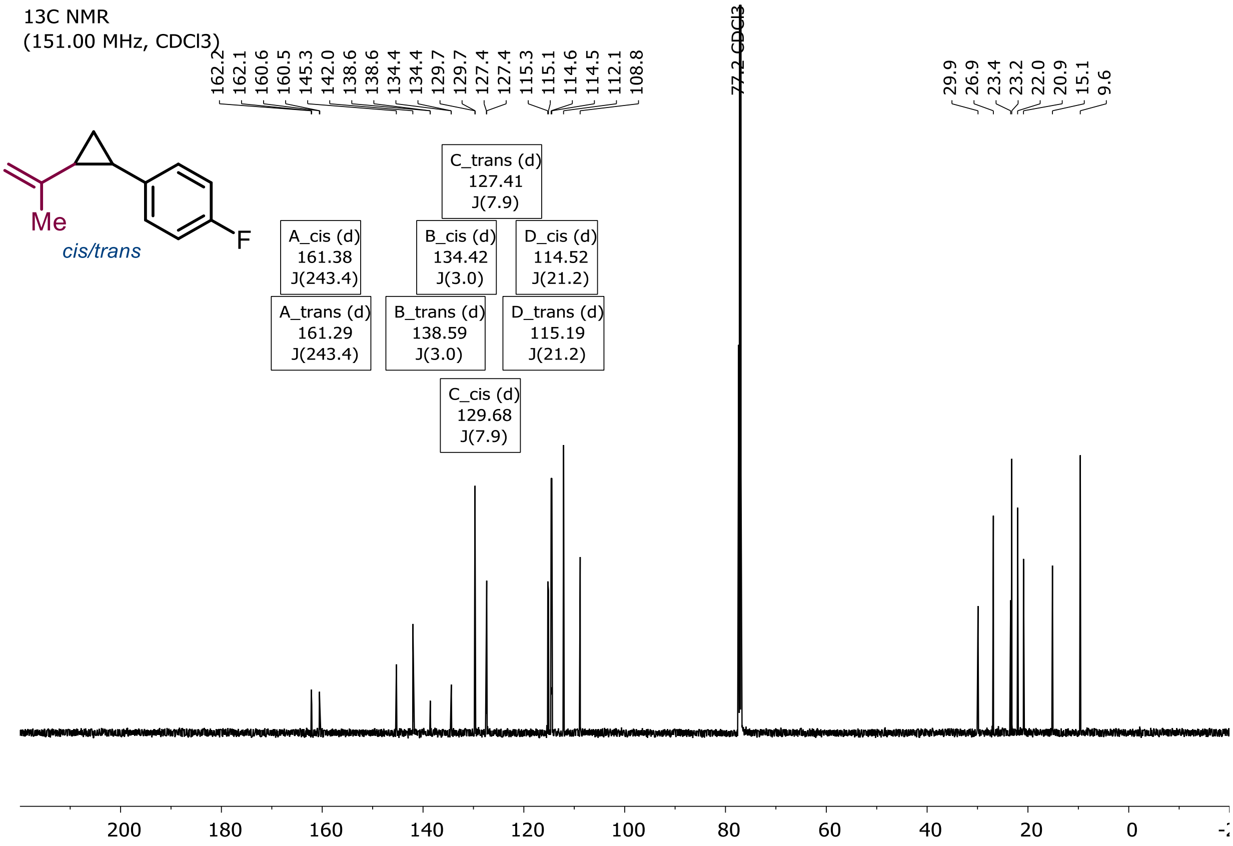

19F NMR
(564.92 MHz, CDCl3)

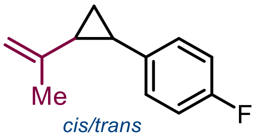

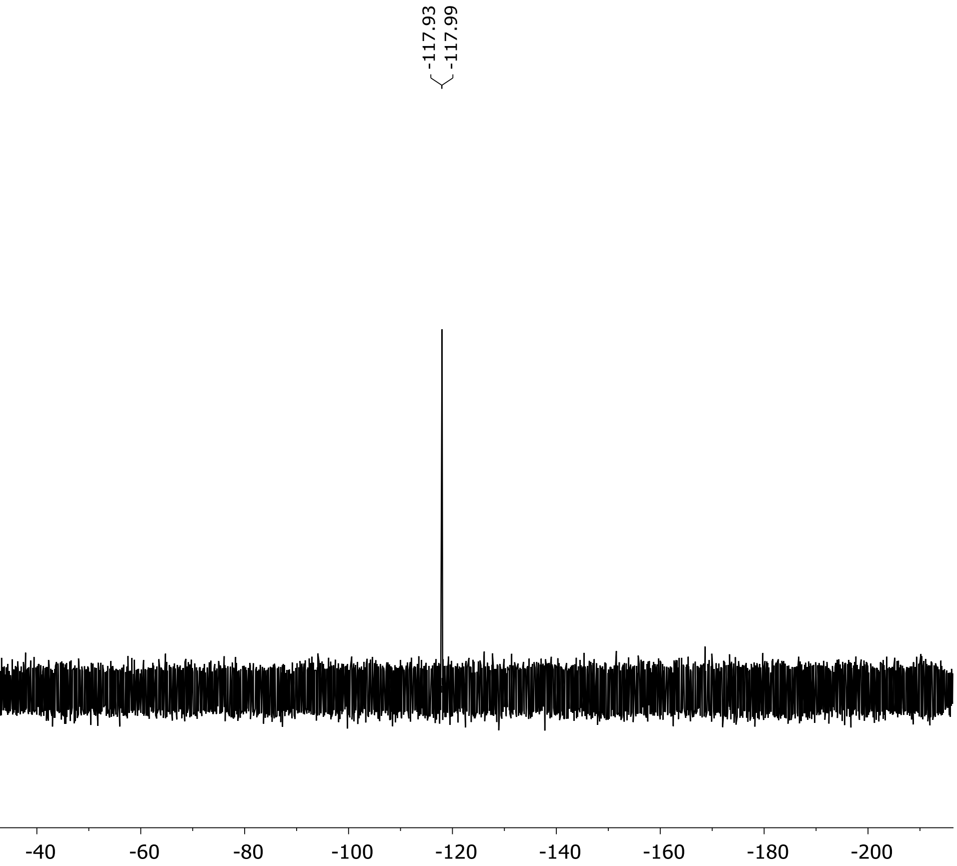

trans-1-fluoro-4-(2-(prop-1-en-2-yl)cyclopropyl)benzene (36)

1H NMR
(600.44 MHz, CDCl3)

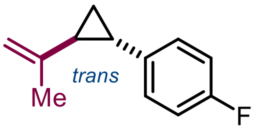

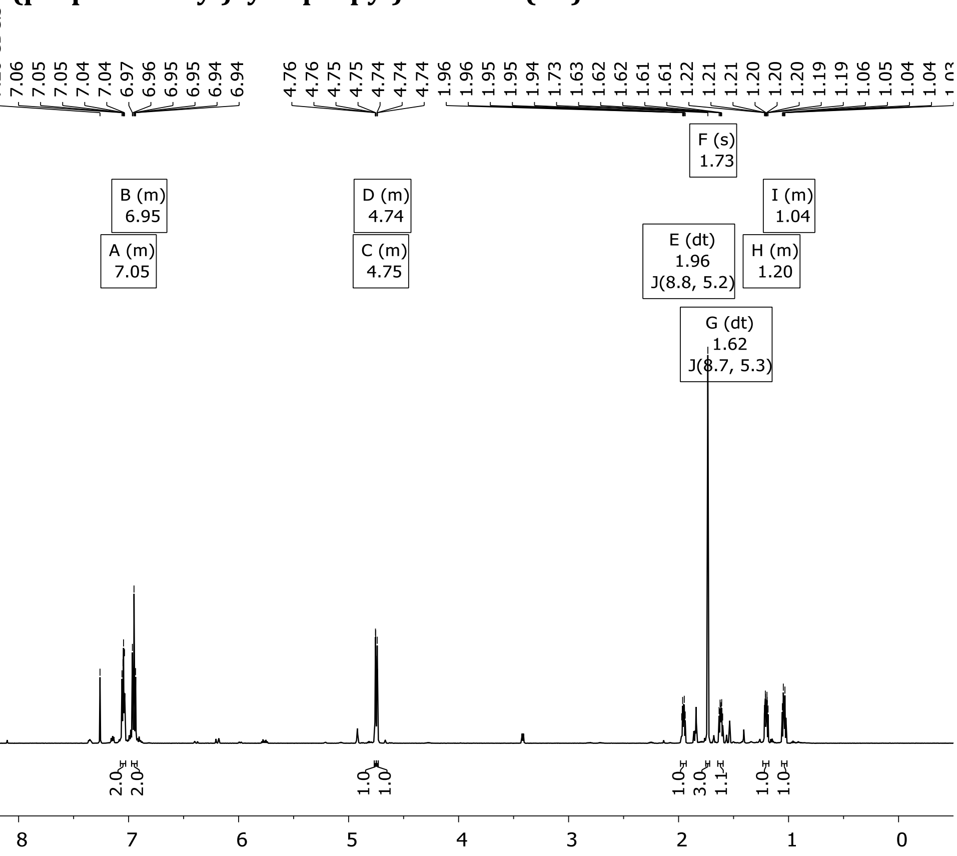

13C NMR
(151.00 MHz, CDCl3)

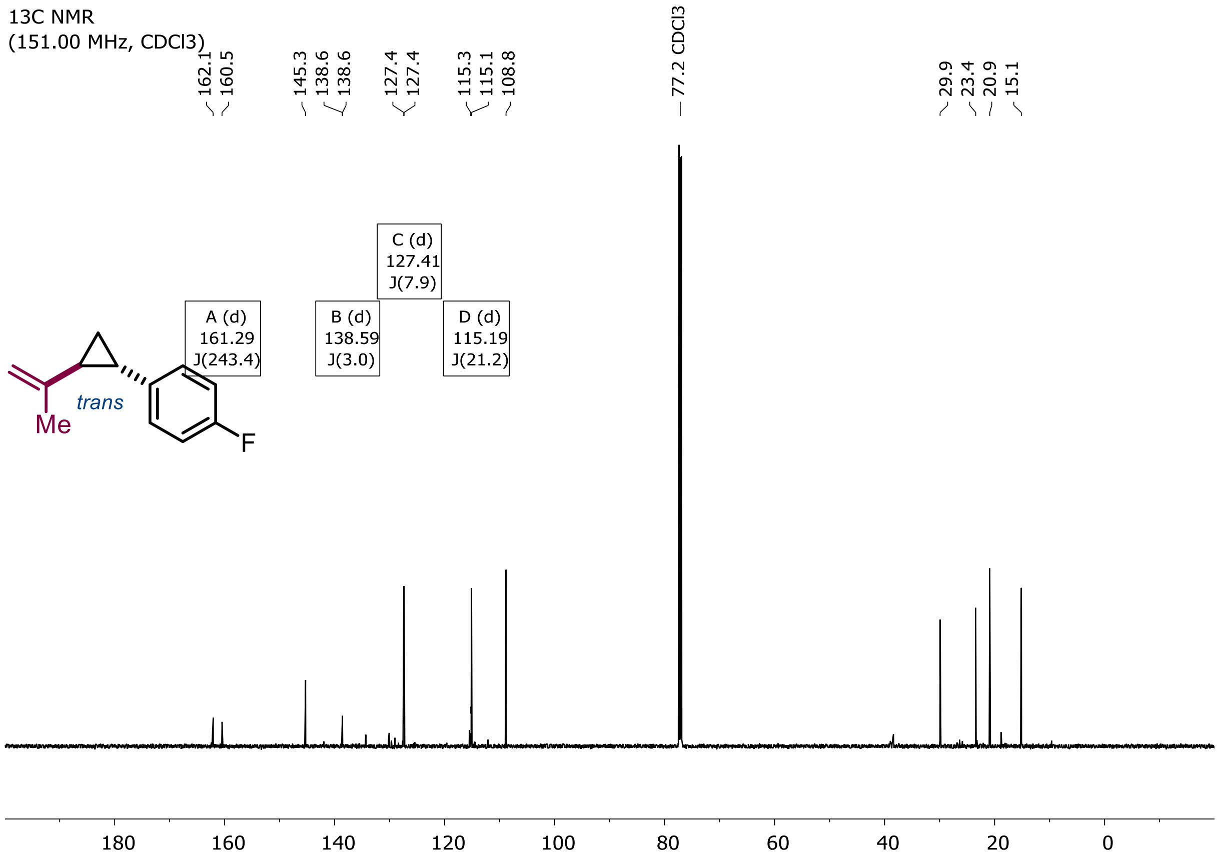

19F NMR
(564.92 MHz, CDCl3)

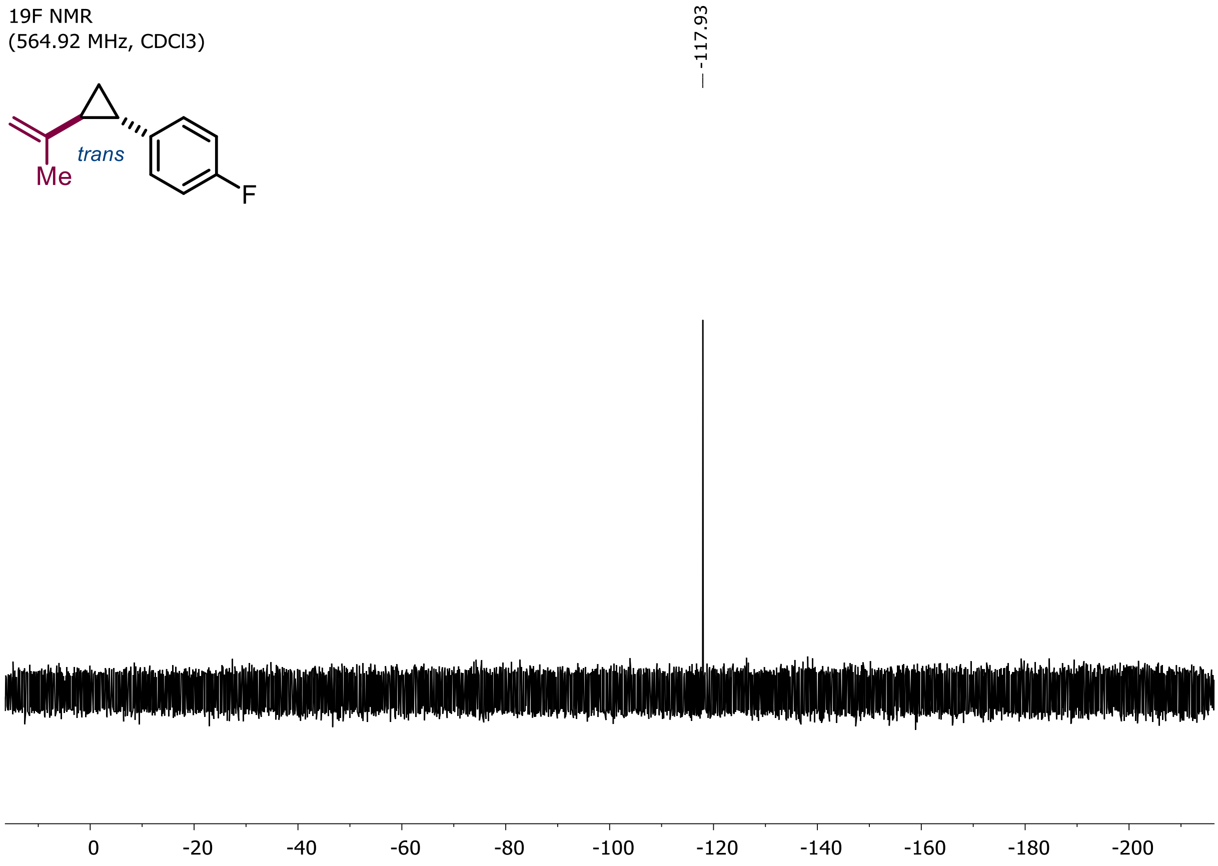

tert-butyl (1*R*,2*S*)-1-((*tert*-butoxycarbonyl)amino)-2-vinylcyclopropane-1-carboxylate (37)

1H NMR
(600.44 MHz, CDCl3)

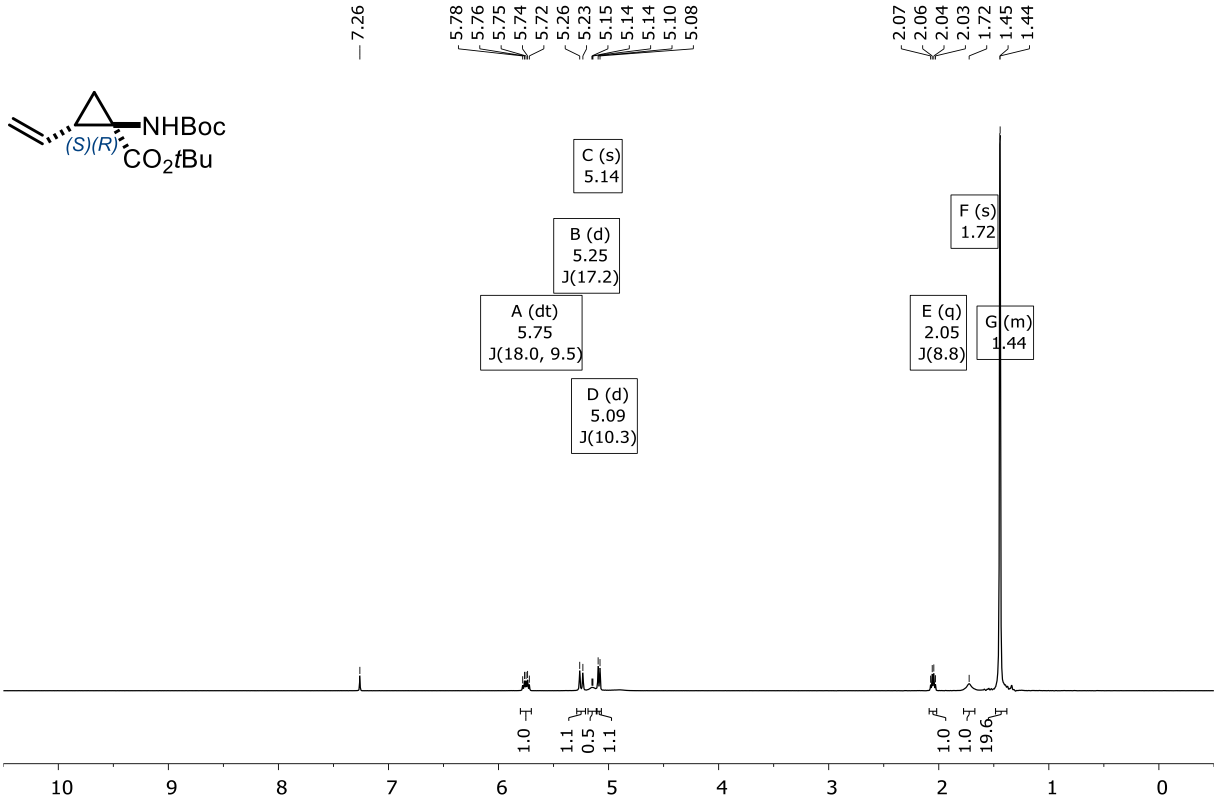

13C NMR
(151.00 MHz, CDCl3)

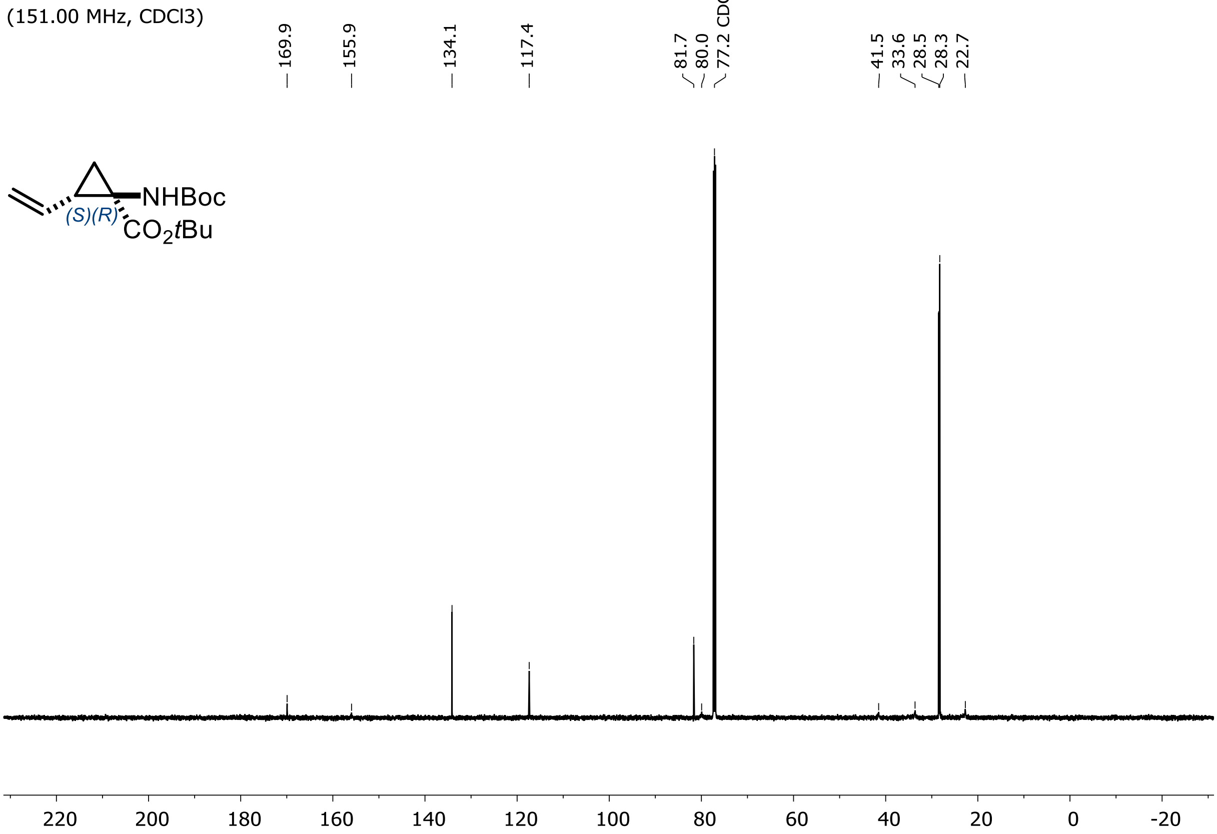

tert-butyl (1*S*,2*S*)-1-((*tert*-butoxycarbonyl)amino)-2-vinylcyclopropane-1-carboxylate (38)

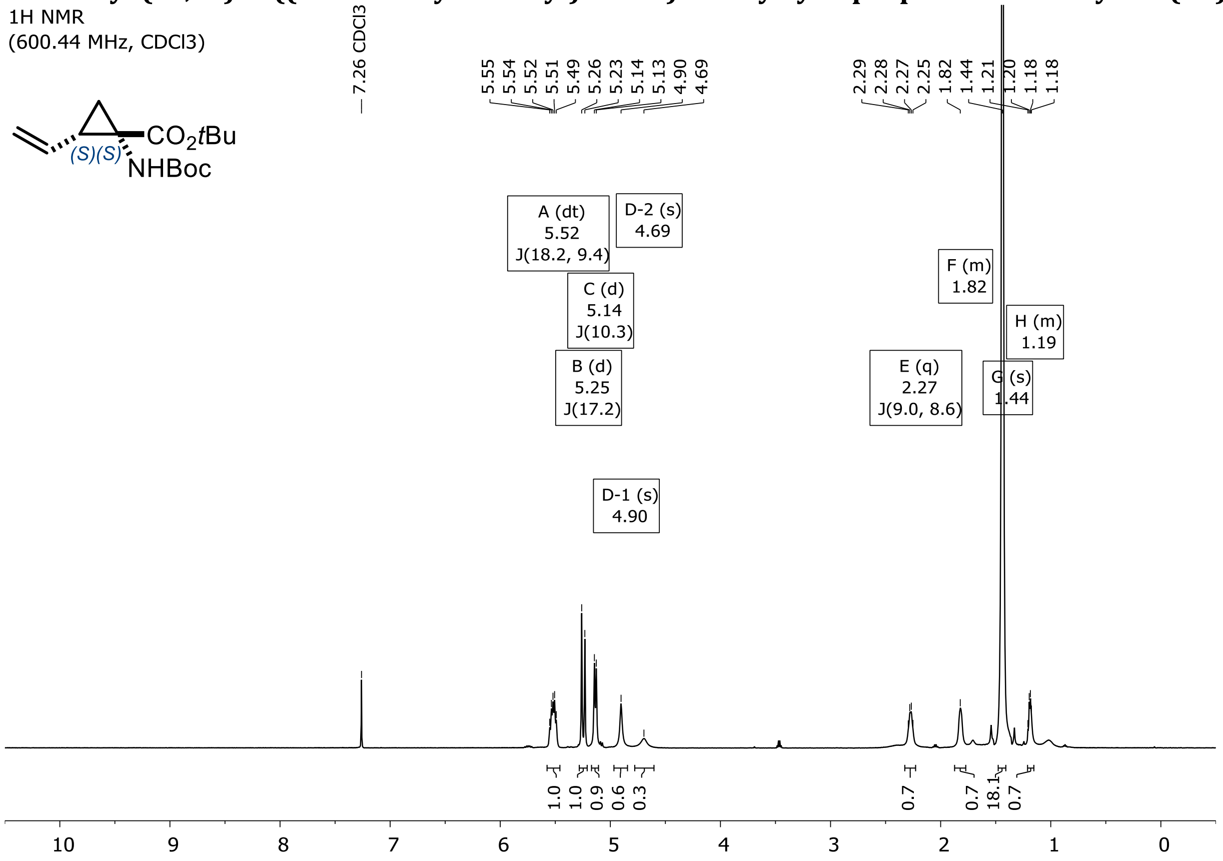

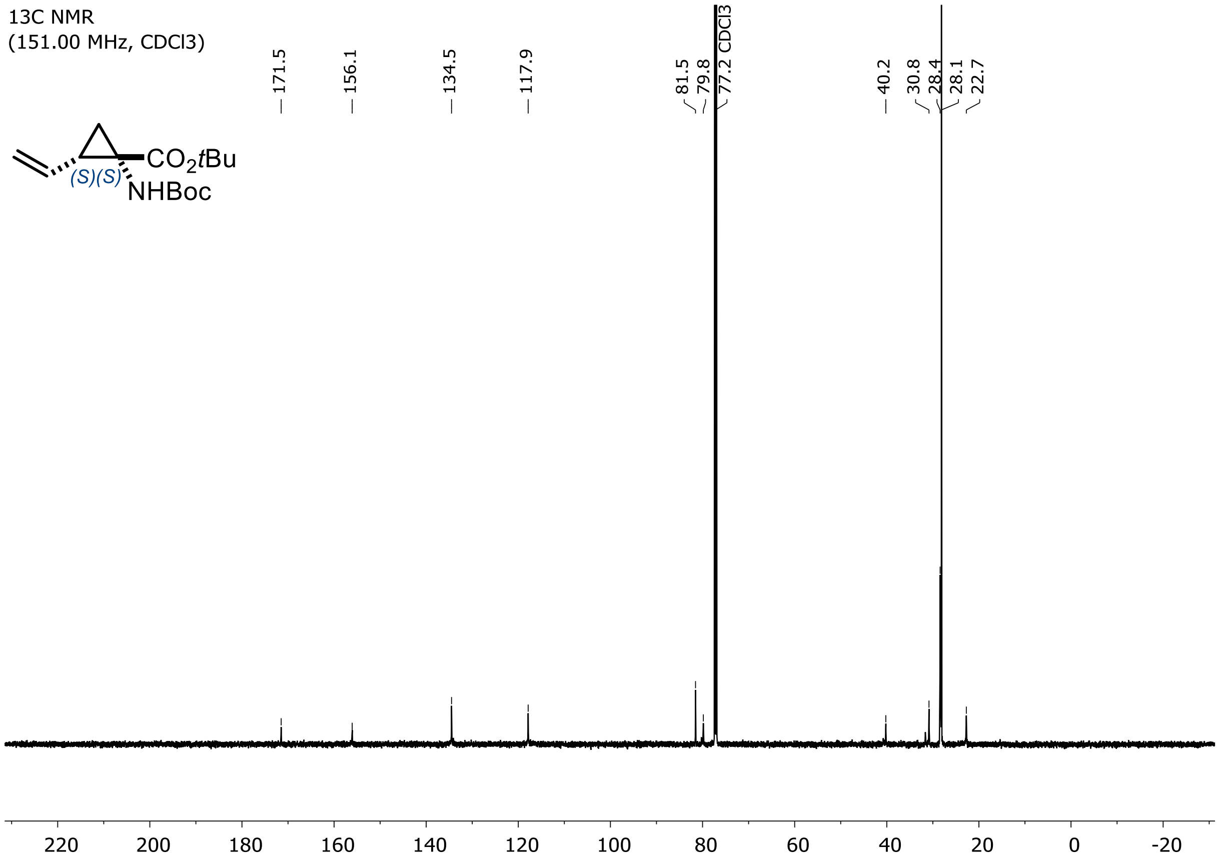

14.2. NMR spectra of chiral vinylcyclopropane probe and intermediates

(*cis*)-2-(4-methoxyphenyl)cyclopropyl)methanol (*rac*-cis)

1H NMR
(600.44 MHz, CDCl3)

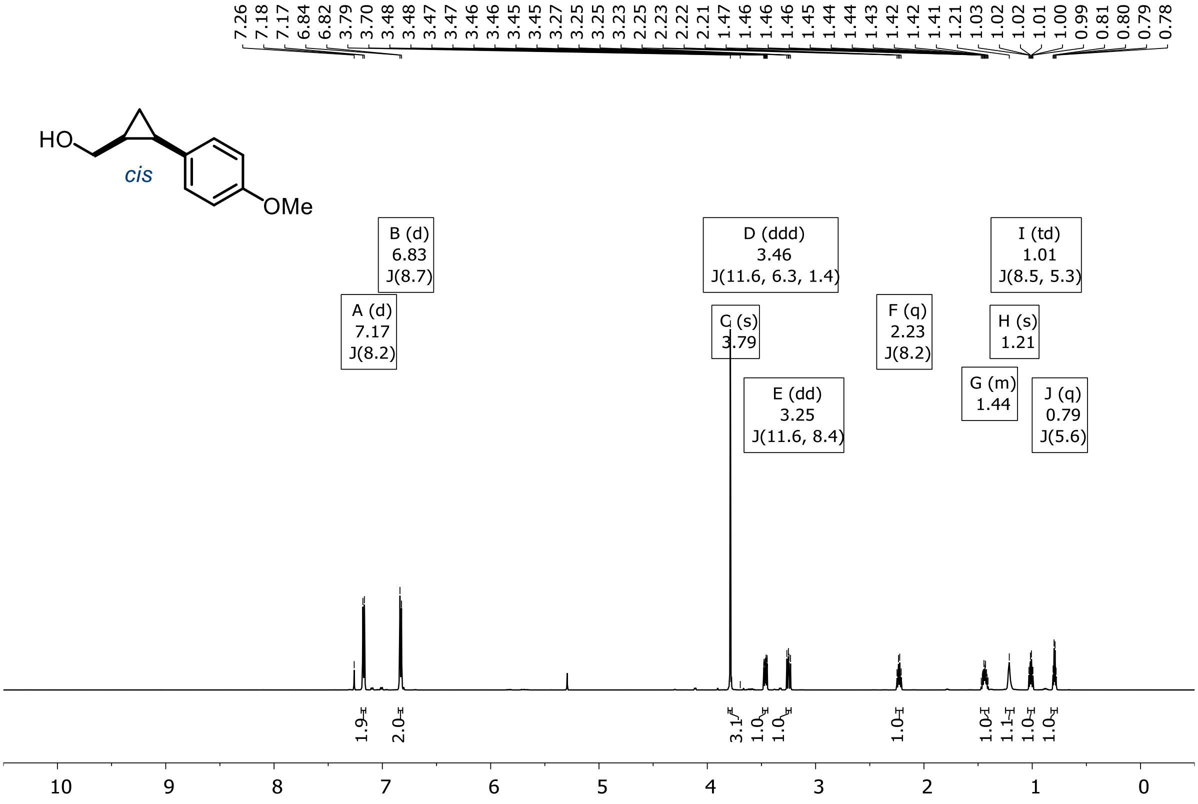

13C NMR
(151.00 MHz, CDCl3)

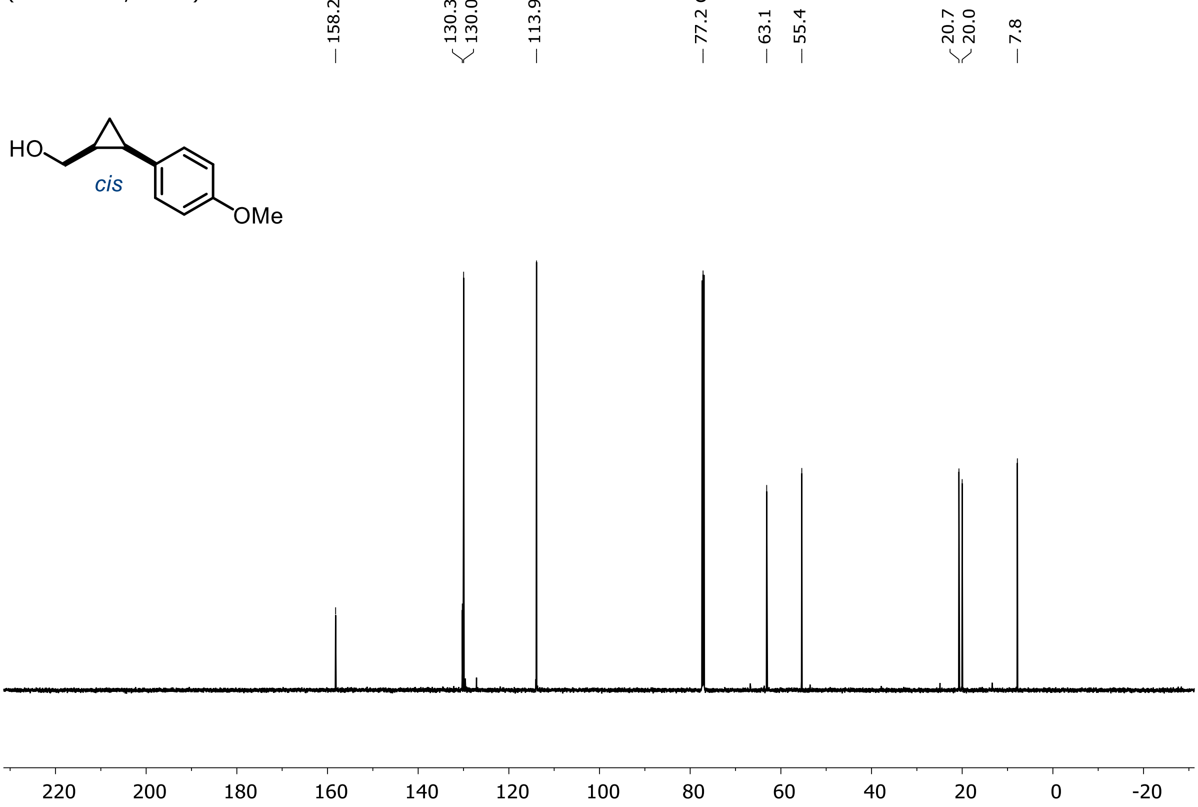

(*trans*)-2-(4-methoxyphenyl)cyclopropyl)methanol (*rac*-trans)

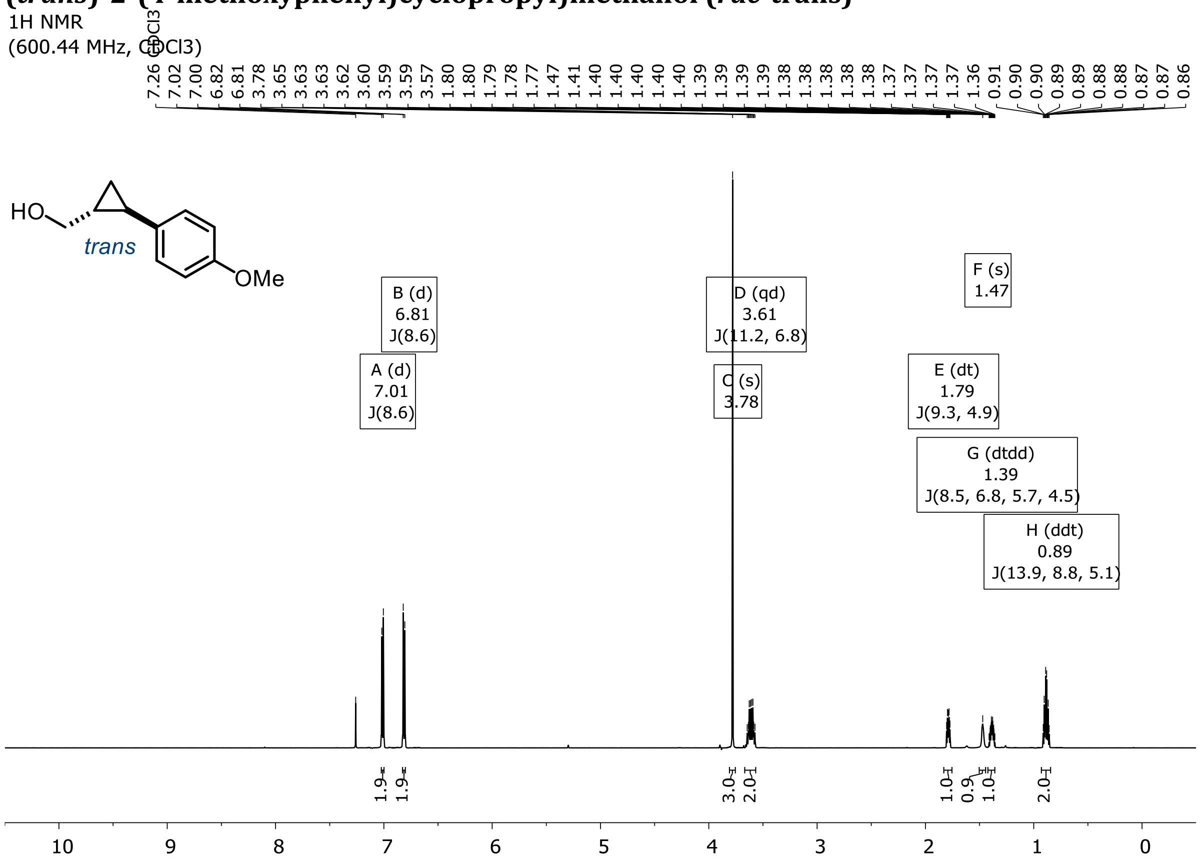

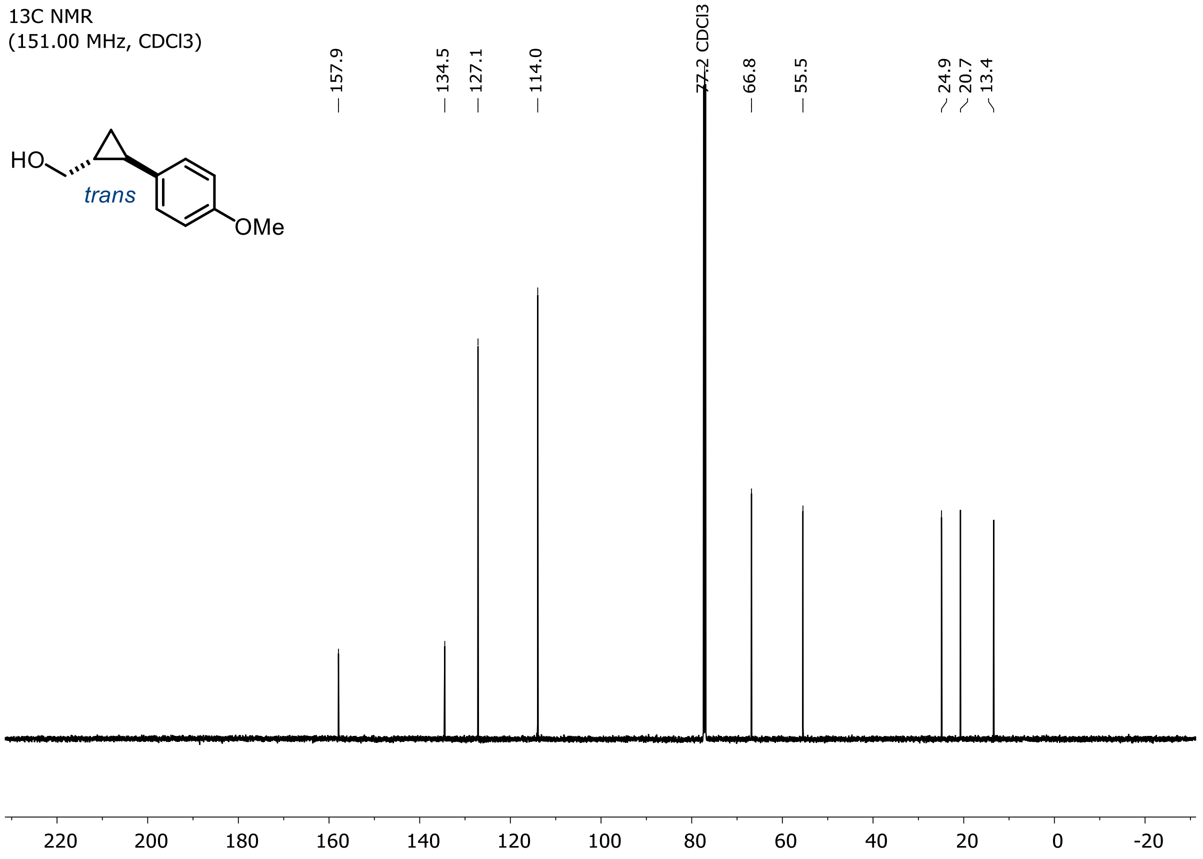

14.3. NMR spectra of divinylcyclopropanes and cyclized products

Dictyopterene A (39)

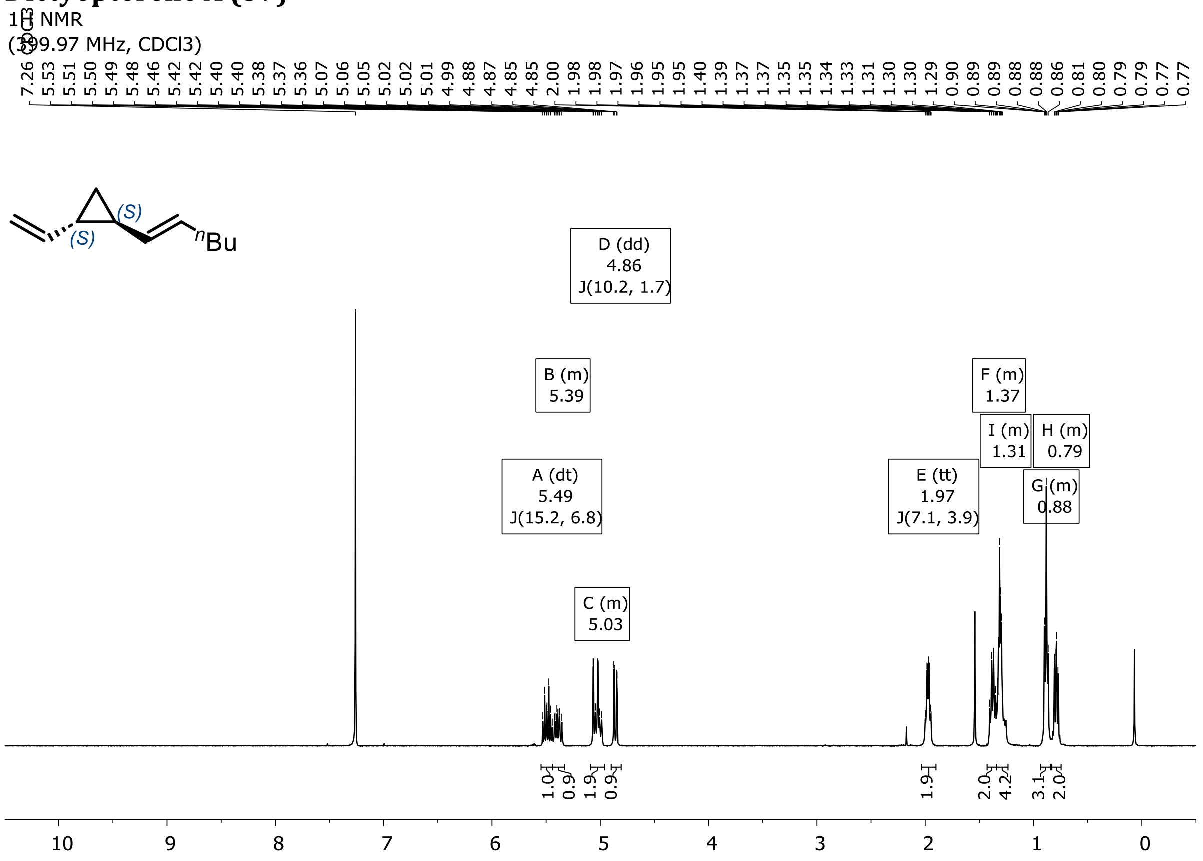

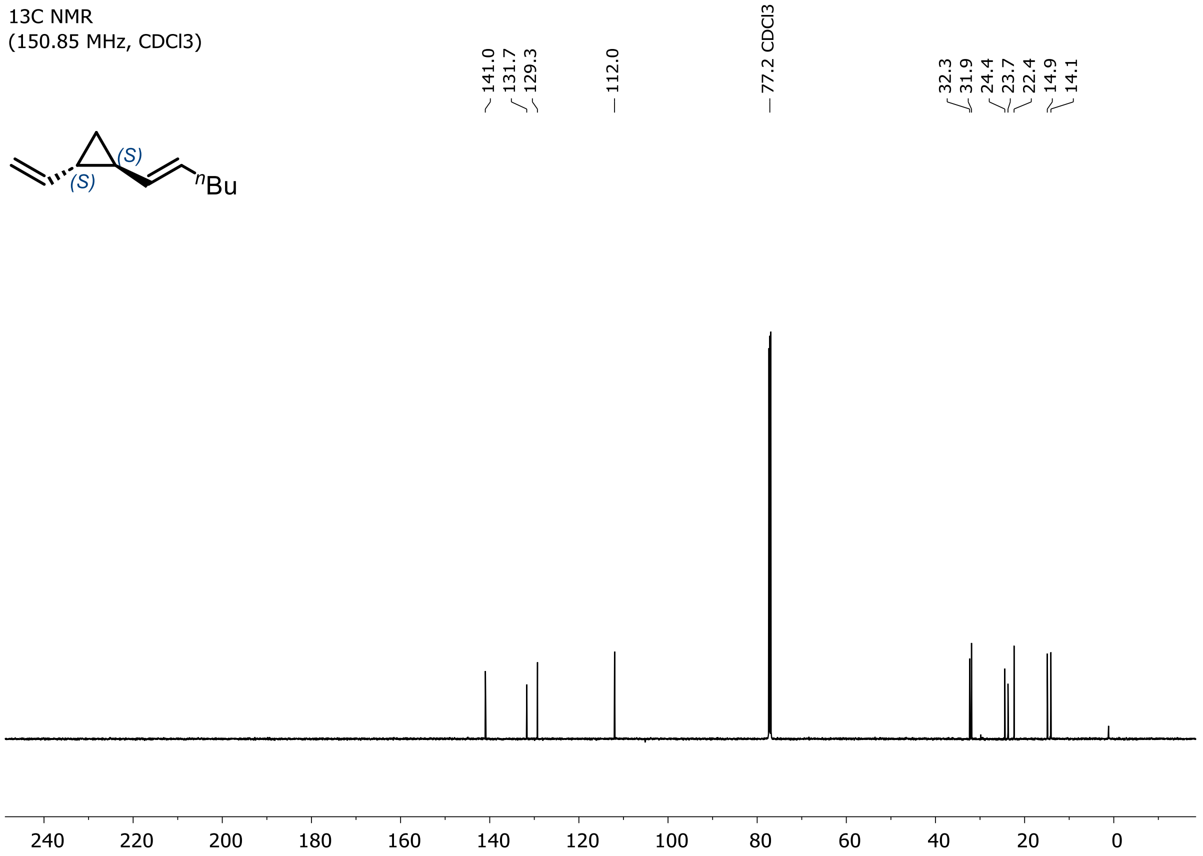

Dictyopterene C' (40)

1H NMR
(400.44 MHz, CDCl3)

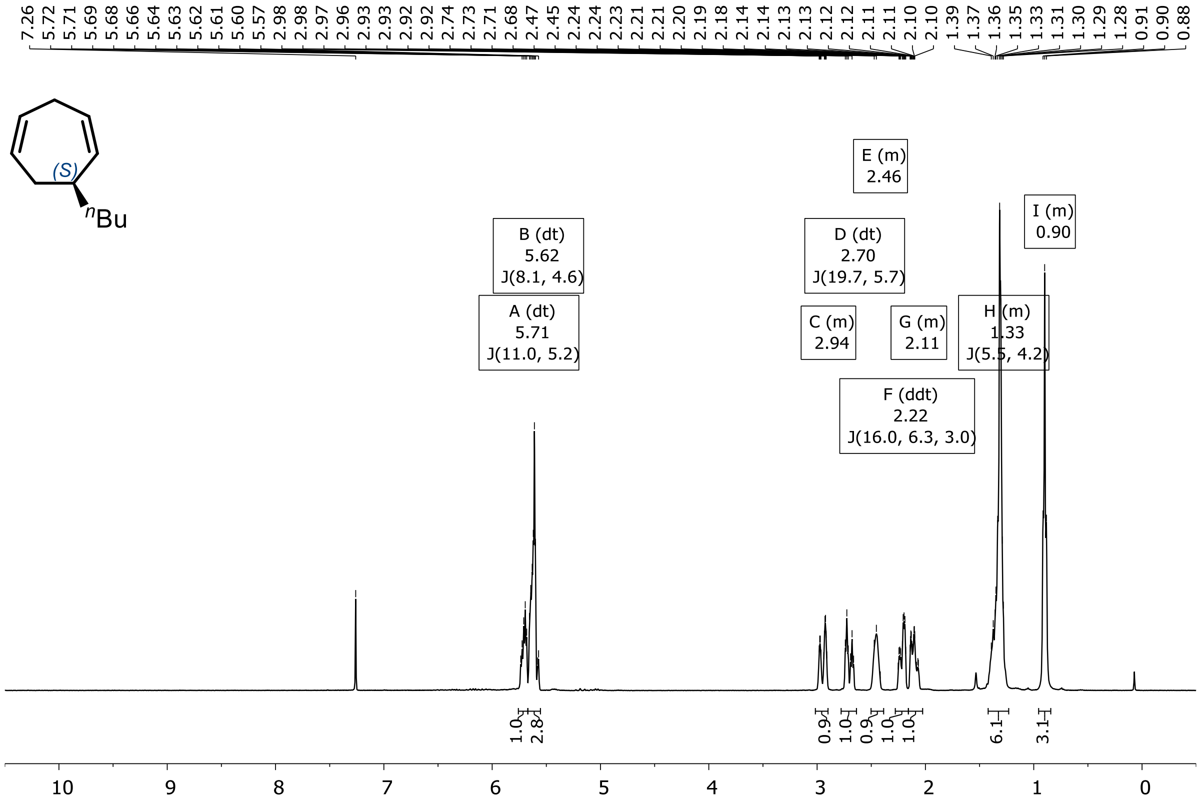

13C NMR
(100.70 MHz, CDCl3)

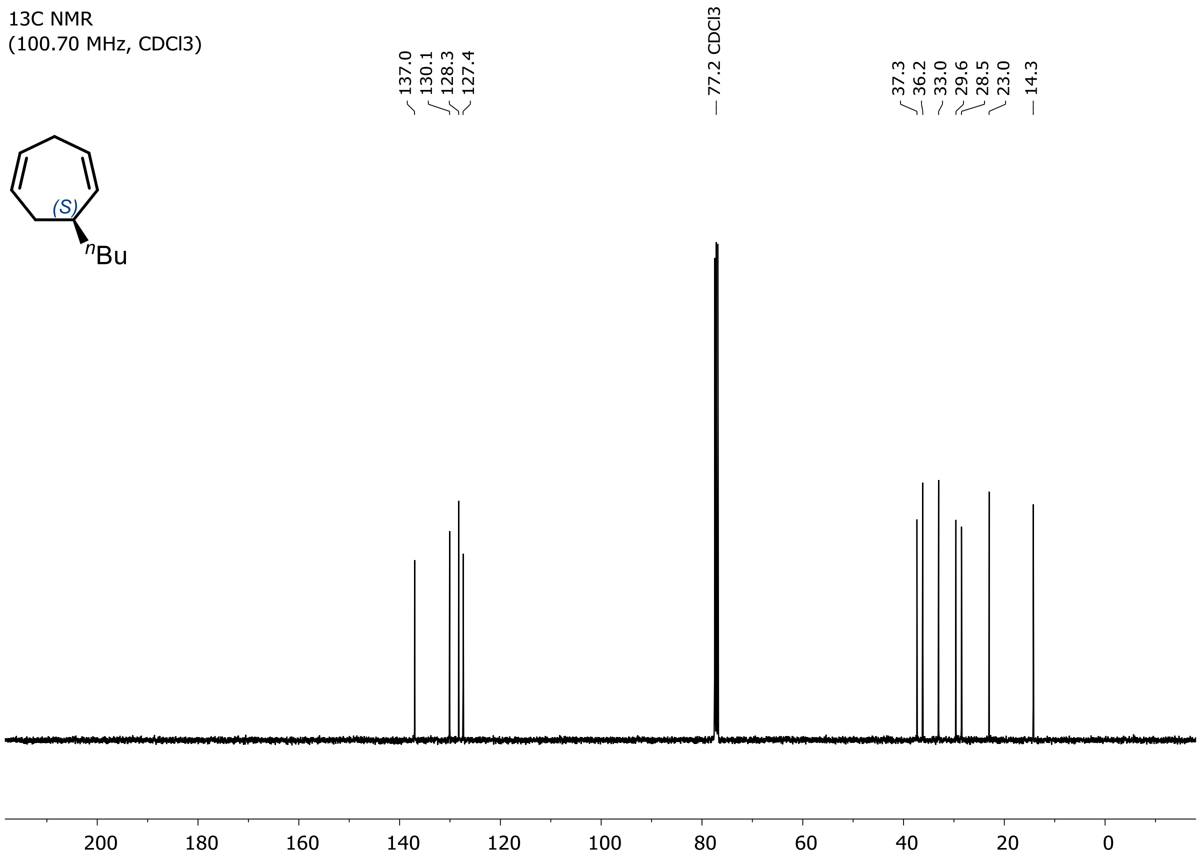

((E)-3-(*trans*-2-vinylcyclopropyl)allyl)benzene (41)

1H NMR
(600.44 MHz, CDCl3)

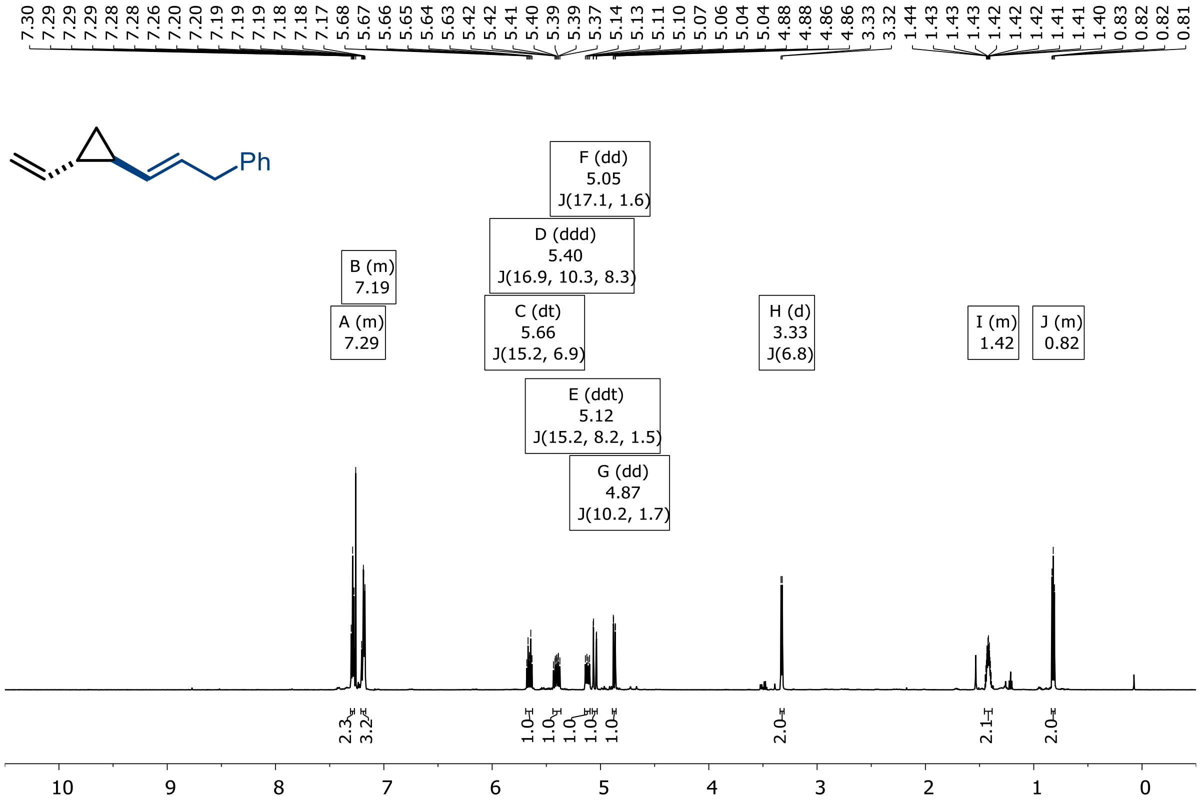

13C NMR
(151.00 MHz, CDCl3)

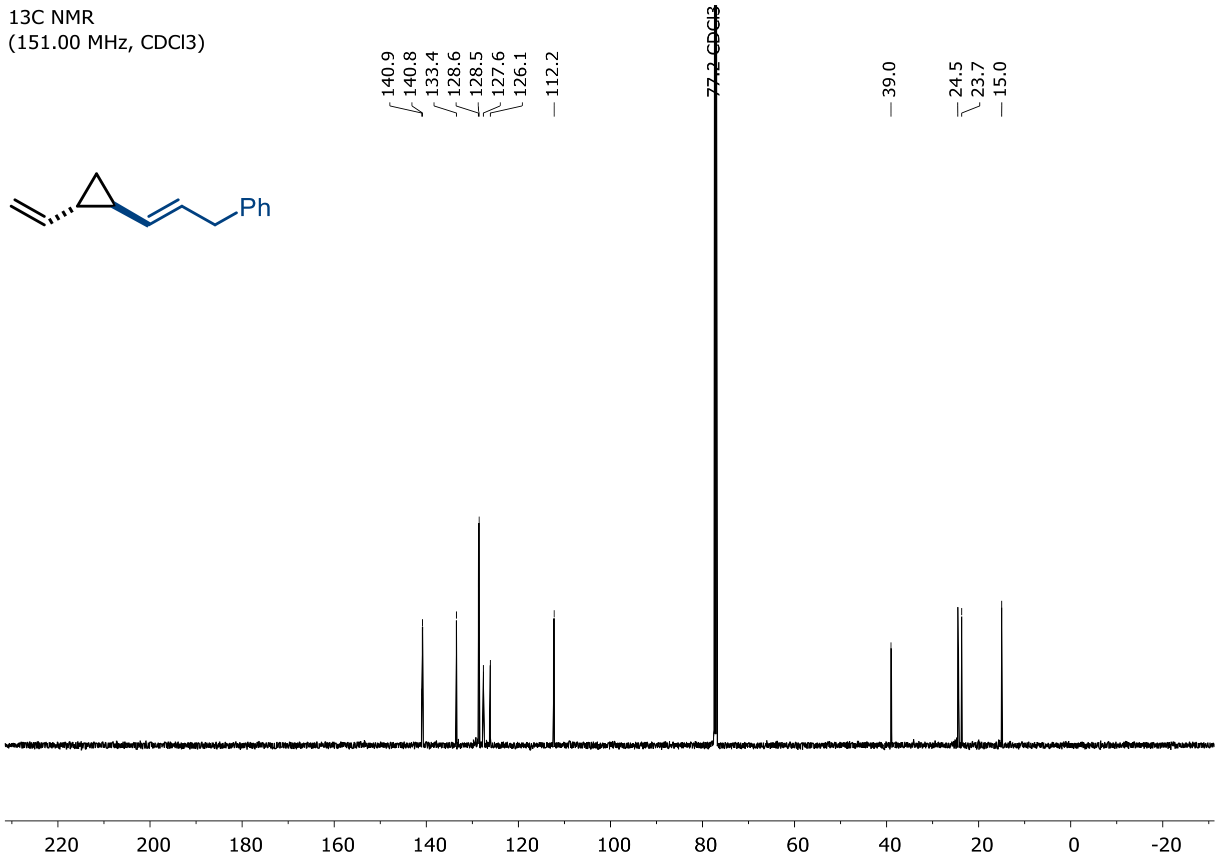

6-benzylcyclohepta-1,4-diene (42)

1H NMR
(600.44 MHz, CDCl3)

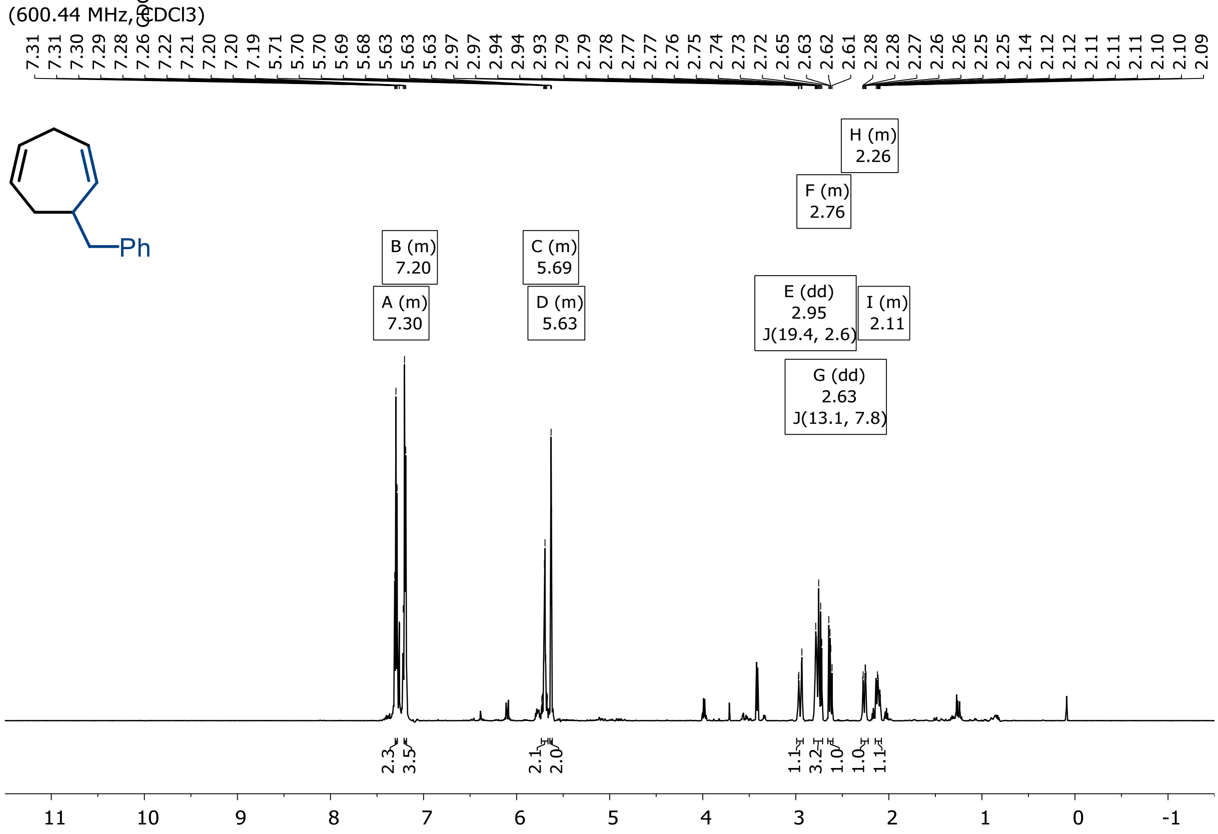

13C NMR
(151.00 MHz, CDCl3)

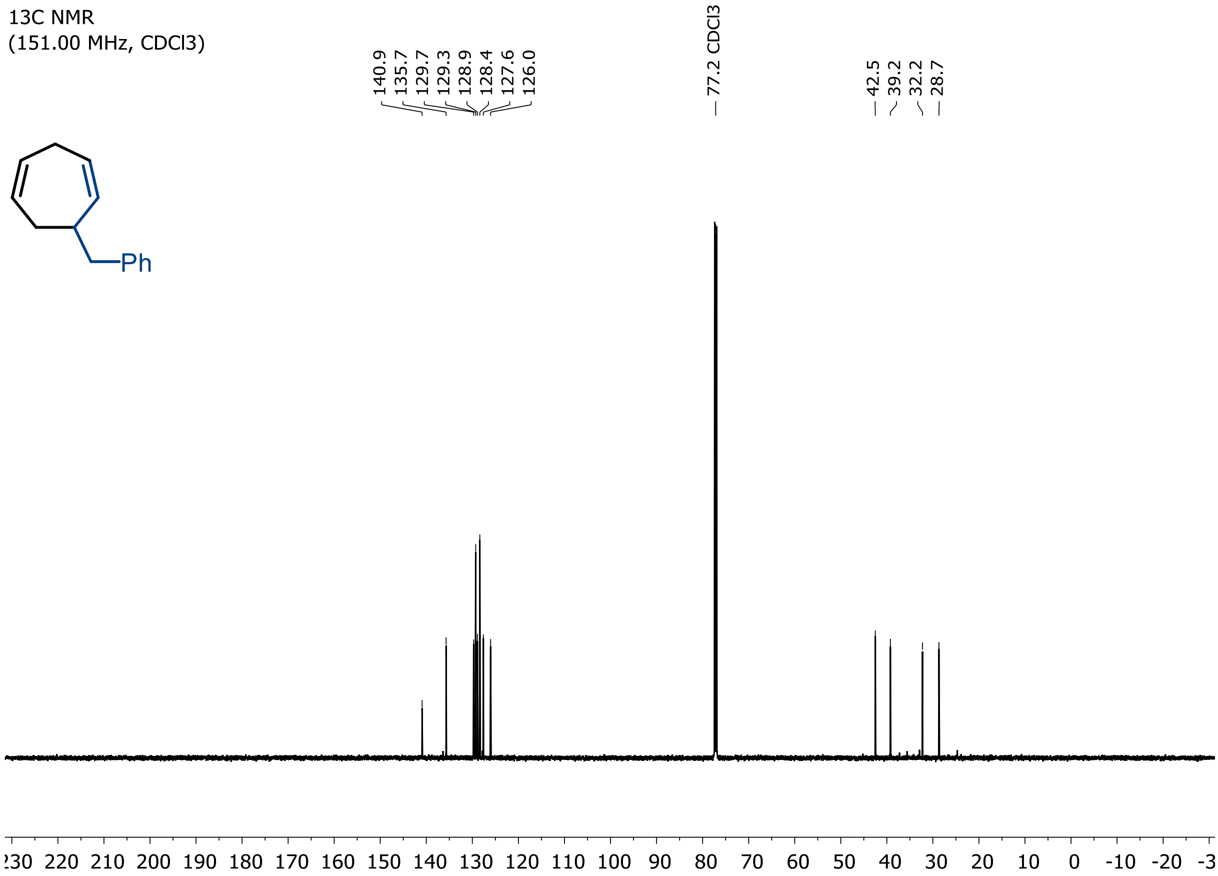

Triethyl((1-((*trans*)-2-vinylcyclopropyl)non-1-en-1-yl)oxy)silane (43)

1H NMR
(600.44 MHz, CD2Cl2)

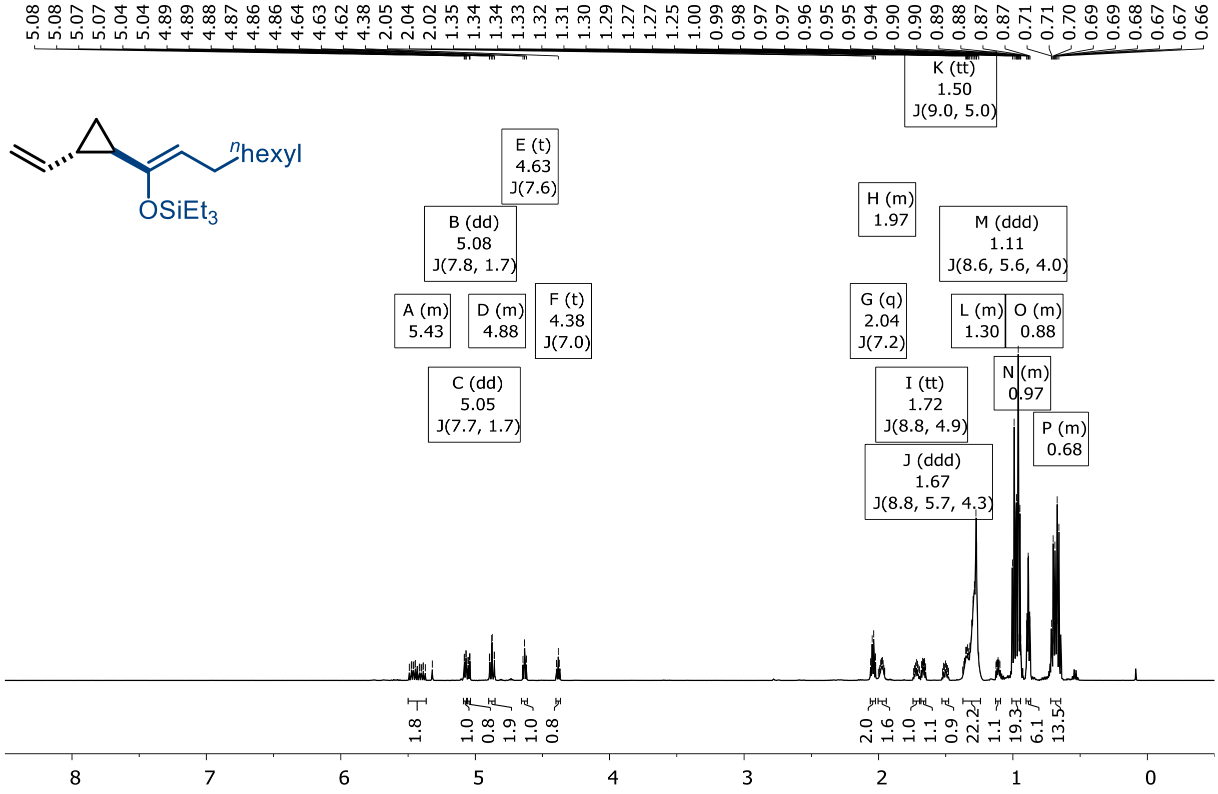

13C NMR
(151.00 MHz, CD2Cl2)

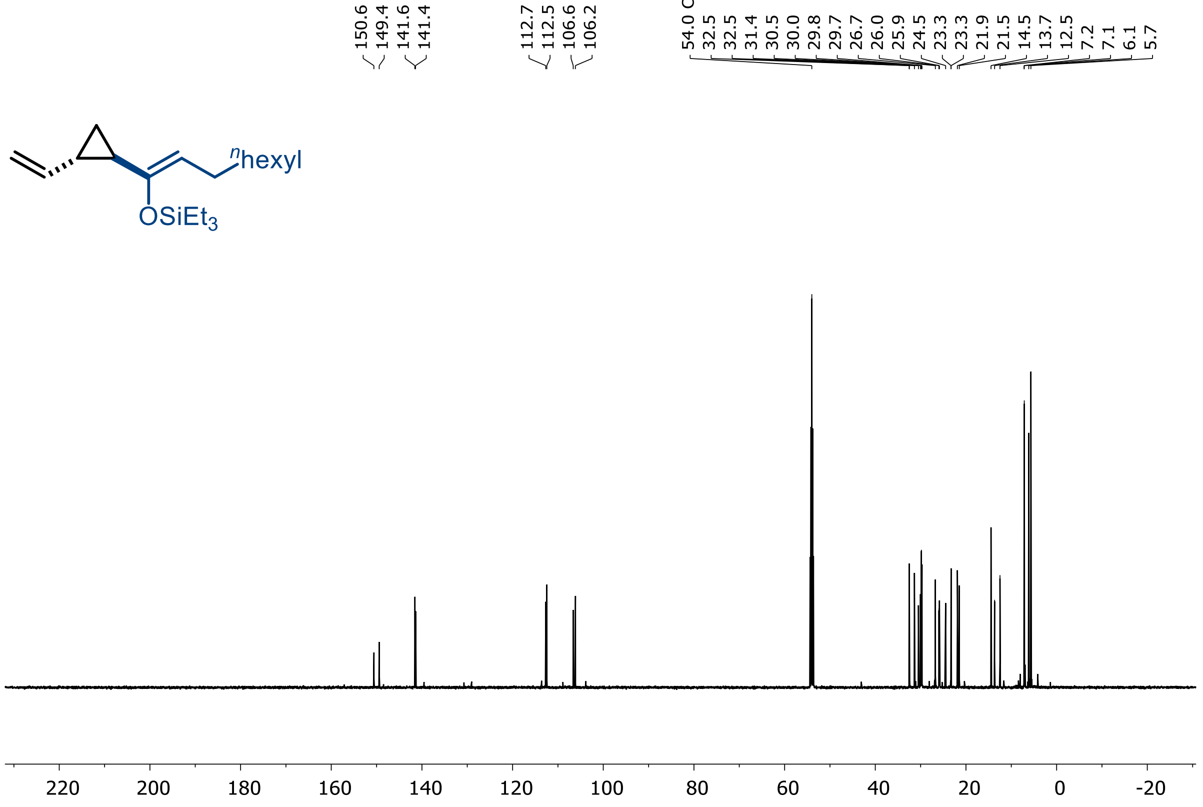

Triethyl((7-heptylcyclohepta-1,4-dien-1-yl)oxy)silane (44)

1H NMR
(600.44 MHz, CD2Cl2)

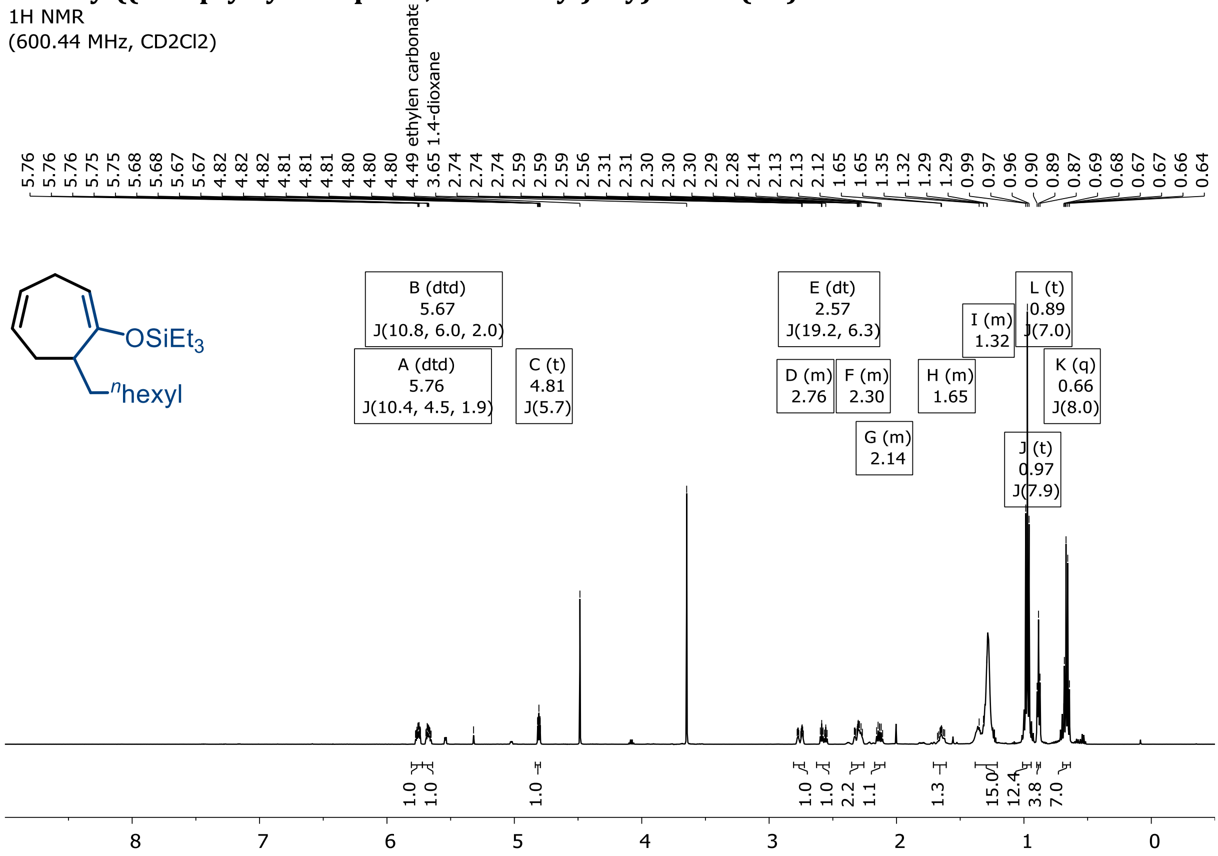

2-Heptylcyclohept-4-en-1-one (45)

1H NMR
(400.44 MHz, CDCl3)

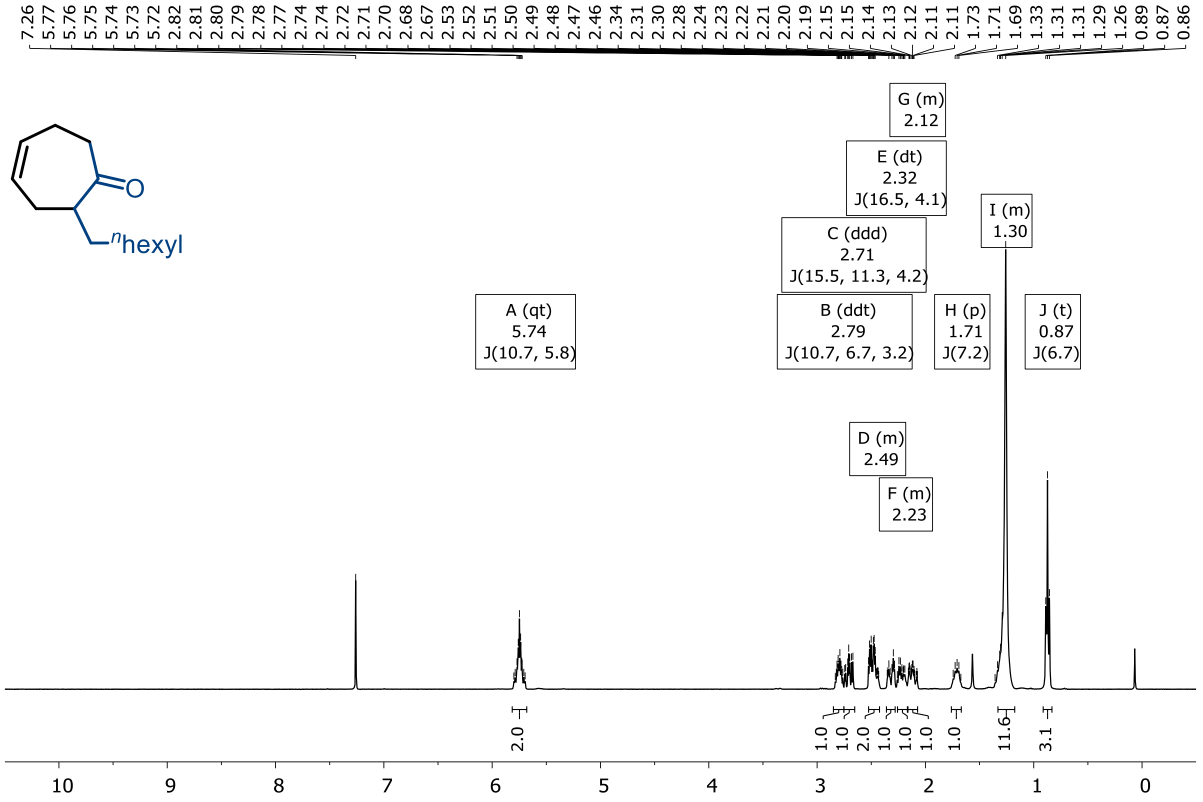

13C NMR
(100.70 MHz, CDCl3)

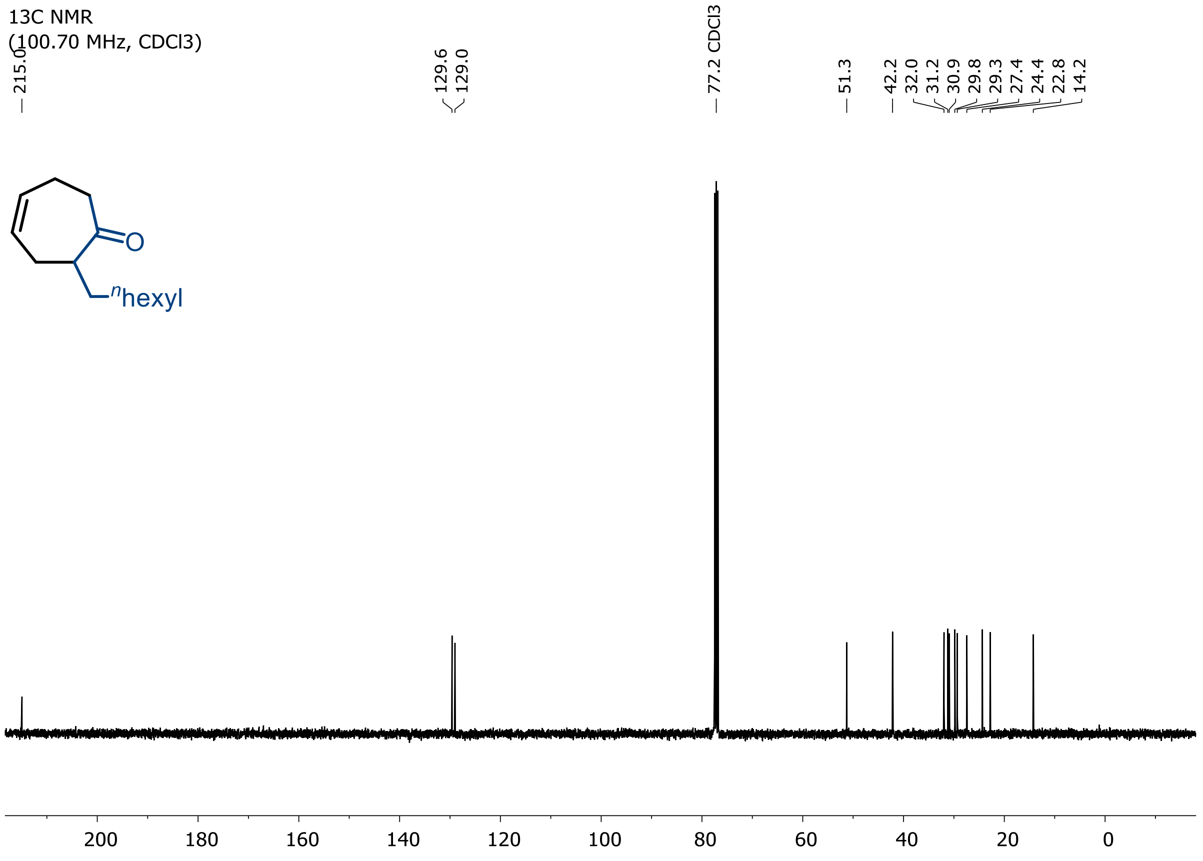

1-((*trans*)-2-vinylcyclopropyl)cyclohex-1-ene (S46)

1H NMR
(600.44 MHz, CDCl3)

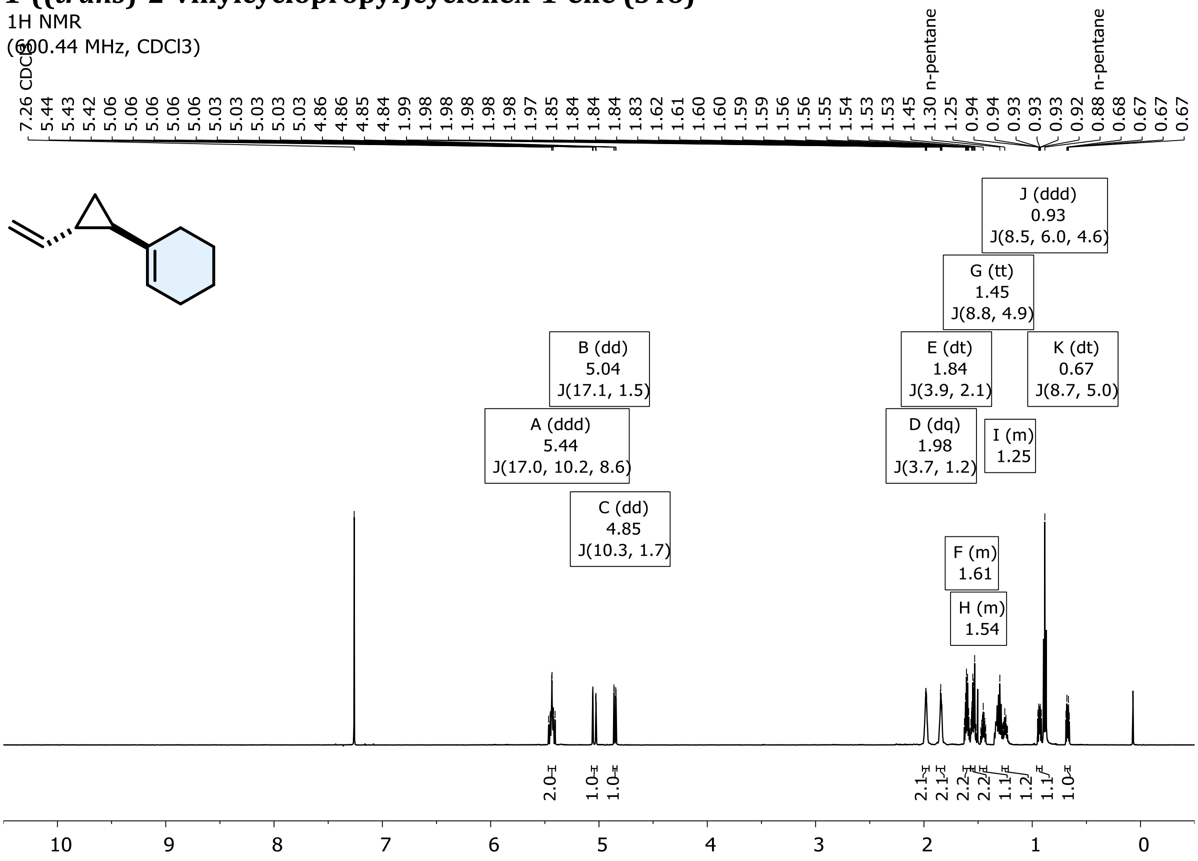

13C NMR
(151.00 MHz, CDCl3)

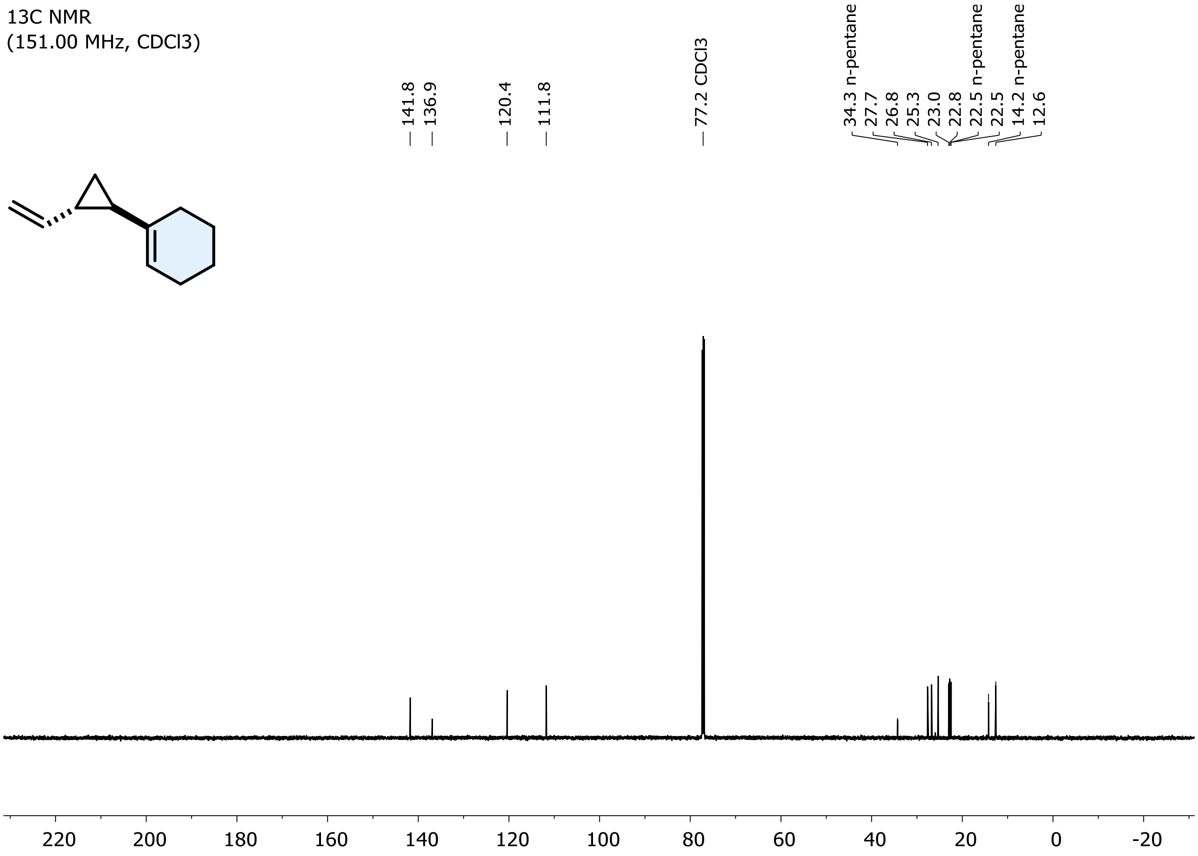

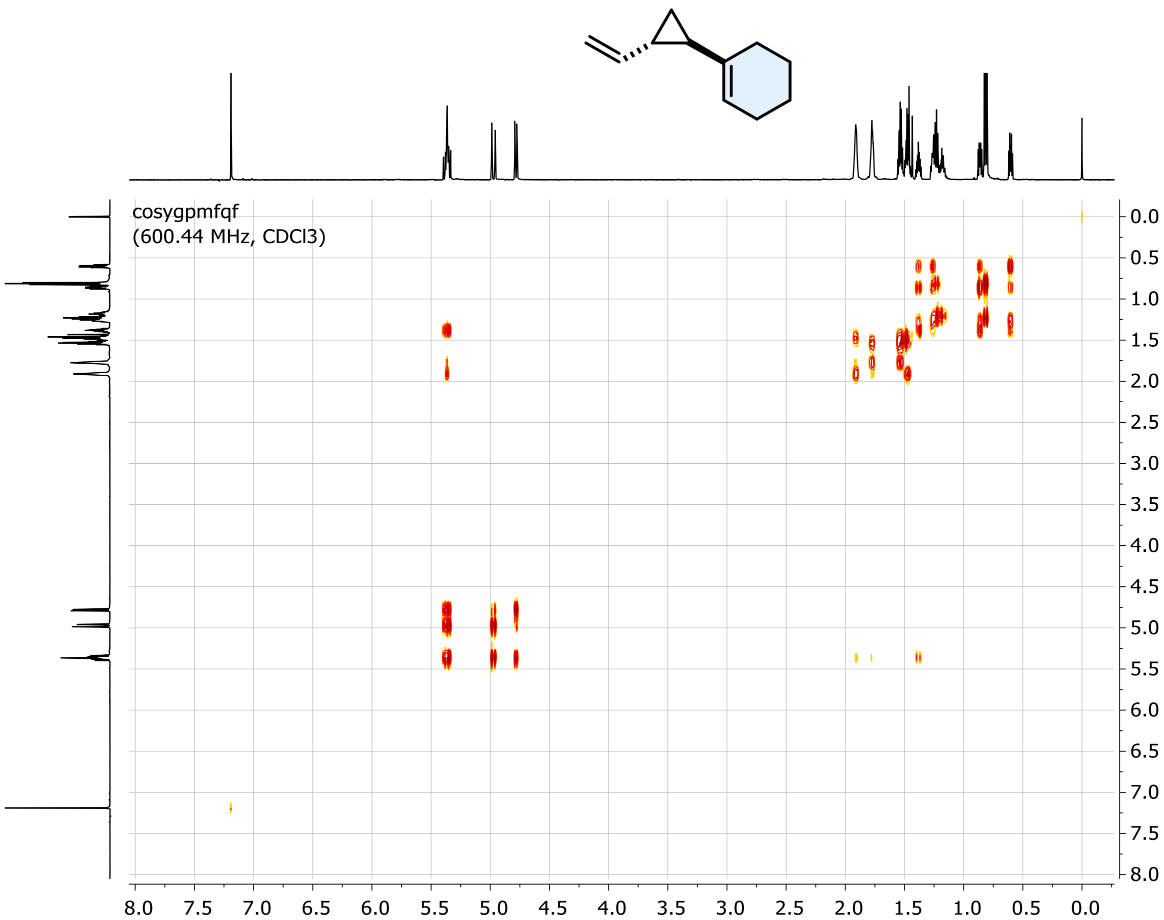
cosygpmfqf
(600.44 MHz, CDCl3)

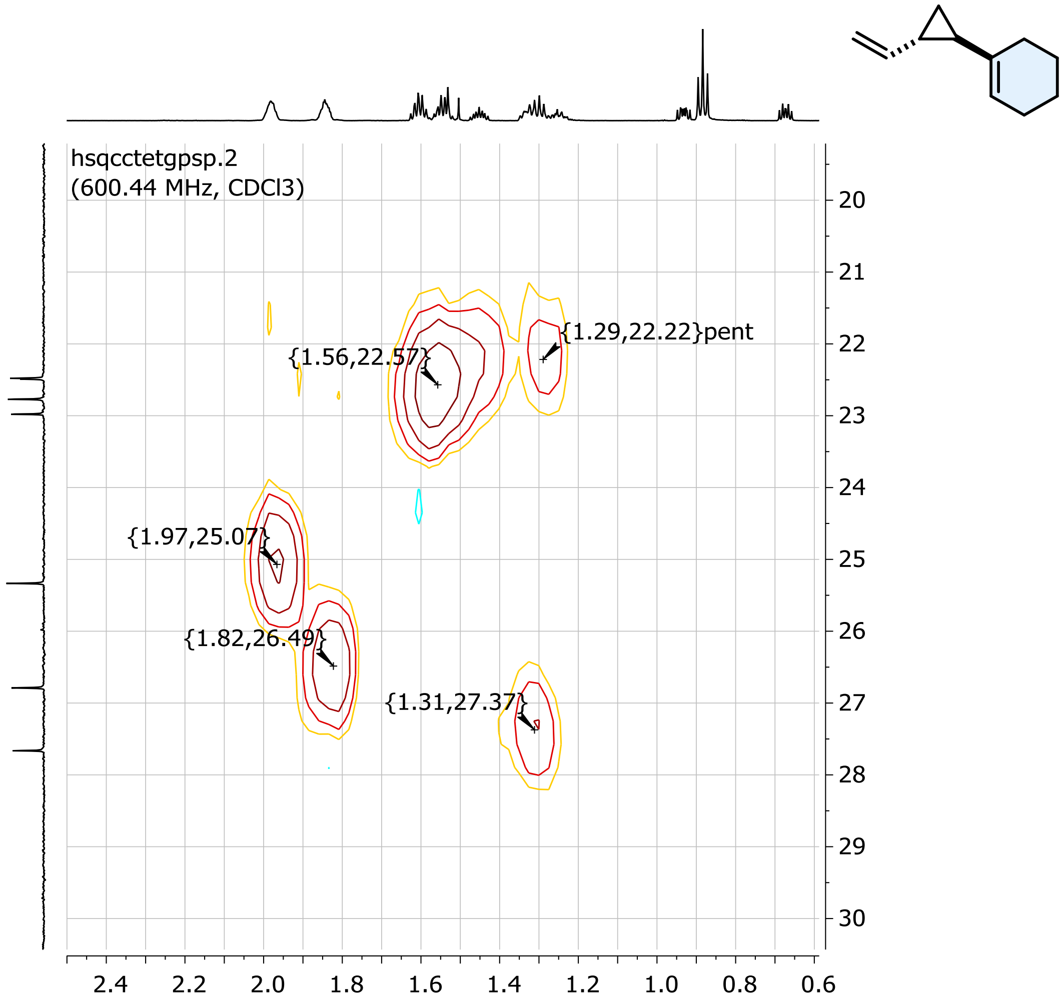
hsqcctetgpsp.2
(600.44 MHz, CDCl3)
{1.56,22.57}
{1.29,22.22}pent
{1.97,25.07}
{1.82,26.49}
{1.31,27.37}

2,3,4,4a,5,8-hexahydro-1*H*-benzo[7]annulene (46)

1H NMR
(600.44 MHz, CDCl3)

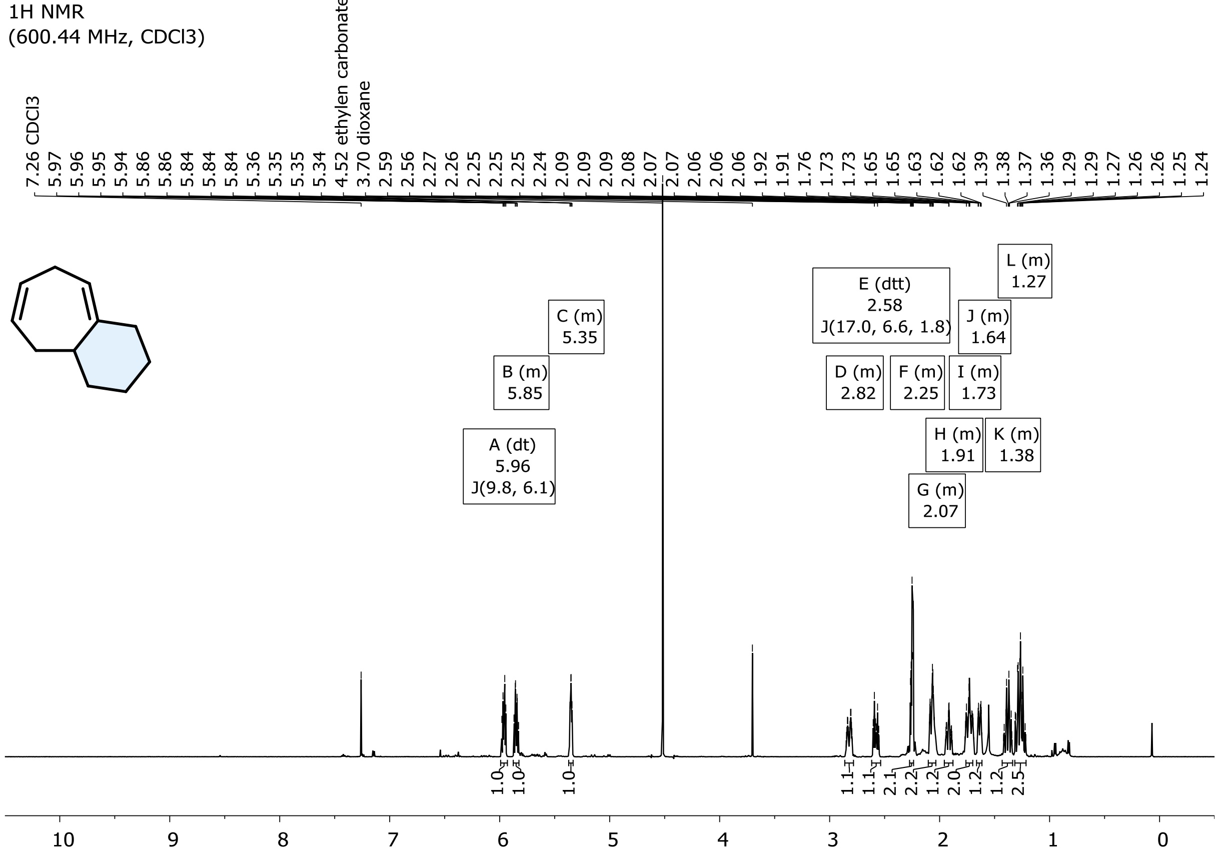

13C NMR
(151.00 MHz, CDCl3)

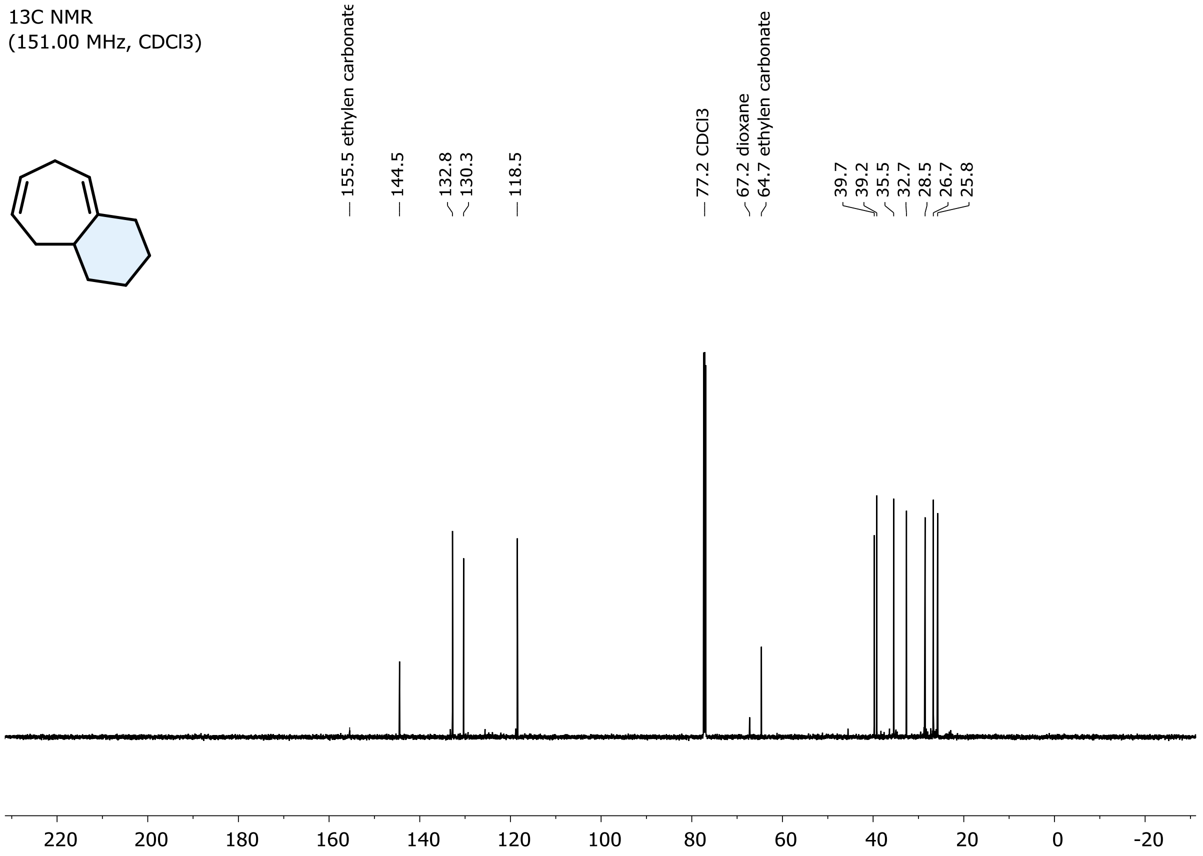

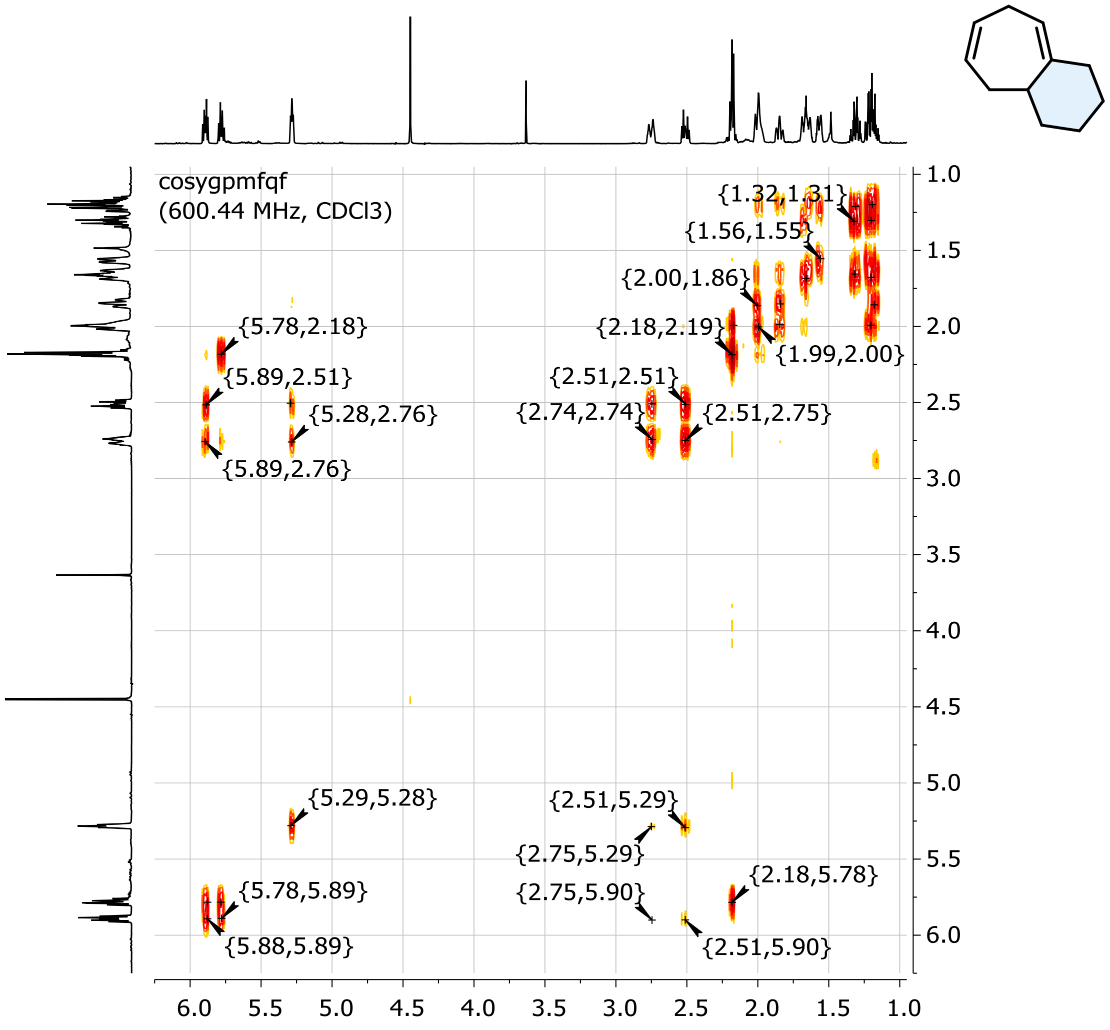
cosygpmfqf
(600.44 MHz, CDCl3)
{1.32,1.31}
{1.56,1.55}
{2.00,1.86}
{2.18,2.19}
{1.99,2.00}
{5.78,2.18}
{5.89,2.51}
{5.28,2.76}
{5.89,2.76}
{2.51,2.51}
{2.74,2.74}
{2.51,2.75}
{5.29,5.28}
{2.51,5.29}
{2.75,5.29}
{2.18,5.78}
{5.78,5.89}
{2.75,5.90}
{5.88,5.89}
{2.51,5.90}

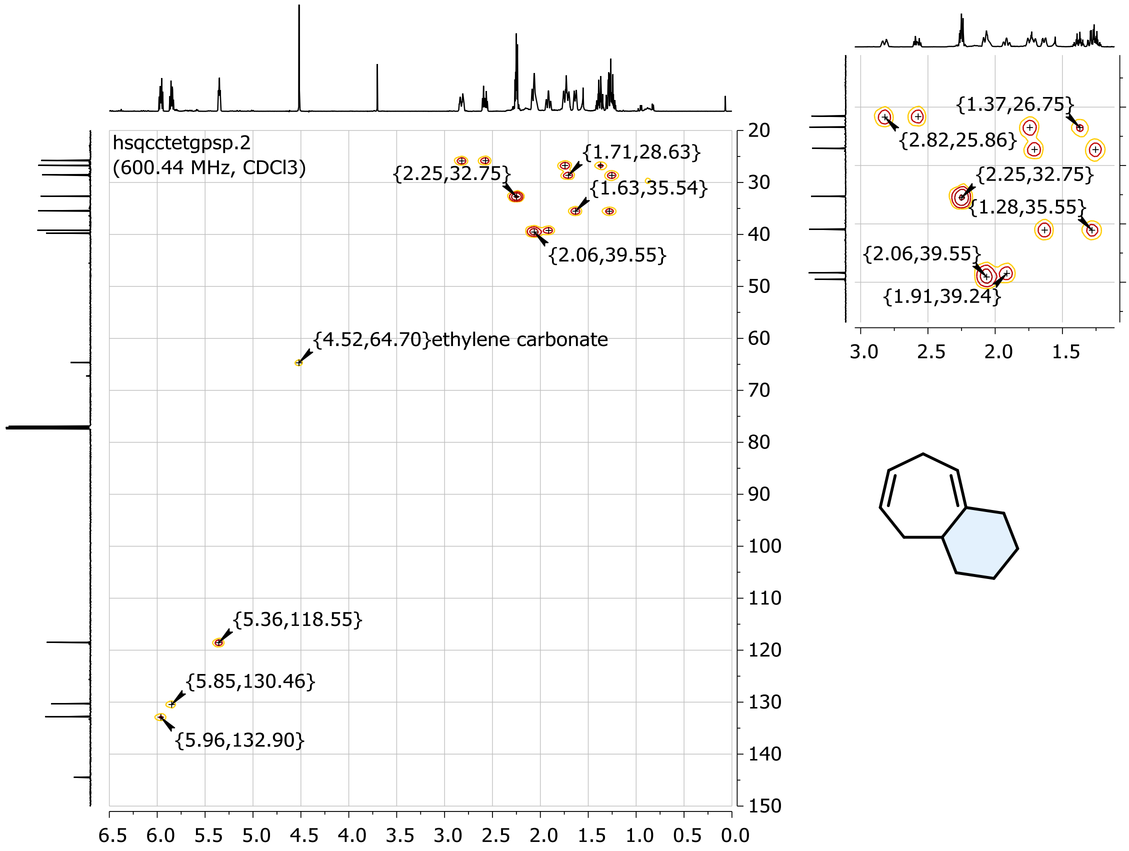
hsqcctetgpsp.2
(600.44 MHz, CDCl3)
{1.71,28.63}
{2.25,32.75}
{1.63,35.54}
{2.06,39.55}
{4.52,64.70}ethylene carbonate
{5.36,118.55}
{5.85,130.46}
{5.96,132.90}
{1.37,26.75}
{2.82,25.86}
{2.25,32.75}
{1.28,35.55}
{2.06,39.55}
{1.91,39.24}

1-(*trans*-2-vinylcyclopropyl)cyclohept-1-ene (S47)

1H NMR
(400.44 MHz, CDCl3)

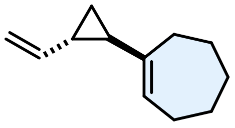

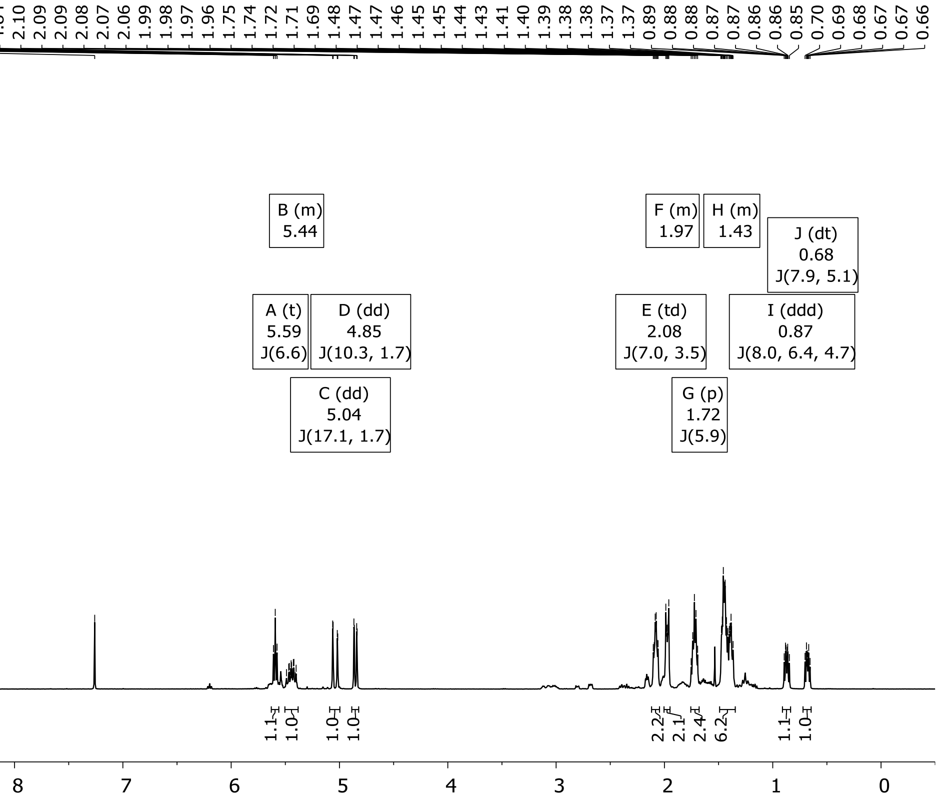

13C NMR
(100.70 MHz, CDCl3)

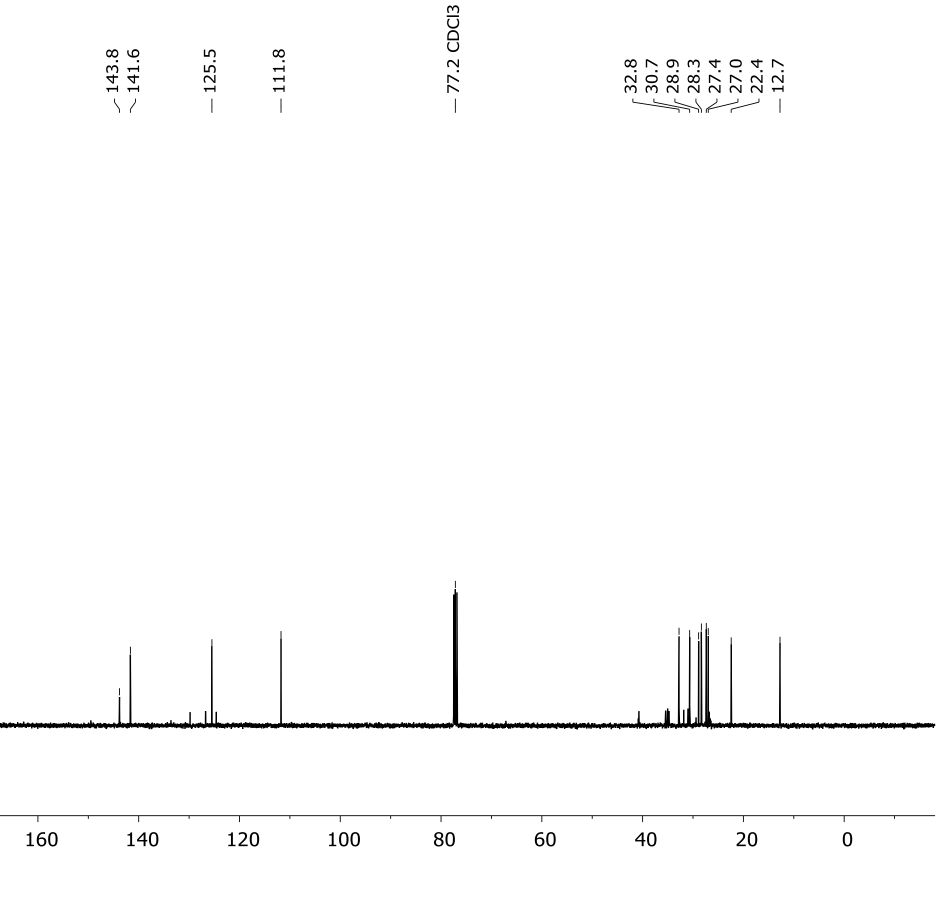

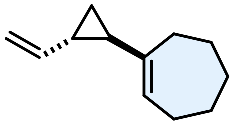

1,2,3,4,5,5a,6,9-octahydroheptalene (47)

1H NMR
(600.44 MHz, CDCl3)

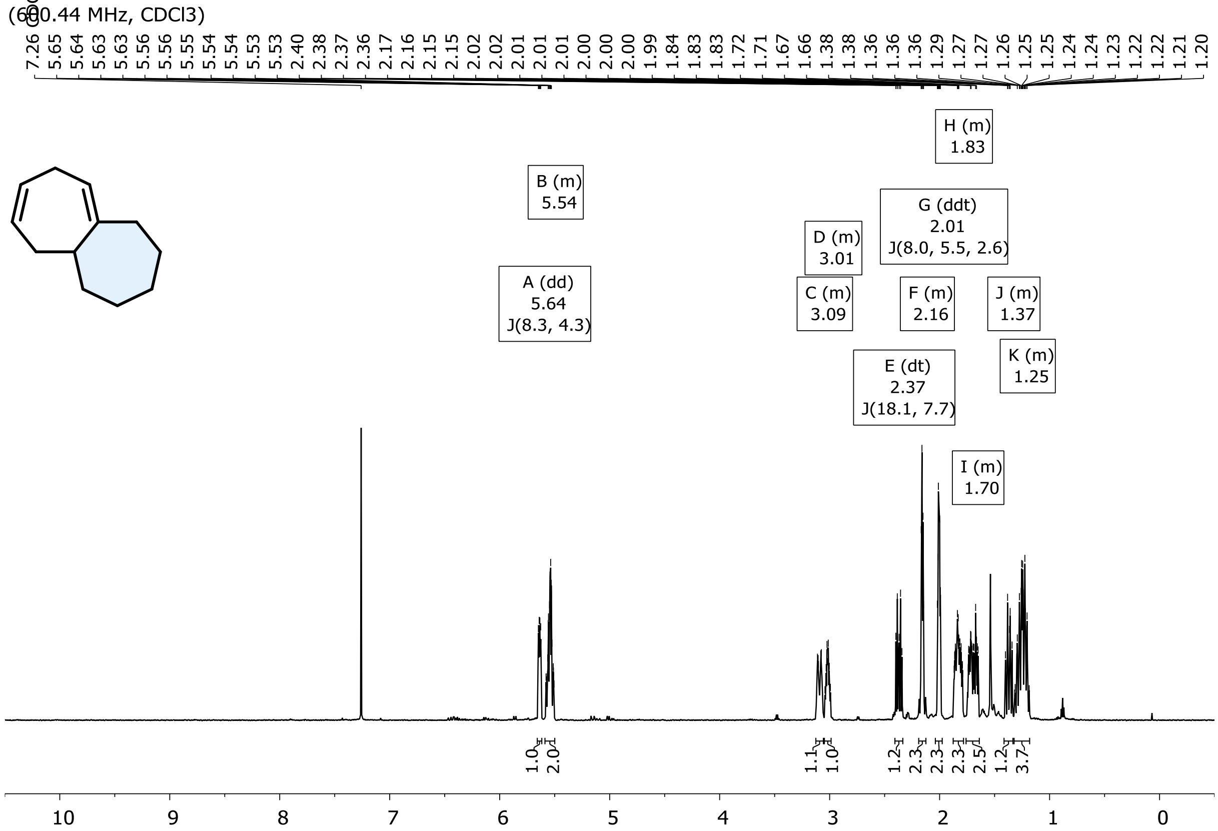

13C NMR
(151.00 MHz, CDCl3)

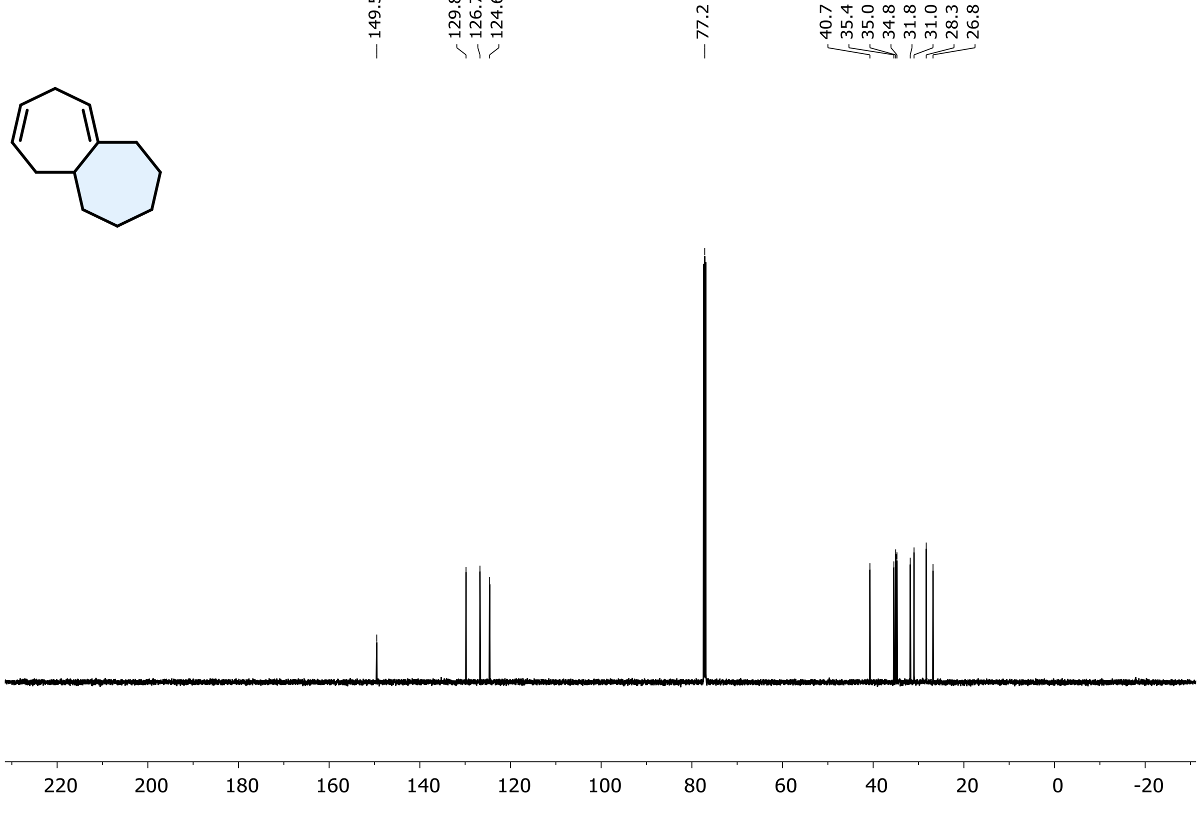

4-((*trans*)-2-vinylcyclopropyl)-3,6-dihydro-2*H*-pyran (*S*48)

1H NMR
(600.44 MHz, CDCl3)

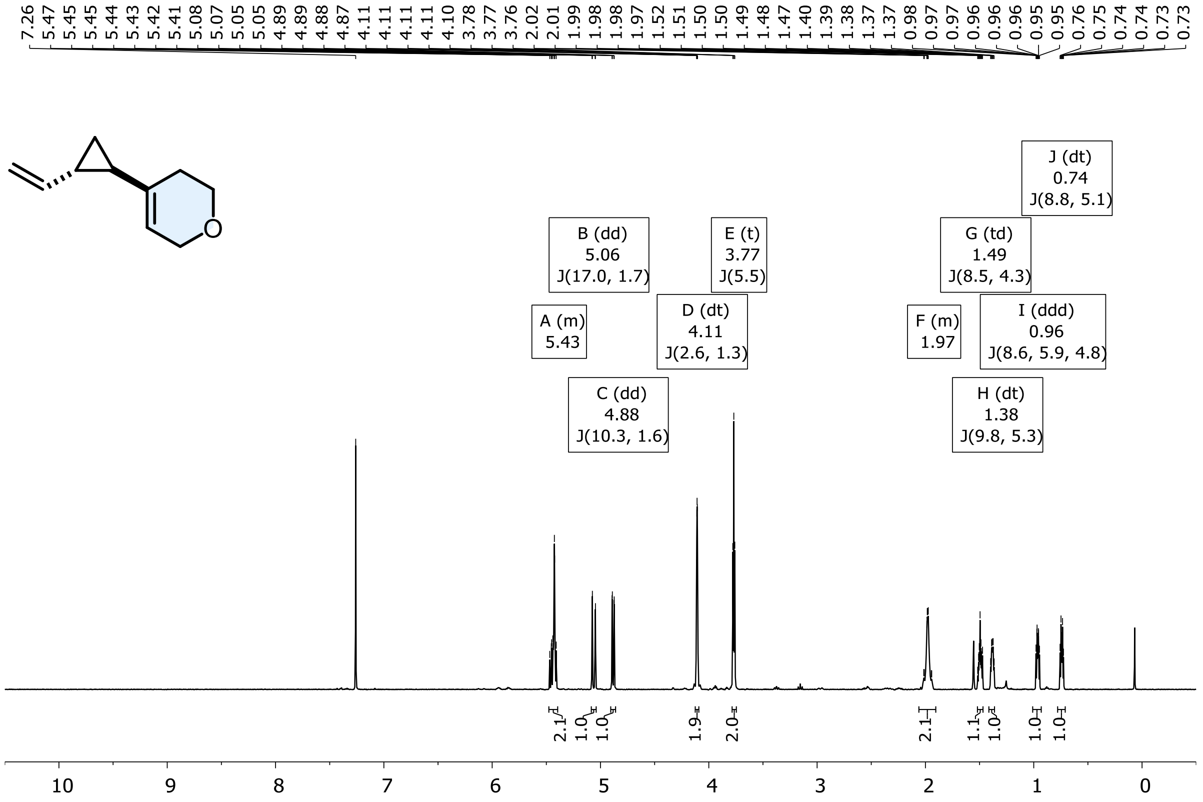

13C NMR
(151.00 MHz, CDCl3)

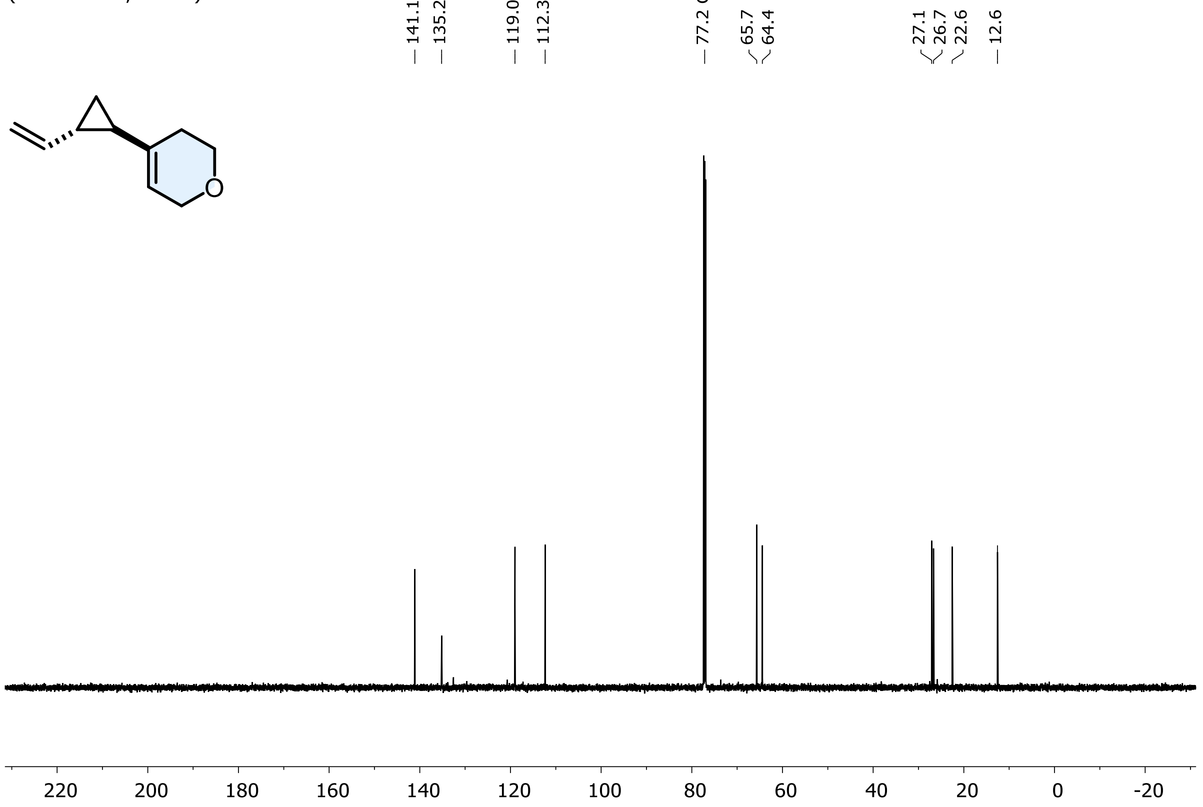

1,3,4,6,9,9a-Hexahydrocyclohepta[c]pyran (48)

1H NMR
(600.44 MHz, CD2Cl2)

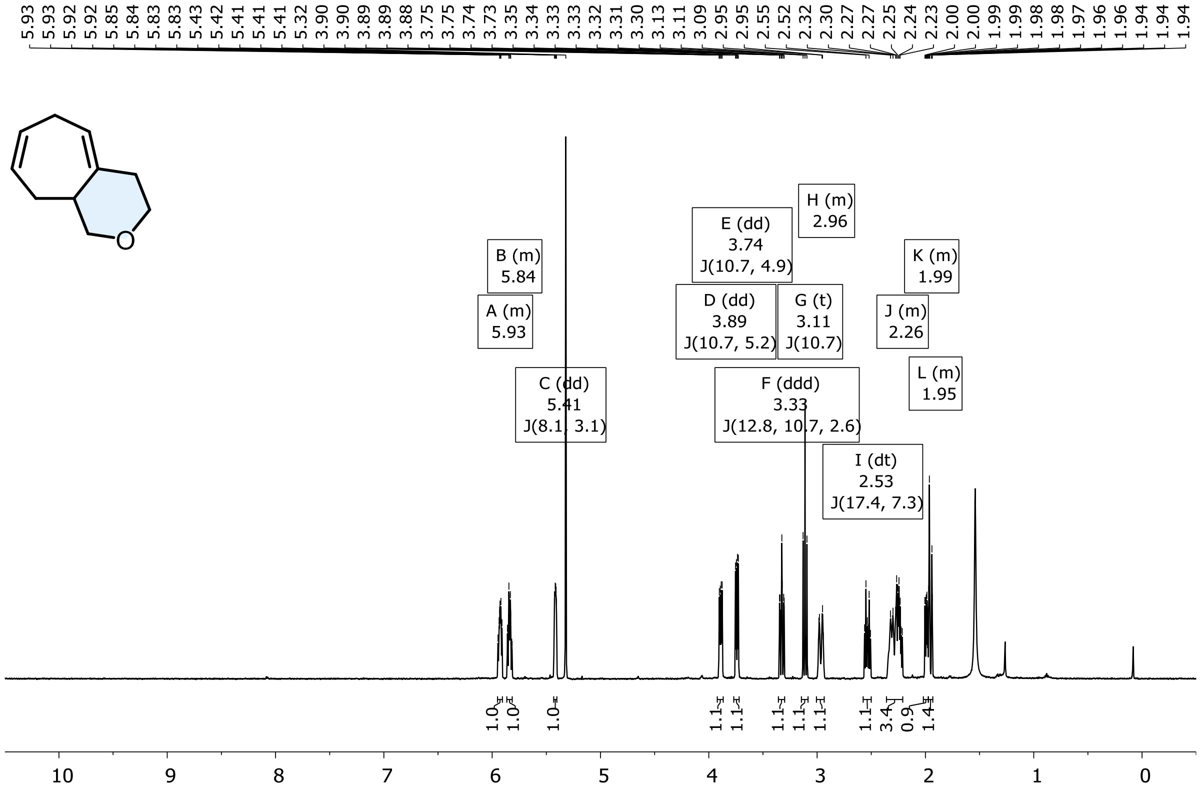

13C NMR
(151.00 MHz, CD2Cl2)

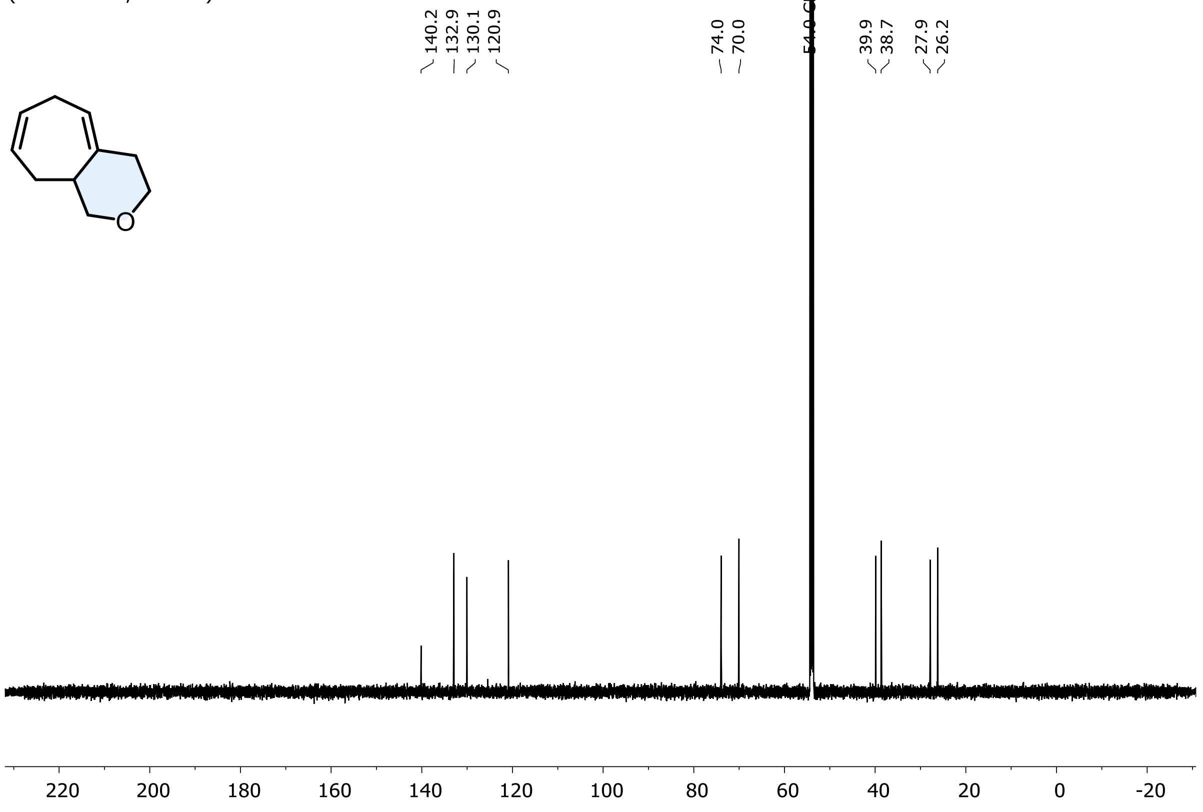

tert-butyl 4-((_trans_)-2-vinylcyclopropyl)-3,6-dihydropyridine-1(2_H_)-carboxylate (_S_49)

1H NMR
(600.44 MHz, CDCl3)

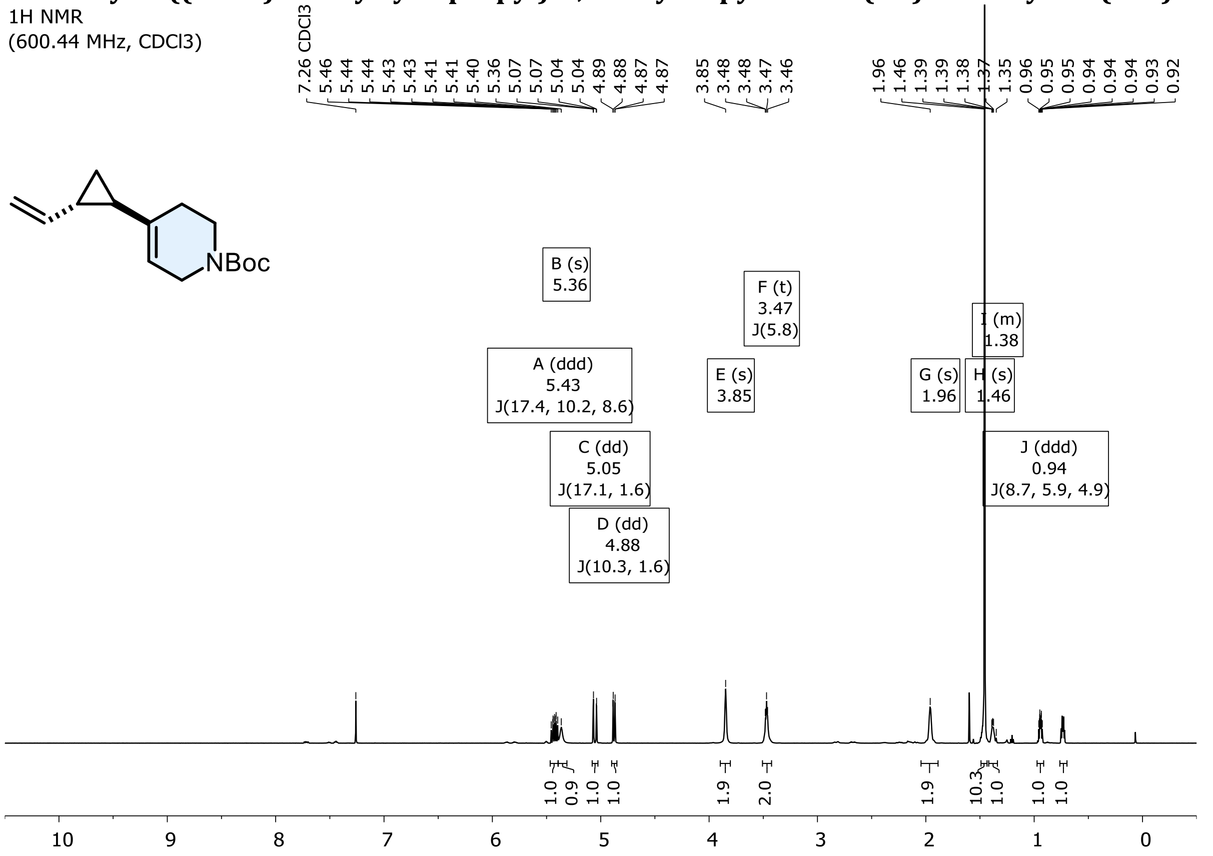

13C NMR
(100.58 MHz, CDCl3)

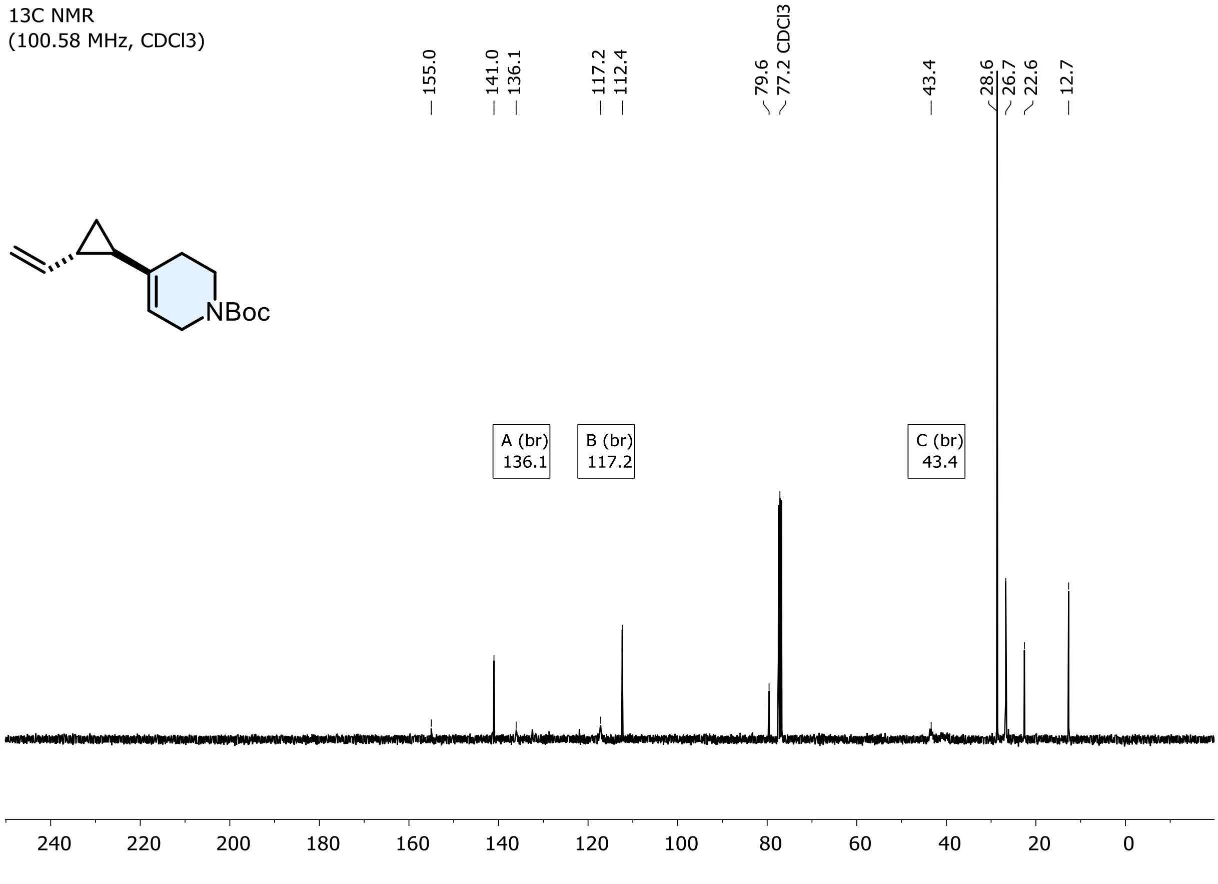

tert-butyl 1,3,4,6,9,9a-hexahydro-2_H_-cyclohepta[_c_]pyridine-2-carboxylate (49)

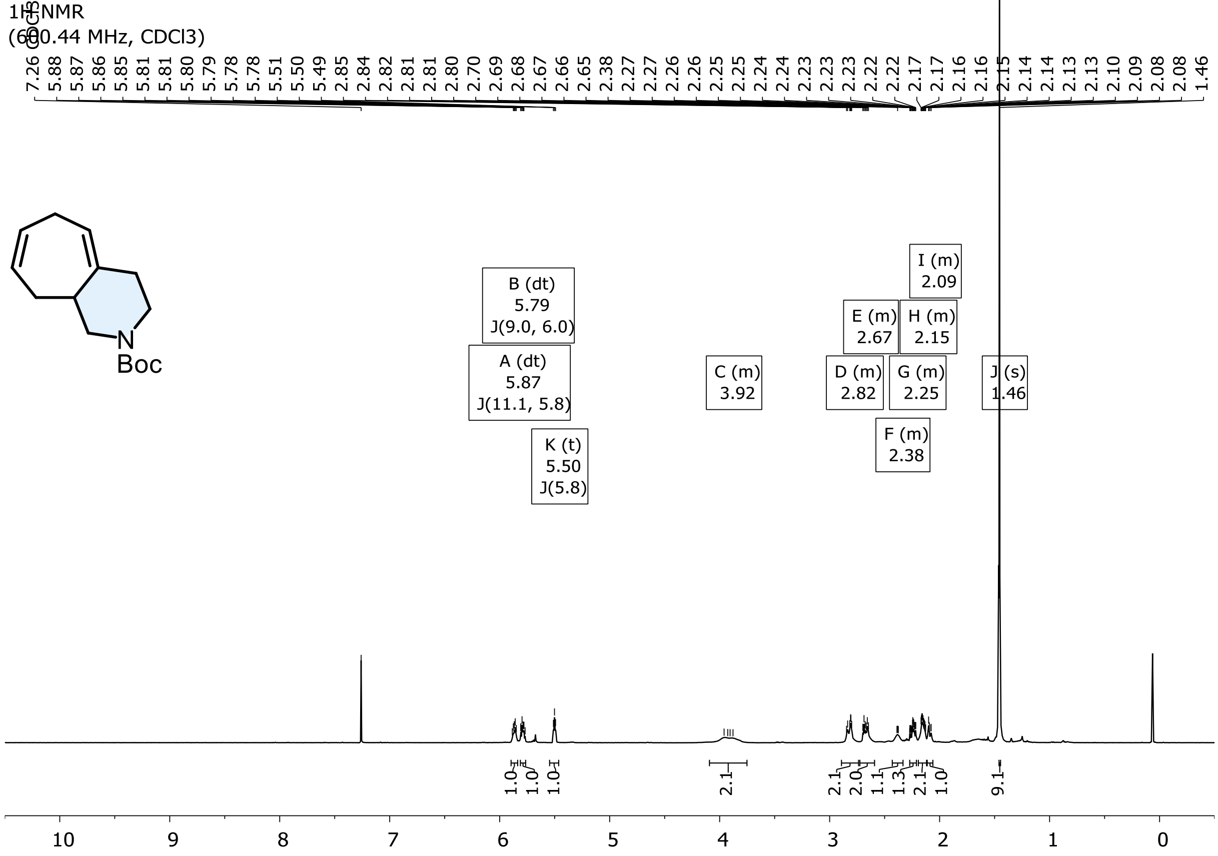

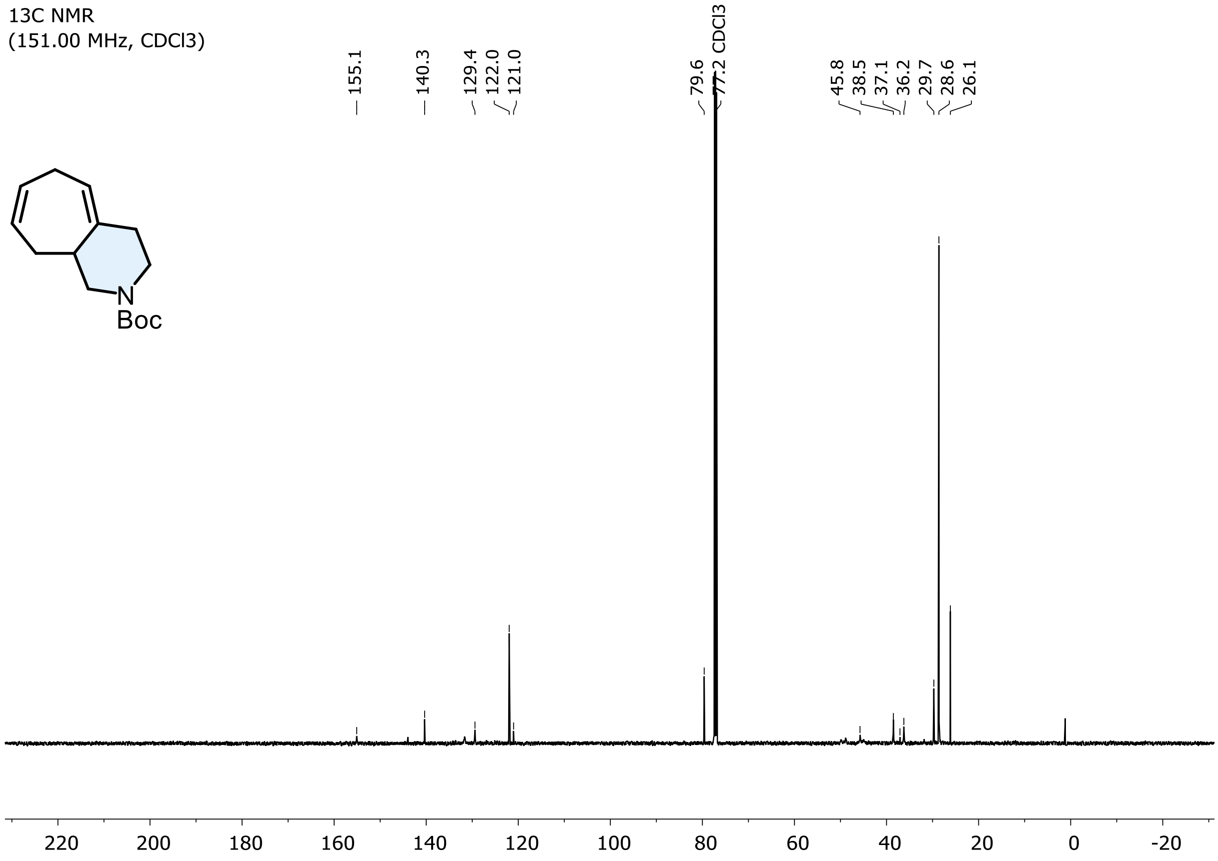

8-((*trans*)-2-vinylcyclopropyl)-1,4-dioxaspiro[4.5]dec-7-ene (*S50*)

1H NMR
(400.44 MHz, CDCl3)

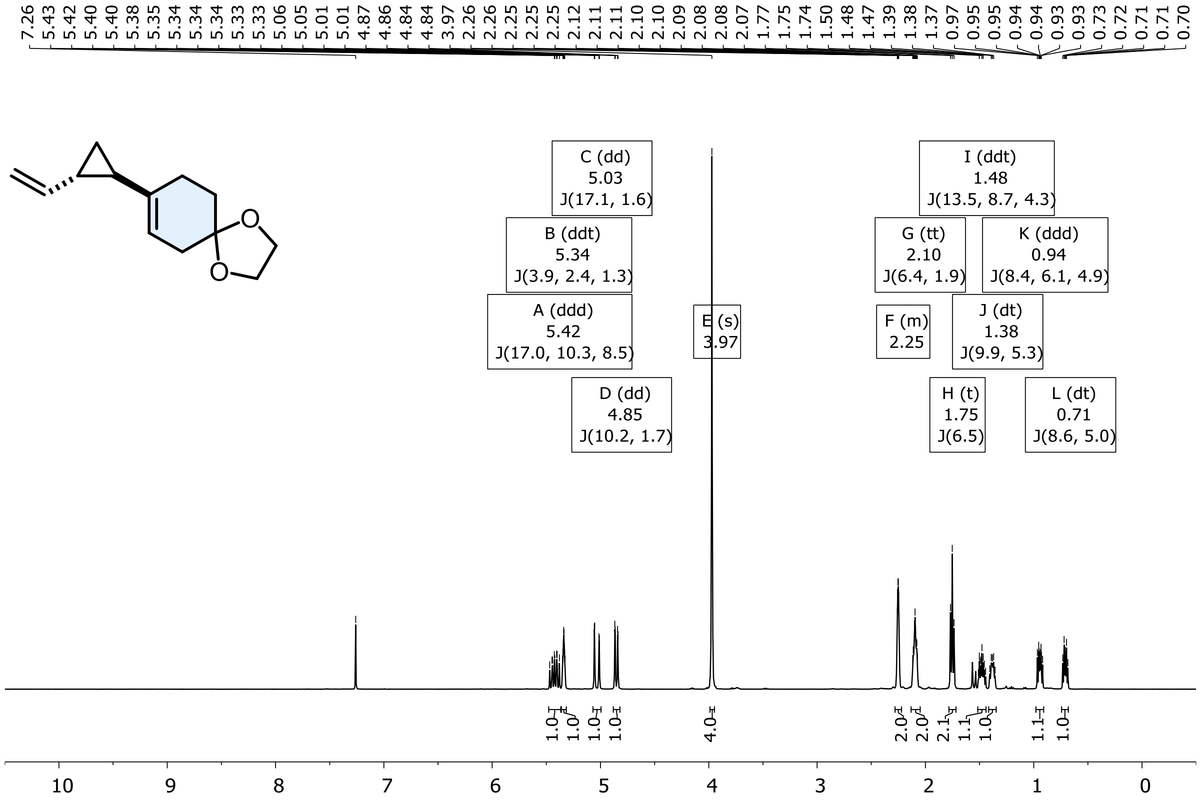

13C NMR
(100.70 MHz, CDCl3)

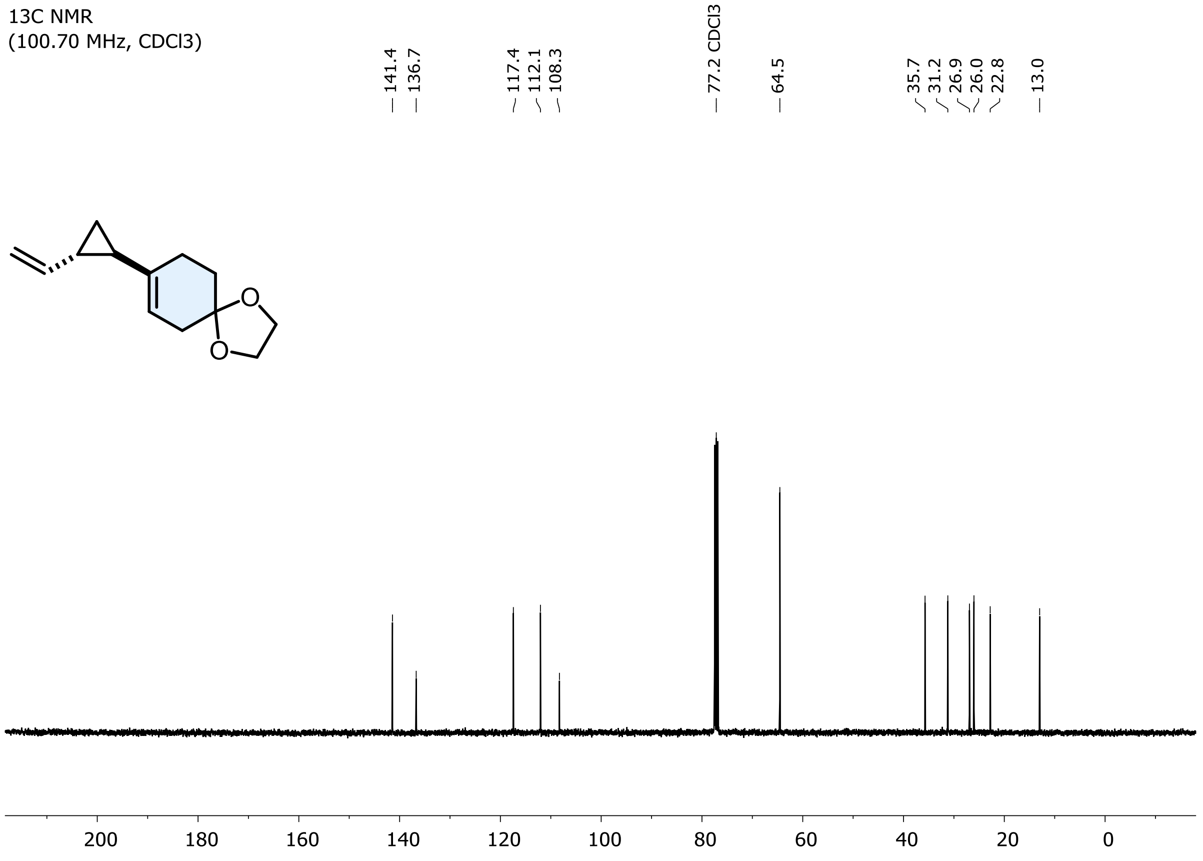

1,3,4,6,9,9a-hexahydrospiro[benzo[7]annulene-2,2'-[1,3]dioxolane] (50)

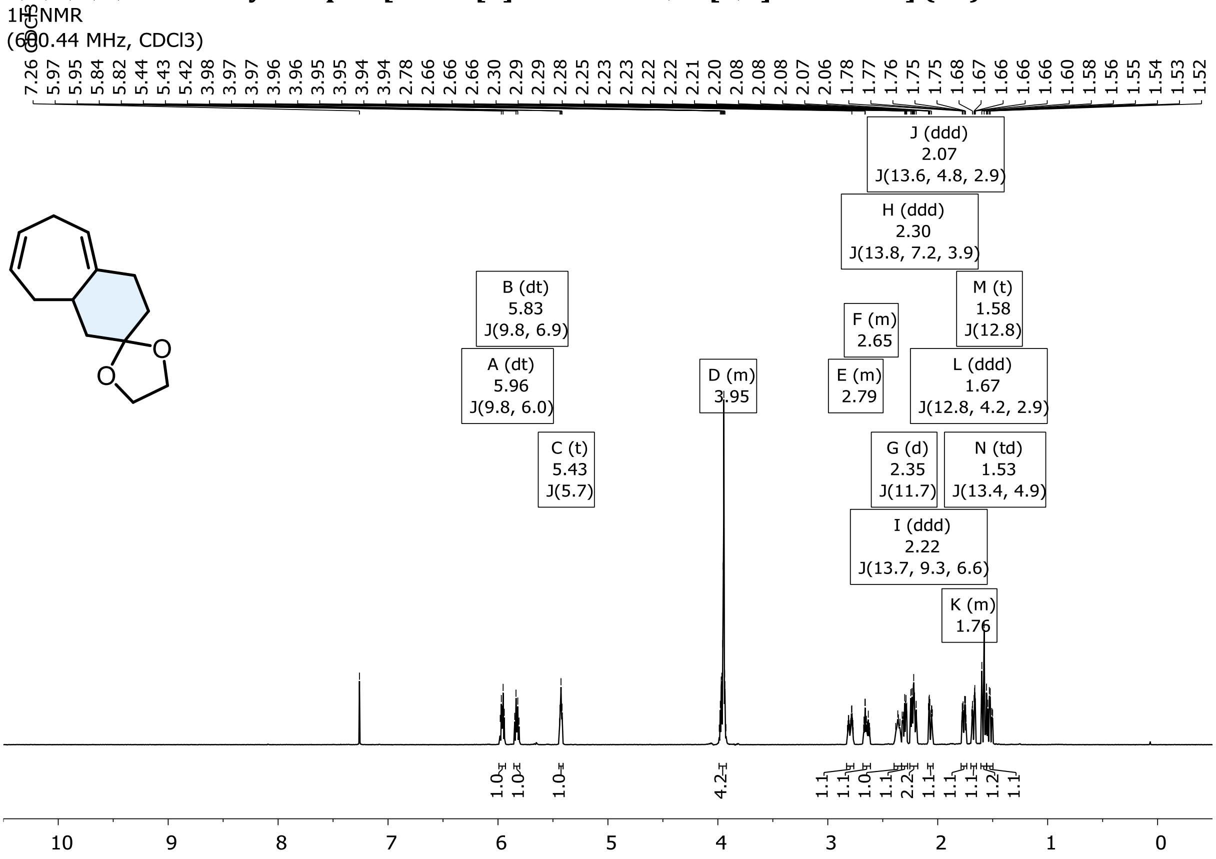

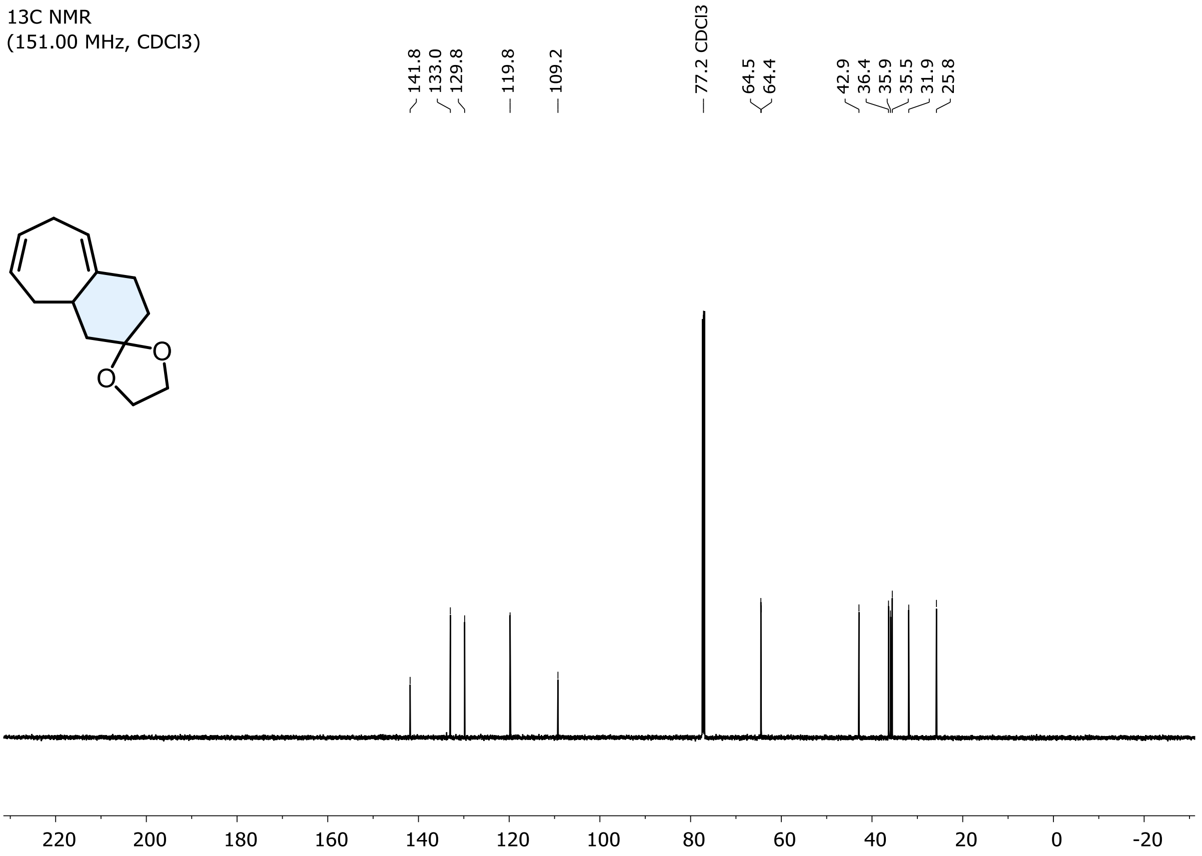

((*trans*-2-vinylcyclopropyl)methylene)cyclohexane (51)

1H NMR
(600.44 MHz, CDCl3)

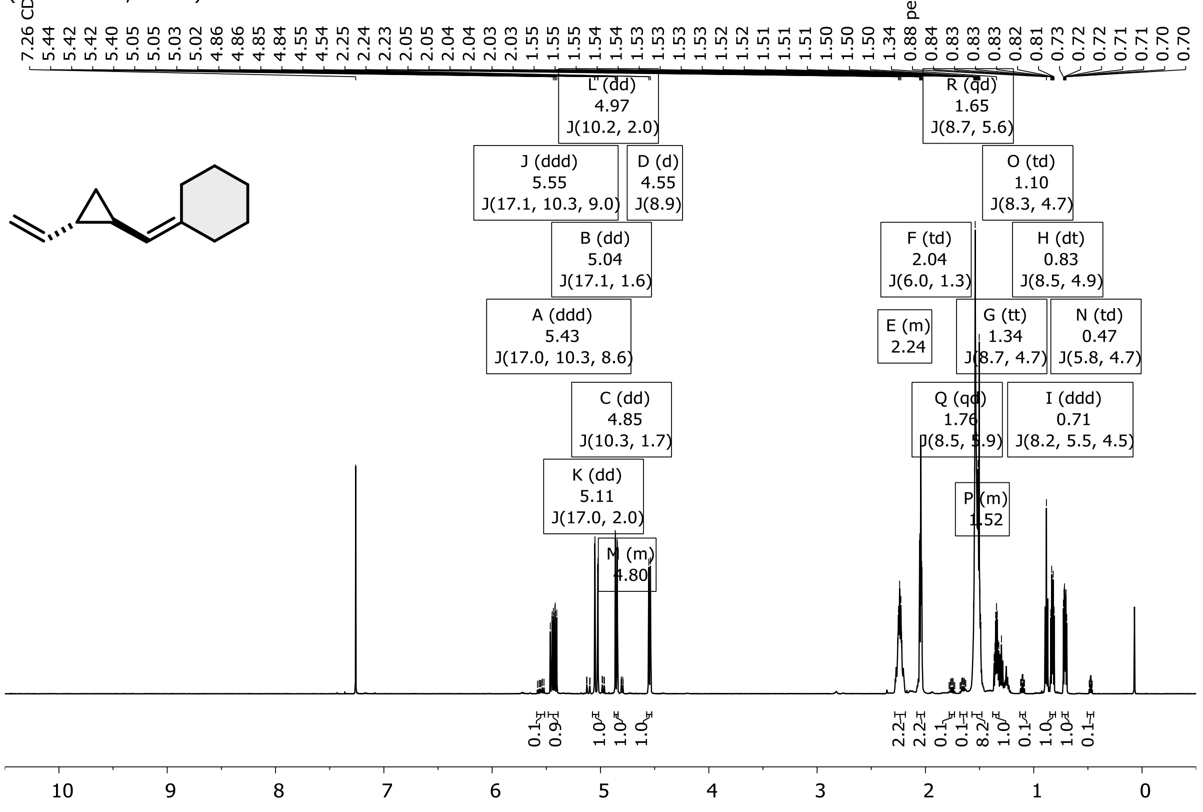

13C NMR
(151.00 MHz, CDCl3)

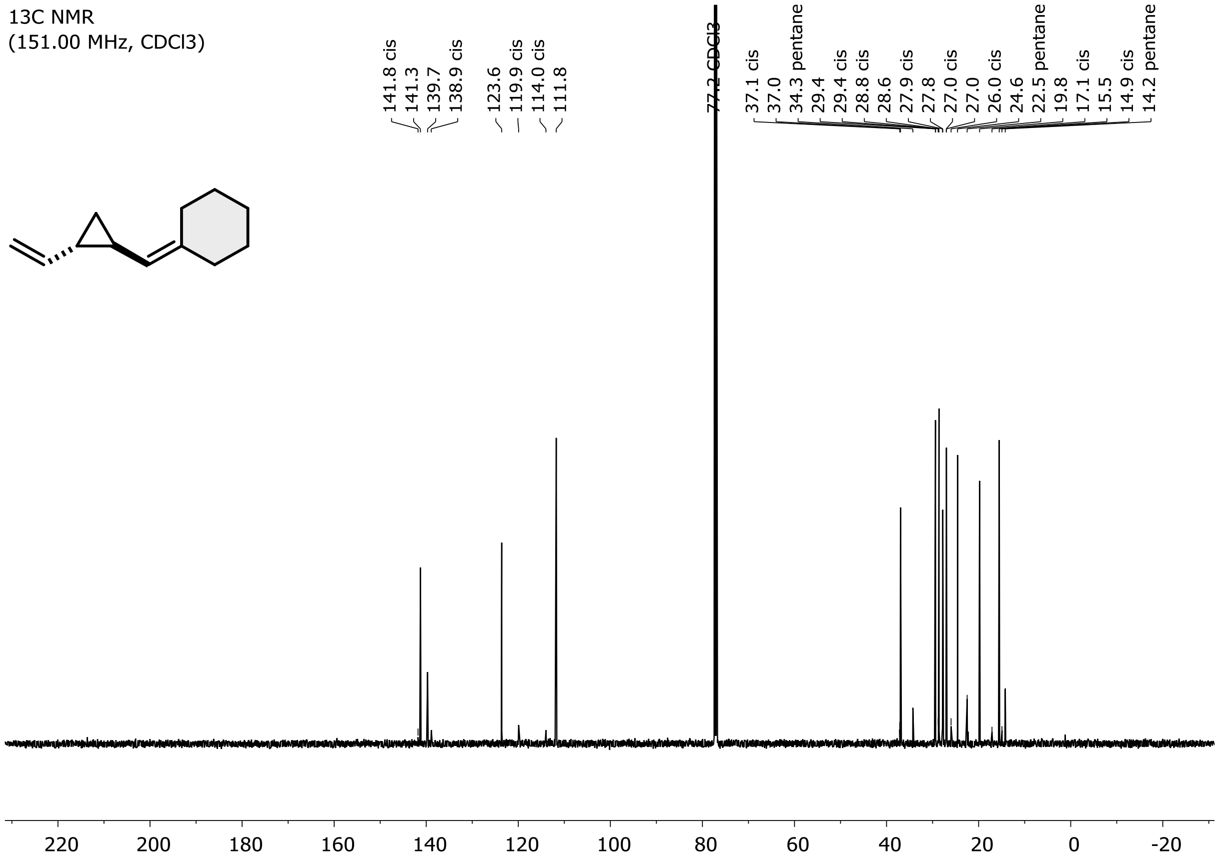

Spiro[5.6]dodeca-7,10-diene (52)

1H NMR
(400.44 MHz, CDCl3)

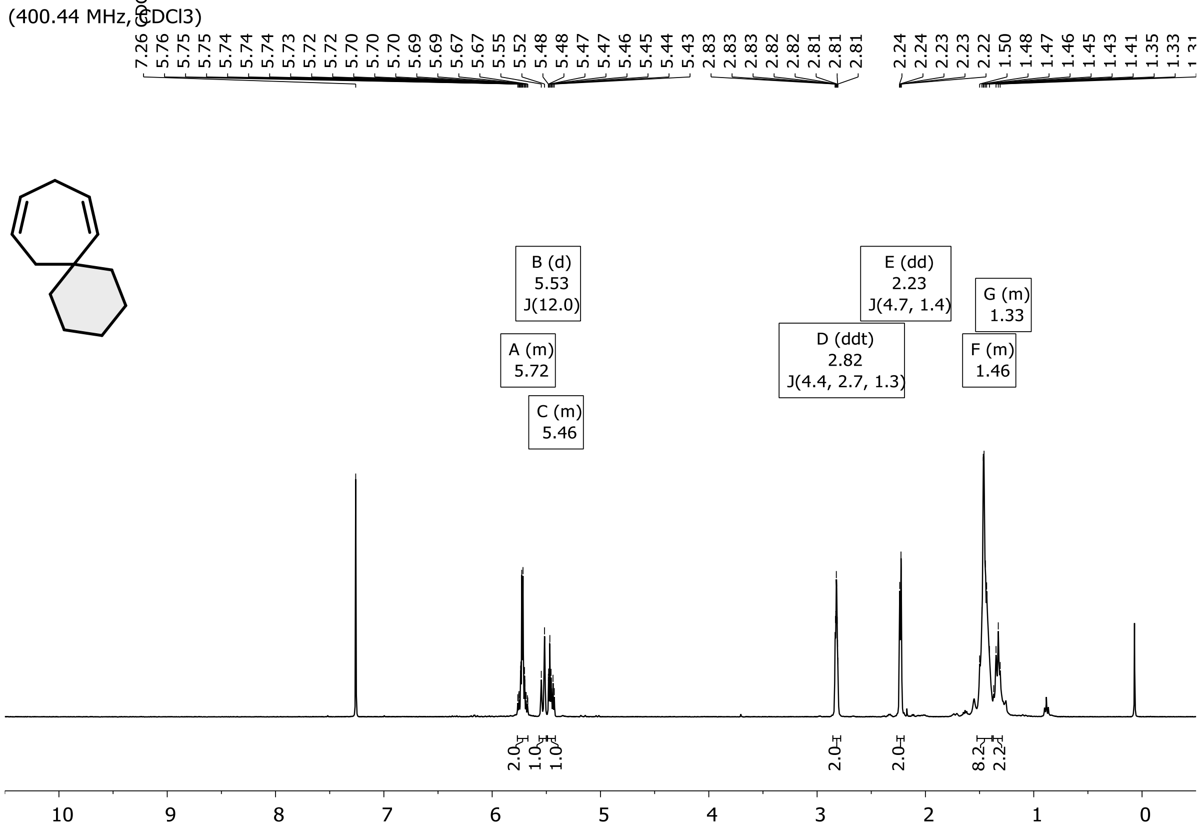

13C NMR
(100.70 MHz, CDCl3)

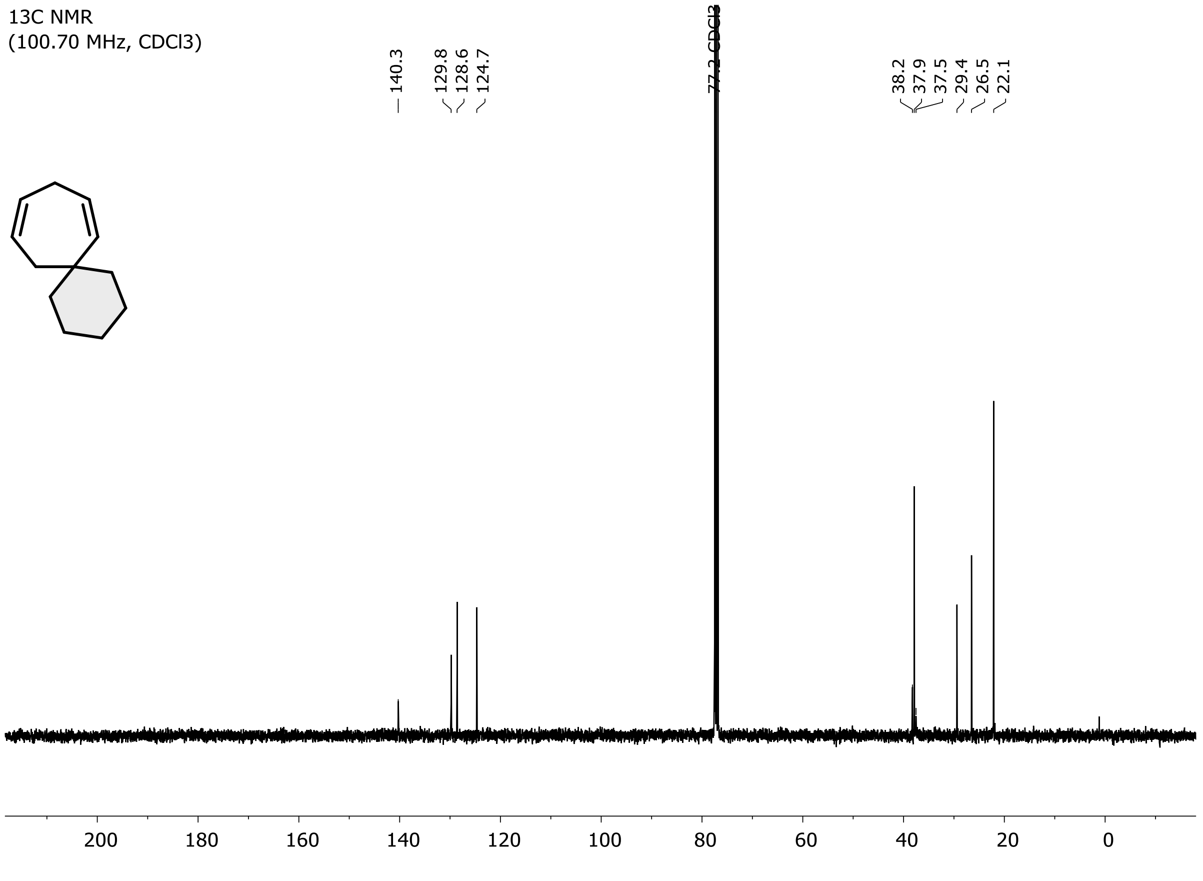

Cyclohexyl((*trans*)-2-vinylcyclopropyl)methanone

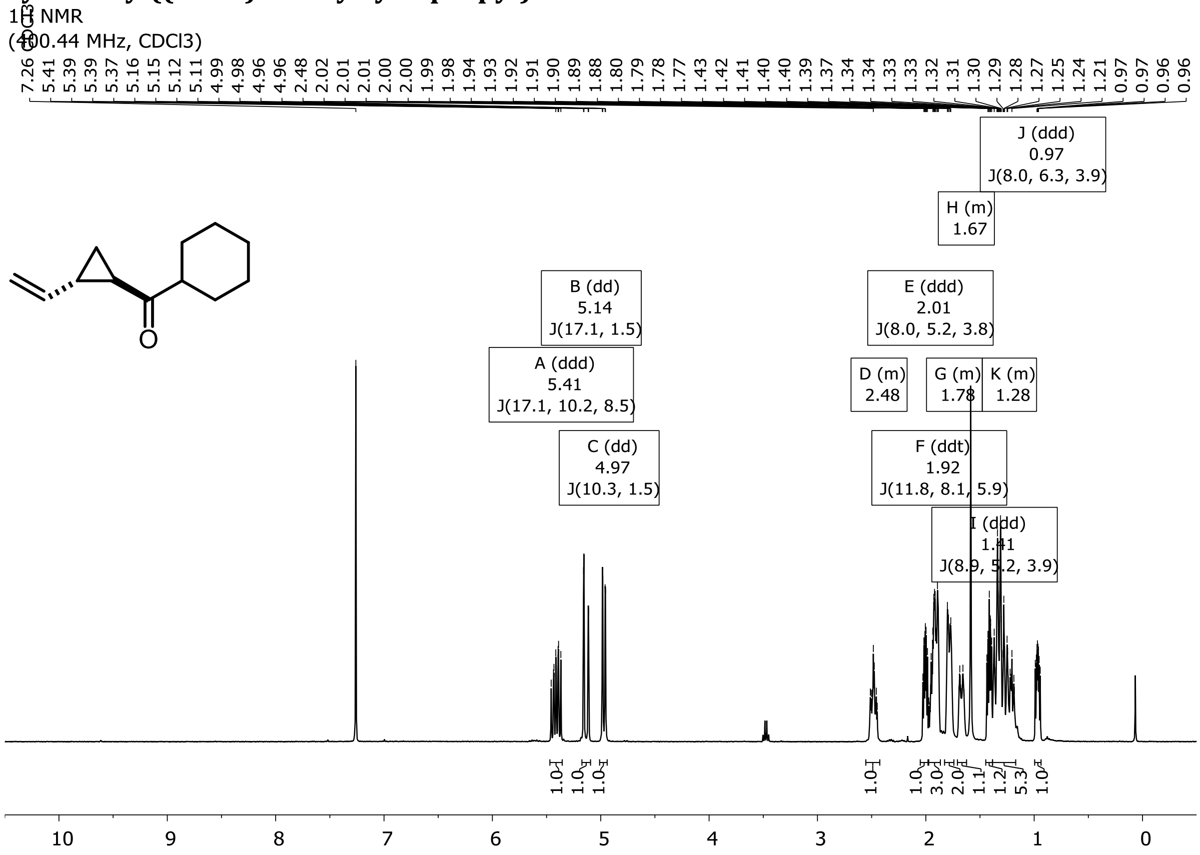

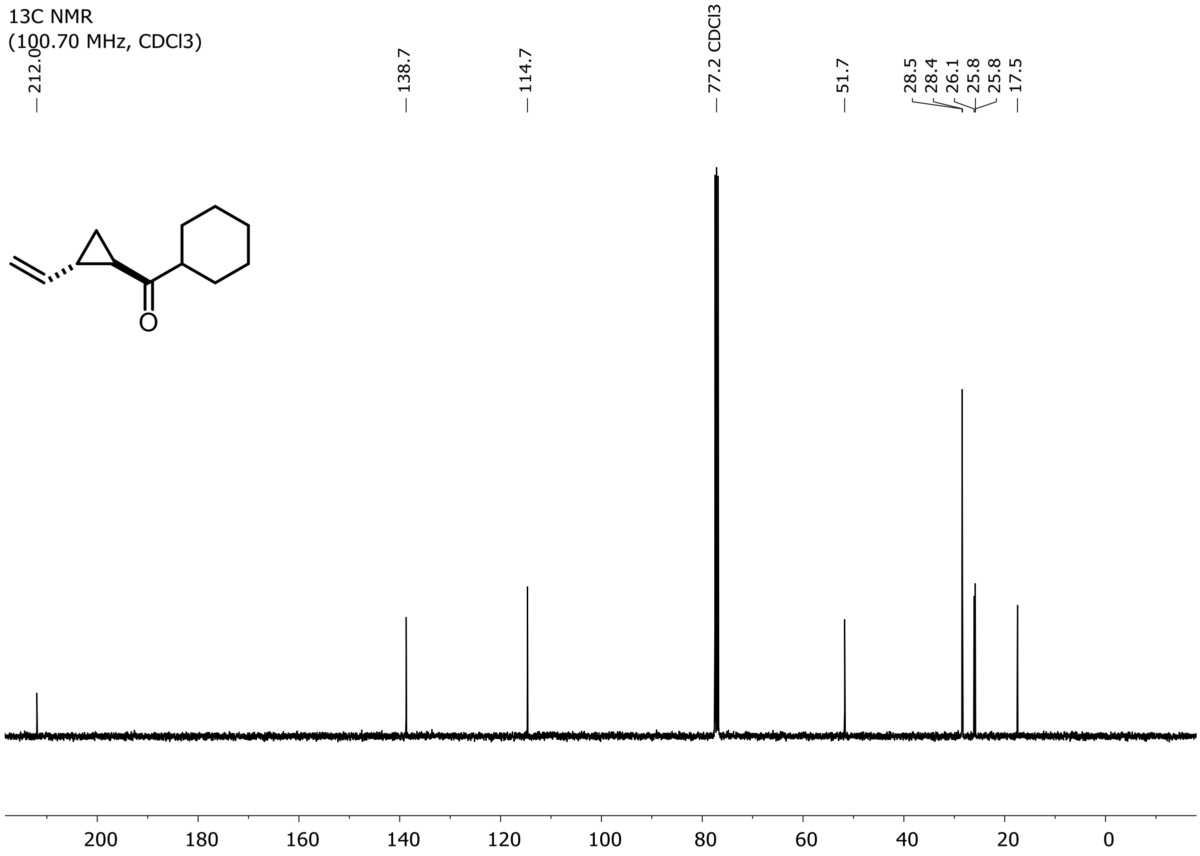

(Cyclohexylidene((*trans*)-2-vinylcyclopropyl)methoxy)trimethylsilane (S53)

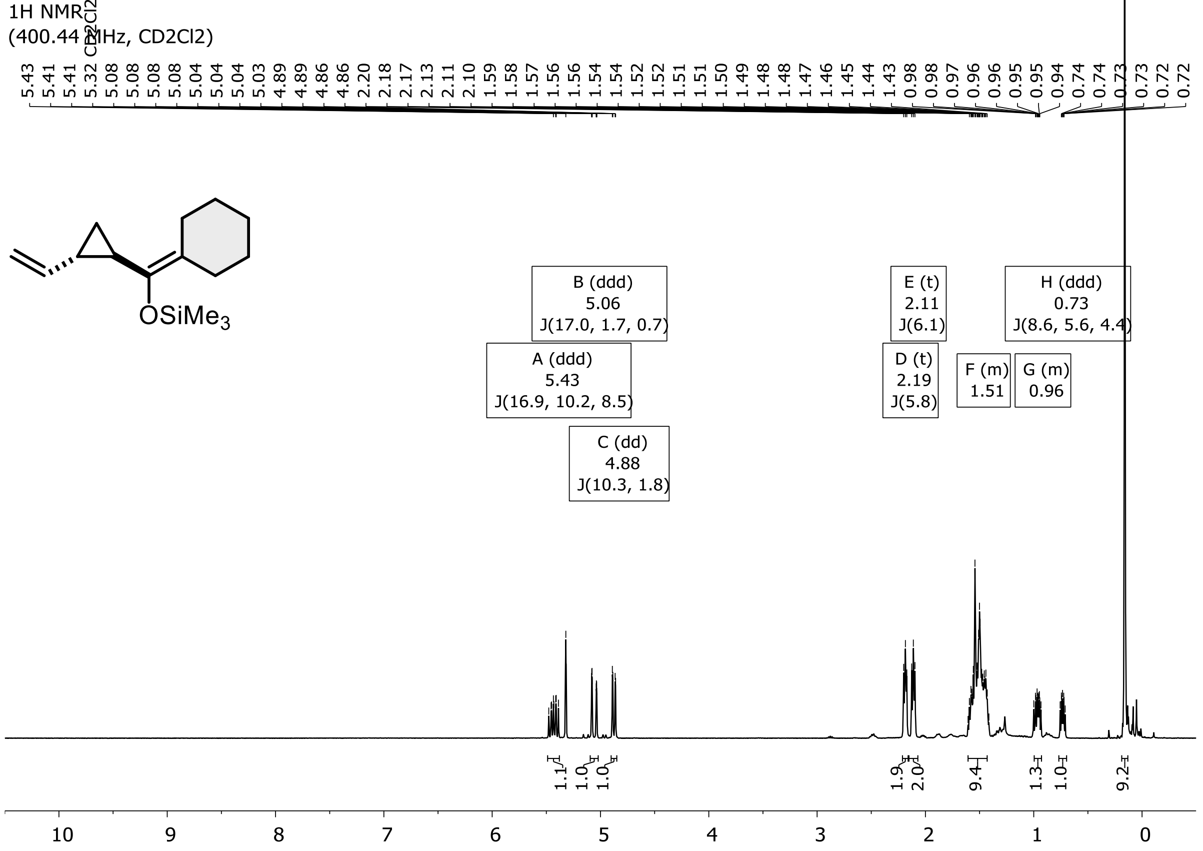

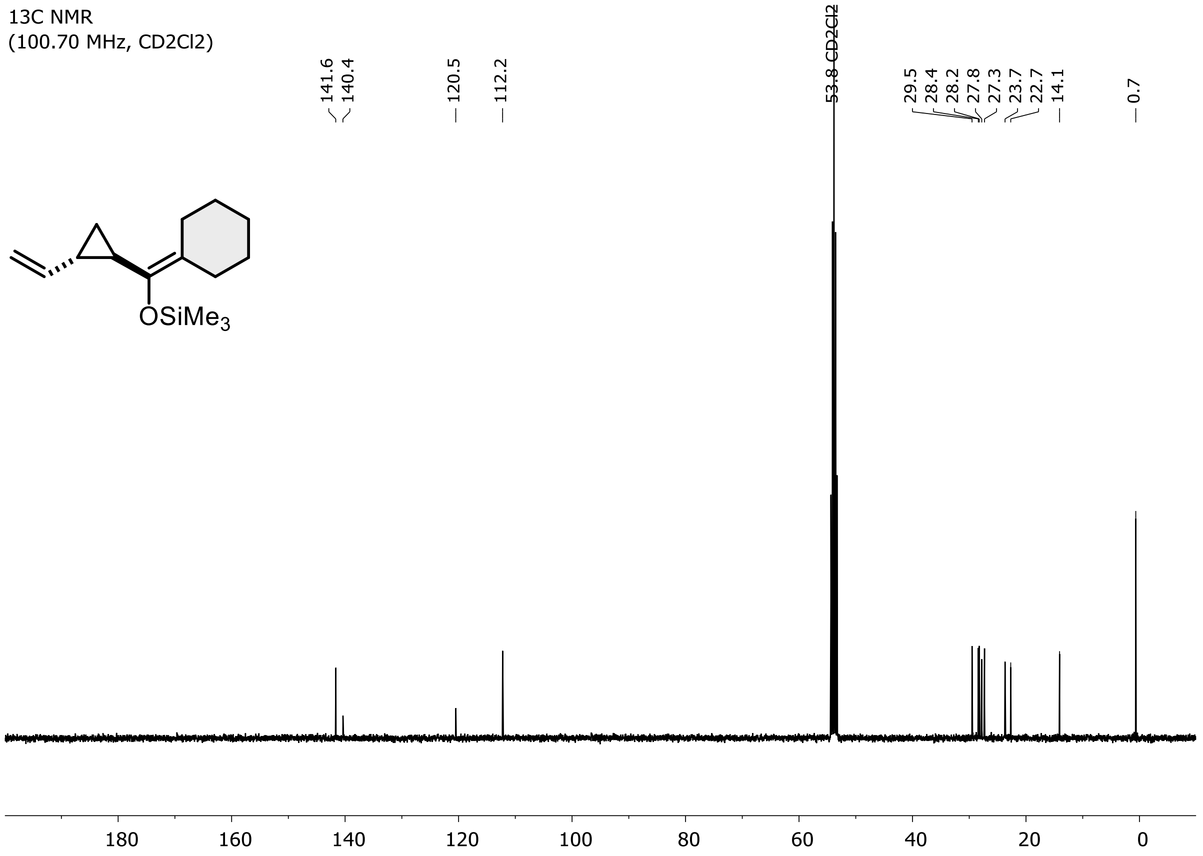

Spiro[5.6]dodec-10-en-7-one (54)

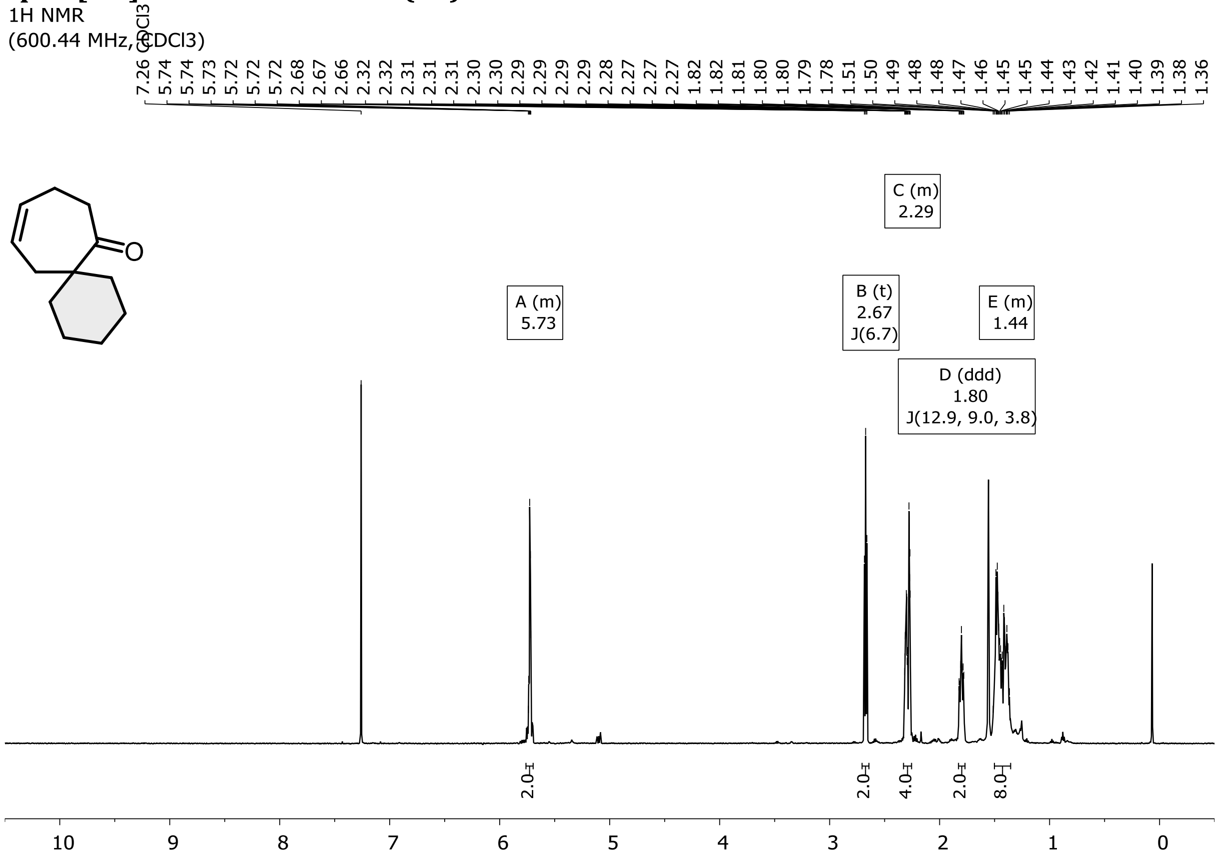

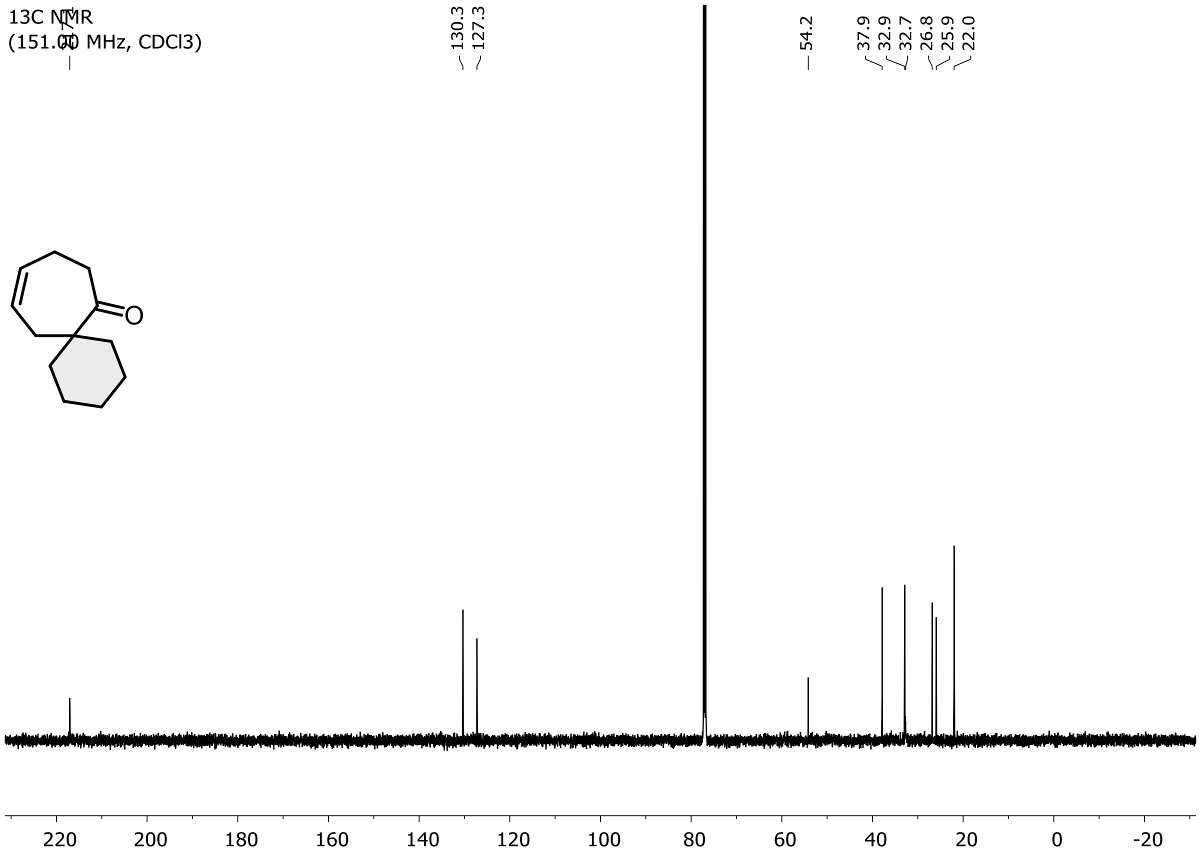

Cyclopentyl((*trans*)-2-vinylcyclopropyl)methanone

1H NMR
(400.44 MHz, CDCl3)

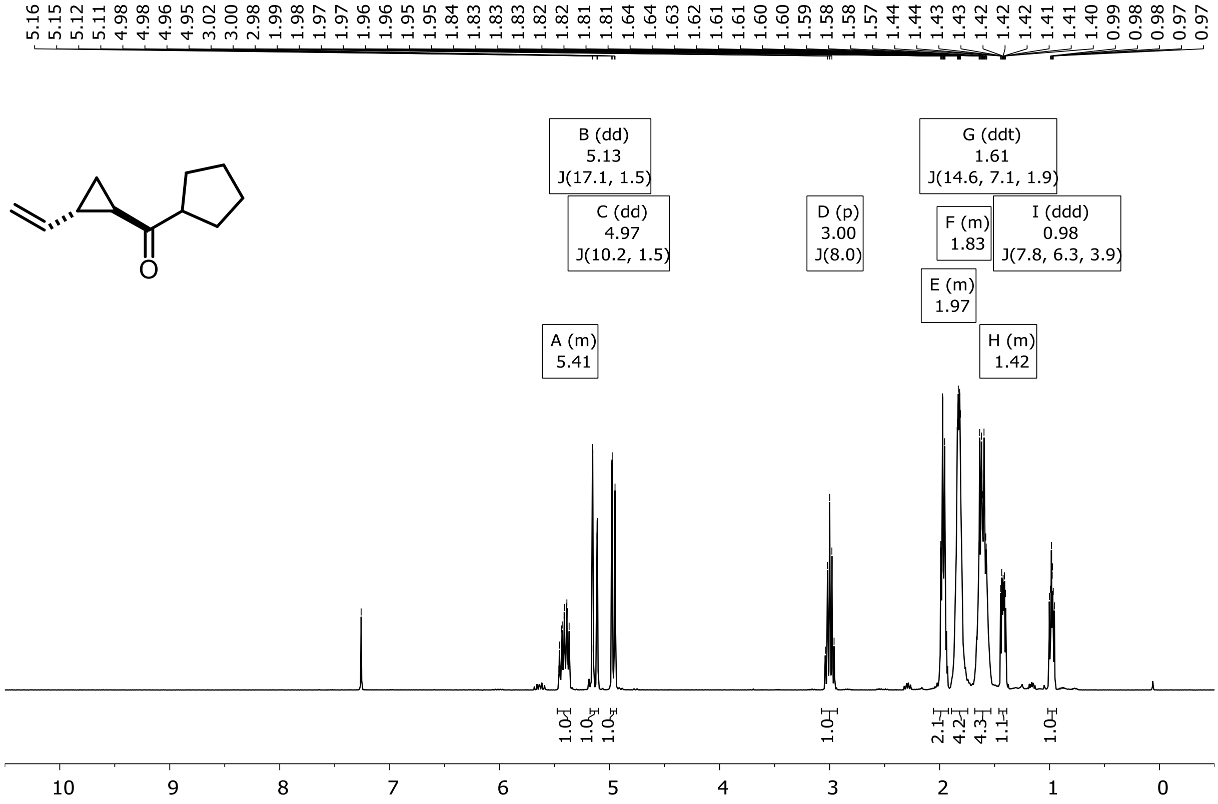

13C NMR
(100.70 MHz, CDCl3)

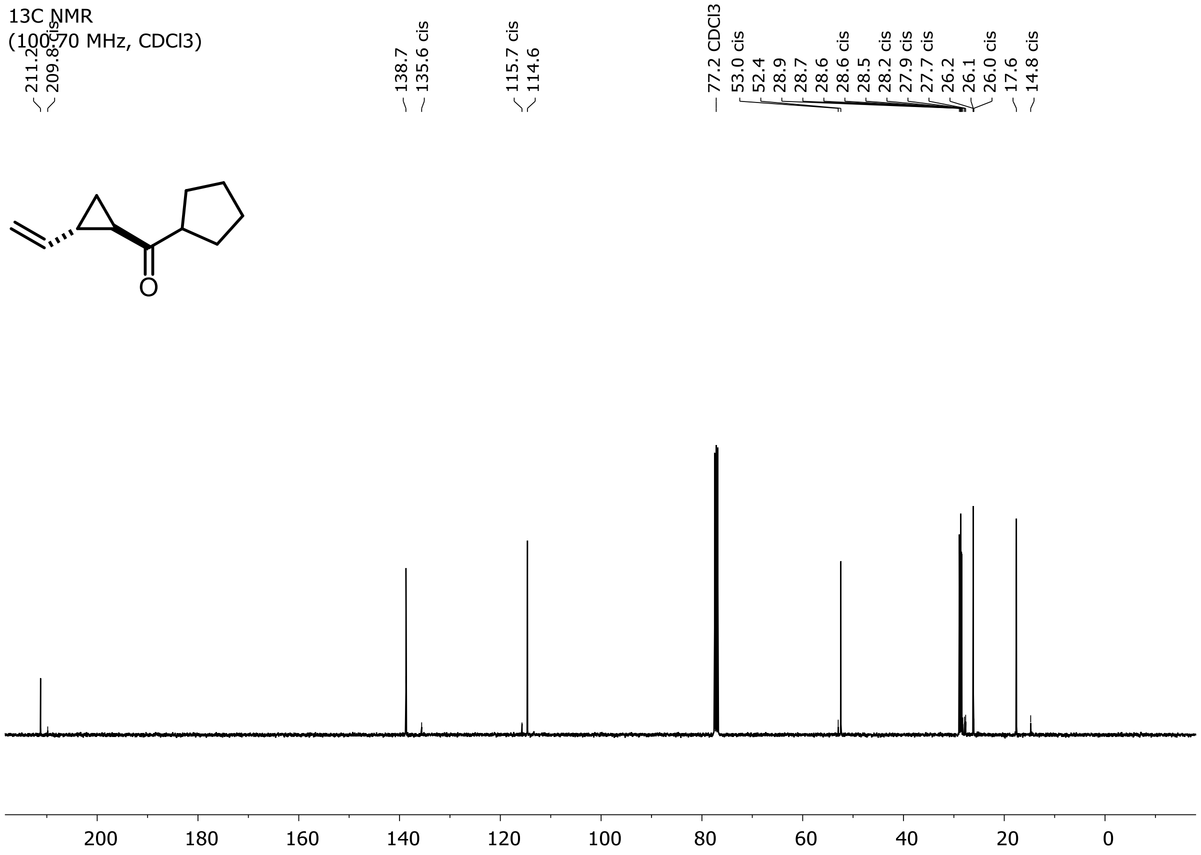

(Cyclopentylidene((*trans*)-2-vinylcyclopropyl)methoxy)triethylsilane (S55)

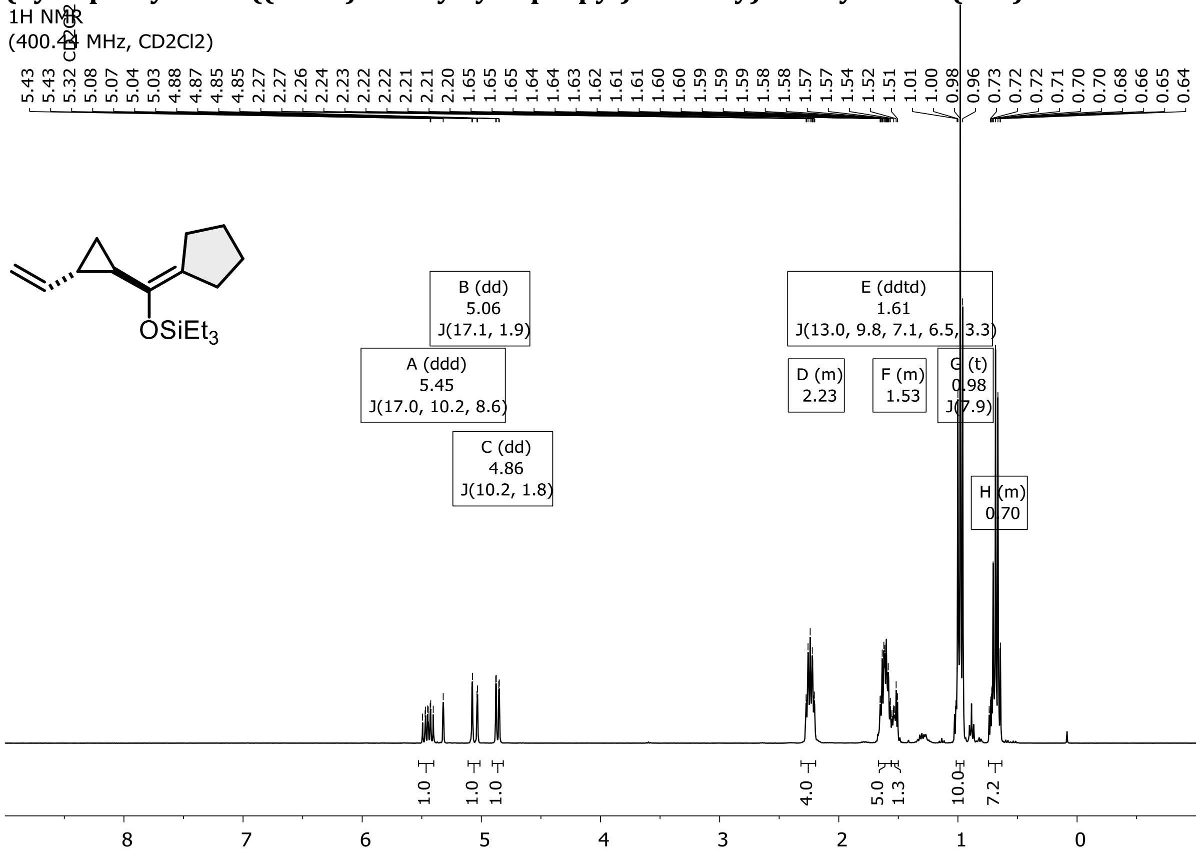

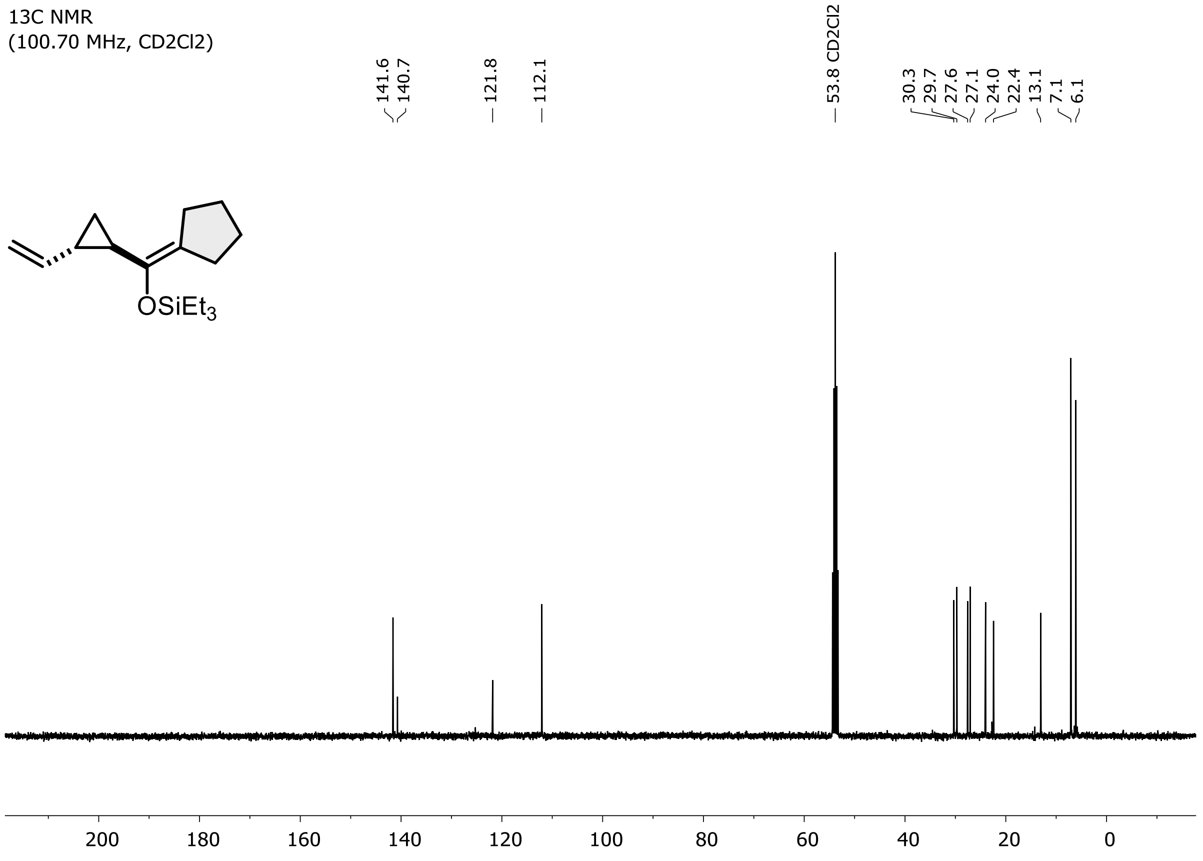

Spiro[4.6]undec-9-en-6-one (56)

1H NMR
(600.44 MHz, CDCl3)

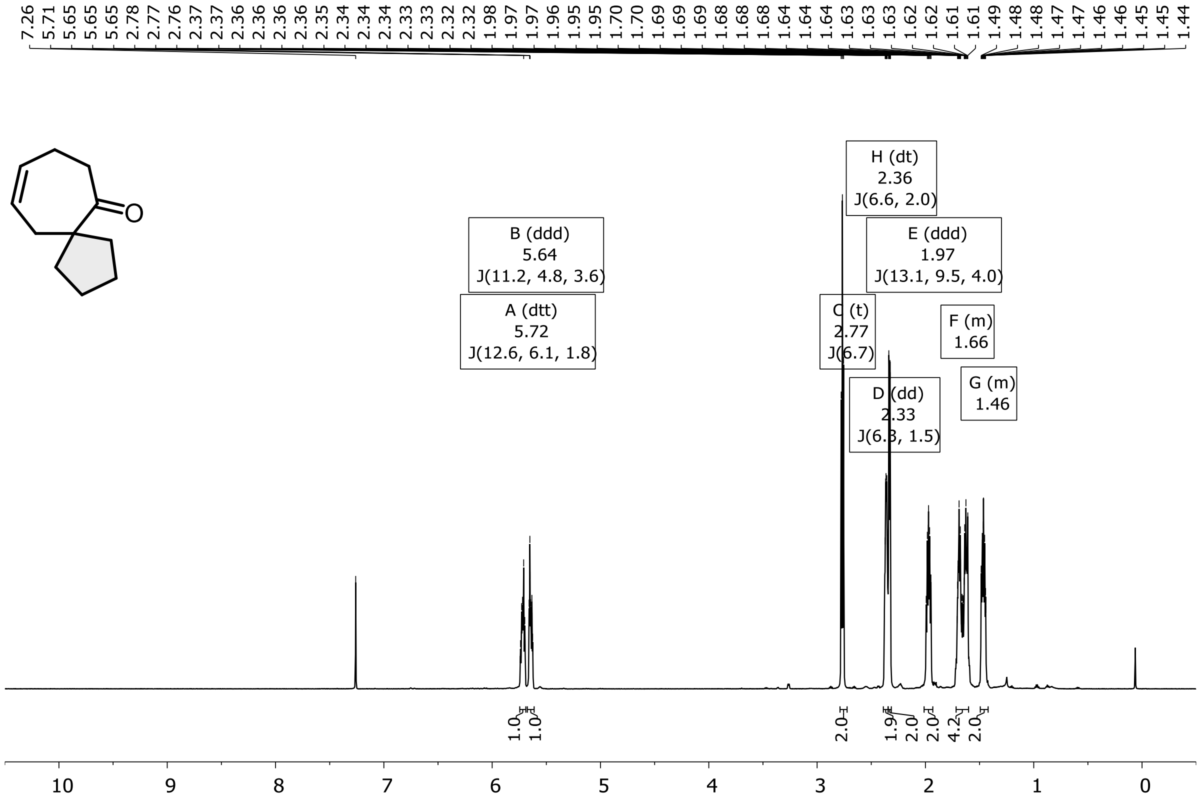

13C NMR
(151.00 MHz, CDCl3)

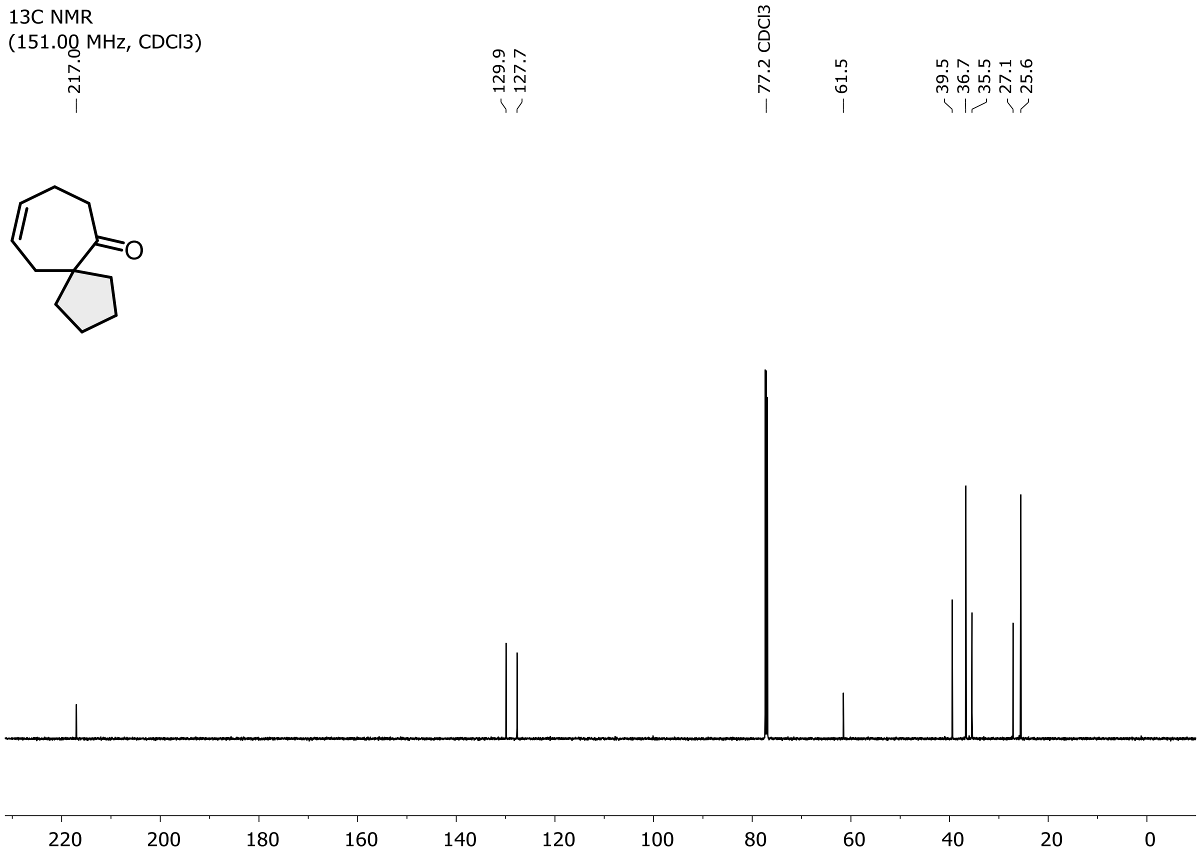

15. References

1. Skotnitzki, J. et al. Stereoselective Csp3–Csp2 Cross-Couplings of Chiral Secondary Alkylzinc Reagents with Alkenyl and Aryl Halides. *Angew. Chem. Int. Ed.* **59**, 320-324 (2020).
2. Lu, X.-L., Shannon, M., Peng, X.-S. & Wong, H. N. C. Stereospecific Iron-Catalyzed Carbon(sp2)–Carbon(sp3) Cross-Coupling with Alkyllithium and Alkenyl Iodides. *Org. Lett.* **21**, 2546-2549 (2019).
3. Suga, T., Takahashi, Y. & Ukaji, Y. One-Shot Radical Cross Coupling Between Benzyl Alcohols and Alkenyl Halides Using Ni/Ti/Mn System. *Adv. Synth. Catal.* **362**, 5622-5626 (2020).
4. Marino, J. P. & Nguyen, H. N. Bulky Trialkylsilyl Acetylenes in the Cadiot–Chodkiewicz Cross-Coupling Reaction. *The Journal of Organic Chemistry* **67**, 6841-6844 (2002).
5. Chanthamath, S., Phomkeona, K., Shibatomi, K. & Iwasa, S. Highly stereoselective Ru(ii)–Pheox catalyzed asymmetric cyclopropanation of terminal olefins with succinimidyl diazoacetate. *Chem. Commun.* **48**, 7750-7752 (2012).
6. Mendel, M., Gnagi, L., Dabranskaya, U. & Schoenebeck, F. Rapid and Modular Access to Vinyl Cyclopropanes Enabled by Air-stable Palladium(I) Dimer Catalysis. *Angew. Chem. Int. Ed.* **62**, e202211167 (2023).
7. Hofstra, J. L., Poremba, K. E., Shimozono, A. M. & Reisman, S. E. Nickel-Catalyzed Conversion of Enol Triflates into Alkenyl Halides. *Angew. Chem. Int. Ed.* **58**, 14901-14905 (2019).
8. Stoll, S. & Schweiger, A. EasySpin, a comprehensive software package for spectral simulation and analysis in EPR. *J. Magn. Reson.* **178**, 42-55 (2006).
9. Briggs, A. J. Improved Preparation of 1,3-Bis(2,6-di-iso-propylphenyl)imidazolium Tetrafluoroborate. *Synth. Commun.* **43**, 3258-3261 (2013).
10. Bantreil, X. & Nolan, S. P. Synthesis of N-heterocyclic carbene ligands and derived ruthenium olefin metathesis catalysts. *Nat. Protoc.* **6**, 69-77 (2011).
11. Dible, B. R., Sigman, M. S. & Arif, A. M. Oxygen-Induced Ligand Dehydrogenation of a Planar Bis-μ-Chloronickel(I) Dimer Featuring an NHC Ligand. *Inorg. Chem.* **44**, 3774-3776 (2005).
12. Matsubara, K. et al. Monomeric Three-Coordinate N-Heterocyclic Carbene Nickel(I) Complexes: Synthesis, Structures, and Catalytic Applications in Cross-Coupling Reactions. *Organometallics* **35**, 3281-3287 (2016).
13. Gallagher, W. P. & Vo, A. Dithiocarbamates: Reagents for the Removal of Transition Metals from Organic Reaction Media. *Org. Process Res. Dev.* **19**, 1369-1373 (2015).
14. Norbeck, D. W., Rosen, T. J. & Sham, H. L. Carbocyclic nucleoside analogs with antiviral activity. (1991).
15. Kirmse, W., van Chiem, P. & Henning, P.-G. The diazo route to 2-vinylcyclopropylidenes. *Tetrahedron* **41**, 1441-1451 (1985).
16. Murai, M., Mizuta, C., Taniguchi, R. & Takai, K. Synthesis of Borylcyclopropanes by Chromium-Promoted Cyclopropanation of Unactivated Alkenes. *Org. Lett.* **19**, 6104-6107 (2017).
17. Sayes, M., Benoit, G. & Charette, A. B. Three-Step Synthesis of 2-(Diiodomethyl)-4,4,5,5-tetramethyl-1,3,2-dioxaborolane from Dichloromethane. *Org. Synth.* **96**, 277-299 (2019).
18. Beaulieu, P. L. et al. Synthesis of (1R,2S)-1-Amino-2-vinylcyclopropanecarboxylic Acid Vinyl-ACCA) Derivatives: Key Intermediates for the Preparation of Inhibitors of the Hepatitis C Virus NS3 Protease. *The Journal of Organic Chemistry* **70**, 5869-5879 (2005).
19. Ning, Y. et al. Difluoroacetaldehyde N-Triftosylhydrazone (DFHZ-Tfs) as a Bench-Stable Crystalline Diazo Surrogate for Diazoacetaldehyde and Difluorodiazoethane. *Angew. Chem. Int. Ed.* **59**, 6473-6481 (2020).
20. Kidonakis, M. & Stratakis, M. Regioselective Diboration and Silaboration of Allenes Catalyzed by Au Nanoparticles. *ACS Catalysis* **8**, 1227-1230 (2018).
21. Neckebroeck, A., Kelly, S. M., Smith, B. O. & Clark, J. S. Synthesis of the Prototypical Cyclopropyl Dipeptide Mimic and Evaluation of Its Turn-Inducing Capability. *The Journal of Organic Chemistry* **87**, 258-270 (2022).
22. Kapat, A., Sperger, T., Guven, S. & Schoenebeck, F. *E*-Olefins through intramolecular radical relocation. *Science* **363**, 391-396 (2019).

23. Miyazaki, S., Koga, Y., Matsumoto, T. & Matsubara, K. A new aspect of nickel-catalyzed Grignard cross-coupling reactions: selective synthesis, structure, and catalytic behavior of a T-shape three-coordinate nickel(I) chloride bearing a bulky NHC ligand. *Chem. Commun.* **46**, 1932-1934 (2010).
24. Calow, A. D. J., Dailler, D. & Bower, J. F. Carbonylative N-Heterocyclization via Nitrogen-Directed C–C Bond Activation of Nonactivated Cyclopropanes. *J. Am. Chem. Soc.* **144**, 11069-11074 (2022).
25. Cheng, D., Huang, D. & Shi, Y. Synergistic effect of additives on cyclopropanation of olefins. *Organic & Biomolecular Chemistry* **11**, 5588-5591 (2013).
26. Bordeaux, M., Tyagi, V. & Fasan, R. Highly Diastereoselective and Enantioselective Olefin Cyclopropanation Using Engineered Myoglobin-Based Catalysts. *Angew. Chem. Int. Ed.* **54**, 1744-1748 (2015).
27. Charette, A. B., Molinaro, C. & Brochu, C. Catalytic Asymmetric Cyclopropanation of Allylic Alcohols with Titanium-TADDOLate: Scope of the Cyclopropanation Reaction. *J. Am. Chem. Soc.* **123**, 12168-12175 (2001).
28. Rachwalski, M., Kaczmarczyk, S., Leśniak, S. & Kiełbasiński, P. Highly Efficient Asymmetric Simmons–Smith Cyclopropanation Promoted by Chiral Heteroorganic Aziridinyl Ligands. *ChemCatChem* **6**, 873-875 (2014).
29. Radi, M. et al. Discovery of Chiral Cyclopropyl Dihydro-Alkylthio-Benzyl-Oxopyrimidine (S-DABO) Derivatives as Potent HIV-1 Reverse Transcriptase Inhibitors with High Activity Against Clinically Relevant Mutants. *J. Med. Chem.* **52**, 840-851 (2009).
30. Melancon, B. J., Perl, N. R. & Taylor, R. E. Competitive Cationic Pathways and the Asymmetric Synthesis of Aryl-Substituted Cyclopropanes. *Org. Lett.* **9**, 1425-1428 (2007).
31. Montesinos-Magraner, M. et al. General Cyclopropane Assembly by Enantioselective Transfer of a Redox-Active Carbene to Aliphatic Olefins. *Angew. Chem. Int. Ed.* **58**, 5930-5935 (2019).
32. Itoh, T., Inoue, H. & Emoto, S. Synthesis of Dictyopterene A: Optically Active Tributylstannylcyclopropane as a Chiral Synthon. *Bull. Chem. Soc. Jpn.* **73**, 409-416 (2000).
33. Jaenicke, L., Akintobi, T. & Marner, F.-J. Ein Beitrag zur Darstellung von Alkyl-cycloheptadienen: Synthese von Ectocarpen und seinen Homologen. *Justus Liebigs Ann. Chem.* **1973**, 1252-1262 (1973).
34. Moore, R. E., Pettus, J. A., Jr. & Mistysyn, J. Odoriferous C11 hydrocarbons from Hawaiian Dictyopteris. *J. Org. Chem.* **39**, 2201-2207 (1974).
35. Moore, R. E. & Pettus, J. A., Jr. Isolation and structure determination of dictyopterenes C' and D' from Dictyopteris. Stereospecificity in the cope rearrangement of dictyopterenes A and B. *J. Am. Chem. Soc.* **93**, 3087-3088 (1971).
36. Moore, R. E. Volatile compounds from marine algae. *Acc. Chem. Res.* **10**, 40-47 (1977).
37. Gaussian 16, Revision A.03 (Gaussian, Inc., Wallingford, CT, 2016).
38. Yu, H. S., He, X., Li, S. L. & Truhlar, D. G. MN15: A Kohn–Sham global-hybrid exchange–correlation density functional with broad accuracy for multi-reference and single-reference systems and noncovalent interactions. *Chem. Sci.* **7**, 5032-5051 (2016).
39. Zhao, Y. & Truhlar, D. G. A new local density functional for main-group thermochemistry, transition metal bonding, thermochemical kinetics, and noncovalent interactions. *J. Chem. Phys.* **125**, 194101 (2006).
40. Neese, F. Software update: The ORCA program system—Version 5.0. *WIREs Computational Molecular Science* **12**, e1606 (2022).
41. Pettersen, E. F. et al. UCSF Chimera—A visualization system for exploratory research and analysis. *J. Comput. Chem.* **25**, 1605-1612 (2004).
42. Lee, C. H., Lutterman, D. A. & Nocera, D. G. Photoactivation of metal–halogen bonds in a Ni(ii) NHC complex. *Dalton Trans.* **42**, 2355-2357 (2013).
43. Ye, S. & Neese, F. Accurate Modeling of Spin-State Energetics in Spin-Crossover Systems with Modern Density Functional Theory. *Inorg. Chem.* **49**, 772-774 (2010).
44. Kwon, D.-H. et al. Catalytic Dinuclear Nickel Spin Crossover Mechanism and Selectivity for Alkyne Cyclotrimerization. *ACS Catal.* **7**, 4796-4804 (2017).
45. Dürr, A. B. et al. Divergent Reactivity of a Dinuclear (NHC)Nickel(I) Catalyst versus Nickel(0) Enables Chemoselective Trifluoromethylselenolation. *Angew. Chem. Int. Ed.* **56**, 13431-13435 (2017).
